# A Unified Approach to Polycyclic Alkaloids of the Ingenamine Estate: Total Syntheses of Keramaphidin B, Ingenamine, and Nominal Njaoamine I

**DOI:** 10.1021/jacs.1c07955

**Published:** 2021-08-27

**Authors:** Zhanchao Meng, Simon M. Spohr, Sandra Tobegen, Christophe Farès, Alois Fürstner

**Affiliations:** Max-Planck-Institut für Kohlenforschung, 45470 Mülheim/Ruhr, Germany

## Abstract

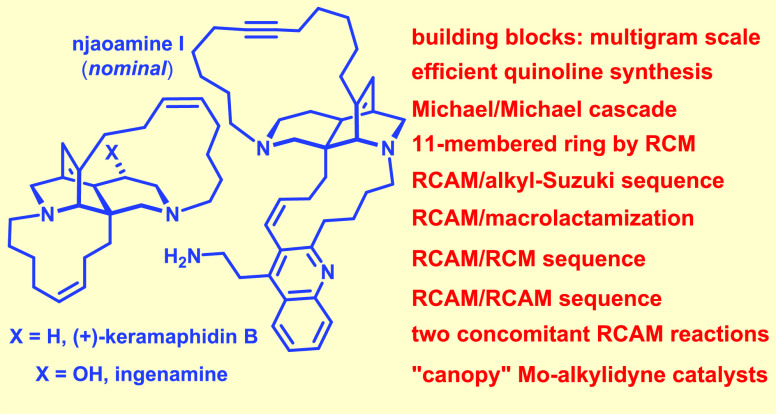

Many polycyclic marine alkaloids are thought to derive from partly reduced macrocyclic alkylpyridine derivatives via a transannular Diels–Alder reaction that forms their common etheno-bridged diaza-decaline core (“Baldwin–Whitehead hypothesis”). Rather than trying to emulate this biosynthesis pathway, a route to these natural products following purely chemical logic was pursued. Specifically, a Michael/Michael addition cascade provided rapid access to this conspicuous tricyclic scaffold and allowed different handles to be introduced at the bridgehead quarternary center. This flexibility opened opportunities for the formation of the enveloping medium-sized and macrocyclic rings. Ring closing alkyne metathesis (RCAM) proved most reliable and became a recurrent theme en route to keramaphidin B, ingenamine, xestocyclamine A, and nominal njaoamine I (the structure of which had to be corrected in the aftermath of the synthesis). Best results were obtained with molybdenum alkylidyne catalysts endowed with (tripodal) silanolate ligands, which proved fully operative in the presence of tertiary amines, quinoline, and other Lewis basic sites. RCAM was successfully interlinked with macrolactamization, an intricate hydroboration/protonation/alkyl-Suzuki coupling sequence, or ring closing olefin metathesis (RCM) for the closure of the second lateral ring; the use of RCM for the formation of an 11-membered cycle is particularly noteworthy. Equally rare are RCM reactions that leave a pre-existing triple bond untouched, as the standard ruthenium catalysts are usually indiscriminative vis-à-vis the different π-bonds. Of arguably highest significance, however, is the use of two consecutive or even concurrent RCAM reactions en route to nominal njaoamine I as the arguably most complex of the chosen targets.

## Introduction

**Scheme 1:**
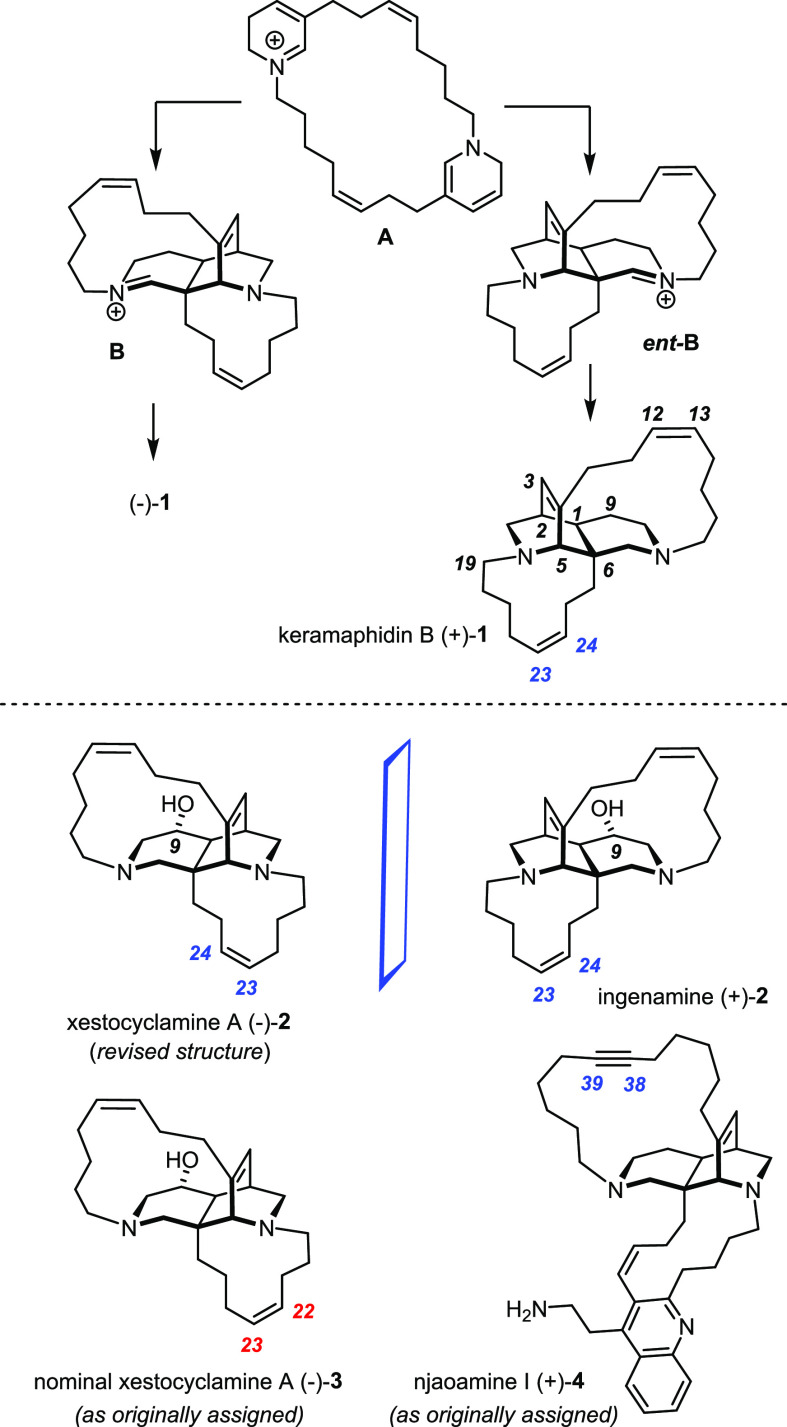
Key Step of the Proposed Biosynthesis of Keramaphidin B; Representative Alkaloids Thought To Originate from Similar Pathways

In a recent Communication we reported the first total syntheses of ingenamine and nominal xestocyclamine A.^1^ These polycyclic alkaloids had originally been proposed to be “pseudoenantiomeric” to each other, differing in the exact positioning of the double bond embedded into the 11-membered ring (Scheme 1).^2−4^ Our data, however, provided compelling evidence that the originally assigned structure of xestocyclamine A needs to be corrected in exactly this detail: natural xestocyclamine A ((−)-**2**) and ingenamine ((+)-**2**) are in fact almost certainly true enantiomers.^1^

At the meta-level, this conclusion is not all that surprising in view of the proposed biosynthesis of these and related alkaloids. It has long been speculated that the gambit of the famous “Baldwin–Whitehead pathway” might not be enzyme-dependent:^5,6^ it consists of a transannular Diels–Alder reaction of a partly reduced macrocyclic dipyridine derivative of type **A**, which affords the enantiomeric iminium ions **B** and *ent*-**B** in the first place; reduction leads to keramaphidin B (**1**), which indeed occurs in nature in both enantiomeric forms.^4,7−9^ Although this observation strongly suggests that the initial [4 + 2] cycloaddition proceeds without intervention of a biocatalyst, attempts at emulating this step in the laboratory met with limited success in that the yield of (±)-**1** was minute (0.2–0.3%), despite considerable experimentation.^10^ Even a more conventional approach based on an *intermolecular* Diels–Alder reaction followed by two concurrent macrocyclizations via ring closing olefin metathesis (RCM) was very low yielding (1–2%).^11^

Studies toward keramaphidin B^12−15^ as well as (nominal) xestocyclamine A^16,17^ that were not intending to emulate the proposed biosynthesis have also been reported.^18−20^ The different strategies notwithstanding, none of the approaches reached these targets in the end. This is also why the originally mis-assigned structure of xestocyclamine A^2^ went unrecognized for over 25 years.

**Scheme 2:**
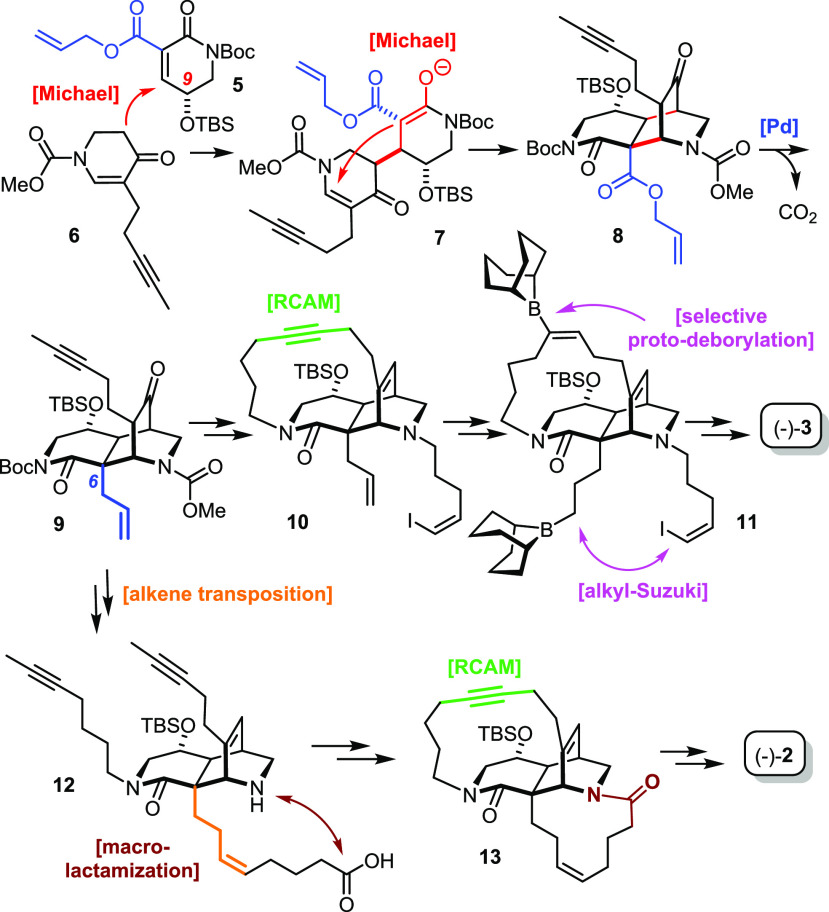
Executive Summary of the “First-Generation” Approach

Our successful foray differed from this literature precedent in that the 1,4-etheno-bridged 2,7-diazadecalin core was formed by two consecutive Michael addition reactions, which we had hoped would proceed in one pot but ultimately had to be carried out in a stepwise manner.^1,21,22^ For the closure of the enveloping medium-sized and macrocyclic rings, we opted for methods that are strictly orthogonal in chemical terms (Scheme 2). This decision paid valuable dividends, as it ultimately allowed compound **9** to be used as a common intermediate for the total synthesis of nominal xestocyclamine A ((−)-**3**) and *ent*-ingenamine (that is, natural xestocyclamine A ((−)-**2**)) alike. Specifically, ring closing alkyne metathesis (RCAM)^23−25^ allowed the 13-membered cycle to be forged before an involved maneuver merging hydroboration/protonation with an alkyl-Suzuki coupling^26,27^ was used to close the yet missing 11-membered ring of (−)-**3**. In the case of (−)-**2**, an inverse order was successful in that macrolactamization with formation of the medium-sized ring preceded macrocyclizaton via RCAM.^1^

With the first total syntheses of nominal xestocyclamine A ((−)-**3**) and (−)-ingenamine ((−)-**2**) completed and the likely misassignment of the former corrected,^1^ we revisited this project altogether with the intention of rendering the individual syntheses even more productive and the underlying blueprint more comprehensive. To this end, the following chemical, tactical, and strategic issues had to be addressed: (i) although the building blocks **5** and **6** serving as Michael acceptor and donor for the preparation of the central core could be made on decagram scale, the yields were moderate; (ii) an upgrade of the stepwise Michael/Michael addition sequence to a true reaction cascade should be aimed for;^28,29^ (iii) an extension to members of the opposite enantiomeric series is desirable to provide material for future biological testing; (iv) the original route allowed only an allyl substituent to be placed at the bridgehead C6-position of the 2,7-diazadecalin core by means of a palladium catalyzed decarboxylative allylation (**8** → **9**);^30,31^ the ability to install other handles would increase the flexibility during the subsequent macrocyclization phase and hence expand the reach and scope of the chosen approach;^32^ (v) the same is true if a larger panel of chemically orthogonal macrocyclization reactions could be utilized; (vi) in parallel work, a new class of alkyne metathesis catalysts was developed in our laboratory that hold the promise of working well in the presence of amines and basic heterocyclic motifs;^33−37^ the ingenamine estate of alkaloids provides an arguably stringent testing ground.^38−46^

All of these aspects have been successfully addressed as manifested in a second-generation synthesis of natural (+)-ingenamine (as the enantiomer of xestocyclamine A), the first highly efficient conquest of keramaphidin B after the only low-yielding biomimetic approaches cited above,^10,11^ and the first total synthesis of a member of the njaoamine family, even though a subtle misassignment of the originally proposed structure was noticed and corrected.^47−49^ This class of marine alkaloids, though obviously related to ingenamine/xestocylamine A, is considerably more challenging in synthetic terms for it contains an additional side chain terminating in a primary amine as well as a quinoline moiety annulated to the macrocyclic ring.

## Results and Discussion

### Building Blocks and the Michael/Michael Cascade Revisited

Our initial design had tried to match the reactivity of the Michael acceptor and donor in the best possible way (Scheme 2).^1^ To this end, the highly electrophilic alkylidene β-ketoester **5** was deemed optimal, as it was expected to render the first Michael addition particularly facile;^50^ at the same time, the malonate-type anion **7** primarily formed should expedite the subsequent intramolecular Michael addition that closes the diaza-tricyclic core of **8**.^51^ An allyl ester was chosen as the exocyclic activating group in **5**, as it lends itself to decarboxylative allylation with formation of the required quarternary C6-bridgehead position.^30,31,52,53^

Although successfully reduced to practice on gram scale, we ultimately found this setting suboptimal. The fact that the formation of the 2,7-diazadecaline core had to be carried out in a stepwise manner was tentatively attributed to the fact that enolate **7** derived from a 1,3-dicarbonyl derivative is actually too stabilized. Although it likely engages in the second Michael addition, it also seems to be too good a leaving group; as such, it renders this step reversible, thus preventing an efficient cyclization cascade from occurring.^1^ Moreover, one might want to revisit the choice of the allyl ester: although the palladium-catalyzed decarboxylative allylation worked perfectly well in terms of yield and selectivity, this reaction is limiting in conceptual terms, as it does not allow other substituents to be attached to the bridgehead position. This handicap had already surfaced in our original campaign: while the allyl handle was ideal for the synthesis of *nominal* xestocyclamine A ((−)-**3**) via hydroboration/cross coupling, a stepwise transposition of the double bond by hydroboration/oxidation and subsequent Wittig olefination with formation of **12** was necessary on the way to *actual* xestocyclamine A ((−)-**2**).^1^

**Scheme 3:**
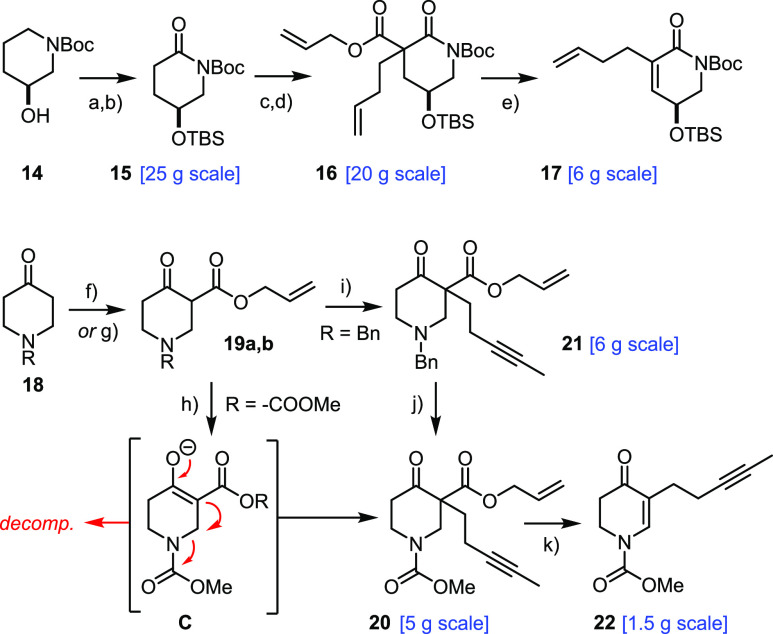
Building Blocks (Ingenamine Series) Reagents and conditions: (a) TBSCl, Et_3_N, CH_2_Cl_2_, quant.; (b) RuO_2_ (6 mol %), NaIO_4_, EtOAc/H_2_O, 55%; (c) LiHMDS, allyl chloroformate, THF, −78 °C, 94%; (d) 4-bromo-1-butene, Cs_2_CO_3_, DMF, 94%; (e) Pd_2_(dba)_3_·CHCl_3_ (5 mol %), MeCN, reflux, 83%; (f) LiHMDS, allyl chloroformate, toluene, −78 °C → 0 °C, 50% (R = COOMe); (g) NaH, diallyl carbonate, THF, 45% (R = Bn); (h) 1-iodo-3-pentyne, K_2_CO_3_, acetone, reflux, 36%; (i) 1-iodo-3-pentyne, Cs_2_CO_3_, DMF, 91%; (j) ClCOOMe, toluene, reflux, quant.; (k) Pd_2_(dba)_3_·CHCl_3_ (5 mol %), MeCN, reflux, 96%

In an attempt to remedy these issues, to save steps in the longest linear sequence, and gain higher flexibility at the same time, it was decided to include the appropriate handle for macrocyclization in the Michael acceptor from the very beginning. For proof-of-concept, compound **17** carrying a butenyl substituent was prepared by O-silylation of commercial **14** followed by regioselective C–H oxidation with RuO_2_ cat./NaIO_4_ (Scheme 3).^1,54^ The elaboration of **15** thus formed into **16** was also high yielding. A particularly noteworthy improvement concerns the use of a modified Saegusa-type decarboxylative dehydrogenation catalyzed by Pd_2_(dba)_3_ to set the internal double bond of the Michael acceptor **17**;^55^ the formation of the original building block **5**, which is much more electrophilic and hence more sensitive, had mandated stoichiometric selenation/selenoxide elimination for this purpose.^1^

The preparation of the required Michael donor was also much improved in practical terms. Direct alkylation of **19a** (R = −COOMe), as previously described, does work on scale but is rather inefficient (36% yield);^1^ this poor outcome is attributed to the good leaving group properties of the carbamate adjacent to the enolate **C** formed upon deprotonation. To circumvent this problem, the alkylation was carried out with the N-Bn protected derivative **19b**, which, indeed, led to a much more favorable outcome. To prevent the basic amine site from interfering with any downstream process, most notably the palladium catalyzed Saegusa oxidation, **21** was reacted with ClC(=O)OMe in refluxing toluene: under these conditions, the benzyl group is cleanly swapped for the carbamate and the stage set for yet another palladium-catalyzed decarboxylative dehydrogenation.^55^ This sequence to the desired fragment **22** is considerably more efficient than the original route. In addition, it is flexible with regard to the side chain; this aspect is best illustrated by the total synthesis of nominal njaoamine I outlined below, which would not have been successful otherwise.

**Scheme 4:**
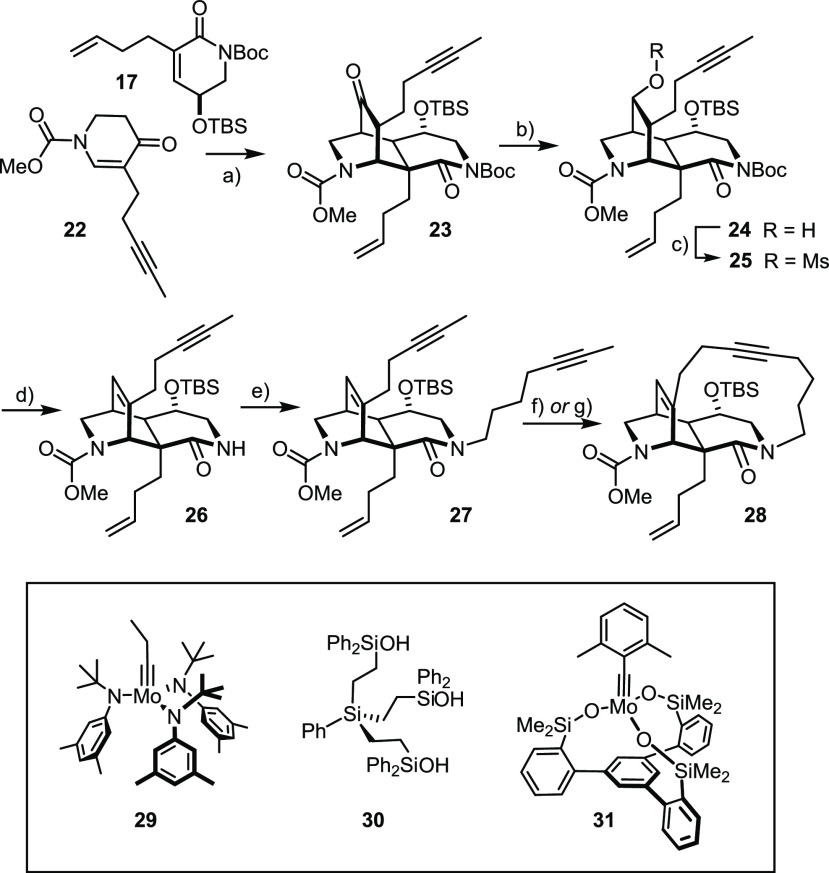
Michael/Michael Cascade and Further Elaboration Reagents and conditions: (a) (i) *t*BuOLi, THF, −50 °C → RT; (ii) Boc_2_O, DMAP; (b) NaBH_4_, MeOH, 0 °C, 53% (over two steps); (c) MsCl, Et_3_N, DMAP, CH_2_Cl_2_, 0 °C → RT, 91%; (d) (i) 2,6-lutidine, 170 °C; (ii) TBSOTf, CH_2_Cl_2_, 73%; (e) 1-iodo-5-heptyne, NaH, DMF, 0 °C, 95%; (f) **29** (25 mol %), **30** (30 mol %), toluene, MS 5 Å, 100 °C, 79%; (g) **31** (20 mol %), toluene, MS 5 Å, reflux, 83% (1.3 g scale).

Gratifyingly, the redesigned building blocks could be coaxed to participate in a true Michael/Michael cascade (Scheme 4).^28,29^ After some experimentation, it was found that the reaction is best performed with LiO*t*Bu as the base; although the N-Boc group was also cleaved, the desired diaza-tricyclic product **23** was the only discrete isomer detected in the crude material (after reprotection); reduction with NaBH_4_ furnished alcohol **24** as a single isomer, which is more polar than **23** and hence easier to purify by flash chromatography. This key compound was isolated in analytically pure form in 53% yield over two steps (740 mg scale, single largest batch), which marks yet another significant improvement over our original foray.^1^ The base-induced elimination of the derived mesylate **25** required harsh conditions but proceeded cleanly; as the −NBoc group was concomitantly cleaved, the stage was nicely set for subsequent N-alkylation of **26** with formation of **27**.

**Figure 1:**
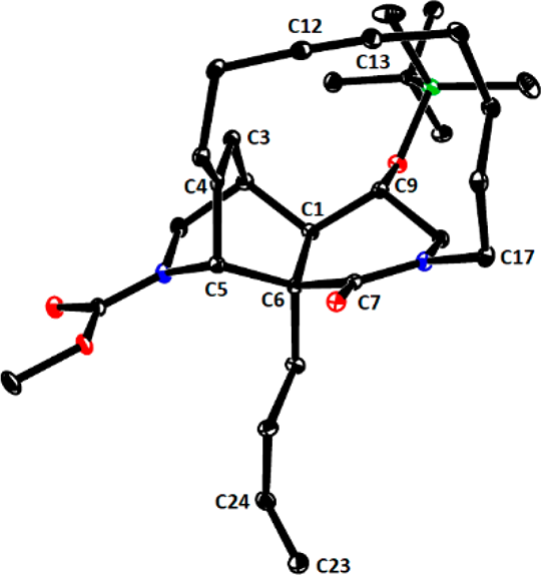
Structure of cycloalkyne **28** in the solid state.

As expected, this substrate readily succumbed to RCAM when treated with a catalyst generated by mixing complex **29** and trisilanol **30**, which had also served our original study in this field.^56−58^ Although the true nature of the active species generated in situ is unknown, all evidence suggests that the chosen silanolate ligand partly cross-links the active metal fragments; the resulting mixture of (cyclo)oligomeric alkylidynes effects the desired transformation. Gratifyingly, we found that the newly developed and molecularly well-defined molybdenum alkylidyne complex **31** distinguished by a tripodal silanolate ligand sphere is at least equipotent.^33−35^ Although a fairly high loading and rather forcing conditions proved necessary to overcome the strain of the incipient tetracyclic scaffold, cycloalkyne **28** was obtained in 83% yield on a 1.3 g scale (for reaction optimization, see the Supporting Information (SI)); its constitution and stereostructure were unambiguously established by 2D NMR spectroscopy and X-ray diffraction (Figure 1).

### Second Generation Synthesis of (+)-Ingenamine

With a short, efficient, and scalable route to **28** secured, we were in the favorable position to explore new ways of forging the yet missing medium-sized ring en route to the ingenamine estate of alkaloids. Most notably, alternatives to the alkyl-Suzuki coupling used in the original synthesis of (−)-**3** or the more conventional macrolactamization en route to (−)-**2** seemed worth investigating.^1,32^

The unsaturated handle branching off the bridgehead in **28** invited the use of ring closing olefin metathesis (RCM),^59−61^ even though the number of successful applications to 11-membered carbo- and heterocycles is comparatively small and the yields were rather moderate in many cases.^62−78^ Since RCM is largely driven by the gain in entropy when a given diene substrate is converted into a cyclic olefin plus ethylene (which desolvates upon evaporation), the reaction does not allow large enthalpic barriers to be overcome. 11-Membered rings, however, largely draw their chemical and physical attributes from transannular as well as angle strain; in the transition state, these factors represent formidable kinetic handicaps for ring closure.^79^

In addition to this fundamental aspect, serious chemoselectivity issues needed to be considered. All modern alkyne metathesis catalysts leave alkenes untouched,^23−25^ whereas the standard olefin metathesis catalysts are largely indiscriminative: in fact, productive enyne metathesis is only possible because metal carbenes react with both types of π-systems similarly well.^59,80^ In the projected case, any ene/yne crossover would be detrimental. However, we conjectured that conformational preorganization by the rigid tricyclic backbone of the substrates in question might mitigate the risk and make the successive use of RCAM and RCM possible.^81^

**Scheme 5:**
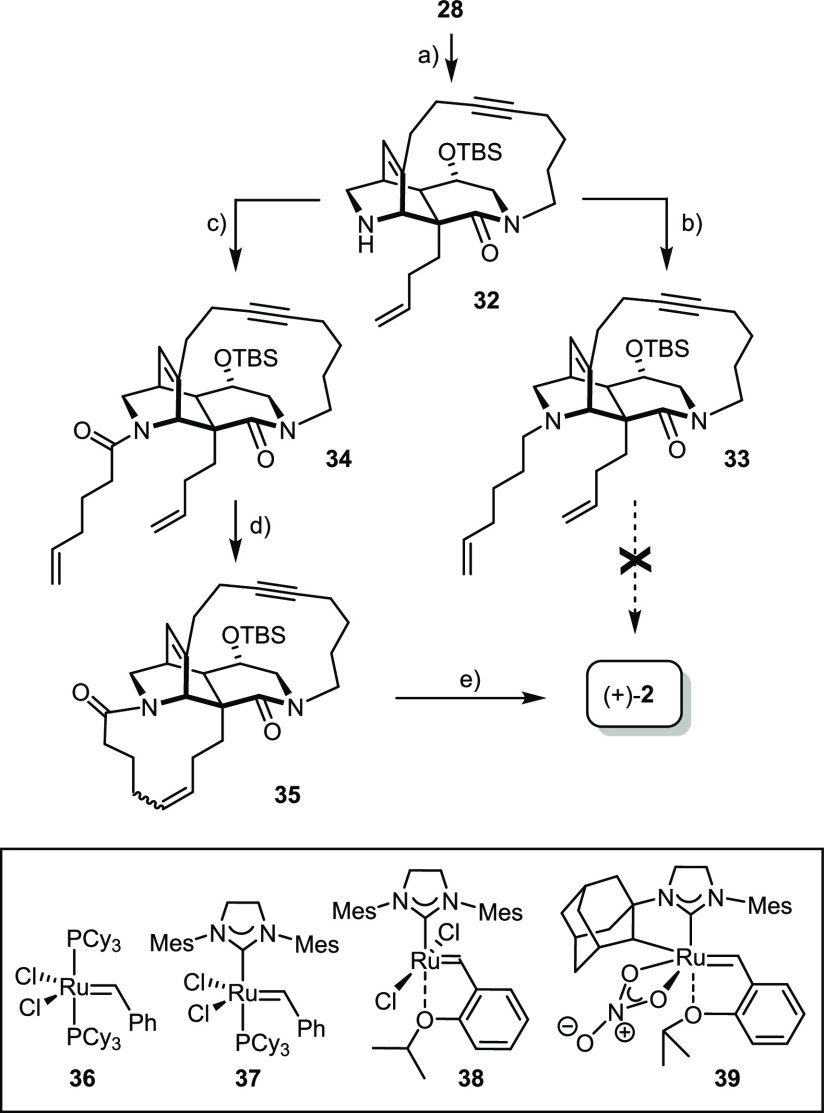
Reagents and conditions: (a) l-Selectride, THF, 40 °C, 91%; (b) hex-5-enal, NaBH(OAc)_3_, CH_2_Cl_2_, 96%; (c) hex-5-enoyl chloride, Et_3_N, CH_2_Cl_2_, 0 °C → RT, 71%; (d) **36** (50 mol %), toluene, 100 °C, 97% (*E/Z* = 60:40); (e) see ref (1).

To test this enticing scenario, **28** was elaborated by selective cleavage of the methyl carbamate group with l-selectride in THF (Scheme 5).^82^ The resulting free amine **32** was converted into amide **34** as well as into *tert*-amine **33**. All attempts at subjecting the latter to ring closure met with failure; the outcome was independent of whether the free base was first protonated with CSA or not^83−89^ and of whether Grubbs-type ruthenium carbenes of the first or second generation were used. When seen against this backdrop, the success of the RCAM reactions in the presence of two different *tert*-amines and a quinoline, as pursued en route to nominal njaoamine I (**4**), will be fully appreciated (see below).

Amide **34** was more compliant. Interestingly, however, only the classical first-generation Grubbs carbene **36**(90) allowed the ring to be closed under high dilution conditions in toluene at elevated temperatures. Since the rate of decomposition of this complex under such forcing conditions is on the same order as productive RCM,^91^ the catalyst had to be slowly added to the mixture; this provision notwithstanding, a very high loading (50 mol %) was necessary to reach full conversion. The reaction furnished **35** as an isomer mixture, in which the undesired *E*-alkene slightly prevailed (*E*/*Z* ≈ 60:40). All attempts at improving this outcome by recourse to the more reactive second-generation catalysts (**37**, **38**; decomposition)^92−94^ or the (*Z*)-selective cyclometalated variant **39** (unreactive)^95^ were to no avail.

The isolation of the required (*Z*)-**35** was only possible by HPLC. This compound intercepts our previous route to ingenamine/xestocyclamine A (except that the original foray had been carried out in the enantiomeric series).^1^ The way from **35** to (+)-**2** commences with a semihydrogenation of the triple bond over nickel boride,^96,97^ followed by reduction of the two remaining amide groups with excess AlH_3_ generated in situ; under these conditions, the silyl ether is concomitantly cleaved. The constitutional and stereochemical integrity of ingenamine thus formed has previously been proven by X-ray diffraction.^1^

In chemical terms, this “second generation” synthesis of ingenamine based on RCAM/RCM is shorter than the original route^1^ relying on macrolactamization/RCAM (16 versus 19 steps, longest linear sequence; see the SI). With ∼2%, however, the overall yields of the two approaches are virtually identical because the advantage of the shorter sequence is outweighed by the lack of stereocontrol in the RCM reaction.

### Total Synthesis of (+)-Keramaphidin B

As mentioned in the Introduction, keramaphidin B (**1**) is the first distinct product on the Baldwin–Whitehead pathway;^5,10,11^ it is thought to derive from the initial Diels–Alder product **B** by reduction of the imine functionality (Scheme 1); interestingly, **1** was isolated in scalemic form from an Okinawan marine sponge of the genus *Amphimedon* sp. only after this intriguing biosynthetic prediction had been made.^4,7,9,98^

(+)-**1** and (−)-**1** differ from ingenamine ((+)-**2**) and xestocyclamine A ((−)-**2**), respectively, in that the −OH group at C-9 is missing. In the context of our synthesis, however, this particular substituent plays a quintessential role: placed in the initial Michael acceptor in −OTBS protected form, it enforces the *trans*-attack of the incoming Michael donor as necessary for proper closure of the caged diaza-tricyclic scaffold (Scheme 2). In this way, the stereochemical information encoded in the C9–OH is relayed to all other chiral centers of the core. Only after it has exerted this critically important function, the directing −OR group can be removed in an attempt to gain access to keramaphidin B.

**Scheme 6:**
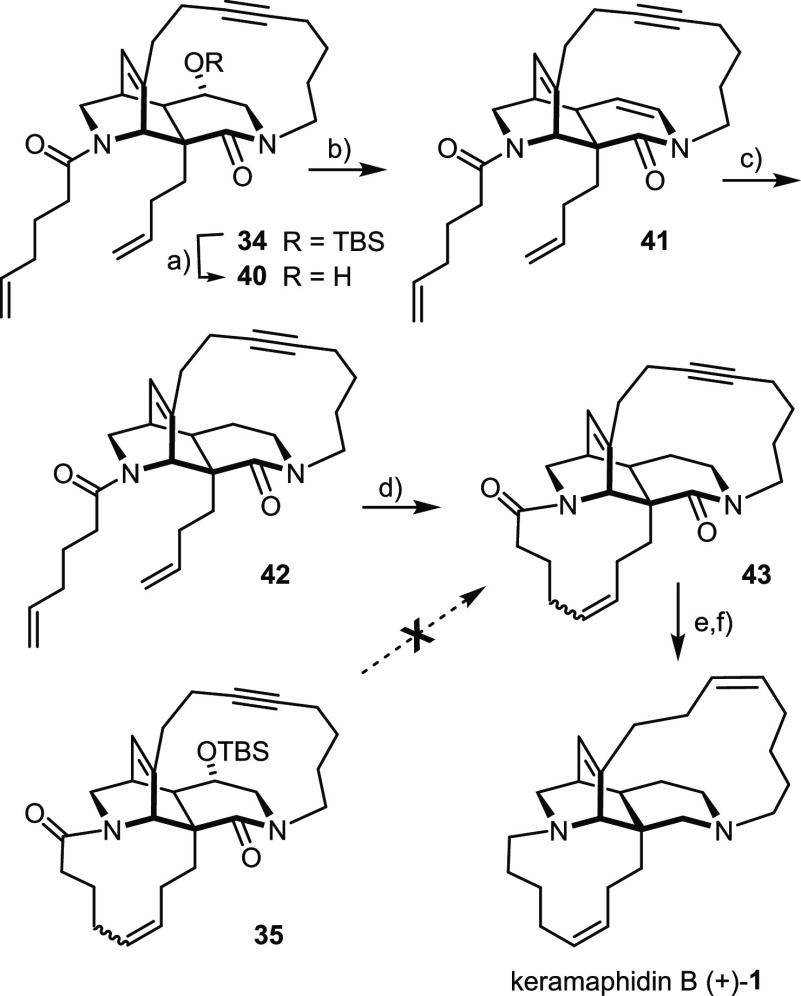
Reagents and conditions: (a) TBAF, THF, 0 °C, quant.; (b) Martin’s sulfurane, toluene, 100 °C, 97%; (c) NaBH_3_CN, TFA, CH_2_Cl_2_, 0 °C → RT, 73%; (d) **36** (50 mol %), 1,2-dichloroethane, 83% (*E*/*Z* = 1:1); (e) Ni(OAc)_2_·4H_2_O, NaBH_4_, ethylenediamine, H_2_ (1 bar), EtOH, 37% (over two steps, pure isomer); (f) Dibal-H, Et_2_O/hexane, 38%.

We had originally planned to perform the deoxygenation at the very end of the synthesis, after both enveloping macrocyclic rings have been set. Although no exhaustive screening exercise was carried out, attempted elimination of the −OH group of **35** under various conditions was not encouraging (Scheme 6). The effort was discontinued when we learned that Martin’s sulfurane^99^ effected the analogous reaction in almost quantitative yield at the stage of **40**, prior to the RCM reaction. Enamide **41** thus formed was then swiftly reduced on treatment with NaBH_3_CN and trifluoroacetic acid.

**Figure 2:**
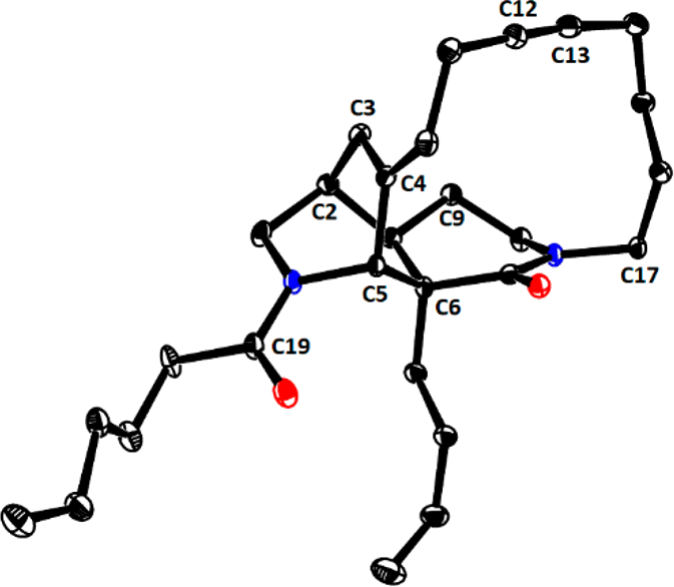
Structure of compound **42** in the solid state.

Figure 2 shows the structure of the resulting amide **42** in the solid state. Of particular interest is the conformation adopted by the tangling 5-hexenamide group that has to participate in the projected RCM reaction with the butenyl group at the C6-bridgehead position: both substituents point “downwards”, away from the triple bond (C12–C13). Although the static picture of the X-ray structure must not be overinterpreted, it might explain why the closure of the 11-membered ring worked without any competing ene/yne crossover. In the event, treatment of **42** with Grubbs catalyst **36** (50 mol %)^90^ in 1,2-dichloroethane at reflux temperature was necessary to enforce the reaction; under these conditions, product **43** was obtained as a 1:1 mixture of the olefin isomers in high yield, which were separable by conventional flash chromatography after the subsequent *Z*-selective semihydrogenation of the triple bond with the aid of nickel-boride.^96,100^ Reduction of both amide groups then completed the total synthesis of (+)-**1**. The analytical and spectral data of the synthetic samples matched those of authentic keramaphidin B^7,9^ very well and hence leave no doubt about structural integrity and identity (for details, see the SI).

### Total Synthesis of Nominal Njaoamine I: Background and Strategic Considerations

**Figure 3:**
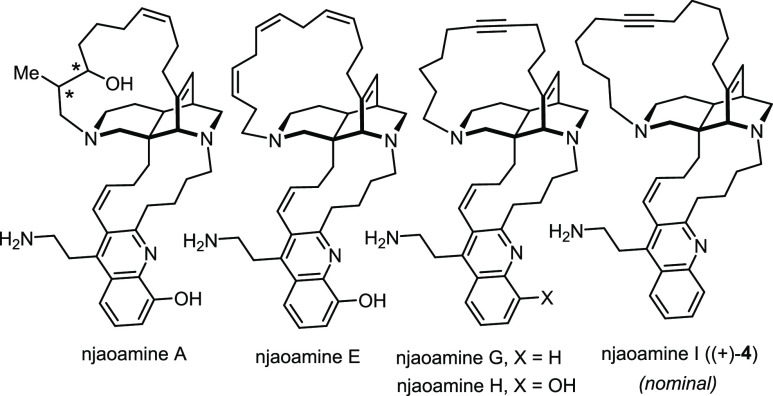
Representative members of the njaoamine family (* stereocenters of unknown configuration).

The polycyclic alkaloids of the njaoamine family were isolated from marine sponges of the genera *Reniera* and *Neopetrosia* sp. collected off the Tanzania coast line.^47−49^ They differ from each other mainly in the size and degree of unsaturation of one of the macrocyclic rings as well as in the oxidation pattern of the quinoline nucleus (Figure 3). Although no comprehensive biological profiling of these compounds was reported, they exhibit moderate to high cytotoxicity against three human cancer cell lines as well as potent activity in a brine shrimp assay.^47−49^ The structural relationship with keramaphidin B/ingenamine is obvious; in terms of biosynthesis, it was proposed that a hetero-Diels–Alder reaction between an ingenamine-type precursor and an oxidized tryptophane derivative entails annulation of the quinoline.^47^ As one might infer from the isolation reports, the absolute configuration of these enticing natural products was tacitly assumed to be that of (+)-keramaphidin B/ingenamine, even though a rigorous proof was missing.

An extension of our program to compounds of this level of complexity, which have never been targeted in the past, was tempting. For the excellent record of RCAM in the current campaign, (nominal) njaoamine I ((+)-**4**) incorporating an intact triple bond in one of the peripheral rings was chosen as our prime target. The answer to the question as to how the second unsaturated macrocycle annulated to the distinguishing quinoline moiety should be forged was less compelling. In view of the failed attempts to effect RCM of an amine or the derived ammonium salt en route to keramaphidin B (see above), this method was unlikely to work in the present context for the additional basic sites in **4** and was therefore ruled out from the very beginning.

Cross-coupling, as pursued in the original synthesis of nominal xestocyclamine A,^1^ should be viable; yet, we conjectured that a second RCAM reaction might actually be a better option. Neither tertiary alkylamines nor substituted pyridine derivatives seem to interfere with the activity of the latest generation of catalysts such as **31**, despite the high-valent Mo(+6) center in the operative alkylidyne unit.^33,56,101,102^ Therefore it was tempting to make use of RCAM twice en route to **4**, even though—or because—such a maneuver was without precedent at the outset of this project.

### Quinoline Building Block

**Scheme 7:**
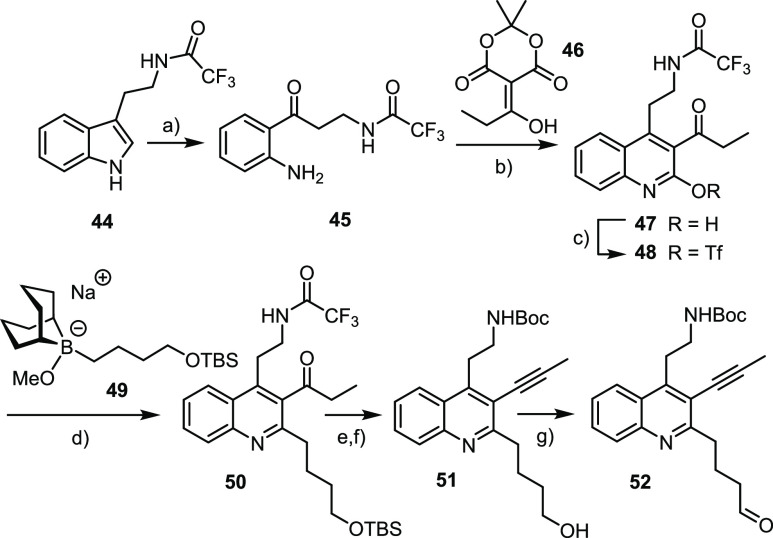
Reagents and conditions: (a) (i) NaIO_4_, MeOH/H_2_O; (ii) aq. HCl, MeOH, 81%; (b) toluene, reflux, then SiO_2_, 94%; (c) Tf_2_O, pyridine, 0 °C → RT, 88%; (d) **49**, Pd(PPh_3_)_4_ (5 mol %), THF, reflux, 73%; (e) (i) PhNTf_2_, KHMDS (excess), THF, −78 °C; (ii) Boc_2_O, DMAP, MeCN; (f) TBAF, THF, 0 °C → RT, 72% (over three steps); (g) pyridine·SO_3_, DMSO, Et_3_N, 0 °C → RT, 77%.

Tryptamine was *N*-trifluoroacetylated before the heterocyclic ring was oxidatively cleaved with NaIO_4_ and the resulting formamide hydrolyzed off with aqueous HCl (Scheme 7). A Dieckmann-type condensation of **45** thus formed with **46** furnished hydroxyquinoline **47** on scale.^103^ The derived triflate **48** was cross-coupled with borate **49** formed by hydroboration of TBS-protected 3-butene-1-ol with 9-H-9-BBN followed by addition of 1 equiv of NaOMe; no further base is necessary under these conditions for the alkyl-Suzuki reaction to proceed.^27,104^ The ketone group of **50** was then transformed into the required triple bond via the corresponding enol triflate, which succumbed to spontaneous elimination when excess KHMDS was present in the mixture.^105^ Treatment of the resulting product with Boc_2_O followed by a workup with NH_4_Cl swapped the protecting group at the amine terminus. Cleavage of the silyl ether followed by oxidation gave aldehyde **52** to be incorporated into the RCAM precursor via reductive amination.

### First Foray

**Scheme 8:**
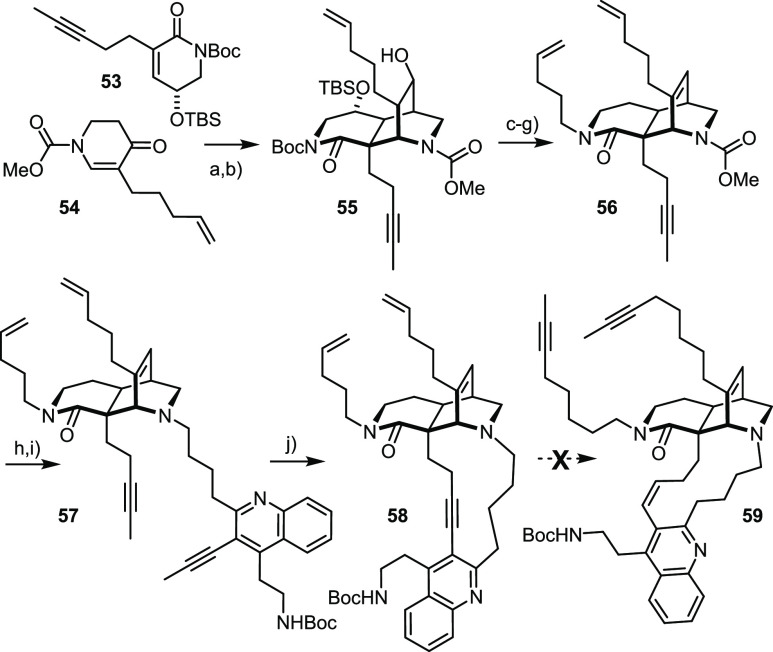
Failed First Foray Reagents and conditions: (a) (i) Bn_2_NLi, DMPU, – 50 °C → 0 °C; (ii) Boc_2_O, DMPU; (b) NaBH_4_, MeOH, 0 °C, 42% (over two steps); (c) MsCl, Et_3_N, DMAP, CH_2_Cl_2_, 85%; (d) 2,6-lutidine, 170 °C, 81%; (e) (i) NaH, 1-iodo-4-pentene, DMF; (ii) TBAF, 98%; (f) (i) ClCH_2_SO_2_Cl, Et_3_N, DMAP, CH_2_Cl_2_; (ii) DBU, 77% (over two steps); (g) NaBH_3_CN, TFA, CH_2_Cl_2_, 0 °C → RT, 60%; (h) l-Selectride, THF, 40 °C; (i) **52**, NaBH(OAc)_3_, CH_2_Cl_2_, 73% (over two steps); (j) **29** (30 mol %), **30** (30 mol %), MS 5 Å, toluene, reflux, 77%.

The improved syntheses of the required building blocks and the ability to forge the nitrogenous core by a true Michael/Michael cascade proved enabling at this stage of the project. Simple adaptation opened access to **53** and **54** and the derived double-Michael adduct **55** comprising a pentynyl group branching off the bridgehead (Scheme 8; for details, see the SI). Note that the stereochemistry matches that of natural xestocyclamine A rather than ingenamine; this choice reflected nothing but the material supply at this point of the project. Elaboration of **55** into **56** echoed the route outlined above without need for major adaptation.^106^ Cleavage of the methyl carbamate followed by reductive amination^107^ with aldehyde **52** furnished **57** in readiness for RCAM. Gratifyingly, this transformation proceeded smoothly, despite the presence of a *tert*-amine and a quinoline functionality.

We had planned to elaborate cycloalkyne **58** in a single operation into diyne **59**, as required for the second macrocyclization, by exhaustive hydroboration, selective proto-deborylation of the alkenylborane originating from the triple bond,^108^ and cross-coupling of the remaining alkylborane functionalities derived from the terminal alkenes with 1-halo-1-propyne (or a synthetic equivalent thereof).^109^ Very much to our dismay, this plan could not be reduced to practice, despite considerable experimentation. Various alternative and more conventional stepwise approaches also failed. Although the complexity of the resulting mixtures prevented full analyses, the reluctance of the triple bond of **58** to undergo hydrometalation (or semihydrogenation) and the unexpected resilience of the alkenylmetal species, once formed, to protodemetalation were identified as major obstacles.

### Tactical Change and Completion of the Total Synthesis

As the projected late-stage introduction of the alkynes needed for the second RCAM reaction was unsuccessful, we had no choice but to carry these handles through the synthesis in masked format. Surrogates of or protecting groups for triple bonds are not commonplace; in the present case, they have to withstand (strongly) basic, acidic, oxidative, and various reductive conditions and must not be cleaved by fluoride either. Actually, none of the established alkyne surrogates seemed to meet these boundary conditions;^110^ after careful consideration of the possible pitfalls, we finally opted for *vic*-dibromoalkenes,^111,112^ despite the danger that the halide atoms could jeopardize the success of the palladium-catalyzed Saegusa-type oxidations and/or the necessary alkyne semireduction over a (noble) metal catalyst.

**Scheme 9:**
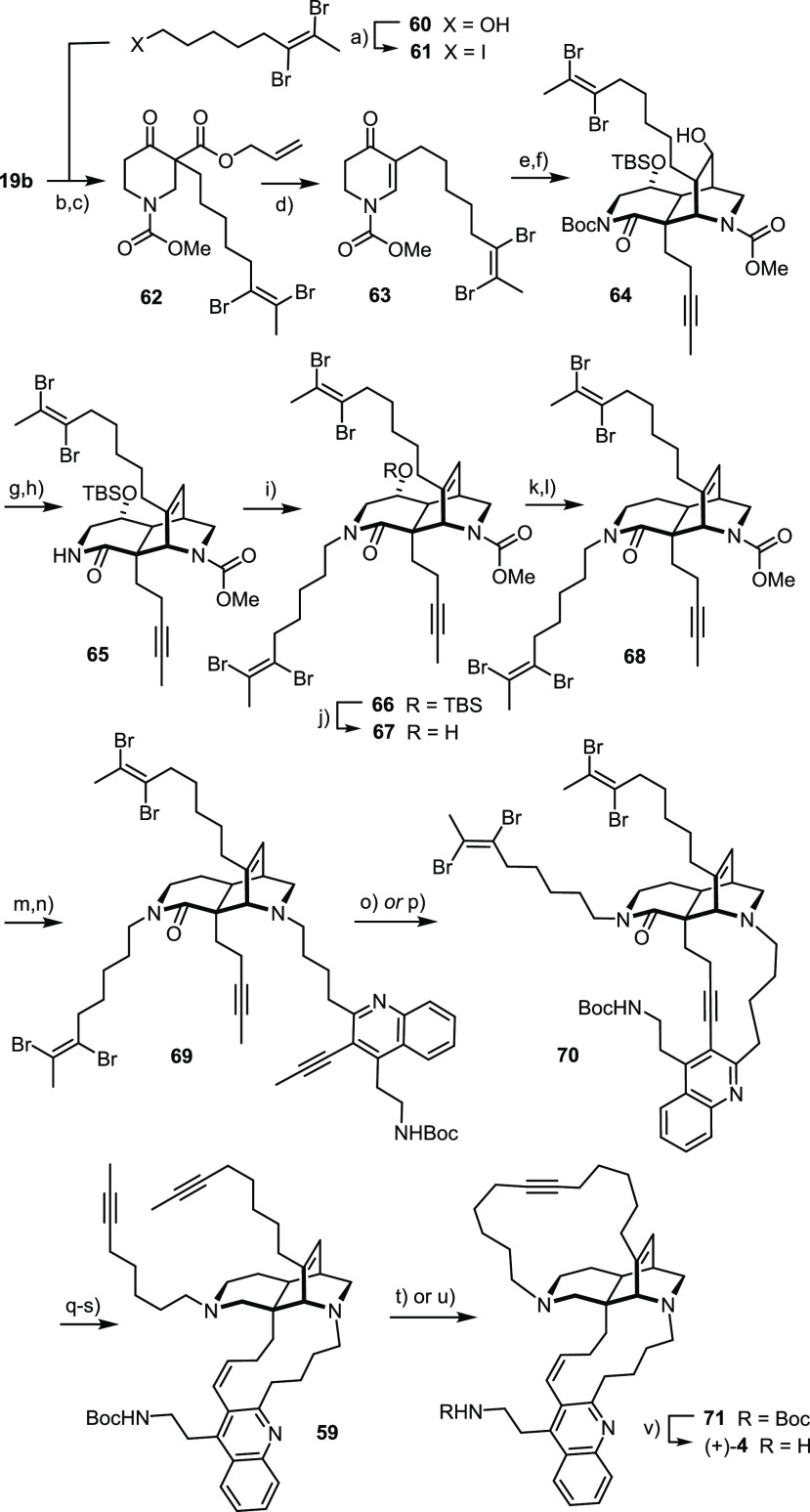
Reagents and conditions: (a) I_2_, PPh_3_, imidazole, CH_2_Cl_2_, 0 °C, 99%; (b) **61**, Cs_2_CO_3_, DMF, 67%; (c) ClCOOMe, toluene, 100 °C, 97%; (d) Pd_2_(dba)_3_ (5 mol %), MeCN, reflux, 92%; (e) (i) **53**, *t*BuOLi, THF, −50 °C → RT; (ii) Boc_2_O, DMAP; (f) NaBH_4_, MeOH, 0 °C, 55% (over two steps); (g) MsCl, Et_3_N, DMAP, CH_2_Cl_2_, 0 °C → RT, 94%; (h) (i) 2,6-lutidine, 170 °C; (ii) TBSOTf, CH_2_Cl_2_, 78%; (i) **61**, NaH, DMF/THF, 0 °C → RT; (j) TBAF, THF, 92% (over two steps); (k) Martin’s sulfurane, toluene, 100 °C, quant.; (l) NaBH_3_CN, TFA, CH_2_Cl_2_, 0 °C → RT, 66%; (m) TMSI, CH_2_Cl_2_; (n) **52**, NaBH(OAc)_3_, CH_2_Cl_2_, 67% (over two steps); (o) **29** (30 mol %), **30** (30 mol %), MS 5 Å, toluene, reflux, 77%; (p) **31** (30 mol %), MS 5 Å, toluene, reflux, 77%; (q) H_2_ (1 bar), Pd/CaCO_3_, THF, 52%; (r) Dibal-H, Et_2_O, 0 °C → RT; (s) Zn, THF, HOAc, 44% (over two steps); (t) **29** (30 mol %), **30** (30 mol %), MS 5 Å, toluene, reflux, 73%; (u) **31** (30 mol %), MS 5 Å, toluene, reflux, 98%; (v) HCl in 1,4-dioxane, EtOAc, quant.

The first of these concerns was quickly found to be unjustified (Scheme 9). By following the now established route, **19b** was alkylated with iodide **61** comprising such a *vic*-dibromoalkene moiety (readily available from 6-octyne-1-ol). Gratifyingly, this group did not interfere at all in the subsequent Saegusa oxidation: this favorable result is tentatively attributed to the fact that the reaction works with Pd_2_(dba)_3_ as the catalyst without need for an external ligand:^55^ while this “bare” palladium species is capable of activating the allyl ester, it is not sufficiently electron-rich to engage in competitive oxidative insertion into the alkenyl bromide bonds, which would be detrimental in the present case.

The base-induced Michael/Michael cascade and subsequent elaboration of the core fragment **64** proceeded uneventfully; the only minor concern was the reduction of enamide formed upon elimination of the 9-OH group of **67** with Martin’s sulfurane:^99^ as the reaction requires treatment with excess NaBH_3_CN/TFA, it proved mandatory to quench the reaction as soon as full conversion of the substrate was reached; under this proviso, the results were well reproducible (66%, 660 mg scale, single largest batch).

An unexpected problem was encountered in the seemingly trivial cleavage of the methyl carbamate. l-Selectride had served this purpose well before (see above) but was found inadequate for **68**, as it led to extensive reduction of the *vic*-dibromoalkenes present in this substrate (see below). The problem was ultimately circumvented by recourse to TMSI.^113^ Subsequent attachment of the quinoline-containing side chain via reductive amination furnished diyne **69** as necessary for the first macrocyclization event.

This exigent transformation worked again very nicely, independent of whether the in situ catalyst mixture (**29**/**30**)^56^ or the structurally defined canopy catalyst **31**(33−35) was used. The need for fairly high loadings is again tentatively ascribed to the forcing conditions necessary to override the incipient ring strain of the polycyclic product **70**, which certainly also accelerate catalyst decomposition (for reaction optimization, see the SI); this notion is supported by recent results from our laboratory.^114^ As expected, the *vic*-dibromoalkenes neither compromise the activity of the catalyst nor become damaged. This result adds another important entry to the list of functional groups that are compatible with these versatile molybdenum alkylidynes.

The difficulties encountered in the first foray to subject cycloalkyne **58** to any kind of stereoselective semireduction foreshadowed the challenge to engage the closely related but even more highly functionalized analogue **70**; in fact, hydrometalation was not viable. With regard to more classical hydrogenations, it is pointed out that this substrate incorporates a quinoline which likely acts as an internal catalyst poison. Though tentative, this may explain why many standard reagents and catalysts either failed to react altogether or resulted in intractable mixtures.^100^ After many failed attempts, it was found that *unpoisoned* Pd/CaCO_3_ was appropriate, provided the reaction was carried out in THF as the solvent and the “catalyst” was used in excess (2 equiv). Under these conditions, the required (*Z*)-alkene was isolated as the only isomer in 52% yield with the lateral four alkenyl bromide moieties intact.

The subsequent selective reduction of the amide turned out to be no less demanding: it required considerable experimentation to find that Dibal-H in Et_2_O shows the proper selectivity profile. The choice of solvent is critical, and the conversion must be carefully monitored: once the substrate is consumed, the reaction has to be immediately quenched to prevent reduction of the C–Br bonds.

Compared with these two rather taxing chemoselective reduction steps, the completion of the total synthesis was straightforward. Thus, treatment with zinc dust in acidic medium swiftly unmasked the triple bonds.^111,115^ In line with our expectations, the subsequent RCAM reaction of **59** proceeded cleanly. Once again, the in situ catalyst mixture **29**/**30**^56^ and the well-defined canopy catalyst **31**(33−35) were both operative, with the latter affording the desired cycloalkyne **71** in essentially quantitative yield. This result is deemed very rewarding, since the effectiveness of the high-valent molybdenum alkylidyne endowed with the silanolate ligand sphere is not compromised by the presence of two different tertiary amines, a quinoline, and a Lewis basic carbamate group in the substrate. To properly assess this result, one has to recall that RCM of **33** comprising a single *tert*-amine could not be enforced at all with the aid of Grubbs-type ruthenium carbene complexes, even after protonation. Therefore, this example—together with a number of other advanced applications^39−44,57,116−126^—may help correct the misperception that high-valent early transition metal catalysts provide (too) few opportunities when working with densely decorated compounds; in any case, it is fair to claim that the newly developed molybdenum alkylidynes with (tripodal) silanolate ligands are distinguished by a remarkable and enabling functional group tolerance.

Final cleavage of the −NBoc group with HCl in 1,4-dioxane/EtOAc^127^ completed the total synthesis of what had been proposed to be njaoamine I ((+)-**4**). Yet, the analytical and spectral properties of the synthetic samples showed small deviations from the tabulated NMR data.^49^ A comparison with an authentic sample confirmed that the differences are beyond the error bar even though they are extremely subtle and the compounds were not distinguishable by HPLC either (see the SI). The fact, however, that the mismatch was clustered in the macrocycle comprising the triple bond suggested that the issue might have to do with the exact location of the alkyne within this ring.

### Structure Revision of Njaoamine I

With the tiny amount (<1 mg) of the isolated natural product made available to us by the generosity of the isolation team, we were able to confirm this supposition.

To this end, a complete and unambiguous reassignment of the entire 12-carbon chain from C33 to C44 was mandatory; this task proved challenging because the dilute sample in [D_5_]-pyridine rendered heteronuclear long-range coupling experiments (in particular HMBC) impractical. Moreover, the limited resolution impeded assignment, especially between 1.1 and 1.7 ppm where 20 methylene protons (14 of them from the chain in question) and one methine proton resonate; likewise, there are two very similar methylene ^13^C signals at 27.7 and 27.8 ppm, the correlations of which can only be separated by very high-resolution multidimensional experiments.

**Figure 4:**
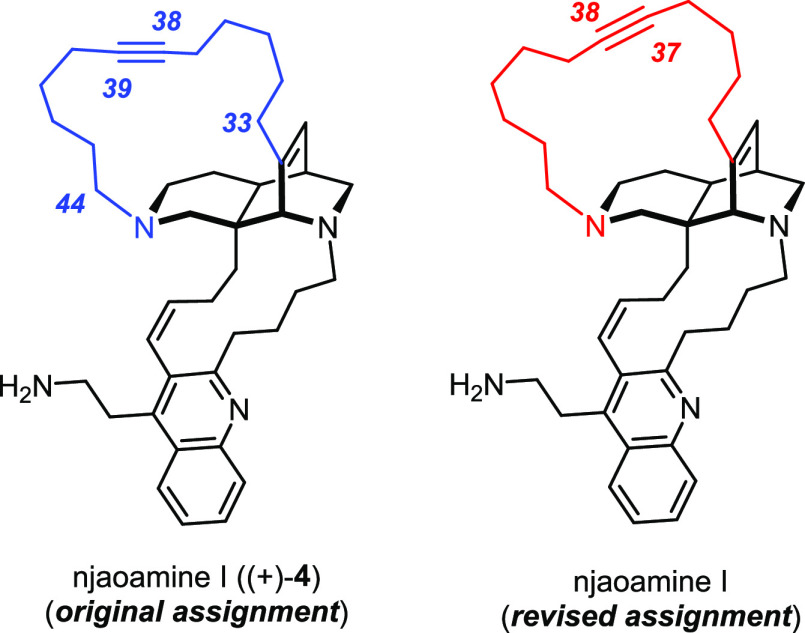
Originally assigned and revised structure of njaoamine I.

The challenges were ultimately met by a series of CLIP-COSY,^128^ high-resolution HSQC, and high-resolution HSQC-TOCSY experiments (see the SI); the latter proved particularly informative: they provided compelling evidence that the triple bond is located at C37≡C38 (rather than C38≡C39, as had originally been proposed by the isolation team).^49^ Njaoamine I is hence a regioisomer of the originally assigned structure in which the triple bond is shifted by one C atom (Figure 4).

A brief comment on the absolute configuration is also warranted. In contrast to what the drawing in the isolation paper might insinuate,^49^ it seems probable that the absolute configuration of the natural product is *opposite* to that of natural ingenamine: though isomeric to each other, synthetic nominal njaoamine I ((+)-**4**) and authentic njaoamine I are both dextrorotatory. Since (+)-**4** derives from *ent*-**14** (via **53**), it is likely that njaoamine I is a sister compound of xestocyclamine A rather than ingenamine (for details, see the SI).

### Coda: Concurrent Formation of Both Macrocycles

The poor results obtained in the biomimetic studies directed toward keramaphidin B mentioned in the Introduction, in which concurrent formation of both macrocycles had been attempted,^10,11^ had prompted us to pursue a stepwise approach toward nominal njaomamine I in the first place as outlined above. Yet, the ability to effect an RCAM/RCM sequence en route to **35** without competing ene/yne crossover implies that the orientation of the tangling side chains on a rigid tricyclic core is favorable. Therefore, *simultaneous closure of both enveloping rings* by RCAM of a tetra-yne derivative seemed possible.

**Scheme 10:**
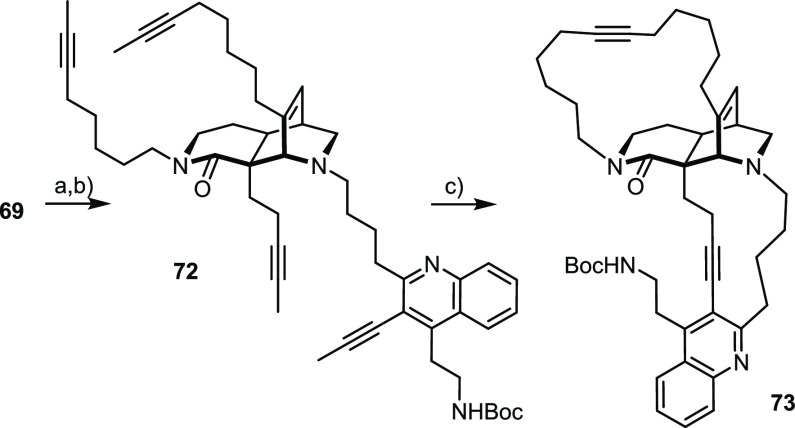
Two Concurrent RCAM Reactions Reagents and conditions: (a) l-Selectride, THF, 40 °C; (b) **52**, NaBH(OAc)_3_, CH_2_Cl_2_, HOAc, 67% (over two steps); (c) **29** (60 mol %), **30** (60 mol %), MS 5 Å, toluene, reflux, 35% (**73** + 17% (isomer), see text).

To test this tantalizing concept, **69** was treated with l-selectride as a nonoptimized shortcut for the preparation of the required tetra-yne derivative **72** (Scheme 10). Both catalyst systems used herein did an excellent job in converting this compound into **73** and small amounts of a second as yet unidentified isomer formed by improper concatenation of the ends. The (almost) copolarity of the silanolate ligand hydrolyzed off the catalysts during workup turned out to be a technical issue; for these separation problems, the isolated yield of analytically pure **73** was only 35%, even though the reaction itself is clean. No further attempt was made to optimize the result (ligand variation, catalyst loading, etc.) because the subsequent site-selective reduction of the very hindered C31–C32 alkyne without touching the more accessible C36–C37 triple bond has so far not worked out. Yet, the successful double ring closure opens new perspectives for the use of RCAM in target-oriented synthesis at large; opportunities along these lines are currently under investigation in this laboratory.

## Conclusions

The triumph of olefin metathesis is not only rooted in the abundance of olefins but also in the availability of catalysts that activate double bonds while leaving almost all other functional groups intact.^129−131^ This virtue allows retrosynthetic disconnections to be realized that are largely orthogonal to the conventional logic.^59−61,132,133^ Although (internal) alkynes in general are chemically more “expensive” than olefins and their use therefore only justified when specific and purposeful advantage is taken of their rich reactivity,^23−25^ it is of note that the latest generation of alkyne metathesis catalysts work in the presence of certain functional groups that even some of the most accomplished olefin metathesis catalysts cannot handle. The conquests of the polycyclic alkaloids keramaphidin B, ingenamine, xestocyclamine A, and, not least, nominal njaoamine I bear witness of the notion that alkyne metathesis has reached strategy level. This aspect notwithstanding, further improvements of the catalysts are necessary and perhaps likely in the near future.^134,135^

At the same time, the present study illustrates that the implementation of alkyne metathesis can be limited by the lack of appropriate triple bond surrogates and/or the inability to address and manipulate a given alkyne in the presence of other functionality: the difficulties in subjecting compounds **58** and **70** to seemingly trivial chemoselective hydrometalation or semihydrogenation illustrate this aspect. In the future, the significance of alkyne metathesis will also be defined by the upstream and/or downstream alkyne chemistry. Therefore, our laboratory is committed to explore opportunities along these lines^136^ in parallel to our ongoing work on fundamental and applied aspects of alkyne metathesis proper.^35,36,114,137,138^

## Supporting Information Available

Experimental section including supporting crystallographic information, experimental procedures, and characterization data; copies of NMR spectra of new compounds (PDF)

## Accession Codes

CCDC 2081189–2081190 contain the supplementary crystallographic data for this paper. These data can be obtained free of charge via www.ccdc.cam.ac.uk/data_request/cif, or by emailing data_request@ccdc.cam.ac.uk, or by contacting The Cambridge Crystallographic Data Centre, 12 Union Road, Cambridge CB2 1EZ, UK; fax: +44 1223 336033.
