# Supplementary material for: A Unified Approach to Polycyclic Alkaloids of the Ingenamine Estate: Total Syntheses of Keramaphidin B, Ingenamine, and Nominal Njaoamine I
Source: J Am Chem Soc. 2021 Aug 27;143(35):14402–14. doi: 10.1021/jacs.1c07955 (PMC8431342; doi:10.1021/jacs.1c07955)
Supplement: Supplementary file 1 — ja1c07955_si_001.pdf [file ja1c07955_si_001.pdf]

Supporting Information

A Unified Approach to Polycyclic Alkaloids of the Ingenamine Estate: Total Syntheses of Keramaphidin B, Ingenamine, and Nominal Njaoamine I

Zhanchao Meng,[+] Simon M. Spohr,[+] Sandra Tobegen, Christophe Farès, and Alois Fürstner*

[+] these authors contributed equally

Max-Planck-Institut für Kohlenforschung, 45470 Mülheim/Ruhr, Germany

Email: fuerstner@kofo.mpg.de

Table of Contents

Supporting Crystallographic Information	S2
General	S4
Ingenamine and Keramaphidine	S5
Nominal Njaoamine I. The Quinoline Building Block	S25
The Failed First Foray	S28
Completion of the Total Synthesis	S38
NMR Assignment and Structure Confirmation of Synthetic Nominal Njaomine I ((+)-**4**)	S51
Comparison of Synthetic Nominal Njaomine I ((+)-**4**) with Native Njaoamine I	S65
Structure Revision of Njaoamine I Based on a Re-Assessment of an Authentic Sample	S69
Two Concurrent RCAM Reactions	S78
Copies of Spectra	S80
References	S199

Supporting Crystallographic Information

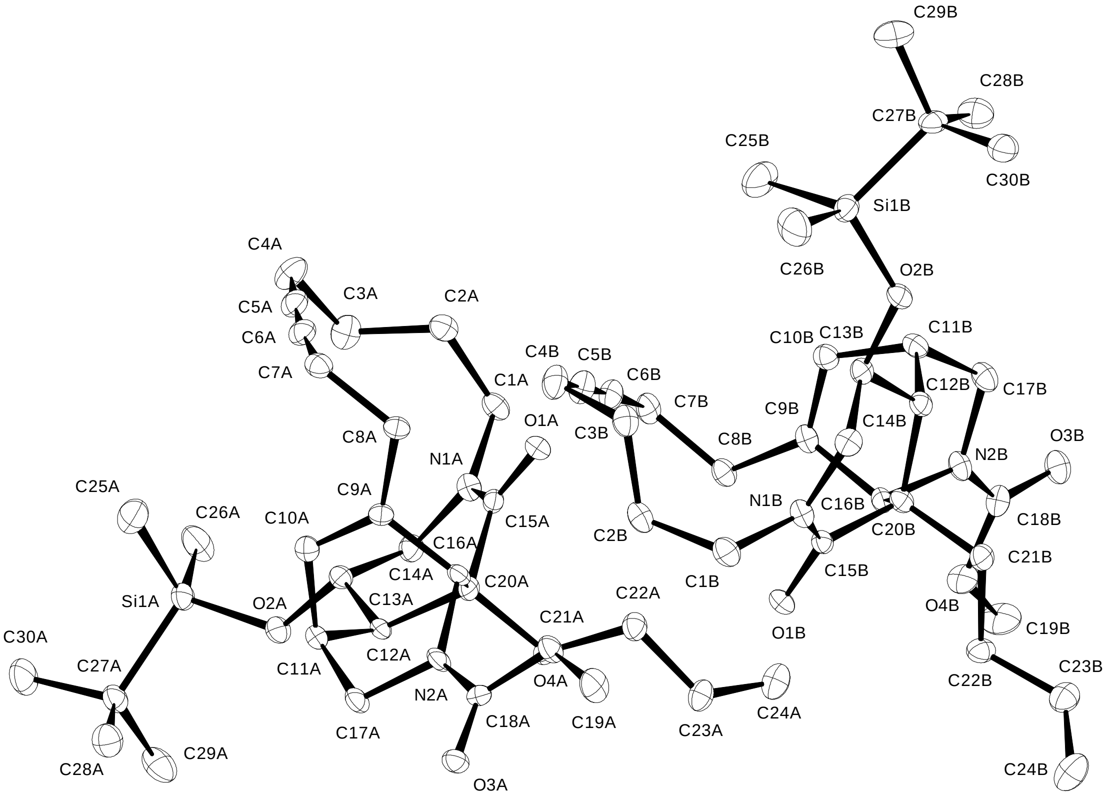

Figure S1. Molecular structure of the two independent molecules of cycloalkyne **28** in the solid state; atomic displacement ellipsoids are shown at the 50% probability level, H-atoms omitted for clarity

X-ray Crystal Structure Analysis of Compound 28: $C_{30}H_{46}N_2O_4Si$, M_r = 526.78 g · mol^{-1}, colorless needle, crystal size 0.140 x 0.034 x 0.025 mm^3, monoclinic, space group $P2_1$ [4], a = 15.6435(7) Å, b = 8.6081(4) Å, c = 23.3896(10) Å, β = 109.531(2)°, V = 2968.4(2) Å^3, T = 100(2) K, Z = 4, D_{calc} = 1.179 g · cm^3, λ = 0.71073 Å, $\mu(Mo\text{-}K_\alpha)$ = 0.115 mm^{-1}, analytical absorption correction ($T_{\min}$ = 0.99, $T_{\max}$ = 1.00), Bruker-AXS Kappa Mach3 APEX-II diffractometer with a Iµs microsource, 1.381 < θ < 32.467°, 106114 measured reflections, 20839 independent reflections, 16939 reflections with $I > 2\sigma(I)$, R_{int} = 0.0706, S = 1.031, 680 parameters, absolute structure parameter = 0.02(3), residual electron density +0.4 (1.12 Å from H3AA) / –0.4 (0.13 Å from Si1A) e · Å^{-3}.

The structure was solved by *SHELXT* and refined by full-matrix least-squares (*SHELXL*) against F^2 to R_1 = 0.049 [$I > 2\sigma(I)$], wR_2 = 0.107. **CCDC-2081190**.

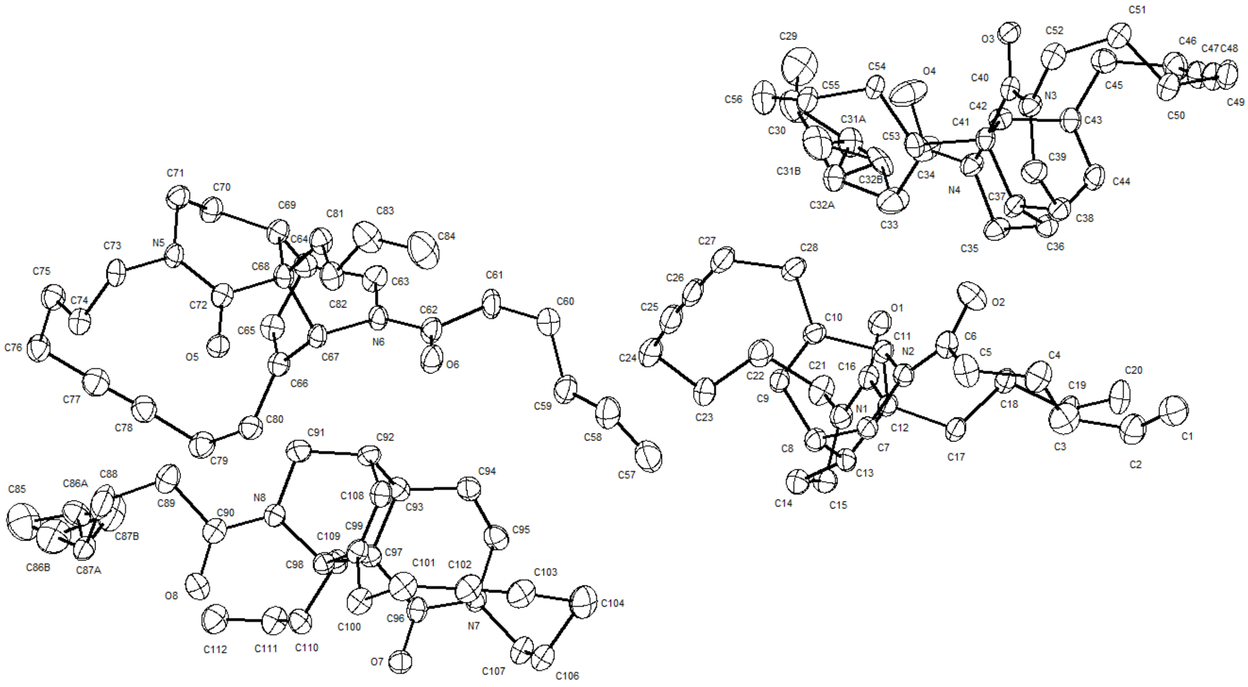

Figure S2. Molecular structure of the four independent molecules of compound **42** in the solid state; atomic displacement ellipsoids are shown at the 50% probability level, H-atoms omitted for clarity

X-ray Crystal Structure Analysis of Compound 42: $C_{28}H_{38}N_2O_2$, M_r = 434.60 g · mol^{-1}, colorless plate, crystal size 0.180 x 0.155 x 0.111 mm^3, triclinic, space group *P1* [2], *a* = 11.7256(5) Å, *b* = 13.3258(6) Å, *c* = 15.6551(7) Å, α = 89.927(2)°, β = 89.955(2)°, γ = 83.357(2)°, *V* = 2429.73(19) Å^3, *T* = 100(2) K, *Z* = 4, D_{calc} = 1.188 g · cm^3, λ = 1.54178 Å, $\mu(Cu\text{-}K_\alpha)$ = 0.576 mm^{-1}, analytical absorption correction ($T_{\min}$ = 0.92, $T_{\max}$ = 1.00), Bruker AXS Enraf-Nonius KappaCCD diffractometer with a FR591 rotating Cu-anode X-ray source, 2.823 < θ < 72.989°, 105618 measured reflections, 18363 independent reflections, 17486 reflections with $I > 2\sigma(I)$, R_{int} = 0.0426, *S* = 1.145, 1190 parameters, absolute structure parameter = –0.09(6), residual electron density +0.2 (0.71 Å from H33B) / –0.2 (0.86 Å from C108) e · Å^{-3}.

The structure was solved by *SHELXT* and refined by full-matrix least-squares (*SHELXL*) against F^2 to R_1 = 0.040 [$I > 2\sigma(I)$], wR_2 = 0.093. **CCDC-2081189**.

General. Unless stated otherwise, all reactions were carried out in flame-dried glassware using anhydrous solvents under argon atmosphere. The solvents were purified by distillation over the following drying agents and were transferred under argon: THF, Et_2O (Mg/anthracene); MeCN, 2,6-lutidine, CH_2Cl_2, DCE (CaH_2); toluene (Na/K alloy); MeOH (Mg, stored over MS 3 Å). DMSO, DMF, NEt_3, pentane and pyridine were dried by an adsorption solvent purification system based on molecular sieves. Thin layer chromatography (TLC): Macherey-Nagel precoated plates (POLYGRAM®SIL/UV254). Detection was achieved under UV-Light (254 nm) and by staining with either acidic *p*-anisaldehyde, cerium ammonium molybdenate or basic $KMnO_4$ solution. Flash chromatography: Merck silica gel 60 (40–63 µm) with predistilled or HPLC grade solvents. NMR: Spectra were recorded on Bruker AV 400, AV 500, AVIII 600 or AVneo 600 spectrometers in the solvents indicated; chemical shifts (δ) are given in ppm relative to TMS, coupling constants (*J*) in Hz. The solvent signals were used as references and the chemical shifts converted to the TMS scale ($CDCl_3$: δ_C = 77.00 ppm; residual $CHCl_3$ in $CDCl_3$: δ_H = 7.26 ppm; CD_3OD: δ_C = 49.00 ppm, residual CD_2HOD in CD_3OD: δ_H = 3.31 ppm; $(CD_3)_2SO$: δ_C = 39.52 ppm, residual CD_2HSOCD_3 in $(CD_3)_2SO$: δ_H = 2.50 ppm); all spectra were recorded at 25 °C. Multiplicities are indicated by the following abbreviations: s: singlet, d: doublet, t: triplet, q: quartet, p: pentet, h: hextet, hept: heptet, m: multiplet, br: broad signal. ^{13}C NMR spectra were recorded in ^{1}H-decoupled manner and the values of the chemical shifts are rounded to one decimal point. Signal assignments were established using HSQC, HMBC, COSY, NOESY and other 2D experiments. IR: Spectra were recorded on an Alpha Platinum ATR instrument (Bruker), wavenumbers ($\tilde{\nu}$) in cm^{-1}. MS (ESI-MS): Finnigan MAT 8200 (70 eV), ESI-MS: ESQ3000 (Bruker), accurate mass determinations: Bruker APEX III FTMS (7 T magnet) or Mat 95 (Finnigan). Optical rotations ($[\alpha]_D$) were measured with an A-Krüss Otronic Model P8000-t polarimeter at a wavelength of 589 nm. Preparative LC was performed with an Agilent 1260 infinity prep system (fraction collector G7159 B + G7166A, diode array detector G7115A); stationary phase and conditions for each compound are specified below.

Molecular sieves (5 Å) were activated at 150°C for 24 h in high vacuum (1×10^{-3} mbar) and stored under argon.

Unless stated otherwise, commercially available compounds (Alfa Aesar, Aldrich, TCI, Strem Chemicals, ChemPUR) were used as received. The following compounds were prepared according to the cited literature: 5-iodopent-2-yne[1], 7-iodohept-2-yne[2] and molybdenum alkylidyne complex **31**.[3]

Table S1. Statistical Analysis

Target	Macrocyclization Tactics	Step Count (LLS)[a]	Total Step Count	Overall Yield
nominal xestocyclamine A[b]	RCAM / alkyl-Suzuki coupling	16	20	1.97%
actual xestocyclamine A (= *ent*-ingenamine A)[b]	RCAM / macrolactamization	19	23	1.94%
ingenamine A	RCAM / RCM	16	20	2.01%
keramaphidine B	RCAM / RCM	19	23	0.93%
nominal njaoamine I	RCAM / RCAM	21	32	1.14%

[a] starting from commercially available materials; [b] see ref. 2; LLS = longest linear sequence; RCAM = ring closing alkyne metathesis; RCM = ring closing olefin metathesis

Ingenamine and Keramaphidine

***tert*-Butyl (*S*)-3-((*tert*-butyldimethylsilyl)oxy)piperidine-1-carboxylate (14).** 4-Dimethylamino-pyridine

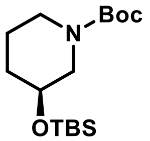

(68.3 mmol, 8.3 g) and triethylamine (124.22 mmol, 17.3 mL) were added to a stirred solution of (*S*)-1-Boc-3-hydroxypiperidine (124.22 mmol, 25.00 g) at room temperature. After 5 min, *tert*-butyldimethylsilylchloride (132.91 mmol, 20.03 g) was added and the resulting mixture stirred for 4 h at room temperature. Next, the mixture was poured into ice-cooled water (100 mL), which was extracted with CH_2Cl_2 (3 x 250 mL). The combined organic phases were washed with brine (50 mL), dried over Na_2SO_4 and concentrated *in vacuo*. The residue was purified by flash chromatography on silica (hexanes/EtOAc, 10:1), providing the title compound as a colorless oil (39.09 g, quant.). $[\alpha]_{D}^{25}$ = +14.7° (c = 1.0, $CHCl_3$)[4]; ^{1}H NMR (400 MHz, $CDCl_3$): δ = 3.91 – 3.73 (m, 1H), 3.68 (dt, *J* = 13.3, 3.7 Hz, 1H), 3.59 (dp, *J* = 8.3, 4.0 Hz, 1H), 2.89 (tt, *J* = 10.0, 3.3 Hz, 1H), 2.80 (t, *J* = 9.7 Hz, 1H), 1.90 – 1.78 (m, 1H), 1.77 – 1.64 (m, 1H), 1.63 – 1.57 (m, 1H), 1.45 (s, 10H), 0.88 (s, 9H), 0.07 (d, *J* = 3.4 Hz, 6H); ^{13}C NMR (101 MHz, $CDCl_3$): δ = 154.8, 79.3, 67.1, 51.1, 43.6, 33.9, 28.4, 25.8, 23.1, 18.1, -4.8; IR (film): $\tilde{\nu} =$ 2930, 2886, 2857, 1697, 1465, 1421, 1391, 1365, 1278, 1254, 1239, 1176, 1154, 1099, 1041, 981, 904, 873, 858, 837, 775 cm^{-1}. HRMS (ESI): *m/z* calcd. for $C_{16}H_{33}NO_3SiNa$ [M+Na$^+$]: 338.21219, found: 338.21235.

***tert*-Butyl (*S*)-5-((*tert*-butyldimethylsilyl)oxy)-2-oxopiperidine-1-carboxylate (15).** Ruthenium(IV) oxide

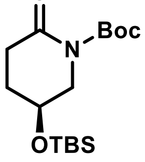

hydrate (7.31 mmol, 974 mg) was added to a solution of piperidine **14** (122.02 mmol, 38.50 g) and $NaIO_4$ (569.83 mmol,121.88 g) in EtOAc/H_2O (1.62 L, 1:3). The resulting mixture was vigorously stirred in a flask open to air at room temperature for 1.5 h. The organic phase was separated and the aqueous layer extracted with EtOAc (3 x 300 mL). The combined

organic extracts were stirred with isopropanol (20 mL) for 3h to decompose any remaining catalyst before they were filtered. The filtrate was washed with brine (100 mL), dried over Na_2SO_4 and concentrated *in vacuo*. The residue was purified by flash chromatography on silica (hexanes/EtOAc, 20:1 to 10:1), furnishing the title compound as a white solid (22.10 g, 55% yield). M.p. = 36.3-37.2 °C; $[\alpha]_D^{25}$ = +8.2° (c = 1.0, $CHCl_3$)[4]; ^{1}H NMR (400 MHz, $CDCl_3$): δ = 4.19 – 4.10 (m, 1H), 3.69 – 3.58 (m, 2H), 2.71 (ddd, *J* = 17.2, 9.1, 6.7 Hz, 1H), 2.42 (dt, *J* = 17.2, 6.2 Hz, 1H), 1.95 (dddd, *J* = 13.2, 9.0, 6.4, 3.9 Hz, 1H), 1.82 (ddtd, *J* = 13.6, 6.8, 5.7, 1.2 Hz, 1H), 1.51 (s, 9H), 0.87 (s, 9H), 0.07 (s, 6H); ^{13}C NMR (101 MHz, $CDCl_3$): δ = 170.9, 152.5, 82.9, 64.4, 52.4, 31.1, 29.0, 28.0, 25.7, 18.0, -4.9; IR (film): $\tilde{\nu}$ = 2954, 2931, 2895, 2857, 1773, 1716, 1472, 1391, 1368, 1346, 1296, 1251, 1151, 1114, 1087, 1061, 1020, 984, 938, 881, 836, 777, 702 cm^{-1}. HRMS (ESI): *m/z* calcd. for $C_{16}H_{31}NO_4SiNa$ [M+Na$^+$]: 352.19146, found: 352.19136.

3-Allyl 1-(*tert*-butyl) (5*S*)-5-((*tert*-butyldimethylsilyl)oxy)-2-oxopiperidine-1,3-dicarboxylate (S1).

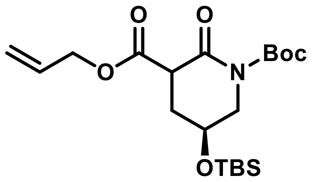

LiHMDS (1 M in THF, 114.82 mmol, 19.21 g) was added dropwise to a solution of oxopiperidine **15** (49.92 mmol, 16.45 g) in anhydrous THF (250 mL) at –78 °C. The mixture was stirred at –78 °C for 1 h, before allyl chloroformate (52.42 mmol, 5.6 mL) was added. The resulting yellow solution was stirred for 25 min at –78 °C before the reaction was quenched with sat. aq. NH_4Cl solution (50 mL) and the mixture warmed to ambient temperature. The aqueous phase was diluted with H_2O (100 mL) and extracted with EtOAc (3 x 300 mL). The combined extracts were washed with brine (100 mL), dried over $MgSO_4$ and concentrated *in vacuo*. The residue was purified by flash chromatography on silica (hexanes/EtOAc, 5:1), furnishing the title compound as a white solid (19.45 g, 94% yield). M.p. = 49.4-50.3 °C. $[\alpha]_D^{25}$ = +15.0° (c = 1.0, $CHCl_3$); ^{1}H NMR (400 MHz, $CDCl_3$, mixture of diastereomers) δ = 5.93 (ddtd, *J* = 17.2, 10.5, 5.7, 2.0 Hz, 1H), 5.35 (dp, *J* = 17.2, 1.5 Hz, 1H), 5.25 (ddt, *J* = 10.5, 2.1, 1.2 Hz, 1H), 4.73 – 4.61 (m, 2H), 4.30 – 4.21 (m, 0.7H, major), 4.13 (tdd, *J* = 6.4, 5.6, 3.7 Hz, 0.3H, minor), 3.84 – 3.78 (m, 0.7H, major), 3.77 – 3.63 (m, 1.7H, major), 3.60 (ddd, *J* = 13.2, 3.9, 0.9 Hz, 0.3H, minor), 3.46 (dd, *J* = 10.0, 7.3 Hz, 0.3H, minor), 2.37 – 2.19 (m, 1.3H), 2.09 (dddd, *J* = 13.6, 6.4, 4.6, 1.6 Hz, 0.7H, major), 1.51 (d, *J* = 1.7 Hz, 9H), 0.87 (d, *J* = 1.6 Hz, 9H), 0.12 – 0.04 (m, 6H); ^{13}C NMR (101 MHz, $CDCl_3$, mixture of diastereomers): δ = 169.6, 168.8, 166.9, 166.9, 152.5, 152.2, 131.6, 131.6, 118.7, 118.6, 83.5, 83.4, 66.1, 64.3, 63.2, 52.2, 51.3, 49.5, 48.0, 33.4, 32.8, 27.9, 25.6, 17.9, -4.8, -4.9, -5.0, -5.0; IR (film): $\tilde{\nu}$ = 2955, 2932, 2896, 2857, 1776, 1746, 1722, 1472, 1391, 1369, 1296, 1255, 1147, 1103, 1030, 1005, 970, 927, 838, 810, 778 cm^{-1}. HRMS (ESI): *m/z* calcd. for $C_{20}H_{35}NO_6SiNa$ [M+Na$^+$]: 436.21259, found: 436.21242.

3-Allyl 1-(*tert*-butyl) (5*S*)-3-(but-3-en-1-yl)-5-((*tert*-butyldimethylsilyl)oxy)-2-oxopiperidine-1,3-dicarboxylate (16).

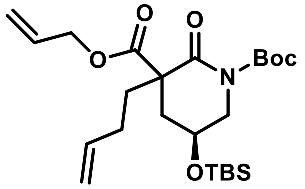

4-Bromobut-1-ene (70.62 mmol, 7.2 mL) and caesium carbonate (75.32 mmol, 24.54 g) were added to a solution of compound **S1** (47.08 mmol, 19.47 g) in anhydrous DMF (47 mL) at room temperature. The mixture was vigorously stirred for 16 h before the reaction was quenched with sat. aq. NH_4Cl (10 mL) and the mixture was extracted with EtOAc (3x 200 mL). The combined organic extracts were washed with brine (20 mL), dried over $MgSO_4$ and concentrated *in vacuo*. The residue was purified by flash chromatography (hexanes/*tert*-butyl methyl ether, 5:1), furnishing the title compound as a colorless oil (20.60 g, 94% yield). $[\alpha]_D^{25}$ = +3.8° (c = 1.0, $CHCl_3$); ^{1}H NMR (400 MHz, $CDCl_3$, mixture of diastereomers): δ = 5.95 – 5.72 (m, 2H), 5.34 (ddq, *J* = 17.2, 4.4, 1.5 Hz, 1H), 5.23 (ddq, *J* = 11.0, 8.5, 1.3 Hz, 1H), 5.03 (dp, *J* = 17.1, 1.7 Hz, 1H), 4.96 (dt, *J* = 10.1, 1.6 Hz, 1H), 4.70 – 4.56 (m, 2H), 4.20 (dtd, *J* = 7.0, 5.9, 3.8 Hz, 0.5H), 4.14 – 4.05 (m, 0.5H), 3.83 (ddd, *J* = 13.1, 4.4, 1.1 Hz, 0.5H), 3.72 (dd, *J* = 13.3, 5.9 Hz, 0.5H), 3.54 – 3.39 (m, 1H), 2.63 (ddd, *J* = 13.9, 5.8, 1.1 Hz, 0.5H), 2.48 (ddd, *J* = 13.9, 6.5, 0.9 Hz, 0.5H), 2.23 – 1.90 (m, 4H), 1.70 (dd, *J* = 13.9, 7.1 Hz, 0.6H), 1.52 (s, 9.4H), 0.87 (d, *J* = 4.4 Hz, 9H), 0.13 – 0.03 (m, 6H); ^{13}C NMR (101 MHz, $CDCl_3$, mixture of diastereomers): δ = 171.3, 171.3, 169.8, 169.2, 152.7, 152.6, 137.6, 137.5, 131.4, 131.2, 119.0, 118.4, 115.1, 83.1, 83.1, 66.2, 66.0, 64.0, 63.9, 55.5, 54.7, 51.2, 51.0, 38.8, 35.8, 35.4, 29.0, 28.6, 27.9, 25.7, 25.6, 18.1, 17.9, –4.8, –4.8, –5.0; IR (film): $\tilde{\nu}$ = 2955, 2931, 2897, 2858, 1777, 1723, 1642, 1472, 1462, 1392, 1368, 1302, 1256, 1151, 1126, 985, 914, 870, 838, 810, 778 cm^{-1}. HRMS (ESI): *m/z* calcd. for $C_{24}H_{41}NO_6SiNa$ [M+Na$^+$]: 490.25954, found: 490.25960.

***tert*-Butyl (*S*)-5-(but-3-en-1-yl)-3-((*tert*-butyldimethylsilyl)oxy)-6-oxo-3,6-dihydropyridine-1(2*H*)-carboxylate (17).**

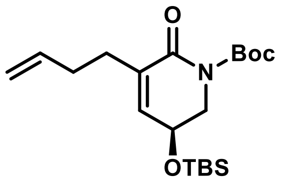

$Pd_2(dba)_3 \cdot CHCl_3$ (1.07 mmol, 1.11 g) was added to a solution of compound **16** (21.38 mmol, 10.00 g) in anhydrous MeCN (86 mL). The mixture was stirred at 80 °C for 30 min. The crude mixture was filtered through a plug of Celite, which was carefully washed with *tert*-butyl methyl ether. The combined filtrates were concentrated in vacuo and the resulting crude material was purified by flash chromatography on silica (toluene, then hexane/*tert*-butyl methyl ether, 10:1) to furnish the title compound as a colorless oil (6.77 g, 83% yield). $[\alpha]_D^{25}$ = +62.6° (c = 1.0, $CHCl_3$); ^{1}H NMR (400 MHz, $CDCl_3$): δ = 6.38 (dq, *J* = 3.4, 1.1 Hz, 1H), 5.79 (ddt, *J* = 16.9, 10.2, 6.6 Hz, 1H), 5.05 – 4.94 (m, 2H), 4.43 (dddt, *J* = 8.0, 4.7, 3.3, 1.3 Hz, 1H), 3.88 (ddd, *J* = 12.8, 4.8, 1.1 Hz, 1H), 3.65 (dd, *J* = 12.8, 7.9 Hz, 1H), 2.38 (ddt, *J* = 8.5, 5.8, 1.3 Hz, 2H), 2.29 – 2.18 (m, 2H), 1.54 (s, 9H), 0.89 (s, 9H), 0.10 (s, 6H); ^{13}C NMR (101 MHz, $CDCl_3$): δ = 163.8, 152.8, 141.0, 137.7, 135.3, 115.2, 83.1, 63.8, 50.7, 32.3, 29.6, 28.1, 25.7, 18.1, –4.7, –4.7; IR (film): $\tilde{\nu}$ = 2955, 2930, 2889, 2858, 1768, 1716, 1651, 1472, 1389, 1368, 1337, 1303, 1256, 1194, 1149,

1091, 1034, 1005, 980, 954, 913, 876, 837, 810, 778 cm^{-1}. HRMS (ESI): *m/z* calcd. for $C_{20}H_{35}NO_4SiNa$ $[M+Na^+]$: 404.22276, found: 404.22262.

Allyl 1-benzyl-4-hydroxy-1,2,5,6-tetrahydropyridine-3-carboxylate (19b). NaH (127.81 mmol, 3.07 g) was transferred into a Schlenk flask before anhydrous THF (54 mL) was added. The mixture was cooled to 0 °C and a solution of 1-benzyl-4-piperidone (51.12 mmol, 9.5 mL) in THF (16.6 mL) was added dropwise. Once the addition was complete, the mixture was warmed to room temperature before diallyl carbonate (76.68 mmol, 11.0 mL) was added. The resulting mixture was stirred at room temperature for 18 h before sat. aq. NH_4Cl (30 mL) was carefully added to quench the reaction. The aqueous phase was diluted with H_2O (5 mL) and extracted with EtOAc (3 x 150 mL). The combined extracts were washed with brine (20 mL), dried over $MgSO_4$ and concentrated *in vacuo*. The residue was purified by flash chromatography on silica (hexanes/EtOAc, 5:1), furnishing the title compound as a colorless oil (6.35 g, 45% yield). ^{1}H NMR (400 MHz, $CDCl_3$): δ = 11.93 (s, 0.7H), 7.37 – 7.27 (m, 5H), 5.91 (dddt, *J* = 17.2, 10.4, 9.1, 5.6 Hz, 1H), 5.38 – 5.19 (m, 2H), 4.73 – 4.56 (m, 2H), 3.65 (d, *J* = 2.5 Hz, 2H), 3.50 (ddd, *J* = 7.9, 5.0, 1.3 Hz, 0.25H), 3.24 (t, *J* = 1.8 Hz, 1.5H), 3.08 (ddd, *J* = 11.6, 7.8, 1.2 Hz, 0.25H), 2.96 (ddd, *J* = 11.7, 5.0, 1.7 Hz, 0.25H), 2.84 (dddd, *J* = 11.7, 6.3, 5.6, 1.7 Hz, 0.25H), 2.75 (dddd, *J* = 11.3, 8.1, 4.8, 1.2 Hz, 0.25H), 2.64 – 2.50 (m, 2H), 2.41 (td, *J* = 5.9, 3.1 Hz, 1.5H); ^{13}C NMR (101 MHz, $CDCl_3$): δ = 203.9, 170.7, 170.6, 168.5, 137.8, 132.0, 131.6, 129.0, 128.8, 128.4, 128.4, 127.4, 127.3, 118.7, 118.1, 96.7, 65.8, 64.8, 62.0, 61.6, 56.6, 55.1, 53.1, 50.0, 48.5, 40.8, 29.4; IR (film): $\tilde{\nu} = 3063$, 3028, 2935, 2808, 2764, 1743, 1720, 1664, 1622, 1495, 1453, 1418, 1403, 1367, 1350, 1302, 1285, 1233, 1212, 1193, 1168, 1126, 1078, 1052, 1028, 994, 972, 934, 815, 742, 699 cm^{-1}. HRMS (ESI): *m/z* calcd. for $C_{16}H_{20}NO_3$ $[M+H^+]$: 274.14377, found: 274.14376.

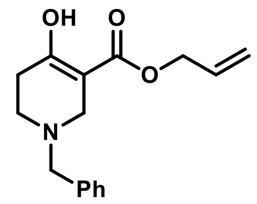

Allyl 1-benzyl-4-oxo-3-(pent-3-yn-1-yl)piperidine-3-carboxylate (21). 5-Iodopent-2-yne (58.06 mmol, 14.72 g)[1] and caesium carbonate (60.38 mmol, 19.67 g) were added in three portions (1:1:0.5) to a solution of compound **19b** (23.22 mmol, 6.35 g) in anhydrous DMF (24 mL) at room temperature (the second and third portion were added after 30 min and 1h, respectively). The mixture was stirred for 3 h, before the reaction was quenched with sat. aq. NH_4Cl (15 mL). The aqueous phase was extracted with EtOAc (3 x 250 mL), the combined organic extracts were washed with brine (30 mL), dried over $MgSO_4$ and concentrated *in vacuo*. The residue was purified by flash chromatography on silica (hexanes/EtOAc, 5:1) to give the title compound as a colorless oil (7.15 g, 91% yield). ^{1}H NMR (400 MHz, $CDCl_3$): δ = 7.29 – 7.20 (m, 5H), 5.82 (ddt, *J* = 17.2, 10.4, 5.8 Hz, 1H), 5.32 – 5.14 (m, 2H), 4.58 (qdt, *J* = 13.1, 5.8, 1.4 Hz, 2H), 3.52 (d, *J* = 1.9 Hz,

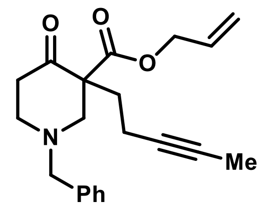

2H), 3.36 (dd, *J* = 11.6, 2.6 Hz, 1H), 2.92 (dtd, *J* = 12.8, 5.9, 3.5 Hz, 1H), 2.79 (ddd, *J* = 16.0, 12.3, 6.6 Hz, 1H), 2.39 – 2.28 (m, 2H), 2.28 – 2.12 (m, 2H), 2.04 – 1.86 (m, 2H), 1.76 – 1.68 (m, 1H), 1.67 (t, *J* = 2.4 Hz, 3H); ^{13}C NMR (101 MHz, $CDCl_3$): δ = 205.8, 170.9, 137.7, 131.5, 128.8, 128.3, 127.4, 118.9, 78.3, 75.9, 65.9, 61.8, 61.1, 60.7, 53.4, 40.5, 31.7, 14.5, 3.5; IR (film): $\tilde{\nu}$ = 3028, 2957, 2919, 2807, 1717, 1649, 1495, 1453, 1423, 1348, 1316, 1227, 1186, 1121, 1076, 1059, 1029, 1000, 971, 936, 742, 699 cm^{-1}. HRMS (ESI): *m/z* calcd. for $C_{21}H_{26}NO_3$ [M+H$^+$]: 340.19072, found: 340.19053.

3-Allyl 1-methyl 4-oxo-3-(pent-3-yn-1-yl)piperidine-1,3-dicarboxylate (20). Methyl chloroformate (73.65 mmol, 5.7 mL) was added to a solution of compound **21** (14.73 mmol, 5.00 g) in toluene (21 mL). The reaction was stirred at 100 °C for 14 h. The mixture was cooled to room temperature and concentrated *in vacuo.* The residue was purified by flash chromatography on silica (hexanes/EtOAc, 3:1 to 2:1), furnishing the title compound as a yellow oil (4.52 g, quant.).

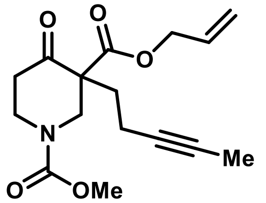

^{1}H NMR (400 MHz, $CDCl_3$): δ = 5.86 (ddt, *J* = 16.5, 9.9, 5.8 Hz, 1H), 5.36 – 5.20 (m, 2H), 4.65 – 4.50 (m, 3H), 4.27 – 3.93 (br, 1H), 3.73 (s, 3H), 3.39 (br, 1H), 3.22 (d, *J* = 13.7 Hz, 1H), 2.68 (ddd, *J* = 14.1, 9.8, 6.3 Hz, 1H), 2.48 (dt, *J* = 14.7, 4.7 Hz, 1H), 2.28 – 2.02 (m, 3H), 1.86 (br, 1H), 1.76 – 1.68 (m, 3H); ^{13}C NMR (101 MHz, $CDCl_3$): δ = 203.9, 169.4, 155.6, 131.1, 119.3, 77.8, 76.5, 66.3, 60.6, 53.1, 50.1, 43.6, 39.6, 31.1, 14.3, 3.4; IR (film): $\tilde{\nu}$ = 2956, 2920, 2860, 1699, 1650, 1447, 1474, 1413, 1375, 1264, 1238, 1220, 1189, 1130, 1067, 1028, 995, 935, 876, 767, 528 cm^{-1}. HRMS (ESI): *m/z* calcd. for $C_{16}H_{21}NO_5Na$ [M+Na$^+$]: 330.13119, found: 330.13101.

Methyl 4-oxo-5-(pent-3-yn-1-yl)-3,4-dihydropyridine-1(2*H*)-carboxylate (22). $Pd_2(dba)_3{\cdot}CHCl_3$ (0.73 mmol, 668 mg) was added to a solution of compound **20** (14.60 mmol, 4.49 g) in anhydrous MeCN (59 mL). The mixture was stirred at 80 °C for 30 min before it was cooled to ambient temperature and filtered through a plug of Celite, which was carefully washed with *tert*-butyl methyl ether. The combined filtrates were concentrated in vacuo and the resulting crude material was purified by flash chromatography on silica (hexane/EtOAc, 3:1 to 1:1) to give the title compound as a white solid (3.10 g, 96% yield).

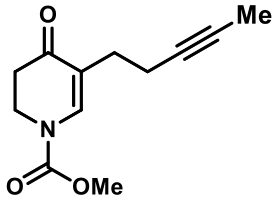

M.p. = 69.8-70.5 °C; ^{1}H NMR (400 MHz, $CDCl_3$): δ = 7.77 (br, 1H), 3.98 (t, *J* = 7.3 Hz, 2H), 3.85 (s, 3H), 2.57 – 2.51 (m, 2H), 2.35 – 2.28 (m, 2H), 2.24 (dddd, *J* = 7.7, 6.1, 2.9, 2.1 Hz, 2H), 1.74 (t, *J* = 2.5 Hz, 3H); ^{13}C NMR (101 MHz, $CDCl_3$): δ = 192.8, 153.4, 140.9, 117.3, 78.4, 76.6, 53.9, 42.6, 35.8, 26.9, 18.7, 3.4; IR (film): $\tilde{\nu}$ = 2956, 2919, 2857, 1722, 1662, 1615, 1440, 1399, 1369, 1322, 1300, 1245, 1204, 1174, 1122, 1077, 1049, 1017, 969, 927, 909, 868, 767, 666, 512, 484, 438 cm^{-1}. HRMS (ESI): *m/z* calcd. for $C_{12}H_{15}NO_3Na$ [M+Na$^+$]: 244.09441, found: 244.09442.

Compound 24. A solution of LiO*t*Bu (2.89 mmol, 223 mg) in THF (6 mL) was added dropwise to a solution

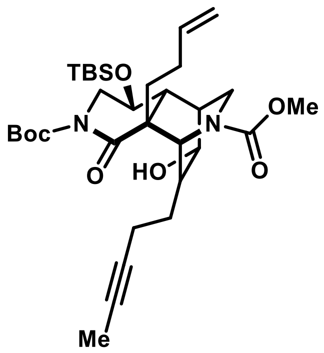

of compound **22** (2.79 mmol, 617 mg) in anhydrous THF (11 mL) at –50 °C. The resulting red solution was stirred for 10 min before a solution of compound **17** (2.33 mmol, 887 mg) in THF (5 mL) was added. The mixture was warmed to room temperature over the course of 5 h and stirring was continued for another 16 h. Next, 4-dimethyl-aminopyridine (4.65 mmol, 568 mg) and di-*tert*-butyl dicarbonate (4.65 mmol, 1.1 mL) were added and the resulting mixture was stirred for 1 h. sat. aq. NH_4Cl (10 mL) was carefully introduced to quench the reaction. The aqueous phase was extracted with EtOAc (3 x 150 mL) and the combined extracts were washed with brine (20 mL), dried over $MgSO_4$ and concentrated *in vacuo*. The crude residue was purified by flash chromatography on silica (hexane/*tert*-butyl methyl ether, 10:1; then hexane/EtOAc, 10:1), furnishing compound **23** as a white foam which was used in the next step without further purification.

$NaBH_4$ (9.42 mmol, 356 mg) was added in portions to a solution of **23** in methanol (15.9 mL) at 0 °C. The mixture was stirred for 20 min, before the reaction was quenched with sat. aq. NH_4Cl (5 mL) at this temperature. The aqueous phase was extracted with EtOAc (3 x 100 mL) and the combined extracts were washed with brine (10 mL), dried over $MgSO_4$ and concentrated *in vacuo*. The residue was purified by flash chromatography on silica (pentane/*tert*-butyl methyl ether, 3:1) to furnish the title compound as a white foam (742 mg, 53% yield over 2 steps). $[\alpha]_{\mathrm{D}}^{25}$ = –66.9° (c = 1.0, $CHCl_3$); ^{1}H NMR (400 MHz, $CDCl_3$, mixture of rotamers): δ = 5.76 – 5.60 (m, 1H), 5.00 – 4.83 (m, 2H), 4.49 (tdd, *J* = 10.6, 4.1, 2.2 Hz, 1H), 4.32 (s, 0.3H, minor), 4.20 (s, 0.7H, major), 4.10 (ddd, *J* = 12.4, 4.2, 1.8 Hz, 1H), 3.68 (s, 3H), 3.66 – 3.61 (m, 1H), 3.31 (ddd, *J* = 20.7, 11.4, 3.0 Hz, 1H), 3.18 – 3.05 (m, 2H), 2.44 – 2.01 (m, 5H), 2.00 – 1.84 (m, 1H), 1.82 – 1.56 (m, 8H), 1.52 (s, 10H), 0.88 (s, 9H), 0.10 (d, *J* = 3.3 Hz, 6H); ^{13}C NMR (101 MHz, $CDCl_3$, mixture of rotamers): δ = 172.2, 172.1, 156.7, 156.6, 151.8, 151.7, 137.9, 137.4, 115.0, 114.7, 83.3, 83.2, 79.1, 78.9, 76.5, 76.4, 75.5, 75.3, 67.7, 67.7, 52.7, 52.6, 52.6, 52.5, 52.4, 52.1, 52.1, 51.6, 50.0, 49.9, 48.1, 46.2, 46.0, 40.2, 39.8, 34.3, 34.2, 32.3, 32.3, 28.3, 28.1, 28.0, 25.8, 17.9, 16.4, 16.4, 3.4, –4.5, –4.5, –4.6; IR (film): $\tilde{\nu} = 3493$, 2952, 2930, 2885, 2857, 1766, 1707, 1681, 1641, 1453, 1394, 1369, 1338, 1298, 1256, 1190, 1156, 1125, 1074, 1005, 914, 865, 839, 779 cm^{-1}. HRMS (ESI): *m/z* calcd. for $C_{32}H_{52}N_2O_7SiNa$ [M+Na$^+$]: 627.34360, found: 627.34354.

Compound 25. Triethylamine (69.64 mmol, 9.7 mL), 4-dimethylaminopyridine (11.16 mmol, 1.36 g) and methanesulfonyl chloride (27.68 mmol, 2.14 mL) were successively added to a solution of compound **24** (4.46 mmol, 2.70 g) in CH_2Cl_2 (22 mL) at 0 °C. The mixture was warmed to room temperature after 5 min and stirred for 1 h. The reaction was quenched with sat. aq. $NaHCO_3$ (20 mL) and the aqueous phase extracted with *tert*-butyl methyl ether (3 x 250 mL). The combined organic extracts were washed with brine (50 mL), dried over $MgSO_4$ and concentrated *in vacuo*. The crude product was purified by flash chromatography on silica (hexane/EtOAc, 5:1 to 4:1), furnishing the title compound as a white foam (2.79 g, 91% yield). $[\alpha]_D^{25}$ = –30.7° (c = 1.0, $CHCl_3$); ^{1}H NMR (400 MHz, $CDCl_3$, mixture of rotamers): δ = 5.68 (dtt, *J* = 17.0, 10.5, 6.5 Hz, 1H), 5.01 – 4.86 (m, 2H), 4.52 – 4.27 (m, 2H), 4.26 – 4.14 (m, 2H), 3.71 (d, *J* = 4.8 Hz, 3H), 3.38 (ddd, *J* = 10.9, 8.3, 2.6 Hz, 1H), 3.30 – 3.12 (m, 2H), 3.04 (d, *J* = 5.0 Hz, 4H), 2.59 (dp, *J* = 17.1, 2.6 Hz, 1H), 2.48 – 2.03 (m, 3H), 2.02 – 1.83 (m, 1H), 1.83 – 1.59 (m, 7H), 1.52 (s, 9H), 0.88 (d, *J* = 0.9 Hz, 9H), 0.14 (d, *J* = 6.1 Hz, 6H); ^{13}C NMR (101 MHz, $CDCl_3$, mixture of rotamers) δ = 171.2, 171.1, 156.5, 156.4, 151.4, 151.3, 137.5, 137.0, 115.3, 115.0, 84.6, 84.3, 83.6, 83.5, 78.5, 78.1, 76.3, 76.2, 67.8, 67.7, 52.9, 52.2, 51.8, 51.6, 50.3, 49.8, 49.3, 49.2, 48.0, 42.8, 42.8, 40.0, 39.5, 38.8, 38.7, 34.0, 33.8, 31.8, 31.7, 28.3, 28.1, 28.0, 25.8, 17.9, 16.1, 16.0, 3.5, 3.5, –4.3, –4.3, –4.5, –4.6; IR (film): $\tilde{\nu}$ = 2953, 2931, 2857, 1770, 1704, 1641, 1450, 1389, 1366, 1338, 1297, 1256, 1176, 1155, 1125, 1096, 1051, 994, 964, 941, 897, 838, 779, 754, 686, 666, 527 cm^{-1}. HRMS (ESI): *m/z* calcd. for $C_{33}H_{54}N_2O_9SSiNa$ [M+Na$^+$]: 705.32115, found: 705.32108.

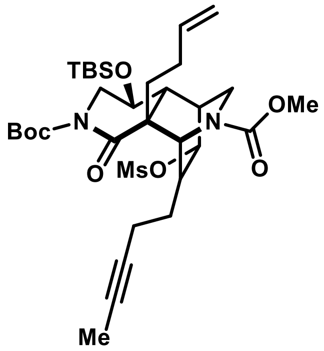

Compound 26. A solution of mesylate **25** (3.83 mmol, 2.616 g) in 2,6-lutidine (21 mL) was stirred at 170 °C for 5 d. The mixture was cooled to 0 °C before CH_2Cl_2 (22 mL) and *tert*-butyldimethylsilyl trifluoromethanesulfonate (15.32 mmol, 3.52 mL) were added. Stirring was continued at room temperature for 45 min before sat. aq. $NaHCO_3$ (5 mL) was added at 0 °C. Next, the mixture was poured into a solution of HCl (2 M, 45 mL), which was vigorously stirred for 15 min. The aqueous phase was extracted with EtOAc (3 x 200 mL), the combined organic extracts were washed with sat. aq. $NaHCO_3$ (50mL) and brine (25 mL), before they were dried over $MgSO_4$ and concentrated *in vacuo*. The residue was purified by flash chromatography on silica (CH_2Cl_2/*tert*-butyl methyl ether, 6:1), furnishing the title compound as a white foam (1.357 g, 73% yield). $[\alpha]_D^{25}$ = –69.4° (c = 1.0, $CHCl_3$); ^{1}H NMR (400 MHz, $CDCl_3$, mixture of rotamers): δ = 6.43 (d, *J* = 6.2 Hz, 0.3H, minor), 6.37 (d, 0.7H, major), 5.94 (dd, *J* = 10.2, 6.6 Hz, 1H), 5.72 (dddd, *J* = 16.7, 13.0, 10.2, 6.0 Hz, 1H), 5.04 – 4.86 (m,

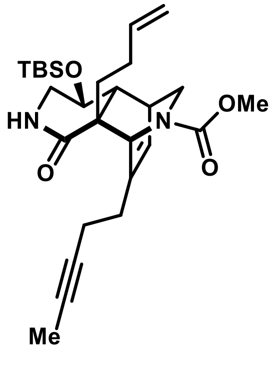

2.3H, minor), 4.80 (d, *J* = 1.6 Hz, 0.7H, major), 3.69 (s, 2H), 3.66 (s, 1H), 3.37 (tdd, *J* = 9.4, 5.0, 1.7 Hz, 1H), 3.22 – 2.92 (m, 4H), 2.84 – 2.72 (m, 1H), 2.48 – 2.14 (m, 5H), 2.14 – 1.96 (m, 1H), 1.77 – 1.55 (m, 6H), 0.89 (s, 9H), 0.08 (d, *J* = 16.6 Hz, 6H); ^{13}C NMR (101 MHz, $CDCl_3$, mixture of rotamers): δ = 173.5, 173.5, 156.1, 156.1, 146.6, 145.9, 138.3, 137.8, 125.6, 125.1, 114.8, 114.5, 78.5, 78.1, 75.9, 75.5, 70.9, 70.8, 54.3, 54.2, 52.8, 52.5, 52.4, 52.3, 51.5, 51.5, 47.2, 47.0, 45.6, 39.8, 39.5, 33.7, 33.4, 33.2, 33.1, 28.6, 28.3, 25.6, 17.8, 16.9, 16.8, 3.4, 3.4, –4.3, –4.4, –4.8, –4.8; IR (film): $\tilde{\nu}$ = 3209, 3075, 2953, 2929, 2896, 2857, 1702, 1667, 1448, 1389, 1345, 1329, 1300, 1273, 1257, 1220, 1191, 1120, 1091, 1006, 956, 913, 873, 838, 776, 685 cm^{-1}. HRMS (ESI): *m/z* calcd. for $C_{27}H_{42}N_2O_4SiNa$ [M+Na$^+$]: 509.28061, found: 509.28065.

Compound 27. A solution of amide **26** (2.79 mmol, 1.357 g) in DMF (2 mL) and 7-iodohept-2-yne (9.76 mmol, 2.166 g)[2] were successively added to a mixture of NaH (41.81 mmol, 1.003 g) in DMF (25 mL) at 0 °C. The mixture was stirred for 30 min before sat. aq. NH_4Cl (5 mL) was carefully added. The aqueous phase was diluted with H_2O (5 mL) and extracted with EtOAc (3 x 100 mL). The combined organic extracts were washed with brine (20 mL), dried over $MgSO_4$ and concentrated *in vacuo*. The residue was purified by flash chromatography on silica (pentane/*tert*-butyl methyl ether, 4:1), furnishing the title compound as a colorless oil (1.545 g, 95% yield). $[\alpha]_D^{25}$ = –54.7° (c = 1.0, $CHCl_3$); ^{1}H NMR (400 MHz, $CDCl_3$, mixture of rotamers): δ = 5.92 (t, *J* = 7.7 Hz, 1H), 5.73 (dddt, *J* = 16.8, 13.2, 10.2, 6.5 Hz, 1H), 5.04 – 4.85 (m, 3H), 3.71 (s, 2H, major), 3.66 (s, 1H, minor), 3.38 (dt, *J* = 13.8, 7.0 Hz, 1H), 3.34 – 3.21 (m, 2H), 3.21 – 3.11 (m, 2H), 3.03 (dd, *J* = 10.5, 2.8 Hz, 0.7H, major), 2.99 – 2.92 (m, 1.3H, minor), 2.81 – 2.70 (m, 1H), 2.47 – 2.08 (m, 7H), 2.08 – 1.95 (m, 1H), 1.75 (t, *J* = 2.5 Hz, 3H), 1.71 (t, *J* = 2.3 Hz, 3H), 1.69 – 1.47 (m, 5H), 1.45 – 1.33 (m, 2H), 0.91 (s, 9H), 0.10 (d, *J* = 11.7 Hz, 6H); ^{13}C NMR (101 MHz, $CDCl_3$, mixture of rotamers): δ = 170.4, 156.2, 156.1, 147.0, 146.3, 138.5, 138.1, 125.2, 124.6, 114.6, 114.3, 78.6, 78.6, 78.5, 78.3, 75.9, 75.8, 75.4, 70.7, 70.6, 54.8, 54.7, 53.1, 52.9, 52.4, 52.3, 52.1, 52.0, 51.0, 47.1, 46.9, 46.9, 40.0, 39.7, 33.7, 33.4, 33.1, 33.0, 28.7, 28.4, 26.6, 26.6, 25.9, 25.7, 18.4, 18.3, 17.8, 16.8, 16.8, 3.4, 3.4, –4.3, –4.4, –4.8, –4.8; IR (film): $\tilde{\nu}$ = 2951, 2928, 2857, 1701, 1645, 1446, 1389, 1347, 1328, 1299, 1259, 1190, 1161, 1104, 1088, 1049, 1005, 956, 908, 871, 837, 811, 776 cm^{-1}. HRMS (ESI): *m/z* calcd. for $C_{34}H_{52}N_2O_4SiNa$ [M+Na$^+$]: 603.35886, found: 603.35906.

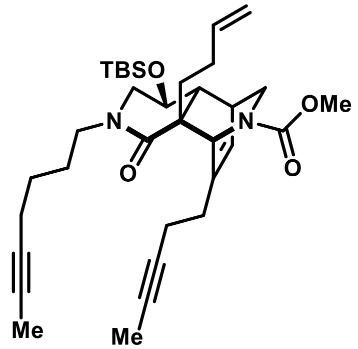

Compound 28. A solution of the molybdenum complex **31** (0.45 mmol, 351 mg)[3] in toluene (10 mL) was added dropwise to a suspension comprising diyne **27** (2.26 mmol, 1.310 g) and powdered MS (5 Å, 30 g) in toluene (1.17 l) at reflux temperature. After stirring for 10 min, EtOH (10 mL) was added, the mixture was cooled to room temperature and filtered through a short pad of Celite, which was carefully rinsed with EtOAc. The combined filtrates were concentrated *in vacuo* and the residue was purified by flash chromatography on silica (toluene/EtOAc, 8:1), furnishing the title compound as a white solid (983 mg, 83% yield). M.p. = 163.9-165.1 °C; $[\alpha]_D^{25}$ = –102.4° (c = 0.5, $CHCl_3$); ^1H NMR (400 MHz, $CDCl_3$, mixture of rotamers): δ = 6.00 – 5.89 (m, 1H), 5.73 (dddt, *J* = 16.8, 13.1, 10.1, 6.5 Hz, 1H), 5.04 – 4.80 (m, 3H), 4.04 – 3.92 (m, 1H), 3.72 – 3.59 (m, 4H), 3.39 (dd, *J* = 12.3, 10.6 Hz, 1H), 3.19 (dd, *J* = 10.5, 2.0 Hz, 1H), 3.06 – 2.89 (m, 2H), 2.89 – 2.77 (m, 1H), 2.70 – 2.51 (m, 1H), 2.47 – 2.03 (m, 8H), 1.92 (ddt, *J* = 16.3, 13.2, 3.1 Hz, 1H), 1.74 – 1.51 (m, 4H), 1.40 – 1.27 (m, 1H), 1.27 – 1.08 (m, 1H), 0.89 (s, 9H), 0.09 (d, *J* = 25.2 Hz, 6H); ^{13}C NMR (101 MHz, $CDCl_3$, mixture of rotamers): δ = 170.0, 170.0, 156.3, 146.0, 145.2, 138.6, 138.2, 123.4, 122.9, 114.6, 114.3, 81.0, 80.9, 79.3, 79.2, 70.4, 70.3, 55.2, 54.7, 54.7, 54.5, 54.2, 52.4, 52.4, 52.1, 52.0, 50.6, 47.2, 47.0, 39.8, 39.5, 33.9, 33.6, 32.2, 32.1, 28.7, 28.5, 26.2, 26.1, 25.7, 18.7, 17.8, 14.1, –4.2, –4.2, –4.6, –4.7; IR (film): $\tilde{\nu}$ = 2953, 2928, 2857, 1699, 1640, 1449, 1423, 1390, 1350, 1319, 1262, 1218, 1190, 1170, 1157, 1140, 1103, 1085, 1051, 1006, 955, 909, 870, 836, 809, 775, 754, 723, 712, 683, 665, 442 cm^{-1}. HRMS (ESI): *m/z* calcd. for $C_{30}H_{46}N_2O_4SiNa$ [$M+Na^+$]: 549.31191, found: 549.31220.

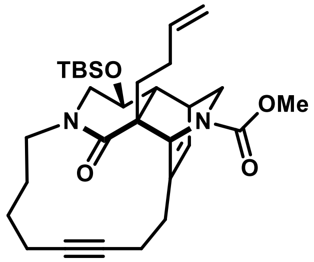

Table S2. RCAM of Diyne **27**: Reaction Optimization*a*

Entry	Catalyst	Temperature	Yield of 28
1	**31** (10 mol%)	110°C	63%
2	**31** (20 mol%)	110°C	83%
3	**29** (25 mol%) + **30** (30 mol%)	100°C	79%

a All reactions were performed in toluene in the presence of MS 5Å

Compound 32. L-Selectride (1 M in THF, 8.28 mmol, 8.28 mL) was added to a solution of carbamate **28** (2.07 mmol, 1.090 g) in THF (19 mL). The mixture was stirred at 40 °C for 16 h. Next, the mixture was cooled to 0 °C before MeOH (5 mL) was carefully added. The solution was concentrated *in vacuo* and the residue was purified by flash chromatography on silica (CH_2Cl_2/MeOH, 95:5 to 90:10), furnishing the title compound as a yellow oil (878 mg, 91% yield). $[\alpha]_D^{25}$ = –45.5° (c = 0.4, $CHCl_3$); ^{1}H NMR (400 MHz, $CDCl_3$): δ = 5.97 (dd, *J* = 6.8, 2.1 Hz, 1H), 5.84 (ddt, *J* = 16.8, 10.2, 6.4 Hz, 1H), 5.05 (dq, *J* = 17.1, 1.7 Hz, 1H), 4.94 (dq, *J* = 10.2, 1.3 Hz, 1H), 4.04 – 3.92 (m, 1H), 3.86 (s, 1H), 3.65 (ddd, *J* = 10.6, 8.8, 4.5 Hz, 1H), 3.38 (dd, *J* = 12.2, 10.6 Hz, 1H), 2.99 – 2.85 (m, 2H), 2.77 (dd, *J* = 7.0, 2.4 Hz, 1H), 2.62 – 2.07 (m, 10H), 1.95 (dddd, *J* = 22.0, 19.4, 12.6, 4.1 Hz, 2H), 1.72 (ddd, *J* = 13.7, 11.3, 5.5 Hz, 1H), 1.66 – 1.52 (m, 2H), 1.34 (tq, *J* = 12.3, 3.4 Hz, 1H), 1.28 – 1.11 (m, 2H), 0.90 (s, 9H), 0.10 (d, *J* = 25.8 Hz, 6H); ^{13}C NMR (101 MHz, $CDCl_3$) δ = 170.6, 146.2, 138.5, 122.6, 114.5, 81.2, 79.2, 70.4, 55.6, 55.2, 54.3, 50.7, 50.6, 45.1, 39.7, 33.4, 31.9, 28.8, 26.3, 26.2, 25.7, 18.8, 17.9, 14.2, –4.2, –4.6; IR (film): $\tilde{\nu}$ = 2952, 2927, 2856, 1638, 1484, 1452, 1422, 1388, 1357, 1327, 1258, 1171, 1141, 1092, 1006, 924, 910, 868, 836, 804, 775, 750, 678, 664, 439 cm^{-1}. HRMS (ESI): *m/z* calcd. for $C_{28}H_{45}N_2O_2Si$ [M+H$^+$]: 469.32448, found: 469.32463.

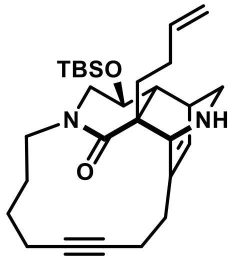

Compound 33. $NaBH(OAc)_3$ (39.0 mg, 0.18 mmol) was added to a solution of secondary amine **32** (43.2 mg, 0.09 mmol) and 5-hexenal (40.7 mg, 0.42 mmol) in CH_2Cl_2 (0.9 mL) and the resulting mixture was stirred at ambient temperature for 3 h. The mixture was diluted with CH_2Cl_2 (5 mL) and the reaction quenched with sat. aq. $NaHCO_3$ (5 mL). The aqueous phase was extracted with EtOAc (3 x 20 mL), the combined organic fractions were dried over Na_2SO_4 and concentrated *in vacuo*. The residue was purified by flash chromatography on silica (hexane/EtOAc, 8:1), furnishing the title compound as a colorless oil (48.5 mg, 96% yield). $[\alpha]_D^{25}$ = –24.4° (c = 1.0, $CHCl_3$); ^{1}H NMR (400 MHz, $CDCl_3$): δ = 5.94 (d, *J* = 4.6 Hz, 1H), 5.87 – 5.71 (m, 2H), 4.99 (ddq, *J* = 17.2, 10.4, 1.7 Hz, 2H), 4.94 – 4.87 (m, 2H), 4.04 – 3.91 (m, 1H), 3.65 – 3.53 (m, 2H), 3.35 (dd, *J* = 12.1, 10.6 Hz, 1H), 2.96 (dd, *J* = 9.6, 2.0 Hz, 1H), 2.89 (dd, *J* = 12.1, 4.5 Hz, 1H), 2.58 (ttd, *J* = 8.8, 6.9, 1.8 Hz, 2H), 2.42 (dt, *J* = 11.5, 7.2 Hz, 1H), 2.33 (ddt, *J* = 12.3, 7.9, 4.2 Hz, 4H), 2.26 – 2.00 (m, 8H), 1.90 (ddq, *J* = 15.9, 12.8, 3.0 Hz, 1H), 1.77 (dd, *J* = 9.5, 2.7 Hz, 1H), 1.72 – 1.51 (m, 2H), 1.52 – 1.25 (m, 6H), 1.18 (qd, *J* = 12.2, 3.0 Hz, 1H), 0.89 (s, 9H), 0.09 (d, *J* = 24.2 Hz, 6H); ^{13}C NMR (101 MHz, $CDCl_3$): δ = 171.36, 143.24, 139.22, 139.05, 121.67, 114.19, 113.91, 81.22, 79.09, 70.75, 61.86, 57.68, 54.48, 54.37, 54.05, 52.04, 50.55, 39.35, 34.31, 33.65, 28.92, 27.96, 26.47, 26.20, 26.13, 25.73, 18.82, 17.86, 14.28, -4.25, -4.61; IR (film): $\tilde{\nu}$ = 2929, 2881, 2857, 1642, 1482, 1451, 1419, 1357, 1328, 1287, 1257,

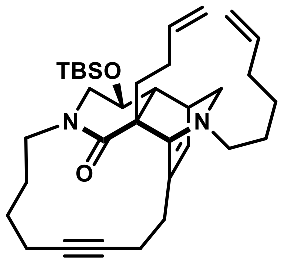

1171, 1157, 1123, 1086, 1042, 1065, 1006, 997, 925, 908, 870, 836, 775, 804 cm^{-1}. HRMS (ESI): *m/z* calcd. for $C_{34}H_{55}N_2O_2Si$ [M+H$^+$]: 551.40273, found: 551.40310.

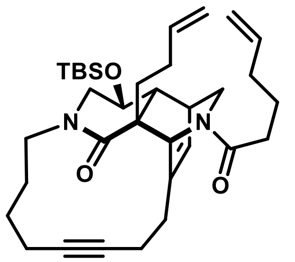

Compound 34. DMF (2 drops) and oxalyl chloride (2.06 mmol, 0.18 mL) were added to a solution of 5-hexenoic acid (1.72 mmol, 0.20 mL) in CH_2Cl_2 (5.5 mL) at 0 °C. The mixture was warmed to room temperature and stirred for 2 h. The resulting solution was added to a solution of amine **32** (1.87 mmol, 878 mg) and triethylamine (9.37 mmol, 1.3 mL) in CH_2Cl_2 (10 mL) at 0 °C. After 5 min, the mixture was warmed to room temperature and stirred for 1 h. sat. aq. $NaHCO_3$ (5 mL) was added and the aqueous phase extracted with EtOAc (3 x 100 mL). The combined organic extracts were washed with brine (10 mL), dried over $MgSO_4$ and concentrated *in vacuo*. The crude product was purified by flash chromatography on silica (hexane/EtOAc, 4:1 to 3:1) to give the title compound as a white foam (756 mg, 71% yield). $[\alpha]_D^{25}$ = –103.0° (c = 1.0, $CHCl_3$); ^1H NMR (400 MHz, $CDCl_3$, mixture of rotamers): δ = 6.03 – 5.92 (m, 1H), 5.85 – 5.63 (m, 2H), 5.50 (d, *J* = 1.5 Hz, 0.4H, minor), 5.07 – 4.85 (m, 4H), 4.61 (d, *J* = 1.5 Hz, 0.6H, major), 4.01 (dt, *J* = 9.6, 3.0 Hz, 1H), 3.68 (dtd, *J* = 10.6, 8.8, 4.6 Hz, 1H), 3.41 (ddd, *J* = 12.3, 10.7, 5.1 Hz, 1H), 3.30 (dd, *J* = 9.5, 2.0 Hz, 0.4H, minor), 3.22 – 3.12 (m, 1.2H, major/major), 3.06 (dd, *J* = 9.5, 2.8 Hz, 0.4H, minor), 3.00 – 2.85 (m, 2H), 2.72 – 2.52 (m, 1H), 2.46 – 1.87 (m, 13H), 1.83 – 1.51 (m, 6H), 1.44 – 1.11 (m, 2H), 0.91 (d, *J* = 0.8 Hz, 9H), 0.11 (d, *J* = 27.2 Hz, 6H); ^{13}C NMR (101 MHz, $CDCl_3$, mixture of rotamers): δ = 172.2, 171.9, 170.2, 169.9, 146.4, 144.4, 138.6, 138.2, 138.0, 137.4, 124.2, 122.8, 115.2, 115.1, 114.3, 81.0, 80.8, 79.5, 79.3, 70.4, 69.9, 58.2, 55.0, 54.3, 54.2, 53.4, 52.5, 52.1, 51.8, 50.7, 50.5, 48.1, 46.5, 40.0, 39.6, 34.2, 33.6, 33.3, 33.3, 33.0, 33.0, 32.9, 32.3, 32.3, 32.2, 29.0, 28.5, 26.3, 26.2, 26.1, 25.7, 24.3, 23.9, 18.8, 18.7, 17.9, 14.2, 14.1, –4.2, –4.2, –4.6, –4.6; IR (film): $\tilde{\nu} = 2952$, 2928, 2857, 1645, 1472, 1484, 1452, 1415, 1358, 1326, 1299, 1260, 1170, 1141, 1120, 1086, 1005, 910, 871, 837, 809, 776 cm^{-1}. HRMS (ESI): *m/z* calcd. for $C_{34}H_{52}N_2O_3SiNa$ [M+Na$^+$]: 587.36394, found: 587.36424.

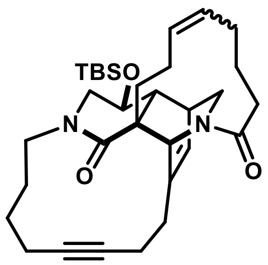

Compound 35. A solution of benzylidene-bis(tricyclohexylphosphino)-dichlororuthenium ("first generation" Grubbs catalyst, 0.009 mmol, 7.3 mg) in toluene (2 mL) was slowly added to a solution of compound **34** (0.018 mmol, 10 mg) in toluene (16 mL) at 100 °C over the course of 2.5 h. After the addition was complete, stirring was continued at 100 °C for another 2h before a solution of potassium 2-isocyanoacetate (0.154 mmol, 19 mg) in MeOH (3 mL) was added at 100 °C. The mixture was cooled to room temperature and stirred for an additional 30 min, before it was concentrated *in vacuo.* The residue was purified by flash chromatography on silica

(hexane/EtOAc, 2:1 to 1:1), furnishing the title compound as a mixture of olefin isomers (9.2 mg, 97% yield, *E*-/*Z*-Isomeric mixture 60:40) as a white solid. ^{1}H NMR (400 MHz, $CDCl_3$, mixture of *E*-/*Z*-Isomers ca. 60:40): δ 5.98 (dd, *J* = 15.7, 6.9 Hz, 1H, major/minor), 5.91 – 5.84 (m, 0.6H, major), 5.59 (s, 0.4H, minor), 5.51 – 5.40 (m, 0.6H, major), 5.32 (td, *J* = 10.3, 5.2 Hz, 0.4H, minor), 5.08 (d, *J* = 1.6 Hz, 0.6H, major), 4.99 (d, *J* = 1.5 Hz, 0.4H, minor), 4.01 (dt, *J* = 9.7, 2.8 Hz, 1H, major/minor), 3.74 – 3.62 (m, 1H, major/minor), 3.39 (ddd, *J* = 12.3, 10.5, 3.4 Hz, 1H, major/minor), 3.27 – 3.08 (m, 2H, major/minor), 2.98 (ddd, *J* = 12.4, 4.4, 2.5 Hz, 1H, major/minor), 2.84 (dq, *J* = 5.4, 2.4 Hz, 1H, major/minor), 2.77 – 2.55 (m, 1H, major/major/minor), 2.46 – 2.23 (m, 7H, major/minor), 2.22 – 2.04 (m, 4H, major/minor), 2.01 – 1.96 (m, 1H, major/minor), 1.90 (td, *J* = 15.3, 14.9, 3.8 Hz, 3H, major/minor), 1.83 – 1.75 (m, 1H, major/minor), 1.66 – 1.51 (m, 3H, major/minor), 1.38 (dddd, *J* = 15.5, 11.2, 7.6, 3.6 Hz, 1H, major/minor), 1.22 – 1.12 (m, 1H, major/minor), 0.91 (d, *J* = 0.9 Hz, 9H, major/minor), 0.14 (s, 3H, major/minor), 0.08 (s, 3H, major/minor); HRMS (ESI): *m*/*z* calcd. for $C_{32}H_{48}N_2O_3SiNa$ [M+Na^{+}]: 559.33209, found: 559.33264.

The isomer mixture was separated by preparative HPLC (two consecutive Multochrom 100-3 Si columns, 250 mm x 20 mm, iso-hexane/isopropanol 95:5, 20 mL/min, λ = 220 nm, t_R (*Z*-Isomer) = 31.0 min). The pure *Z*-isomer analyzed as follows: ^{1}H NMR (600 MHz, $CDCl_3$): δ = 5.95 (d, *J* = 6.6 Hz, 1H), 5.60 (s, 1H), 5.31 (td, *J* = 10.5, 5.4 Hz, 1H), 4.98 (s, 1H), 4.04 – 3.98 (m, 1H), 3.66 (ddd, *J* = 10.1, 8.7, 4.3 Hz, 1H), 3.39 (dd, *J* = 12.3, 10.5 Hz, 1H), 3.22 (dd, *J* = 11.5, 1.5 Hz, 1H), 3.12 (dd, *J* = 12.0, 3.0 Hz, 1H), 2.98 (dd, *J* = 12.3, 4.3 Hz, 1H), 2.83 (dd, *J* = 6.7, 2.6 Hz, 1H), 2.71 (ddd, *J* = 14.3, 12.1, 2.4 Hz, 1H), 2.62 (ddt, *J* = 15.4, 13.6, 2.2 Hz, 1H), 2.44 – 2.32 (m, 4H), 2.31 – 2.26 (m, 1H), 2.26 – 2.17 (m, 2H), 2.16 – 2.03 (m, 4H), 1.98 – 1.84 (m, 3H), 1.79 (s, 1H), 1.70 – 1.56 (m, 3H), 1.43 – 1.31 (m, 1H), 1.23 – 1.12 (m, 1H), 0.90 (s, 9H), 0.13 (s, 3H), 0.07 (s, 3H); ^{13}C NMR (151 MHz, $CDCl_3$): δ = 173.3, 170.3, 145.1, 130.0, 129.6, 123.6, 80.7, 79.4, 70.4, 58.2, 55.6, 54.3, 52.3, 50.9, 47.7, 38.3, 33.2, 31.8, 30.6, 26.3, 26.1, 25.9, 25.7, 25.4, 22.8, 18.8, 17.9, 14.2, –4.3, –4.6.

Compound 40. Tetrabutylammonium fluoride (1 M in THF, 1.78 mmol, 1.78 mL) was added to a solution of TBS-ether **34** (0.89 mmol, 502 mg) in THF (50 mL) at 0 °C. The solution was stirred for 20 min before sat. aq. NH_4Cl (15 mL) was added. The aqueous phase was extracted with EtOAc (3 x 100 mL) and the combined organic extracts were washed with brine (10 mL), dried over $MgSO_4$ and concentrated *in vacuo*. The residue was purified by flash chromatography on silica (hexane/EtOAc, 1:2 to pure EtOAc), furnishing the title compound as a white foam (400 mg, quant.). $[\alpha]_D^{25}$ = –134.2° (c = 0.5, $CHCl_3$); ^{1}H NMR (400 MHz, $CDCl_3$, mixture of rotamers): δ = 6.10 – 6.00 (m, 1H), 5.84 – 5.63 (m, 2H), 5.47 (d, *J* = 1.6 Hz, 0.4H, minor), 5.06 – 4.86 (m, 4H), 4.60 (d, *J* = 1.5 Hz, 0.6H, major), 3.99 (ddt, *J* = 12.3, 5.7, 2.7

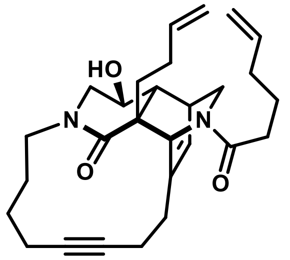

Hz, 1H), 3.81 – 3.66 (m, 1H), 3.45 (ddd, *J* = 12.2, 10.8, 5.8 Hz, 1H), 3.32 (dd, *J* = 9.3, 1.7 Hz, 0.4H, minor), 3.22 – 3.02 (m, 4.6H, major), 2.69 – 2.51 (m, 1H), 2.46 – 2.33 (m, 4H), 2.33 – 1.99 (m, 8H), 1.90 (ddd, *J* = 16.7, 13.2, 3.0 Hz, 1H), 1.80 – 1.68 (m, 3H), 1.68 – 1.52 (m, 3H), 1.36 (tdd, *J* = 14.9, 11.3, 6.7 Hz, 1H), 1.23 – 1.06 (m, 1H); ^{13}C NMR (101 MHz, $CDCl_3$, mixture of rotamers): δ = 172.3, 172.1, 170.3, 170.0, 145.9, 144.2, 138.4, 138.1, 137.9, 137.3, 124.5, 123.2, 115.3, 115.3, 115.1, 114.6, 81.0, 80.8, 79.7, 79.5, 69.3, 68.9, 58.4, 54.4, 54.2, 52.8, 52.6, 52.1, 51.9, 50.7, 50.5, 48.1, 46.7, 40.0, 39.7, 34.2, 33.7, 33.3, 33.2, 33.0, 32.3, 29.1, 28.6, 26.3, 26.2, 26.1, 24.3, 23.9, 18.9, 18.8, 14.1, 14.1; IR (film): $\tilde{\nu}$ = 3364, 2924, 2863, 1635, 1612, 1487, 1418, 1356, 1326, 1264, 1236, 1167, 1137, 1116, 1063, 1034, 996, 911, 831, 812, 751, 685, 665, 646, 579, 443 cm^{-1}. HRMS (ESI): *m/z* calcd. for $C_{28}H_{38}N_2O_3Na$ [M+Na$^+$]: 473.27746, found: 473.27731.

Compound 41. Martin's sulfurane (2.10 mmol, 1.41 g) was added to a mixture of alcohol **40** (0.84 mmol, 378 mg) in toluene (38 mL) at room temperature. The mixture was stirred at 100 °C for 1 h before it was cooled to room temperature and sat. aq. $NaHCO_3$ (10 mL) was added. The aqueous phase was extracted with EtOAc (3 x 100 mL) and the combined organic extracts were washed with brine (10 mL), dried over Na_2SO_4 and concentrated *in vacuo*. The residue was purified by flash chromatography on silica (toluene/EtOAc, 8:1 to 4:1) to furnish the title compound as a colorless oil (351 mg, 97% yield).

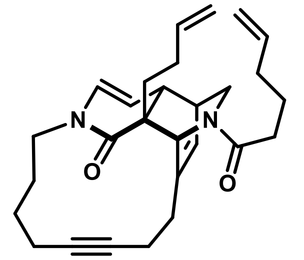

$[\alpha]_D^{25}$ = –121.3° (c = 1.0, $CHCl_3$); ^{1}H NMR (400 MHz, $CDCl_3$, mixture of rotamers): δ = 6.25 – 6.11 (m, 1H), 5.87 – 5.64 (m, 3H), 5.41 (d, *J* = 1.6 Hz, 0.3H, minor), 5.06 – 4.85 (m, 4H), 4.79 (ddd, *J* = 19.5, 8.1, 4.9 Hz, 1H), 4.52 (d, *J* = 1.5 Hz, 0.7H, major), 4.16 (dt, *J* = 14.0, 2.7 Hz, 1H), 3.33 – 3.24 (m, 1H), 2.88 – 2.79 (m, 1H), 2.73 (tt, *J* = 6.4, 1.8 Hz, 1H), 2.61 – 2.31 (m, 5H), 2.31 – 1.95 (m, 10H), 1.92 – 1.64 (m, 3H), 1.58 – 1.33 (m, 4H); ^{13}C NMR (101 MHz, $CDCl_3$, mixture of rotamers): δ = 172.4, 169.1, 169.1, 145.9, 144.1, 138.3, 138.2, 138.0, 137.7, 130.8, 130.5, 125.0, 123.5, 115.2, 115.0, 114.9, 114.5, 105.2, 104.4, 81.4, 81.2, 80.9, 80.7, 59.4, 54.5, 53.5, 53.3, 50.4, 49.2, 48.7, 48.7, 44.6, 43.9, 41.0, 40.8, 37.9, 37.2, 33.4, 33.3, 33.2, 33.0, 32.5, 29.2, 29.1, 27.0, 26.7, 24.2, 23.8, 18.6, 18.6, 14.3; IR (film): $\tilde{\nu}$ = 3072, 2920, 2862, 1639, 1450, 1408, 1398, 1355, 1330, 1308, 1263, 1230, 1197, 1167, 1150, 1132, 1068, 1044, 1026, 995, 910, 852, 825, 750, 724, 695, 646, 608, 591, 434 cm^{-1}. HRMS (ESI): *m/z* calcd. for $C_{28}H_{36}N_2O_2Na$ [M+Na$^+$]: 455.26690, found: 455.26713.

Compound 42. $NaBH_3CN$ (0.89 mmol, 56 mg) and trifluoroacetic acid (1.78 mmol, 0.14 mL) were successively added to a solution of compound **41** (0.18 mmol, 77 mg) in CH_2Cl_2 (7 mL) at 0 °C. The mixture was then stirred at room temperature for 1 h. Next, sat. aq. $NaHCO_3$ (5 mL) was added and the resulting mixture was vigorously stirred for 45 min. The aqueous phase was extracted with EtOAc (3 x 50 mL), the combined organic extracts were washed with brine (10 mL), dried over Na_2SO_4 and concentrated *in vacuo*. The crude material was purified by flash chromatography (hexane/EtOAc, 2:1 to 1:1) to give the title compound as a white solid (57 mg, 73% yield). M.p. = 86.2-86.9 °C; $[\alpha]_{\mathrm{D}}^{25}$ = –153.0° (c = 1.0, $CHCl_3$); ^1H NMR (400 MHz, $CDCl_3$, mixture of rotamers): δ = 6.04 – 5.93 (m, 1H), 5.85 – 5.61 (m, 2H), 5.49 (d, *J* = 1.5 Hz, 0.34H, minor), 5.07 – 4.83 (m, 4H), 4.61 (d, *J* = 1.5 Hz, 0.66H, major), 4.03 (dt, *J* = 12.9, 3.1 Hz, 1H), 3.47 (tdd, *J* = 12.7, 5.4, 2.1 Hz, 1H), 3.29 (dd, *J* = 9.3, 2.1 Hz, 0.34H, minor), 3.21 (dd, *J* = 11.8, 2.0 Hz, 0.66H, major), 3.11 – 2.95 (m, 2H), 2.72 – 2.53 (m, 2H), 2.45 – 2.15 (m, 8H), 2.15 – 1.80 (m, 7H), 1.80 – 1.10 (m, 8H); ^{13}C NMR (101 MHz, $CDCl_3$, mixture of rotamers) δ = 172.1, 171.8, 170.7, 170.4, 145.7, 143.7, 138.7, 138.3, 138.0, 137.6, 124.0, 122.6, 115.2, 115.0, 115.0, 114.2, 81.0, 80.8, 79.8, 79.6, 58.3, 52.6, 51.6, 51.5, 50.7, 50.5, 48.5, 48.2, 48.1, 47.1, 46.0, 44.3, 39.9, 39.7, 37.2, 36.7, 33.3, 33.2, 32.9, 32.4, 32.4, 30.0, 29.6, 29.2, 28.7, 26.4, 26.3, 26.3, 26.2, 24.3, 23.9, 18.9, 18.8, 14.1, 14.1; IR (film): $\tilde{\nu} =$ 3073, 2924, 2859, 1632, 1489, 1451, 1415, 1355, 1342, 1310, 1279, 1229, 1164, 1145, 1109, 1021, 997, 910, 809, 753, 661, 432 cm^{-1}. HRMS (ESI): *m/z* calcd. for $C_{28}H_{39}N_2O_2$ $[M+H^+]$: 435.30060, found: 435.30068.

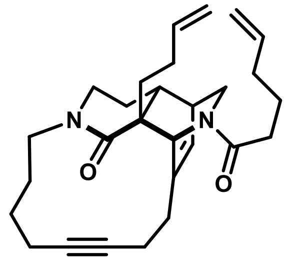

Compound S2. A solution of benzylidene-bis(tricyclohexylphosphino)-dichlororuthenium (Grubbs first generation catalyst, 0.018 mmol, 14.2 mg) in 1,2-dichloroethane (2 mL) was slowly added to a refluxing solution of diene **42** (0.069 mmol, 30 mg) in 1,2-dichloroethane (140 mL) over 10 min. Stirring was continued at reflux temperature for 2 h before a second batch of benzylidene-bis(tricyclohexylphosphino)-dichlororuthenium (0.018 mmol, 14.2 mg) was slowly added as a solution in 1,2-dichloroethane (2mL) over 10 min. After stirring for another 2 h, a solution of potassium 2-isocyanoacetate (0.154 mmol, 19 mg) in MeOH (3 mL) was added at reflux temperature. The mixture was cooled to room temperature and stirred for an additional 30 min. All volatile materials were evaporated *in vacuo* and the residue was purified by flash chromatography on silica (hexane/EtOAc, 1:1 to pure EtOAc) to furnish compound **43** as an mixture of olefin isomers.

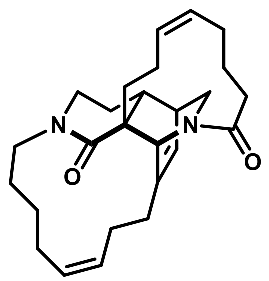

$NaBH_4$ (0.267 mmol, 10 mg) was added to a vigorously stirred solution of $Ni(OAc)_2 \cdot 4\ H_2O$ (0.241 mmol, 60 mg) in EtOH (3 mL) at room temperature. The resulting black suspension was vigorously stirred for 1 h

before ethylenediamine (0.968 mmol, 65 µL) was introduced. After stirring for another 30 min, the mixture was added to a flask purged with hydrogen containing compound **43**. Stirring was continued for 4 h under a hydrogen atmosphere, before the suspension was filtered through a plug of silica, which was carefully rinsed with EtOAc. The combined filtrates were evaporated and the crude product was purified by flash chromatography on silica (hexane/EtOAc, 1:1 to pure EtOAc) to provide the title compound in isomerically pure form as a white amorphous solid (10.4 mg, 37% yield over 2 steps). $[\alpha]_D^{25}$ = –56.5° (c = 0.4, $CHCl_3$); ^{1}H NMR (600 MHz, $CDCl_3$): δ = 5.87 (dd, *J* = 6.5, 1.7 Hz, 1H), 5.54 – 5.40 (m, 2H), 5.40 – 5.29 (m, 2H), 4.99 (s, 1H), 4.37 (dt, *J* = 13.8, 7.8 Hz, 1H), 3.23 (dd, *J* = 11.8, 1.9 Hz, 1H), 3.14 – 3.08 (m, 2H), 3.05 (dd, *J* = 11.8, 2.9 Hz, 1H), 2.77 (ddd, *J* = 14.4, 11.7, 2.5 Hz, 1H), 2.60 – 2.54 (m, 2H), 2.50 (dtd, *J* = 14.9, 9.2, 6.5 Hz, 1H), 2.31 – 2.21 (m, 3H), 2.20 – 2.10 (m, 5H), 2.06 – 1.98 (m, 2H), 1.95 – 1.87 (m, 2H), 1.78 (ddd, *J* = 9.1, 6.4, 2.2 Hz, 1H), 1.76 – 1.62 (m, 3H), 1.57 – 1.50 (m, 2H), 1.50 – 1.43 (m, 1H), 1.39 (ddq, *J* = 13.9, 11.5, 5.7 Hz, 1H), 1.10 (dddd, *J* = 21.5, 11.8, 8.0, 5.1 Hz, 1H); ^{13}C NMR (151 MHz, $CDCl_3$) δ = 173.1, 171.2, 145.3, 130.7, 130.4, 130.1, 128.4, 126.3, 58.5, 51.2, 47.9, 45.6, 44.2, 42.7, 39.7, 36.6, 33.8, 31.0, 28.9, 27.2, 26.7, 25.8, 25.6, 24.8, 24.2, 21.8; IR (film): $\tilde{\nu}$ = 3003, 2927, 2859, 1625, 1488, 1443, 1416, 1342, 1327, 1276, 1230, 1203, 1162, 923, 728, 665, 644 cm^{-1}. HRMS (ESI): *m/z* calcd. for $C_{26}H_{37}N_2O_2$ [M+H$^+$]: 409.28495, found: 409.28469.

(+)-Keramaphidin B ((+)-1). DIBAL-H (1 M in hexane, 0.15 mmol, 0.15 mL) was added to a solution of

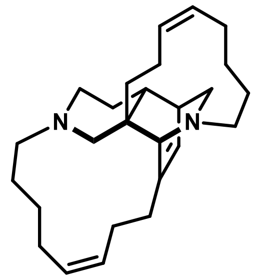

bislactam **S2** (0.015 mmol, 6.0 mg) in diethyl ether (0.15 mL). The mixture was stirred at rt for 3.5 h, before it was cooled to 0 °C and diluted with CH_2Cl_2 (1 mL). Next, sat. aq. Rochelle's salt solution (0.5 mL) was carefully added and the mixture was vigorously stirred for 1 h. The aqueous phase was extracted with CH_2Cl_2 (3 x 15 mL), the combined organic extracts were dried over $MgSO_4$ and concentrated *in vacuo*. The residue was purified by preparative HPLC (YMC Triart C18, 5 µm, 150 mm x 10 mm, methanol:20 mM NH_4HCO_3 pH 9.0 = 85:15, 4.7 mL/min, λ = 210 nm, t_R = 4.0 min) to afford the title compound as a white amorphous solid (2.1 mg, 38% yield). $[\alpha]_D^{20}$ = +27.0° (c = 0.20, MeOH); For ^{1}H- and ^{13}C-NMR Data see Tables S3-S7. IR (film): $\tilde{\nu}$ = 3005, 2920, 2851, 1486, 1460, 1340, 1317, 1299, 1275, 1220, 1207, 1174, 1130, 1103, 1048, 989, 933, 908, 819, 764, 721, 685, 666, 461 cm^{-1}. HRMS (ESI): *m/z* calcd. for $C_{26}H_{41}N_2$ [M+H$^+$]: 381.32642, found: 381.32671.

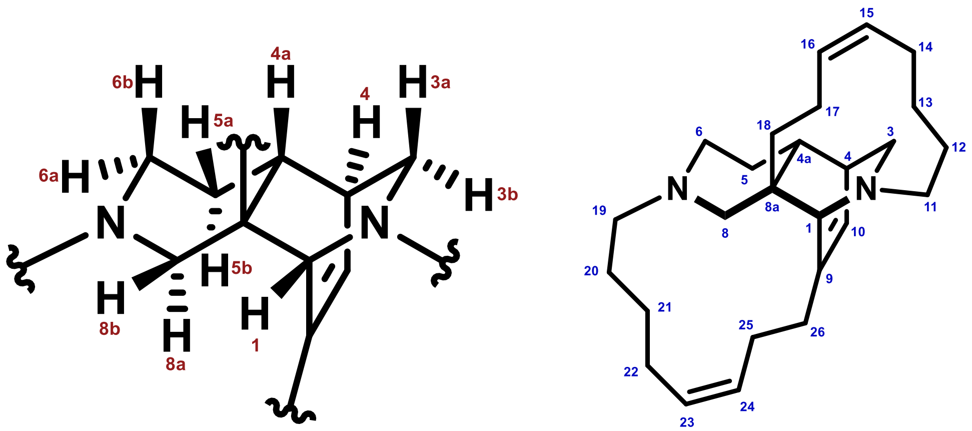

Figure S3. Numbering scheme for Keramaphidin B adopted from Kobayashi *et al*.[5]

Table S3. Comparison of ^{1}H NMR ([D$_4$]-MeOH) data of synthetic Keramaphidin B with isolated Keramaphidin B[6] (numbering scheme as shown in Figure S3)

**Position**	**Original Assignment**[5]	**^{1}H NMR Synthetic δ (ppm), *J* (Hz)**	**^{1}H NMR Isolated[5] δ (ppm), *J* (Hz)**
**1**	1	3.12, d	3.18, br s
**3a**	3	2.87 (dd, *J* = 9.1, 2.0)	2.89 (dd, *J* = 9.2, 1.9)
**3b**	3	1.67, (dd, *J* = 9.1, 2.8)	1.68 (dd, *J* = 9.2, 2.6)
**4**	4	2.25, m	2.30, m
**4a**	4a	0.90, (ddd, *J* = 12.4, 5.8, 2.4)	0.98 (ddd, *J* = 12.5, 5.5, 2.1)
**5a**	5	1.20 (tdd, *J*=13.7, 12.4, 4.1)	1.23 (qd, *J*=14.0, 4.1)
**5b**	5	1.36, (pd, *J* = 13.5, 3.2, 2.8, 2.0)	1.50, m
**6a**	6	2.68 (dt, *J* = 12.9, 4.1, 2.8)	2.88, m
**6b**	6	2.76 (td, *J* = 13.8, 13.0, 2.7)	2.97 (td, *J* = 13.5, 2.6)
**8a**	8	2.09, m	2.16 (d, *J*=11.6)
**8b**	8	2.34, m	2.70 (d, *J*=11.6)
**10**	10	5.85 (d, *J*= 6.4)	5.91 (d, *J* = 6.4)
**11**	11	2.21, m	2.21, (ddd, *J* = 12.5, 5.2, 1.2)
		2.98 (td, *J*= 12.6, 5.0)	2.99 (td, J = 12.5, 5.2)
**12**	12	1.27, m	1.27, m
		1.48, m	1.53, m
**13**	13	1.52, m	1.50, m
		1.58, m	1.61, m
**14**	14	1.55, m	1.56, m
		2.42, m	2.41, m
**15**	15	5.65, m	5.65, m
**16**	16	5.64, m	5.65, m
**17**	17	1.75, m	1.76, m
		2.35, m	2.38, m
**18**	18	1.67, m	1.75, m
		1.77, m	1.75, m
**19**	19	2.24, m	2.52, (ddd, *J* = 13.5, 7.5, 2.5)

		3.06, (ddd, J = 13.8, 8.2, 6.8)	3.24, (dt, *J* = 13.5, 7.5)
**20**	20	1.44, m	1.49, m
		1.61, m	1.73, m
**21**	21	1.35, m	1.44, m
		1.49, m	1.52, m
**22**	22	1.98, m	2.02 (br d, *J*=15.2)
		2.22, m	2.26, m
**23**	23	5.24 (tt, *J*=10.8, 2.7)	5.28 (tt, J=10.8, 2.8)
**24**	24	5.38, m	5.41, m
**25**	25	2.09, m	2.11, m
		2.36, m	2.35, m
**26**	26	2.35, m	2.38, m
		2.29, (ddd, *J* = 10.8, 6.1, 1.5)	2.31, m

Table S4. Comparison of ^{13}C NMR data ([D$_4$]-MeOH) of synthetic Keramaphidin B with those of isolated Keramaphidin B[6] (numbering scheme as shown in Figure S3); reassigned positions are shown in red

Position	Original Assignment[5]	^{13}C NMR Synthetic δ (ppm)	^{13}C NMR Isolated[5] δ (ppm)	Δδ (ppm)
**20**	20	21.5	20.9	+0.6
**17**	17	21.8	21.6	+0.2
**14**	14	23.8	23.8	0
**22**	22	26.1	26.1	0
**25**	25	26.6	26.5	+0.1
**12**	5	27.2	26.8	+0.4
**13**	12	27.5	27.1	+0.4
**21**	21	27.7	27.1	+0.6
**5**	13	28.0	27.5	+0.5
**26**	26	37.9	37.6	+0.3
**4**	4	39.1	38.8	+0.3
**18**	18	42.3	41.8	+0.5
**4a**	4a	44.9	44.1	+0.8
**8a**	8a	45.9	45.0	+0.9
**6**	6	48.5	48.8	-0.3
**8**	8	51.0	50.8	+0.2
**3**	3	54.6	54.3	+0.3
**11**	11	55.2	55.1	+0.1
**19**	19	57.1	56.9	+0.2
**1**	1	65.3	64.6	+0.7
**10**	10	124.3	125.0	-0.7
**15**	16	131.5	131.0	+0.5
**16**	23	132.4	132.6	-0.2
**23**	15	132.6	132.8	-0.2
**24**	24	133.3	133.4	-0.1
**9**	9	143.0	142.8	+0.2

Table S5. Comparison of the ^{13}C NMR ([D_4]-MeOH) data of synthetic Keramaphidin B with those of a sample of Keramaphidin B prepared by Baldwin *et al.*, [7] which had been doped with authentic material provided by Kobayashi et al.[5] (numbering scheme as shown in Figure S3)

Position	^{13}C NMR Synthetic δ (ppm)	^{13}C NMR (literature) δ (ppm)	Δδ (ppm)
20	21.5	21.3	+0.2
17	21.8	21.7	+0.1
14	23.8	23.8	0
22	26.1	26.1	0
25	26.6	26.5	+0.1
12	27.2	27.1	+0.1
13	27.5	27.5	0
21	27.7	-	-
5	28.0	-	-
26	37.9	37.8	+0.1
4	39.1	39.0	+0.1
18	42.3	42.1	+0.2
4a	44.9	44.7	+0.2
8a	45.9	-	-
6	48.5	48.8	-0.3
8	51.0	50.9	+0.1
3	54.6	54.5	+0.1
11	55.2	55.2	0
19	57.1	57.0	+0.1
1	65.3	65.0	+0.3
10	124.3	124.5	-0.2
15	131.5	131.3	+0.2
16	132.4	132.6	-0.2
23	132.6	132.6	0
24	133.3	133.3	0
9	143.0	142.9	+0.1

Table S6. Comparison of ^{1}H NMR ($CDCl_3$) data of synthetic Keramaphidin B with those of the isolated sample fo Keramaphidin B[5] (numbering scheme as shown in Figure S3); reassigned positions are shown in red

Position	Original Assignment[6]	^{1}H NMR Synthetic δ (ppm), *J* (Hz)	^{1}H NMR Isolated[6] δ (ppm), *J* (Hz)
**1**	1	3.01, s	3.01, s
**3a**	3	2.85 (dd, *J* = 9.1, 2.1)	2.86 (dd, *J* = 8.5, 1.5)
**3b**	3	1.64 (dd, *J* = 9.1, 2.7)	1.64 (dd, *J* = 9.0, 2.3)
**4**	4	2.20, m	2.22, m
**4a**	4a	0.91, m	0.93 (ddd, *J* = 11.6, 5.6, 1.9)
**5a**	5	1.16 (qd, *J* = 13.0, 4.6)	1.17 (ddd, *J* =13.0, 8.7, 4.4)
**5b**	5	1.30, m	1.36, m
**6a**	6	2.62 (d, *J*= 12.2)	2.63 (dt, *J* = 12.3, 3.6)
**6b**	6	2.67 (t, *J* = 12.4)	2.75, m
**8a**	8	2.07, m	2.08 (d, *J* = 10.7)
**8b**	8	2.12, m	2.23 (d, *J* = 12.3)
**10**	10	5.79 (d, *J* = 6.5)	5.81
**11**	11	2.22, m	2.23, m
	3	2.88 (dd, *J* = 12.6, 5.2)	2.91 (dd, *J* = 20.7, 9.7)
**12**	12	1.25, m	1.24, m
		1.45, m	1.45, m
**13**	13	1.49, m	1.46, m
		1.58, m	1.58, m
**14**	14	1.58, m	1.57, m
		2.34, m	2.35, m
**15**	16	5.70 (td, *J* = 10.4, 9.7, 6.3)	5.69 (ddd, *J* = 13.6, 10.1, 6.3)
**16**	15	5.64 (td, *J* = 10.4, 5.1)	5.64 (ddd, *J* = 13.6, 10.1, 5.2)
**17**	17	1.73, br s	1.78, m
		2.28, m	2.27, m
**18**	18	1.62, m	1.61, m
		1.86 (td, *J*= 12.1, 7.6)	1.88 (dt, *J* = 12.3, 7.6)
**19**	19	2.16, m	2.24, m
		3.05, br s	3.07, m
**20**	20	1.30, m	1.34, m
		1.54, m	1.55, m
**21**	21	1.29, m	1.32, m
		1.46, m	1.48, m
**22**	22	1.95 (d, *J* = 15.1)	1.96 (br d, *J*= 15.2)
		2.16, m	2.14, m
**23**	23	5.23 (tt, *J* = 10.8, 3.1)	5.24 (br d, *J* = 10.8)
**24**	24	5.35, m	5.36 (br d, *J* = 10.8)
**25**	25	2.10, m	2.12, m
		2.28, m	2.29, m
**26**	26	2.23, m	2.25, m
		2.32, m	2.33, m

Table S7. Comparison of ^{13}C NMR ($CDCl_3$) data of synthetic Keramaphidin B with those of isolated Keramaphidin B[5] (numbering scheme as shown in Figure S3)

Position	Original Assignment[6]	^{13}C NMR Synthetic δ (ppm)	^{13}C NMR Isolated[6] δ (ppm)	Δδ (ppm)
**17**	17	21.0	21.0	0
**20**	20	21.2	21.1	+0.1
**14**	14	22.9	22.9	0
**22**	22	24.9	25.0	-0.1
**25**	25	25.5	25.6	-0.1
**12**	12	26.1	26.1	0
**13**	13	26.4	25.6	+0.8
**21**	21	27.3	27.2	+0.1
**5**	5	27.8	27.6	+0.2
**26**	26	37.0	37.0	0
**4**	4	37.9	38.0	-0.1
**18**	18	41.6	41.6	0
**4a**	4a	43.4	43.3	+0.1
**8a**	8a	45.2	45.1	+0.1
**6**	6	47.4	47.4	0
**8**	8	50.7	50.8	-0.1
**3**	3	53.6	53.6	0
**11**	11	54.0	54.1	-0.1
**19**	19	56.2	56.2	0
**1**	1	64.3	64.3	0
**10**	10	122.5	122.6	-0.1
**15**	16	130.9	130.9	0
**16**	15	131.2	131.2	0
**23**	23	131.5	131.5	0
**24**	24	132.0	132.0	0
**9**	9	141.7	141.8	-0.1

Nominal Njaoamine I

The Quinoline Building Block

***N*-(3-(2-Aminophenyl)-3-oxopropyl)-2,2,2-trifluoroacetamide (45).** Trifluoroacetic acid anhydride (9.5 mL, 68.3 mmol) was slowly added to a solution of tryptamine (8.0 g, 50.0 mmol) in CH_2Cl_2 (300 mL) at 0 °C. After stirring for 2h at this temperature, H_2O (50 mL) was added to terminate the reaction. The mixture was extracted with CH_2Cl_2 (3 x 500 mL), the combined extracts were dried over anhydrous Na_2SO_4, the solvent was removed under vacuum, and the crude product was used for next step without further purification.

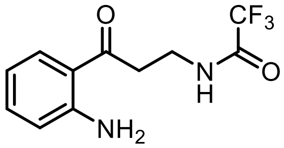

The crude product was dissolved in MeOH (800 mL) and the solution was added dropwise to a solution of $NaIO_4$ (54.8 g, 256 mmol) in H_2O (800 mL) at 0 °C. The ice bath was removed and stirring continued at ambient temperature for 24 h. The mixture was poured into H_2O (500 mL), the aqueous layer was extracted with CH_2Cl_2 (3 × 800 mL), and the combined organic phases were washed with brine and dried over anhydrous Na_2SO_4. After filtration, the solvent was evaporated to provide the crude product which was directly used for the next step.

conc. HCl (6.4 mL, 80.0 mmol) was added dropwise to a solution of this crude material in MeOH (640 mL). The mixture was stirred at reflux temperature for 1 hour before it was cooled to room temperature and aq. K_2CO_3 (1 M, 76 mmol) was added until a pH ≈ 6 was reached. The yellow residue was poured into H_2O (80 mL), the aqueous phase was extracted with CH_2Cl_2 (3 × 500 mL), the combined extracts were dried over anhydrous Na_2SO_4 and filtered. After removing the solvent, the residue was purified by flash chromatography on silica gel (CH_2Cl_2/*tert*-butyl methyl ether, 20:1) to afford the title compound as a yellow solid (13.1 g, 81 % over 3 steps). ^{1}H NMR (400 MHz, $CDCl_3$): δ = 7.67–7.63 (m, 1H), 7.30 (ddd, *J* = 8.6, 7.1, 1.5 Hz, 1H), 7.20 (s, 1H), 6.66 (td, *J* = 8.2, 1.0 Hz, 2H), 6.29 (s, 2H), 3.80–3.73 (m, 2H), 3.25 (dd, *J* = 6.0, 5.0 Hz, 2H) ppm; ^{13}C NMR (101 MHz, $CDCl_3$): δ = 200.5, 150.5, 135.1, 130.9, 117.5, 117.3, 117.1, 116.1, 114.4, 37.6, 34.9 ppm; ^{19}F NMR (282 MHz, $CDCl_3$) δ = –76.1 ppm; IR (film) $\tilde{\nu}$ = 3468, 3348, 1708, 1616, 1550, 1452, 1204, 1159, 971, 750 cm^{-1}; MS (EI)*: m/z* (%): 120 (100), 260 (32.9); HRMS (ESI): *m/z:* calcd. for $C_{11}H_{11}N_2O_3F$ [M^+]: 260.07671, found: 260.07733.

2,2,2-Trifluoro-N-(2-(2-hydroxy-3-propionylquinolin-4-yl)ethyl)acetamide (47). Compound **46** (5.50 g, 27.5 mmol)[8] was added to a solution of compound **45** (4.78 g, 18.4 mmol) in toluene (60 mL) at ambient temperature. The resulting mixture was stirred at reflux temperature for 2 h before it was cooled to ambient temperature and directly loaded on silica filled into a flash column. After a contact time of 24

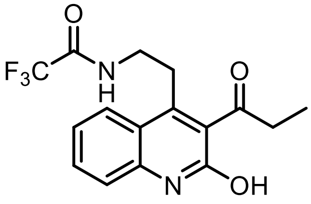

h, the product was eluted (hexanes/acetone, 3:1 to 0:1) to provide the tilte compound as a yellow solid (5.89 g, 94 %). ^{1}H NMR (400 MHz, [D$_4$]-MeOH): δ = 8.07 (dd, *J* = 8.2, 1.3 Hz, 1H), 7.61 (ddd, *J* = 8.4, 7.1, 1.3 Hz, 1H), 7.42– 7.31 (m, 2H), 3.58 (dd, *J* = 8.5, 6.5 Hz, 2H), 3.07-3.02 (m, 2H), 2.92 (q, *J* = 7.2 Hz, 2H), 1.18 (t, *J* = 7.2 Hz, 3H) ppm; ^{13}C NMR (101 MHz, [D$_4$]-MeOH): δ = 208.1, 162.1, 146.9, 139.8, 134.8, 132.7 126.6, 124.4, 120.3, 119.0, 117.4, 116.1, 40.9, 37.9, 29.8, 7.9 ppm; ^{19}F NMR (282 MHz, MeOD): δ = –77.4 ppm; IR (film) $\tilde{\nu}$ = 3307, 2942, 2883, 1701, 1652, 1563, 1187, 1152, 757 cm^{-1}; MS (EI)*: m/z* (%): 212 (100), 340 (12); HRMS (ESI): *m/z:* calcd. for $C_{16}H_{15}F_3N_2O_3$ [*M*$^+$]: 340.10293, found: 340.10283.

2,2,2-Trifluoro-N-(2-(2-hydroxy-3-propionylquinolin-4-yl)ethyl)acetamide (48). Tf_2O (2.3 mL, 13.7 mmol) was added to a solution of compound **47** (3.18 g, 9.30 mmol) in pyridine (50.0 mL) at 0 °C. After 10 min, the cooling bath was removed and the mixture stirred at ambient temperature for 12 h. The mixture was poured into H_2SO_4 (2 M, 400 mL) at 0 °C, the aqueous phase was extracted with EtOAc (3 x 500 mL), and the combined organic layers were washed with brine and dried over $MgSO_4$. After filtration and evaporation of the solvent, the crude product was purified by flash chromatography on silica gel (CH_2Cl_2/*tert*-butyl methyl ether, 40:1) to provide the title compound as a yellow solid material (3.88 g, 88 %). ^{1}H NMR (400 MHz, $CDCl_3$): δ = 8.21 (dd, *J* = 8.6, 0.8 Hz, 1H), 8.07 (ddd, *J* = 8.4, 1.4, 0.6 Hz, 1H), 7.87 (ddd, *J* = 8.4, 7.0, 1.4 Hz, 1H), 7.79–7.66 (m, 2H), 3.74 (td, *J* = 6.9, 5.1 Hz, 2H), 3.28 (t, *J* = 7.0 Hz, 2H), 2.95 (q, *J* = 7.3 Hz, 2H), 1.27 (t, *J* = 7.2 Hz, 3H) ppm; ^{13}C NMR (101 MHz, $CDCl_3$): δ = 204.7, 158.5, 158.1, 157.7, 157.4, 149.3, 147.6, 145.5, 132.3, 129.8, 128.9, 126.1, 125.8, 124.3, 120.1, 117.0, 116.9, 114.2, 40.2, 38.3, 28.5, 7.9 ppm; ^{19}F NMR (282 MHz, $CDCl_3$) δ = –72.4, –76.0 ppm; IR (film) $\tilde{\nu}$ = 3342, 2955, 1703, 1563, 1420, 1178, 1121, 997, 760 cm^{-1}; MS (ESI)*: m/z*: 473 [*M*+H$^+$], 495 [*M*+Na$^+$]; HRMS (ESI): *m/z:* calcd. for $C_{17}H_{14}F_6N_2O_5SNa$ [*M*+Na$^+$]: 495.04199, found: 495.04193.

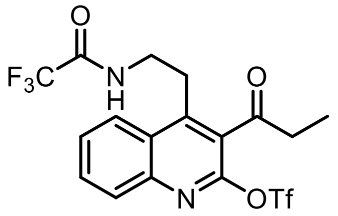

***N*-(2-(2-(4-((*tert*-butyldimethylsilyl)oxy)butyl)-3-propionylquinolin-4-yl)ethyl)-2,2,2-trifluoroacet-amide (50).** Neat (but-3-en-1-yloxy)(tert-butyl)dimethylsilane (8.0 mL, 29.1 mmol)[9] was added to a solution of 9-H-9-BBN (0.5 M in THF, 31.6 mL, 15.8 mmol) at ambient temperature. After stirring at this temperature for 12 h, the solution was warmed to 40 °C and stirring was continued for another 6 h before the mixture was cooled to ambient temperature. MeONa (821 mg, 15.2 mmol) was added and the resulting mixture was stirred for 1 h at ambient temperature. $Pd(PPh_3)_4$ (475 mg, 0.411 mmol) and triflate **48** (3.88 g, 8.21 mmol) were successively added to this solution. The resulting mixture was stirred at 80 °C for 15 h before it was

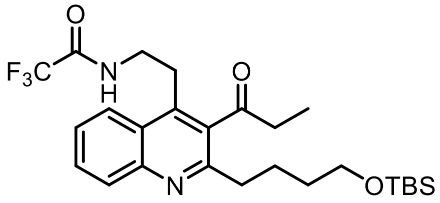

cooled to ambient temperature. The mixture was diluted with *t*ert-butyl methyl ether (3 x 100 mL) and washed with brine, the organic phase was dried with Na_2SO_4, the solvent was evaporated under vacuum, and the crude product was purified by flash chromatography on silica gel (hexanes/EtOAc*,* 10:1 to 4 :1) to afford the title compound as a yellow oil (3.08 g, 73 %). ^{1}H NMR (400 MHz, $CDCl_3$): δ = 8.08 (ddd, *J* = 8.5, 1.3, 0.6 Hz, 1H), 8.04 (ddd, *J* = 8.5, 1.4, 0.6 Hz, 1H), 7.75 (ddd, *J* = 8.4, 6.9, 1.3 Hz, 1H), 7.69 (s, 1H), 7.60 (ddd, *J* = 8.3, 6.9, 1.3 Hz, 1H), 3.68 (td, *J* = 6.6, 4.8 Hz, 2H), 3.63 (t, *J* = 6.4 Hz, 2H), 3.16 (s, 2H), 2.86–2.79 (m, 4H), 1.94–1.81 (m, 2H), 1.63–1.53 (m, 2H), 1.27 (t, *J* = 7.2 Hz, 3H), 0.88 (s, 9H), 0.03 (s, 6H) ppm; ^{13}C NMR (101 MHz, $CDCl_3$): δ = 211.4, 157.9, 157.5, 156.5, 147.9, 138.9, 135.5, 130.2, 130.0, 127.2, 124.4, 123.3, 114.2, 62.7, 40.1, 39.2, 37.0, 32.6, 27.8, 26.0, 25.9, 18.3, 8.0, -5.4ppm; ^{19}F NMR (282 MHz, $CDCl_3$): δ = –75.9 ppm; IR (film) $\tilde{\nu}$ = 3309, 2931, 2858, 1703, 1208, 1160, 835, 762 cm^{-1}; MS (ESI)*: m/z*: 511 [*M*+H$^+$], 533 [*M*+Na$^+$]; HRMS (ESI): *m/z:* calcd. for $C_{26}H_{37}N_2O_3F_3SiNa$ [*M*+Na$^+$]: 533.24178, found: 533.24155.

***tert*-Butyl (2-(2-(4-hydroxybutyl)-3-(prop-1-yn-1-yl)quinolin-4-yl)ethyl)carbamate (S3).** KHMDS (1.0 M in THF, 31.7 mL, 31.7 mmol) was added to a solution of compound **50** (3.08 g, 6.03 mmol) and $PhNTf_2$ (3.39 g, 9.49 mmol) in THF (40 mL) at –78 °C. After stirring at this temperature for 1 h, the reaction was quenched with sat. aq. $NaHCO_3$ (10 mL). The aqueous layer was extracted with EtOAc (3 x 100 mL), and the combined extracts were washed with brine and dried with anhydrous Na_2SO_4. After filtration and evaporation of the solvent, the crude material was dissolved in CH_3CN (30 mL). DMAP (3.10 g, 25.4 mmol) and Boc_2O (5.34 g, 24.5mmol) were successively added at 0 °C., the cooling bath was removed after 5 min, and the mixture stirred at ambient temperature for 2 h before the reaction was quenched with sat. aq. NH_4Cl (30 mL). The resulting mixture was stirred for 5 h before it was extracted with *t*ert-butyl methyl ether (3 x 100 mL). The combined organic layers were washed with brine, dried with anhydrous $MgSO_4$, filtered and the solvent was evaporated. The residue was purified by flash chromatography on silica gel (hexanes/*tert*-butyl methyl ether, 8:1 to 2:1).

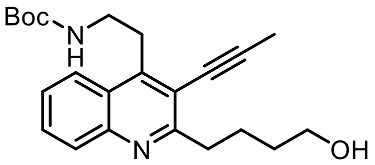

TBAF (1.0 M in THF, 24.0 mL, 24.0 mmol) was added to a solution of the product thus obtained in THF (10 mL) at 0 °C. The mixture was then stirred at ambient temperature for 1 h before the reaction was quenched with sat. aq. NH_4Cl (50 mL). The aqueous layer was extracted with EtOAc (4 x 100 mL), the combined organic phases were washed with brine, dried with anhydrous Na_2SO_4 and filtered. After evaporation of the solvent, the residue was purified by flash chromatography on silica (hexanes/acetone, 8:1 to 1:1) to afford the title compound as a yellow oil (1.66 g, 72 %). ^{1}H NMR (400 MHz, $CDCl_3$): δ = 8.06 (d, *J* = 8.4 Hz, 1H), 7.99 (dd, *J* = 8.4, 1.3 Hz, 1H), 7.64 (ddd, *J* = 8.4, 6.9, 1.4 Hz, 1H), 7.51 (ddd, *J* = 8.3, 6.9, 1.3 Hz, 1H), 4.69

(s, 1H), 3.69 (t, J = 6.3 Hz, 2H), 3.48 (d, J = 2.8 Hz, 5H), 3.20 (t, J = 7.3 Hz, 2H), 2.87 (s, 1H), 2.21 (s, 3H), 2.08 – 1.89 (m, 2H), 1.83 – 1.55 (m, 1H), 1.42 (s, 9H) ppm; ^{13}C NMR (101 MHz, $CDCl_3$): δ = 162.6, 155.9, 147.4, 145.7, 129.4, 129.1, 126.4, 125.7, 123.8, 117.7, 95.9, 79.3, 75.8, 62.3, 40.4, 36.8, 32.3, 30.9, 28.4, 24.1, 4.8 ppm; IR (film) $\tilde{\nu}$ = 3322, 2933, 1691, 1498, 1365, 1252, 1170, 1072, 761 cm^{-1}; MS (ESI): *m/z*: 383 [M+H$^+$], 405 [M+Na$^+$]; HRMS (ESI): *m/z:* calcd. for $C_{23}H_{31}N_2O_3$ [M+H$^+$]: 383.23292, found: 383.23288.

***tert*-Butyl (2-(2-(4-oxobutyl)-3-(prop-1-yn-1-yl)quinolin-4-yl)ethyl)carbamate (52).** Sulfur trioxide pyridine complex (750 mg, 4.71 mmol) was added to a solution of anhydrous Et_3N (1.3 mL, 9.32 mmol), alcohol **S3** (604 mg, 40.8 mg) and DMSO (0.56 mL, 7.88 mmol) in CH_2Cl_2 (6.3 mL) at 0 °C. After 10 min, the cooling bath was removed and stirring was continued at ambient temperature for 3 h before sat. aq. $NaHCO_3$ (2.0 mL) was added. The aqueous layer was extracted with EtOAc (3 x 30 mL), and the combined organic phases were dried over Na_2SO_4, filtered, and concentrated under vacuum. The residue was purified by flash chromatography on silica gel (hexanes/acetone, 6:1 to 3:1) to afford the title compound as a yellow oil (461 mg, 77 %). ^{1}H NMR (400 MHz, $CDCl_3$): δ = 9.79 (t, J = 1.7 Hz, 1H), 8.05 (d, J = 8.4 Hz, 1H), 7.98 (dd, J = 8.4, 1.3 Hz, 1H), 7.64 (ddd, J = 8.3, 6.8, 1.4 Hz, 1H), 7.51 (ddd, J = 8.3, 6.9, 1.3 Hz, 1H), 4.73 (s, 1H), 3.47 (d, J = 2.8 Hz, 4H), 3.25–3.03 (m, 2H), 2.57 (td, J = 7.2, 1.8 Hz, 2H), 2.32–2.15 (m, 5H), 1.42 (s, 9H) ppm; ^{13}C NMR (101 MHz, $CDCl_3$): δ = 202.5, 161.7, 155.9, 147.3, 146.0, 129.4, 129.3, 126.4, 125.7, 123.8, 117.7, 96.0, 75.7, 43.4, 40.3, 36.7, 30.8, 28.4, 21.1, 4.7 ppm; IR (film) $\tilde{\nu}$ = 3368, 2977, 2936, 1713, 1498, 1367, 1250, 872, 764 cm^{-1}; MS (ESI): *m/z*: 381 [M+H$^+$]; HRMS (ESI): *m/z:* calcd. for $C_{23}H_{29}N_2O_3$ [M+H$^+$]: 381.21727, found: 381.21717.

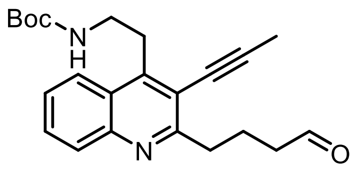

The Failed First Foray

3-Allyl 1-(*tert*-butyl) (5*R*)-5-((*tert*-butyldimethylsilyl)oxy)-2-oxo-3-(pent-3-yn-1-yl)piperidine-1,3-dicarboxylate (S4). LiHMDS (1 M in THF, 26.7 ml, 26.7 mmol) was slowly added to a solution of *ent*-**15** (3.83 g, 11.6 mmol) in anhydrous THF (58 ml) at –78 °C. The mixture was stirred for 1 h before allyl chloroformate (1.3 ml, 12.2 mmol) was added. After 30 min, the reaction was quenched with sat. aq. NH_4Cl (10 mL). The aqueous layer was extracted with EtOAc (3 x 200 mL), and the combined organic phases were washed with brine (20 mL), dried over Na_2SO_4 and filtered. After evaporation of the solvent, the residue was purified by flash chromatography on silica (hexanes/EtOAc, 5 :1 to 3:1) to provide a yellow oil.

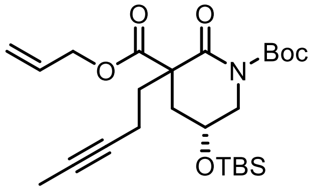

Cs_2CO_3 (7.66 g , 23.5 mmol) was added to a solution of this compound and 5-iodopent-2-yne (4.5 g, 23.2 mmol)[1] in DMF (20.0 mL) at ambient temperature. The mixture was stirred for 12 h before the reaction was quenched with sat. aq. NH_4Cl (10 mL). The resulting solution was extracted with EtOAc (3 x 50 mL). The combined extracts were dried over anhydrous Na_2SO_4, filtered, and concentrated under reduced pressure. The residue was purified by flash chromatography on silica (hexane/EtOAc, 1:10) to afford the title product as a white solid material (5.17 g, 93 %). $[\alpha]_D^{20}$ = –10.2 (c = 1.8, $CHCl_3$); ^1H NMR (400 MHz, $CDCl_3$): δ = 5.99–5.73 (m, 1H), 5.42–5.27 (m, 1H), 5.25–5.16 (m, 1H), 4.68–4.52 (m, 2H), 4.20–4.04 (m, 1H), 3.83–3.69 (m, 1H), 3.51–3.37 (m, 1H), 2.68–2.41 (m, 1H), 2.31–1.96 (m, 5H), 1.80–1.66 (m, 3H), 1.49 (s, 9H), 0.89–0.79 (m, 9H), 0.09 – –0.03 (m, 6H) ppm; ^{13}C NMR (101 MHz, $CDCl_3$): δ = 171.0, 170.9, 169.4, 168.9, 152.7, 152.5, 131.3, 131.1, 119.0, 118.4, 83.1, 83.1, 78.2, 78.1, 76.2, 76.2, 66.2, 66.0, 63.9, 63.8, 55.2, 54.4, 51.1, 50.9, 38.7, 38.6, 35.8, 35.4, 27.9, 25.6, 25.6, 18.0, 17.9, 14.7, 14.4, 3.4, 3.4, –4.8, –4.9, –5.04, –5.00 ppm; IR (film) $\tilde{\nu}$ = 2926, 2856, 1717, 1376, 1300, 1254, 1147, 1092, 836, 777 cm^{-1}; MS (ESI): *m/z*: 502 [*M*+Na^+]; HRMS (ESI): *m/z:* calcd. for $C_{25}H_{41}NO_6SiNa$ [*M*+Na^+]: 502.2595, found: 502.2597.

***tert*-Butyl (*R*)-3-((*tert*-butyldimethylsilyl)oxy)-6-oxo-5-(pent-3-yn-1-yl)-3,6-dihydropyridine-1(2*H*)-carboxylate (53).** $Pd_2(dba)_3$•$CHCl_3$ (974 mg, 0.941 mmol) was added to a solution of compound **S4** (9.03 g, 18.8 mmol) in CH_3CN (76 mL) at ambient temperature. The mixture was stirred at 80 °C for 30 min before it was cooled to ambient temperature and filtered through a pad of Celite. The filtrate was evaporated and the residue was purified by chromatography on silica (hexanes/CH_2Cl_2, 1:1 to 1:4 to remove the dba, then the elutant was changed to hexanes/*tert*-butyl methyl ether, 4:1) to afford the title compound as a colorless oil (5.84 g, 79 %). $[\alpha]_D^{20}$ = 56.3 (c = 0.9, $CHCl_3$); ^1H NMR (400 MHz, $CDCl_3$) δ = 6.51–6.48 (m, 1H), 4.50–4.44 (m, 1H), 3.91 (ddd, *J* = 12.8, 4.9, 1.2 Hz, 1H), 3.64 (dd, *J* = 12.8, 8.2 Hz, 1H), 2.57–2.36 (m, 2H), 2.32 (ddddd, *J* = 6.3, 5.3, 3.8, 2.6, 1.0 Hz, 2H), 1.75 (t, *J* = 2.5 Hz, 3H), 1.54 (s, 9H), 0.89 (s, 9H), 0.11 (s, 6H) ppm; ^{13}C NMR (101 MHz, $CDCl_3$) δ = 163.6, 152.7, 142.1, 134.0, 83.1, 78.3, 63.9, 50.7, 29.9, 28.1, 25.7, 18.1, 17.9, 3.4, –4.7, –4.7 ppm; IR (film) $\tilde{\nu}$ = 2930, 2857, 1715, 1368, 1301, 1255, 1093, 837, 778 cm^{-1}; MS (ESI): *m/z*: 416 [*M*+Na^+]; HRMS (ESI): *m/z:* calcd. for $C_{21}H_{35}NO_4SiNa$ [*M*+Na^+]: 416.22276, found: 416.22272.

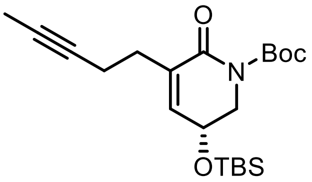

Allyl 1-benzyl-4-oxo-3-(pent-4-en-1-yl)piperidine-3-carboxylate (S5). Cs_2CO_3 (3.83 g, 11.8 mmol) was added to a solution of compound **19b** (1.6 g, 5.85 mmol), 1-bromo-4-pentene (1.4 g, 11.8 mmol) and NaI (1.78 g, 11.9 mmol) in DMF (11 mL) at ambient temperature. The mixture was stirred for 12 h before the reaction was quenched with sat. aq. NH_4Cl (10 mL). The resulting solution was extracted with EtOAc (3 x 50 mL). The combined organic extracts were dried over anhydrous Na_2SO_4, filtered, and concentrated under reduced pressure. The crude product was purified by flash chromatography on silica (EtOAc/hexane, 1:5) to afford the title compound as a colorless oil (1.91 g, 96%).^{1}H NMR (400 MHz, $CDCl_3$): δ = 7.34 – 7.04 (m, 5H), 5.84 (ddt, *J* = 17.2, 10.4, 5.8 Hz, 1H), 5.70 (ddt, *J* = 16.9, 10.2, 6.6 Hz, 1H), 5.28 (dq, *J* = 17.2, 1.5 Hz, 1H), 5.20 (dq, *J* = 10.4, 1.3 Hz, 1H), 4.97–4.85 (m, 2H), 4.64 (ddt, *J* = 13.2, 5.8, 1.4 Hz, 1H), 4.56 (ddt, *J* = 13.1, 5.8, 1.4 Hz, 1H), 3.59–3.48 (m, 2H), 3.36 (dd, *J* = 11.5, 2.6 Hz, 1H), 2.98–2.91 (m, 1H), 2.88–2.72 (m, 1H), 2.41–2.27 (m, 2H), 2.20 (d, *J* = 11.5 Hz, 1H), 1.97 (ddtt, *J* = 6.6, 5.2, 4.0, 2.9 Hz, 2H), 1.79–1.70 (m, 1H), 1.54 – 1.36 (m, 2H), 1.19 – 1.03 (m, 1H) ppm; ^{13}C NMR (101 MHz, $CDCl_3$): δ = 206.1, 171.3, 138.1, 137.8, 131.6, 128.7, 128.2, 127.3, 118.7, 114.8, 65.7, 61.8, 61.2, 61.2, 53.5, 40.5, 34.0, 31.6, 23.7 ppm; IR (film) ν = 2925, 2806, 1454, 1315, 1224, 1190, 1121, 912, 738 cm^{-1}; MS (ESI)*: m/z*: 342 [*M*+H$^+$]; 364 [*M*+Na$^+$]; HRMS (ESI): *m/z:* calcd. for $C_{21}H_{28}NO_3$ [*M*+H$^+$]: 342.20637, found: 342.20614.

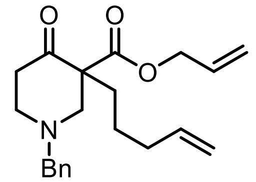

3-Allyl 1-methyl 4-oxo-3-(pent-4-en-1-yl)piperidine-1,3-dicarboxylate (S6). Methyl chloroformate (12 mL, 155 mmol) was added to a solution of compound **S5** (10.0 g, 29.3 mmol) in toluene at ambient temperature. The mixture was stirred at 100 ^{o}C for 12 h before it was adsorbed on silica and directly loaded on top of a silica column, eluting the product with hexanes/EtOAc (5:1 to 1:1). The title compound was obtained as a colorless oil (9.02 g, quant.). ^{1}H NMR (400 MHz, $CDCl_3$): δ = 5.86 (ddt, *J* = 17.2, 10.4, 5.8 Hz, 1H), 5.74 (ddt, *J* = 16.9, 10.2, 6.7 Hz, 1H), 5.31 (dq, *J* = 17.2, 1.5 Hz, 1H), 5.24 (dq, *J* = 10.4, 1.3 Hz, 1H), 5.02–4.91 (m, 2H), 4.62–4.57 (m, 2H), 4.52 (dd, *J* = 13.8, 2.0 Hz, 1H), 4.11 (s, 1H), 3.73 (s, 3H), 3.39 (s, 1H), 3.19 (d, *J* = 13.7 Hz, 1H), 2.66 (ddd, *J* = 14.7, 9.8, 6.3 Hz, 1H), 2.46 (dt, *J* = 14.6, 4.8 Hz, 1H), 2.08–1.97 (m, 2H), 1.90–1.81 (m, 1H), 1.65–1.53 (m, 1H), 1.46–1.30 (m, 2H) ppm; ^{13}C NMR (101 MHz, $CDCl_3$): δ = 204.2, 169.8, 155.6, 137.8, 131.2, 119.2, 115.1, 66.1, 61.2, 53.0, 50.1, 43.7, 39.6, 33.8, 31.1, 23.4 ppm; IR (film) ν = 2955, 1450, 1414, 1239, 1193, 1137, 996 cm^{-1}; MS (ESI)*: m/z*: 310 [*M*+H$^+$]; 332 [*M*+Na$^+$]; HRMS (ESI): *m/z:* calcd. for $C_{16}H_{23}NO_5Na$ [*M*+H$^+$]: 332.14684, found: 332.14658.

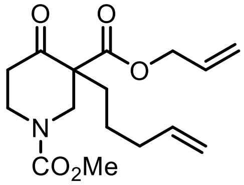

Methyl 4-oxo-5-(pent-4-en-1-yl)-3,4-dihydropyridine-1(2*H*)-carboxylate (54). $Pd_2(dba)_3$ (970 mg, 1.06 mmol) was added to a solution of compound **S6** (6.74 g, 21.8 mmol) in CH_3CN (88 mL) at ambient temperature. The mixture was stirred at 80 °C for 30 min before it was cooled to ambient temperature and filtered through a pad of Celite, which was rinsed with *tert*-butyl methyl ether (100 mL). The combined filtrates were evaporated and the crude product purified by flash chromatography on silica gel (hexanes/*tert*-butyl methyl ether, 4:1 to 1:1) to afford the title compound as a colorless oil (4.43 g, 91%). ^{1}H NMR (400 MHz, $CDCl_3$): δ = 7.65 (s, 1H), 5.79 (ddt, *J* = 16.9, 10.2, 6.6 Hz, 1H), 5.11–4.85 (m, 2H), 3.97 (t, *J* = 7.3 Hz, 2H), 3.85 (s, 3H), 2.76–2.36 (m, 2H), 2.29 – 2.12 (m, 2H), 2.05 (tdt, *J* = 8.0, 6.6, 1.5 Hz, 2H), 1.58–1.37 (m, 2H) ppm; ^{13}C NMR (101 MHz, $CDCl_3$): δ = 193.1, 140.5, 140.0, 138.4, 118.7, 114.7, 53.9, 42.6, 35.9, 33.3, 28.3, 26.8 ppm; IR (film) ν = 2927, 1723, 1665, 1618, 1442, 1371, 1264, 1207, 1007, 912 cm^{-1}; MS (ESI)*: m/z*: 224 [*M*+H$^+$], 246 [*M*+Na$^+$]; HRMS (ESI): *m/z:* calcd. for $C_{12}H_{17}NO_3Na$ [*M*+Na$^+$]: 246.11006, found: 246.10998.

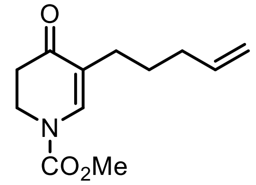

Compound 55. A solution of Bn_2NLi (0.5 M in THF, 3.94 mmol) was added to a solution of compound **54** (1060 mg, 4.75 mmol) in THF (3.0 mL) at –50 °C. The mixture was stirred for 30 min before DMPU (0.48 mL, 3.98 mmol) and a solution of compound **53** (1.06 g, 4.75 mmol) in THF (4.0 mL) were successively added. The resulting mixture was gradually warmed to 20 °C over the course of 4h and stirring continued for 24 h before DMAP (1.65 g,13.5 mmol) and Boc_2O (2.93 g, 13.4 mmol) were added. The resulting solution was stirred for 1h before the reaction was quenched with sat. aq. $NaHCO_3$ (5 mL). The mixture was extracted with EtOAc (3 x 30 mL), and the combined organic extracts were dried over anhydrous Na_2SO_4, filtered, and concentrated under reduced pressure. The residue was purified by flash chromatography on silica (hexanes/EtOAc, 10:1 to 6:1) to afford the product **55** which was used in the next step without further characterization

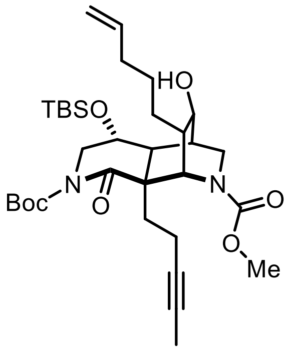

$NaBH_4$ (537 mg, 14.2 mmol) was added in portions to a solution of product **55** in MeOH (31 mL) at 0 °C. The mixture was stirred at this temperature for 30 min before the reaction was quenched with sat. aq. NH_4Cl (10 mL). The resulting mixture was extracted with EtOAc (3 x 50 mL), the combined organic phases were washed with brine (10 mL), dried over $MgSO_4$, filtered, and concentrated. The crude material was purified by flash chromatography on silica gel (hexanes/EtOAc, 4:1 to 2:1) to afford the title compound as a white solid (1.22 g, 42%). $[\alpha]_D^{20}$ = 81.8 (c = 0.4, $CHCl_3$); ^{1}H NMR (400 MHz, $CDCl_3$): δ = 5.78 (ddq, *J* = 16.9, 10.1, 6.7 Hz, 1H), 5.02–4.86 (m, 2H), 4.52–4.41 (m, 1H), 4.32–4.16 (m, 1H), 4.14–4.08 (m, 1H), 3.74–3.67

(m, 3H), 3.57–3.47 (m, 1H), 3.32 (ddd, *J* = 25.1, 11.4, 3.0 Hz, 1H), 3.21–3.04 (m, 2H), 2.34–2.14 (m, 2H), 2.09–1.93 (m, 3H), 1.81–1.76 (m, 2H), 1.73 (q, *J* = 2.4 Hz, 3H), 1.71–1.66 (m, 1H), 1.66–1.59 (m, 2H), 1.54–1.49 (m, 9H), 1.48–1.43 (m, 2H), 1.39–1.31 (m, 2H), 0.89 (s, 9H), 0.10–0.09 (m, 6H) ppm; ^{13}C NMR (101 MHz, $CDCl_3$): δ = 171.6, 156.8, 156.6, 151.4, 138.8, 138.6, 114.6, 114.5, 83.4, 83.4, 78.5, 77.9, 76.3, 76.1, 75.9, 52.7, 52.7, 52.5, 52.3, 52.1, 51.8, 51.3, 50.8, 49.8, 48.0, 46.1, 45.9, 40.1, 39.8, 34.7, 34.6, 33.9, 33.9, 32.7, 32.5, 28.1, 28.0, 27.6, 26.1, 26.0, 25.8, 17.9, 13.8, 3.5, 3.4, –4.4, –4.4 ppm; IR (film) ν = 3506, 2953, 2930, 2887, 2857, 1704, 1681, 1453, 1393, 1299, 1257, 1124, 839, 779 cm^{-1}; MS (ESI)*: m/z*: 641 [*M*+Na$^+$]; HRMS (ESI): *m/z:* calcd. for $C_{33}H_{54}N_2O_7SiNa$ [*M*+Na$^+$]: 641.35925, found: 641.35960.

Compound S7. Et_3N (1.4 mL, 10.0 mmol), DMAP (408 mg, 3.34 mmol) and MsCl (0.52 mL, 6.72 mmol) were successively added to a solution of compound **S6** in CH_2Cl_2 (23 mL) at 0 °C. After 5 min, the cooling bath was removed and the mixture stirred at ambient temperature for 2 h before sat. aq. $NaHCO_3$ (10 mL) was added. The aqueous layer was extracted with EtOAc (3 x 50 mL), the combined organic phases were washed with brine (5 mL), dried over $MgSO_4$, filtered, and concentrated. The crude product was purified by flash chromatography on silica gel (hexanes/EtOAc, 4:1 to 2:1) to afford the title compound as a white solid (1.98 g, 85 %). $[\alpha]_D^{20}$ = 24.1 (c = 1.0, $CHCl_3$); ^{1}H NMR (400 MHz, $CDCl_3$): δ = 5.76 (ddddd, *J* = 22.0, 10.1, 6.7, 5.1, 1.4 Hz, 1H), 5.05–4.79 (m, 2H), 4.44–4.28 (m, 2H), 4.23–4.09 (m, 2H), 3.78–3.64 (m, 3H), 3.43–3.33 (m, 1H), 3.26–3.11 (m, 2H), 3.00–2.95 (m, 3H), 2.58 (d, *J* = 18.4 Hz, 1H), 2.42–2.16 (m, 1H), 2.14–1.98 (m, 3H), 1.97–1.88 (m, 1H), 1.86–1.79 (m, 2H), 1.73 (q, *J* = 2.2 Hz, 4H), 1.53 (d, *J* = 1.4 Hz, 12H), 1.41–1.30 (m, 1H), 0.88 (s, 9H), 0.15–0.11 (m, 6H) ppm; ^{13}C NMR (101 MHz, $CDCl_3$): δ = 170.9, 156.5, 156.4, 151.1, 151.0, 138.5, 138.3, 114.9, 114.8, 85.2, 84.9, 83.8, 83.7, 78.1, 77.6, 76.1, 75.6, 67.8, 67.7, 52.9, 52.9, 51.8, 51.7, 51.5, 50.2, 49.7, 49.2, 48.0, 43.3, 43.3, 39.9, 39.6, 38.6, 38.5, 34.0, 33.8, 33.4, 33.4, 31.4, 31.3, 28.0, 25.8, 25.5, 25.3, 17.9, 13.9, 3.5, 3.4, –4.3, –4.5 ppm; IR (film) ν = 2953, 2931, 2858, 1704, 1450, 1389, 1367, 1257, 1125, 839, 779 cm^{-1}; MS (ESI)*: m/z*: 719 [*M*+Na$^+$]; HRMS (ESI): *m/z:* calcd. for $C_{34}H_{56}N_2O_9SSiNa$ [*M*+Na$^+$]: 719.33680, found: 719.33702.

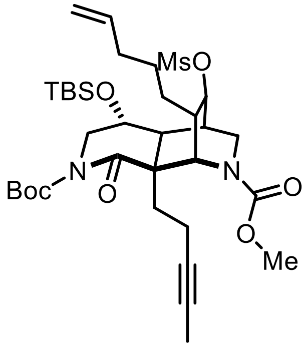

Compound S8. A solution of compound **S7** (900 mg, 1.29 mmol) in 2,6-lutidine (6.5 mL) was stirred at 170 °C for 5 d. The mixture was then cooled to ambient temperature and diluted with CH_2Cl_2 (4.0 mL). TBSOTf (1.5 mL, 6.53 mmol) was added to this solution at 0 °C. After 5 min, the cooling bath was removed and the

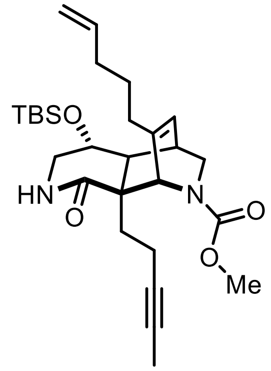

mixture was stirred at ambient temperature for 3 h. sat. aq. $NaHCO_3$ (10 mL) was added at 0 °C, followed by careful addition of HCl (2 M, 40 mL). The mixture was vigorously stirred for 10 min before it was extracted with EtOAc (3 x 50 mL). The combined extracts were washed with sat. aq. $NaHCO_3$ (5 mL), dried over $MgSO_4$, filtered and concentrated. The residue was purified by flash chromatography on silica gel (hexanes/EtOAc, 8:1 to 4:1) to afford the title product as a white solid (524 mg, 81 %). $[\alpha]_D^{20}$ = 27.1 (c = 0.9, $CHCl_3$); ^{1}H NMR (600 MHz, $CDCl_3$, mixture of rotamers, ca. 1.9:1): δ = 5.87 (d, *J* = 5.1 Hz, 0.65H, major), 5.83 (d, *J* = 5.7 Hz, 0.35H, minor), 5.83–5.72 (m, 1H), 5.70 (d, *J* = 4.5 Hz, 1H), 4.98–4.90 (m, 2H), 4.88 (d, *J* = 1.5 Hz, 0.35H, minor), 4.76 (d, *J* = 1.5 Hz, 0.65H, major), 3.72 (s, 2H, major), 3.68 (s, 1H, minor), 3.35 (ddd, *J* = 9.9, 8.9, 4.7 Hz, 1H), 3.19 (ddd, *J* = 8.9, 7.0, 1.9 Hz, 1H), 3.13 (ddt, *J* = 12.2, 10.0, 2.3 Hz, 1H), 3.07–2.92 (m, 2H), 2.79–2.76 (m, 0.65H, major), 2.75–2.71 (m, 0.35H, minor), 2.48–2.10 (m, 5H), 2.01–1.93 (m, 2H), 1.92–1.79 (m, 2H), 1.79–1.70 (m, 3H), 1.61–1.51 (m, 1H), 1.50–1.40 (m, 1H), 0.89 (s, 9H), 0.11–0.01 (m, 6H) ppm; ^{13}C NMR (151 MHz, $CDCl_3$): δ = 172.6, 156.1, 148.4, 147.4, 138.7, 138.5, 124.5, 123.8, 114.6, 114.5, 79.0, 78.4, 75.7, 75.5, 70.8, 70.7, 54.3, 54.2, 52.9, 52.6, 52.5, 52.5, 51.5, 51.3, 47.3, 47.1, 45.6, 45.6, 39.9, 39.7, 33.6, 33.3, 33.3, 33.3, 33.2, 26.2, 26.1, 25.7, 17.8, 14.1, 3.5, 3.4, –4.3, –4.3, –4.8, –4.8 ppm; IR (film) ν = 3200, 2952, 2929, 2858, 1701, 1666, 1448, 1388, 1122, 1068, 837, 776 cm^{-1}; MS (ESI)*: m/z*: 501 [*M*+H$^+$], 523 [*M*+Na$^+$]; HRMS (ESI): *m/z:* calcd. for $C_{28}H_{45}N_2O_4Si$ [*M*+H$^+$]: 501.31431, found: 501.31465.

Compound S9. NaH (327 mg, 13.6 mmol) was added to a solution of compound **S8** (908 mg, 1.81 mmol) and 1-bromo-4-pentene (0.5 mL mg, 4.22 mmol) in DMF (4.5 mL) at 0 °C. After stirring at this temperature

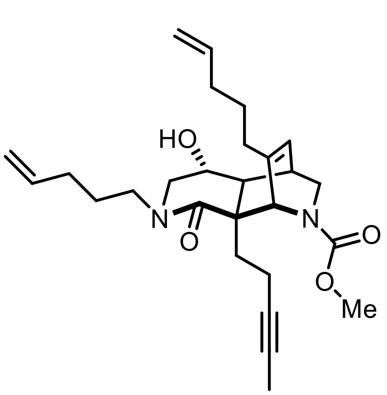

for 1 h, the mixture was added to a sat. aq. NH_4Cl (20 mL). The aqueous layer was extracted with EtOAc (3 x 20 mL), the compbined organic phases were washed with brine, dried over $MgSO_4$, filtered, and concentrated. The residue was purified by flash chromatography on silica gel (hexanes/*tert*-butyl methyl ether, 8:1 to 4:1) to afford a colorless oil.

TBAF (1 M in THF, 2.4 mL, 2.40 mmol) was added to a solution of this compound in THF (3.5 mL). The mixture was stirred at ambient temperature for 1 h before sat. aq. NH_4Cl (5.0 mL) was added. The aqueous layer was extracted with EtOAc

(3 x 10 mL), the combined organic phases were washed with brine, dried over Na_2SO_4, filtered and concentrated. The residue was purified by flash chromatography on silica gel (hexanes/EtOAc, 6:1 to 2:1) to afford the title compound as a yellow oil (808 mg, 98%). $[\alpha]_D^{20}$ = 60.9 (c = 1.4, $CHCl_3$); ^{1}H NMR (400 MHz, $CDCl_3$, mixture of rotamers, ca. 2:1): δ = 5.90–5.84 (m, 1H), 5.83–5.69 (m, 2H), 5.05–4.77 (m, 5H), 3.72 (s, 2H, major), 3.68 (s, 1H, minor), 3.44–3.36 (m, 1H), 3.35–3.25 (m, 2H), 3.24–3.16 (m, 2H), 3.12 (dd, *J* = 12.0, 4.4 Hz, 1H), 2.99 (ddd, *J* = 26.5, 10.4, 2.8 Hz, 1H), 2.91–2.83 (m, 1H), 2.50–2.25 (m, 2H), 2.25–2.05 (m, 3H), 2.03–1.92 (m, 4H), 1.92–1.83 (m, 1H), 1.80–1.69 (m, 5H), 1.63–1.59 (m, 1H), 1.56–1.48 (m, 2H), 1.48–1.37 (m, 1H) ppm; ^{13}C NMR (101 MHz, $CDCl_3$): δ = 169.9, 169.9, 156.2, 156.1, 148.3, 147.4, 138.7, 138.5, 137.7, 137.6, 124.3, 123.6, 115.1, 115.1, 114.5, 114.5, 79.1, 78.6, 75.7, 75.6, 69.7, 69.6, 53.9, 53.8, 53.5, 53.3, 52.6, 52.5, 52.0, 51.9, 51.1, 47.3, 47.3, 47.1, 39.8, 39.5, 33.7, 33.4, 33.2, 33.2, 33.1, 30.8, 30.8, 26.7, 26.7, 26.1, 26.0, 14.2, 3.5, 3.5 ppm; IR (film) ν = 3409, 2925, 2847, 1699, 1678, 1449, 1391, 1271, 1115, 912, 767 cm^{-1}; MS (ESI)*: m/z*: 455 [*M*+H$^+$], 477 [*M*+Na$^+$]; HRMS (ESI): *m/z:* calcd. for $C_{27}H_{38}N_2O_4Na$ [*M*+H$^+$]: 477.27238, found: 477.27262.

Compound S10. Et_3N (0.55 mL, 3.95 mmol), DMAP (160 mg, 1.31 mmol) and chloromethanesulfonyl chloride (McCl, 0.23 mL, 2.53 mmol) were added to a solution of compound **S9** (583 mg, 1.28 mmol) in CH_2Cl_2 (6.5 mL) at 0 °C. After 30 min, the cooling bath was removed and the mixture stirred at ambient temperature for 4 h before sat. aq. $NaHCO_3$ (5 mL) was added. The aqueous phase was extracted with EtOAc (3 x 20 mL), the combined organic layers were washed with brine (2 mL), dried over $MgSO_4$, filtered, and concentrated.

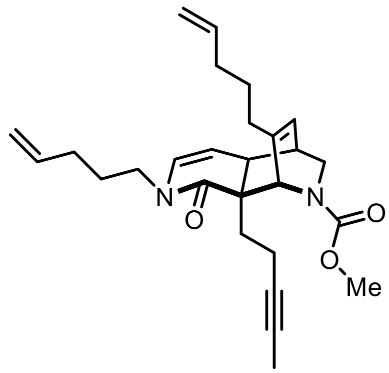

The resulting crude product was dissolved in toluene (0.8 mL). DBU (1.2 mL) was added and the resulting mixture stirred at 100 °C for 2 h. After cooling to ambient temperature, the mixture was loaded onto a silica gel column and the product eluted with (hexanes/EtOAc, 8:1 to 4:1) to afford the title product as a colorless oil (432 mg). $[\alpha]_D^{20}$ = 28.7 (c = 1.0, $CHCl_3$); ^{1}H NMR (400 MHz, $CDCl_3$, mixture of rotamers, ca. 2:1): δ = 5.97 (ddd, *J* = 8.8, 6.7, 1.7 Hz, 1H), 5.83–5.67 (m, 3H), 5.07–4.75 (m, 6H), 3.72 (s, 2H), 3.69 (s, 1H), 3.52 (dddd, *J* = 13.4, 8.1, 6.5, 2.1 Hz, 1H), 3.21 (ddd, *J* = 10.0, 4.8, 2.3 Hz, 1H), 3.07 (dddd, *J* = 13.8, 9.0, 6.5, 2.6 Hz, 1H), 2.84 (ddd, *J* = 25.9, 10.0, 2.4 Hz, 1H), 2.72–2.57 (m, 1H), 2.30 (dq, *J* = 3.4, 1.8 Hz, 1H), 2.23–1.89 (m, 10H), 1.74 (q, *J* = 2.6 Hz, 3H), 1.54 (tdd, *J* = 8.0, 6.5, 1.6 Hz, 3H), 1.46–1.33 (m, 1H) ppm; ^{13}C NMR (101 MHz, $CDCl_3$): δ = 168.7, 168.7, 156.5, 156.4, 147.6, 146.8, 138.8, 138.6, 137.7, 137.7, 128.0, 127.9, 125.9, 125.3, 115.4, 115.4, 114.7, 107.1, 106.8, 78.7, 78.6, 75.8, 75.8, 56.5, 56.2, 53.5, 53.4, 52.7, 52.6, 48.3, 48.2,

46.9, 46.8, 44.2, 41.2, 37.7, 37.5, 33.7, 33.7, 33.4, 33.3, 30.8, 30.8, 27.6, 27., 26.2, 26.1, 15.1 ppm; IR (film) ν = 2925, 2861, 1697, 1642, 1389, 1338, 1255, 1110, 911, 766 cm^{-1}; MS (ESI): *m/z*: 437 [*M*+H^+], 459 [*M*+Na^+]; HRMS (ESI): *m/z:* calcd. for $C_{27}H_{38}N_2O_3Na$ [*M*+Na^+]: 459.26181, found: 459.26175.

Compound 56. $NaBH_3CN$ (180 mg, 2.86 mmol) was added to a solution of compound **S10** (250 mg, 0.573 mmol equiv) in CH_2Cl_2 (23 m) at 0 °C. TFA (0.44 mL, 5.74 mmol) was slowly added and the mixture stirred for 1 h at ambient temperature before the reaction was quenched with sat. $NaHCO_3$ (5.0 mL). The aqueous layer was extracted with EtOAc (3 x 20 mL), the combined extracts were washed with brine, dried over Na_2SO_4, filtered, and concentrated. The residue was purified by flash chromatography on silica gel (hexanes/EtOAc, 8:1 to 3:1) to afford the title product as a colorless oil (151 mg, 60%). $[\alpha]_D^{20}$ = 71.2 (c = 0.6, $CHCl_3$); ^1H NMR (400 MHz, $CDCl_3$, mixture of rotamers, ca. 2:1): δ = 5.88–5.83 (m, 1H), 5.82–5.73 (m, 2H), 5.04–4.89 (m, 4H), 4.87 (d, *J* = 1.6 Hz, 0.4 H, minor), 4.76 (d, *J* = 1.5 Hz, 0.6 H, major), 3.72 (s, 2 H, major), 3.68 (s, 1H, minor), 3.36–3.18 (m, 4H), 3.07 (ddd, *J* = 12.6, 5.3, 3.6 Hz, 1H), 2.96 (dd, *J* = 10.3, 2.6 Hz, 0.6 H, major), 2.88 (dd, *J* = 10.0, 2.6 Hz, 0.4 H, major), 2.53 (dq, *J* = 6.7, 2.3 Hz, 0.7 H, major), 2.49 (dt, *J* = 6.6, 2.2 Hz, 0.3 H, minor), 2.38–2.04 (m, 5H), 2.03–1.88 (m, 5H), 1.84–1.79 (m, 1H), 1.74 (t, *J* = 2.5 Hz, 3H), 1.64 (dddd, *J* = 13.6, 11.8, 7.1, 4.7 Hz, 1H), 1.57 –1.50 (m, 3H), 1.47–1.34 (m, 2H) ppm; ^{13}C NMR (101 MHz, $CDCl_3$): δ = 170.4, 170.4, 156.2, 156.2, 147.2, 146.4, 138.8, 138.6, 137.9, 137.9, 137.8, 124.3, 123.5, 115.0, 114.9, 114.5, 114.5, 79.2, 78.8, 75.4, 75.3, 54.4, 54.3, 52.5, 52.4, 52.1, 51.9, 48.2, 48.0, 47.5, 47.5, 45.2, 45.2, 45.1, 44.9, 39.5, 39.4, 37.2, 36.9, 33.3, 33.3, 33.2, 33.2, 31.0, 30.9, 29.8, 29.8, 29.7, 26.7, 26.1, 26.0, 14.5, 14.4, 3.5, 3.5 ppm; IR (film) ν = 2924, 1679, 1632, 1648, 1446, 1389, 1286, 1109, 911, 767 cm^{-1}; MS (ESI): *m/z*: 439 [*M*+H^+], 461 [*M*+Na^+]; HRMS (ESI): *m/z:* calcd. for $C_{34}H_{50}F_3N_2O_7SSi$ [*M*+H^+]: 461.27746, found: 461.27755.

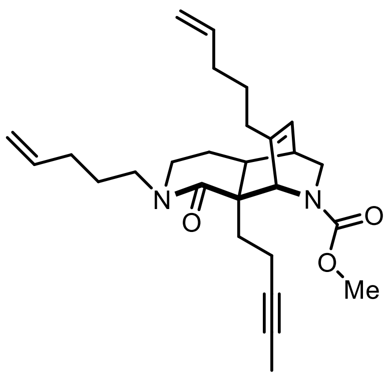

Compound 57. L-Selectride (1 M in THF, 1.70 mL, 1.70 mol) was added to a solution of compound **56** (151 mg, 0.344 mol) in THF (0.3 mL) at ambient temperature. The mixture was stirred at 40 °C for 12h before the reaction was quenched by cautious addition of MeOH (1.0 mL) at 0 °C. The mixture was loaded onto an amino cartridge (pre-equilibrated with MeOH, H_2O, MeOH (volume of three column length each)) and the product was eluted with MeOH:H_2O (90:10) to provide a white solid.

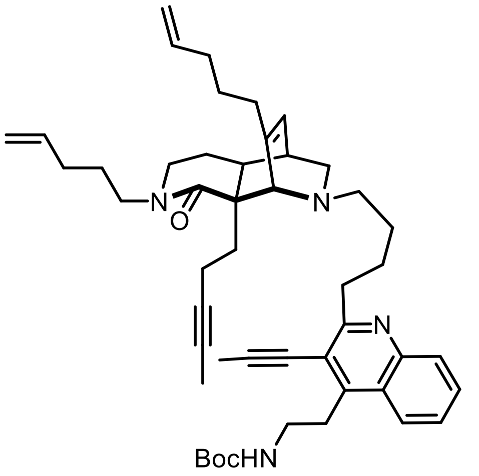

HOAc (0.1 mL, 1.75 mmol) was added to a solution of this secondary amine and aldehyde **52** (393 mg, 1.03 mmol) in CH_2Cl_2 (1.4 mL) at ambient temperature. After stirring for 30 min, $NaBH(OAc)_3$ (256 mg, 1.21 mmol) was added and the resulting mixture was stirred at ambient temperature for 3 h before the reaction was quenched with sat. aq. $NaHCO_3$ (0.5 mL). After removing the solvent under argon, the residue was purified on an amino cartridge (MeOH; 100%) to provide a white solid. This compound was then subjected to preparative HPLC (Kromasil-5-C18, 5 µm, 150 mm × 30 mm, MeOH: H_2O = 95:5, 35 mL/min, λ = 254 nm, t = 3.8 min) to afford the title compound as a colorless oil (192 mg, 75 %). $[\alpha]_D^{20}$ = –22.3 (c = 0.3, $CHCl_3$); ^{1}H NMR (400 MHz, $CDCl_3$): δ = 8.05 (d, *J* = 8.4 Hz, 1H), 8.00 (dd, *J* = 8.5, 1.3 Hz, 1H), 7.63 (ddd, *J* = 8.4, 6.9, 1.4 Hz, 1H), 7.49 (ddd, *J* = 8.2, 6.8, 1.3 Hz, 1H), 5.88–5.67 (m, 3H), 5.04–4.87 (m, 4H), 4.72 (s, 1H), 3.49 (d, *J* = 3.0 Hz, 4H), 3.39 (d, *J* = 1.6 Hz, 1H), 3.31–3.10 (m, 5H), 3.04 (ddd, *J* = 20.6, 7.8, 3.1 Hz, 2H), 2.49 (ddd, *J* = 11.5, 8.7, 7.0 Hz, 1H), 2.30–2.17 (m, 6H), 2.17–2.07 (m, 3H), 2.07–1.75 (m, 10H), 1.73–1.67 (m, 2H), 1.65 (d, *J* = 2.5 Hz, 3H), 1.58–1.47 (m, 4H), 1.44 (s, 9H), 1.36 (dqt, *J* = 21.1, 8.4, 3.4 Hz, 2H) ppm; ^{13}C NMR (101 MHz, $CDCl_3$): δ = 171.9, 163.3, 156.0, 147.1, 146.1, 144.5, 138.9, 138.1, 129.5, 129.2, 126.2, 125.7, 123.8, 122.5, 117.7, 114.8, 114.4, 95.6, 79.8, 79.2, 76.1, 74.9, 62.5, 58.0, 55.6, 52.2, 47.4, 45.4, 44.2, 40.4, 39.1, 37.9, 37.9, 34.6, 33.6, 31.0, 30.8, 29.7, 28.8, 28.4, 26.8, 26.7, 26.0, 14.7, 4.9, 3.5 ppm; IR (film) ν = 3327, 2974, 2928, 2858, 1708, 1626, 1496, 1391, 1170, 911, 760 cm^{-1}; MS (ESI)*: m/z*: 745 [*M*+H$^+$], 767 [*M*+Na$^+$]; HRMS (ESI): *m/z:* calcd. for $C_{48}H_{65}N_4O_3$ [*M*+H$^+$]: 745.50512, found: 745.50462.

Compound 58. A solution of ligand **30** (37.4 mg, 0.0475 mmol) in toluene (1.0 mL) was added to complex

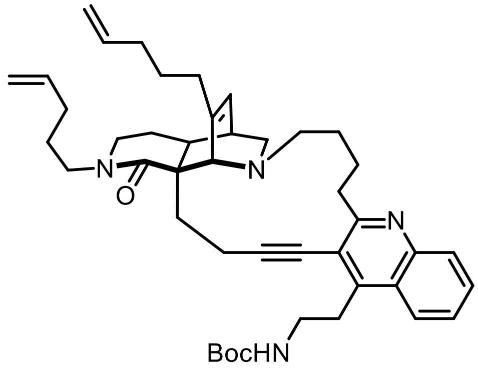

29 (27.6 mg, 0.0474 mmol), and the resulting mixture was stirred for 5 min. This mixture was added to a suspension containing diyne **57** (118 mg, 0.158 mmol) and 5 Å MS powder (3.0 g) in toluene (79 mL) at 110 °C. After 10 min, the mixture was allowed to cool to ambient temperature, before it was filtered through a pad of Celite and the filtrate was concentrated. The crude product was then subjected to preparative HPLC (Kromasil-5-C18, 5 µm, 150 mm × 30 mm, MeOH: H_2O = 95:5, 35 mL/min, λ = 254 nm, t = 6.6 min) to afford the title compound as a white solid (84.7 mg, 77%). $[\alpha]_D^{20}$ = –48.6 (c = 0.35, $CHCl_3$); ^1H NMR (400 MHz, $CDCl_3$): δ = 8.06 (d, *J* = 7.9 Hz, 1H), 7.99 (d, *J* = 8.3 Hz, 1H), 7.67–7.58 (m, 1H), 7.52–7.43 (m, 1H), 5.82 (dtt, *J* = 19.5, 6.5, 3.1 Hz, 3H), 5.00 (dddd, *J* = 20.9, 18.9, 10.2, 5.8 Hz, 4H), 4.84 (s, 1H), 3.73 (d, *J* = 1.6 Hz, 1H), 3.56–3.41 (m, 6H), 3.39–3.18 (m, 1H), 3.18–2.97 (m, 3H), 2.93 (dd, *J* = 9.4, 2.1 Hz, 1H), 2.81 (td, *J* = 11.7, 2.6 Hz, 1H), 2.74–2.58 (m, 2H), 2.50 (t, *J* = 11.6 Hz, 1H), 2.37–1.82 (m, 12H), 1.73 (dd, *J* = 9.5, 2.5 Hz, 1H), 1.55 (dq, *J* = 15.4, 7.6, 6.8 Hz, 6H), 1.44 (d, *J* = 5.8 Hz, 10H), 1.30–1.18 (m, 1H) ppm; ^{13}C NMR (101 MHz, $CDCl_3$): δ = 171.9, 164.3, 156.0, 146.4, 146.1, 145.3, 139.0, 138.0, 129.2, 129.1, 126.2, 125.8, 123.9, 122.3, 117.9, 115.0, 114.3, 102.2, 79.2, 76.3, 60.8, 55.3, 54.3, 52.5, 47.3, 46.9, 45.3, 40.5, 38.6, 37.4, 37.0, 34.6, 33.7, 30.9, 30.8, 30.4, 28.4, 27.8, 26.9, 26.2, 25.1, 13.4 ppm; IR (film) ν = 2929, 1706, 1632, 1496, 1449, 1365, 1250, 1170, 911, 760 cm^{-1}; MS (ESI)*: m/z*: 691 [*M*+H^+], 713 [*M*+Na^+]; HRMS (ESI): *m/z:* calcd. for $C_{44}H_{59}N_3O_4$ [*M*+H^+]: 691.45817, found: 691.45836.

Compound S11. DIBAL-H (1.0 M in Hexane, 0.4 mL, 0.40 mmol) was added to a solution of compound **58**

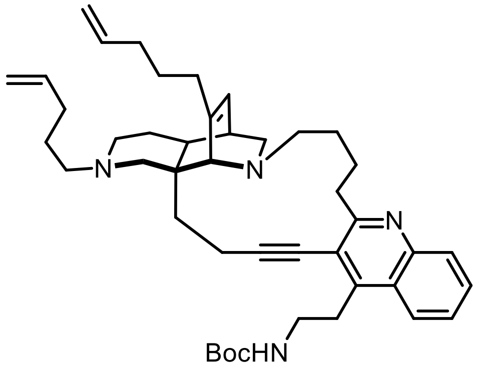

(42 mg, 0.0608 mmol) in Et_2O (0.4 mL) at 0 °C. The mixture was gradually warmed to 20 °C over 2h before the reaction was quenched with EtOAc (0.4 mL) at 0 °C. After 10 min, aq. NaOH (3.0 M, 0.1 mL) was added and the resulting mixture was stirred for another 30 min before it was extracted with EtOAc (3 x 5 mL), the combined extracts were washed with brine, dried over Na_2SO_4, filtered, and concentrated. The residue was dissolved in CH_2Cl_2 (0.9 mL). MnO_2 (372 mg, 4.28 mmol) was added and the resulting mixture was stirred at 40 °C for 1 d. Then the mixture was filtered through a PFE filter (0.45 μL), the filtrate was concentrated, and the crude product was subjected to preparative HPLC (Kromasil-5-C18, 5 µm, 150 mm × 30 mm, MeOH: 20 mmol NH_4HCO_3 PH 9 = 95:5, 35 mL/min, λ = 254 nm, t = 15.2 min)

to afford the title compound as a yellow solid (25.6 mg, 62%). $[\alpha]_D^{20} = -82.2$ (c = 0.4, $CHCl_3$); ^1H NMR (600 MHz, $CDCl_3$): δ = 8.08 (s, 1H), 7.98 (d, *J* = 8.4 Hz, 1H), 7.63 (ddd, *J* = 8.3, 6.8, 1.3 Hz, 1H), 7.50 (t, *J* = 7.7 Hz, 1H), 5.89–5.78 (m, 3H), 5.08–5.01 (m, 2H), 4.99 (dd, *J* = 10.3, 1.8 Hz, 2H), 4.77 (s, 1H), 3.54–3.43 (m, 5H), 2.90 (d, *J* = 9.6 Hz, 1H), 2.83 (s, 1H), 2.77–2.71 (m, 2H), 2.67–2.59 (m, 1H), 2.56–2.44 (m, 3H), 2.41–2.37 (m, 2H), 2.24 (s, 2H), 2.20–2.00 (m, 9H), 1.77–1.68 (m, 4H), 1.68–1.49 (m, 5H), 1.47–1.37 (m, 11H), 1.34–1.25 (m, 1H), 1.04 (s, 1H) ppm; ^{13}C NMR (151 MHz, $CDCl_3$): δ = 164.4, 156.0, 146.3, 146.1, 142.9, 138.6, 129.2, 126.2, 125.8, 123.9, 121.0, 117.8, 114.7, 114.5, 102.3, 76.2, 62.8, 58.6, 55.2, 53.8, 50.3, 49.6, 47.2, 43.1, 40.5, 38.6, 37.3, 36.8, 35.8, 31.4, 30.9, 28.4, 27.8, 27.3, 26.8, 26.0, 25.1, 13.6 ppm; IR (film) ν = 2930, 2854, 2225, 1703, 1497, 1447, 1365, 1250, 1170, 911, 759 cm^{-1}; MS (ESI): *m/z*: 677 [*M*+H^+]; HRMS (ESI): *m/z:* calcd. for $C_{44}H_{61}N_2O_4$ [*M*+H^+]: 677.47890, found: 677.47917.

Completion of the Total Synthesis

(*E*)-2,3-Dibromo-8-iodooct-2-ene (61). Bromine (5.2 mL, 101.5 mmol) was added to a solution of oct-6-yn-1-ol (10.6 g, 84.0 mmol) in CH_2Cl_2 (420 mL) at 0 °C. After stirring for 20 min at this temperature, the reaction mixture was poured into a solution of sat. aq. Na_2SO_3 (500 mL). The aqueous phase was extracted with CH_2Cl_2 (3 x 500 mL), the combined organic layers were washed with brine, dried with anhydrous $MgSO_4$ and filtered, and the solvent was evaporated. The crude dibromide **60** thus obtained was used in the next step without further purification.

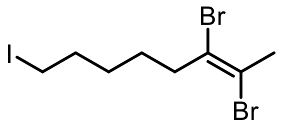

Iodine (25.6 g, 100.9 mmol) was added to a vigorously stirred solution of PPh_3 (26.5 g, 101.0 mmol) and imidazole (6.88 g, 101.1 mmol) in CH_2Cl_2 (280 mL) at 0 C. After stirring at this temperature for 30 min, a solution of the crude dibromide **60** in CH_2Cl_2 (50 mL) was added and the resulting mixture was stirred for 2 h before the reaction was quenched with aq. sat. $Na_2S_2O_3$ (200 mL). The aqueous phase was extracted with CH_2Cl_2 (2 x 300 mL), the combined extracts were dried over Na_2SO_4 and evaporated. The residue was purified by chromatography on silica (pentane) to afford the title compound as a colorless oil (33.0 g, 99 %). ^1H NMR (400 MHz, $CDCl_3$): δ = 3.20 (t, *J* = 7.1 Hz, 2H), 2.67 (t, *J* = 7.5 Hz, 2H), 2.42 (d, *J* = 1.1 Hz, 3H), 1.86 (ddd, *J* = 13.0, 7.9, 6.5 Hz, 2H), 1.65–1.55 (m, 2H), 1.48–1.38 (m, 2H). ^{13}C NMR (101 MHz, $CDCl_3$): δ = 121.6, 115.6, 40.3, 33.2, 29.3, 28.8, 26.3, 6.7 ppm; IR (film) $\tilde{\nu}$ = 2929, 2857, 1453, 1428, 1375, 1349, 1298, 1267, 1204, 1165, 1104, 1069, 1030, 957, 723, 615, 505 cm^{-1}; MS (EI): *m/z* (%):107 (100), 213 (43), 396 (4); HRMS (ESI): *m/z:* calcd. for $C_8H_{13}IBr_2$ [M^+]: 393.84235, found: 393.84232.

Allyl (*E*)-1-benzyl-3-(6,7-dibromooct-6-en-1-yl)-4-oxopiperidine-3-carboxylate (S12). Cs_2CO_3 (27.2 g , 83.5 mmol) was added to a solution of compound **19b** (14.5 g, 53.0 mmol) and iodide **61** (33.0 g, 83.4 mmol) in DMF (128 mL) at ambient temperature. The mixture was stirred for 12 h before the reaction was quenched with sat. aq. NH_4Cl (10 mL). The resulting mixture was extracted with EtOAc (3 x 300 mL), and the combined organic extracts were dried over anhydrous Na_2SO_4, filtered, and concentrated under reduced pressure. The residue was purified by flash chromatography on silica (EtOAc/hexanes, 1:8) to afford the title compound as a colorless oil (19.1 g, 67 %).^{1}H NMR (400 MHz, $CDCl_3$): δ = 7.42–7.14 (m, 5H), 5.89 (ddt, *J* = 17.3, 10.4, 5.8 Hz, 1H), 5.38–5.20 (m, 2H), 4.76–4.51 (m, 2H), 3.64–3.51 (m, 2H), 3.41 (dd, *J* = 11.5, 2.6 Hz, 1H), 2.98 (dtd, *J* = 12.6, 5.2, 2.7 Hz, 1H), 2.91–2.76 (m, 1H), 2.66–2.58 (m, 2H), 2.49–2.32 (m, 5H), 2.25 (d, *J* = 11.6 Hz, 1H), 1.87–1.72 (m, 1H), 1.61–1.46 (m, 3H), 1.45–1.34 (m, 1H), 1.33–1.22 (m, 2H), 1.17–1.05 (m, 1H) ppm; ^{13}C NMR (101 MHz, $CDCl_3$): δ = 205.9, 171.2, 137.7, 131.6, 128.7, 128.1, 127.2, 121.8, 118.6, 115.1, 65.5, 61.7, 61.1, 61.0, 53.4, 40.4, 40.3, 31.9, 28.7, 28.6, 26.9, 24.1 ppm; IR (film) $\tilde{\nu}$ = 3027, 2927, 2859, 2805, 1716, 1649, 1494, 1454, 1348, 1318, 1221, 1195, 1160, 1122, 1073, 1027, 972, 997, 931, 820, 734, 698, 616, 554, 501, 462 cm^{-1}; MS (ESI): *m/z*: 540 [M+H$^+$]; 562 [M+Na$^+$]; HRMS (ESI): *m/z:* calcd. for $C_{24}H_{32}NO_3Br_2$ [M+H$^+$]: 540.07369, found: 540.07475.

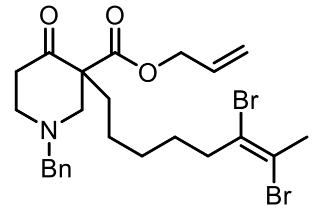

3-Allyl 1-methyl (*E*)-3-(6,7-dibromooct-6-en-1-yl)-4-oxopiperidine-1,3-dicarboxylate (62). Methyl chloroformate (13.6 mL, 176 mmol) was added to a solution of **S12** (19.1 g, 35.3 mmol) in toluene (35 mL) at ambient temperature. The mixture was stirred at 100 °C for 6 h before it was directly loaded on a column of silica. The product was eluted with hexanes/EtOAc (5:1 to 1:1) to provide the desired product as a colorless oil (17.4 g, 97 %). ^{1}H NMR (400 MHz, $CDCl_3$): δ = 5.87 (ddt, *J* = 16.5, 10.5, 5.9 Hz, 1H), 5.37–5.18 (m, 2H), 4.62 (d, *J* = 5.8 Hz, 2H), 4.58–4.47 (m, 1H), 4.11 (s, 1H), 3.74 (s, 3H), 3.39 (s, 1H), 3.22–3.08 (m, 1H), 2.72–2.60 (m, 3H), 2.47 (dt, *J* = 14.6, 4.8 Hz, 1H), 2.40 (s, 3H), 1.91–1.80 (m, 1H), 1.58 (td, *J* = 19.4, 14.8, 8.4 Hz, 3H), 1.31 (dt, *J* = 13.3, 6.9 Hz, 4H) ppm; ^{13}C NMR (101 MHz, $CDCl_3$): δ = 204.3, 169.8, 155.7, 131.3, 121.8, 119.2, 115.4, 66.1, 61.2, 53.1, 50.2, 43.7, 40.3, 39.7, 31.5, 28.7, 28.6, 27.0, 24.0 ppm; IR (film) $\tilde{\nu}$ = 2930, 2860, 1703, 1650, 1448, 1412, 1376, 1308, 1272, 1236, 1192, 1132, 1073, 994, 933, 767, 616 cm^{-1}; MS (ESI): *m/z*: 508 [M+H$^+$]; 530 [M+Na$^+$]; HRMS (ESI): *m/z:* calcd. for $C_{19}H_{28}NO_5Br_2$ [M+H$^+$]: 508.03290, found: 508.03322.

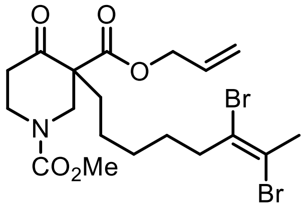

Methyl (*E*)-5-(6,7-dibromooct-6-en-1-yl)-4-oxo-3,4-dihydropyridine-1(2*H*)-carboxylate (63). $Pd_2(dba)_3$

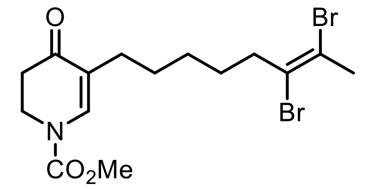

(859 mg, 0.938 mmol) was added to a solution of compound **63** (9.54 g, 18.7 mmol) in CH_3CN (94 mL). The mixture was stirred at 80 °C for 30 min before it was cooled to ambient temperature and filtered filtered through a pad of Celite, rinsing with *tert*-butyl methyl ether (100 mL). The combined filtrates were evaporated and the residue was purified by flash chromatography on silica (hexanes/EtOAc, 4:1 to 1:1) to afford the title compound as a colorless oil (7.22 g, 92 %).^{1}H NMR (400 MHz, $CDCl_3$): δ = 7.66 (s, 1H), 3.98 (t, *J* = 7.3 Hz, 2H), 3.85 (d, *J* = 1.0 Hz, 3H), 2.72–2.62 (m, 2H), 2.59–2.48 (m, 2H), 2.41 (d, *J* = 1.1 Hz, 3H), 2.17 (t, *J* = 7.6 Hz, 2H), 1.62–1.53 (m, 2H), 1.50–1.40 (m, 2H), 1.33 (tt, *J* = 10.2, 4.3 Hz, 2H) ppm; ^{13}C NMR (101 MHz, $CDCl_3$): δ = 193.2, 122.0, 115.2, 53.9, 42.6, 40.5, 36.0, 29.0, 28.8, 28.2, 27.2, 27.2 ppm; IR (film) $\tilde{\nu}$ = 2926, 2857, 1723, 1665, 1617, 1439, 1398, 1370, 1322, 1301, 1243, 1204, 1153, 1122, 1061, 1048, 1006, 974, 917, 766, 668, 615, 511 cm^{-1}; MS (ESI)*: m/z*: 422 [*M*+H$^+$], 444 [*M*+Na$^+$]; HRMS (ESI): *m/z:* calcd. for $C_{15}H_{22}NO_3Br_2$ [*M*+H$^+$]: 421.99612, found: 421.99593.

Compound 64. The Michael donor **53** (4.50 g, 10.6 mmol) was dissolved in THF (35 mL) and the solution

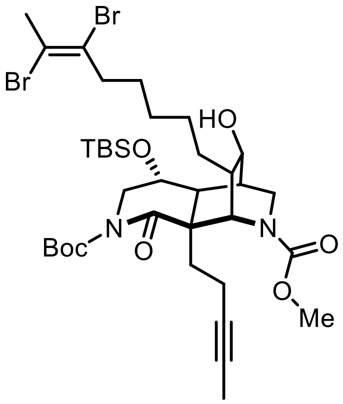

cooled to –50 °C before a solution of LiO*t*Bu (854 mg, 10.7 mmol) in THF (18 mL) was added dropwise. After the addition was complete, stirring was continued for 10 min at –50 °C. Then, a solution of the Michael acceptor **53** (3.27 g, 8.89 mmol) in THF (17 mL) was added dropwise at –50 °C. The reaction was warmed to 25 °C over the course of 5 h and then stirred at that temperature for another 16 h. DMAP (1.63 g, 13.3 mmol) and Boc_2O (1.63 g, 13.3 mmol) were added and stirring continued for 1 h before the reaction was quenched with sat. aq. NH_4Cl solution (20 mL). The aqueous phase was extracted with EtOAc (3 x 100 mL), the combined extracts were washed with brine, dried over magnesium sulfate and filtered. After removal of the organic solvents in vacuum, the crude material was purified by flash chromatography on silica (hexanes/EtOAc, 10:1 to 6:1) to afford the desired product **64**.

$NaBH_4$ (1.0 g, 26.4 mmol) was added in portions to a solution of compound **64** in MeOH (35.0 mL) at 0 °C. The mixture was stirred at this temperature for 30 min before the reaction was quenched with sat. aq. NH_4Cl (10 mL). The resulting mixture was extracted with EtOAc (3 x 50 mL), the combined organic phases were washed with brine (10 mL), dried over $MgSO_4$ and filtered. After evaporation of the solvent, the crude product was purified by flash chromatography on silica gel (hexanes/EtOAc, 8:1 to 4:1) to afford the title product as a white solid (3.98 g, 55 %). $[\alpha]_D^{20}$ = 48.0 (c = 1.0 , $CHCl_3$); ^{1}H NMR (400 MHz, $CDCl_3$, mixture of

rotamers, 2.3:1) : δ = 4.46 (tdd, *J* = 10.6, 4.1, 1.8 Hz, 1H), 4.27 (s, 0.3H, minor), 4.18 (s, 0.7H, major), 4.09 (dd, *J* = 12.5, 4.1 Hz, 1H), 3.70 (dd, *J* = 2.5, 1.1 Hz, 3H), 3.57 – 3.49 (m, 1H), 3.30 (ddd, *J* = 22.5, 11.5, 2.8 Hz, 1H), 3.17 – 3.03 (m, 2H), 2.67 – 2.58 (m, 2H), 2.39 (dd, *J* = 2.6, 1.1 Hz, 3H), 2.35 – 2.25 (m, 1H), 2.25 – 2.11 (m, 1H), 1.98 (dtt, *J* = 16.5, 8.5, 4.0 Hz, 1H), 1.84 – 1.58 (m, 8H), 1.58 – 1.23 (m, 17H), 0.87 (s, 9H), 0.08 (s, 6H); ^{13}C NMR (101 MHz, $CDCl_3$): δ = 171.6, 156.8, 156.5, 151.5, 151.4, 122.2, 122.0, 115.2, 115.1, 83.4, 83.3, 78.5, 77.9, 76.2, 76.0, 75.8, 75.5, 67.8, 67.7, 52.7, 52.5, 52.2, 52.1, 51.8, 51.3, 50.8, 49.7, 48.0, 46.2, 46.0, 40.4, 40.1, 39.8, 34.6, 34.5, 33.2, 32.9, 28.7, 28.7, 28.6, 28.0, 27.2, 27.2, 26.5, 26.4, 25.7, 17.9, 13.8, 13.8, 3.5, 3.4, –4.4, –4.5; IR (film) $\tilde{\nu}$ = 3502, 2951, 2929, 2884, 2857, 1766, 1703, 1680, 1454, 1393, 1369, 1339, 1296, 1255, 1191, 1156, 1122, 1067, 991, 939, 865, 838, 808, 779, 756, 685, 671, 666 cm^{-1}; MS (ESI)*:* *m/z*: 839 [*M*+Na$^+$]; HRMS (ESI): *m/z:* calcd. for $C_{36}H_{58}N_2O_7SiBr_2Na$ [*M*+Na$^+$]: 839.22725, found: 839.22744.

Compound S13. Et_3N (1.6 mL, 11.5 mmol), DMAP (474 mg, 3.88 mmol) and MsCl (0.75 mL, 9.69 mmol) were successively added to a solution of alcohol **64** (3.18 g, 3.88 mmol) in CH_2Cl_2 (16.0 mL) at 0 °C. After 5 min, the cooling bath was removed and the mixture stirred at ambient temperature for 2 h before sat. aq. $NaHCO_3$ (10 mL) was added. The aqueous layer was extracted with EtOAc (3 x 50 mL), the combined extracts were washed with brine (5 mL), dried over $MgSO_4$ and filtered, and the solvent was evaporated in vacuum. The residue was purified by flash chromatography on silica gel (hexanes/EtOAc, 4:1 to 2:1) to afford the title compound as a white solid (3.28 g, 94 %). $[\alpha]_{\mathrm{D}}^{20}$ = 29.4 (c = 1.0, $CHCl_3$); ^{1}H NMR (400 MHz, $CDCl_3$, mixture of rotamers, 2:1): δ = 4.42–4.29 (m, 2H), 4.23–4.15 (m, 2H), 3.75 (s, 2H, major), 3.73 (s, 1H, minor), 3.40 (td, *J* = 11.8, 2.7 Hz, 1H), 3.28–3.16 (m, 2H), 3.02–2.99 (m, 3H), 2.67–2.56 (m, 3H), 2.40 (dt, *J* = 3.2, 1.0 Hz, 3H), 2.22 (dddd, *J* = 16.3, 9.6, 6.2, 2.8 Hz, 1H), 2.08–1.99 (m, 1H), 1.97–1.89 (m, 1H), 1.87–1.81 (m, 2H), 1.73 (h, *J* = 2.5 Hz, 4H), 1.60–1.56 (m, 3H), 1.53 (s, 10H), 1.43–1.24 (m, 4H), 0.89 (s, 9H), 0.15 (s, 3H), 0.14 (s, 3H) ppm; ^{13}C NMR (101 MHz, $CDCl_3$): δ = 170.9, 156.6, 156.5, 151.1, 151.0, 122.1, 121.9, 115.3, 85.1, 84.8, 83.8, 83.7, 78.2, 77.6, 76.1, 75.8, 67.8, 67.7, 52.9, 51.8, 51.7, 51.6, 51.5, 50.3, 49.8, 49.2, 48.1, 43.5, 43.4, 40.5, 40.4, 40.0, 39.6, 38.7, 38.6, 34.0, 33.8, 32.2, 31.9, 28.8, 28.5, 28.4, 28.0, 27.2, 27.1, 26.1, 26.1, 25.8, 17.9, 13.9, 3.5, 3.4, –4.2, –4.5 ppm; IR (film) $\tilde{\nu}$ = 2931, 2858, 1770, 1704, 1449, 1389, 1367, 1340, 1298, 1256, 1177, 1155, 1125, 1065, 991, 962, 941, 899, 838, 779, 754, 666, 617, 526, 490 cm^{-1}; MS (ESI)*:* *m/z*: 917 [*M*+Na$^+$]; HRMS (ESI): *m/z:* calcd. for $C_{37}H_{60}N_2O_9SiBr_2Na$ [*M*+Na$^+$]: 917.20480, found: 917.20512.

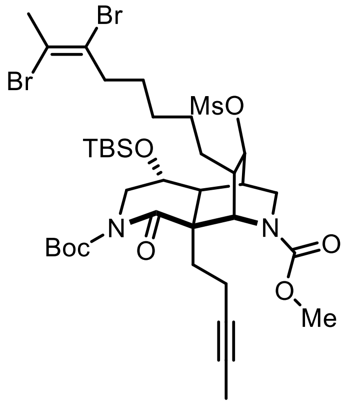

Compound 65. *Note: To assure reproducibility, the starting material should be stirred and dried under high vacuum for 2 d until it has turned into a fine power.*

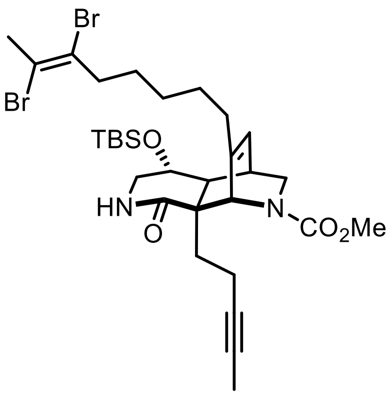

Mesylate **S13** (2.28 g, 2.54 mmol) was dissolved in 2,6-lutidine (12.7 mL) and the resulting solution was stirred at 170 °C (bath temperature) for 5 d. The mixture was then cooled to ambient temperature and diluted with CH_2Cl_2 (6.0 mL).

TBSOTf (2.9 mL, 12.6 mmol) was added to this solution at 0 °C. After 5 min, the cooling bath was removed and the mixture stirred at ambient temperature for 3h. sat. aq. $NaHCO_3$ (10 mL) was added at 0 °C, followed, after 5 min, by careful addition of HCl (2 M, 40 mL). After stirring for 10 min, the mixture was extracted with EtOAc (3 x 100 mL), the combined organic phases were washed with sat. aq. $NaHCO_3$ (5 mL) and dried with $MgSO_4$. After filtration and evaporation of the solvent in vacuum, the residue was purified by flash chromatography on silica gel (hexanes/EtOAc, 8:1 to 4:1) to afford the title product as a yellow solid (1.40 g, 78 %). $[\alpha]_D^{20}$ = 30.0 (c = 1.0, $CHCl_3$); ^{1}H NMR (400 MHz, $CDCl_3$, mixture of rotamers, ca. 2:1): δ = 5.93–5.82 (m, 1H), 5.80–5.65 (m, 1H), 4.87 (d, *J* = 1.6 Hz, 0.34H, minor), 4.75 (d, *J* = 1.5 Hz, 0.66H, major), 3.73 (s, 2H), 3.68 (s, 1H), 3.35 (td, *J* = 9.4, 4.7 Hz, 1H), 3.20–3.09 (m, 2H), 3.08–2.91 (m, 2H), 2.79–2.70 (m, 1H), 2.66–2.58 (m, 2H), 2.42–2.37 (m, 3H), 2.33 – 2.08 (m, 4H), 1.86 (dq, *J* = 10.7, 5.3 Hz, 2H), 1.74 (t, *J* = 2.5 Hz, 3H), 1.69–1.60 (m, 2H), 1.59–1.48 (m, 2H), 1.44–1.35 (m, 1H), 1.30–1.22 (m, 2H), 0.89 (s, 9H), 0.10 (s, 3H), 0.06 (s, 3H) ppm; ^{13}C NMR (101 MHz, $CDCl_3$): δ = 172.6, 156.0, 156.0, 148.4, 147.6, 124.3, 123.7, 122.1, 122.0, 115.2, 115.1, 79.0, 78.5, 75.7, 75.4, 70.8, 70.7, 54.3, 54.2, 52.9, 52.6, 52.5, 51.4, 51.3, 47.3, 47.1, 45.6, 40.6, 40.6, 39.9, 39.7, 33.6, 33.6, 33.3, 28.7, 28.0, 27.9, 27.2, 27.1, 27.0, 26.6, 26.4, 25.7, 17.8, 14.1, 3.5, 3.4, –4.3, –4.3, –4.8 ppm; IR (film) $\tilde{\nu}$ = 2950, 2928, 2857, 1699, 1664, 1446, 1386, 1339, 1299, 1273, 1254, 1216, 1190, 1120, 1107, 1064, 1006, 981, 955, 927, 876, 836, 814, 774, 708, 685, 660, 616 cm^{-1}; MS (ESI)*: m/z*: 699 [*M*+H$^+$], 721 [*M*+Na$^+$]; HRMS (ESI): *m/z:* calcd. for $C_{29}H_{46}N_5O_3SiBr_2Na$ [*M*+Na$^+$]: 721.16291, found: 721.16321.

Compound 67. NaH (254 mg, 10.6 mmol) was added to a solution of compound **65** (1.40 g, 1.99 mmol) and iodide **61** (0.75 mL, 2.38 mmol) in DMF/THF (10 mL, 1:1) at 0 °C. After stirring at this temperature for 1h, the mixture was poured into a solution of sat. aq. NH_4Cl (20 mL). The resulting mixture was extracted with EtOAc (3 x 50 mL), the combined organic phases were washed with brine, dried over $MgSO_4$ and filtered. After evaporation of the solvent in vacuum, the residue was purified by flash chromatography on silica gel (hexanes/*tert*-butyl methyl ether, 8:1 to 4:1) to afford product **66** as a colorless oil.

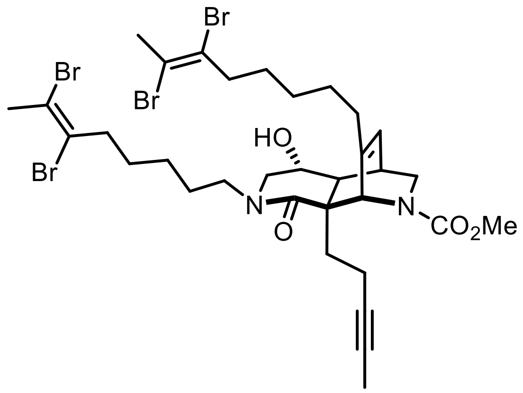

This compound was dissolved in THF (4.2 mL) and TBAF (1 M in THF, 4.0 mL, 4.0 mmol) was added. The resulting mixture was stirred at ambient temperature for 1 h before the reaction was quenched with sat. aq. NH_4Cl (5.0 mL). The resulting mixture was extracted with EtOAc (3 x 10 mL), the combined organic phases were washed with brine and dried over Na_2SO_4. After filtration and evaporation of the solvent in vacuum, the crude material was purified by flash chromatography on silica gel (hexanes/acetone, 15:1 to 4:1) to afford the title compound as a yellow oil (1.56 g, 92 %). $[\alpha]_D^{20}$ = 36.7 (c = 1.0, $CHCl_3$); ^{1}H NMR (400 MHz, $CDCl_3$, mixture of rotamers, ca. 2:1): δ = 5.92–5.77 (m, 1H), 4.89 (s, 0.4H, minor), 4.78 (s, 0.6H, major), 3.72 (s, 2H, major), 3.66 (s, 1H, minor), 3.44–3.23 (m, 3H), 3.21–3.09 (m, 3H), 3.03–2.82 (m, 2H), 2.70–2.56 (m, 5H), 2.43–2.36 (m, 6H), 2.31–2.04 (m, 4H), 1.92–1.81 (m, 1H), 1.73 (q, *J* = 2.8 Hz, 4H), 1.62–1.54 (m, 4H), 1.51–1.42 (m, 4H), 1.31–1.16 (m, 4H) ppm; ^{13}C NMR (101 MHz, $CDCl_3$): δ = 169.9, 169.9, 156.1, 156.1, 148.2, 147.4, 124.3, 123.7, 122.1, 121.9, 121.7, 121.7, 115.5, 115.4, 115.2, 115.1, 79.1, 78.6, 75.7, 75.5, 69.6, 69.5, 53.8, 53.8, 53.4, 53.1, 52.6, 52.5, 51.9, 51.8, 51.1, 47.6, 47.5, 47.3, 47.0, 40.6, 40.5, 40.3, 40.3, 39.8, 39.5, 33.7, 33.5, 33.4, 28.7, 28.0, 27.9, 27.2, 27.2, 27.1, 27.1, 27.0, 26.6, 26.4, 25.5, 25.4, 14.2, 3.6, 3.5 ppm; IR (film) $\tilde{\nu}$ = 3400, 2926, 2858, 1700, 1678, 1645, 1617, 1487, 1448, 1391, 1340, 1261, 1192, 1159, 1113, 1066, 971, 955, 816, 766, 714, 616, 582 cm^{-1}; MS (ESI)*: m/z*: 851 [*M*+H$^+$], 873 [*M*+Na$^+$]; HRMS (ESI): *m/z:* calcd. for $C_{33}H_{45}N_2O_4Br_4$ [*M*$^+$]: 849.01188, found: 849.01244.

Compound S14. Martin's sulfurane (1.48 g, 2.20 mmol) was added to a solution of compound **67** (1.18 g, 1.38 mmol) in toluene (7.0 mL) at 100 °C. After stirring at this temperature for 20 min, the mixture was cooled to ambient temperature and directly loaded on silica. The product was eluted with hexanes/EtOAc (8:1 to 4:1) to afford the title compound as a colorless oil (1.16 g, quant.). $[\alpha]_D^{20}$ = 15.9 (c = 1.0, $CHCl_3$); ^1H NMR (400 MHz, $CDCl_3$, mixture of rotamers, ca. 2:1): δ = 5.97 (td, *J* = 6.4, 3.2 Hz, 1H), 5.72 (dd, *J* = 8.1, 1.3 Hz, 1H), 4.96–4.76 (m, 2H), 3.74 (s, 2H, major), 3.69 (s, 1H, minor), 3.50 (dddd, *J* = 13.8, 7.9, 6.0, 2.8 Hz, 1H), 3.27–3.18 (m, 1H), 3.09 (dddd, *J* = 13.3, 8.2, 6.7, 3.3 Hz, 1H), 2.84 (ddd, *J* = 25.0, 10.1, 2.4 Hz, 1H), 2.69–2.53 (m, 5H), 2.43–2.38 (m, 6H), 2.32–2.25 (m, 1H), 2.22–1.97 (m, 5H), 1.74 (q, *J* = 2.6 Hz, 3H), 1.65–1.42 (m, 8H), 1.39–1.19 (m, 5H) ppm; ^{13}C NMR (101 MHz, $CDCl_3$): δ = 168.5, 156.3, 156.2, 147.5, 146.7, 127.9, 127.7, 125.7, 125.1, 122.1, 122.0, 121.7,121.7, 115.6, 115.5, 115.3, 106.9, 106.6, 78.6, 78.5, 75.7, 75.6, 56.2, 55.9, 53.2, 52.6, 52.5, 48.2, 47.1, 44.0, 44.0, 41.0, 40.6, 40.5, 40.4, 40.4, 37.6, 37.3, 34.0, 28.8, 28.8, 28.1, 28.1, 27.9, 27.9, 27.2, 27.1, 27.1, 27.0, 26.6, 25.5, 25.4, 15.0, 3.5 ppm; IR (film) $\tilde{\nu}$ = 2927, 2858, 1700, 1648, 1447, 1390, 1414, 1338, 1274, 1257, 1232, 1191, 1152, 1107, 1067, 973, 951, 847, 766, 730, 702, 617, 590 cm^{-1}; MS (ESI): *m/z*: 833 [*M*+H^+], 855 [*M*+Na^+]; HRMS (ESI): *m/z:* calcd. for $C_{33}H_{44}N_2O_3Br_4Na$ [*M*+Na^+]: 854.99781, found: 854.99776.

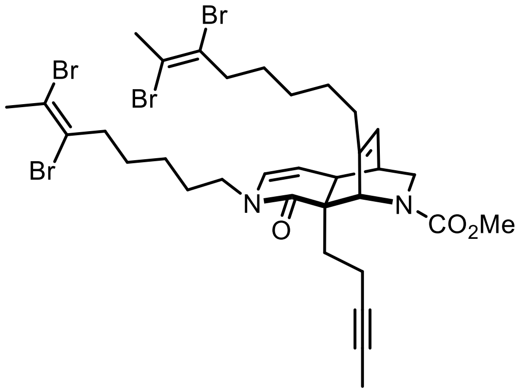

Compound 68. $NaBH_3CN$ (368 mg, 5.86 mmol) was added to a solution of compound **S14** (1.0 g, 1.20 mmol) in CH_2Cl_2 (60 m) at 0 °C. TFA (0.91mL, 11.9 mmol) was slowly added at 0 °C. After stirring for 10 min, cooling bath was removed and the mixture stirred at ambient temperature for 50 min before the reaction was quenched with sat. $NaHCO_3$ (5.0 mL). [*Note: the reaction is seriously time-dependent: any longer reaction time will cause a sharp decrease in yield*]

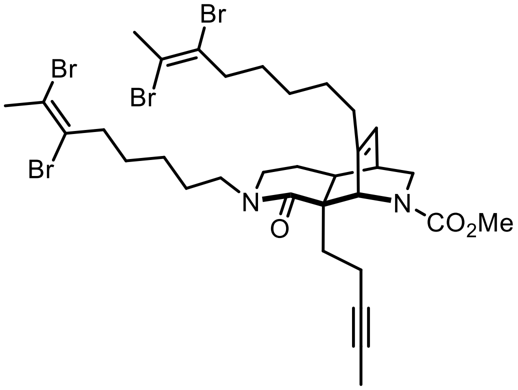

The resulting mixture was extracted with CH_2Cl_2 (3 x 300 mL), the combined organic phases were washed with brine, dried over Na_2SO_4, and filtered. After evaporation of the solvent in vacuum, the residue was purified by flash chromatography on silica (hexanes/EtOAc, 8:1 to 4:1) to afford the title product as a colorless oil (664 mg, 66 %) . $[\alpha]_D^{20}$ = 30.8 (c =

1.0, $CHCl_3$); ^{1}H NMR (400 MHz, $CDCl_3$, mixture of rotamers, ca. 2:1): δ = 5.85 (t, *J* = 7.9 Hz, 1H), 4.86 (d, *J* = 1.5 Hz, 0.35H, minor), 4.75 (d, *J* = 1.5 Hz, 0.65H, major), 3.73 (s, 2H), 3.68 (s, 1H), 3.41–3.30 (m, 1H), 3.28–3.14 (m, 3H), 3.13–3.04 (m, 1H), 2.91 (ddd, *J* = 29.8, 10.1, 2.6 Hz, 1H), 2.69–2.58 (m, 4H), 2.55–2.45 (m, 1H), 2.41 (dq, *J* = 2.8, 1.8, 1.4 Hz, 6H), 2.31–2.15 (m, 2H), 2.12–2.02 (m, 2H), 2.00–1.89 (m, 2H), 1.81 (dd, *J* = 9.1, 6.8 Hz, 1H), 1.74 (t, *J* = 2.5 Hz, 3H), 1.69–1.60 (m, 2H), 1.56–1.32 (m, 8H), 1.31–1.22 (m, 4H) ppm; ^{13}C NMR (101 MHz, $CDCl_3$): δ = 170.4, 170.3, 156.2, 156.2, 147.3, 146.6, 124.1, 123.5, 122.2, 122.0, 121.8, 121.8, 121.8, 115.4, 115.4, 115.2, 79.3, 78.9, 75.4, 75.3, 54.3, 54.2, 52.6, 52.4, 52.0, 51.9, 48.2, 48.0, 47.8, 47.7, 45.2, 45.1, 44.9, 44.9, 40.6, 40.6, 40.4, 40.4, 39.5, 39.4, 37.2, 36.8, 33.7, 33.6, 29.8, 28.8, 28.0, 27.9, 27.3, 27.2, 27.2, 27.1, 27.1, 27.0, 26.6, 26.4, 25.6, 25.6, 14.5, 14.4, 3.6, 3.5 ppm; IR (film) $\tilde{\nu}$ = 2928, 2858, 1699, 1634, 1487, 1447, 1389, 1338, 1275, 1231, 1210, 1190, 1159, 1110, 1068, 970, 767, 616 cm^{-1}; MS (ESI)*: m/z*: 835 [*M*+H$^+$], 857 [*M*+Na$^+$]; HRMS (ESI): *m/z:* calcd. for $C_{33}H_{46}N_2O_3Br_4Na$ [*M*+Na$^+$]: 857.01346, found: 857.01264.

Compound 69. A solution of TMSI (0.13 mL, 0.914 mmol) in CH_2Cl_2 (2.0 mL) was added to a solution of compound **68** (700 mg, 0.835 mmol) in CH_2Cl_2 (17 mL). The resulting mixture was stirred for 1 d at ambient temperature before the reaction was quenched with MeOH (2.0 mL) and sat. aq. $NaHCO_3$ (5.0 mL) at 0 °C. After removal of the solvents, the crude mixture was loaded on an amino cartridge (Agilent, Bond Elut-NH_2, 500 mg, 3 mL, 40 μm, pre-equilibrated with MeOH, H_2O, MeOH (volume of ca. one column length each)) and the amine product was eluted with MeOH to provide a white solid [*purification on silica gel with basic elute gave much lower yields*].

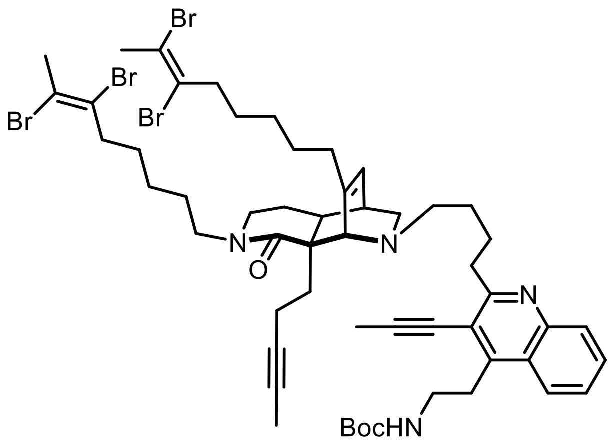

A solution of aldehyde **52** (476 mg, 1.25 mmol) in CH_2Cl_2 (3 mL) was added to a solution of the amine in CH_2Cl_2 (2 mL). After stirring for 10 min at ambient temperature, $NaBH(OAc)_3$ (230 mg, 1.09 mmol) was added and stirring was continued for 1 h. The reaction was quenched with sat. aq. $NaHCO_3$ (0.5 mL). After removing the solvent in high vacuum, the crude material was subjected to preparative HPLC (Kromasil-5-C18, 5 μm, 150 mm × 30 mm, MeOH, 35 mL/min, λ = 220 nm, t = 9.2 min) to afford the title compound as a brownish solid (642 mg, 67 %). $[\alpha]_D^{20}$ = –15.0 (c = 1.2, $CHCl_3$); ^{1}H NMR (400 MHz, $CDCl_3$): δ = 7.96 (d, *J* = 8.4 Hz, 1H), 7.91 (dd, *J* = 8.5, 1.2 Hz, 1H), 7.54 (ddd, *J* = 8.4, 6.9, 1.4 Hz, 1H), 7.40 (ddd, *J* = 8.3, 6.8, 1.3 Hz,

1H), 5.74 (d, *J* = 6.4 Hz, 1H), 4.64 (s, 1H), 3.40 (d, *J* = 3.2 Hz, 4H), 3.30 (s, 1H), 3.23 (dt, *J* = 13.2, 7.4 Hz, 1H), 3.07 (dt, *J* = 16.1, 7.4 Hz, 4H), 3.00–2.84 (m, 2H), 2.62–2.48 (m, 4H), 2.45–2.34 (m, 1H), 2.31 (dd, *J* = 9.2, 1.0 Hz, 6H), 2.15 (s, 5H), 2.08–1.86 (m, 5H), 1.85–1.67 (m, 4H), 1.60 (d, *J* = 8.3 Hz, 2H), 1.57 (d, *J* = 2.4 Hz, 3H), 1.47 (h, *J* = 7.5 Hz, 6H), 1.39–1.32 (m, 12H), 1.29–1.14 (m, 6H) ppm; ^{13}C NMR (101 MHz, $CDCl_3$): δ = 171.8, 163.2, 155.9, 147.0, 146.1, 144.6, 129.4, 129.2, 126.2, 125.7, 123.8, 122.4, 122.1, 121.9, 117.7, 115.4, 115.1, 95.6, 79.8, 79.2, 76.1, 74.9, 62.5, 58.0, 55.6, 52.2, 47.7, 45.3, 44.1, 40.6, 40.4, 39.1, 37.9, 35.0, 30.8, 29.7, 28.8, 28.8, 28.4, 28.4, 27.4, 27.2, 27.2, 26.8, 26.4, 25.7, 14.7, 4.9, 3.5 ppm; IR (film) $\tilde{\nu}$ = 3328, 2928, 2857, 1708, 1628, 1453, 1251, 1171, 759 cm^{-1}; MS (ESI)*: m/z*: 1141 [*M*+H$^+$], 1163 [*M*+Na$^+$]; HRMS (ESI): *m/z:* calcd. for $C_{54}H_{73}Br_4N_4O_3$ [*M*+H$^+$]: 1141.24112, found: 1141.24199.

Compound 70. A flame-dried two-necked flask connected to a reflux condenser was charged with activated molecular sieve powder (5 Å, 1.5 g) and toluene (20 mL). The suspension was purged with argon at room temperature for 30 min. The mixture was then heated to 110 °C for 30 min and a solution of diyne **69** (50.6 mg, 0.044 mmol) in toluene (2 mL) was added. Next, a solution of the Mo-catalyst **31** (9.8 mg, 0.013 mmol)[3] in toluene (0.5 mL) was added dropwise and stirring was continued at 110 °C for 15 min. Ethanol (5 mL) was added to quench the reaction. The mixture was cooled to room temperature and filtered through a plug of Celite, which was carefully rinsed with EtOAc. The combined filtrates were evaporated *in vacuo* and the residue was purified by preparative HPLC (Kromasil-5-C18, 5 µm, 150 mm × 30 mm, MeOH, 35 mL/min, λ = 254 nm, t = 11.5 min) to afford the title compound a white solid (37.1 mg, 77%) as. $[\alpha]_D^{20}$ = –30.9 (c = 1.0, $CHCl_3$); ^{1}H NMR (400 MHz, $CDCl_3$): δ = 8.06 (d, *J* = 8.4 Hz, 1H), 7.99 (dd, *J* = 8.5, 1.2 Hz, 1H), 7.62 (ddd, *J* = 8.3, 6.9, 1.3 Hz, 1H), 7.49 (ddd, *J* = 8.3, 6.8, 1.3 Hz, 1H), 5.84 (dd, *J* = 6.4, 1.8 Hz, 1H), 4.82 (s, 1H), 3.72 (d, *J* = 1.6 Hz, 1H), 3.64–3.41 (m, 7H), 3.29 (td, *J* = 12.4, 2.1 Hz, 1H), 3.09–2.97 (m, 3H), 2.93 (dd, *J* = 9.3, 2.1 Hz, 1H), 2.81 (td, *J* = 11.9, 2.4 Hz, 1H), 2.74–2.56 (m, 6H), 2.55–2.45 (m, 1H), 2.43 (d, *J* = 0.9 Hz, 3H), 2.41 (d, *J* = 0.9 Hz, 3H), 2.33–2.19 (m, 4H), 2.17–2.08 (m, 1H), 1.96 (ddd, *J* = 13.7, 10.4, 5.6 Hz, 1H), 1.86 (ddd, *J* = 13.4, 5.6, 2.4 Hz, 1H), 1.75–1.66 (m, 3H), 1.58 (dtd, *J* = 16.2, 9.1, 8.2, 3.2 Hz, 7H), 1.48 (dd, *J* = 7.9, 2.1 Hz, 2H), 1.43 (s, 9H), 1.37 (qt, *J* = 7.2, 4.0 Hz, 2H), 1.32–1.21 (m, 3H) ppm; ^{13}C NMR (101 MHz, $CDCl_3$): δ = 171.9, 164.3, 156.0, 146.3, 146.1, 145.5, 129.2, 129.1, 126.2, 125.8, 123.9, 122.2, 121.9, 117.8, 115.5, 115.1, 102.2, 79.1, 76.3, 60.8, 55.3, 54.3, 52.5, 47.6, 46.9, 45.3, 40.7, 40.5, 40.4, 38.6, 37.5, 37.0, 35.0, 30.8, 30.4, 28.8, 28.8, 28.4,

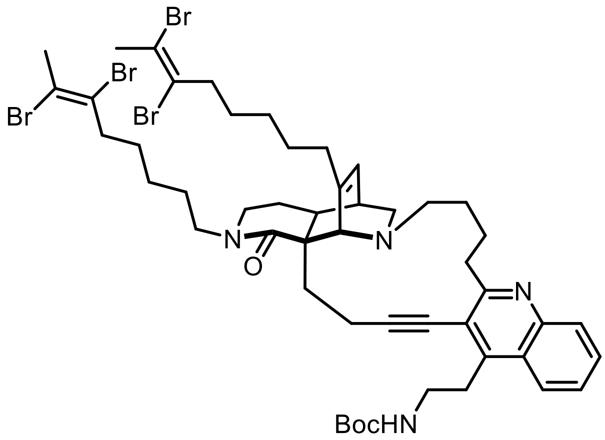

28.4, 27.8, 27.4, 27.4, 27.2, 26.7, 25.5, 25.1, 13.5 ppm; IR (film) $\tilde{\nu}$ = 3339, 2928, 2855, 1705, 1630, 1450, 1169, 1070, 756, 617 cm^{-1}; MS (ESI): *m/z*: 1086 [*M*+H$^+$]; HRMS (ESI): *m/z:* calcd. for $C_{50}H_{67}Br_4N_4O_3$ [*M*+H$^+$]: 1087.19417, found: 1087.19495.

Table S8. RCAM of Diyne **69**: Reaction Optimization[a]

Entry	Catalyst	Conversion	Yield of 70
1	**31** (10 mol%)	≈ 50% (NMR)	n. d.
2	**31** (30 mol%)	quant.	77%
3	**29** (30 mol%) + **30** (30 mol%)	quant.	77%

[a] All reactions were performed in toluene at reflux temperature in the presence of MS 5Å

Compound S15. Pd/$CaCO_3$ (5 mol% *w/w*, *unpoisoned*, 704 mg, 0.331 mmol) was added to solution of compound **70** (180 mg, 0.165mmol) in THF (18 mL) at ambient temperature. After stirring for 2 h, the suspension was filtered through a pad of Celite® and the filtrate was concentrated. The crude product was subjected to purification by preparative HPLC (Kromasil-5-C18, 5 µm, 150 mm × 30 mm, MeOH, 35 mL/min, λ = 254 nm, t = 8.6 min) to afford the title compound as a white solid (94.0 mg, 52%). $[\alpha]_D^{20}$ = 4.8 (c = 0.24, $CHCl_3$); ^{1}H NMR (600 MHz, $CDCl_3$): δ = 8.08–8.00 (m, 2H), 7.65 (t, *J* = 7.0 Hz, 1H), 7.54 (s, 1H), 6.41 (d, *J* = 11.0 Hz, 1H), 5.88–5.81 (m, 1H), 5.79–5.74 (m, 1H), 4.56 (s, 1H), 3.41 (d, *J* = 9.2 Hz, 1H), 3.33–3.10 (m, 9H), 3.02–2.87 (m, 3H), 2.62 (t, *J* = 7.5 Hz, 2H), 2.52 (t, *J* = 7.2 Hz, 2H), 2.41–2.40 (m, 3H), 2.37 (s, 3H), 2.34–2.26 (m, 2H), 2.21 (t, *J* = 12.7 Hz, 1H), 2.15–2.13 (m, 1H), 1.99–1.88 (m, 3H), 1.86–1.73 (m, 3H), 1.71–1.64 (m, 2H), 1.61 (d, *J* = 11.5 Hz, 1H), 1.60–1.52 (m, 3H), 1.50–1.37 (m, 13H), 1.33–1.21 (m, 4H), 1.20 – 1.11 (m, 4H), 1.07 (d, *J* = 13.0 Hz, 1H) ppm; ^{13}C NMR (151 MHz, $CDCl_3$): δ = 171.7, 159.9, 155.8, 146.7, 145.2, 135.7, 130.3, 129.3, 128.6, 126.2, 125.9, 124.5, 124.2, 123.1, 122.2, 121.9, 115.4, 115.0, 79.1, 56.9, 55.5, 54.8, 52.5, 47.4, 46.6, 45.2, 41.6, 40.5, 40.4, 38.5, 37.3, 37.0, 35.1, 30.7, 28.8, 28.7, 28.4, 28.1, 27.3, 27.3, 27.1, 26.5, 26.3, 25.6, 25.2, 24.4 ppm; IR (film) $\tilde{\nu}$ = 2959, 2852, 1253, 1116, 1082, 869, 612 cm^{-1}; MS (ESI): *m/z*: 1089 [*M*+H$^+$]; HRMS (ESI): *m/z:* calcd. for $C_{50}H_{69}Br_4N_4O_3$ [*M*+H$^+$]: 1089.21116, found: 1089.21061.

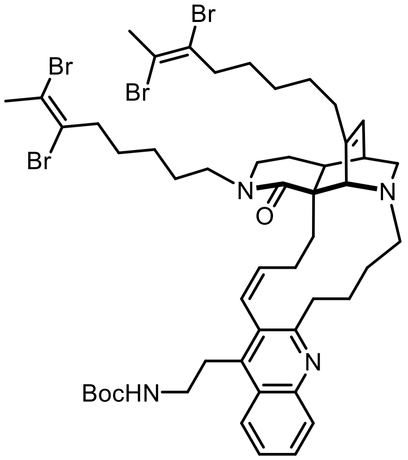

Compound 59. DIBAL-H (1.0 M in hexane, 0.4 mL, 0.40 mmol) was added to a solution of **S15** (64.0 mg, 0.0586 mmol) in Et_2O (0.4 mL) at 0 °C. After 5 min, the cooling bath was remved and the mixture stirred at 20 °C for 80 min [*Note: The reaction time should be strictly followed; longer reaction times will result in serious over-reduction of the vicinal dibromide*].

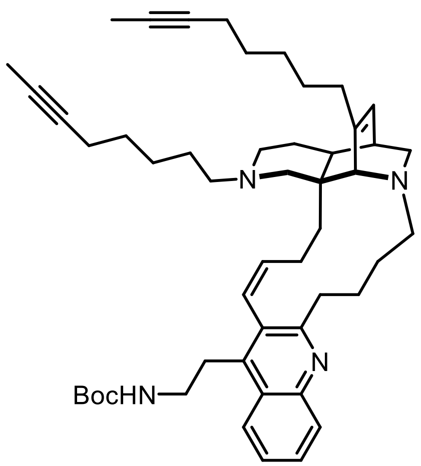

The mixture was diluted with *tert*-butyl methyl ether (2.0 mL) at 0 °C and the reaction quenched with sat. Rochelle's salt solution (0.4 mL). The resulting mixture was vigorously stirred for 5 h. DDQ (13.3 mg/mL) was added to the mixture until the color became brown. The mixture was then filtered through a cartridge (Agilent, Bond Elut-NH_2, 500 mg, 3 mL, 40 µm, pre-equilibrated with of MeOH, H_2O, MeOH (volume of ca. one column length each)), eluting with MeOH. Evaporation of the solvent provided a white solid which was subjected to preparative HPLC (Kromasil-5-C18, 5 µm, 150 mm × 30 mm, MeOH/20 mmol NH_4HCO_3 PH 9 = 98:2, 35 mL/min, λ = 254 nm, t = 29 min) to provide the corresponding amine product as a yellow solid material.

Zn powder (44.0 mg, 0.673 mmol) was added to a solution of this compound in THF/HOAc (1.05 mL, 20:1) at ambient temperature. The mixture was stirred for 1 h before the reaction was carefully quenched with sat. aq. $NaHCO_3$ (0.2 mL). The resulting mixture was passed through a cartridge (Agilent, Bond Elut-NH_2, 500 mg, 3 mL, 40 µm (pre-equilibrated with MeOH, H_2O, MeOH (volume of one column length each); the product was eluted with MeOH to provide a white solid after evaporation of the solvent. The crude material was subjected to preparative HPLC (Kromasil-5-C18, 5 µm, 150 mm × 30 mm, MeOH/ 20 mmol NH_4HCO_3 pH 9 = 98:2, 35 mL/min, λ = 254 nm, t = 7.8 min) to afford the title compound as a white solid (19.7 mg, 44 %). $[\alpha]_D^{20}$ = 210 (c = 0.4, $CHCl_3$); ^1H NMR (400 MHz, $CDCl_3$): δ = 8.21–8.13 (m, 1H), 8.06 (dd, *J* = 8.4, 1.3 Hz, 1H), 7.67 (ddd, *J* = 8.3, 6.8, 1.3 Hz, 1H), 7.55 (t, *J* = 7.6 Hz, 1H), 6.44 (d, *J* = 10.9 Hz, 1H), 5.94 (t, *J* = 10.9 Hz, 1H), 5.79–5.69 (m, 1H), 4.62 (s, 1H), 3.44–3.16 (m, 5H), 2.99 (d, *J* = 8.7 Hz, 1H), 2.88 (td, *J* = 12.9, 4.6 Hz, 1H), 2.82–2.74 (m, 1H), 2.45–2.22 (m, 6H), 2.12 (tt, *J* = 7.1, 2.6 Hz, 2H), 2.10–2.00 (m, 4H), 1.99–1.89 (m, 2H), 1.81 (s, 2H), 1.78 (t, *J* = 2.5 Hz, 4H), 1.74 (t, *J* = 2.5 Hz, 4H), 1.70–1.64 (m, 4H), 1.44 (s, 9H), 1.39–1.31 (m, 9H), 1.30–1.18 (m, 6H), 1.00 (dd, *J* = 12.0, 5.9 Hz, 2H) ppm; ^{13}C NMR (101 MHz, $CDCl_3$): δ = 160.5, 155.8, 146.8, 142.9, 136.4, 130.6, 129.7, 128.4, 126.2, 125.8, 124.1, 123.5, 121.6, 79.3, 79.3, 75.5, 75.3, 59.3, 57.1, 56.2, 55.8, 50.2, 49.8, 45.8, 42.9, 40.4, 38.7, 37.7, 36.5, 36.3, 29.0, 28.9, 28.5, 28.4, 27.3, 27.0, 26.8, 26.3, 26.0, 25.4, 23.7, 18.8, 18.6, 3.5, 3.4 ppm; IR (film) $\tilde{\nu}$ = 3315, 2930, 1562, 1406, 1023,

762, 649 cm^{-1}; MS (ESI): *m/z*: 759 [*M*+H^+]; HRMS (ESI): *m/z:* calcd. for $C_{50}H_{71}N_2O_4$ [*M*+H^+]: 759.55715, found: 759.55745.

Compound 71. A flame-dried two-necked flask connected to a reflux condenser was charged with activated powdered molecular sieves (5 Å, 200 mg) and toluene (4 mL). The suspension was purged with argon at room temperature for 15 min. After the purging had been stopped, the mixture was heated to 110 °C for 30 min before a solution of diyne **59** (7 mg, 0.009 mmol) in toluene (0.5 mL) was added, followed by dropwise addition of a solution of the Mo-complex **31** (2.0 mg, 0.003 mmol)[3] in toluene (0.4 mL). The resulting suspension was stirred at 110 °C for 20 min. Ethanol (1 mL) was added to quench the reaction and the crude mixture was cooled to room temperature and filtered through a plug of Celite, which was carefully rinsed with EtOAc. The solvent was evaporated in vacuo and the crude product was purified by preparative HPLC (Kromasil-5-C18, 5 µm, 150 mm × 30 mm, MeOH/20 mmol NH_4HCO_3 pH 9 = 98:2, 35 mL/min, λ = 230 nm, t = 7.6 min) to afford the title compound as a white solid (6.4 mg, 98%). $[\alpha]_D^{20}$ = 23.0 (c = 0.3, $CHCl_3$); ^{1}H NMR (400 MHz, $CDCl_3$): δ = 8.17 (d, *J* = 8.4 Hz, 1H), 8.05 (dd, *J* = 8.4, 1.2 Hz, 1H), 7.68–7.63 (m, 1H), 7.55 (t, *J* = 7.6 Hz, 1H), 6.43 (dd, *J* = 10.9, 2.2 Hz, 1H), 5.95 (t, *J* = 10.7 Hz, 1H), 5.77 (d, *J* = 6.5 Hz, 1H), 4.57 (s, 1H), 3.36 (d, *J* = 17.3 Hz, 2H), 3.23 (pd, *J* = 9.2, 4.2 Hz, 3H), 3.00 (dd, *J* = 8.8, 2.2 Hz, 1H), 2.86 (ddd, *J* = 24.4, 12.3, 3.6 Hz, 2H), 2.55 (d, *J* = 10.9 Hz, 2H), 2.42 (d, *J* = 12.7 Hz, 1H), 2.37–2.25 (m, 3H), 2.17–2.05 (m, 5H), 2.03–1.97 (m, 3H), 1.96–1.85 (m, 3H), 1.82–1.62 (m, 5H), 1.52–1.39 (m, 15H), 1.38–1.26 (m, 8H), 1.26–1.13 (m, 1H), 1.07–0.93 (m, 2H) ppm; ^{13}C NMR (101 MHz, $CDCl_3$): δ = 160.5, 155.8, 146.8, 142.9, 136.3, 130.6, 129.7, 128.4, 126.3, 125.8, 124.2, 123.6, 120.5, 80.6, 80.2, 79.2, 59.4, 57.3, 56.8, 55.8, 49.6, 49.3, 45.6, 42.9, 40.4, 38.7, 37.5, 36.8, 36.4, 29.0, 28.4, 27.9, 27.7, 27.5, 27.4, 27.1, 26.4, 25.4, 24.6, 23.7, 18.2, 17.8 ppm; IR (film) $\tilde{\nu}$ = 2926, 2857, 1703, 1455, 1365, 1171, 758, 678 cm^{-1}; MS (ESI): *m/z*: 705 [*M*+H^+]; HRMS (ESI): *m/z:* calcd. for $C_{46}H_{65}N_4O_2$ [*M*+H^+]: 705.51020, found: 705.51087.

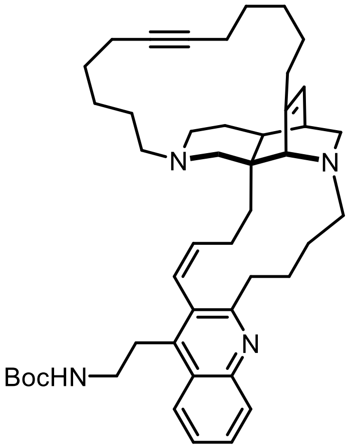

Nominal Njaoamine I ((+)-4). HCl (0.48 mmol, 120 μL, 4 M in 1,4-dioxane) was added dropwise to a solution of compound **71** (9.0 mg, 12.8 μmol) in EtOAc (0.42 mL) and H_2O (80 μL) at 0 °C. The resulting solution was stirred for 2 h at this temperature. The solvent was evaporated in high vacuum to provide the HCl salt of njaoamine I. The HCl salt was passed through an amino cartridge (pre-equilibrated with MeOH, H_2O, MeOH (three volumes of three column length each)), eluting the product with MeOH. After evaporation of the solvent, the free amine was subjected to preparative HPLC (150 mm YMC Triart C18 5 µm, 10.0 mm i.D., Methanol/0.1% TFA in H_2O = 55:45, 4.7 mL/min, λ = 220 nm, t = 1.6 min) to afford the title compound as a white solid (8.6 mg, quant.). $[\alpha]_D^{20}$ = 69.3 (c = 0.2, $CHCl_3$); for the ^1H NMR and ^{13}C NMR data, see Table S9; IR (film) ν = 2936, 1677, 1202, 1182, 1133, 938, 761, 708 cm^{-1}; MS (ESI): *m/z*: 605 [M+H^+]; HRMS (ESI): *m/z:* calcd. for $C_{41}H_{57}N_4$ [M+H^+]: 605.45777, found: 605.45765.

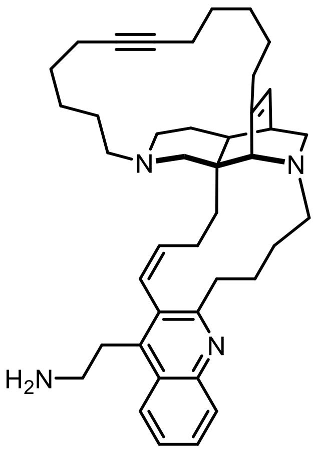

NMR Assignment and Structure Confirmation of Synthetic Nominal Njaomine I ((+)-4)

The complete assignment of synthetic njaoamine I (+)-**4** was achieved on a sample containing 2.6 mg of the material dissolved in [D5]-pyridine inside a 3-mm tube, to meet the conditions used in ref. [10]. All measurements were performed on a Bruker Avance III 600 spectrometer equipped with a cryogenically cooled 5 mm TCI probehead. Using a classical set of 1D (^{1}H, ^{13}C) and 2D (^{13}C-HSQC, HMBC, COSY, TOCSY, ROESY and ^{15}N-HMBC) experiments, as well as 1D ^{1}H selective TOCSY and ^{13}C-HSQC-TOCSY for fragment C, all nuclei could be assigned including the stereotopic ^{1}H in the tricyclic core. Figure S4 shows the key correlations ($^{3}J_{HH}$(COSY), $^{3}J_{CH}$(HMBC) and NOEs(ROESY) in the three fragments A, B and C (following the fragmentation used in ref. [10]).

Fragment A:

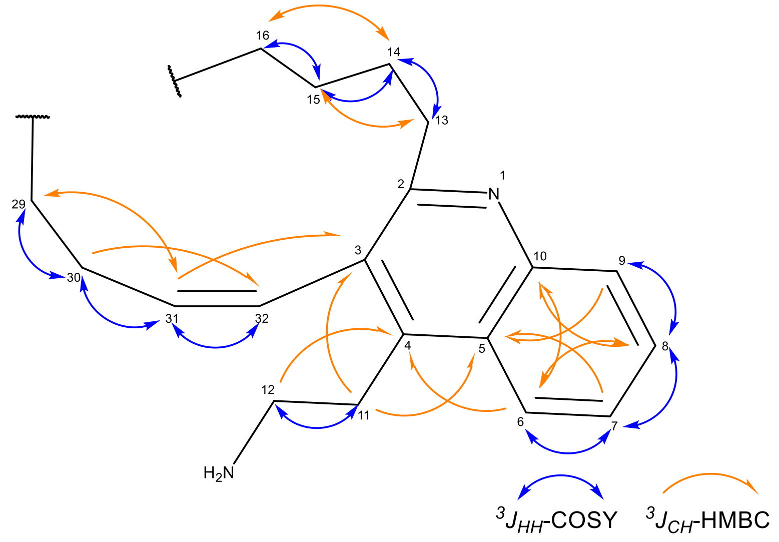

Fragment B:

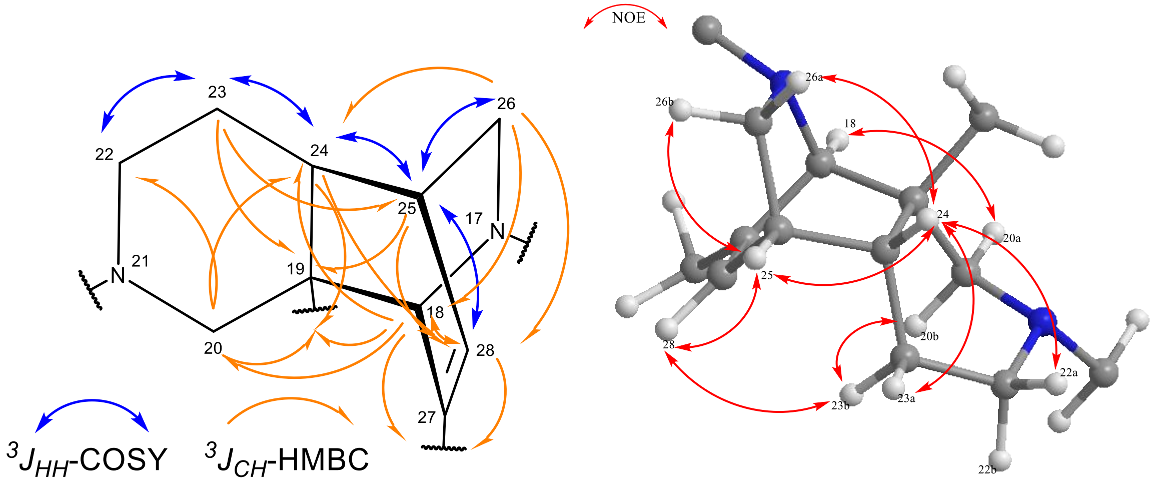

Fragment C:

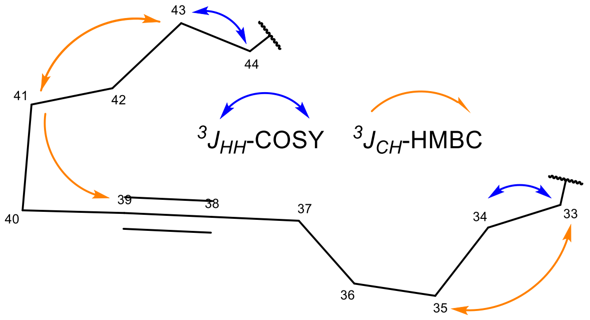

Figure S4. Summary of the 2- and 3-bond connectivities established by NMR for all three fragments. The important through-space NOE contacts in the tricyclic core are also displayed for fragment B.

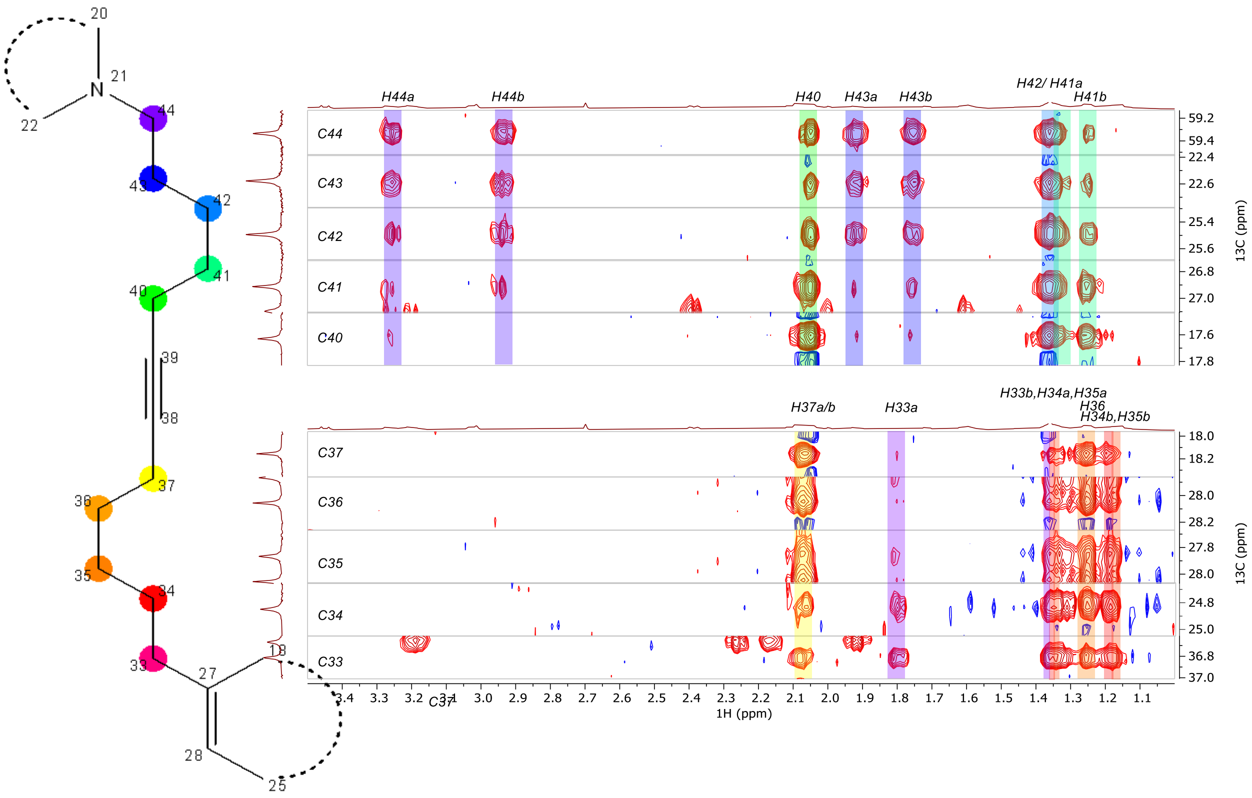

Figure S5. HSQC-TOCSY strips in from the carbons in the C12-chain (fragment C) showing TOCSY correlations to the corresponding ^{1}H-spin-system.

Due to important overlap and mixed phase nature of their cross-peaks, the COSY and HMBC data were particularly difficult to interpret for Fragment C (C12-chain). However, a clear assignment could still be achieved based on selective TOCSY and HSQC-TOCSY experiments. In Figure S5, each strip from the HSQC-TOCSY corresponds to the sequential ^{13}C in the C_{12}-chain (C33-C44) and contains cross-peak correlations to all ^{1}H within the uninterrupted CH_2 spin-system and with intensities roughly inversely related to their distance.

Table S9. Summary of all chemical shifts and correlations for the synthetic nominal njaoamine I (**(+)-4**)

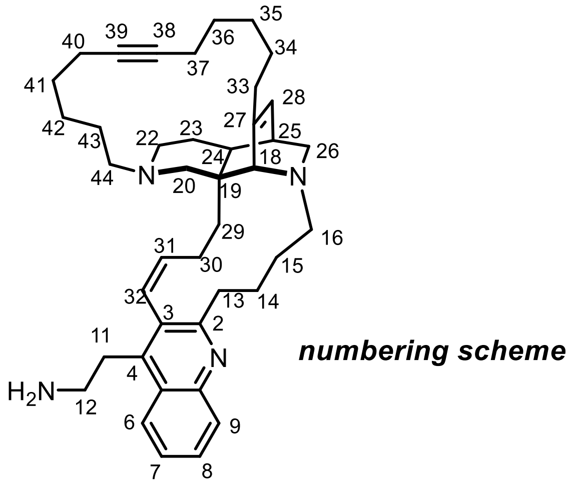

Njaoamine I

Atom	δ (ppm)	J	COSY	HSQC	HMBC	NOESY
**1 N**						
**2 C**	160.59				13a, 13b	
**3 C**	131.26				11a, 11b, 13b, 31	
**4 C**	141.84				6, 11a, 11b	
**5 C**	126.69				6, 7, 9, 11a, 11b	
**6 C**	124.96			6	8	
**H**	8.25	8.3(7)	7	6	4, 5, 8, 10	7, 11a, 11b, 12b
**7 C**	126.88			7	9	
**H**	7.44	6.8(8), 8.3(6)	6, 8	7	5, 9	6
**8 C**	129.34			8	6	
**H**	7.63	8.3(9), 6.8(7)	7, 9	8	6, 10	9
**9 C**	130.35			9	7	
**H**	8.31	8.3(8)	8	9	5, 7	8
**10 C**	147.78				6, 8	
**11 C**	28.44			11a, 11b	12a, 12b	
**Ha**	3.85	12.3(12a), 5.6(12b), 12.3(11b)	11b, 12a, 12b	11	3, 4, 5, 12	6, 11b
**Hb**	3.68	12.3(11a), 4.8(12a), 12.2(12b)	11a, 12a, 12b	11	3, 4, 5	6, 11a, 32
**12 C**	39.72			12a, 12b	11a	

**Ha**	3.59	12.2(12b), 12.3(11a), 4.8(11b)	11a, 11b	12	11	
**Hb**	3.53	12.2(12a), 5.6(11a), 12.2(11b)	11a, 11b	12	11	6
**13 C**	39.14			13a, 13b		
**Ha**	3.26	12.9(13b), 12.8(14?), 4.8(14?)	13b, 14a	13	2, 15	32
**Hb**	3.14	12.9(13a)	13a, 14a, 14b	13	2, 3, 14	
**14 C**	26.29			14a, 14b	13b, 16a	
**Ha**	2.42		13a, 13b, 14b, 15a, 15b	14		14b
**Hb**	1.60		13b, 14a	14		14a, 16a
**15 C**	27.32			15a, 15b	13a	
**Ha**	1.52		14a, 16a	15		
**Hb**	1.43		14a, 16a, 16b	15		18
**16 C**	56.51			16a, 16b	18, 26b	
**Ha**	2.42	12.7(16b)	15a, 15b, 16b	16	14	14b, 16b, 18
**Hb**	2.02	12.7(16a), 12.7(15?), 3.1(15?)	15b, 16a	16		16a
**17 N**						
**18 C**	57.09			18	26a, 28, 29b	
**H**	2.72			18	16, 19, 20, 24, 25, 26, 27, 28, 29, 33	15b, 16a, 20a, 29b, 33b
**19 C**	44.12				18, 20a, 23a, 25, 29a	
**20 C**	49.84			20a, 20b	18, 29a, 44a	
**Ha**	3.47	12.4(20b)	20b	20	19, 22, 24	18, 20b
**Hb**	2.19	12.4(20a)	20a	20	29, 44	20a, 23b
**21 N**	-353.20		22b			
**22 C**	49.25			22a, 22b	20a, 44a	
**Ha**	3.59		22b, 23b	22		22b, 24
**Hb**	3.07		22a, 21	22		22a, 23b
**23 C**	24.46			23a, 23b	24	
**Ha**	1.60		23b, 24	23	19	23b, 24
**Hb**	1.17		22a, 23a	23	25	20b, 22b, 23a, 28
**24 C**	41.79			24	18, 20a, 26a, 26b	
**H**	1.17		23a	24	23, 25, 28, 29	22a, 23a, 25, 26a, 29a
**25 C**	37.20			25	18, 23b, 24, 26a, 26b, 28	
**H**	2.12	6.5(28), 2.3(26b)	26a, 26b, 28	25	19, 27, 28	24, 26a, 26b, 28
**26 C**	57.37			26a, 26b	18	
**Ha**	3.07	9.0(26b)	25, 26b	26	18, 24, 25, 28	24, 25, 26b
**Hb**	1.75	9.0(26a), 2.3(25)	25, 26a	26	16, 24, 25, 28	25, 26a
**27 C**	143.16				18, 25	
**28 C**	122.12			28	18, 24, 25, 26a, 26b	
**H**	5.84	6.5(25)	25	28	18, 25, 33	23b, 25, 34a, 34b

**29 C**	36.64			29a, 29b	18, 20b, 24, 31	
**Ha**	2.33	12.4(29b), 12.4(15?)	29b, 30a, 30b	29	19, 20, 30, 31	24, 29b, 30b
**Hb**	1.96	12.4(29a)	29a, 30a	29	18, 30, 31	18, 29a
**30 C**	24.04			30a, 30b	29a, 29b, 31, 32	
**Ha**	2.83		29a, 29b, 30b	30		31
**Hb**	1.96		29a, 30a, 31	30	31, 32	29a, 31
**31 C**	136.89			31	29a, 29b, 30b	
**H**	6.07	11.1(32)	30b, 32	31	3, 29, 30	30a, 30b
**32 C**	124.94			32	30b	
**H**	6.46	11.1(31)	31	32	30	11b, 13a
**33 C**	36.74			33a, 33b	18, 28	
**Ha**	1.83		33b	33		
**Hb**	1.40		33a	33	34, 35	18
**34 C**	24.98			34a, 34b	33b, 36	
**Ha**	1.40			34		28
**Hb**	1.21			34		28
**35 C**	28.01			35a, 35b	33b, 36	
**Ha**	1.33			35		
**Hb**	1.21			35	36	
**36 C**	28.15			36	35b, 37	
**H2**	1.27			36	34, 35, 37, 38	
**37 C**	18.33			37	36	
**H2**	2.10			37	36, 38	
**38 C**	81.38				36, 37	
**39 C**	80.52				40, 41a, 41b	
**40 C**	17.80			40	41a, 41b	
**H2**	2.10			40	39	
**41 C**	27.09			41b	42a, 42b, 43a, 43b	
**Ha**	1.41				39, 40, 42, 43	
**Hb**	1.31			41	39, 40, 42, 43	
**42 C**	25.64			42a, 42b	41a, 41b, 43a, 43b	
**Ha**	1.39			42	41	
**Hb**	1.38			42	41	
**43 C**	22.59			43a	41a, 41b, 44a, 44b	
**Ha**	1.81	11.8(44b)	44a, 44b	43	41, 42, 44	
**Hb**	1.77	5.0(44b)	44a, 44b	44	41, 42	
**44 C**	59.31			43b, 44a, 44b	20b, 43a	
**Ha**	3.23	11.9(44b)	43a, 43b, 44b	44	20, 22, 43	44b
**Hb**	2.98	11.9(44a), 11.8(43a), 5.0(43b)	43a, 43b, 44a	44	43	44a

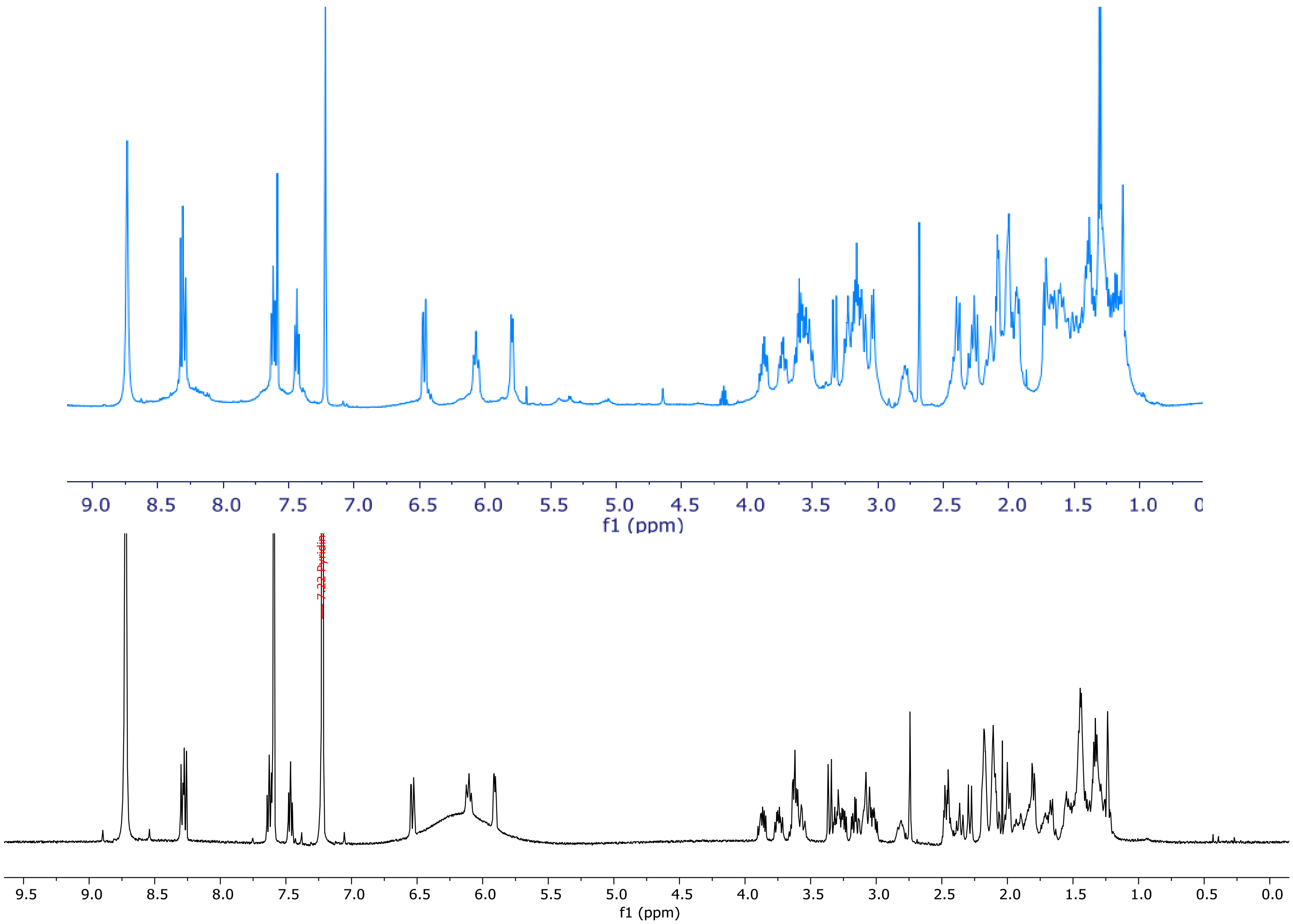

^{1}H NMR: Top: Isolated Njaoamine I (ref.[10]); bottom: Synthetic Nominal Njaoamine I ([D$_5$]-pyridine, 2.6 mg, 65 °C)

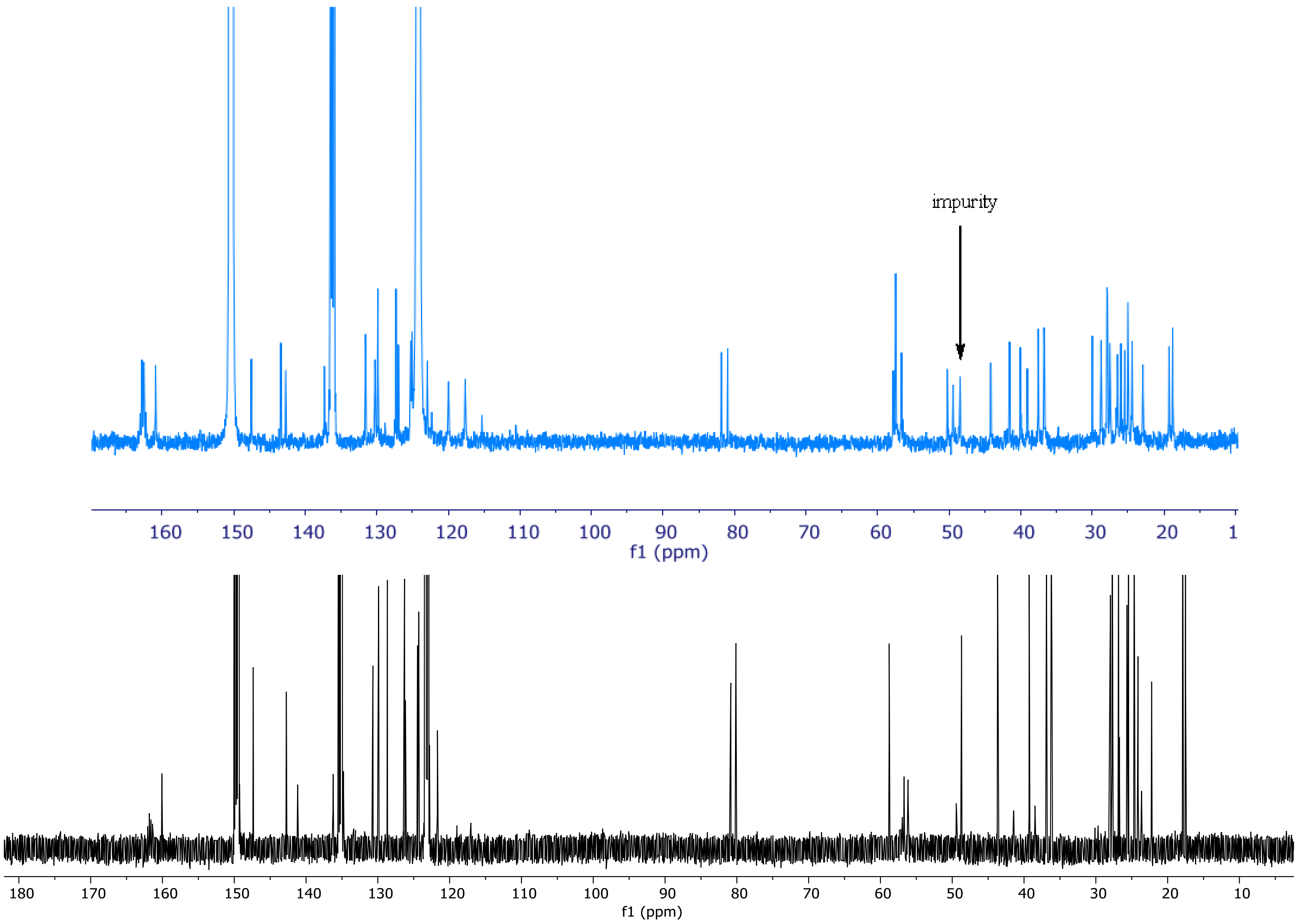

^{13}C NMR: Top: Isolated Njaoamine I (ref.[10]); bottom: Synthetic Nominal Njaoamine I ([D_5]-pyridine, ca.2.6 mg, 65 °C)

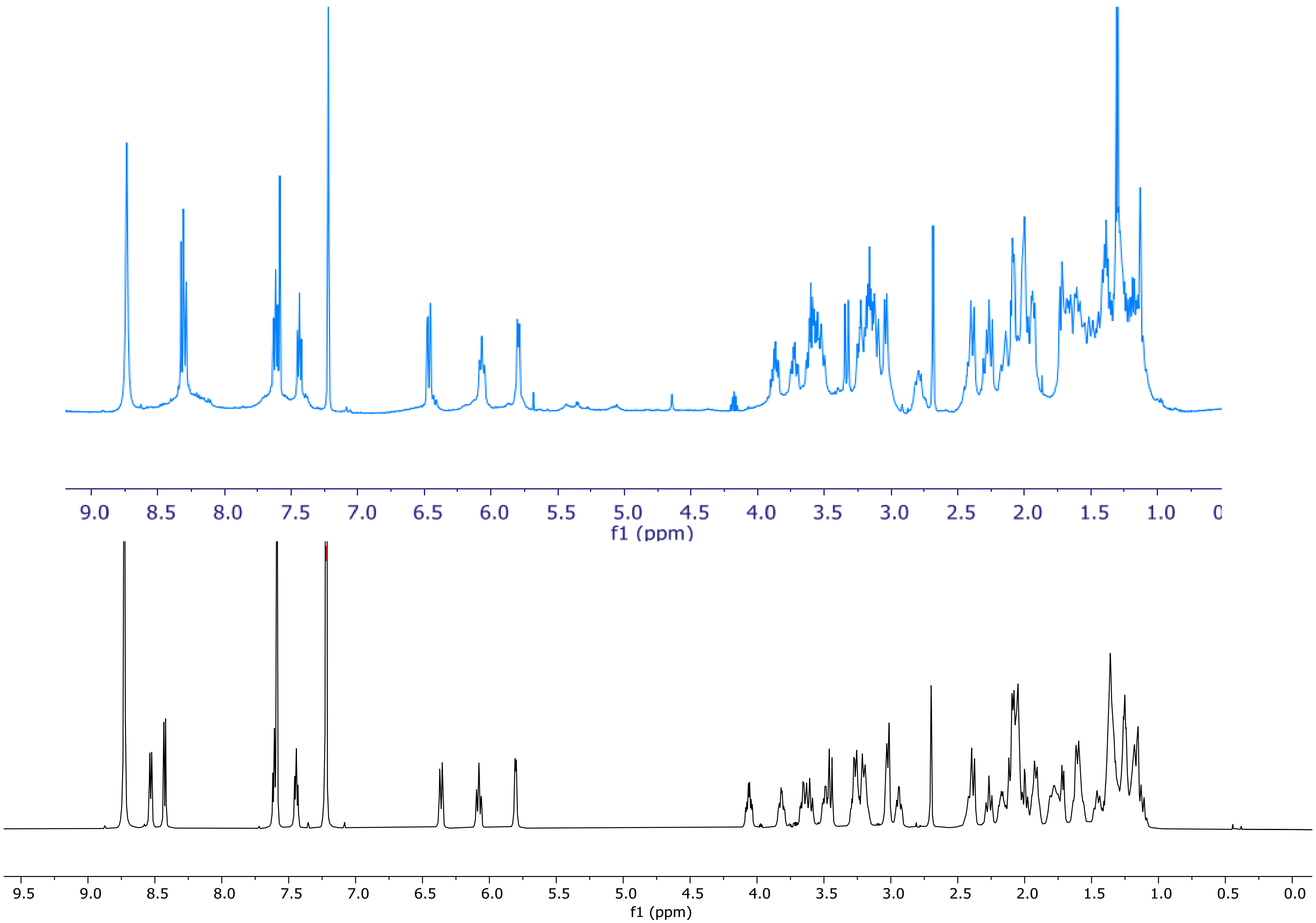

^{1}H NMR: Top: Isolated Njaoamine I (ref.[10]); bottom: Synthetic Nominal Njaoamine I ([D$_5$]-pyridine, ca.7.0 mg, 25 °C)

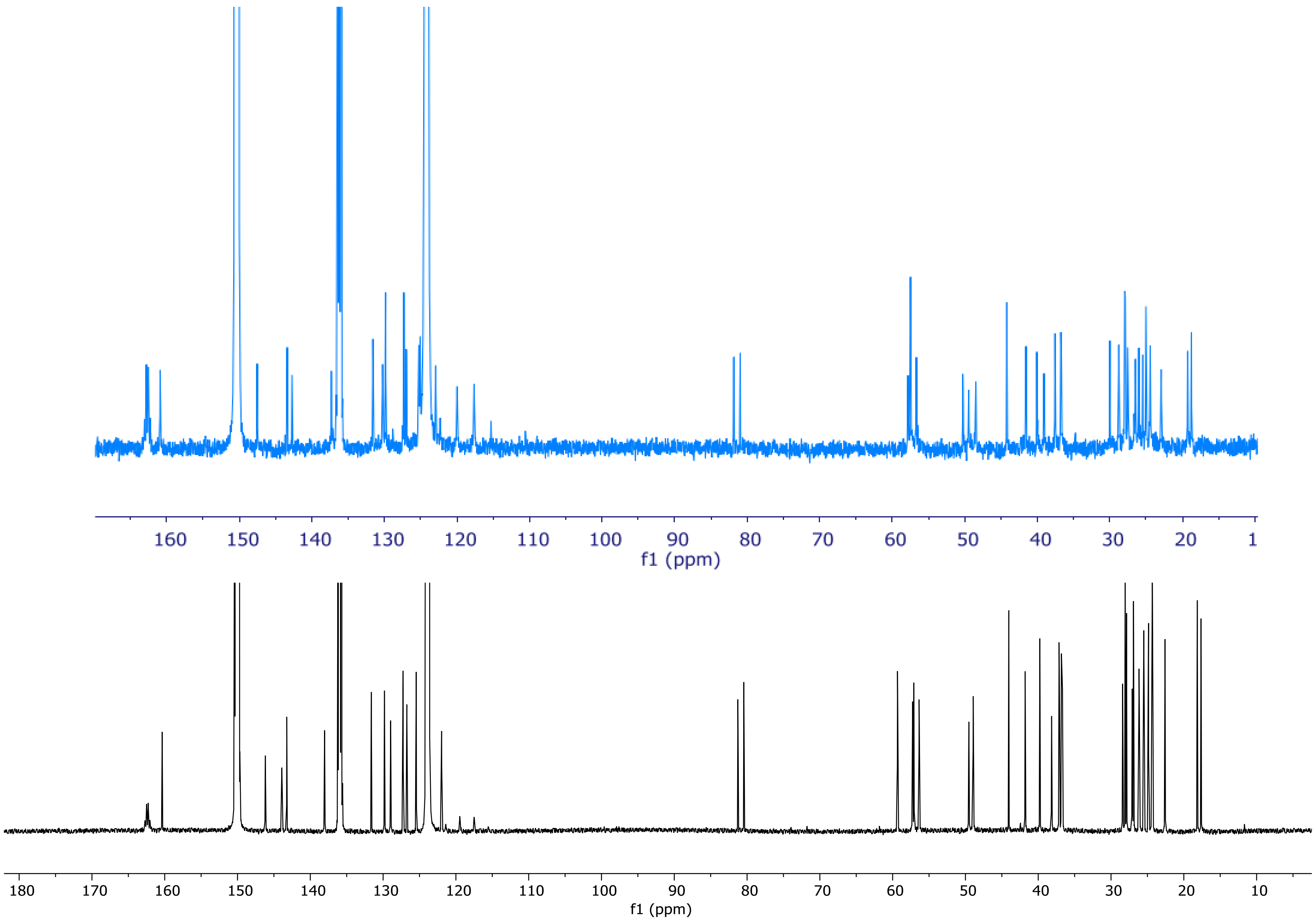

^{13}C NMR: Top: Isolated Njaoamine I (ref.[10]); bottom: Synthetic Nominal Njaoamine I ([D$_5$]-pyridine, ca.7.0 mg, 25 °C)

^{1}H NMR Spectrum of Synthetic Nominal Njaoamine I at Different Temperatures (2.6 mg of the TFA salt in [D5]-pyridine)

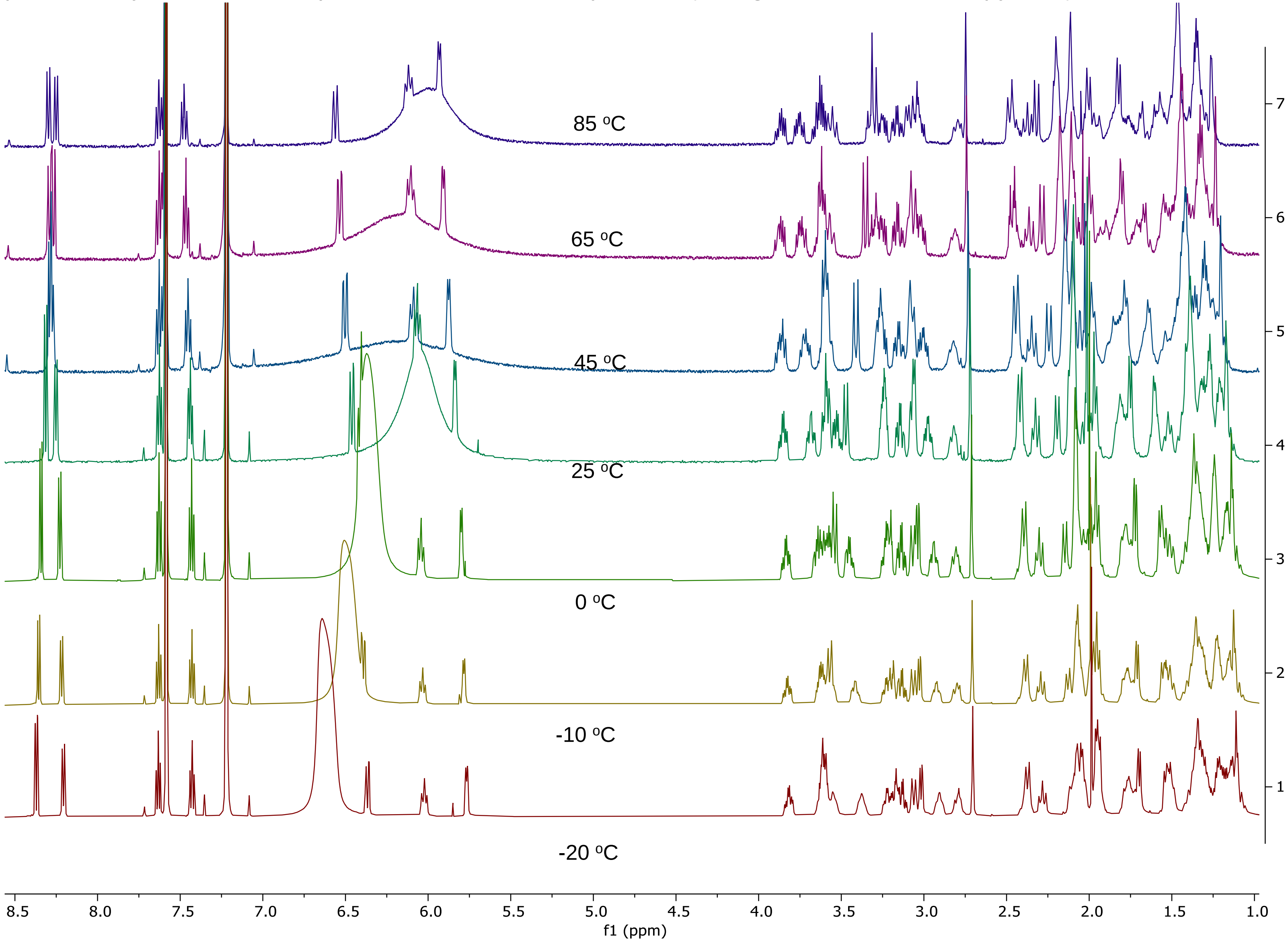

^{13}C NMR Spectrum of Synthetic Nominal Njaoamine I at Different Temperatures (2.6 mg of the TFA salt in [D$_5$]-pyridine)

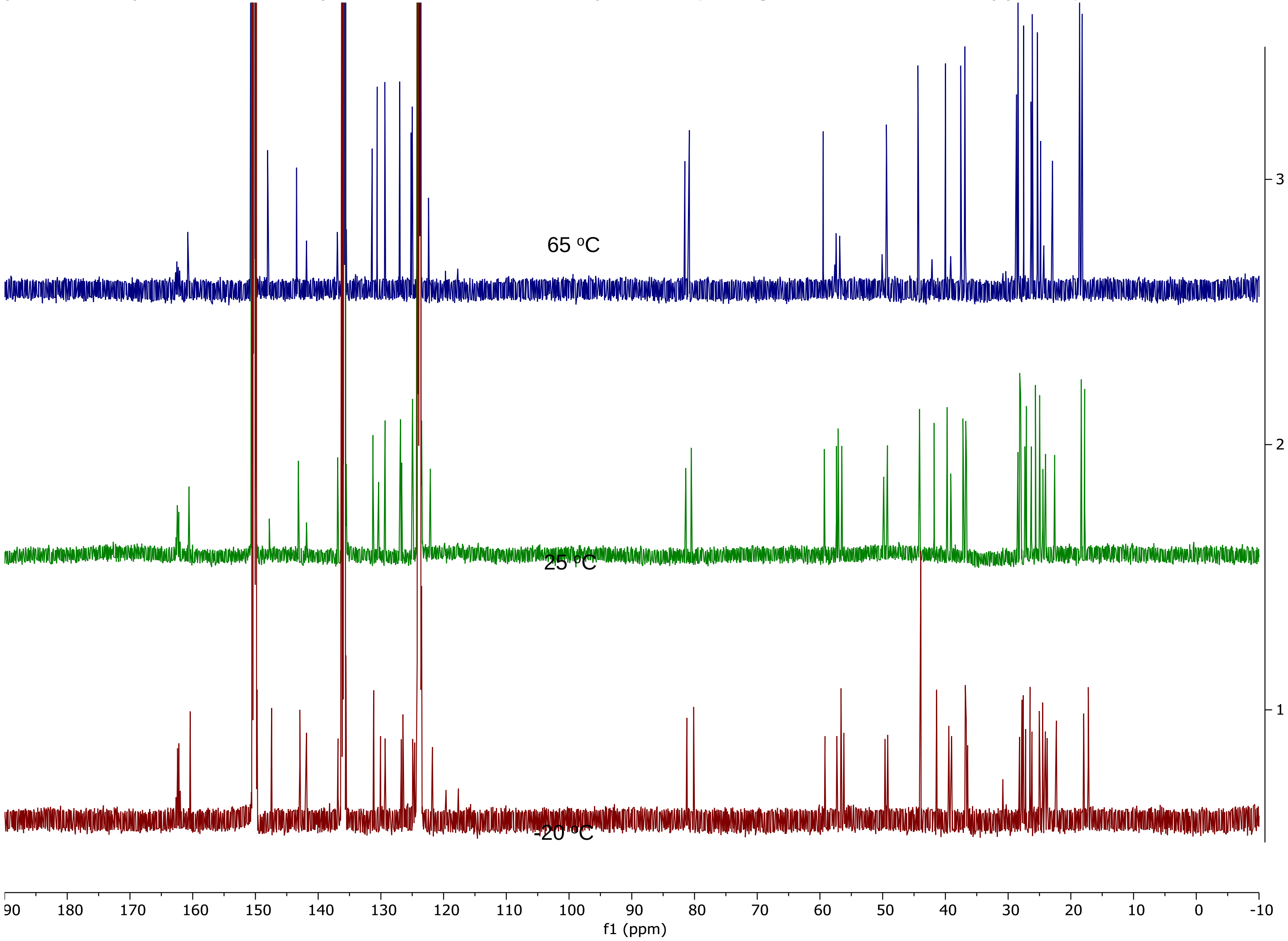

^{1}H NMR Spectrum of Synthetic Nominal Njaoamine I at Different Concentrations

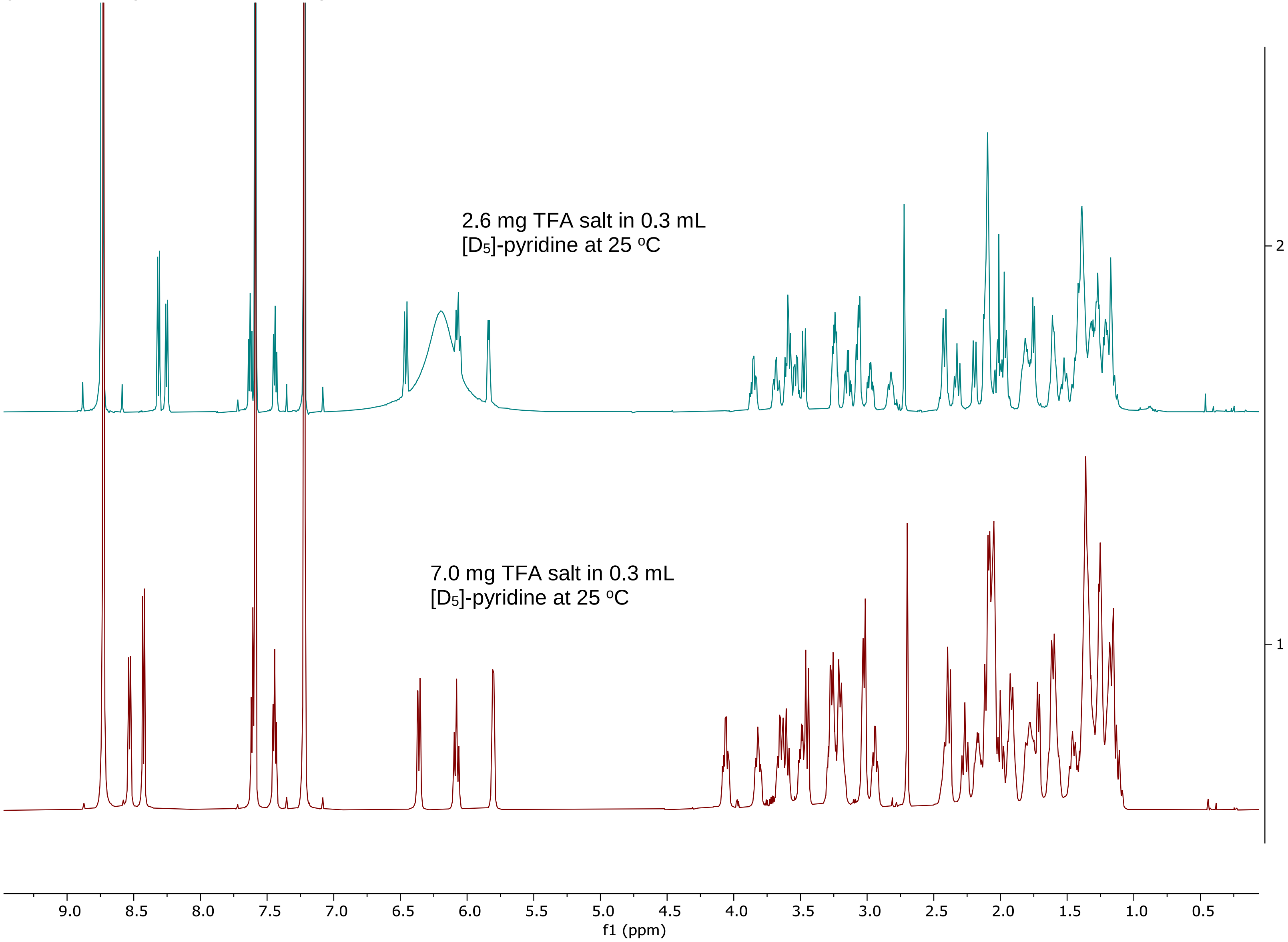

^{1}H NMR Spectrum of Synthetic Nominal Njaoamine I at Different pH Conditions

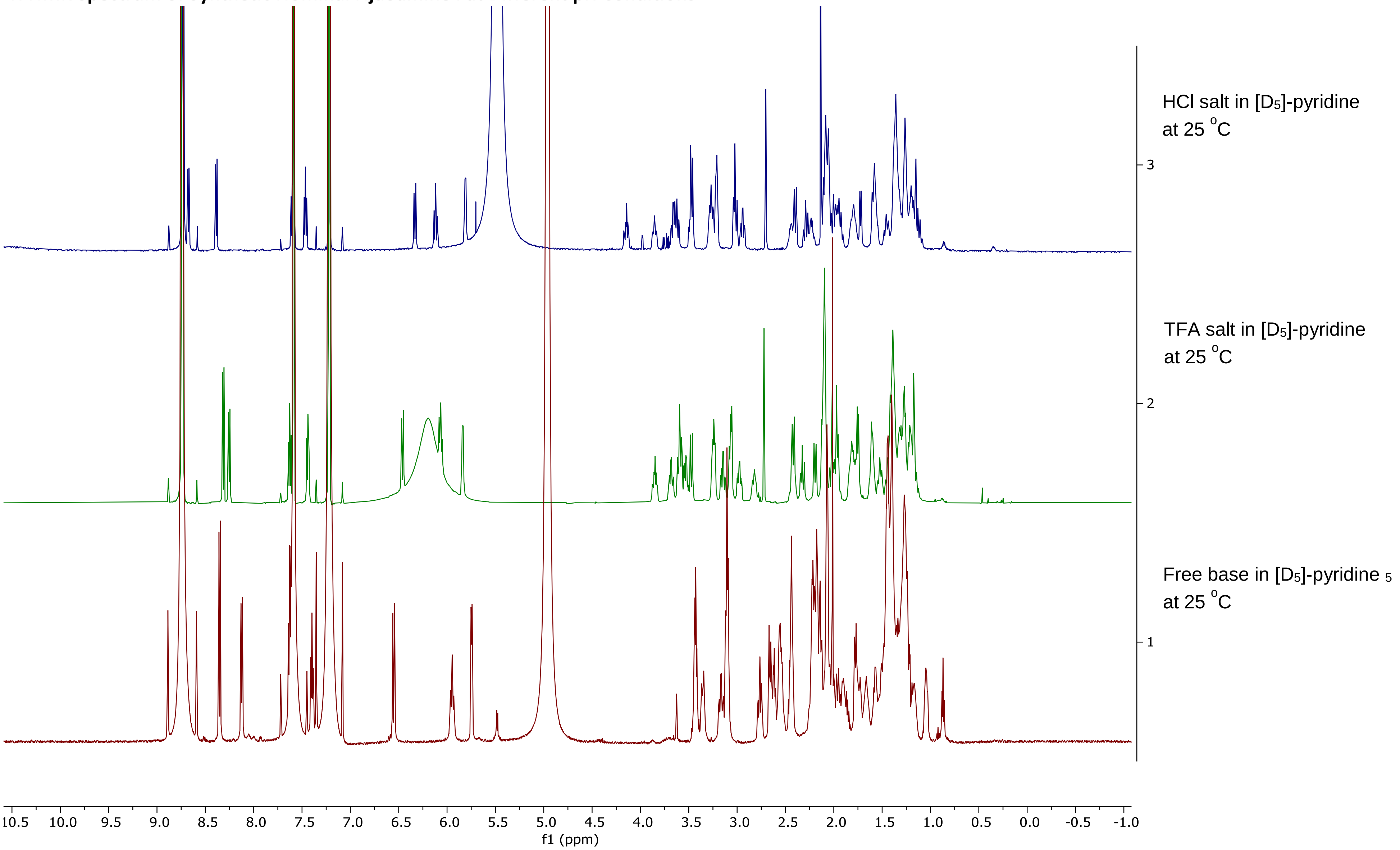

^{13}C NMR Spectrum of Synthetic Nominal Njaoamine I at Different pH Conditions

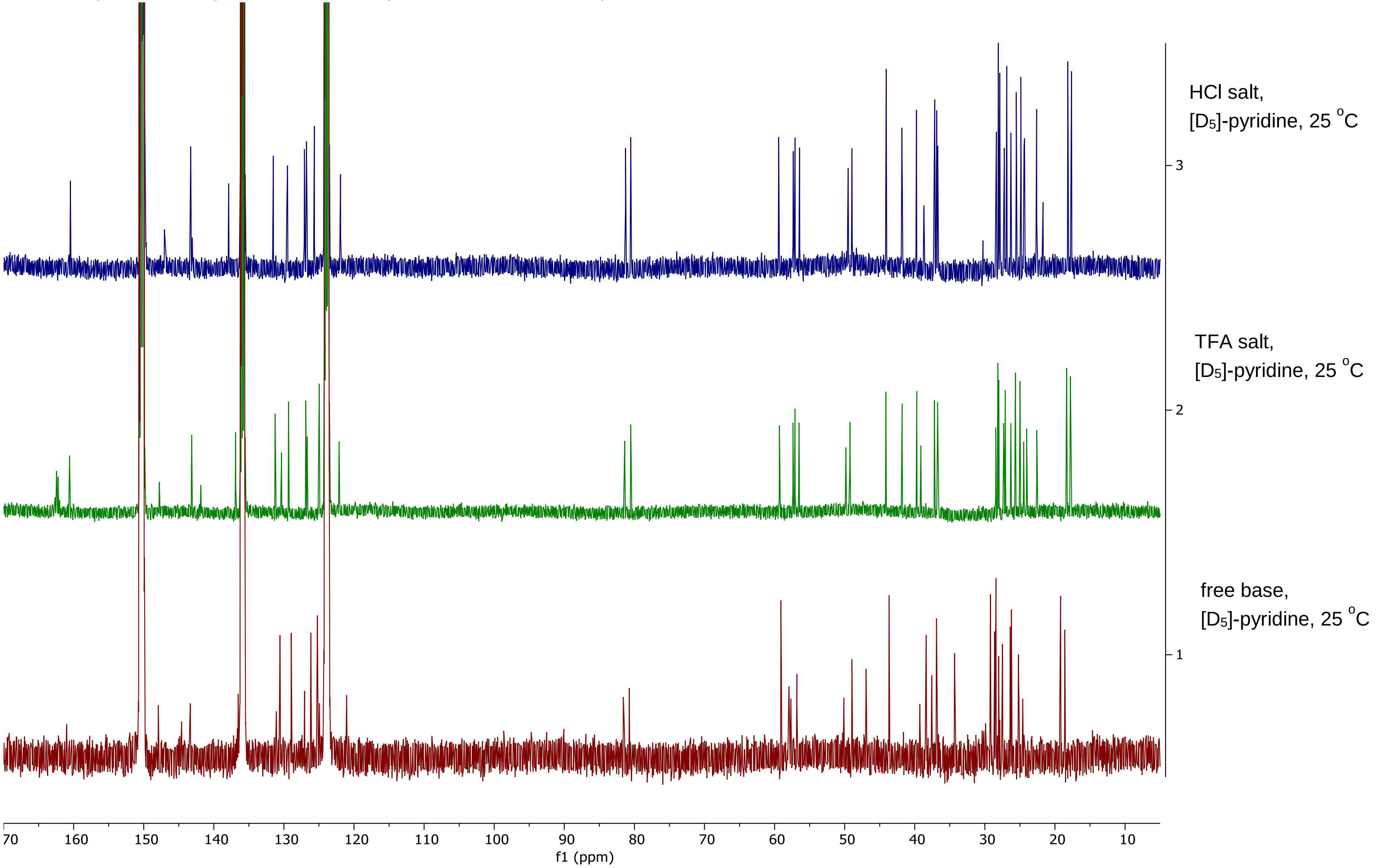

Comparison of Synthetic Nominal Njaomine I ((+)-4) with Native Njaoamine I

Table S10 places all known chemical shifts from the above assignment (synthetic “nominal” Njaoamine I) next to those reported from the natural product (isolated Njaoamine I) as reported in ref. [10]. This Table shows inconsistencies between both assignments with differences up to approx. 3.3 ppm in ^{13}C and up to 0.2 ppm in ^{1}H. Small chemical shift variations are usually expected based on subtle sample differences such as concentration, temperature, basicity, water and impurities, but in this case the possibility that the original report contains either mis-assignments or simply an incorrect structure needed to be taken into consideration. It is notable that the most significant differences cluster in the C_{12}-alkyne fragment (**Fragment C**).

Table S10. Comparison of the ^{13}C and ^{1}H chemical shifts of the isolated natural product njaoamine I and synthetic compound (+)-**4**; significant shift differences are highlighted

	Fragment A					
	**Njaomine I**		**Synthetic (+)-4**			
	δC	δH	δC	δH	|ΔδC|	|ΔδH|
**2 C**	160.50		160.59		0.04	
**3 C**	131.20		131.26		0.07	
**4 C**	142.40		141.84		0.69	
**5 C**	126.60		126.69		0.04	
**6 C**	124.90		124.96		0.07	
**H**		8.25		8.25		0.02
**7 C**	127.00		126.88		0.25	
**H**		7.42		7.44		0.00
**8 C**	129.50		129.34		0.29	
**H**		7.59		7.63		0.02
**9 C**	129.90		130.35		0.32	
**H**		8.28		8.31		0.01
**10 C**	147.20		147.78		0.45	
**11 C**	28.40		28.44		0.09	
**Ha**		3.83		3.85		0.00
**Hb**		3.68		3.68		0.02
**12 C**	39.70		39.72		0.11	
**Ha**		3.55		3.59		0.02
**Hb**		3.55		3.53		0.04
**13 C**	38.70		39.14		0.31	
**Ha**		3.22		3.26		0.02
**Hb**		3.15		3.14		0.03
**14 C**	26.10		26.29		0.06	
**Ha**		2.40		2.42		0.00
**Hb**		1.57		1.60		0.01
**15 C**	27.50		27.32		0.31	
**Ha**		1.39		1.52		0.11
**Hb**		1.39		1.43		0.02
**16 C**	56.30		56.51		0.08	
**Ha**		2.39		2.42		0.01
**Hb**		1.99		2.02		0.01
**29 C**	36.30		36.64		0.21	
**Ha**		2.28		2.33		0.03
**Hb**		1.92		1.96		0.02
**30 C**	24.10		24.04		0.19	
**Ha**		2.78		2.83		0.03
**Hb**		1.93		1.96		
**31 C**	136.90		136.89		0.14	
**H**		6.07		6.07		0.02
**32 C**	124.70		124.94		0.11	
**H**		6.45		6.46		0.01

	Fragment B					
	**nominal Njaomine I**		**synth. Njaomine I (+)-4**			
	δC	δH	δC	δH	|ΔδC|	|ΔδH|
**18 C**	57.10		57.09		0.14	
**H**		2.65		2.72		0.05
**19 C**	43.80		44.12		0.19	
**20 C**	49.10		49.84		0.61	
**Ha**		3.32		3.47		0.13
**Hb**		2.25		2.19		0.08
**22 C**	48.10		49.25		1.02	
**Ha**		3.50		3.59		0.07
**Hb**		3.10		3.07		0.05
**23 C**	25.10		24.46		0.77	
**Ha**		1.60		1.60		0.02
**Hb**		1.13		1.17		0.02
**24 C**	41.20		41.79		0.46	
**H**		1.12		1.17		0.03
**25 C**	37.20		37.20		0.13	
**H**		2.05		2.12		0.05
**26 C**	57.10		57.37		0.14	
**Ha**		3.04		3.07		0.01
**Hb**		1.72		1.75		0.01
**27 C**	143.00		143.16		0.03	
**28 C**	122.60		122.12		0.61	
**H**		5.80		5.84		0.02

	Fragment C					
	**nominal Njaomine I**		**synth. Njaomine I (+)-4**			
	δC	δH	δC	δH	|ΔδC|	|ΔδH|
**33 C**	36.40		36.74		0.21	
**Ha**		1.68		1.83		0.13
**Hb**		1.38		1.40		0.00
**34 C**	25.60		24.98		0.75	
**Ha**		1.45		1.40		0.07
**Hb**		1.27		1.21		0.08
**35 C**	24.60		28.01		3.28	
**Ha**		1.21		1.33		0.10
**Hb**		1.21		1.21		0.02
**36 C**	27.60		28.15		0.42	
**Ha**		1.30		1.27		0.05
**Hb**		1.30		1.27		0.05
**37 C**	18.40		18.33		0.20	
**Ha**		2.14		2.10		0.06
**Hb**		2.08		2.10		0.00
**38 C**	80.60		81.38		0.65	
**39 C**	81.50		80.52		1.11	
**40 C**	18.90		17.80		1.23	
**Ha**		2.00		2.10		0.08
**Hb**		2.00		2.10		0.08
**41 C**	29.70		27.09		2.74	
**Ha**		1.27		1.41		0.12
**Hb**		1.17		1.31		0.12
**42 C**	25.20		25.64		0.31	
**Ha**		1.41		1.39		0.04
**Hb**		1.26		1.38		0.10
**43 C**	22.50		22.59		0.04	
**Ha**		1.69		1.81	0.00	0.10
**Hb**		1.69		1.77	0.00	0.06
**44 C**	57.50		59.31		1.68	
**Ha**		3.16		3.23		0.05
**Hb**		3.16		2.98		0.20

At this point, we were fortunate that PharmaMar (Dr. Marta Perez Alvarez) kindly provided us with a small amount of the original natural product. A straightforward assessment for matching structures is to mix the natural product with its synthetic version into a single sample, which should then reveal a single set of signals. For this purpose, 0.2 mg of each material were mixed in 60 µL [D_5]-pyridine. Since the sample concentration is very low, only 1D-^{1}H spectra were measured for comparison.

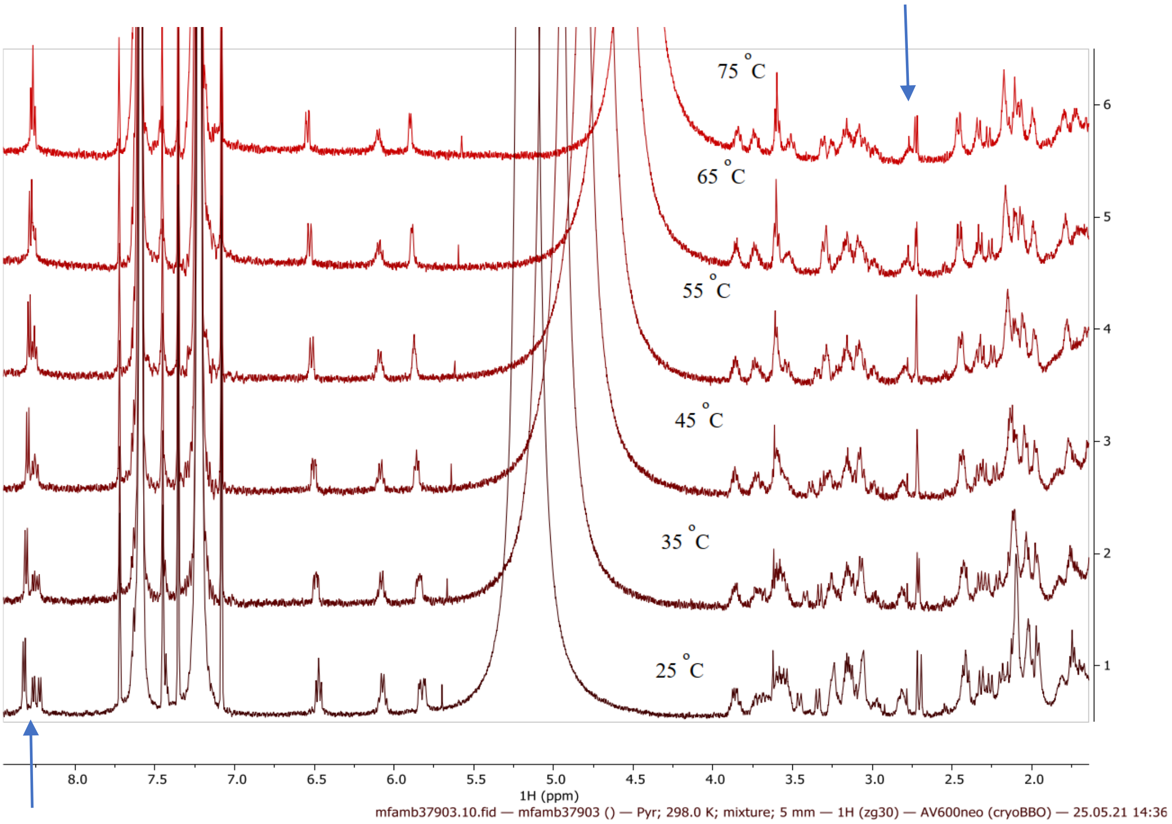

Figure S6. A sample of the trifluoroacetate salts of the isolated natural product (≈ 0.2 mg) and synthetic nominal njaoamine ((+)-**4**, ≈ 0.2 mg) were mixed and the ^{1}H NMR spectrum ([D_5]-pyridine) recorded at different temperatures; subtle inequivalences are visible in the entire temperature range (see arrows)

In the spectrum shown in Figure S6, signal doubling can clearly be observed, with H18 (singlet, 2.70 ppm) or H6 (doublet, 8.25 ppm) being prime examples (see arrows). In order to exclude that the signal doubling is due to a poor equilibration between materials (from widely different sources), the mixture sample was heated step wise from 25 °C to 75 °C and back. Figure S6 shows important temperature-dependent shifts (with signals even crossing) but the expected sustained merging of doubled signals was never observed.

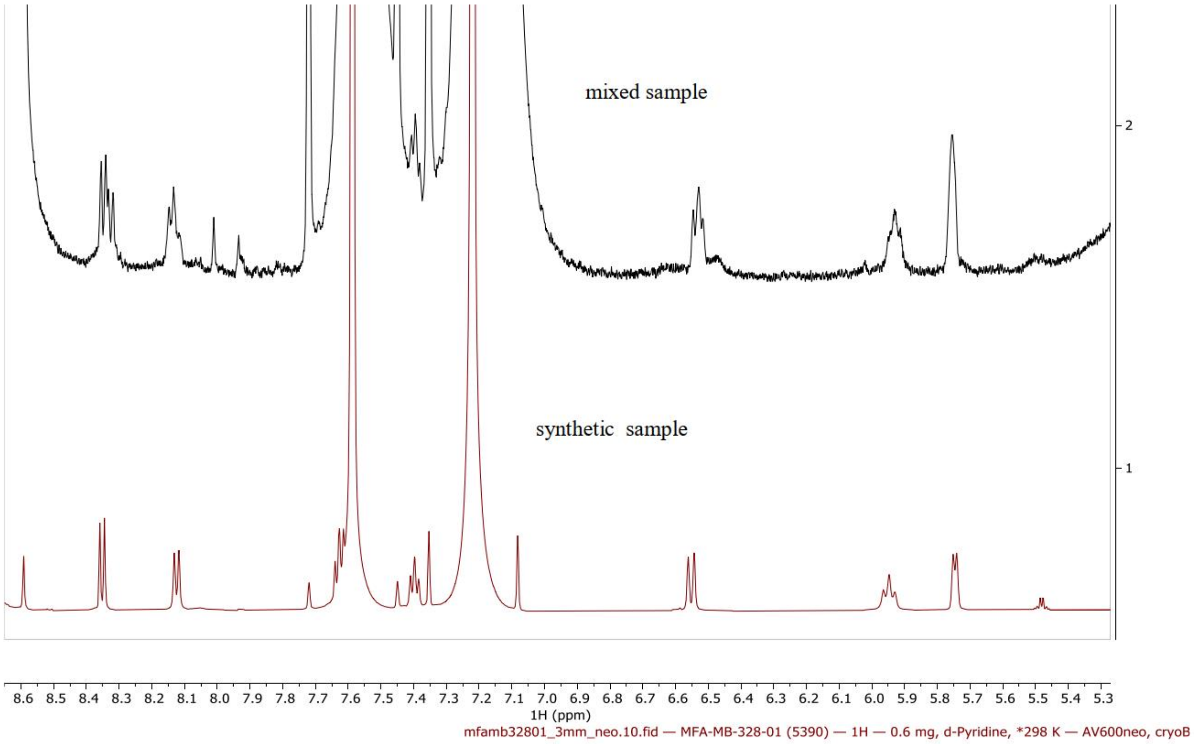

Figure S7. Second Doping Experiment: A sample of the isolated natural product (free base) (≈ 0.2 mg) and synthetic nominal Njaoamine ((+)-**4**, free base, ≈ 0.2 mg) were mixed and the ^{1}H NMR spectrum ([D_5]-pyridine) recorded at ambient temperature; subtle inequivalences are visible in the aromatic region

To exclude that the differences are due to a different counterion in the natural sample, the salt was transformed into the free base by preparative HPLC and the comparison repeated. As shown in Figure S7, a mismatch was also visible in this case. Therefore, one must conclude that there is a subtle yet true mismatch and that the structure assigned to njaoamine I needs to be revised. The chemical shift differences compiled in Table S10 point to fragment C (C12-chain) as the most probable site of mismatch.

Structure Revision of Njaoamine I Based on a Re-Assessment of an Authentic Sample

A targeted NMR assignment of the C12-chain in the natural product the Njaoamine I was undertaken, as it appears to be the most probable source of observed NMR disagreement between the natural product and the synthesized end-product. The labelling of the atoms from C33 to C44 follows the original publication,[10] in which the authors suggest that the alkyne is positioned exactly mid-way at position C38 (triple bond: C38≡C39).

PharmaMar (Dr. Marta Perez Alvarez) kindly provided us with a sample of the original natural product Njaoamine I for this study. The sample contained less than 1 mg of material, which was dissolved in [D$_5$]-pyridine and measured on a 600 MHz NMR Avance III spectrometer (Bruker) equipped with a cryogenically-cooled probehead (TCI) for best available sensitivity and resolution.

The complete assignment of the entire C12-chain proved challenging. For one, the poor sensitivity offered by the dilute sample rendered heteronuclear long-range correlation experiments (in particular HMBC) impractical. Secondly, the limited resolution impeded assignment, particularly among the overlapping coupled spin-systems in the aliphatic region, with 20 methylene protons and one methane proton between 1.1-1.7 ppm. There are also two very similar methylene ^{13}C-chemical shifts at 27.7 and 27.8 ppm, whose correlations can only be separated by very high-resolution multidimensional experiments.

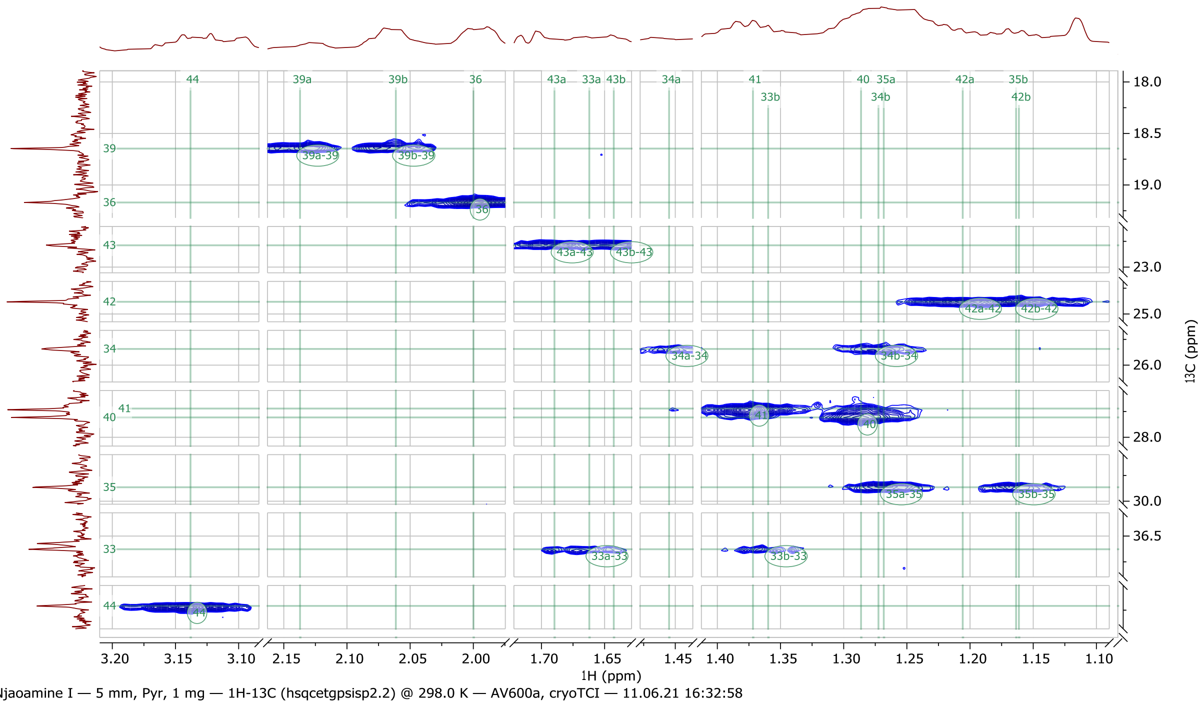

Figure S8. Excerpt from HSQC showing all $^1J_{CH}$ correlation in the C12-chain of njaoamine I.

The approach used was first to clearly identify the ^{1}H and ^{13}C chemical shifts of each of the 10 methylene (CH_2) groups belonging to the C12-chain. For this, a high resolution HSQC (2048 by 2048 points, 50% non-uniform sampling, 32 scans per increment, 7 hours) was measured. This experiment allowed the clear identification of all ^{1}H attached to the 10 methylene carbons, as shown in Figure S8.

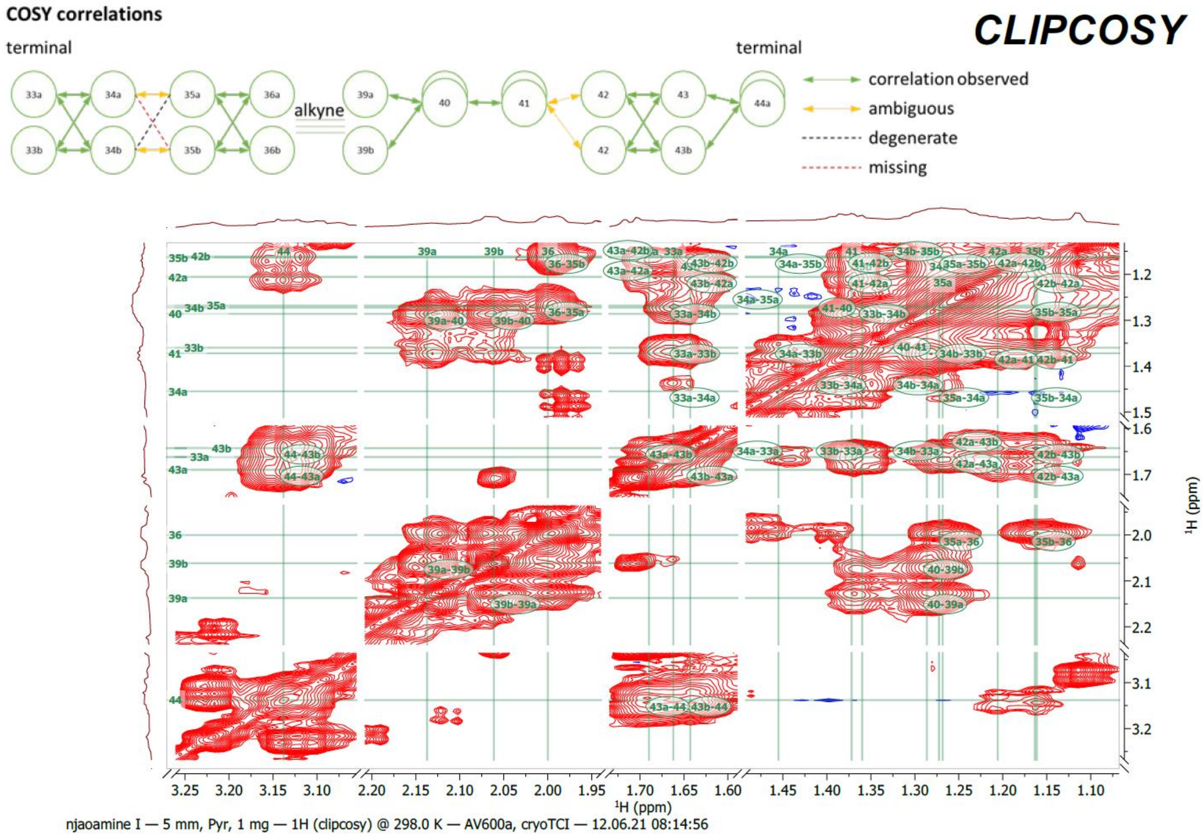

Figure S9. Subplot from the CLIP-COSY of njaoamine I. Labels show the expected $^{3}J_{HH}$ correlations. Inset above: summary of the observed correlations.

Secondly, a CLIP-COSY experiment[11] was used to survey the methylene chain via $^{3}J_{HH}$, in order to link the terminal H44 and H33 to one of the two CH_2-groups flanking the alkyne. The absorptive CLIP-COSY offers better resolution than the classical COSY (in magnitude mode) and its in-phase cross-peaks have better signal intensities compared to the anti-phase DQF-COSY. It however suffers from signal artefacts in cases of strongly coupled spins.[11] This is the case for the quasi-overlapping H34b and H35a, which cause antiphase signals at the position of the expected cross-peaks between H34a and H35b. Figure S9 displays the aliphatic region of the CLIP-COSY and a summary of the correlations observed for the C12-chain (insert). Clearly, ambiguity persists in the center of both aliphatic chains.

Thirdly, we measured a long, high-resolution HSQC-TOCSY experiment (2048x2048, 57% non-uniform sampling, 64 scans per increment, 2.5 d spectrometer time) with a mid-range mixing time of 80 ms. This experiment makes use of the ^{13}C signal dispersion to resolve and identify uninterrupted coupled ^{1}H spin systems. In effect, each ^{13}C carbon should exhibit the same set of correlations with all protons within the associated spin-system. Figure S10 displays ordered strips corresponding to each methylene ^{13}C, and presents the ladder-type peak patterns corresponding the attached ^{1}H spin system. It is expected that closer neighbors (2-3 bonds) generally have more intense cross-peaks then distant ones (4+ bonds).

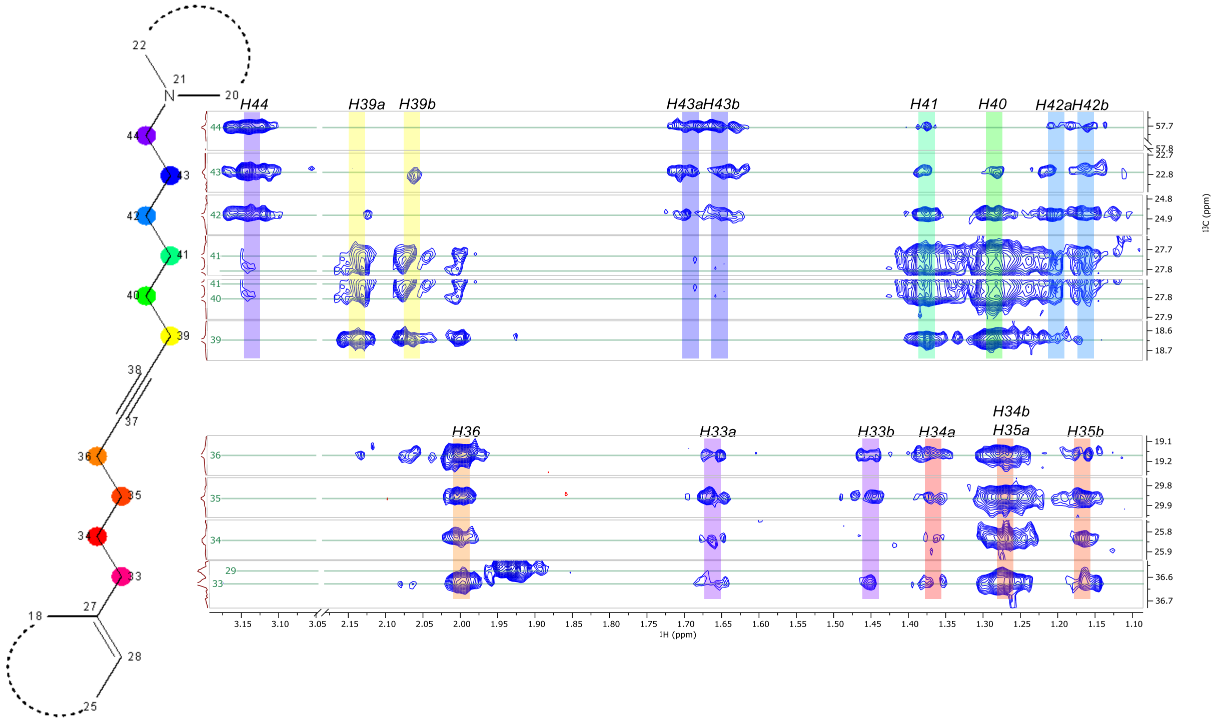

Figure S10. Ordered strips from the HSQC-TOCSY experiment: each ^{13}C-atom exhibits correlation cross peaks to related subset of ^{1}H present in the associated spin-system (color-coded). This clearly supports an asymmetric C12-chain with the alkyne at position C37-C38.

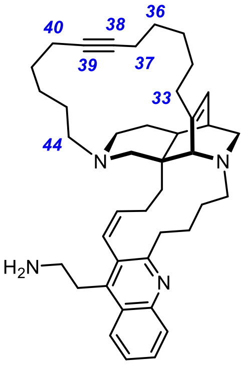

njaoamine I
(***nominal***)

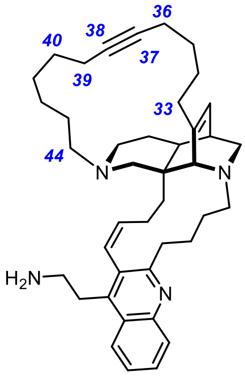

njaoamine I
(***revised assignment***)

A set of selective 1D ^{1}H-TOCSY were also measured with a long mixing-time (140 ms) and with selective irradiation on the terminal methylene protons H44 and H33a as well as on the methylene protons H39 and H36 flanking the alkyne (data not shown). The pattern of correlations also corroborates with the HSQC-TOCSY results shown above. From these observations, we are confident to propose a revised structure, in which the alkyne is at position C37 (triple bond: C37≡C38). The displacement of the rigid section within the otherwise flexible chain likely explains the small chemical shift differences between the natural product and the synthesized compound (nominal njaoamine I, (+)-**4**)) (see above).

For completeness, Table S11 presents the NMR assignments for the isolated natural product njaoamine I, with all observed correlations from TOCSY, COSY, HSQC, HMBC and ROESY experiments.

Table S11. Revised set of chemical shifts and correlations for the natural product njaoamine I

**Atom**	**δ (ppm)**	**J**	**COSY**	**TOCSY**	**HSQC**	**HMBC**	**ROESY**
**1 N**							
**2 C**	160.84					13a, 13b	
**3 C**	131.40					11a, 11b, 13b, 31	
**4 C**	141.95					11a, 11b	
**5 C**	126.83					7, 9, 11a, 11b	
**6 C**	125.05				6	8	
**H**	8.26	8.4(7)	7		6	8, 10	11a, 11b, 12b
**7 C**	127.05				7	9	
**H**	7.42	8.4(6), 6.9(8)	6, 8		7	5, 9	
**8 C**	129.50				8	6	
**H**	7.60	6.9(7)	7		8	6, 10	
**9 C**	130.60				9	7	
**H**	8.29				9	5, 7	
**10 C**	147.95					6, 8	
**11 C**	28.60				11a, 11b		
**Ha**	3.85	5.4(12a), 12.2(12b), 12.5(11b)	11b, 12a, 12b	11b, 12a	11	3, 4, 5, 12	6, 11b

**Hb**	3.71	12.5(11a), 12.0(12a), 4.5(12b)	11a, 12a	11a, 12a	11	3, 4, 5, 12	6, 11a, 32
**12 C**	39.94				12a	11a, 11b	
**Ha**	3.59	12.5(12b), 5.4(11a), 12.0(11b)	11a, 11b, 12b	11a, 11b	12		
**Hb**	3.53	12.5(12a), 12.2(11a), 4.5(11b)	11a, 12a				6
**13 C**	39.29				13a, 13b		
**Ha**	3.23	12.9(13b), 3.7(14?), 3.7(14?)	13b, 14a, 14b	13b, 14a, 14b, 15a, 15b, 16a, 16b	13	2	14a, 14b
**Hb**	3.12	12.9(13a)	13a, 14a, 14b	13a, 14a, 14b, 15a, 15b, 16a, 16b	13	2, 3, 14	14b, 32
**14 C**	26.39				14a, 14b	13b, 16a	
**Ha**	2.40		13a, 13b, 14b, 15a, 15b	13a, 13b, 16a, 16b	14		13a, 14b, 15a, 29a
**Hb**	1.57		13a, 13b, 14a, 15a, 15b	13a, 13b, 15b, 16a, 16b	14		13a, 13b, 14a, 16b
**15 C**	27.45				15a, 15b		
**Ha**	1.48		14a, 14b, 15b, 16a, 16b	13a, 13b, 16a, 16b	15		14a, 16b, 18
**Hb**	1.40		14a, 14b, 15a, 16a, 16b	13a, 13b, 14b, 16a, 16b	15		16a, 18
**16 C**	56.59				16a, 16b	18, 26b	
**Ha**	2.37		15a, 15b, 16b	13a, 13b, 14a, 14b, 15a, 15b, 16b	16	14	15b, 16b, 18, 26b
**Hb**	1.98		15a, 15b, 16a	13a, 13b, 14a, 14b, 15a, 15b, 16a	16		14b, 15a, 16a, 26b
**17 N**							
**18 C**	57.38				18	26a, 29b	
**H**	2.67			25, 26a, 26b, 28	18	16, 19, 24, 26, 27, 28, 29, 33	15a, 15b, 16a, 20a, 20b, 29b, 33a, 33b, 34a
**19 C**	44.07					18, 20a, 20b, 23a, 25	
**20 C**	49.32				20a, 20b	29a	
**Ha**	3.32	12.5(20b)	20b	20b	20	19, 22, 24, 29	18, 20b, 29b, 30a
**Hb**	2.23	12.5(20a)	20a	20a	20	19, 29, 44	18, 20a, 24, 28, 43b, 44
**21 N**							
**22 C**	48.30				22a, 22b	20a, 44	
**Ha**	3.51		22b, 23a, 23b	22b, 23a, 23b, 24, 25	22		22b, 23a, 23b, 29b
**Hb**	3.08		22a, 23a, 23b	22a, 23a, 23b, 24	22		22a, 23b
**23 C**	25.32				23a, 23b	24	
**Ha**	1.59		22a, 22b, 24	22a, 22b, 23b	23	19	22a, 23b, 24, 25
**Hb**	1.12		22a, 22b, 24	22a, 22b, 23a	23		22a, 22b, 23a, 25, 28
**24 C**	41.46				24	18, 20a, 26a, 26b	
**H**	1.11		23a, 23b, 25	22a, 22b, 25, 28	24	23, 28, 29	20b, 23a, 25, 26a, 29a, 29b
**25 C**	37.45				25	26a, 26b, 28	
**H**	2.06	7.0(28)	24, 26a, 26b, 28	18, 22a, 24, 26a, 28	25	19, 27	23a, 23b, 24, 26a, 26b, 28

**26 C**	57.38				26a, 26b	18, 28	
**Ha**	3.03	9.0(26b)	25, 26b	18, 25, 28	26	18, 24, 25, 28	24, 25, 26b, 29a, 30a
**Hb**	1.71	9.0(26a)	25, 26a	18, 28	26	16, 24, 25	16a, 16b, 25, 26a, 28
**27 C**	143.28					18, 25, 33b	
**28 C**	122.77				28	18, 24, 26a, 33b	
**H**	5.78	7.0(25)	25	18, 24, 25, 26a, 26b	28	25, 26	20b, 23b, 25, 26b, 34a, 34b, 35a
**29 C**	36.57				29a, 29b	18, 20a, 20b, 24, 31	
**Ha**	2.28		29b, 30a, 30b	30a, 31, 32	29	20, 30, 31	14a, 24, 26a, 29b, 31
**Hb**	1.93		29a, 30a, 30b	30b, 31, 32	29	18, 30, 31	18, 20a, 22a, 24, 29a, 30a, 31
**30 C**	24.29				30a, 30b	29a, 29b, 31, 32	
**Ha**	2.79		29a, 29b, 31	29a, 30b, 32	30		20a, 26a, 29b, 30b, 31
**Hb**	1.93	8.7(31)	29a, 29b, 31	29b, 30a, 31, 32	30		30a, 31
**31 C**	137.03				31	29a, 29b	
**H**	6.06	8.7(30b), 11.0(32)	30a, 30b, 32	29a, 29b, 30b, 32	31	3, 29, 30	29a, 29b, 30a, 30b
**32 C**	125.11				32		
**H**	6.45	11.0(31), 2.5(?)	31	29a, 29b, 30a, 30b, 31	32	30	11b, 13b
**33 C**	36.63				33a, 33b	18	
**Ha**	1.66		33b, 34a, 34b	33b, 34a, 34b, 35a, 35b, 36	33		18, 33b, 34a, 34b
**Hb**	1.37		33a, 34a, 34b	33a, 34a, 34b, 35a, 35b, 36	33	27, 28	18, 33a, 36
**34 C**	25.84				34a, 34b		
**Ha**	1.45		33a, 33b, 34b, 35a, 35b	33a, 33b, 34b, 35a, 35b, 36	34		18, 28, 33a, 35a, 35b
**Hb**	1.27		33a, 33b, 34a	33a, 33b, 34a, 35b, 36	34		28, 33a
**35 C**	29.86				35a, 35b		
**Ha**	1.27		34a, 35b, 36	33a, 33b, 34a, 35b, 36	35		28, 34a, 36, 39a
**Hb**	1.16		34a, 35a, 36	33a, 33b, 34a, 34b, 35a, 36	35		34a, 36
**36 C**	19.17				36		
**H2**	2.00		35a, 35b	33a, 33b, 34a, 34b, 35a, 35b	36		33b, 35a, 35b
**37 C**	81.73						
**38 C**	80.84					39b	
**39 C**	18.65				39a, 39b		
**Ha**	2.14		39b, 40	39b, 40, 41, 42a, 42b, 43a, 43b, 44	39		35a, 40
**Hb**	2.06		39a, 40	39a, 40, 41, 43a, 43b, 44	39	38	40
**40 C**	27.81				40		
**H2**	1.29		39a, 39b, 41	39a, 39b, 41, 42a, 42b, 43a, 43b, 44	40		39a, 39b, 43a
**41 C**	27.73				41	42a, 42b, 43a, 43b	
**H2**	1.37		40, 42a, 42b	39a, 39b, 40, 42a, 42b, 43a, 43b, 44	41		42b, 43b

**42 C**	24.88				42a, 42b	43a, 43b	
**Ha**	1.21		41, 43a, 43b	39a, 40, 41, 42b, 44	42	41, 43, 44	
**Hb**	1.16		41, 43a, 43b	39a, 40, 41, 42a, 44	42	41, 43, 44	41, 43a, 43b
**43 C**	22.79				43a, 43b	42a, 42b, 44	
**Ha**	1.69		42a, 42b, 43b, 44	39a, 39b, 40, 41, 43b, 44	43	41, 42	40, 42b
**Hb**	1.64		42a, 42b, 43a, 44	39a, 39b, 40, 41, 43a, 44	43	41, 42	20b, 41, 42b
**44 C**	57.71				44	20b, 42a, 42b	
**H2**	3.14		43a, 43b	39a, 39b, 40, 41, 42a, 42b, 43a, 43b	44	22, 43	20b

As a final inspection, Table S12 compares the original assignment as published by the isolation team in ref. [10], with the revised assignment of native njaoamine I, in order to confirm the matching identity of the sample and to highlight the corrected assignments.

As can be seen, seven ^{13}C NMR signals were reassigned in the following way:

- peak at 29.86 ppm was originally assigned to C41(29.7 ppm) and is now assigned to **C35**
- peak at 19.17 ppm was originally assigned to C40(18.9 ppm) and is now assigned to **C36**
- peak at 81.73 ppm was originally assigned to C39(81.5 ppm) and is now assigned to **C37**
- peak at 18.65 ppm was originally assigned to C37(18.4 ppm) and is now assigned to **C39**
- peak at 27.81 ppm was originally assigned to C36(27.6 ppm) and is now assigned to **C40**
- peak at 24.88 ppm was originally assigned to C35(24.6 ppm) and is now assigned to **C42**

A single ^{13}C signal at 27.73 ppm, assigned here to C41, did not have any equivalence in the original assignment probably due to its closeness to C40 (27.81 ppm). Inversely, the original assignment reports a signal at 25.2 ppm, which is not observed in the present assignment. It is unlikely that they correspond to each other, as this would be the largest shift difference (+2.5ppm) among all 41 carbons. More likely is that the peak at 25.1 ppm (C23) was originally interpreted to contain a second carbon signal, and its attached 1H (1.45, 1.27 ppm) were identified as HSQC cross-peaks overlapping with those of C34 (^{13}C: 25.6ppm; ^{1}H: 1.41, 1.26 ppm).

In effect, comparing spectra directly shows very similar shifts throughout, especially in the problematic aliphatic region as depicted in the overlayed 1D ^{13}C and HSQC spectra shown in Figure S11.

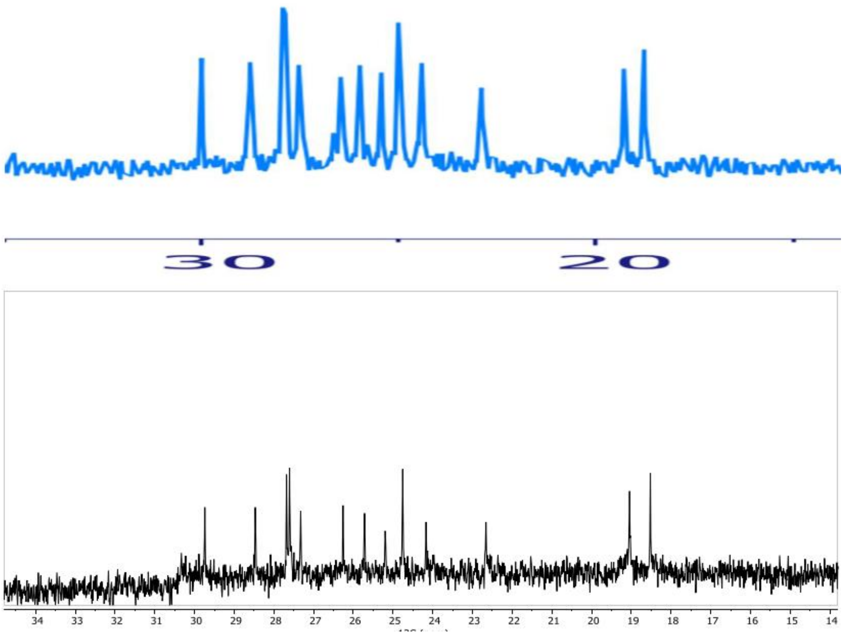

B

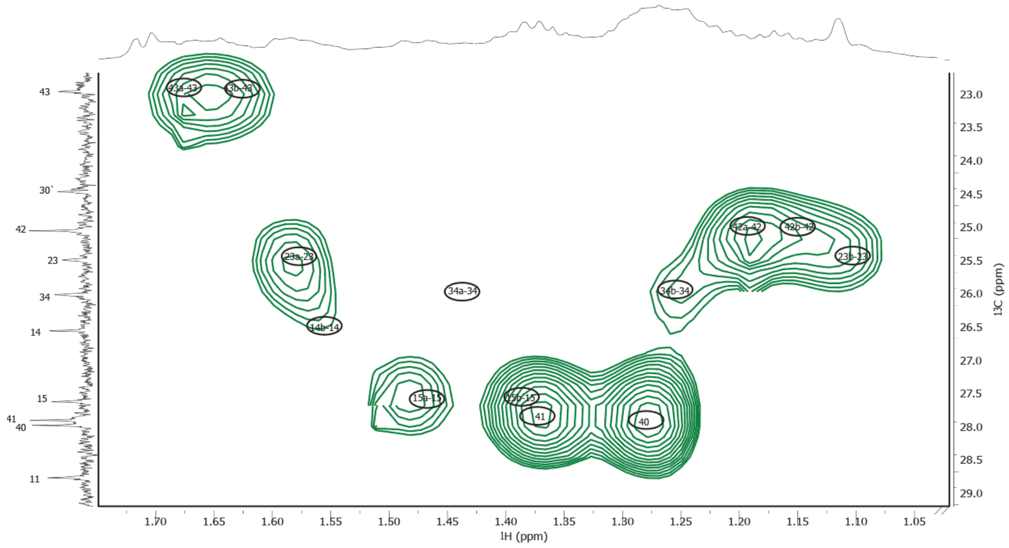

Figure S11. Comparison of the crowded aliphatic region of native njaoamine I: (A) 1D ^{13}C NMR spectra: top: original report (ref. [10], SI p. S5) vs. bottom: this report. (B) ^{13}C-HSQC spectrum: Green contours (original report, ref. [10], SI pS6) vs black ellipses indicating positions of signals in this report.

Table S12. Comparison of the revised and original (ref. 10) assignment of njaoamine I.

native Njaoamine I							
**revised assignment**			**original assignment ***			diff**	
	δC	δH		δC	δH	|ΔδC|	|ΔδH|
**2 C**	160.84		**2 C**	160.5		0.04	
**3 C**	131.40		**3 C**	131.2		0.10	
**4 C**	141.95		**4 C**	142.4		0.75	
**5 C**	126.83		**5 C**	126.6		0.07	
**6 C**	125.05		**6 C**	124.9		0.15	
**H**		8.26	**H**		8.25		0.01
**7 C**	127.05		**7 C**	127.0		0.25	
**H**		7.42	**H**		7.42		0
**8 C**	129.50		**8 C**	129.5		0.30	
**H**		7.60	**H**		7.59		0.01
**9 C**	130.60		**9 C**	129.9		0.40	
**H**		8.29	**H**		8.28		0.01
**10 C**	147.95		**10 C**	147.2		0.45	
**11 C**	28.60		**11 C**	28.4		0.10	
**Ha**		3.85	**Ha**		3.83		0.02
**Hb**		3.71	**Hb**		3.68		0.03
**12 C**	39.94		**12 C**	39.7		0.06	
**Ha**		3.59	**Ha**		3.55		0.04
**Hb**		3.53	**Hb**		3.55		0.02
**13 C**	39.29		**13 C**	38.7		0.29	
**Ha**		3.23	**Ha**		3.22		0.01
**Hb**		3.12	**Hb**		3.15		0.03
**14 C**	26.39		**14 C**	26.1		0.01	
**Ha**		2.40	**Ha**		2.40		0
**Hb**		1.57	**Hb**		1.57		0
**15 C**	27.45		**15 C**	27.5		0.35	
**Ha**		1.48	**Ha**		1.39		0.09
**Hb**		1.40	**Hb**		1.39		0.01
**16 C**	56.59		**16 C**	56.3		0.01	
**Ha**		2.37	**Ha**		2.39		0.02
**17 N**			**17 N**				
**18 C**	57.38		**18 C**	57.1		0.02	
**H**		2.67	**H**		2.65		0.02
**19 C**	44.07		**19 C**	43.8		0.03	
**20 C**	49.32		**20 C**	49.1		0.08	
**Ha**		3.32	**Ha**		3.32		0
**Hb**		2.23	**Hb**		2.25		0.02
**22 C**	48.30		**22 C**	48.1		0.10	
**Ha**		3.51	**Ha**		3.50		0.01
**Hb**		3.08	**Hb**		3.10		0.02
**23 C**	25.32		**23 C**	25.1		0.08	
**Ha**		1.59	**Ha**		1.60		0.01
**Hb**		1.12	**Hb**		1.13		0.01
**24 C**	41.46		**24 C**	41.2		0.04	
**H**		1.11	**H**		1.12		0.01
**25 C**	37.45		**25 C**	37.2		0.05	
**H**		2.06	**H**		2.05		0.01
**26 C**	57.38		**26 C**	57.1		0.02	
**Ha**		3.03	**Ha**		3.04		0.01
**Hb**		1.71	**Hb**		1.72		0.01
**27 C**	143.28		**27 C**	143.0		0.02	
**28 C**	122.77		**28 C**	122.6		0.13	
**H**		5.78	**H**		5.80		0.02
**29 C**	36.57		**29 C**	36.3		0.03	
**Ha**		2.28	**Ha**		2.28		0
**Hb**		1.93	**Hb**		1.92		0.01
**30 C**	24.29		**30 C**	24.1		0.11	
**Ha**		2.79	**Ha**		2.78		0.01
**Hb**		1.93	**Hb**		1.93		
**31 C**	137.03		**31 C**	136.9		0.17	
**H**		6.06	**H**		6.07		0.01
**32 C**	125.11		**32 C**	124.7		0.11	
**H**		6.45	**H**		6.45		0
**33 C**	36.63		**33 C**	36.4		0.07	
**Ha**		1.66	**Ha**		1.68		0.02
**Hb**		1.37	**Hb**		1.38		0.01
**34 C**	25.84		**34 C**	25.6		0.06	
**Ha**		1.45	**Ha**		1.45		0
**Hb**		1.27	**Hb**		1.27		0
**35 C**	29.86		**35 C**	24.6		0.02	
**Ha**		1.27	**Ha**		1.21		0
**Hb**		1.16	**Hb**		1.21		0.05
**36 C**	19.17		**36 C**	27.6		0.09	
**Ha**		2.00	**Ha**		1.30		0.01
**Hb**		2.00	**Hb**		1.30		0.01
**37 C**	81.73		**37 C**	18.4		0.05	
			**Ha**		2.14		0
			**Hb**		2.08		0.02
**38 C**	80.84		**38 C**	80.6		0.06	
**39 C**	18.65		**39 C**	81.5		0.07	
**Ha**		2.14					
**Hb**		2.06					
**40 C**	27.81		**40 C**	18.9		0.03	
**Ha**		1.29	**Ha**		2.00		0
**Hb**		1.29	**Hb**		2.00		0
**41 C**	27.73		**41 C**	29.7		0.14	
**Ha**		1.37	**Ha**		1.27		0
**Hb**		1.37	**Hb**		1.17		0.01
**42 C**	24.88		***42 C***	*25.2*	*****		
**Ha**		1.21	***Ha***		*1.41*		
**Hb**		1.16	***Hb***		*1.26*		
**43 C**	22.79		**43 C**	22.5		0.01	
**Ha**		1.69	**Ha**		1.69	0.00	0
**Hb**		1.64	**Hb**		1.69	0.00	0.05
**44 C**	57.71		**44 C**	57.5		0.09	
**Ha**		3.14	**Ha**		3.16		0.02
**Hb**		3.14	**Hb**		3.16		0.02
					average	0.12	0.01

←→ : 13C reassignment

***from Urba et al. [10]**

****diff: absolute difference between corresponding chemical shifts.**

***** Peak @ 25.2ppm has no equivalent signal in the new 13C**

Two Concurrent RCAM Reactions

Compound 72. L-Selectride (1 M in THF, 1.5 mL, 1.5 mmol) was added to a solution of compound **68** (250 mg, 0.114 mmol) in THF (0.2 mL). The reaction was stirred at 40 °C for 12 h before it was quenched by cautious addition of MeOH (0.5 mL) at 0 °C. The resulting mixture was loaded onto an amino cartridge (pre-equilibrated with MeOH, H_2O, MeOH (volume of three column length each)) and then eluted with MeOH/H_2O (90:10) to provide a white solid.

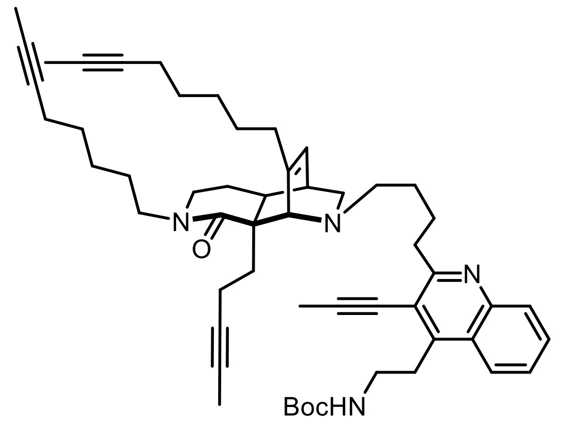

HOAc (0.02 mL, 0.349 mmol) was added to a solution of this secondary amine and aldehyde **52** (320 mg, 0.841 mmol) in CH_2Cl_2 (1.2 mL) at ambient temperature. After stirring for 30 min at this temperature, $NaBH(OAc)_3$ (84 mg, 0.396 mmol) was added and stirring was continued for 3 h. The reaction was quenched with sat. aq. $NaHCO_3$ (0.5 mL). After removing the solvent under argon, the crude product was then subjected to preparative HPLC (Kromasil-5-C18, 5 µm, 150 mm × 30 mm, MeOH:H_2O = 95:5, 35 mL/min, λ = 254 nm, t = 4.2 min) to afford the title compound as a white solid (165 mg, 67 %). $[\alpha]_D^{20}$ = −23.4 (c = 0.5, $CHCl_3$); ^1H NMR (400 MHz, $CDCl_3$): δ = 8.05 (d, *J* = 8.4 Hz, 1H), 8.00 (dd, *J* = 8.5, 1.3 Hz, 1H), 7.62 (ddd, *J* = 8.3, 6.9, 1.4 Hz, 1H), 7.49 (ddd, *J* = 8.3, 6.8, 1.3 Hz, 1H), 5.82 (dd, *J* = 6.5, 1.8 Hz, 1H), 4.72 (s, 1H), 3.49 (d, *J* = 2.9 Hz, 4H), 3.37 (d, *J* = 1.7 Hz, 1H), 3.31 (dt, *J* = 13.2, 7.2 Hz, 1H), 3.20–3.10 (m, 4H), 3.03 (ddd, *J* = 20.0, 7.8, 3.1 Hz, 2H), 2.49 (ddd, *J* = 11.5, 8.6, 7.0 Hz, 1H), 2.29–2.21 (m, 4H), 2.20–2.02 (m, 10H), 2.02–1.97 (m, 1H), 1.95–1.88 (m, 1H), 1.88–1.78 (m, 3H), 1.78–1.74 (m, 6H), 1.72–1.67 (m, 2H), 1.64 (dd, *J* = 5.8, 3.4 Hz, 6H), 1.48–1.40 (m, 14H), 1.39–1.28 (m, 6H) ppm; ^{13}C NMR (101 MHz, $CDCl_3$): δ = 171.8, 163.3, 156.0, 147.1, 146.1, 144.7, 129.5, 129.2, 126.2, 125.9, 123.8, 122.3, 117.7, 95.6, 79.8, 79.4, 79.2, 79.1, 76.1, 75.5, 75.3, 74.9, 62.5, 58.0, 55.6, 52.2, 47.7, 45.3, 44.2, 40.4, 39.1, 37.9, 35.0, 30.8, 29.7, 29.1, 28.8, 28.8, 28.7, 28.4, 27.0, 26.8, 26.2, 26.1, 18.7, 18.6, 14.7, 4.9, 3.5, 3.5 ppm; IR (film) $\tilde{\nu}$ = 3319, 2929, 2857, 1708, 1627, 1568, 1496, 1436, 1404, 1390, 1365, 1272, 1251, 1170, 1074, 1027, 957, 871, 759, 666, 593cm^{-1}; MS (ESI): *m/z*: 825 [M+H$^+$], 847 [M+Na$^+$]; HRMS (ESI): *m/z:* calcd. for $C_{54}H_{73}N_4O_3$ [M+H$^+$]: 825.56772, found: 825.56785.

Compound 73. A flame-dried two-necked flask connected to a reflux condenser was charged with activated 5 Å molecular sieves (powder, 400 mg) and toluene (11 mL). The suspension was purged with argon at room temperature for 30 min. Next, the mixture was stirred at 110 °C for 30 min before a solution of tetrayne **72** (20.0 mg, 0.024 mmol) in toluene (0.9 mL) was added. In a separate flame-dried Schlenk tube under argon, Mo-complex **29** (9.7 mg, 0.015 mmol) was dissolved in toluene (0.5 mL) and transferred via syringe into another Schlenk tube containing the trisilanol **30** (12.4 mg, 0.016 mmol). The resulting mixture was stirred for 30 s, before it was added dropwise to the suspension of the substrate and the molecular sieves in toluene at 110 °C. The mixture was stirred at 110 °C for 30 min, before the reaction was quenched by the addition of ethanol (1 mL). The mixture was cooled to room temperature and filtered through a plug of Celite, which was carefully rinsed with EtOAc. The combined filtrates were evaporated *in vacuo* and the residue purified by preparative HPLC (Kromasil-5-C18, 5 µm, 150 mm × 30 mm, MeOH, 35 mL/min, λ = 230 nm, major product, t = 4.8 min; minor product, t = 4.0 min) to afford the title compound **73** (6.1 mg, 35% yield) and an isomer **S18** (3.0 mg, 17% yield) as a white solid each.

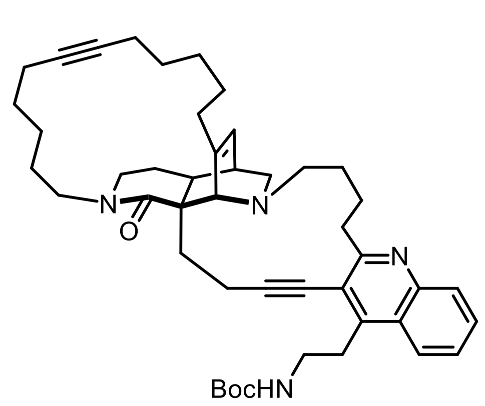

Analytical and spectral data of compound **73**: $[\alpha]_D^{20}$ = –7.3 (c = 0.31, $CHCl_3$); ^{1}H NMR (600 MHz, [D_4]-MeOH): δ = 8.18 (d, *J* = 8.4 Hz, 1H), 7.93 (d, *J* = 8.5 Hz, 1H), 7.69 (ddd, *J* = 8.3, 6.9, 1.3 Hz, 1H), 7.57 (t, *J* = 7.6 Hz, 1H), 5.94–5.85 (m, 1H), 3.95 (ddd, *J* = 13.1, 8.7, 5.9 Hz, 1H), 3.66 (d, *J* = 1.7 Hz, 1H), 3.55–3.48 (m, 1H), 3.49–3.40 (m, 3H), 3.37 (t, *J* = 7.2 Hz, 1H), 3.26 (t, *J* = 12.5 Hz, 1H), 3.22–3.17 (m, 1H), 2.99 (dd, *J* = 18.4, 4.5 Hz, 1H), 2.93 (dd, *J* = 9.5, 2.1 Hz, 1H), 2.84 (t, *J* = 11.9 Hz, 1H), 2.68 – 2.60 (m, 1H), 2.59–2.48 (m, 3H), 2.40–2.31 (m, 3H), 2.25–2.11 (m, 6H), 2.09–2.02 (m, 1H), 1.92 (ddd, *J* = 9.8, 6.8, 3.5 Hz, 1H), 1.79 (dd, *J* = 9.6, 2.6 Hz, 1H), 1.76–1.64 (m, 3H), 1.63–1.57 (m, 2H), 1.55–1.49 (m, 3H), 1.49–1.41 (m, 5H), 1.41–1.34 (m, 9H), 1.31–1.22 (m, 2H), 1.19–1.16 (m, 2H) ppm; ^{13}C NMR (126 MHz, [D_4]-MeOH): δ = 174.3, 165.6, 158.4, 149.1, 146.7, 146.0, 130.7, 129.0, 127.7, 127.2, 125.4, 122.4, 119.3, 103.8, 81.9, 80.4, 80.0, 76.6, 62.9, 56.3, 54.9, 53.5, 45.0, 41.2, 38.9, 38.6, 38.3, 36.7, 32.1, 31.4, 30.2, 28.9, 28.8, 28.8, 28.7, 28.4, 28.4, 26.3, 25.4, 19.6, 18.7, 14.0 ppm; IR (film) $\tilde{\nu}$ = 2930, 2850, 1705, 1634, 1423, 1159, 759 cm^{-1}; MS (ESI)*: m/z*: 717 [*M*+H$^+$]; HRMS (ESI): *m/z:* calcd. for $C_{46}H_{61}N_4O_3$ [*M*+H$^+$]: 717.47382, found: 717.47373.

1H (CDCl3, 400.12 MHz)

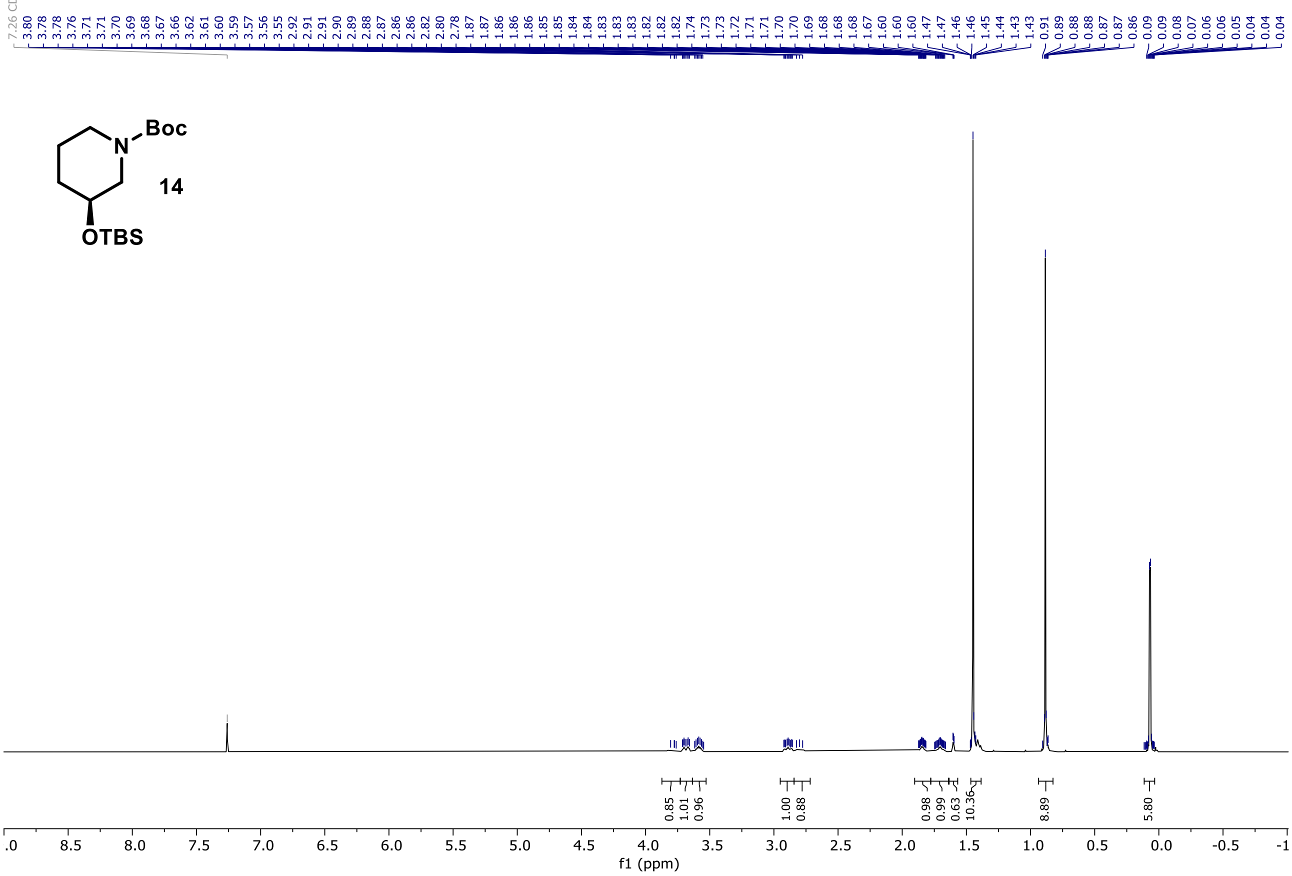

13C (CDCl3, 100.62 MHz)

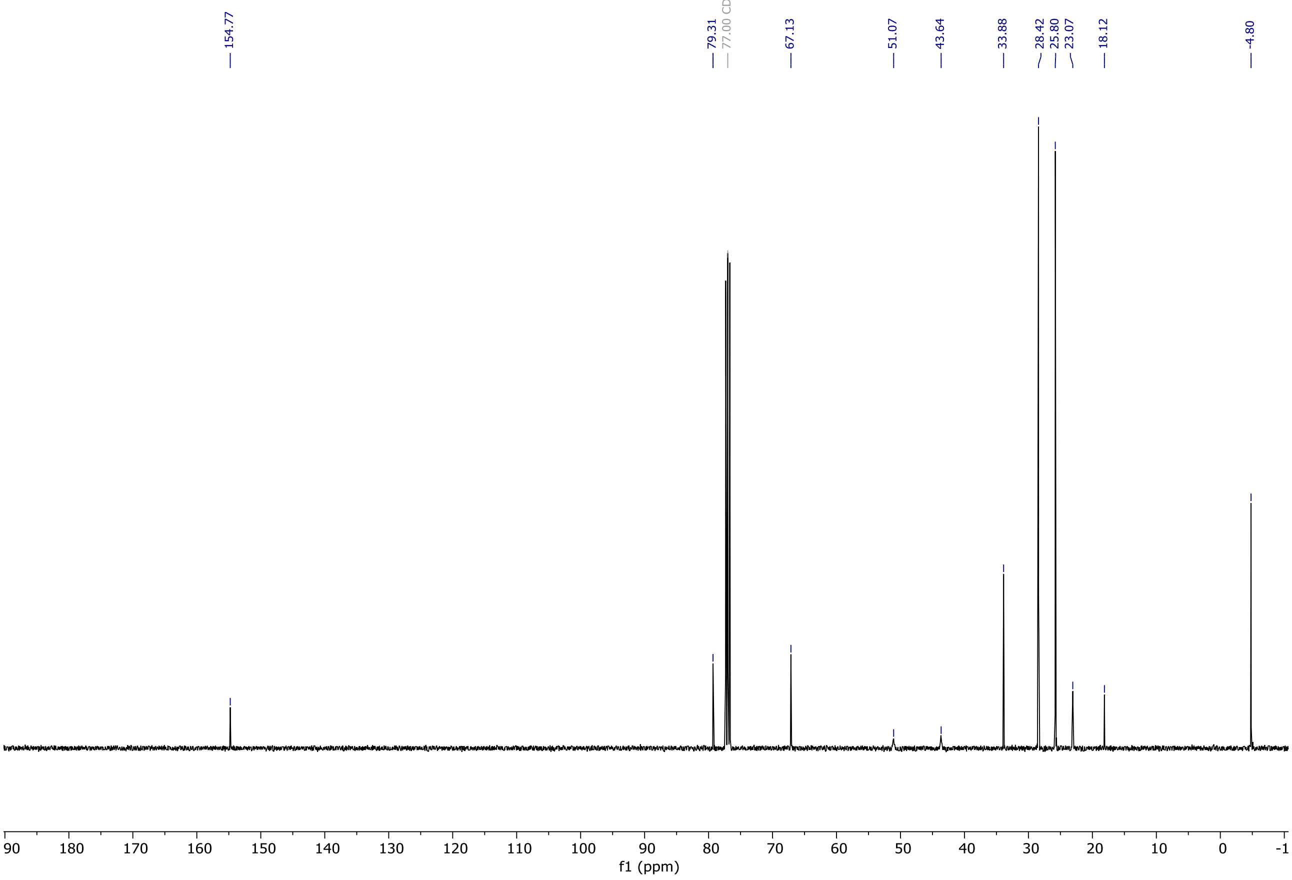

1H (CDCl3, 400.12 MHz)

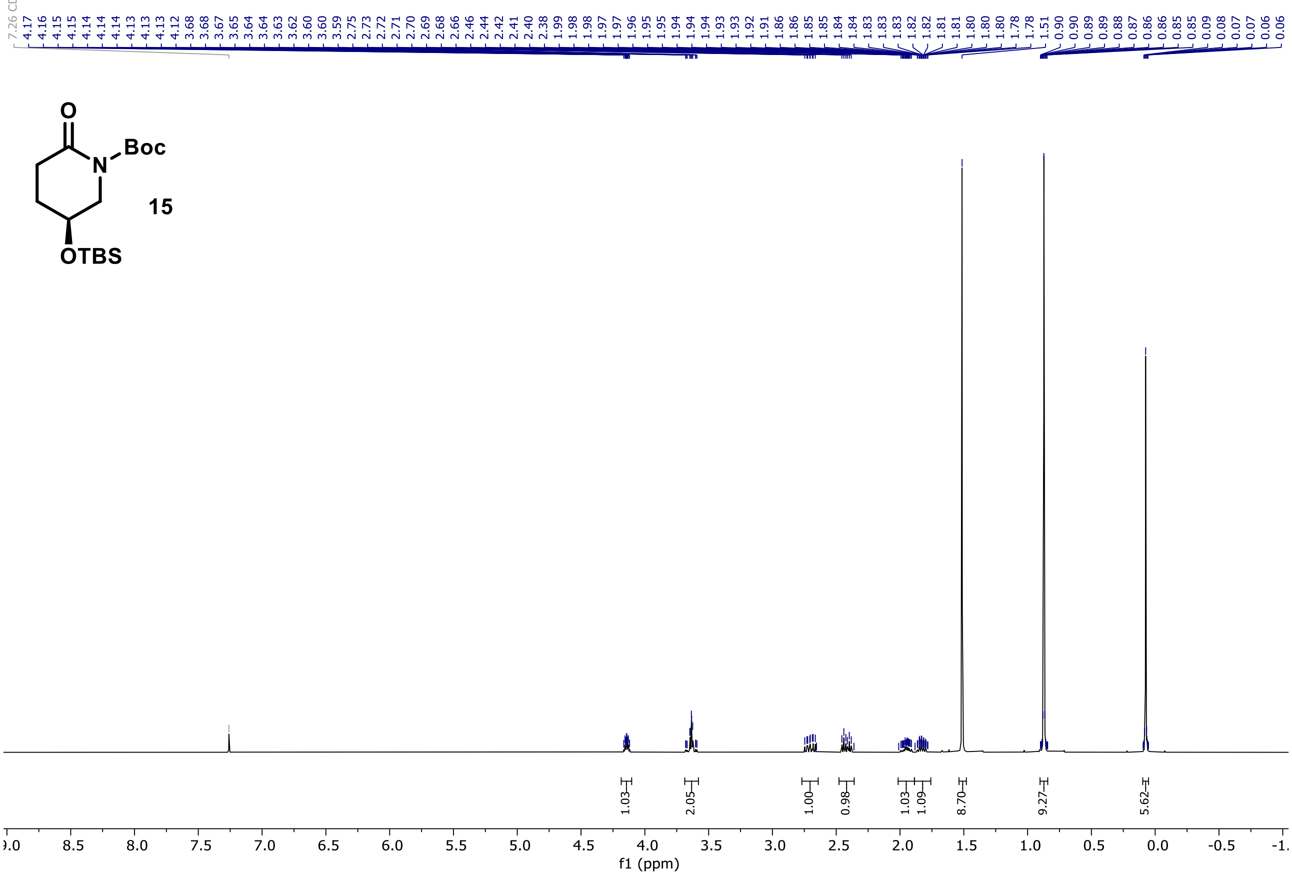

15

13C (CDCl3, 100.62 MHz)

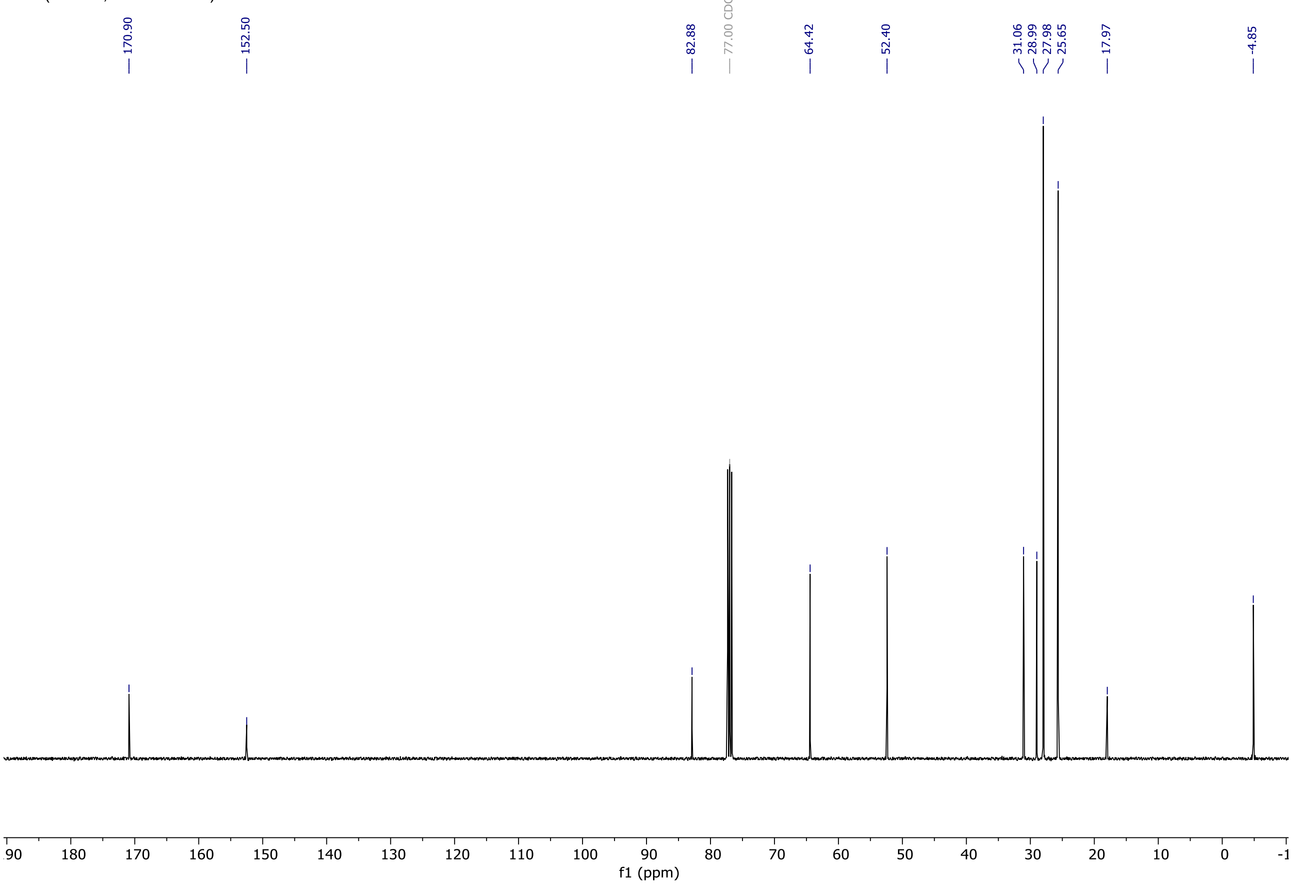

1H (CDCl3, 400.12 MHz)

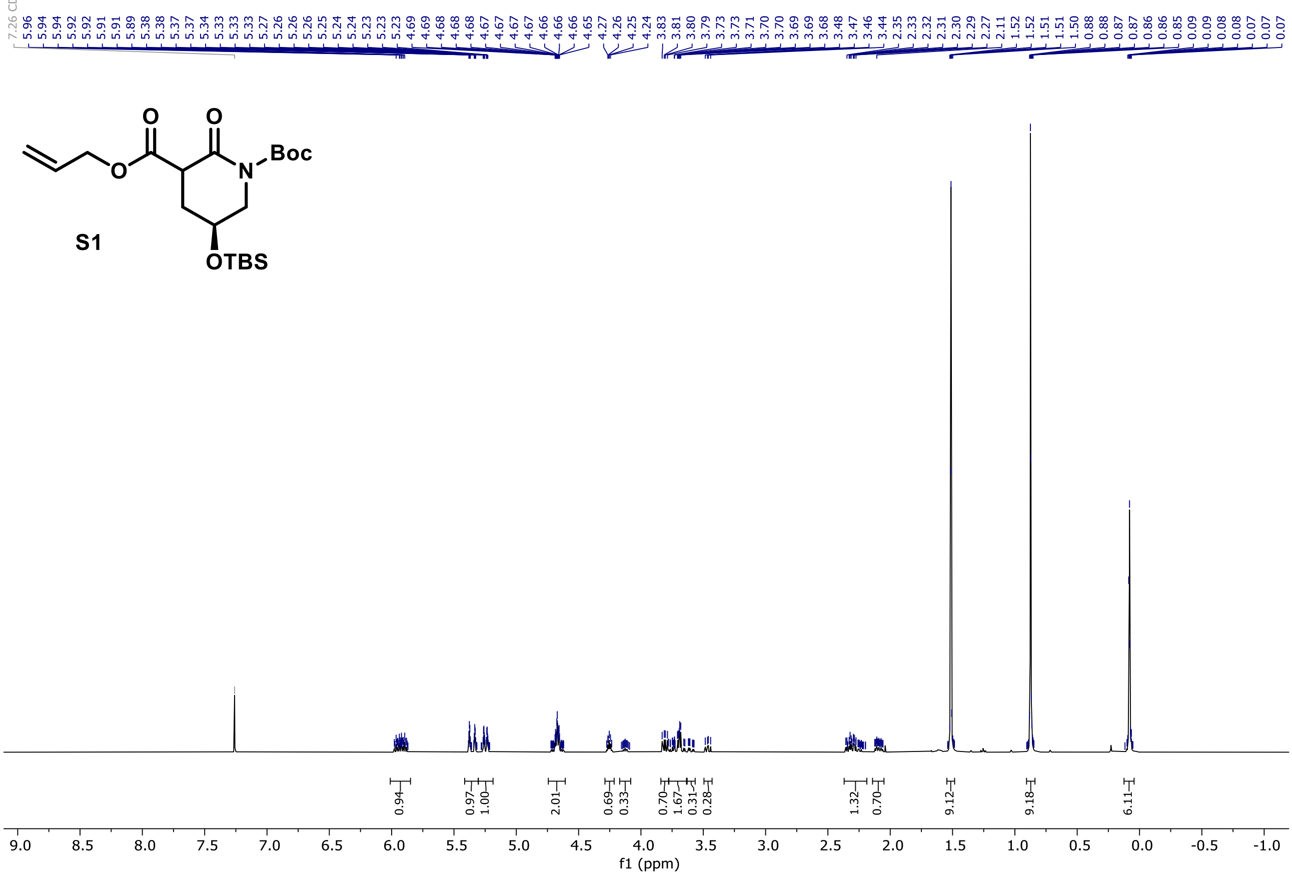

S1

13C (CDCl3, 100.62 MHz)

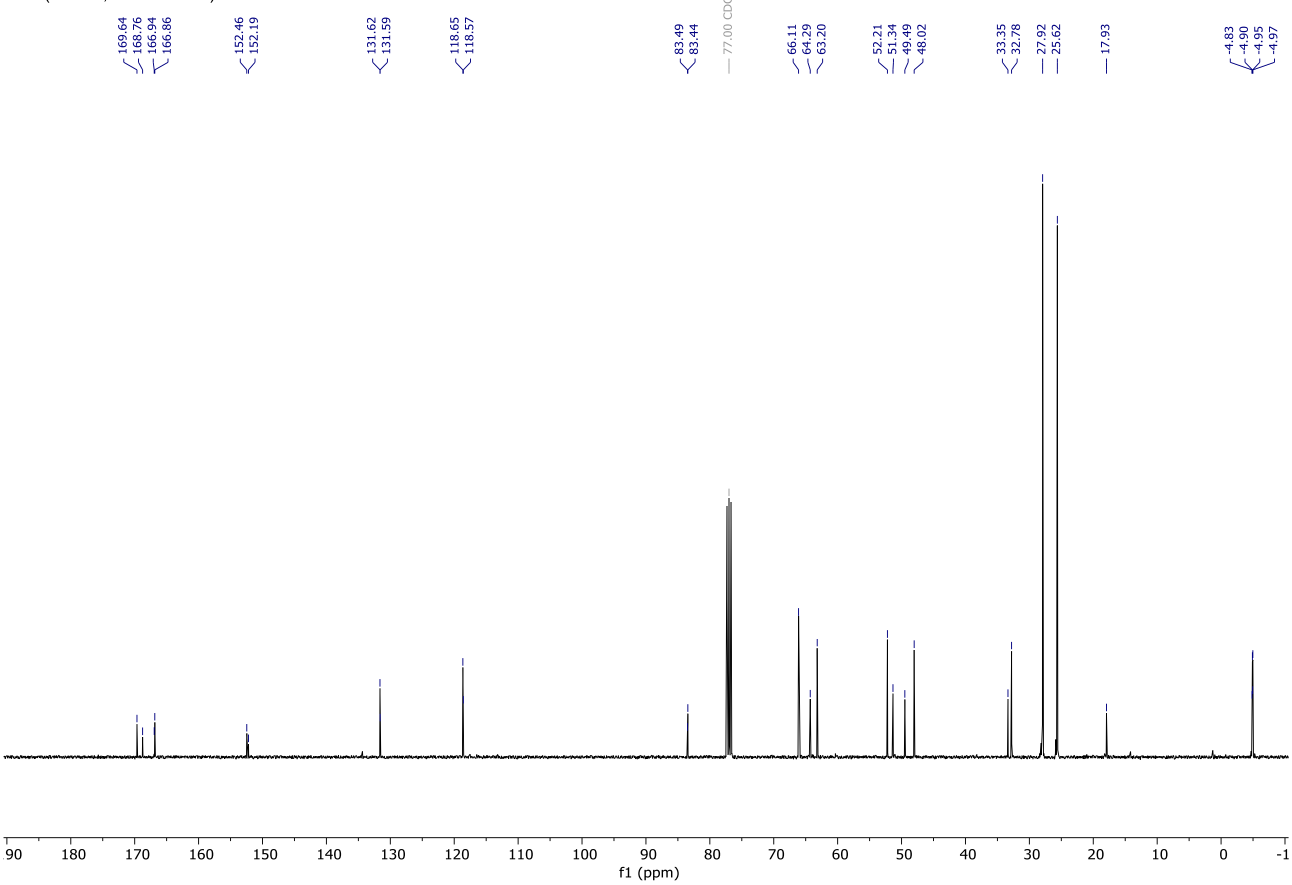

1H (CDCl3, 400.12 MHz)

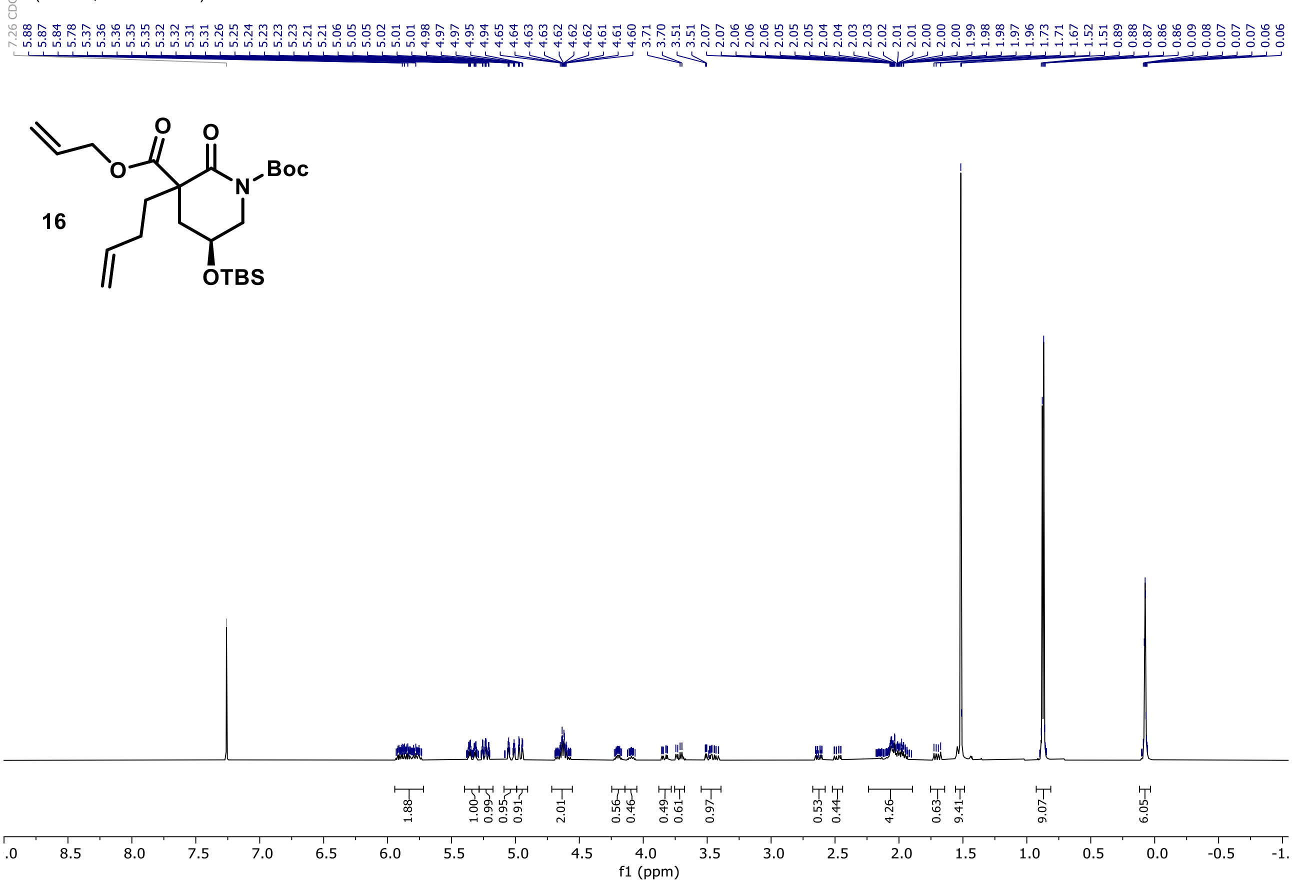

13C (CDCl3, 100.62 MHz)

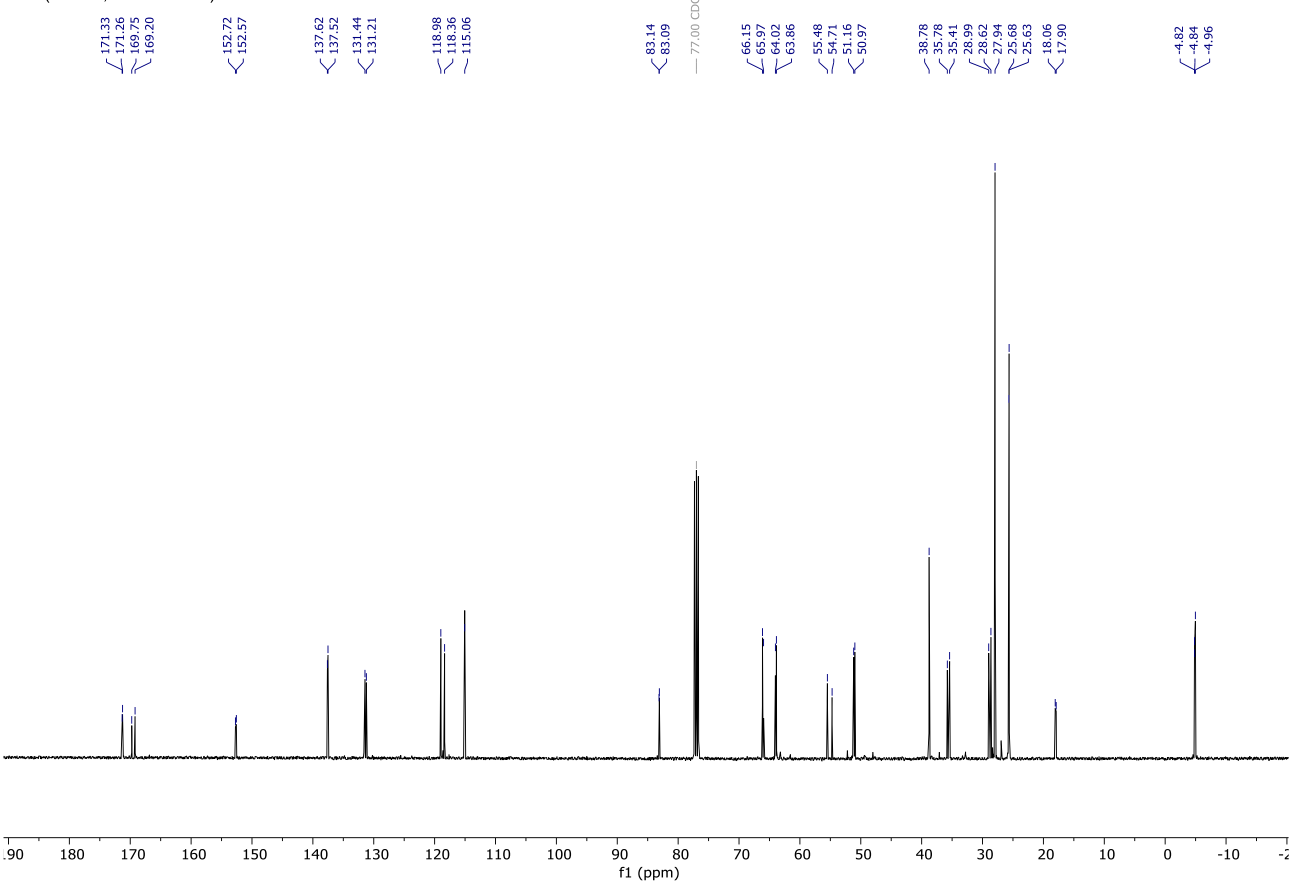

1H (CDCl3, 400.12 MHz)

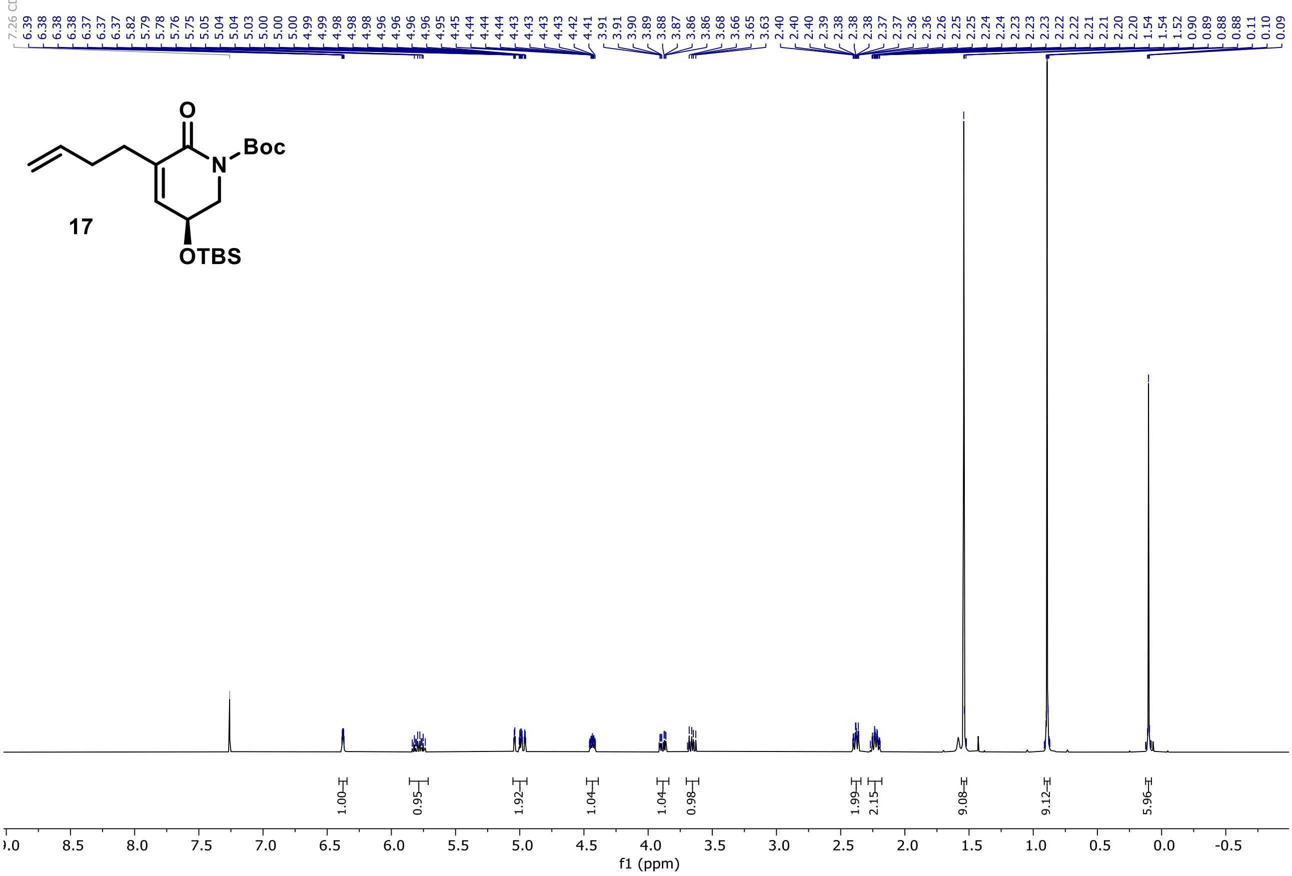

13C (CDCl3, 100.62 MHz)

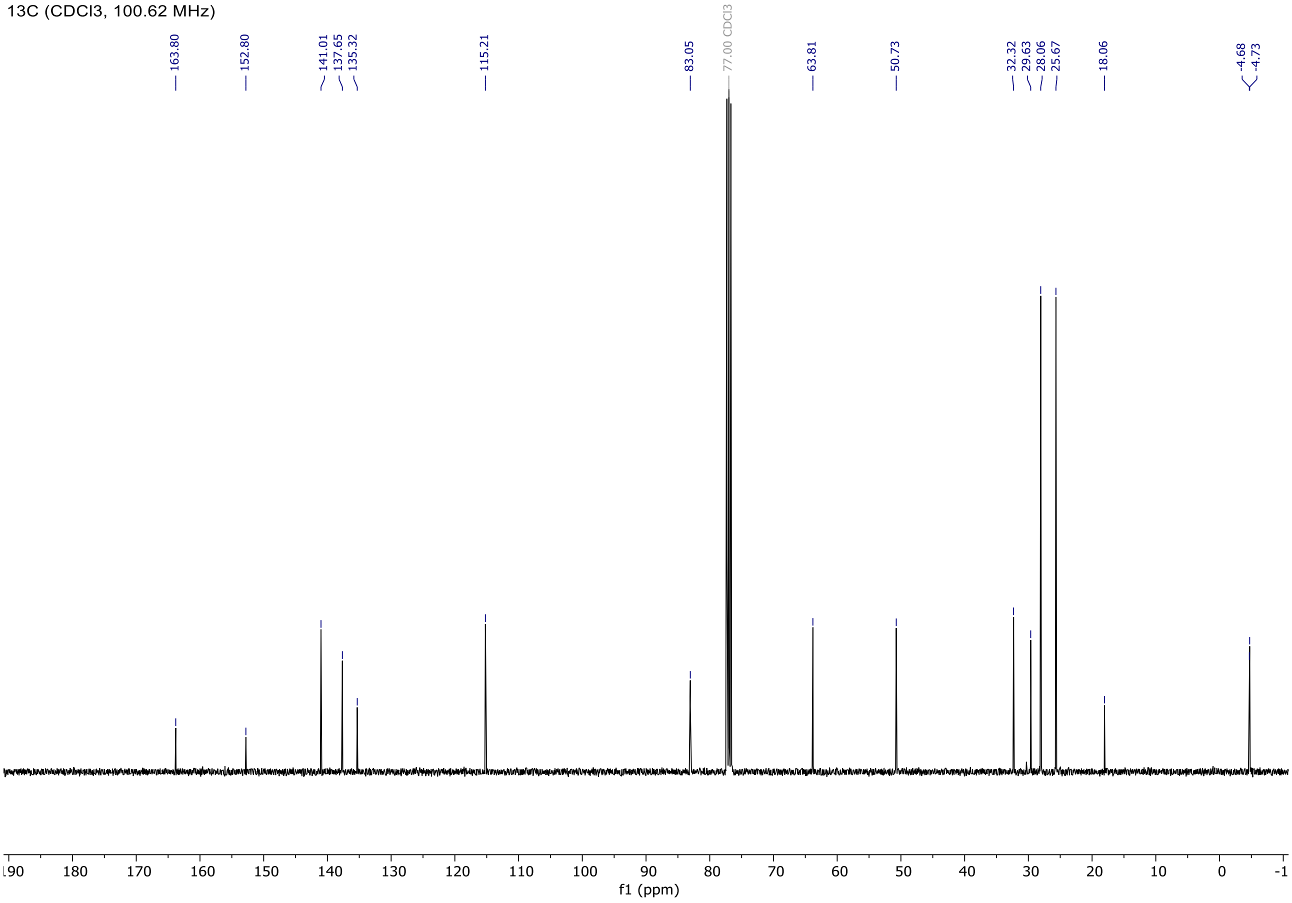

1H (CDCl3, 400.12 MHz)

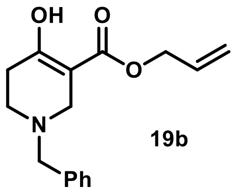

19b

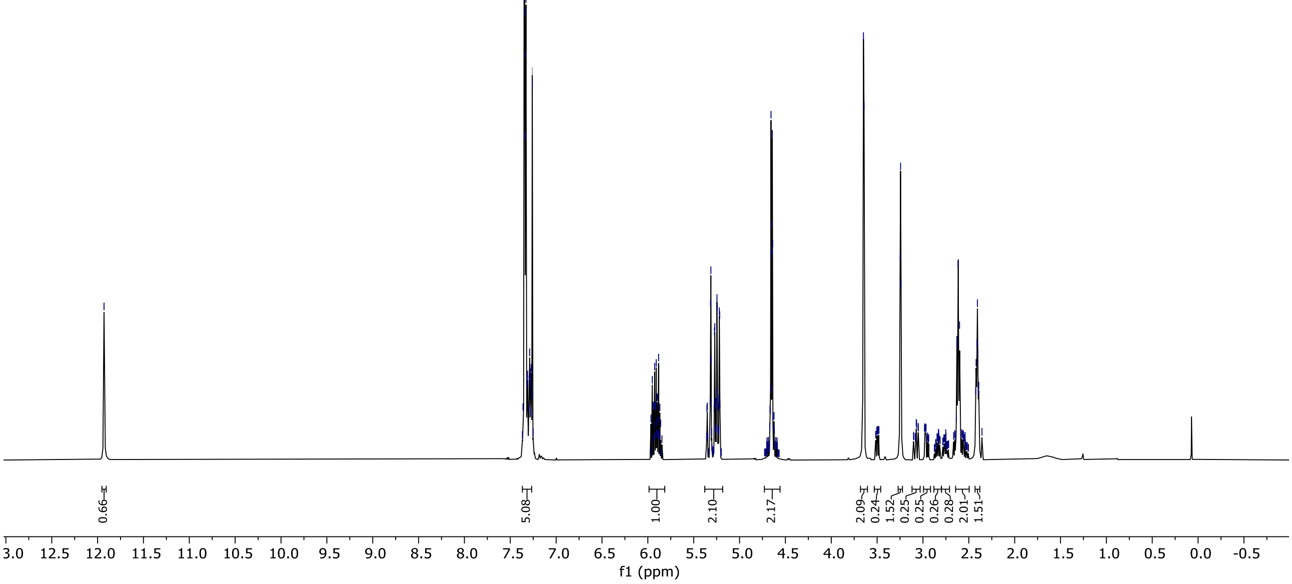

13C (CDCl3, 100.62 MHz)

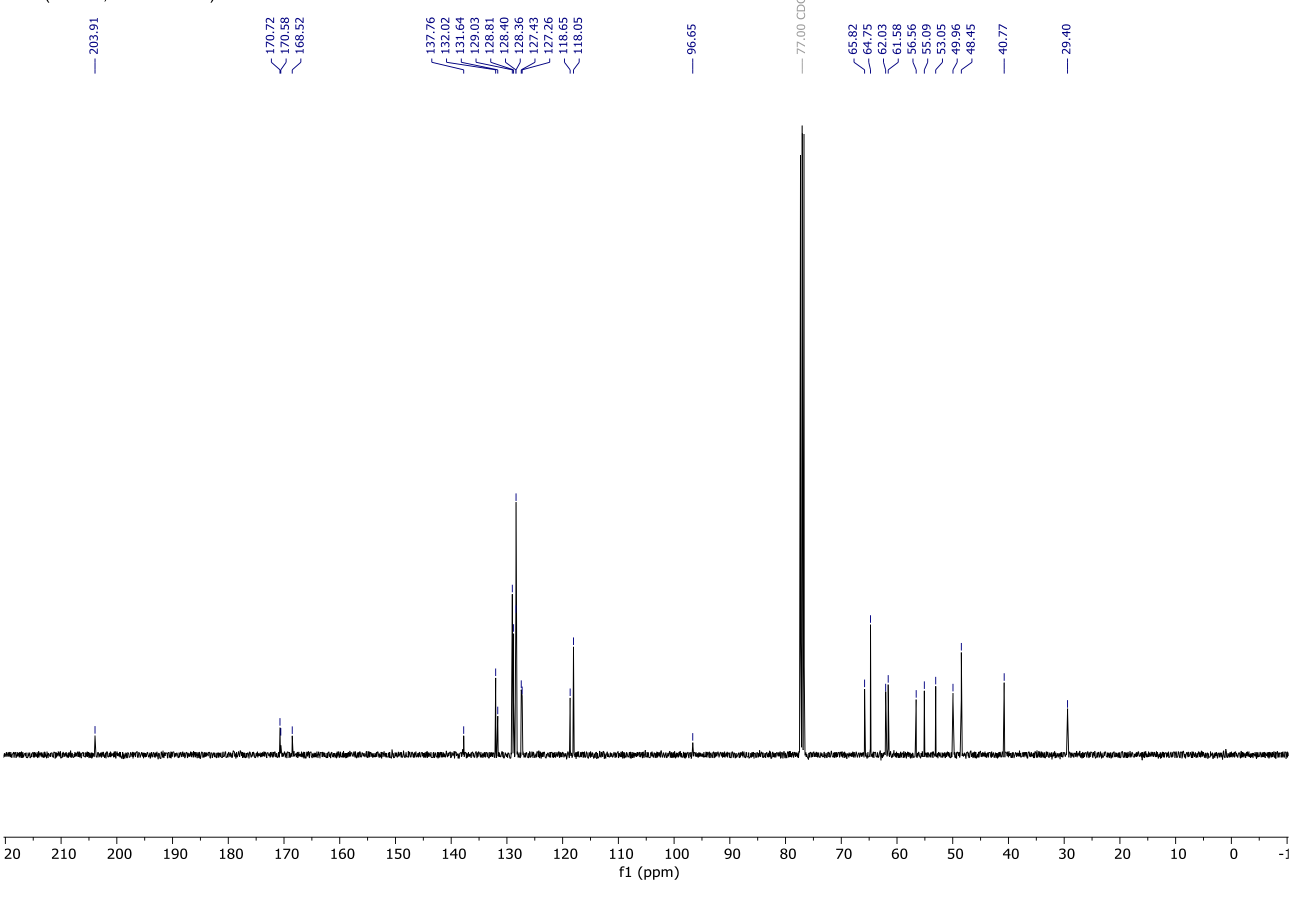

1H (CDCl3, 400.12 MHz)

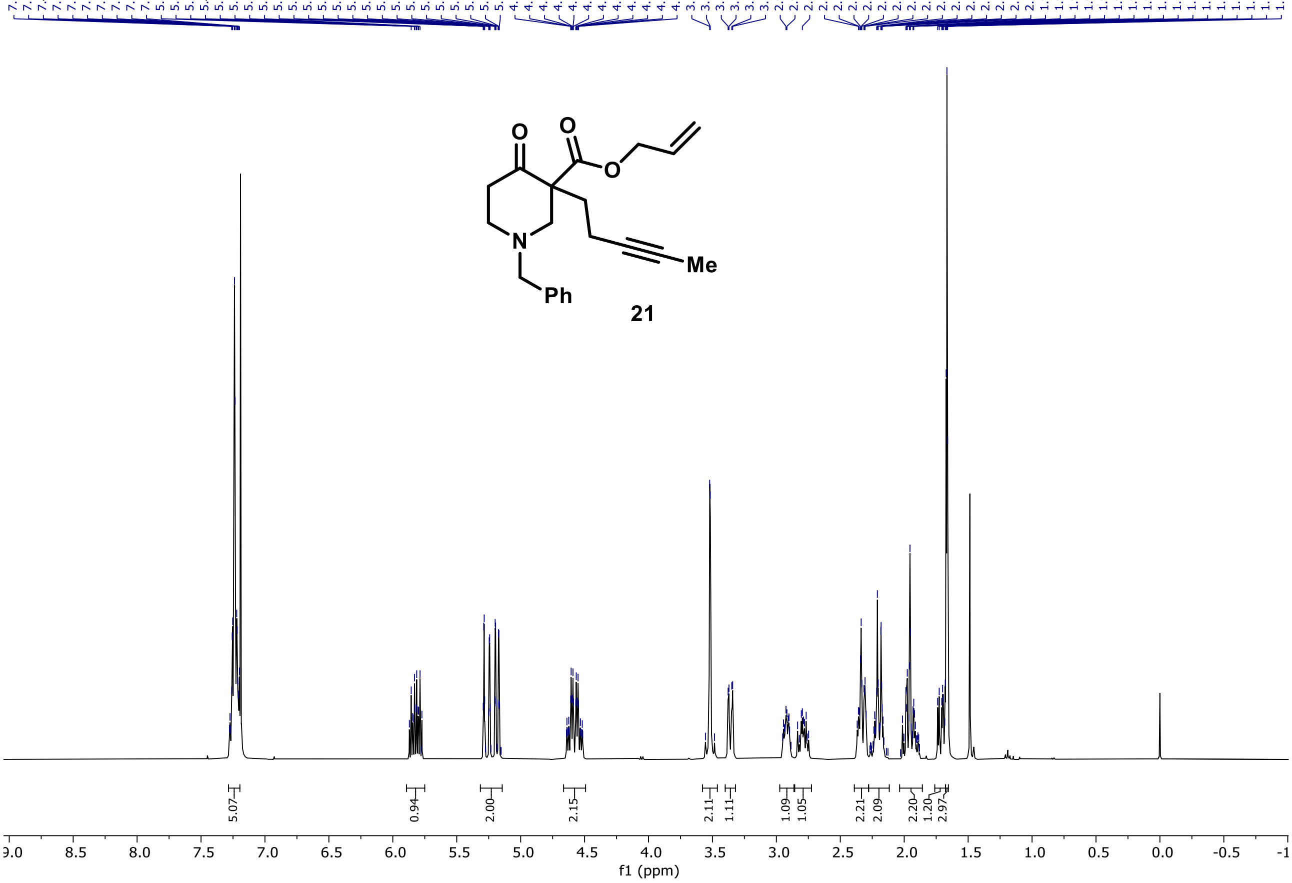

13C (CDCl3, 100.62 MHz)

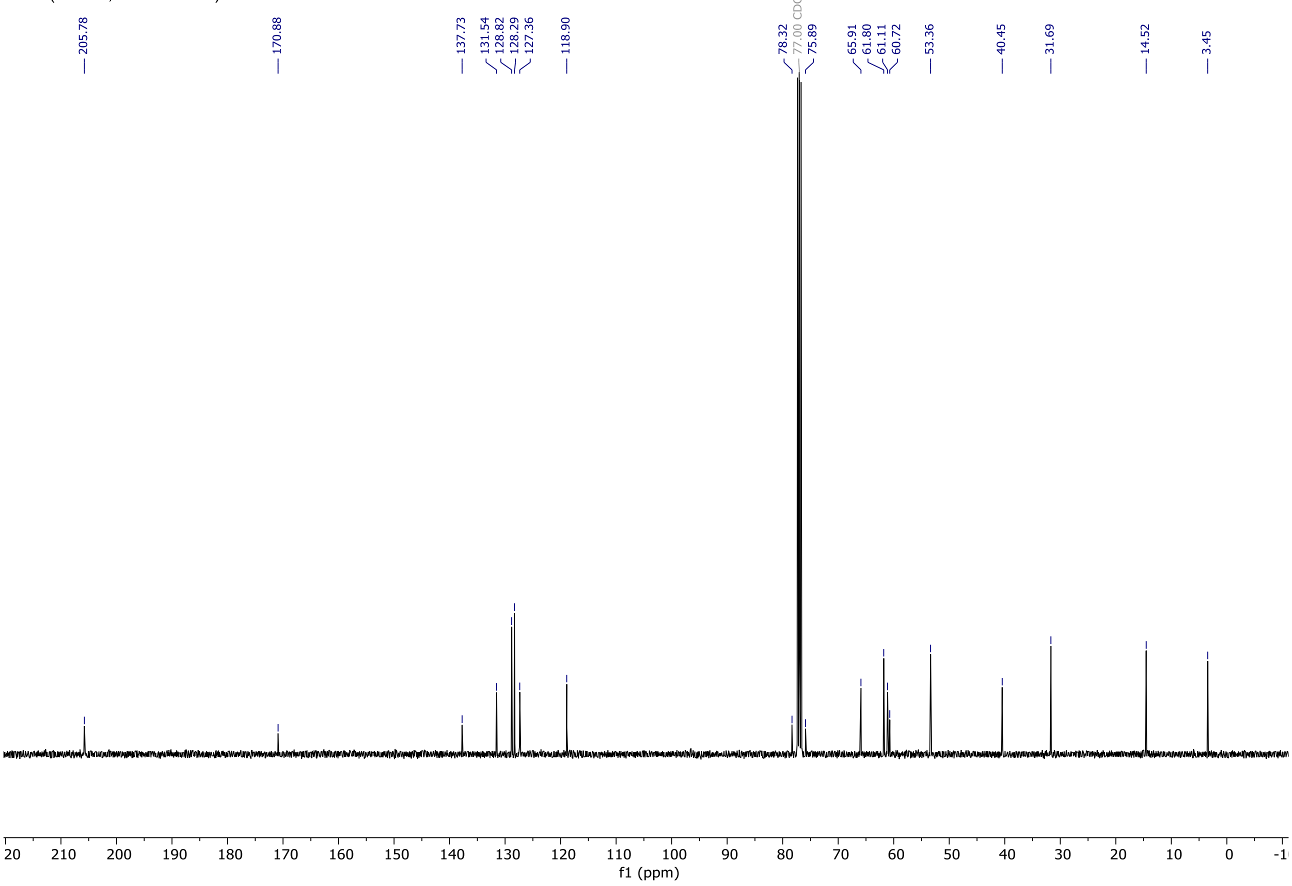

1H (CDCl3, 400.12 MHz)

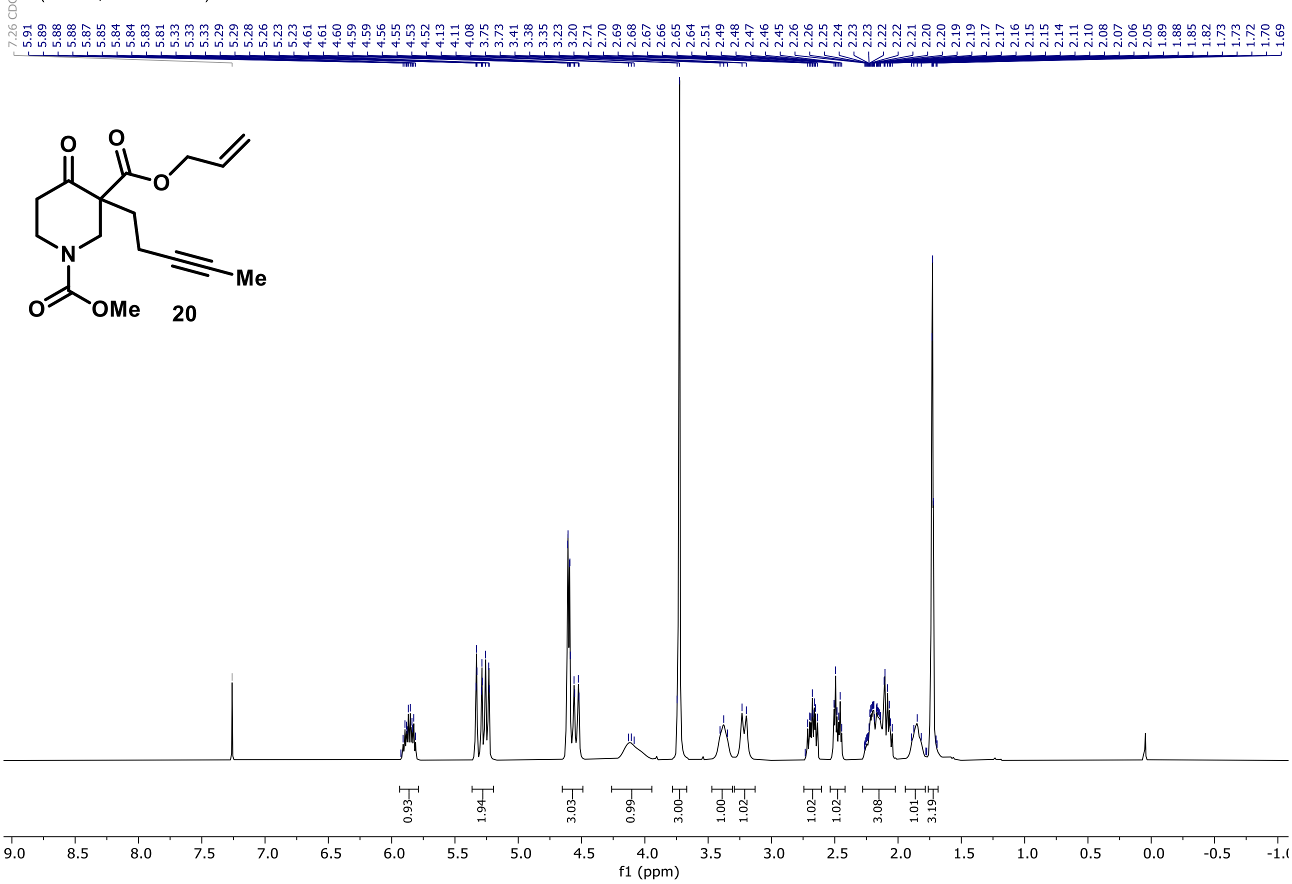

13C (CDCl3, 100.62 MHz)

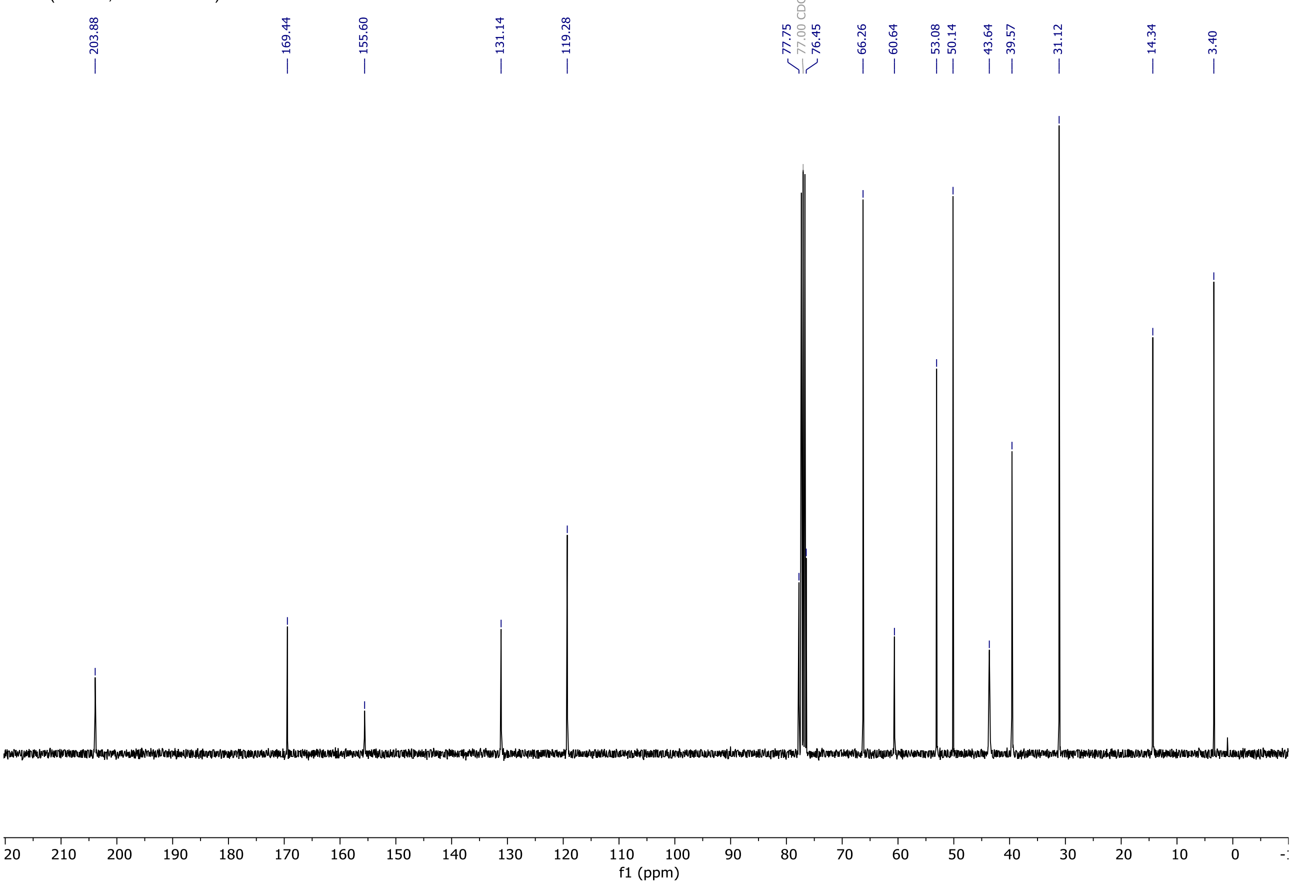

1H (CDCl3, 400.12 MHz)

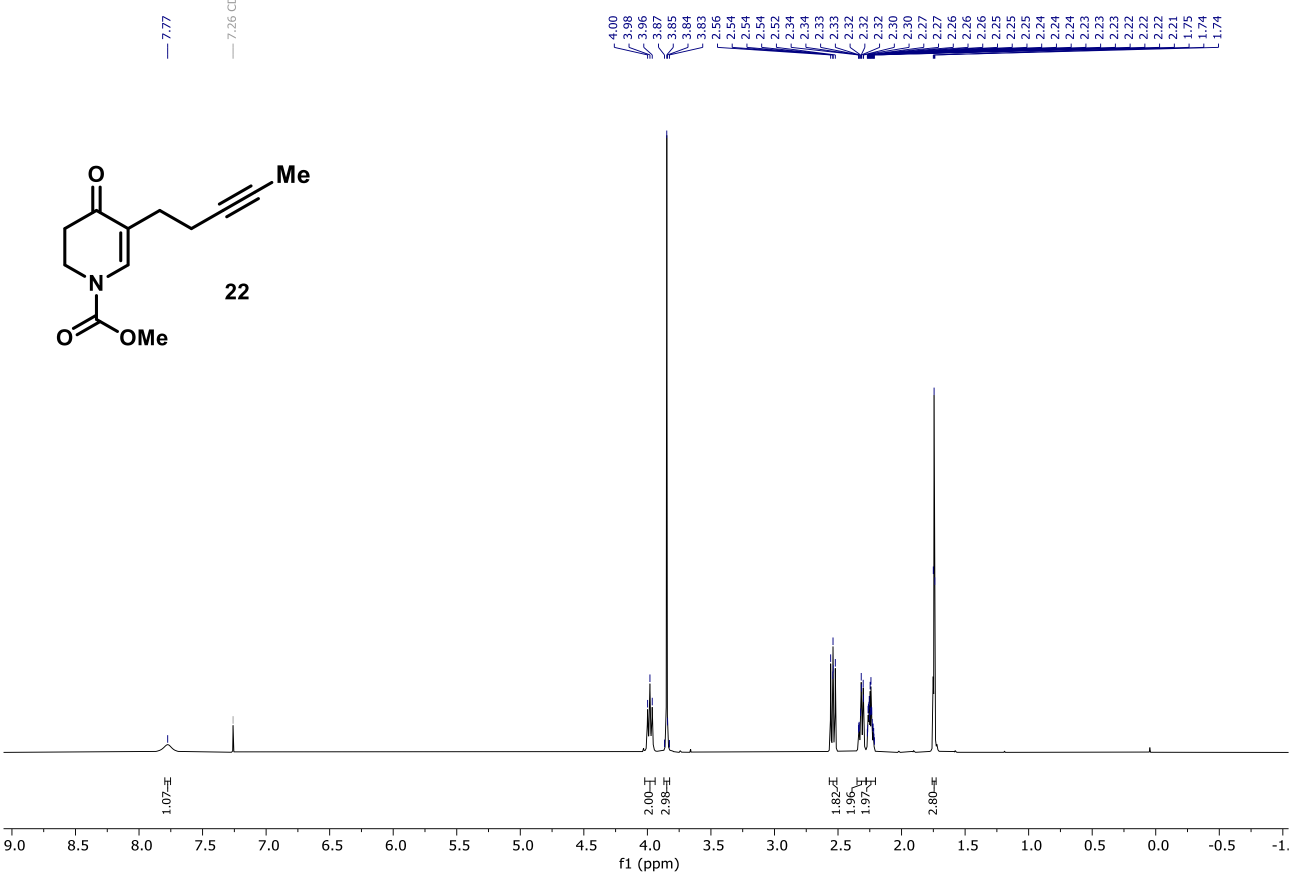

13C (CDCl3, 100.62 MHz)

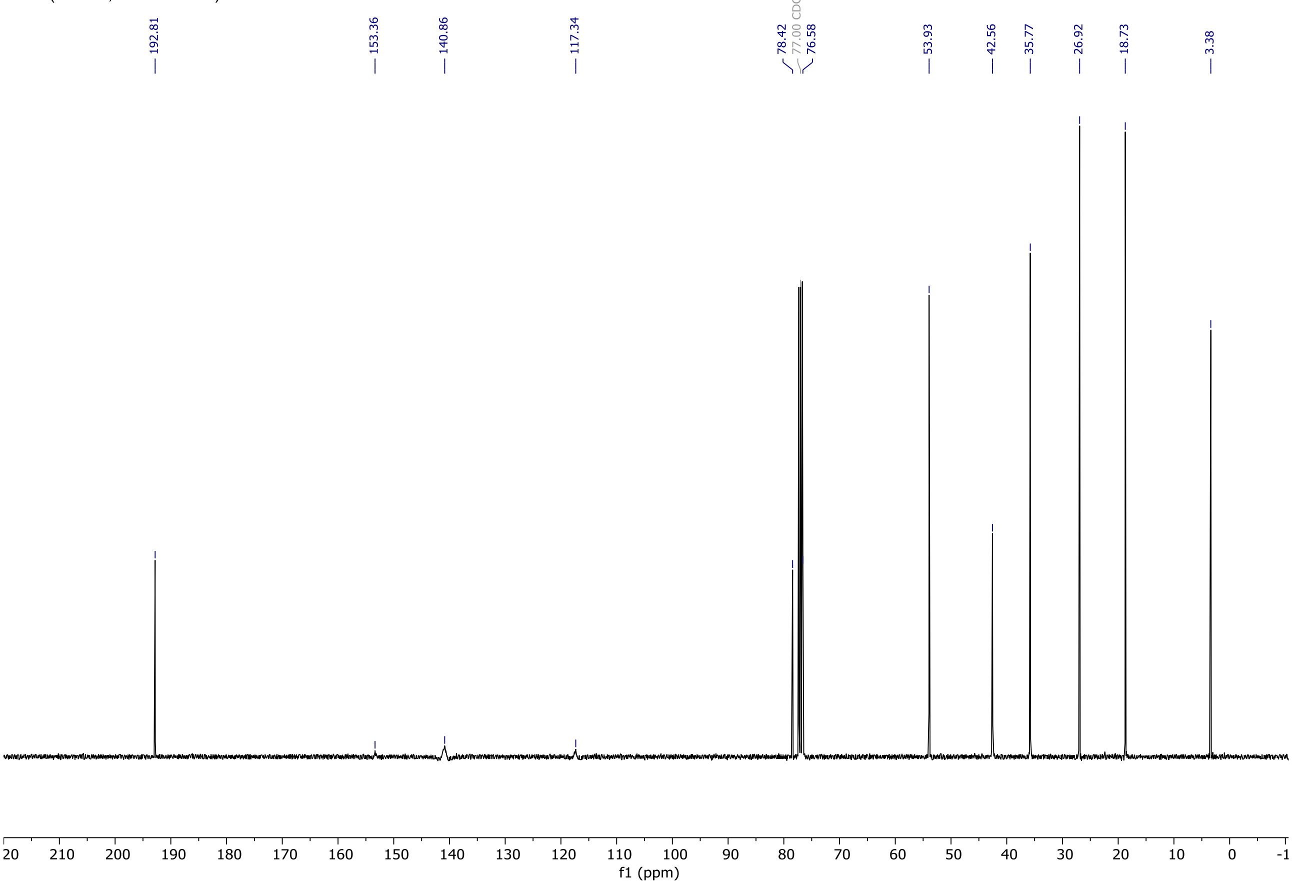

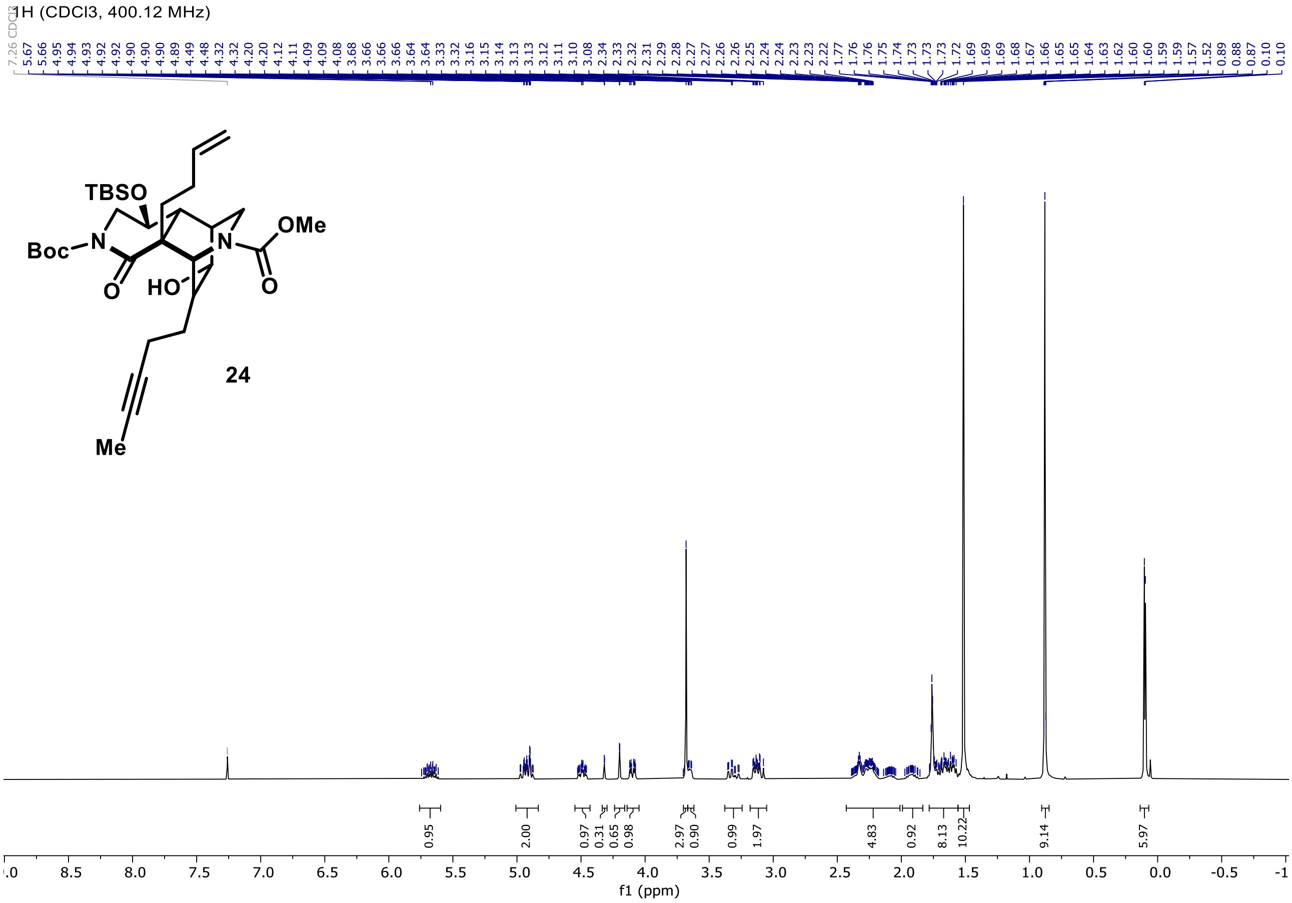

24

1H (CDCl3, 400.12 MHz)

13C (CDCl3, 100.62 MHz)

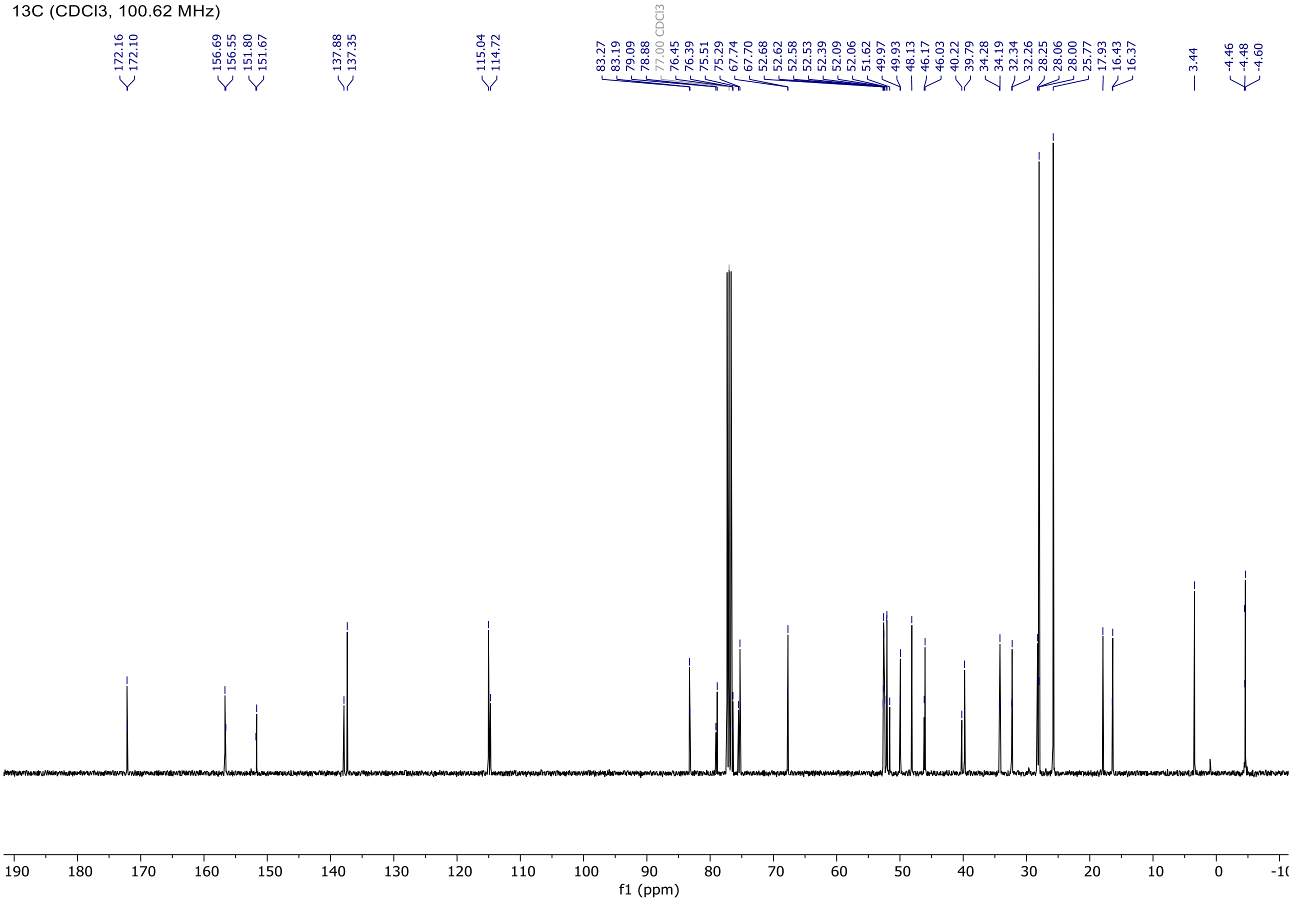

1H (CDCl3, 400.12 MHz)

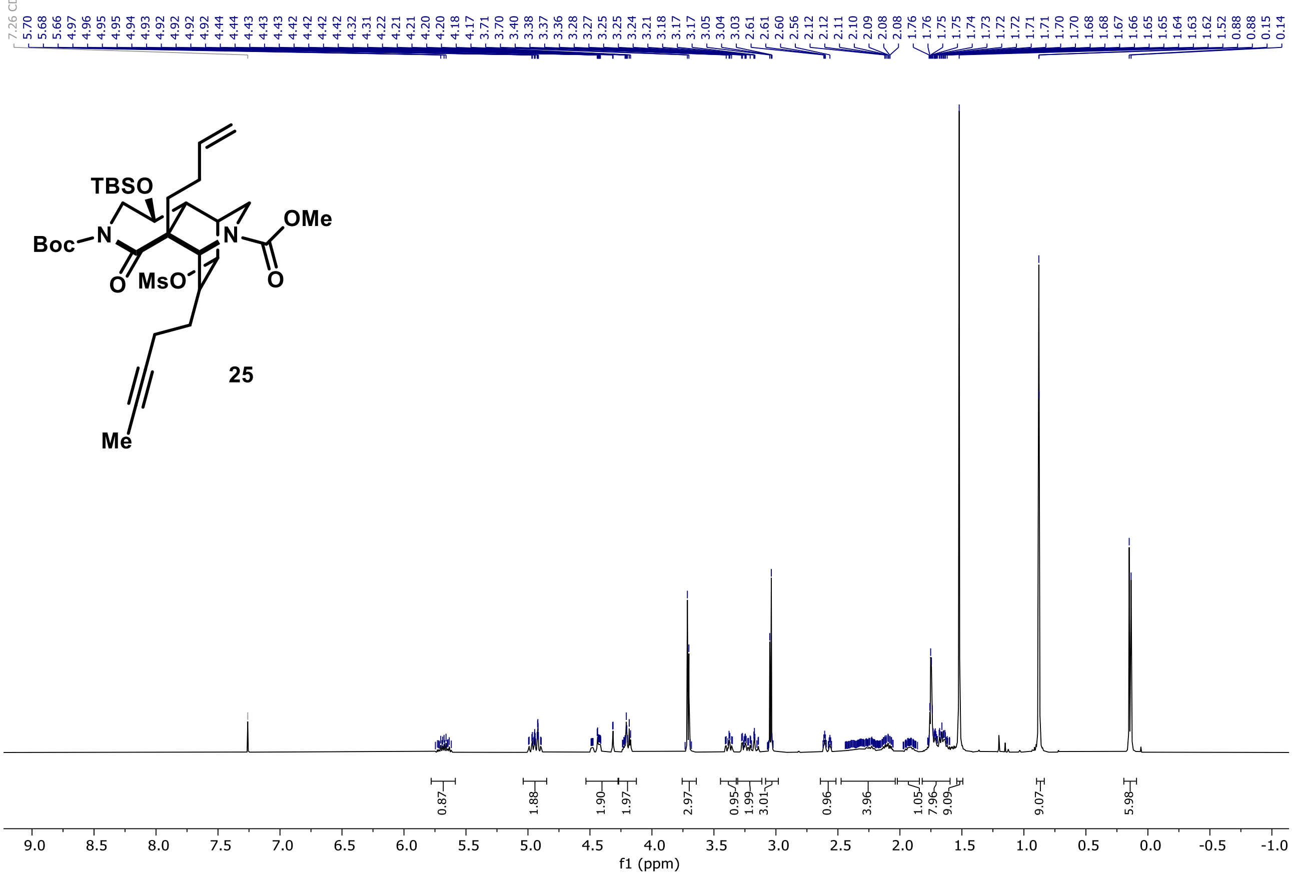

13C (CDCl3, 100.62 MHz)

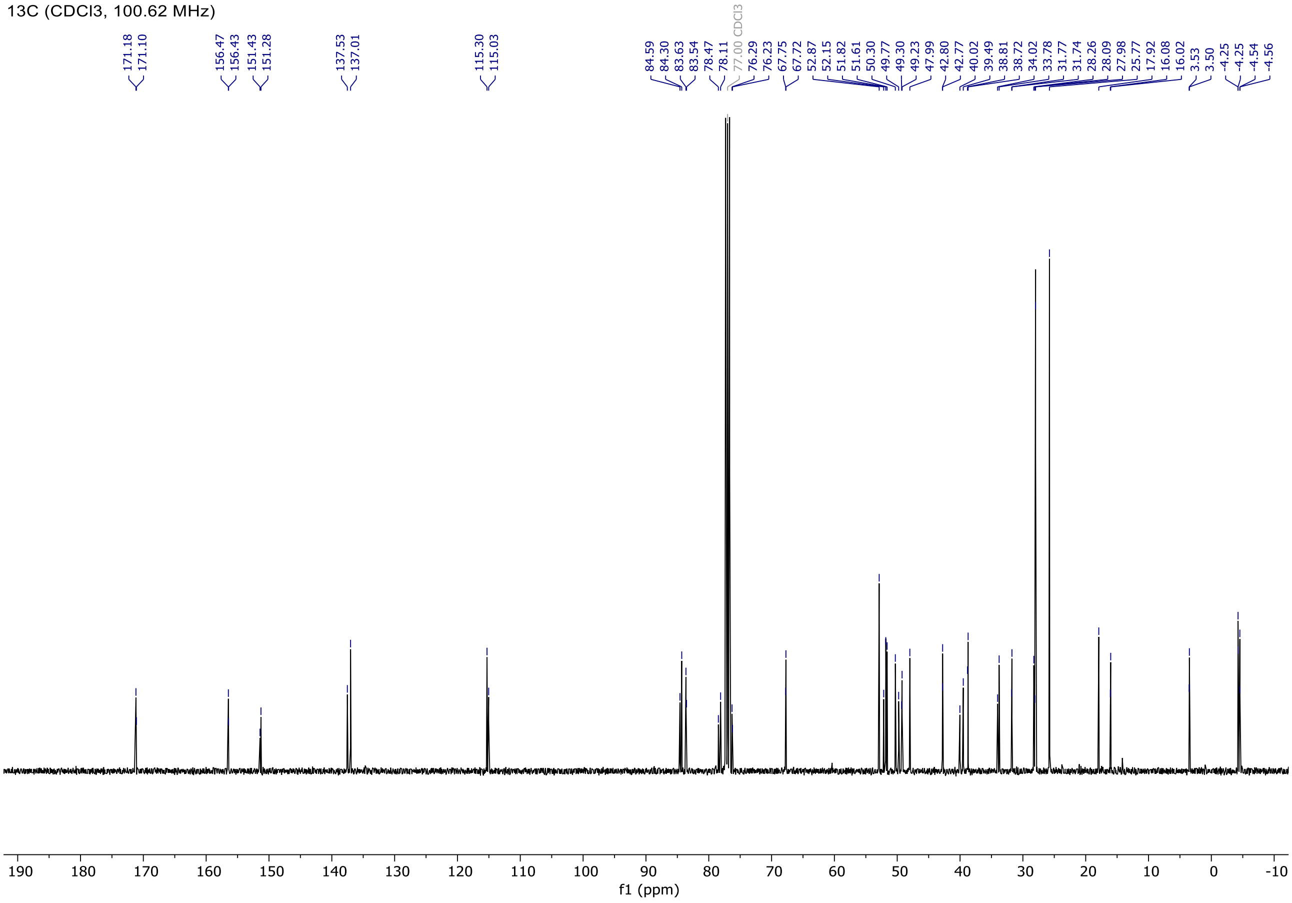

1H (CDCl3, 400.12 MHz)

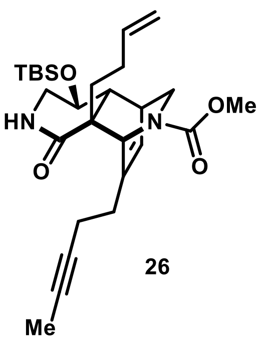

26

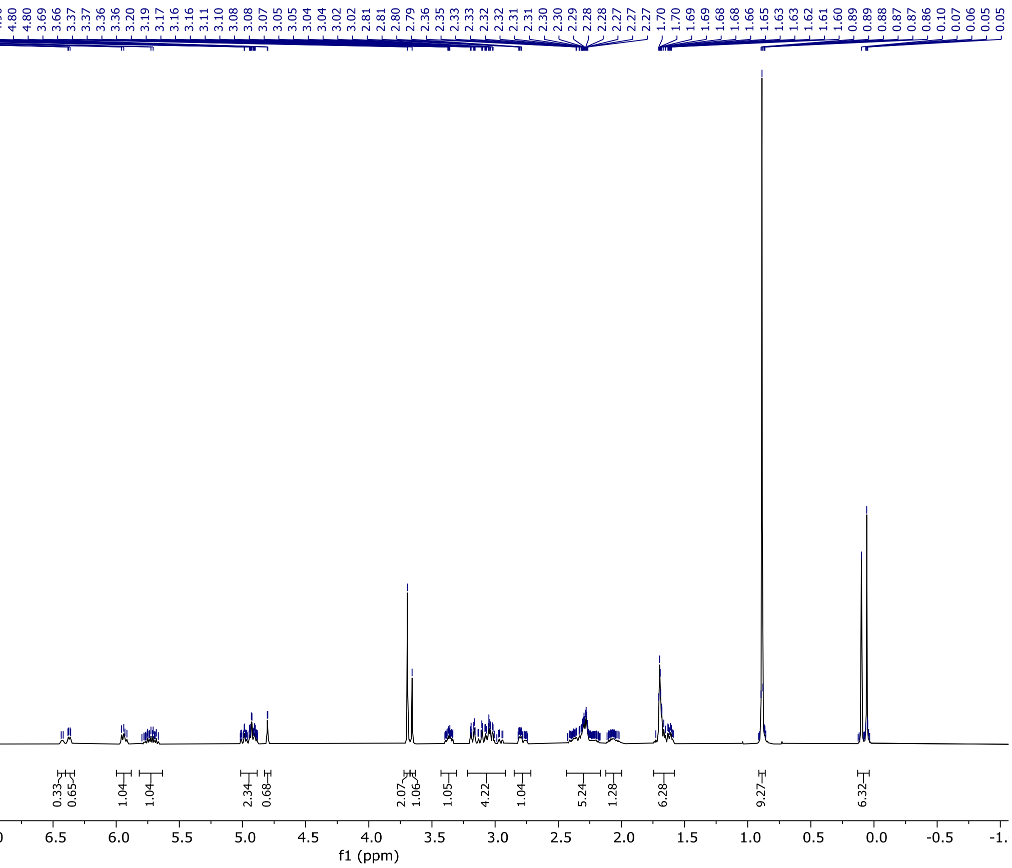

13C (CDCl3, 100.62 MHz)

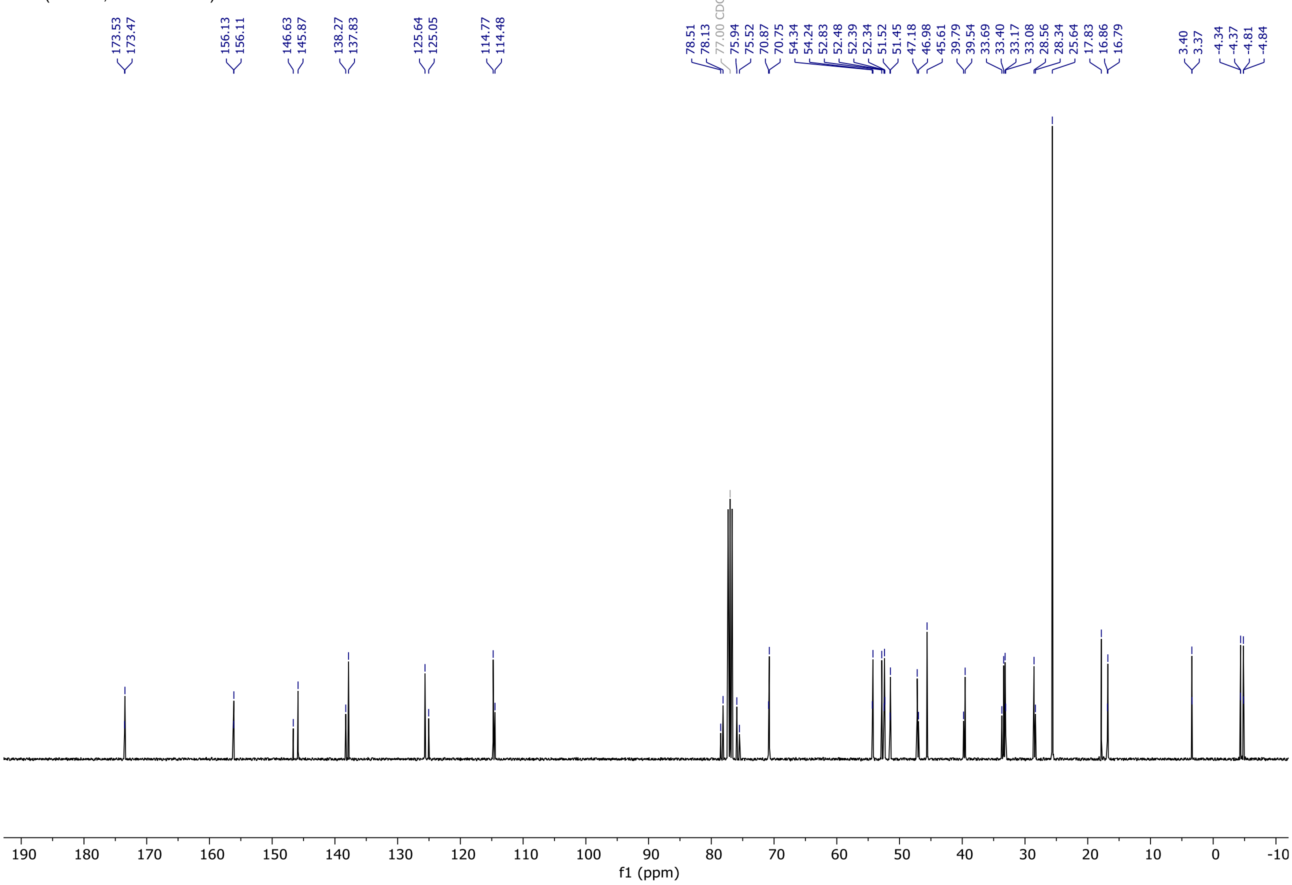

1H (CDCl3, 400.12 MHz)

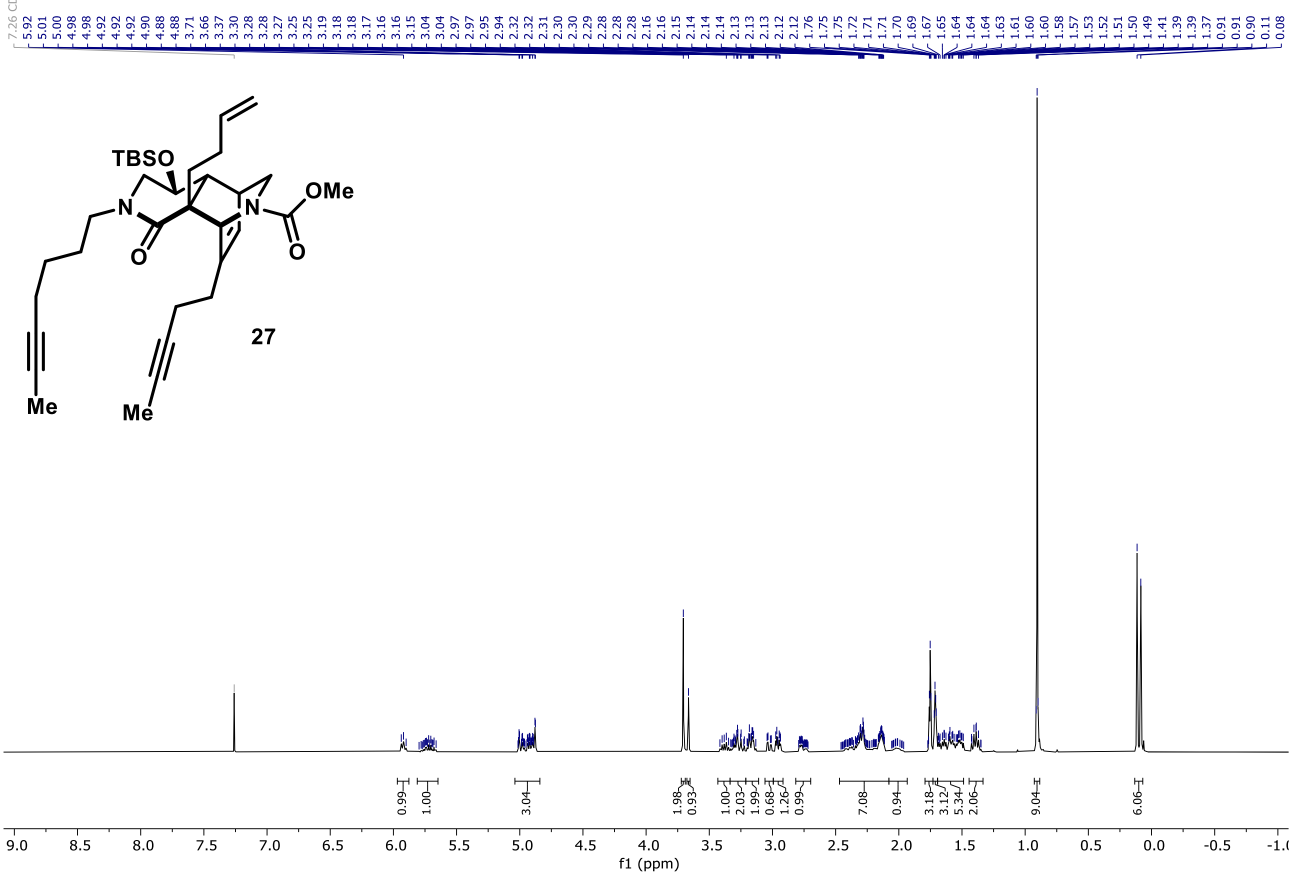

13C (CDCl3, 100.62 MHz)

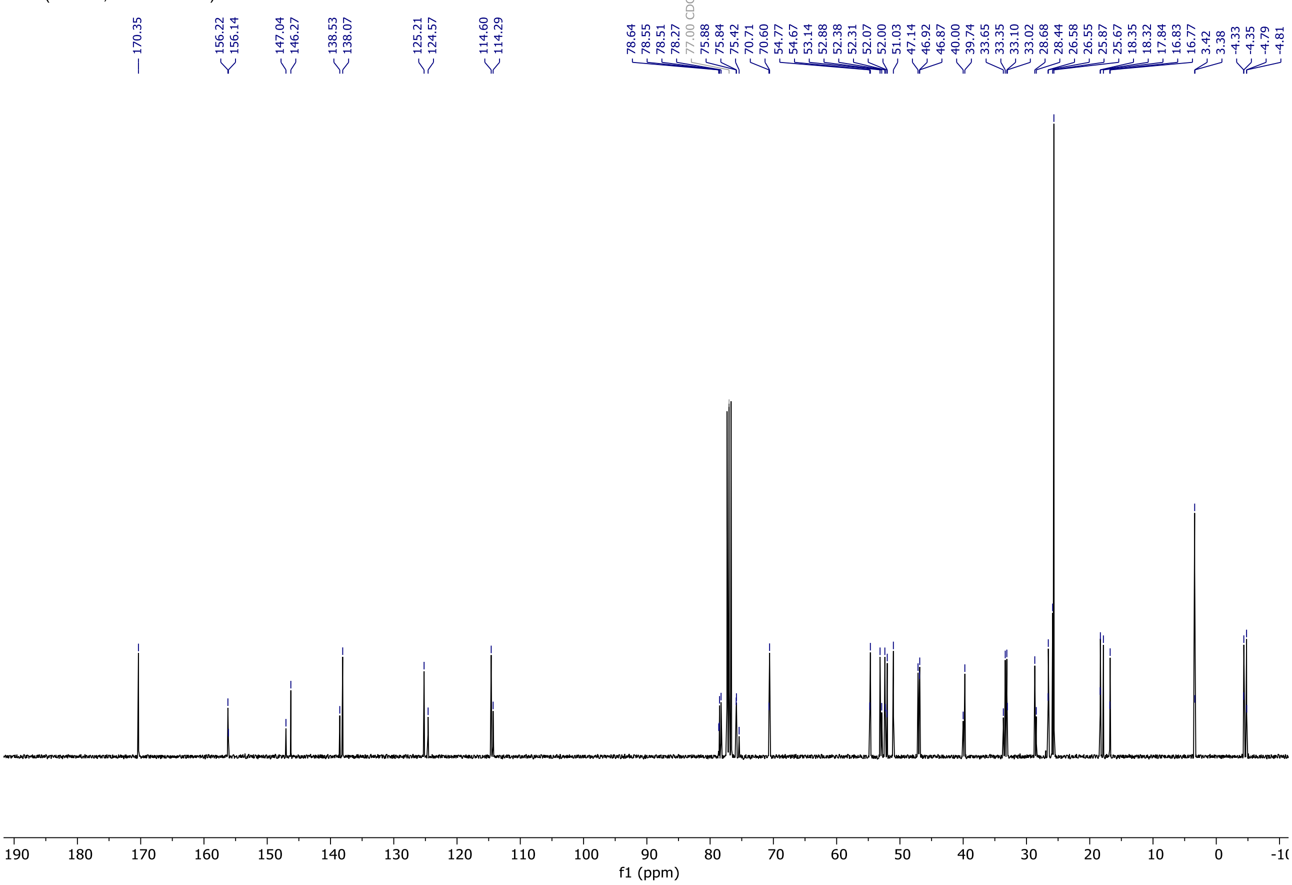

1H (CDCl3, 400.12 MHz)

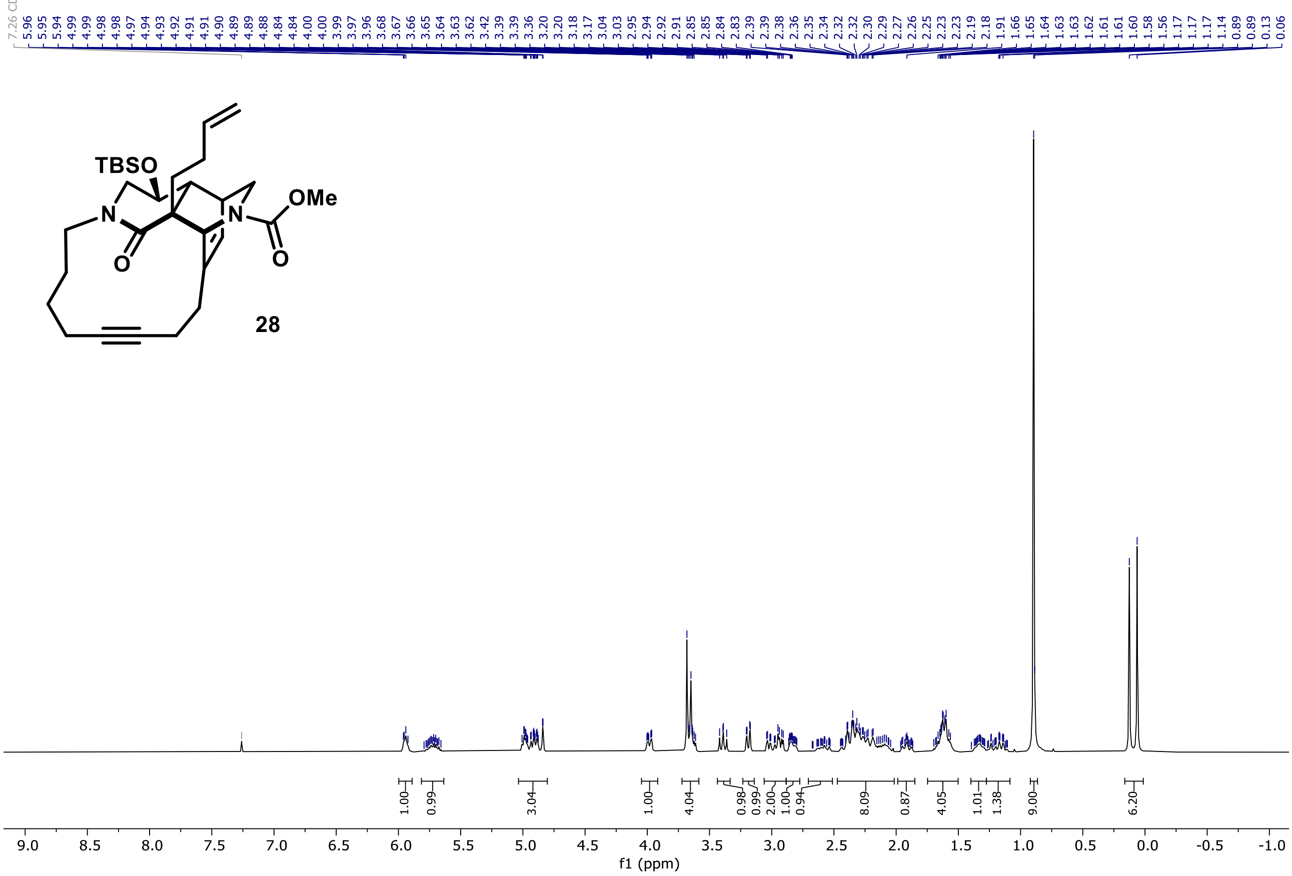

13C (CDCl3, 100.62 MHz)

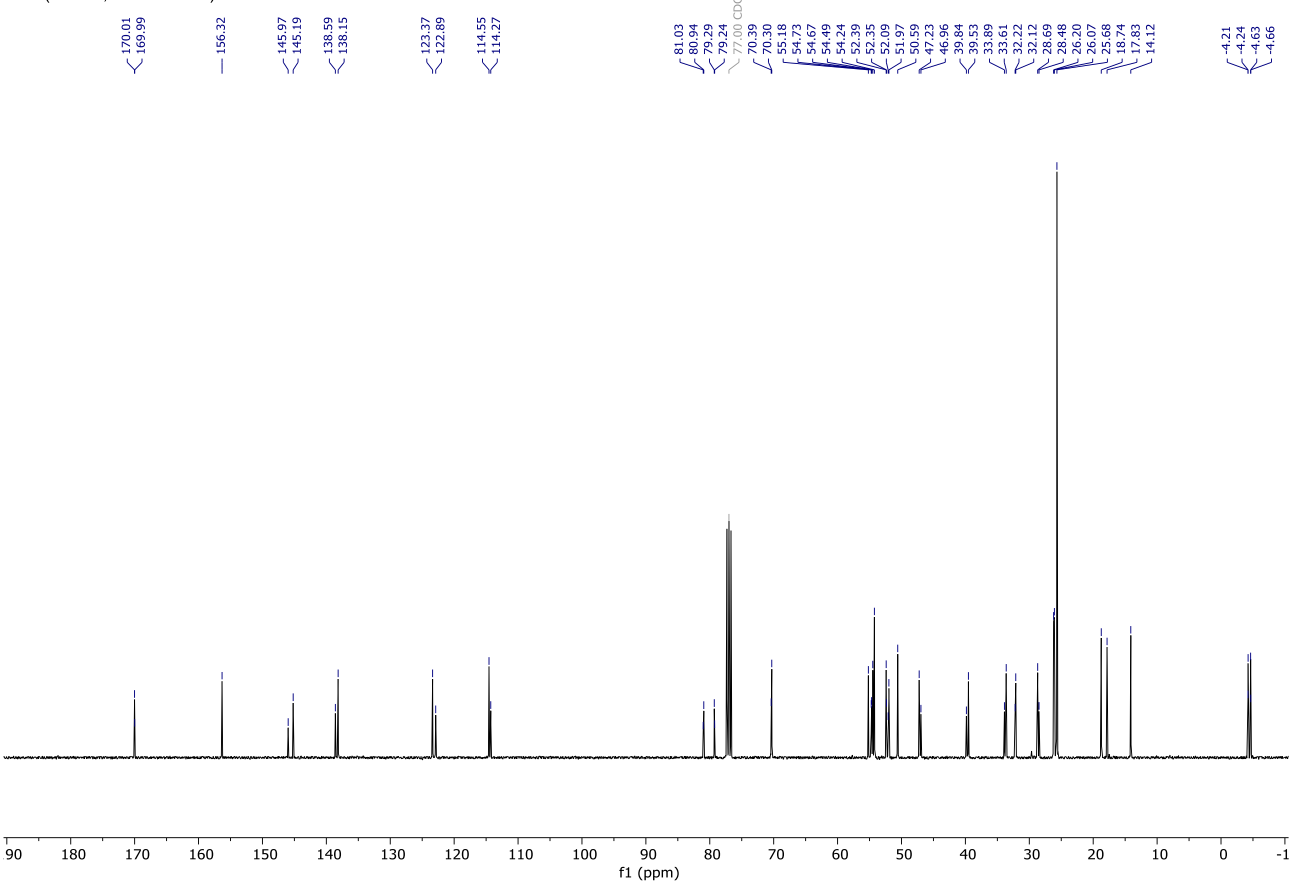

1H (CDCl3, 400.12 MHz)

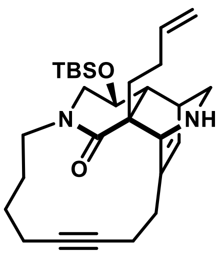

32

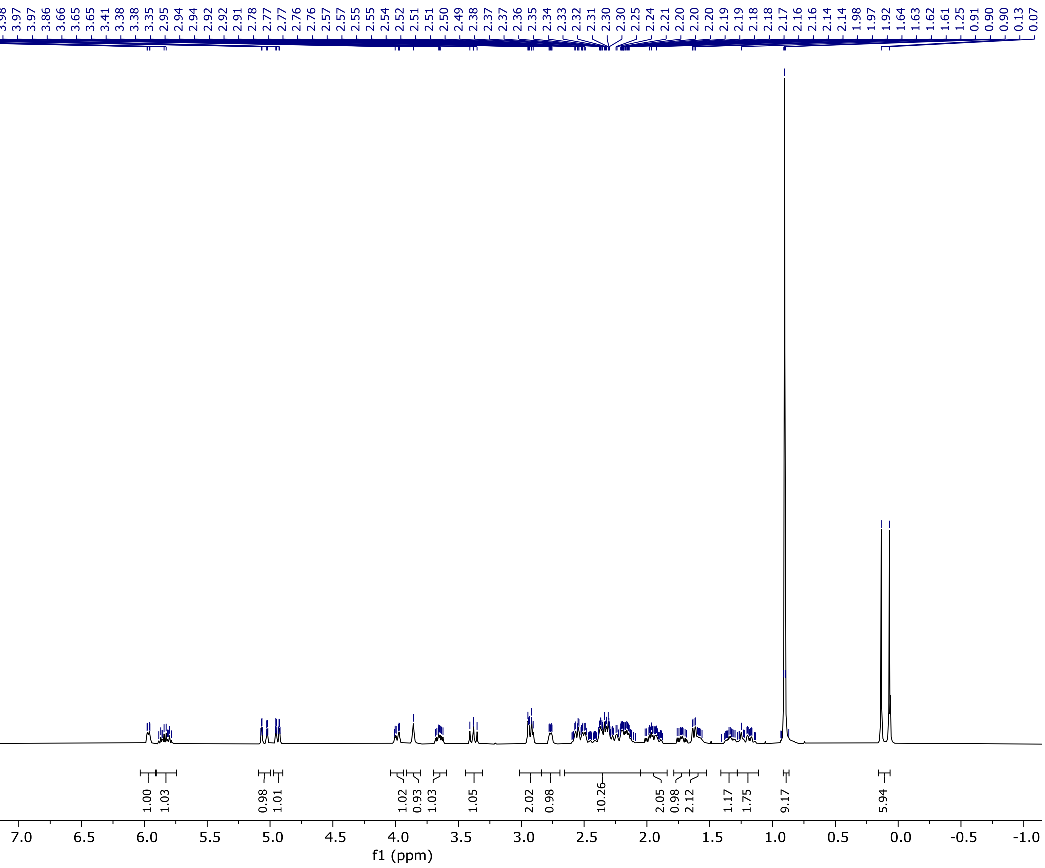

13C (CDCl3, 100.62 MHz)

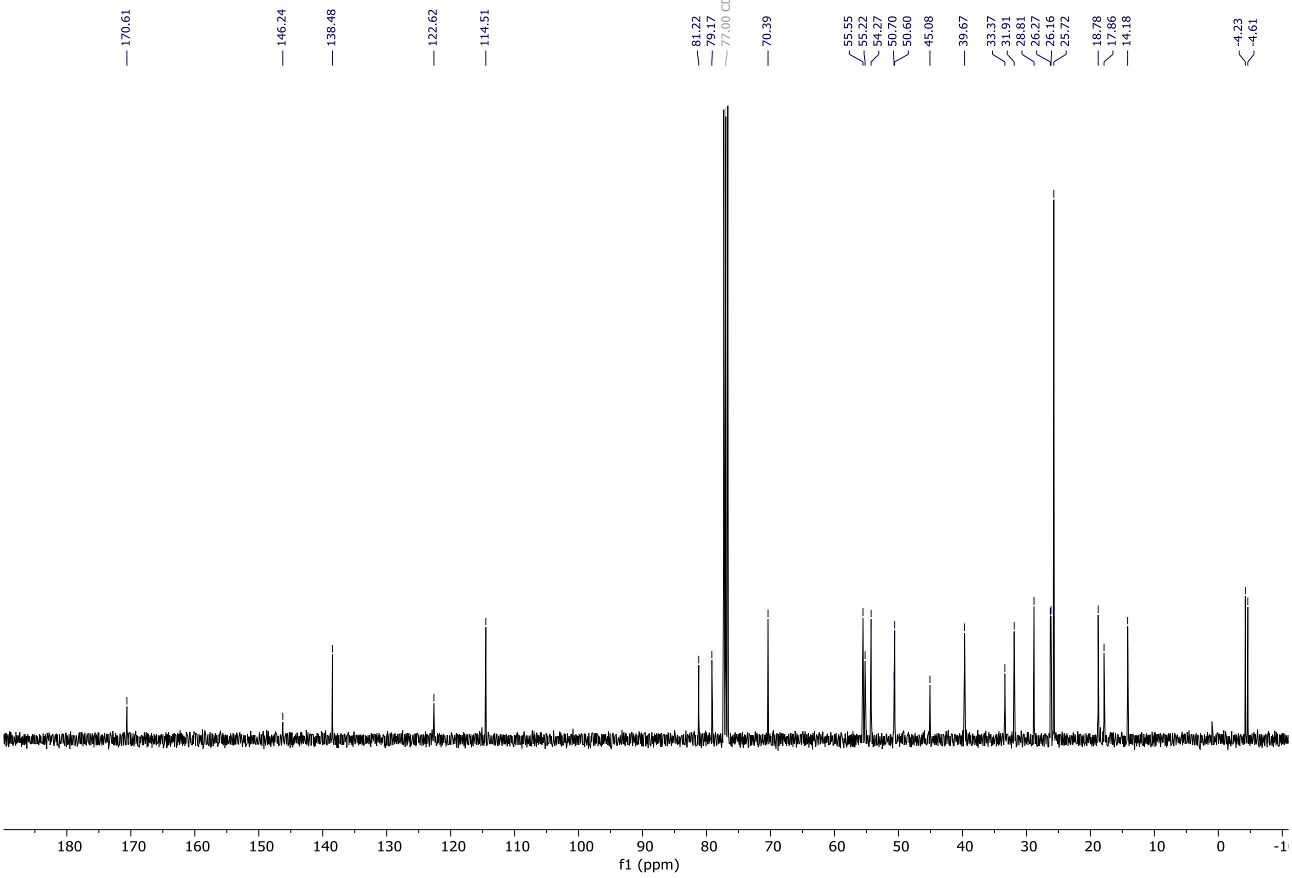

1H (CDCl3, 400.12 MHz)

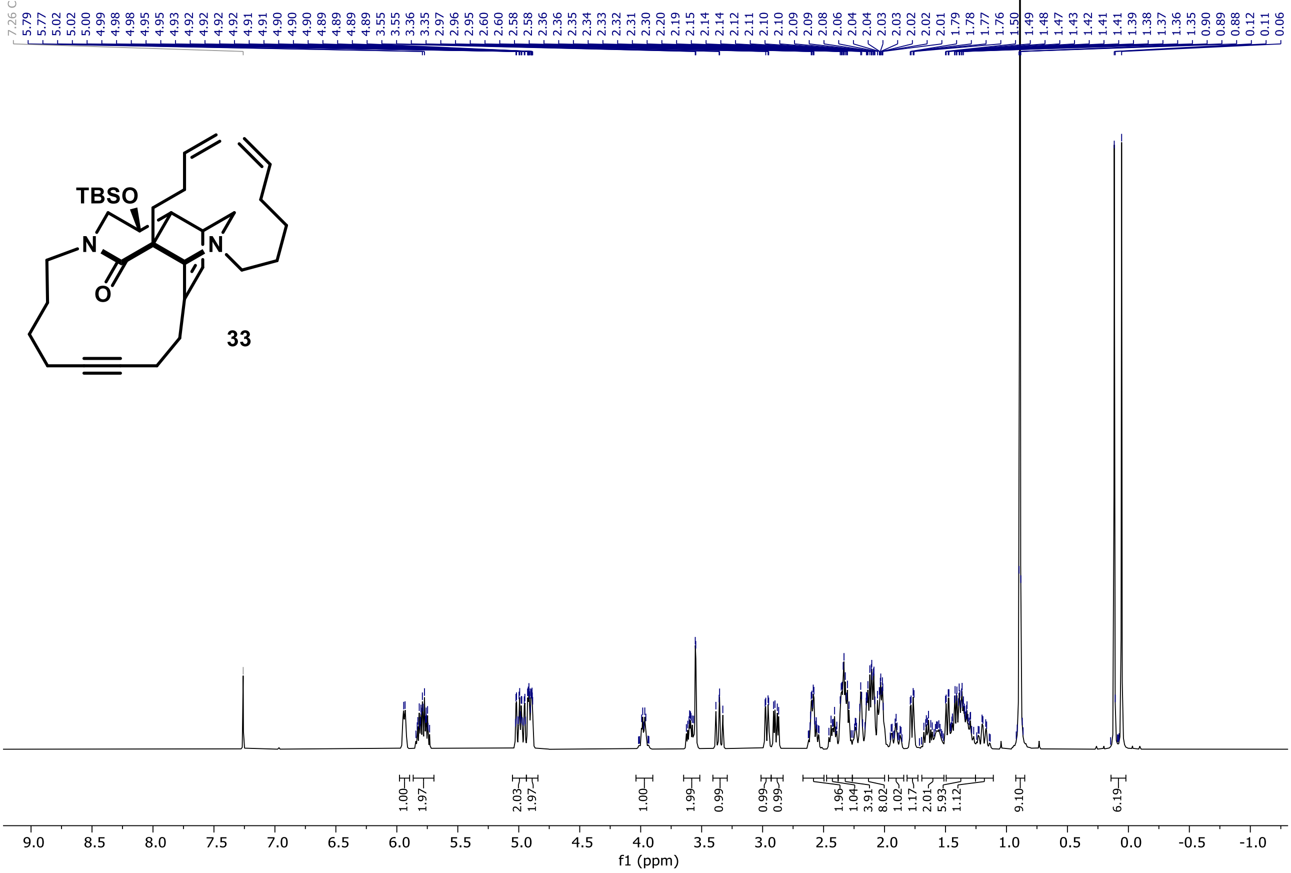

33

13C (CDCl3, 100.62 MHz)

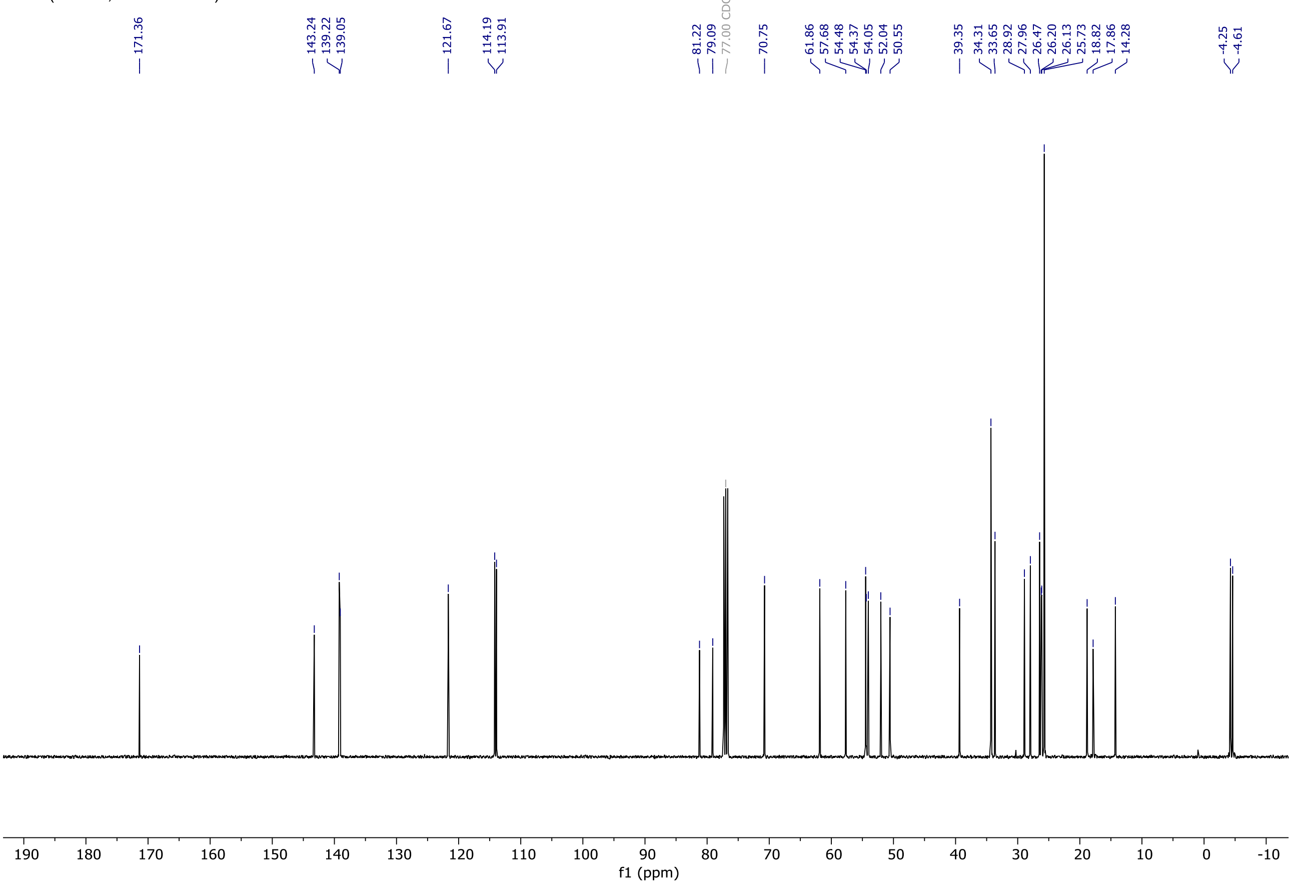

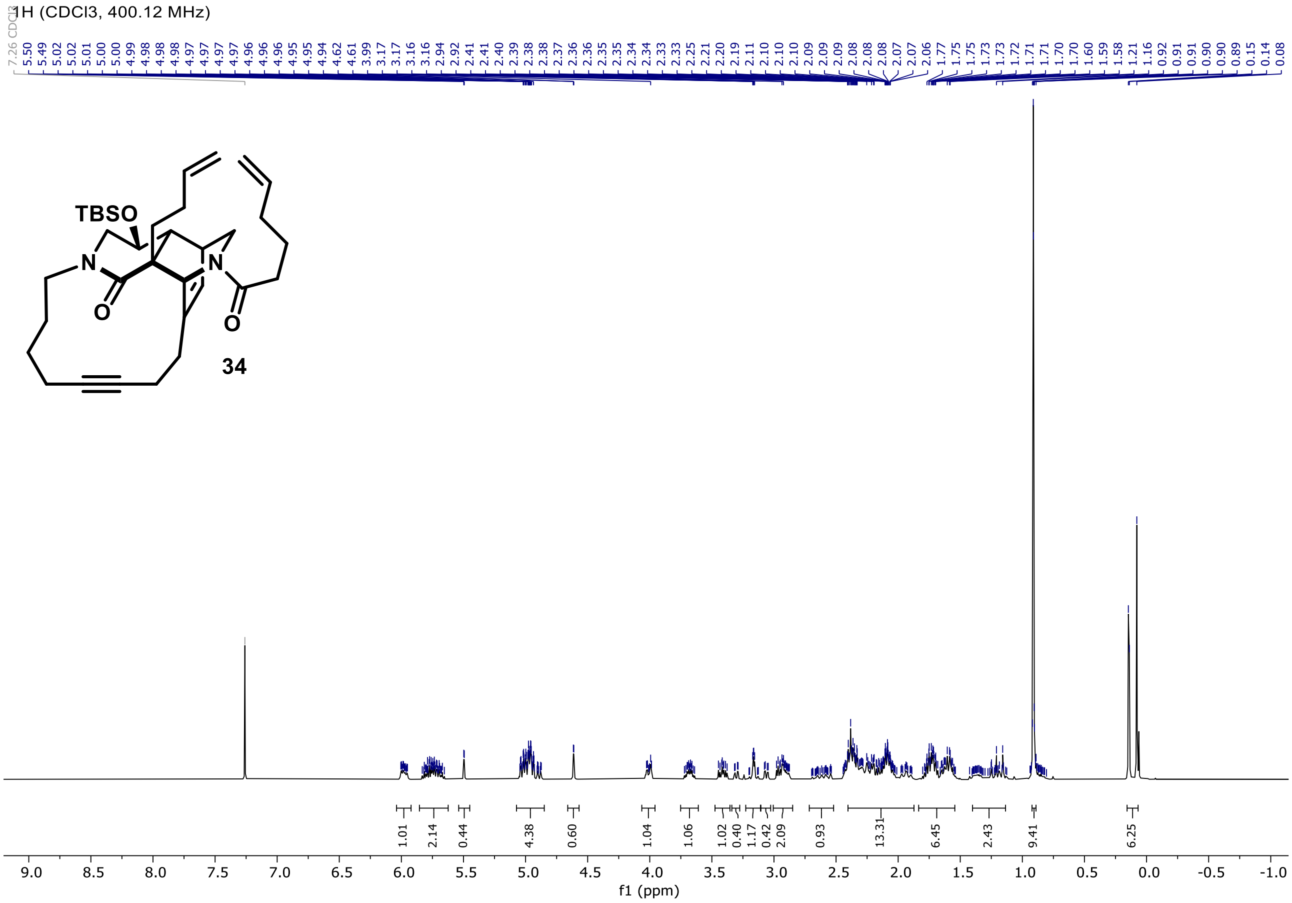
1H (CDCl3, 400.12 MHz)
7.26 CDCl3
5.50 5.49 5.02 5.01 5.00 5.00 4.99 4.98 4.98 4.97 4.97 4.97 4.96 4.96 4.95 4.95 4.94 4.62 4.61 3.99 3.17 3.17 3.16 2.94 2.92 2.41 2.41 2.40 2.39 2.38 2.38 2.37 2.36 2.36 2.35 2.35 2.34 2.34 2.33 2.33 2.25 2.21 2.20 2.19 2.11 2.10 2.10 2.09 2.09 2.09 2.08 2.08 2.08 2.07 2.07 2.06 1.77 1.75 1.75 1.73 1.73 1.72 1.71 1.71 1.70 1.70 1.60 1.59 1.58 1.21 1.16 0.92 0.91 0.91 0.90 0.90 0.89 0.15 0.14 0.08
TBSO
N
N
O
O
34
1.01 2.14 0.44 4.38 0.60 1.04 1.06 1.02 0.40 1.17 0.42 2.09 0.93 13.31 6.45 2.43 9.41 6.25
9.0 8.5 8.0 7.5 7.0 6.5 6.0 5.5 5.0 4.5 4.0 3.5 3.0 2.5 2.0 1.5 1.0 0.5 0.0 -0.5 -1.0
f1 (ppm)

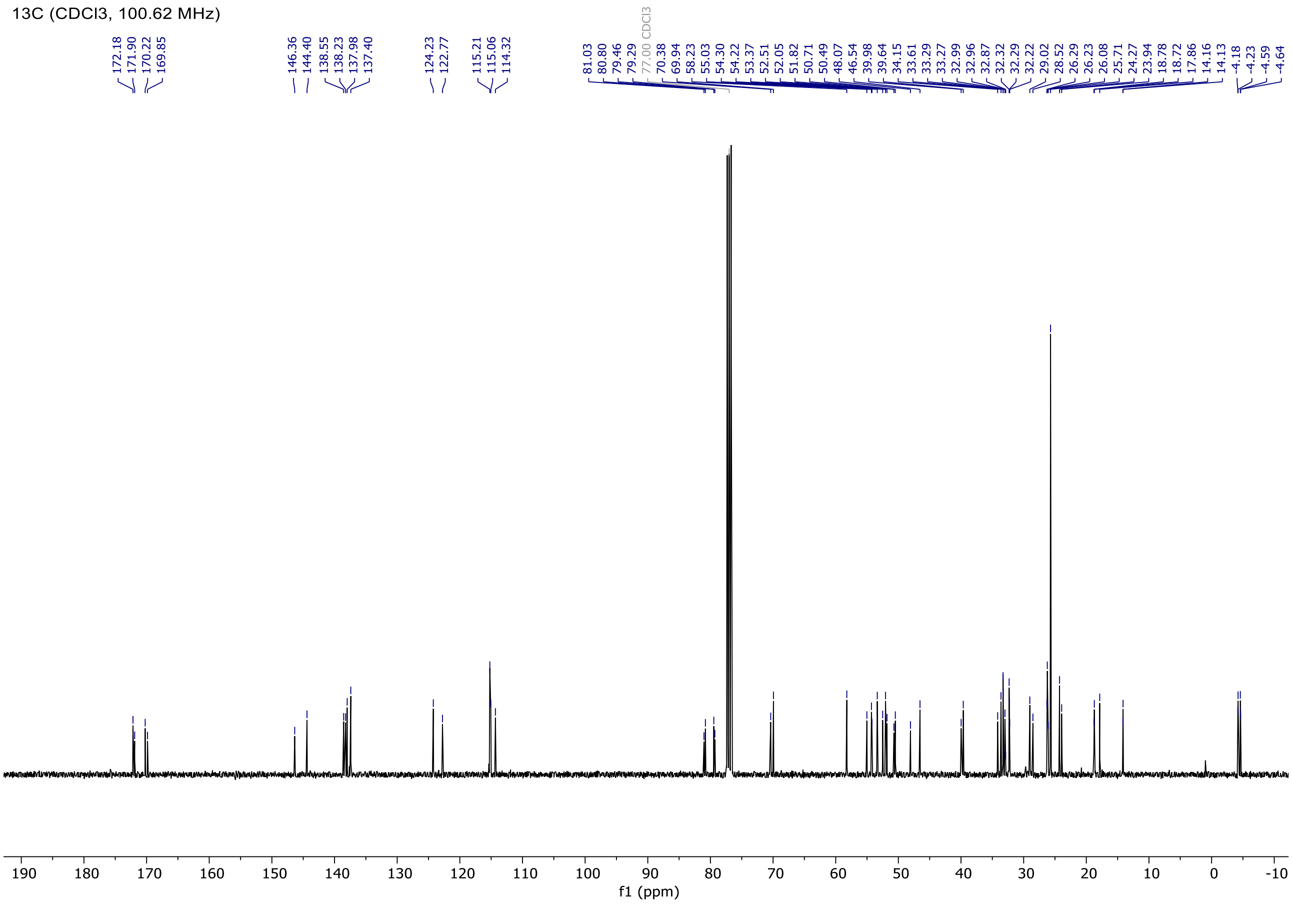
13C (CDCl3, 100.62 MHz)
172.18 171.90 170.22 169.85 146.36 144.40 138.55 138.23 137.98 137.40 124.23 122.77 115.21 115.06 114.32 81.03 80.80 79.46 79.29
77.00 CDCl3
70.38 69.94 58.23 55.03 54.30 54.22 53.37 52.51 52.05 51.82 50.71 50.49 48.07 46.54 39.98 39.64 34.15 33.61 33.29 33.27 32.99 32.96 32.87 32.32 32.29 32.22 29.02 28.52 26.29 26.23 26.08 25.71 24.27 23.94 18.78 18.72 17.86 14.16 14.13 -4.18 -4.23 -4.59 -4.64
190 180 170 160 150 140 130 120 110 100 90 80 70 60 50 40 30 20 10 0 -10
f1 (ppm)

^{1}H NMR Comparison *E*-/*Z*-mixture (top) and pure *Z*-Isomer (bottom)

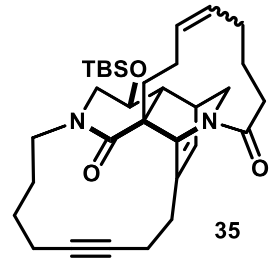

35

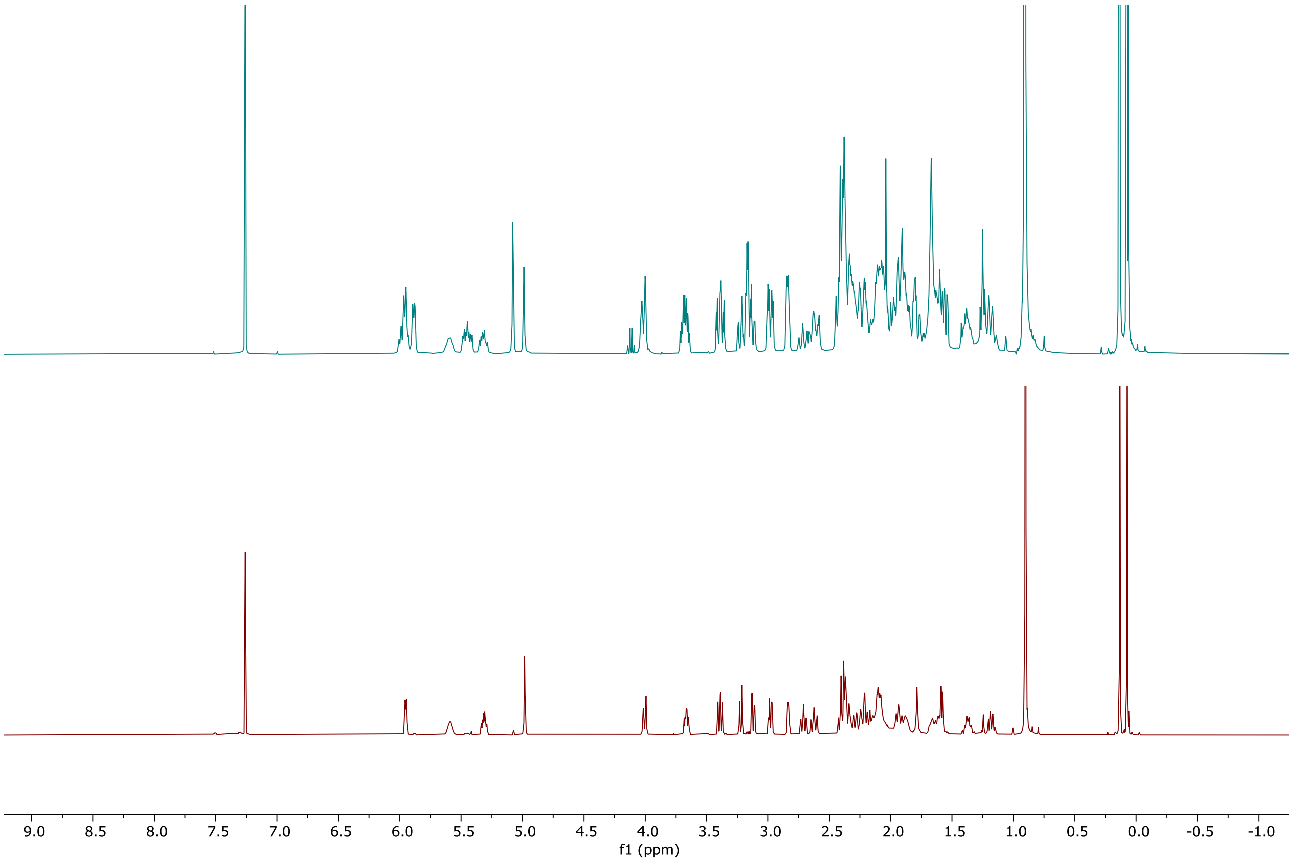

1H (CDCl3, 600.22 MHz)

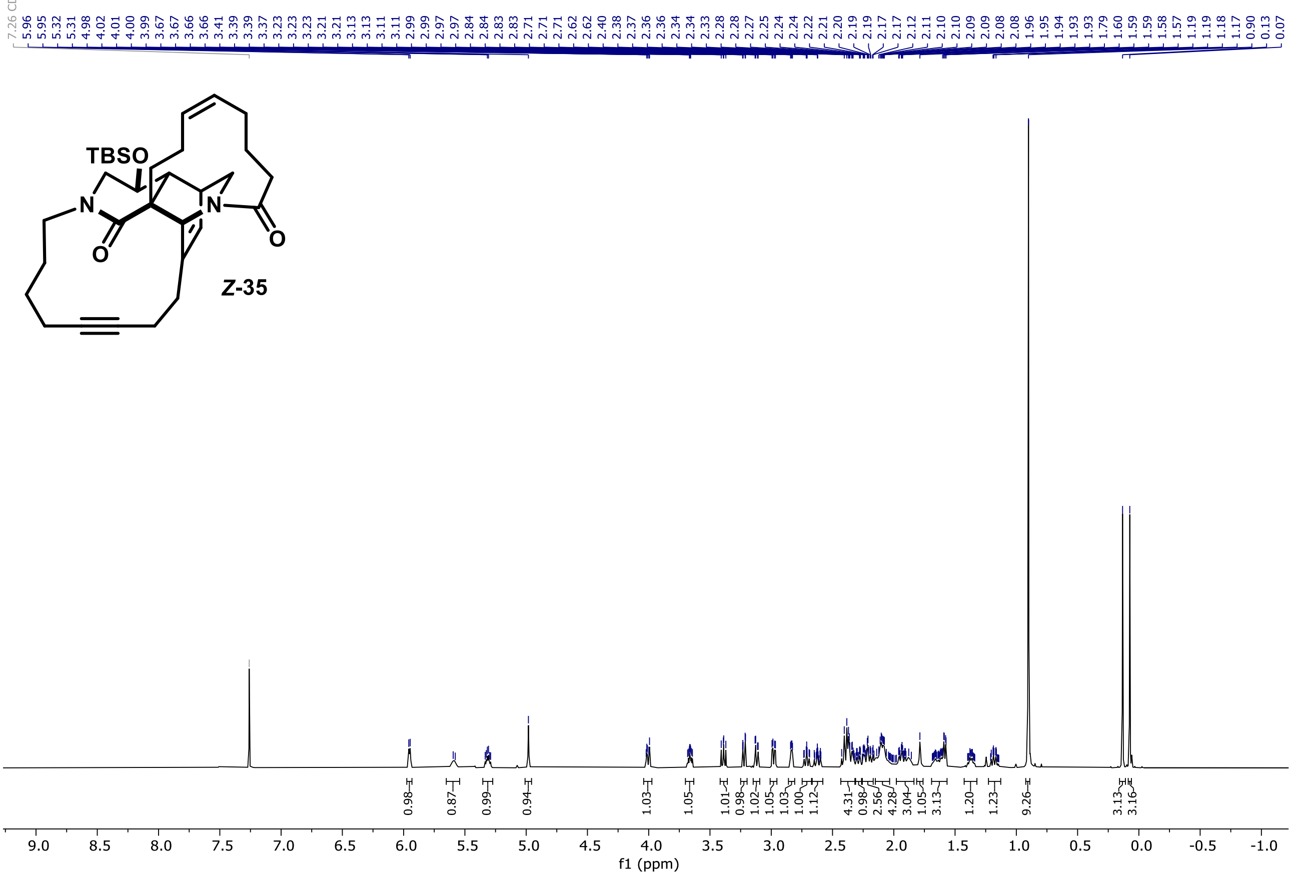

13C (CDCl3, 150.94 MHz)

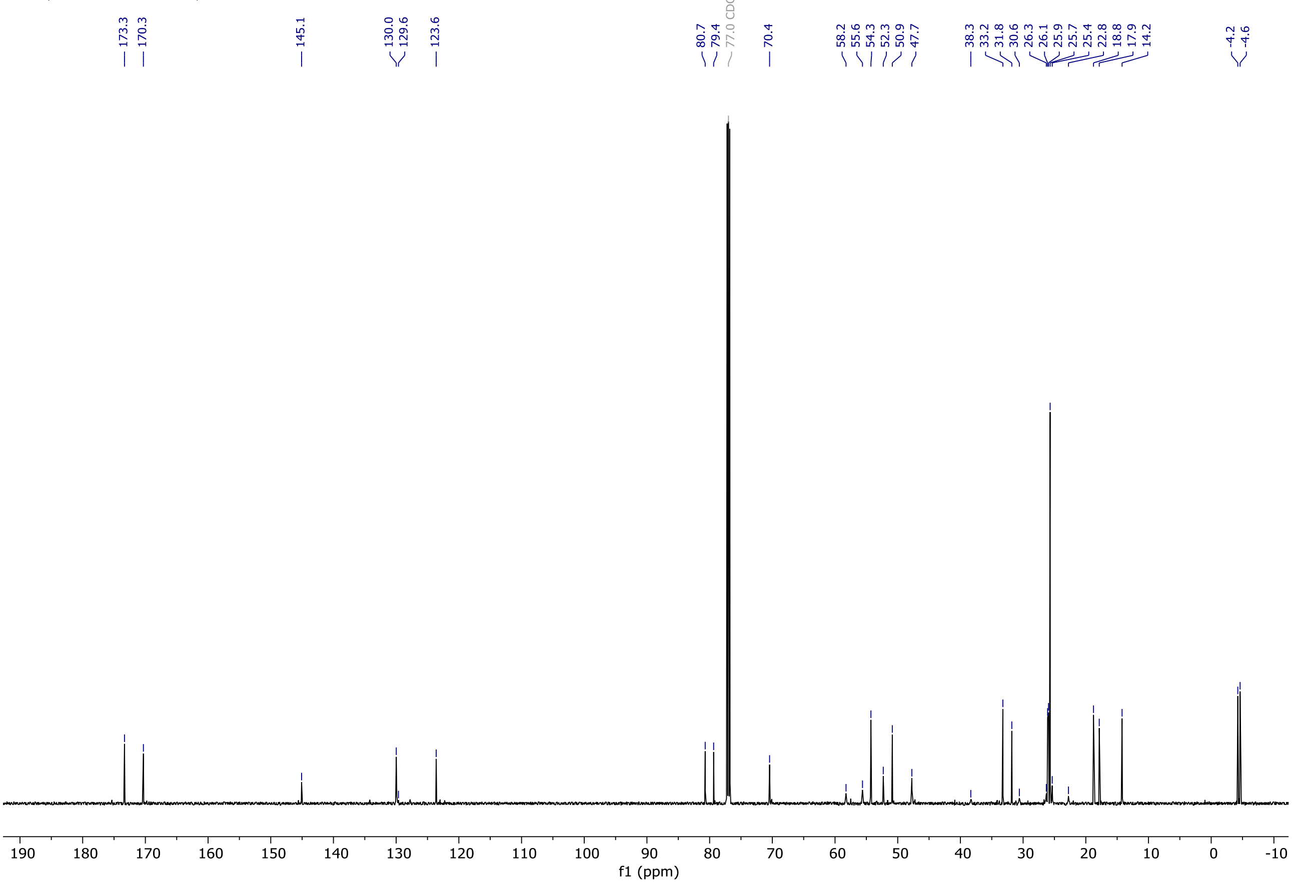

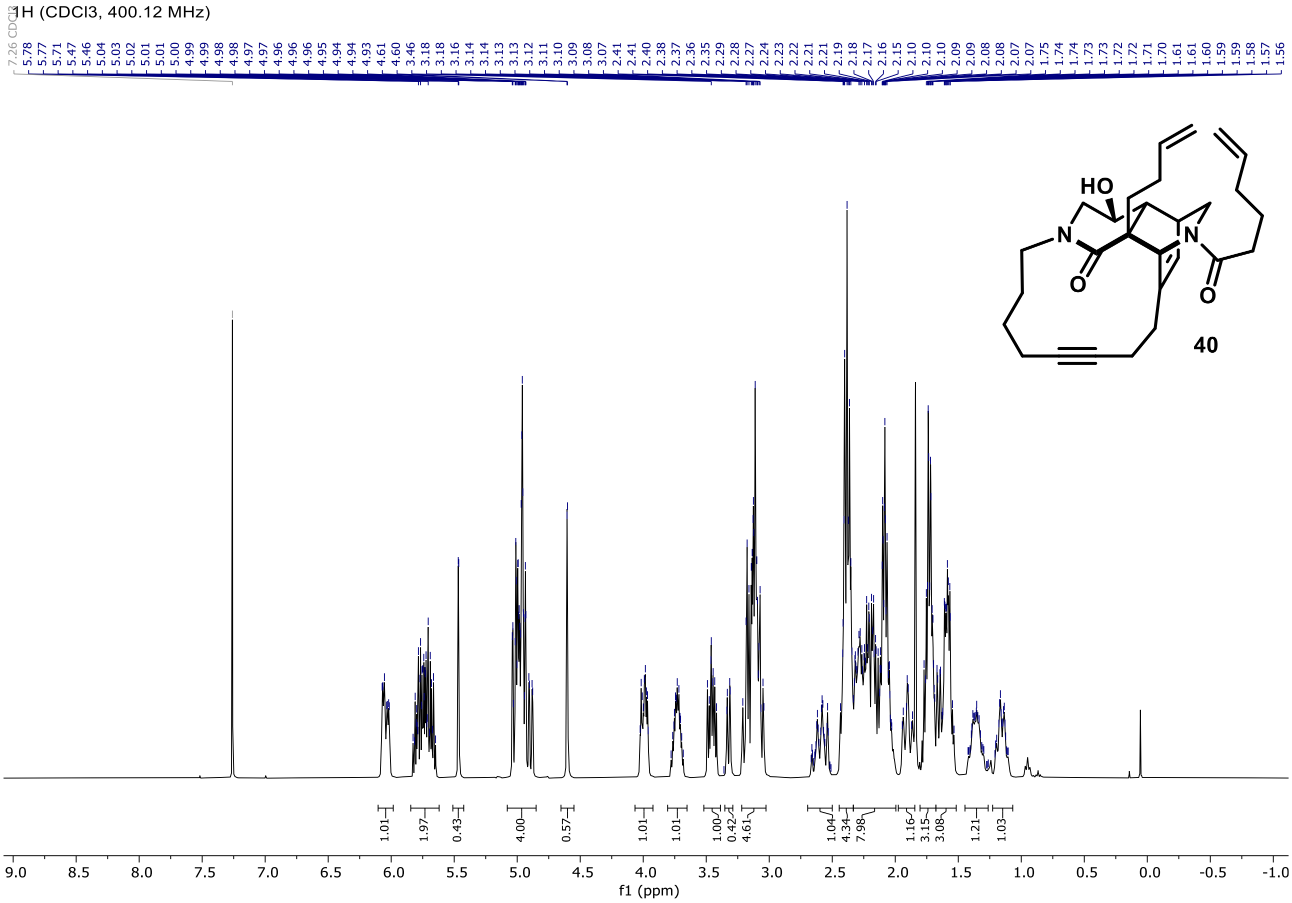
1H (CDCl3, 400.12 MHz)
7.26 CDCl3
5.78 5.77 5.71 5.47 5.46 5.04 5.03 5.02 5.01 5.01 5.00 4.99 4.99 4.98 4.98 4.97 4.97 4.96 4.96 4.96 4.95 4.94 4.94 4.93 4.61 4.60 3.46 3.18 3.18 3.16 3.14 3.14 3.13 3.13 3.12 3.11 3.10 3.09 3.08 3.07 2.41 2.41 2.40 2.38 2.37 2.36 2.35 2.29 2.28 2.27 2.24 2.23 2.22 2.21 2.21 2.19 2.18 2.17 2.16 2.15 2.10 2.10 2.10 2.09 2.09 2.08 2.08 2.07 2.07 1.75 1.74 1.74 1.73 1.73 1.72 1.72 1.71 1.70 1.61 1.61 1.60 1.59 1.59 1.58 1.57 1.56
HO
N
N
O
O
40
1.01 1.97 0.43 4.00 0.57 1.01 1.01 1.00 0.42 4.61 1.04 4.34 7.98 1.16 3.15 3.08 1.21 1.03
9.0 8.5 8.0 7.5 7.0 6.5 6.0 5.5 5.0 4.5 4.0 3.5 3.0 2.5 2.0 1.5 1.0 0.5 0.0 -0.5 -1.0
f1 (ppm)

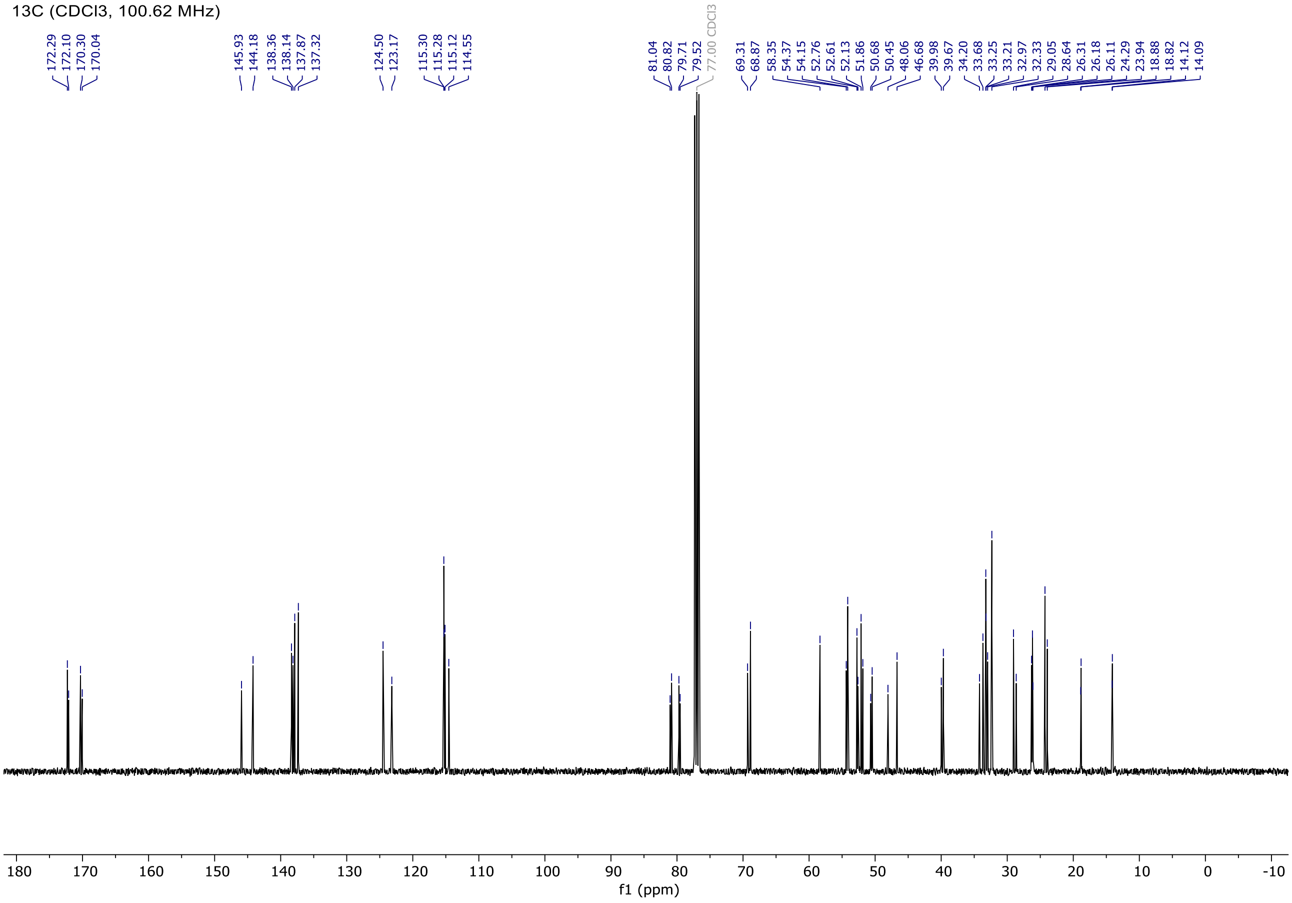
13C (CDCl3, 100.62 MHz)
172.29 172.10 170.30 170.04 145.93 144.18 138.36 138.14 137.87 137.32 124.50 123.17 115.30 115.28 115.12 114.55 81.04 80.82 79.71 79.52 77.00 CDCl3 69.31 68.87 58.35 54.37 54.15 52.76 52.61 52.13 51.86 50.68 50.45 48.06 46.68 39.98 39.67 34.20 33.68 33.25 33.21 32.97 32.33 29.05 28.64 26.31 26.18 26.11 24.29 23.94 18.88 18.82 14.12 14.09
180 170 160 150 140 130 120 110 100 90 80 70 60 50 40 30 20 10 0 -10
f1 (ppm)

1H (CDCl3, 400.12 MHz)

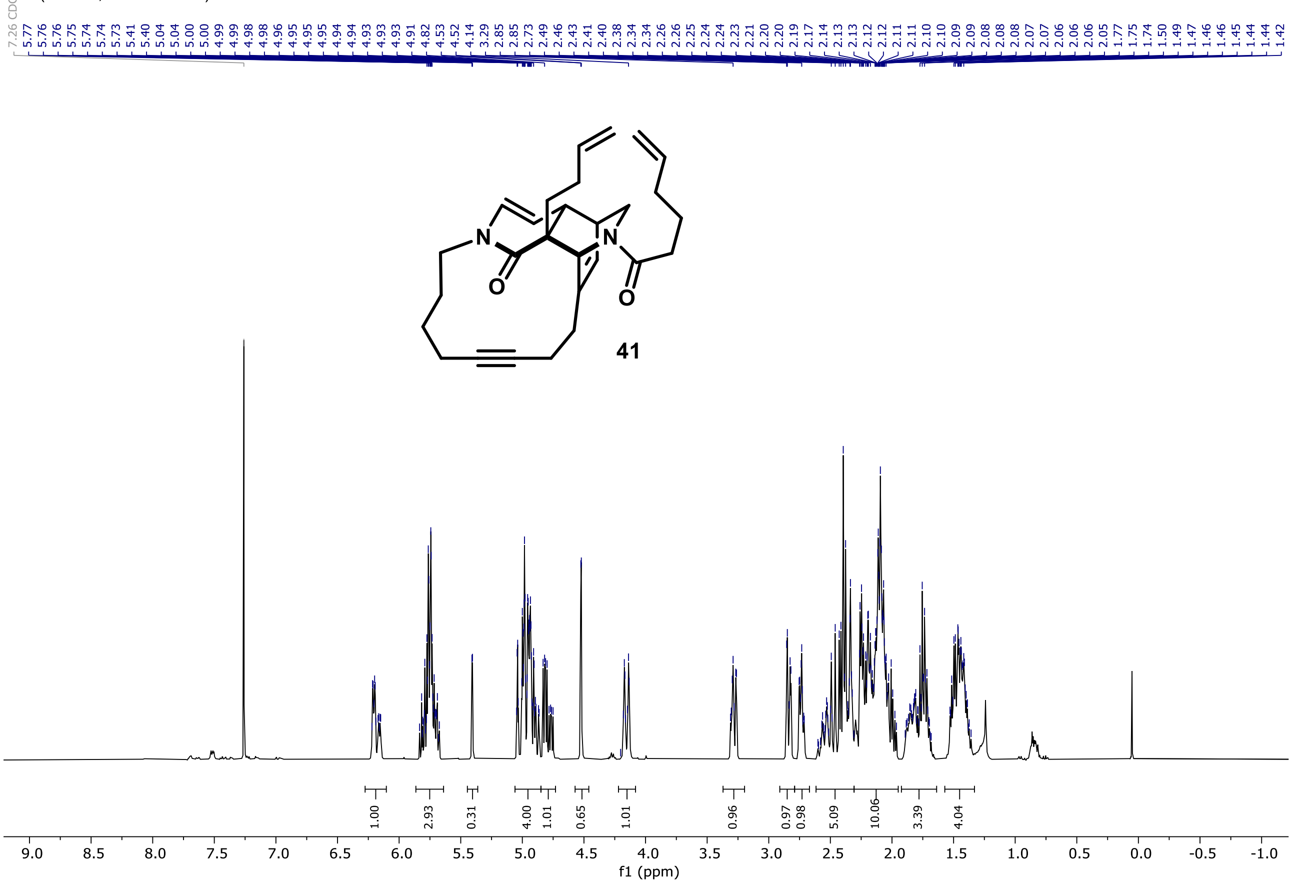

41

13C (CDCl3, 100.62 MHz)

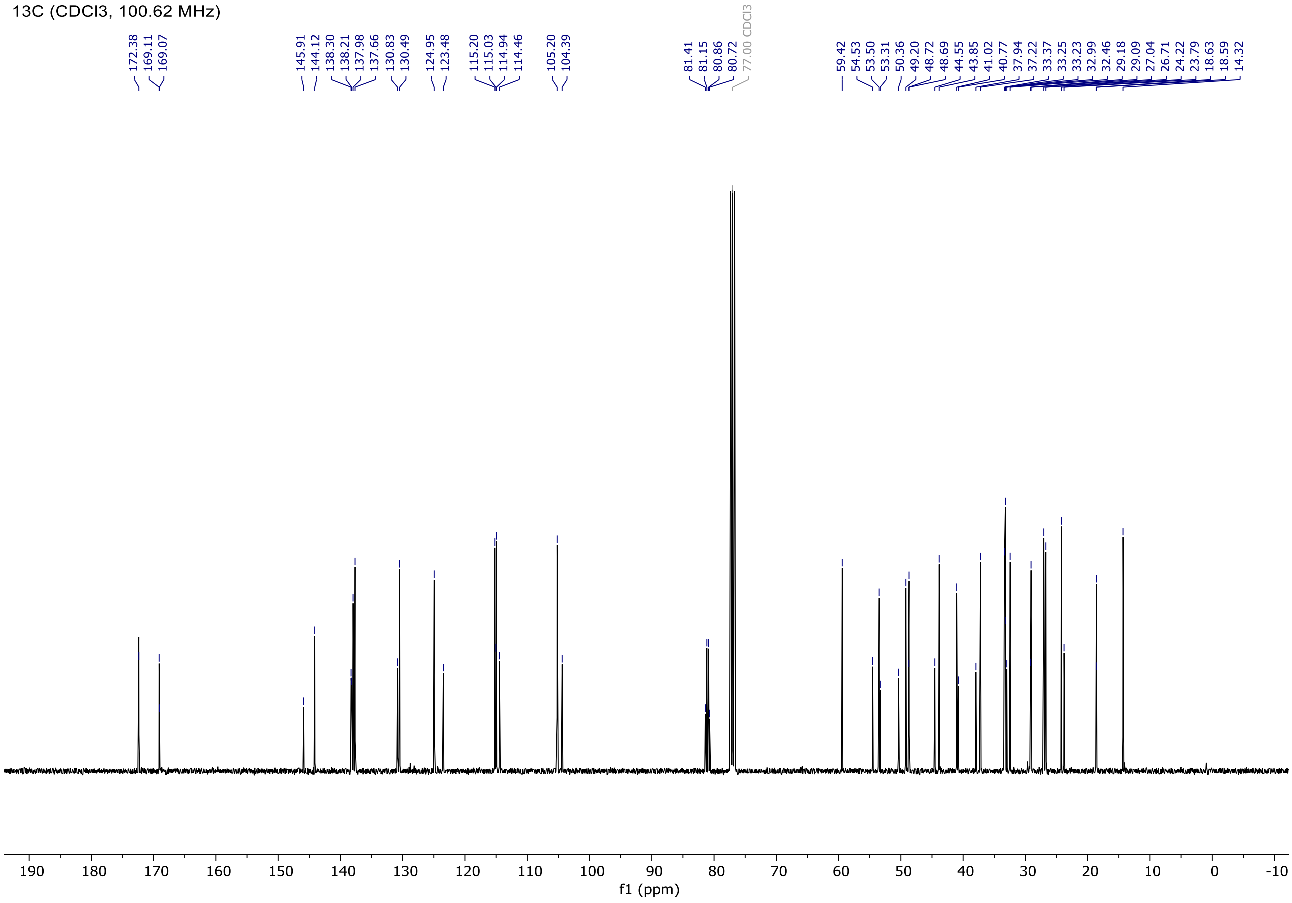

1H (CDCl3, 400.12 MHz)

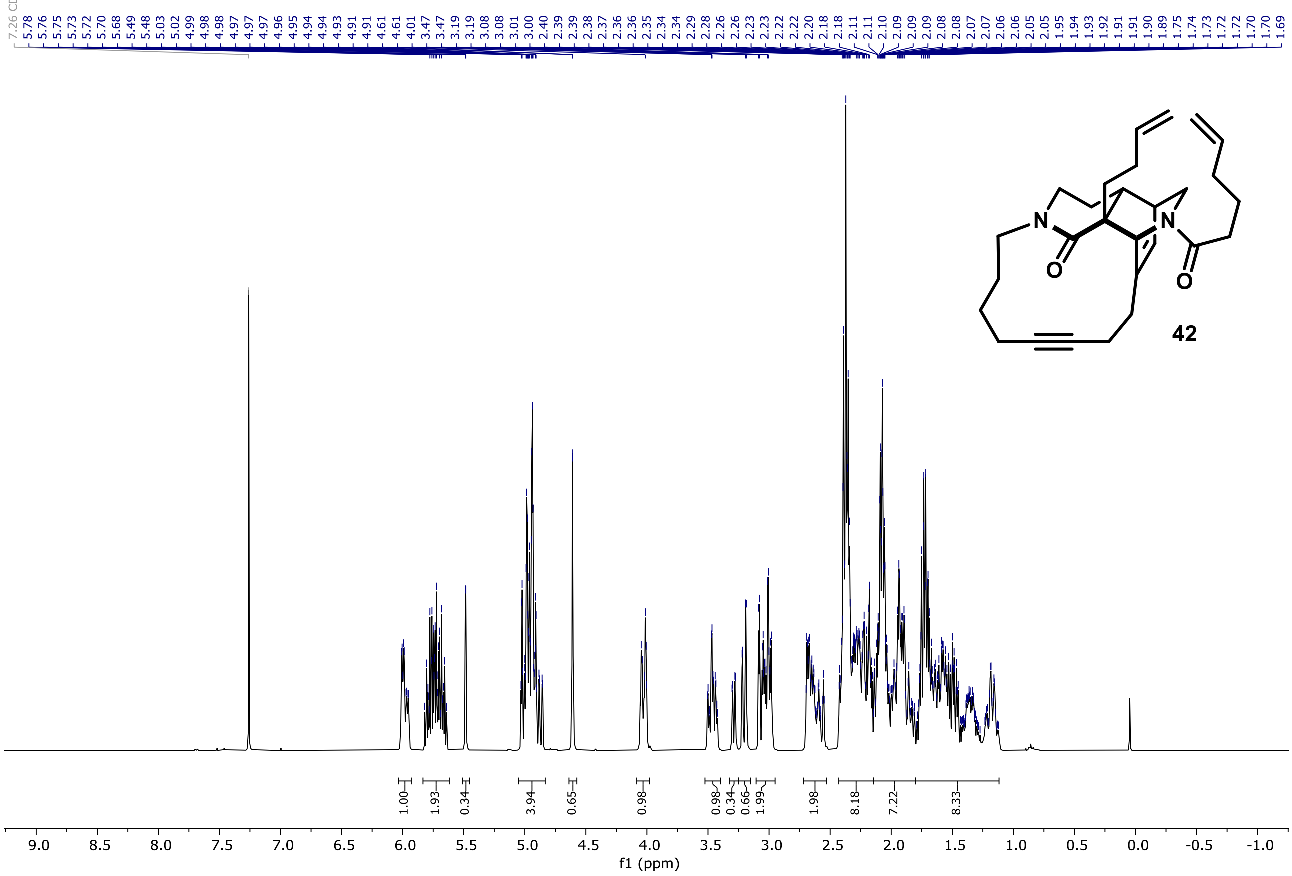

13C (CDCl3, 100.62 MHz)

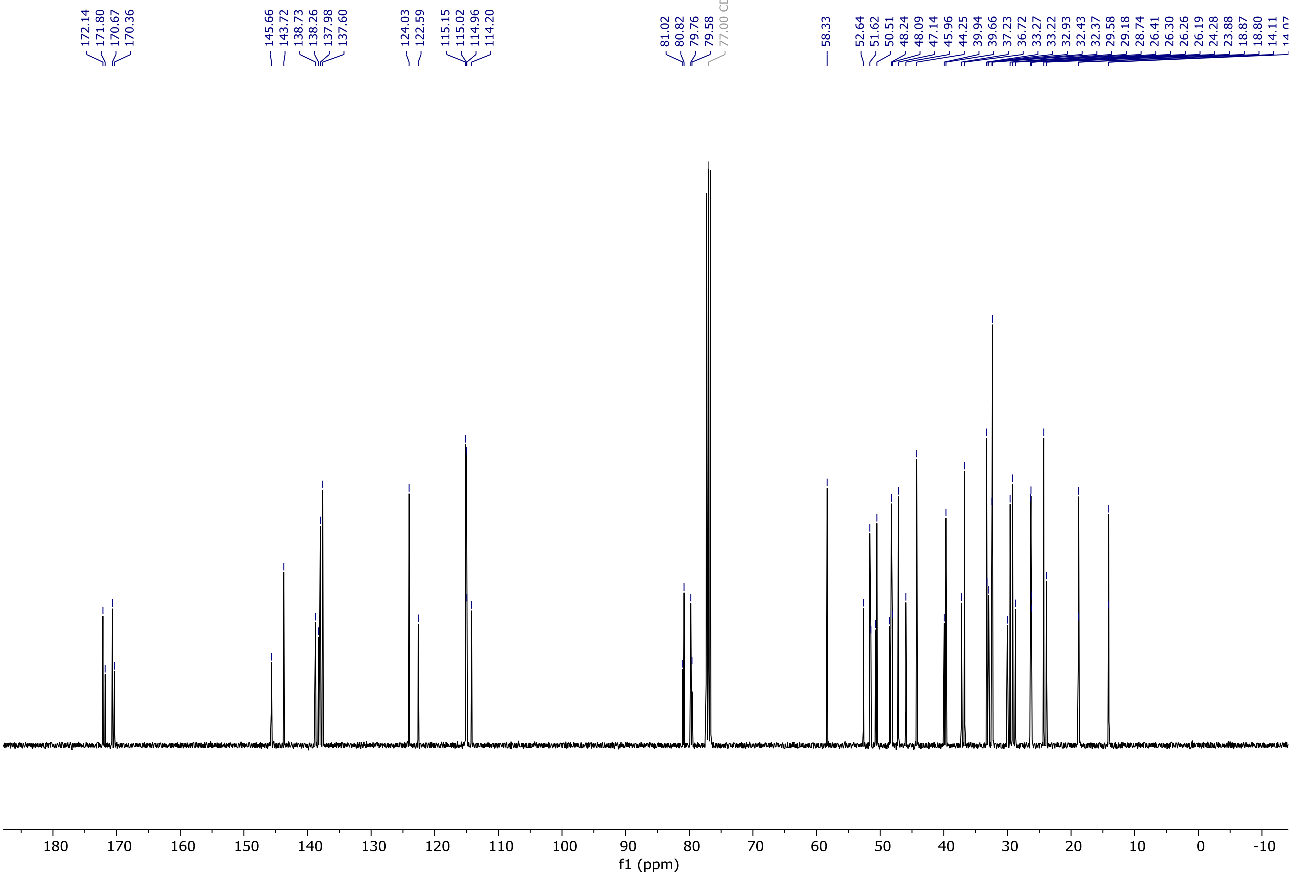

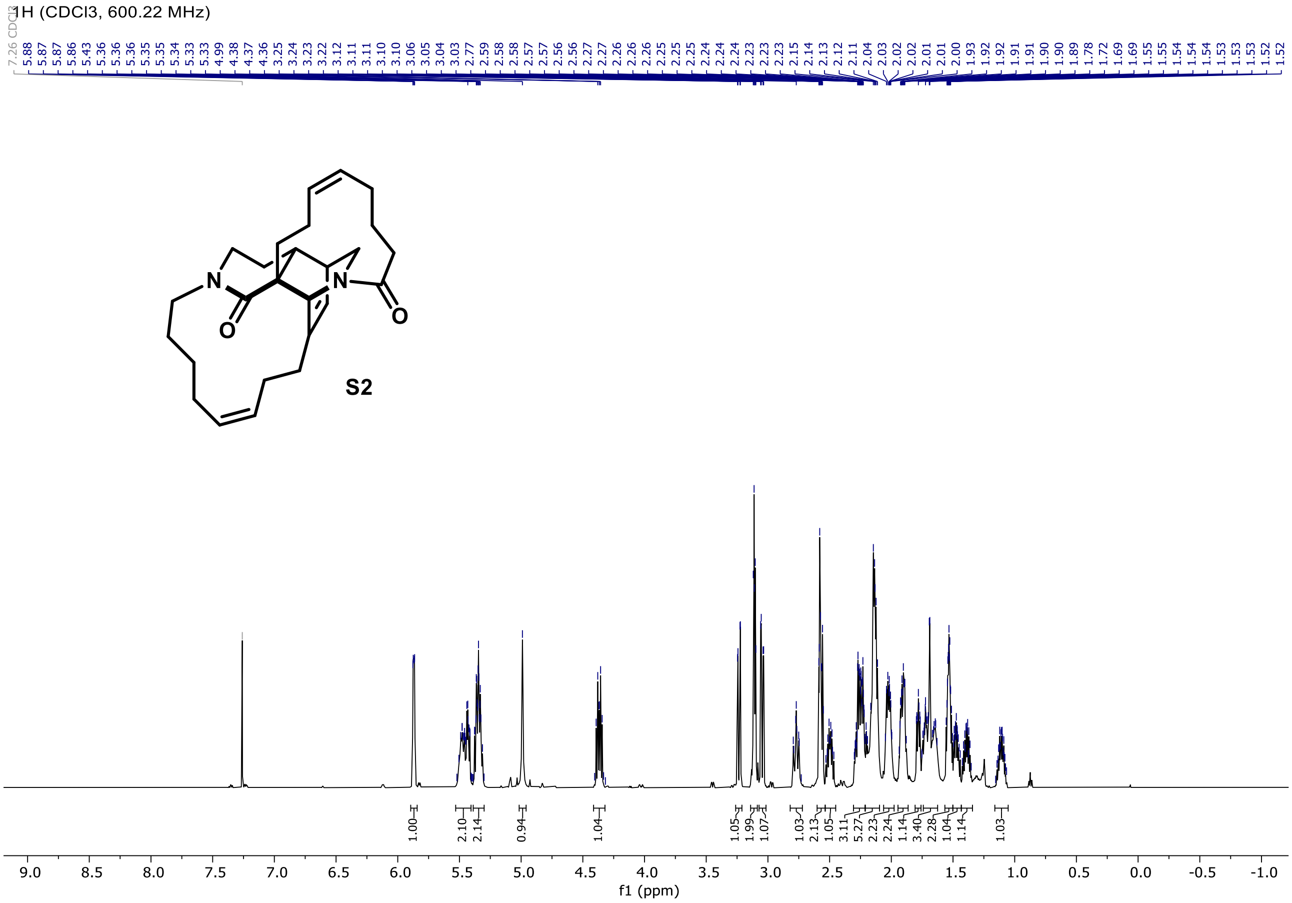
1H (CDCl3, 600.22 MHz)
7.26 CDCl3
5.88 5.87 5.87 5.86 5.43 5.36 5.36 5.36 5.35 5.35 5.34 5.33 5.33 4.99 4.38 4.37 4.36 3.25 3.24 3.23 3.22 3.12 3.11 3.11 3.10 3.10 3.06 3.05 3.04 3.03 2.77 2.59 2.58 2.58 2.57 2.57 2.56 2.56 2.27 2.26 2.26 2.25 2.25 2.25 2.24 2.24 2.24 2.23 2.23 2.23 2.15 2.14 2.13 2.12 2.11 2.04 2.03 2.02 2.02 2.01 2.01 2.00 1.93 1.92 1.92 1.91 1.91 1.90 1.89 1.78 1.72 1.69 1.69 1.55 1.55 1.54 1.54 1.53 1.53 1.53 1.52 1.52
N
N
O
O
S2
1.00 2.10 2.14 0.94 1.04 1.05 1.99 1.07 1.03 2.13 1.05 3.11 5.27 2.23 2.24 1.14 3.40 2.28 1.04 1.14 1.03
9.0 8.5 8.0 7.5 7.0 6.5 6.0 5.5 5.0 4.5 4.0 3.5 3.0 2.5 2.0 1.5 1.0 0.5 0.0 -0.5 -1.0
f1 (ppm)

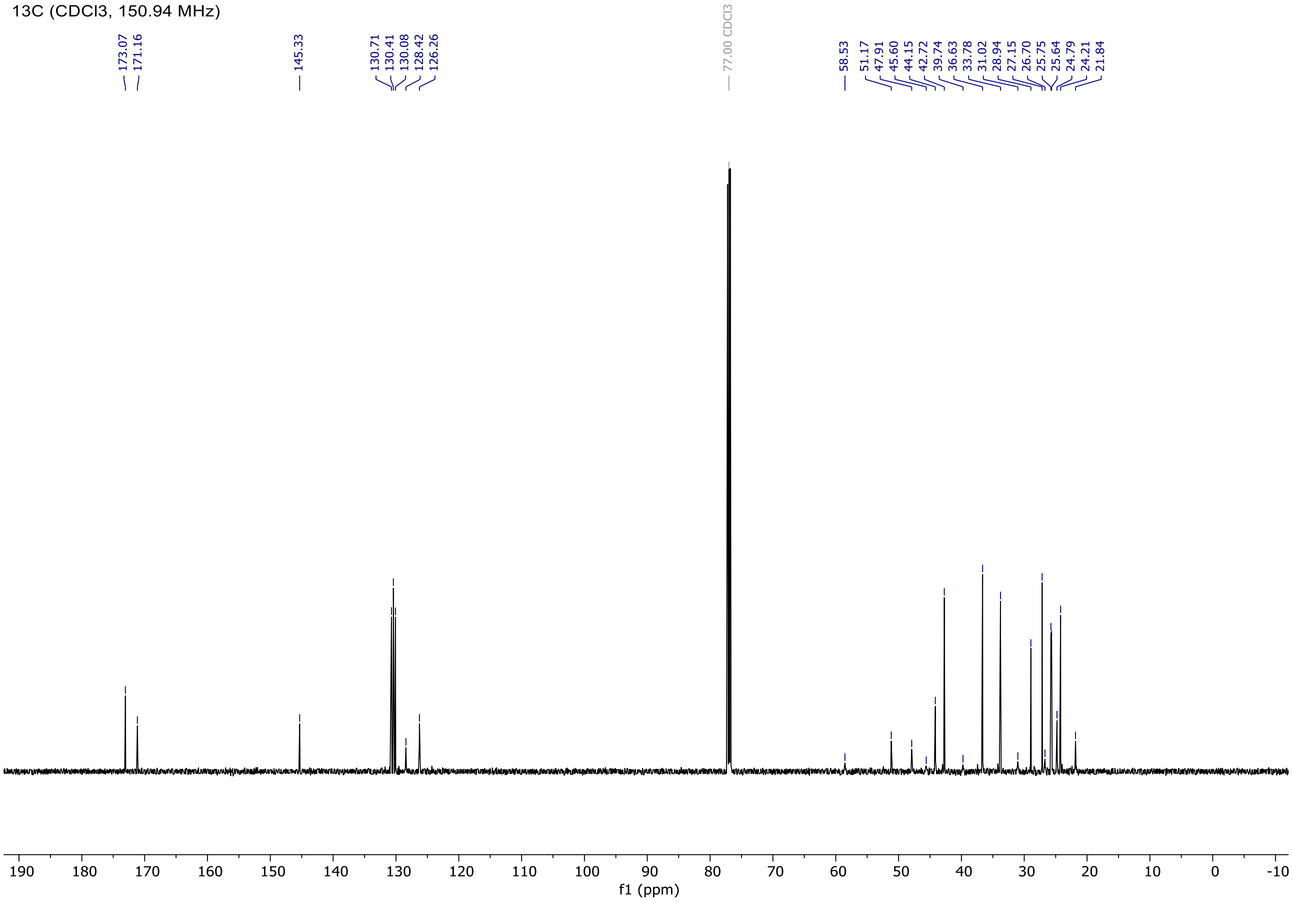
13C (CDCl3, 150.94 MHz)
173.07 171.16 145.33 130.71 130.41 130.08 128.42 126.26
77.00 CDCl3
58.53 51.17 47.91 45.60 44.15 42.72 39.74 36.63 33.78 31.02 28.94 27.15 26.70 25.75 25.64 24.79 24.21 21.84
190 180 170 160 150 140 130 120 110 100 90 80 70 60 50 40 30 20 10 0 -10
f1 (ppm)

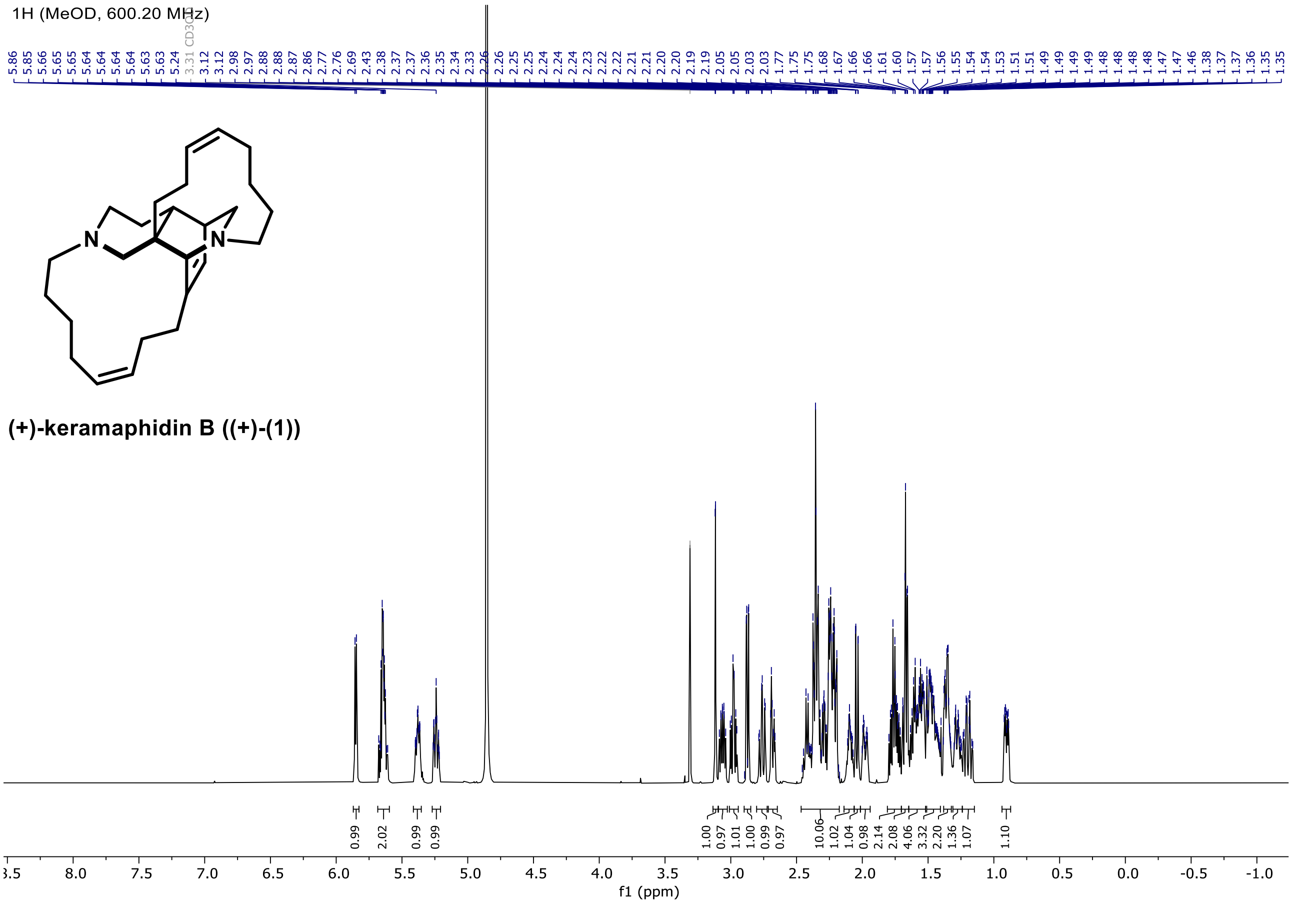

(+)-keramaphidin B ((+)-(1))

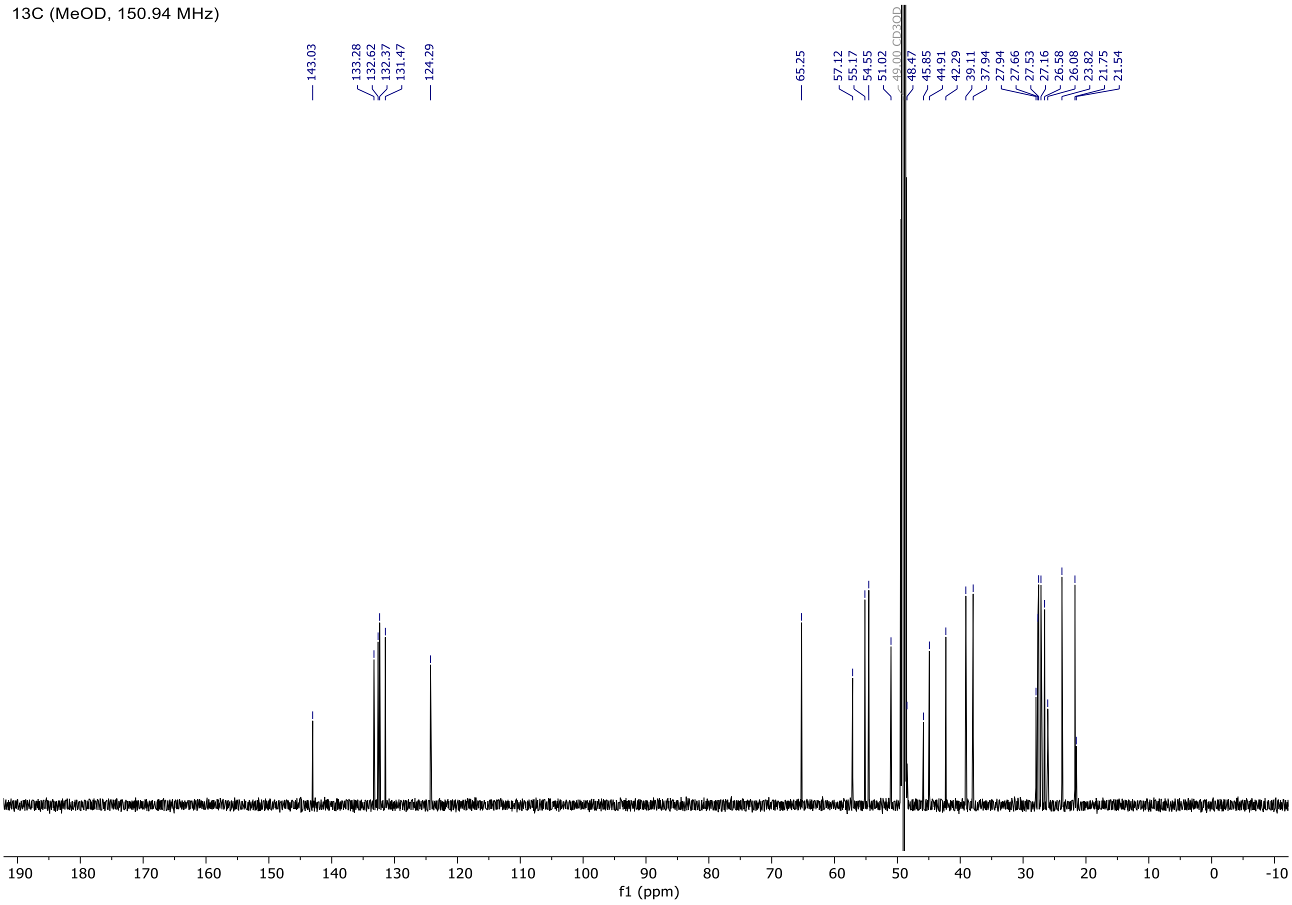

COSY Spectrum of Keramaphidin B (+)-(1) ([D4]-MeOH)

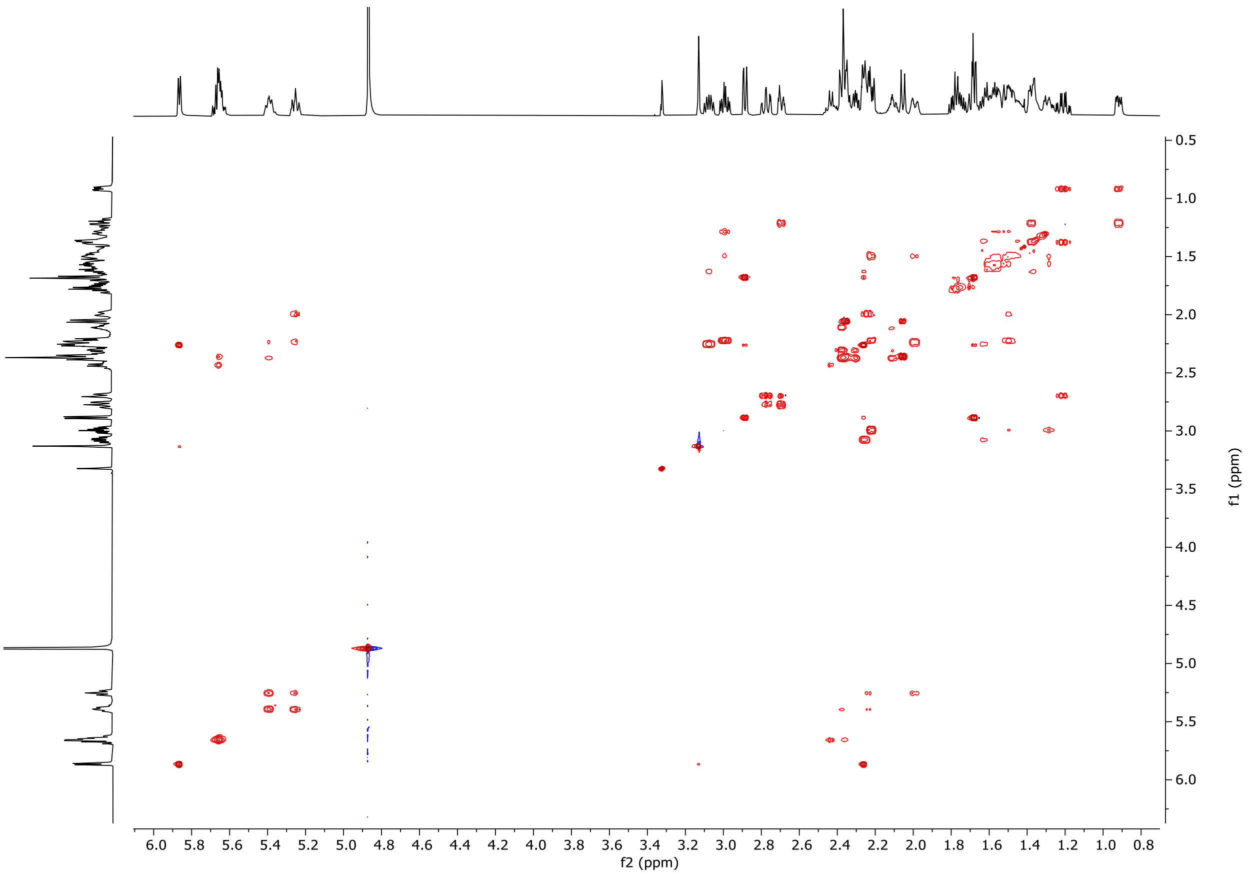

HSQC Spectrum of Keramaphidin B (+)-(1) ([D4]-MeOH)

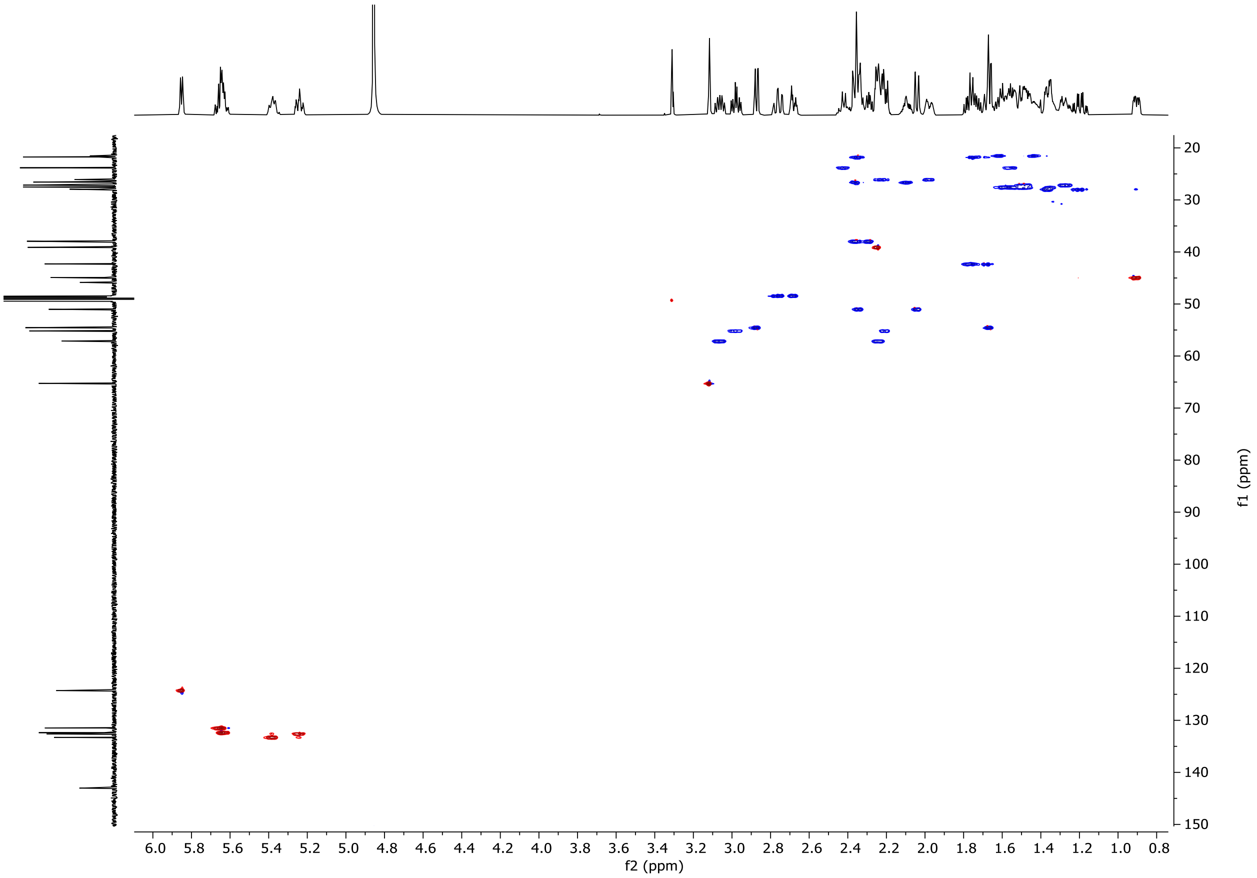

HMQC Spectrum of Keramaphidin B (+)-(1) ([D_4]-MeOH)

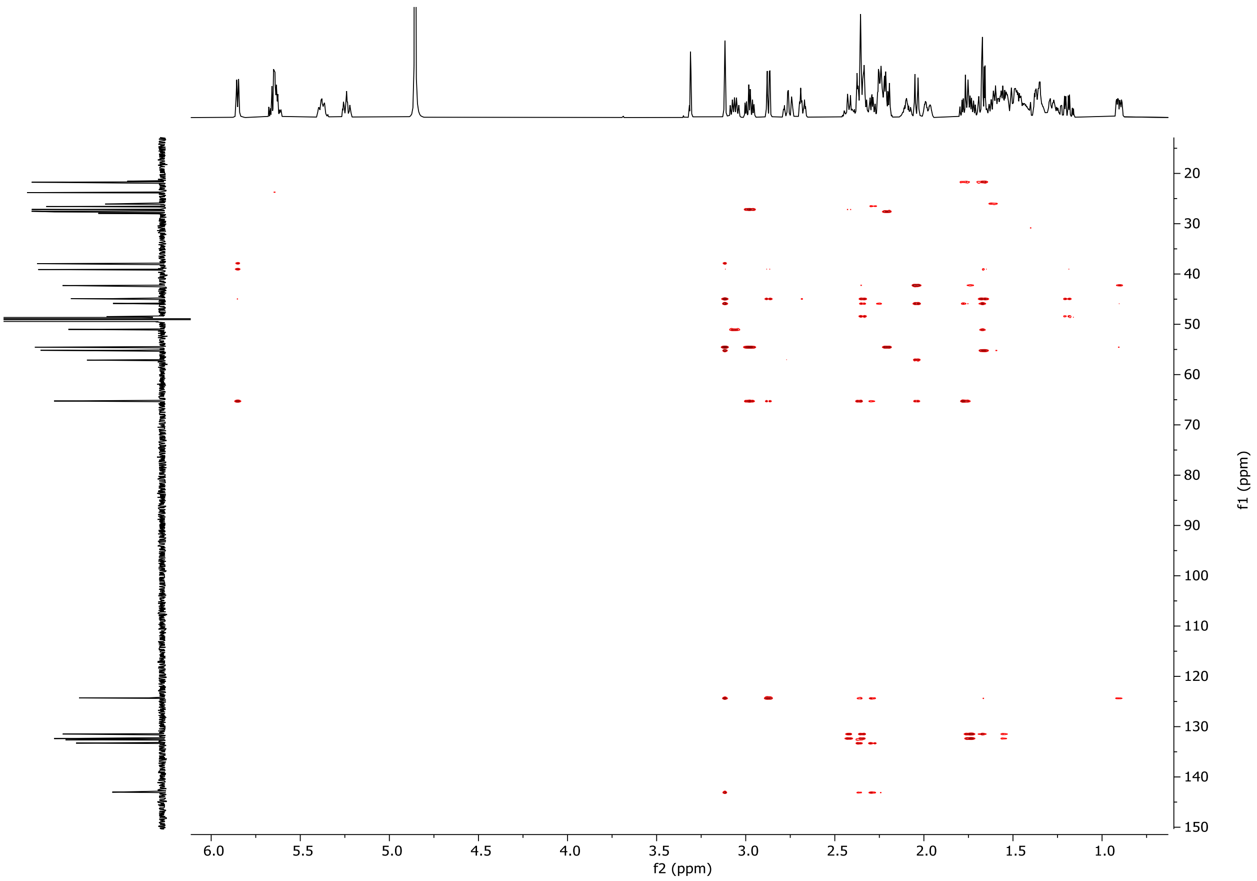

NOESY Spectrum of Keramaphidin B (+)-(1) ([D_4]-MeOH)

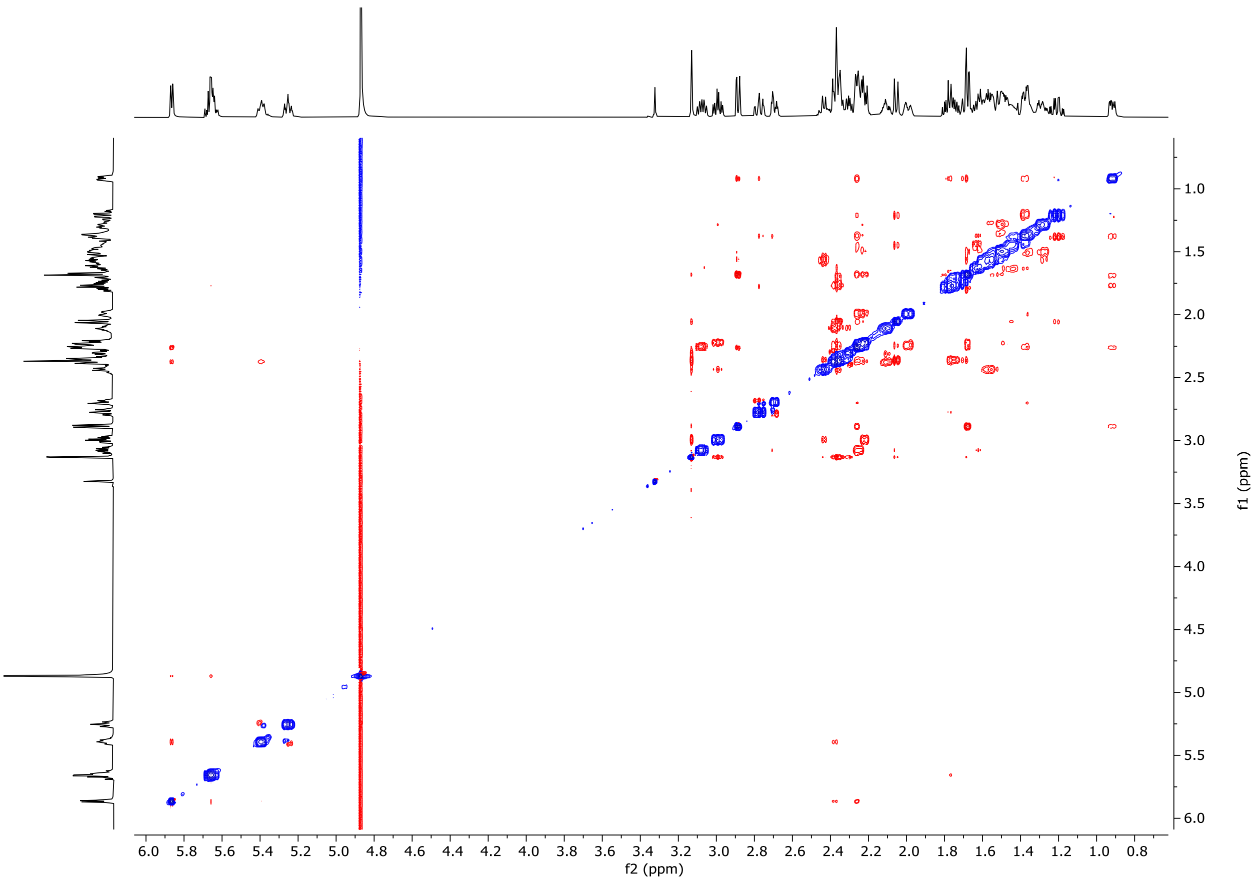

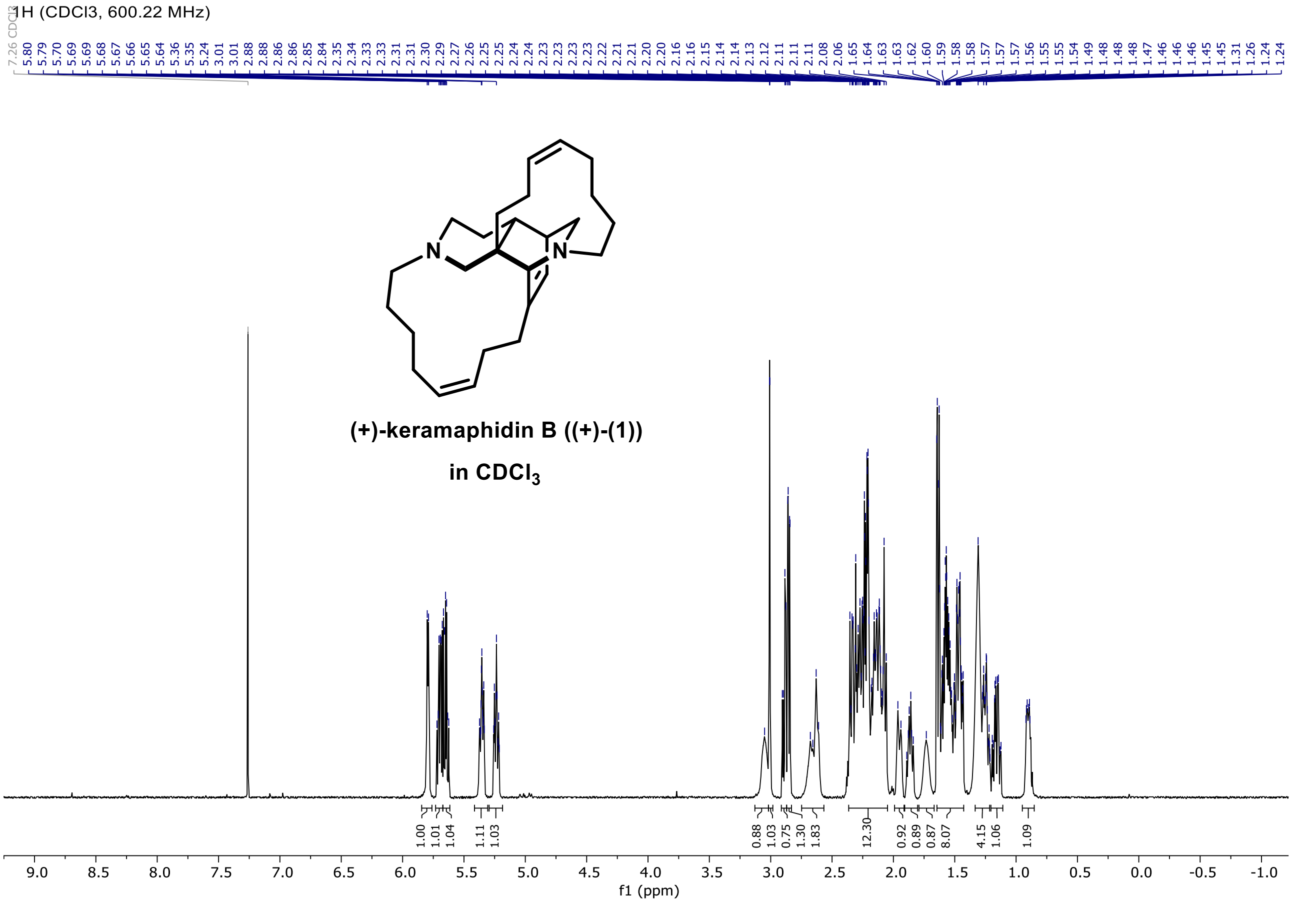
1H (CDCl3, 600.22 MHz)
7.26 CDCl3
5.80 5.79 5.70 5.69 5.69 5.68 5.67 5.66 5.65 5.64 5.36 5.35 5.24 3.01 3.01 2.88 2.88 2.86 2.86 2.85 2.84 2.35 2.34 2.33 2.33 2.31 2.31 2.30 2.29 2.27 2.26 2.25 2.25 2.24 2.24 2.23 2.23 2.23 2.23 2.22 2.21 2.21 2.20 2.16 2.16 2.15 2.14 2.14 2.13 2.12 2.11 2.11 2.11 2.11 2.08 2.06 1.65 1.64 1.63 1.63 1.62 1.60 1.59 1.58 1.58 1.57 1.57 1.56 1.55 1.55 1.54 1.49 1.48 1.48 1.47 1.46 1.46 1.45 1.31 1.26 1.24 1.24
N
N
(+)-keramaphidin B ((+)-(1))
in CDCl3
1.00 1.01 1.04 1.11 1.03 0.88 1.03 0.75 1.30 1.83 12.30 0.92 0.89 0.87 8.07 4.15 1.06 1.09
9.0 8.5 8.0 7.5 7.0 6.5 6.0 5.5 5.0 4.5 4.0 3.5 3.0 2.5 2.0 1.5 1.0 0.5 0.0 -0.5 -1.0
f1 (ppm)

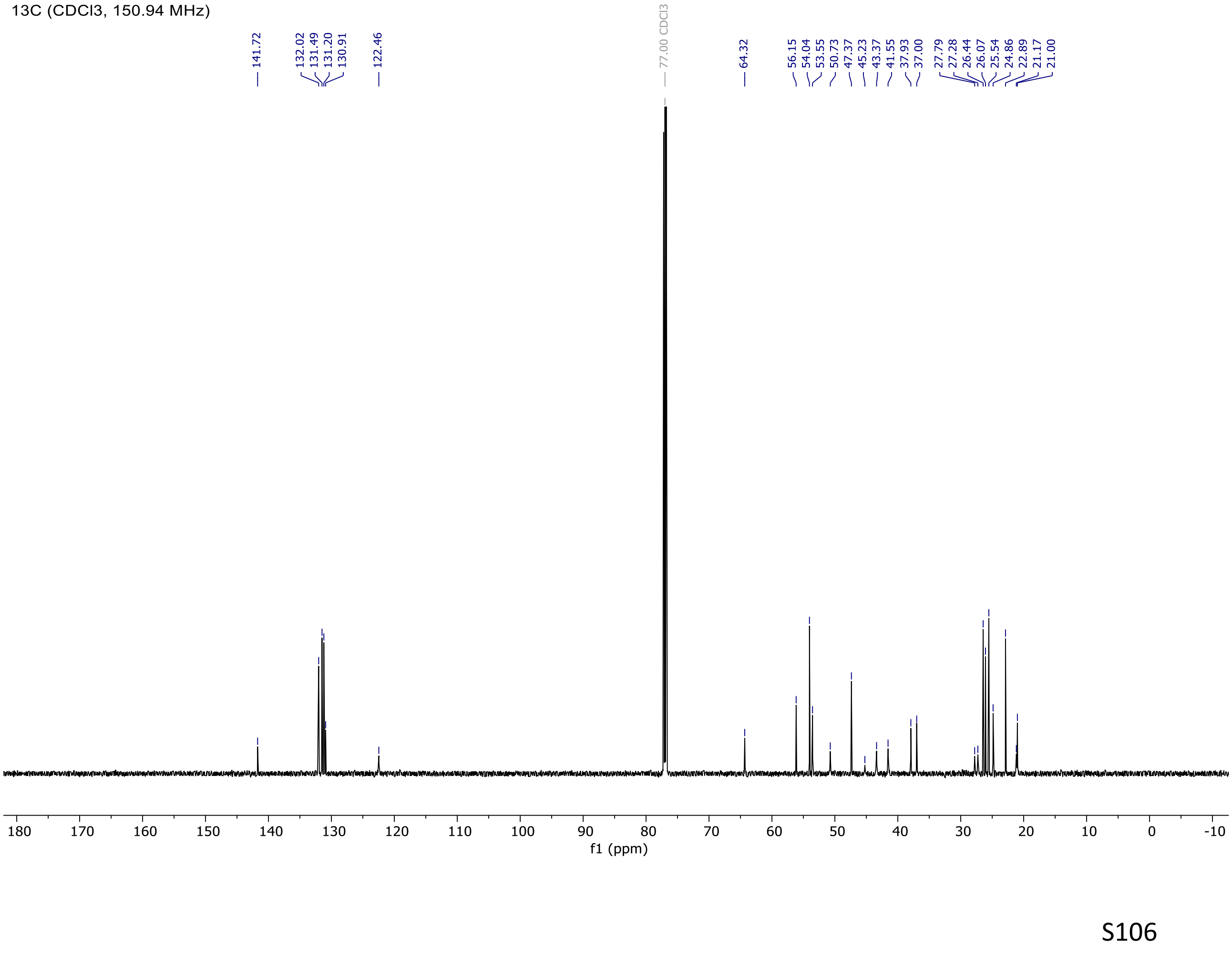
13C (CDCl3, 150.94 MHz)
141.72 132.02 131.49 131.20 130.91 122.46
77.00 CDCl3
64.32 56.15 54.04 53.55 50.73 47.37 45.23 43.37 41.55 37.93 37.00 27.79 27.28 26.44 26.07 25.54 24.86 22.89 21.17 21.00
180 170 160 150 140 130 120 110 100 90 80 70 60 50 40 30 20 10 0 -10
f1 (ppm)

COSY Spectrum of Keramaphidin B (+)-1 ($CDCl_3$)

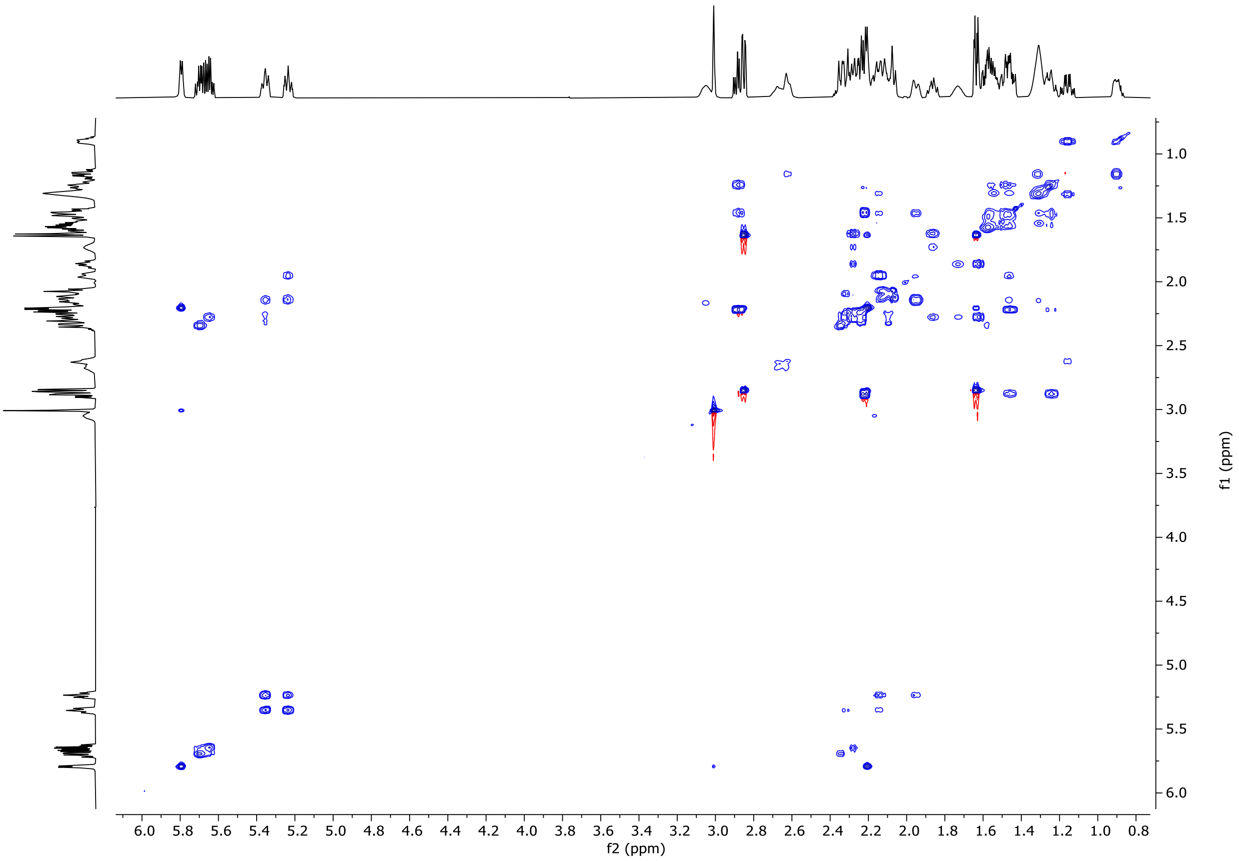

HSQC Spectrum of Keramaphidin B (+)-1 ($CDCl_3$)

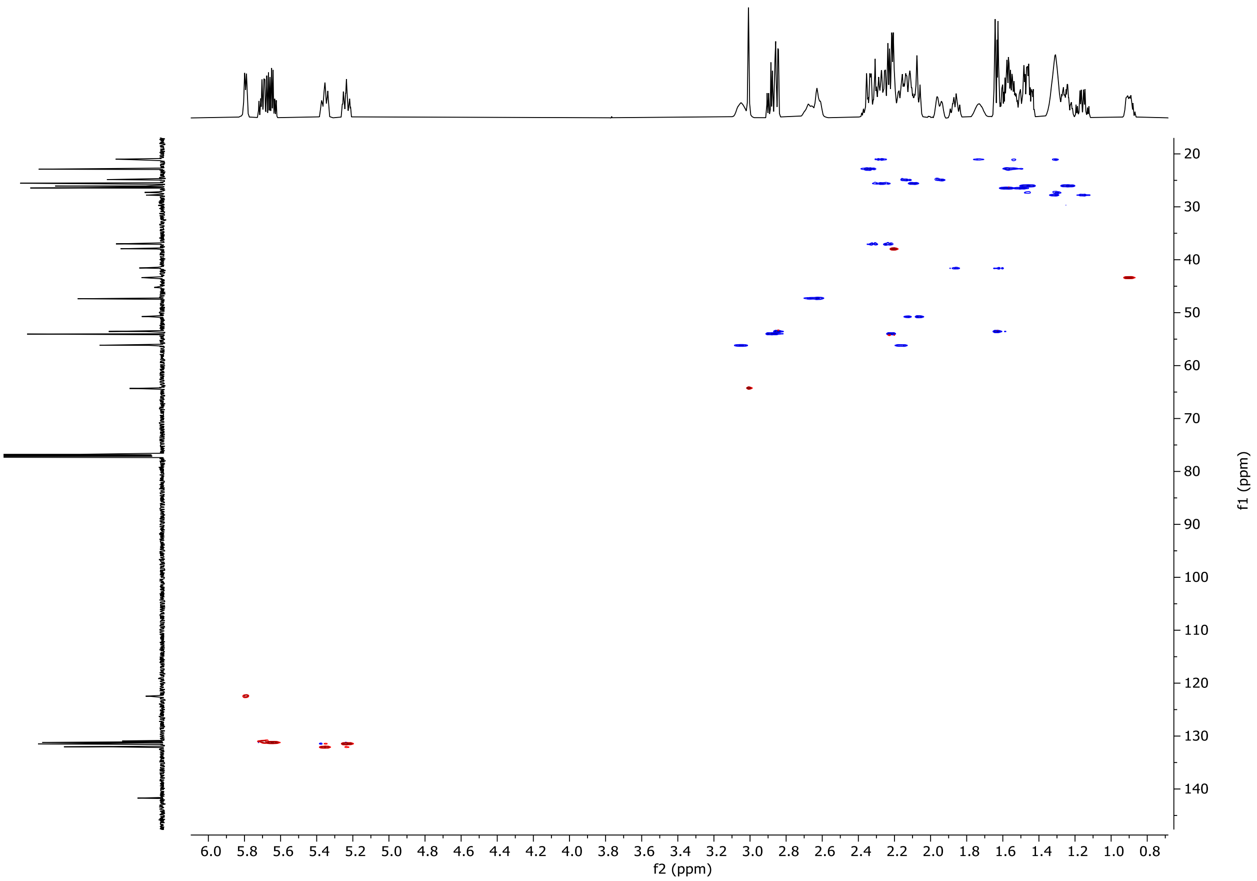

HMQC Spectrum of Keramaphidin B (+)-1 ($CDCl_3$)

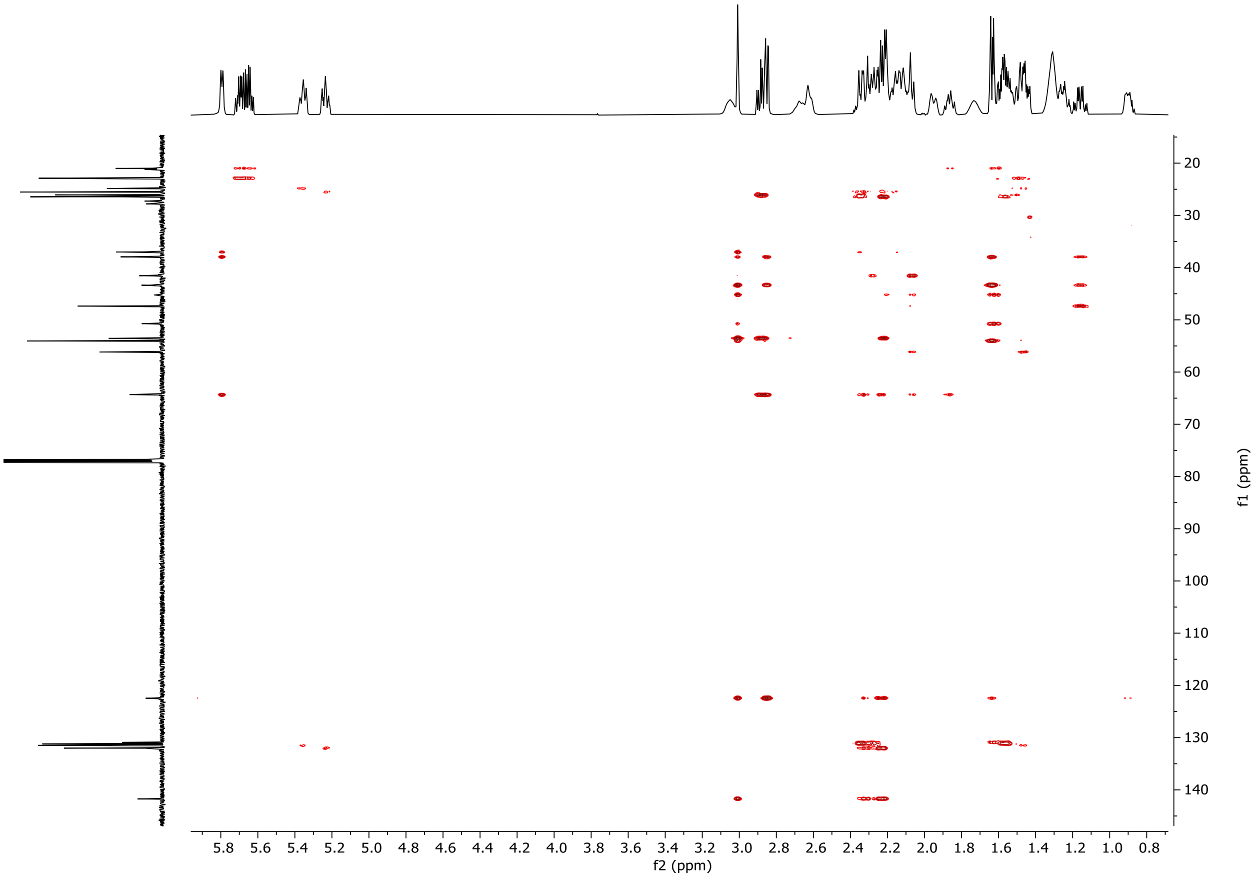

NOESY Spectrum of Keramaphidin B (+)-1 ($CDCl_3$)

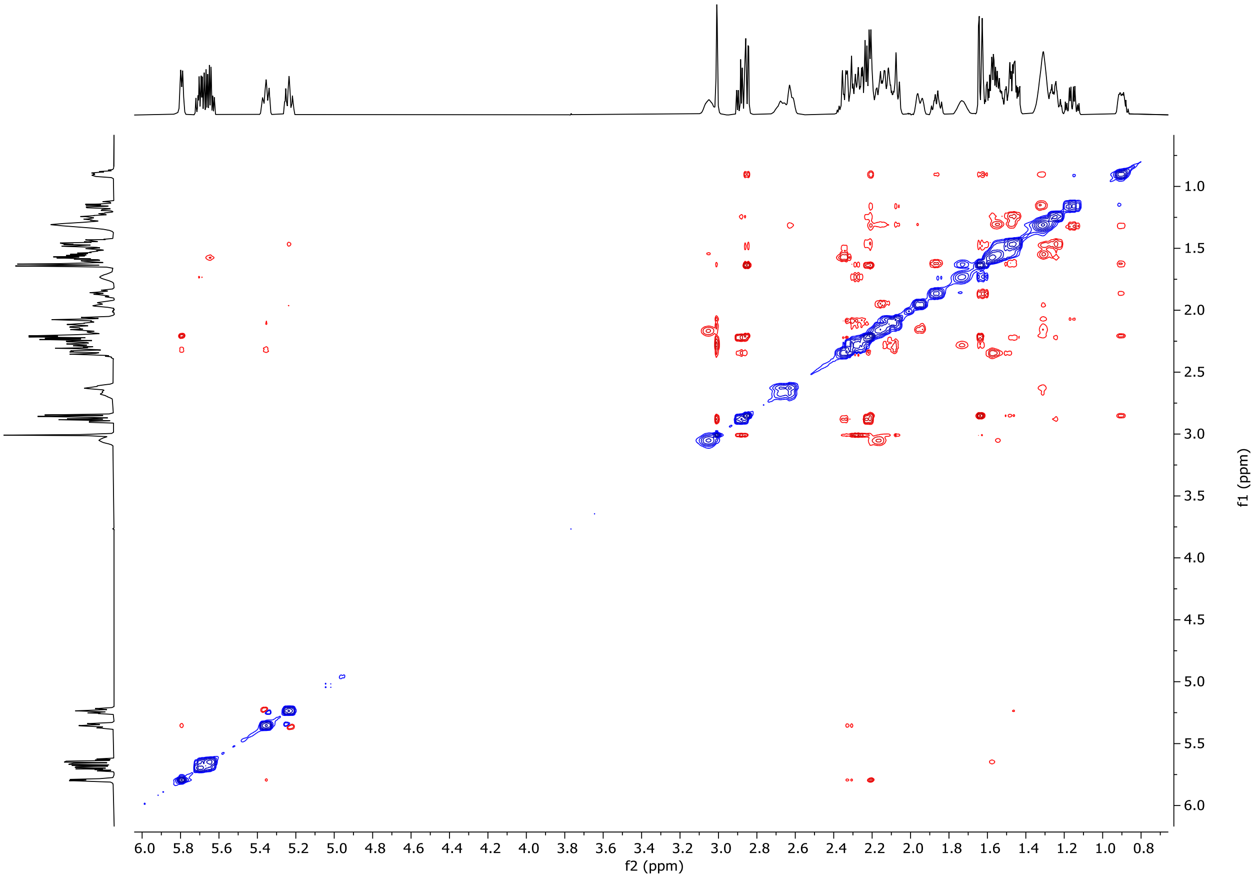

^{1}H NMR Spectrum of 45 (400 MHz, $CDCl_3$)

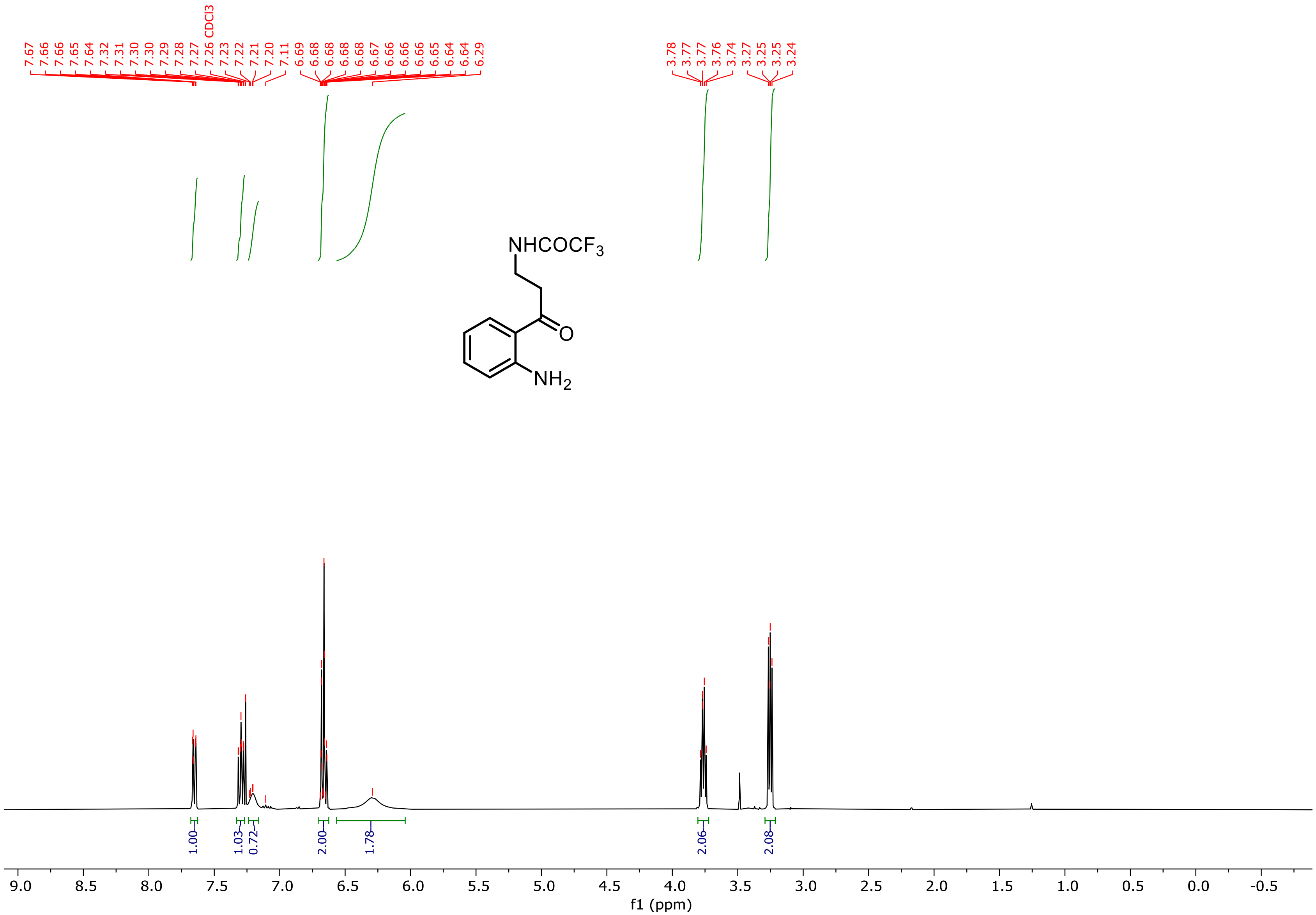

^{13}C NMR Spectrum of 45 (101 MHz, $CDCl_3$)

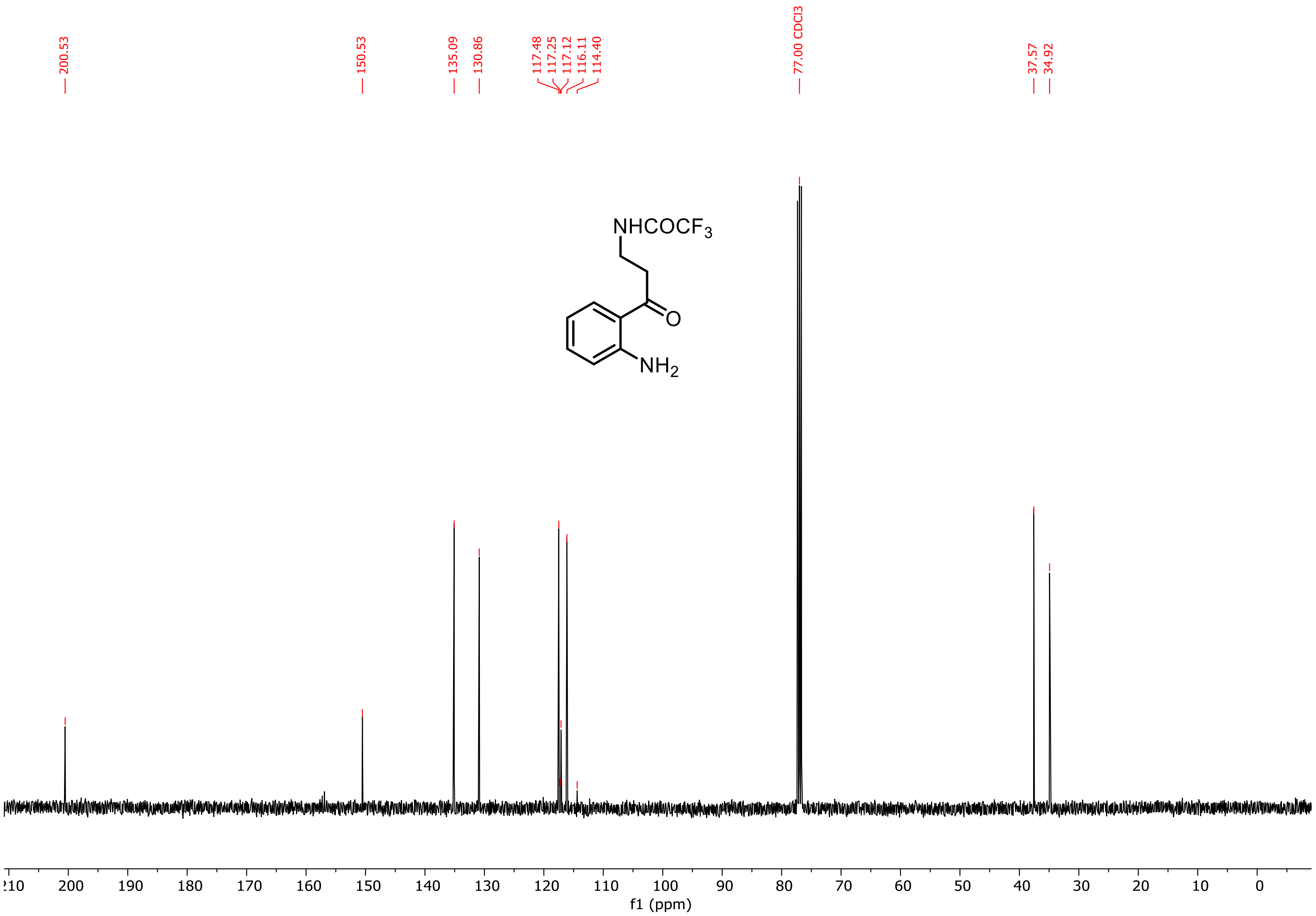

^{19}F NMR Spectrum of 45 (282 MHz, $CDCl_3$)

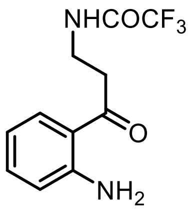

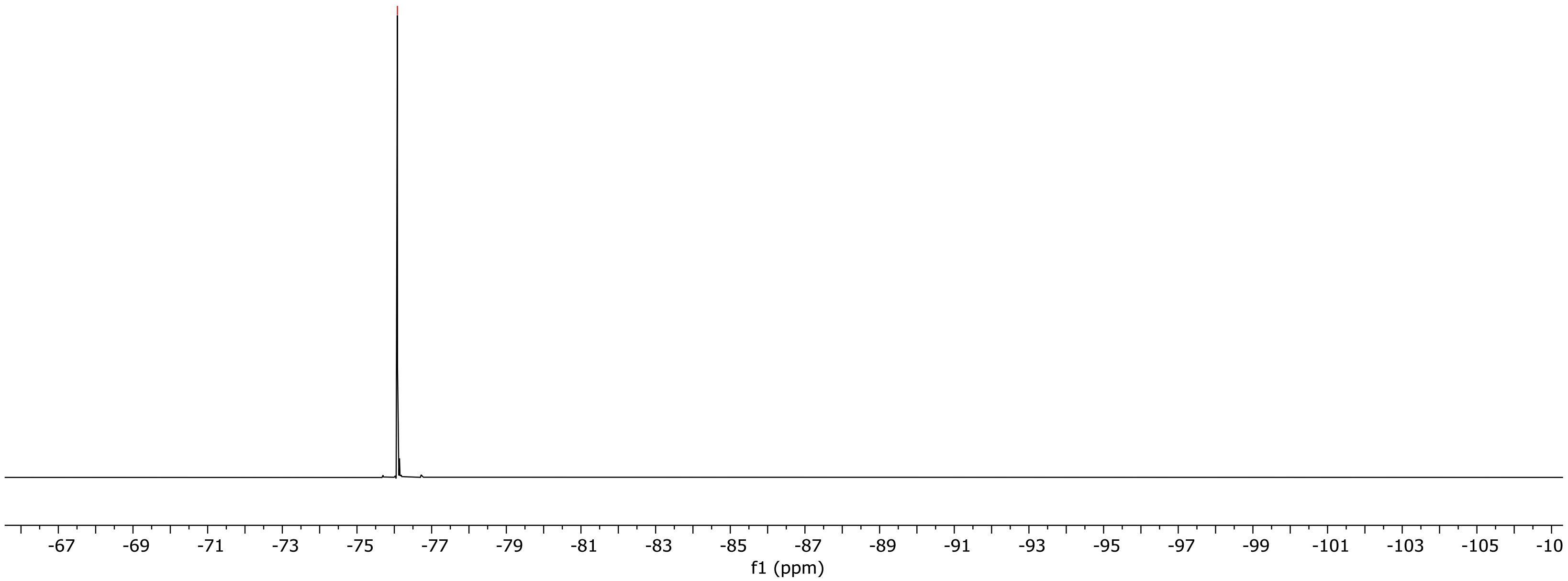

^{1}H NMR Spectrum of 47 (400 MHz, [D$_4$]-MeOH)

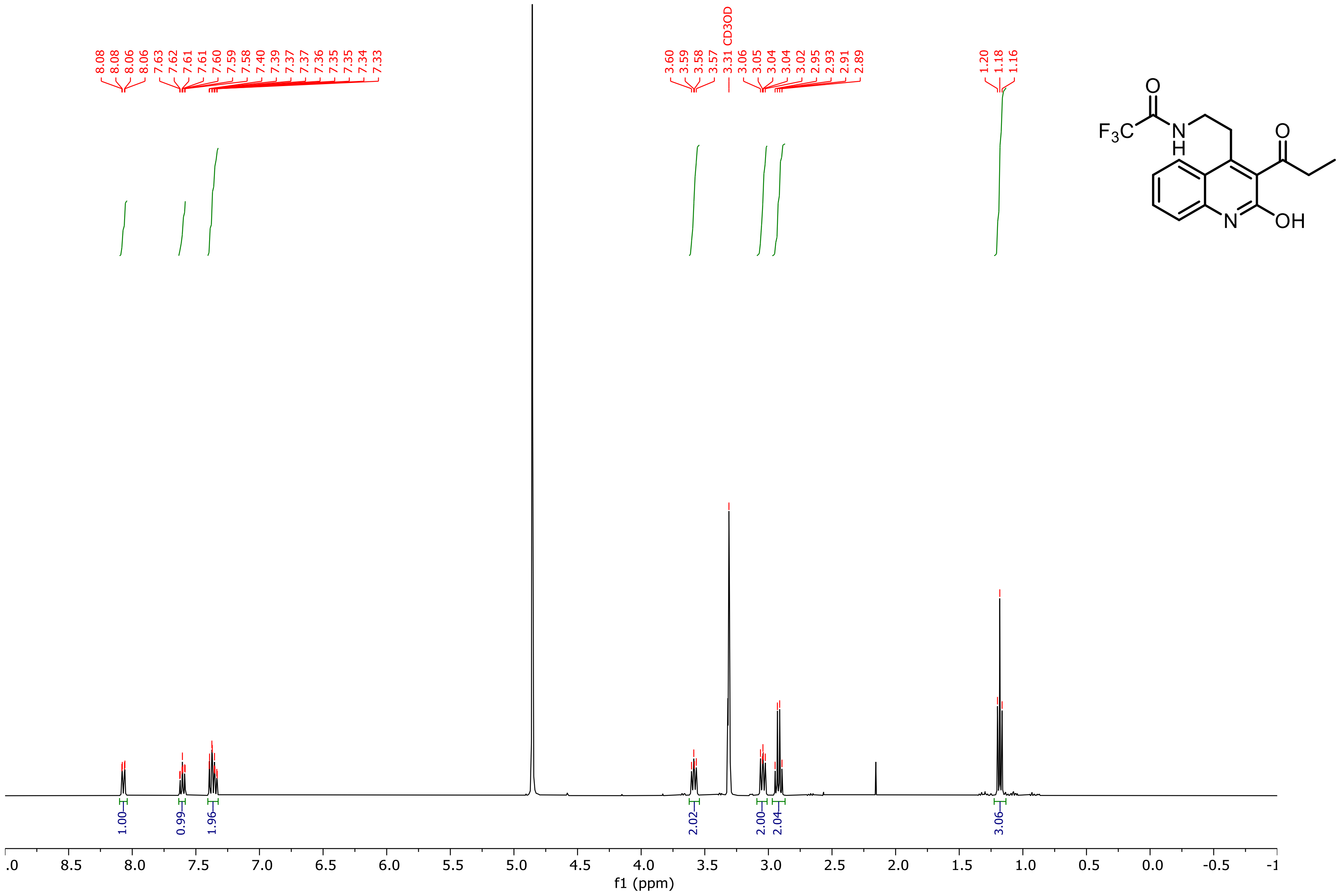

^{13}C NMR Spectrum of 47 (101 MHz, [D$_4$]-MeOH)

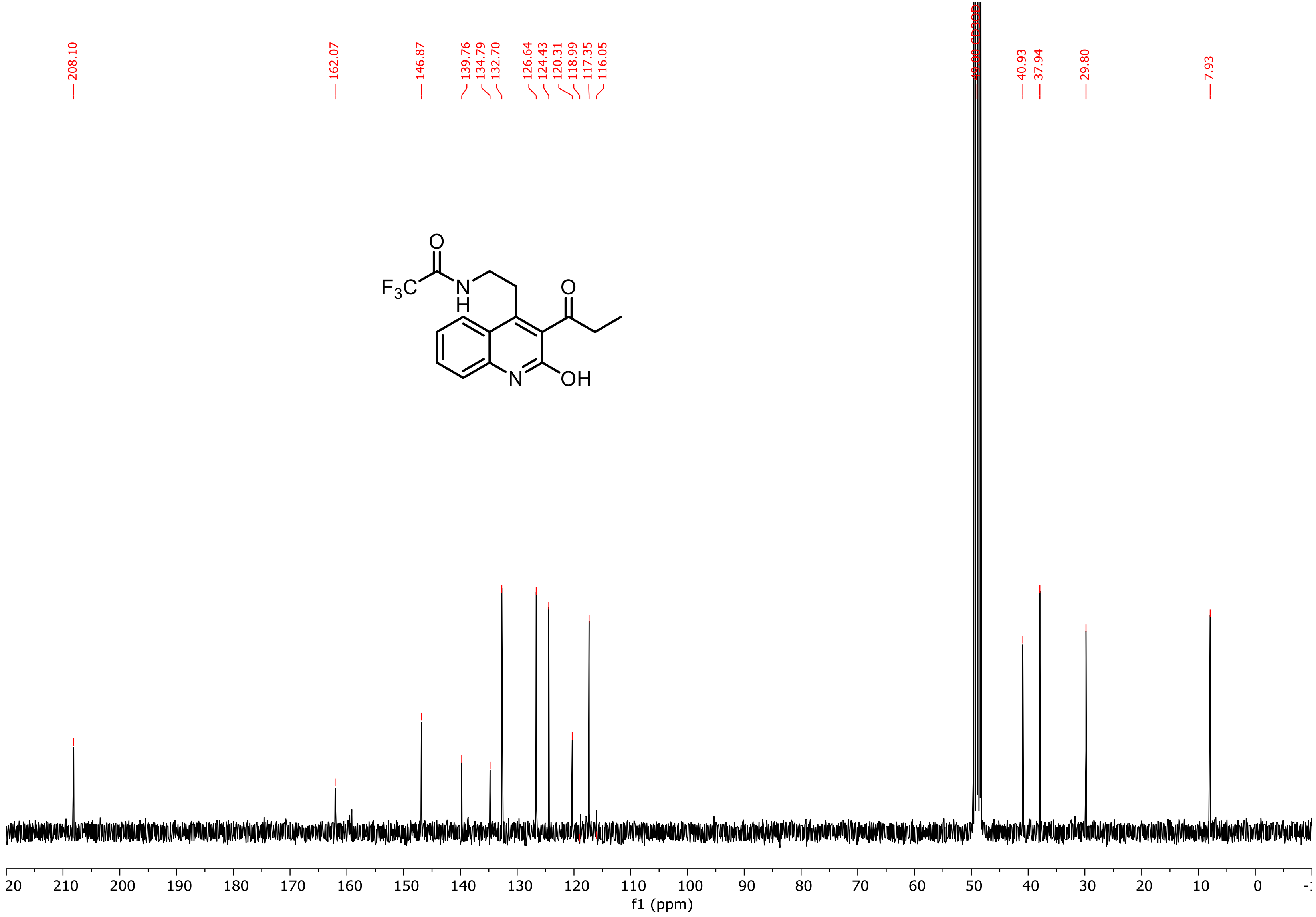

^{19}F NMR Spectrum of 47 (282 MHz, [D$_4$]-MeOH)

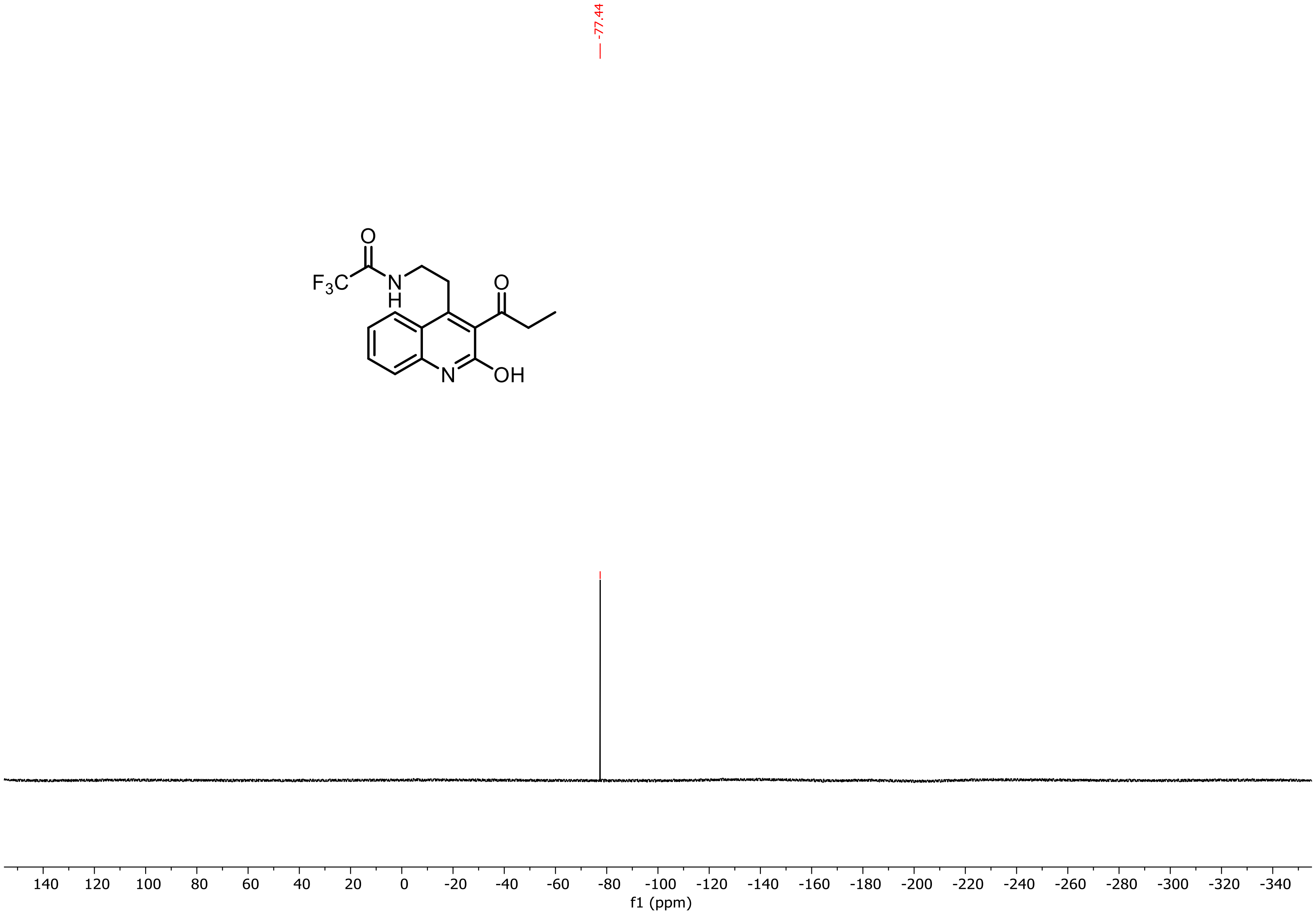

¹H NMR Spectrum of 48 (400 MHz, $CDCl_3$)

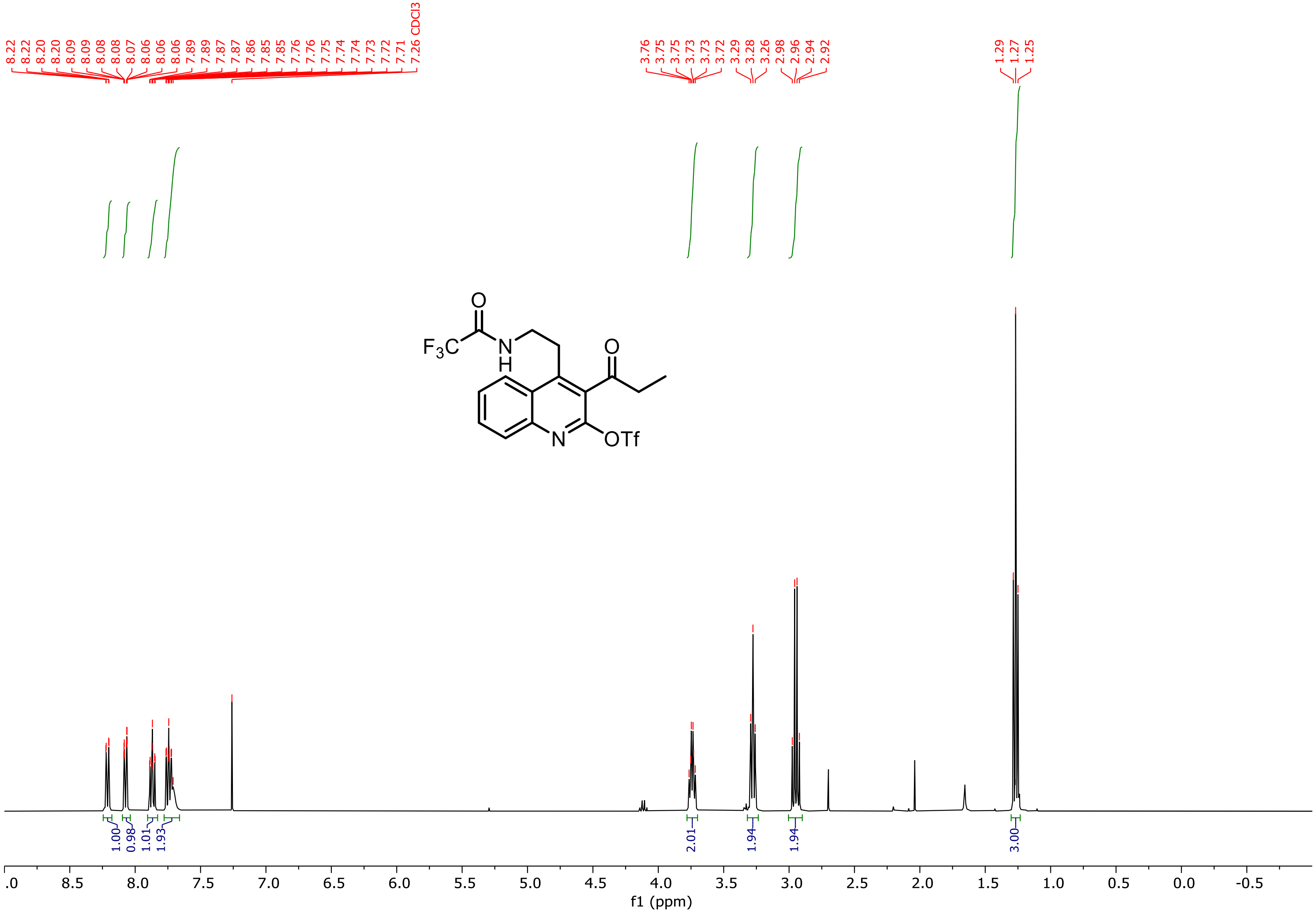

^{13}C NMR Spectrum of 48 (101 MHz, $CDCl_3$)

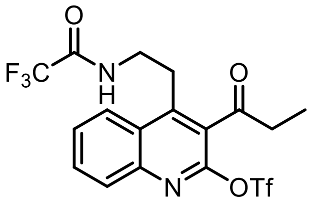

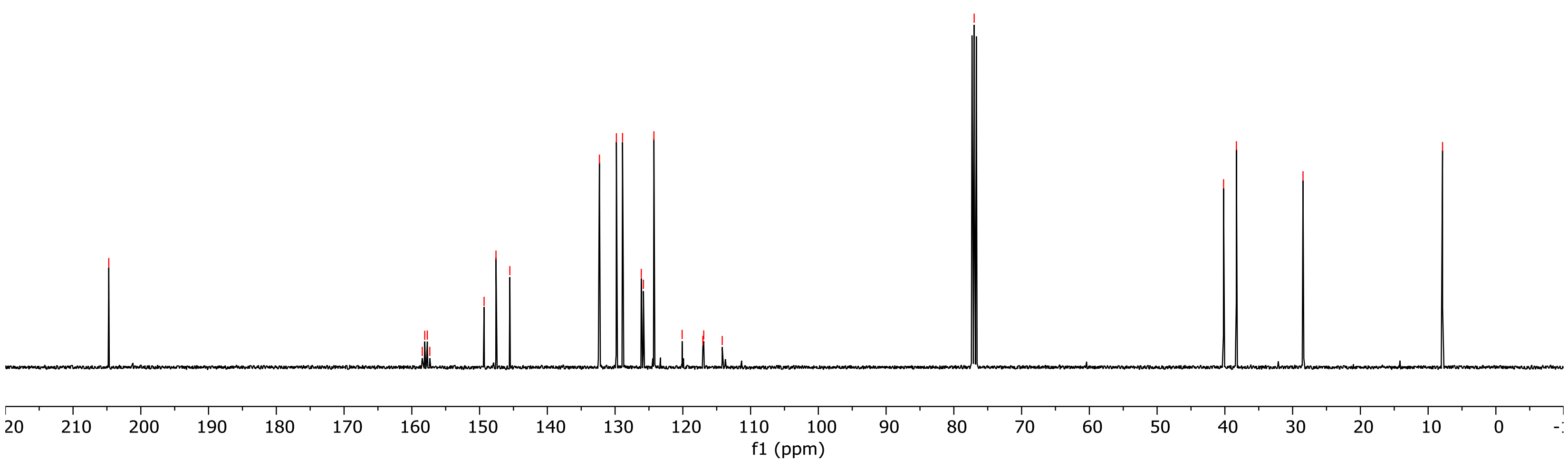

^{19}F NMR Spectrum of 48 (282 MHz, CDCl$_3$)

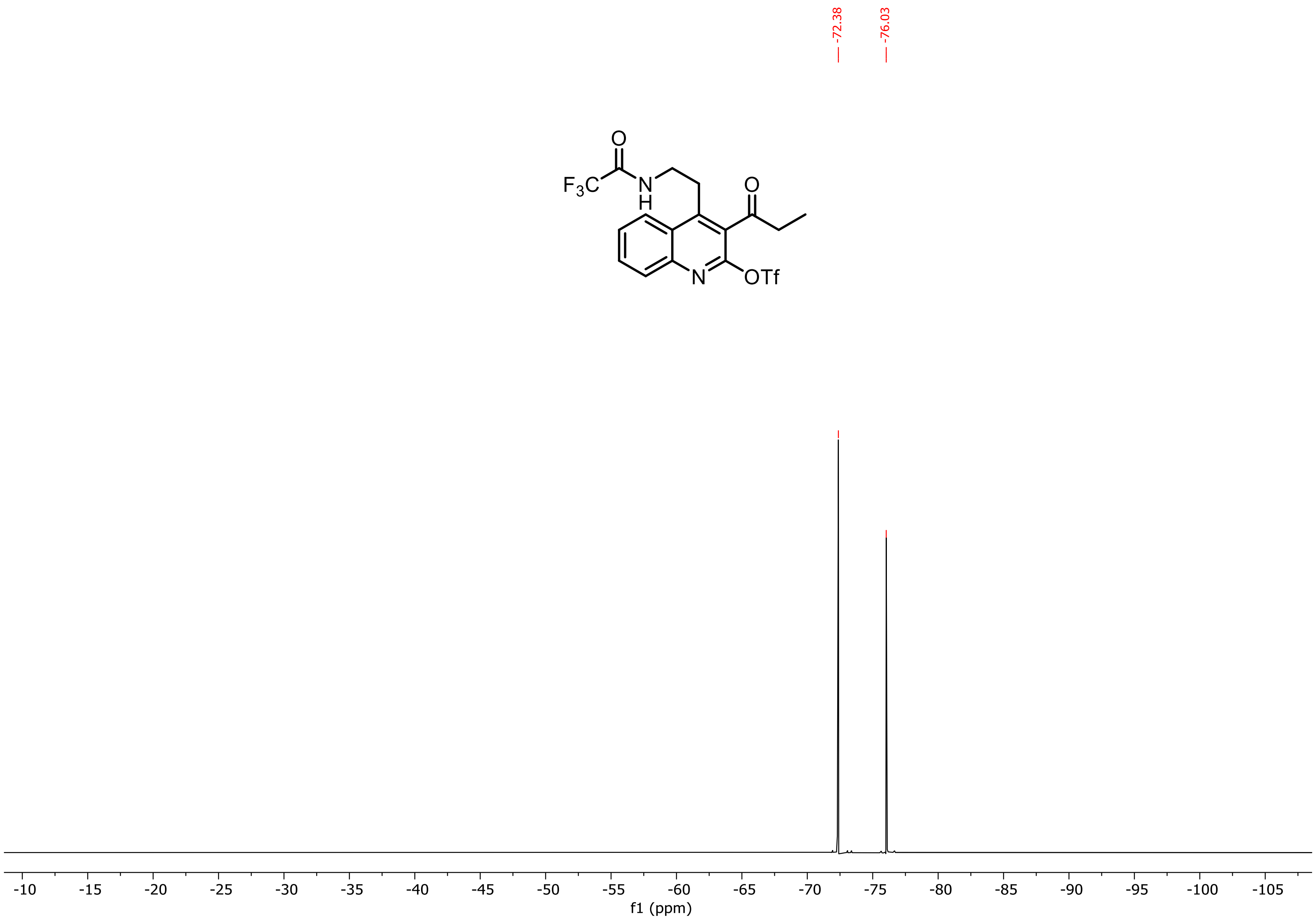

^{1}H NMR Spectrum of 50 (400 MHz, $CDCl_3$)

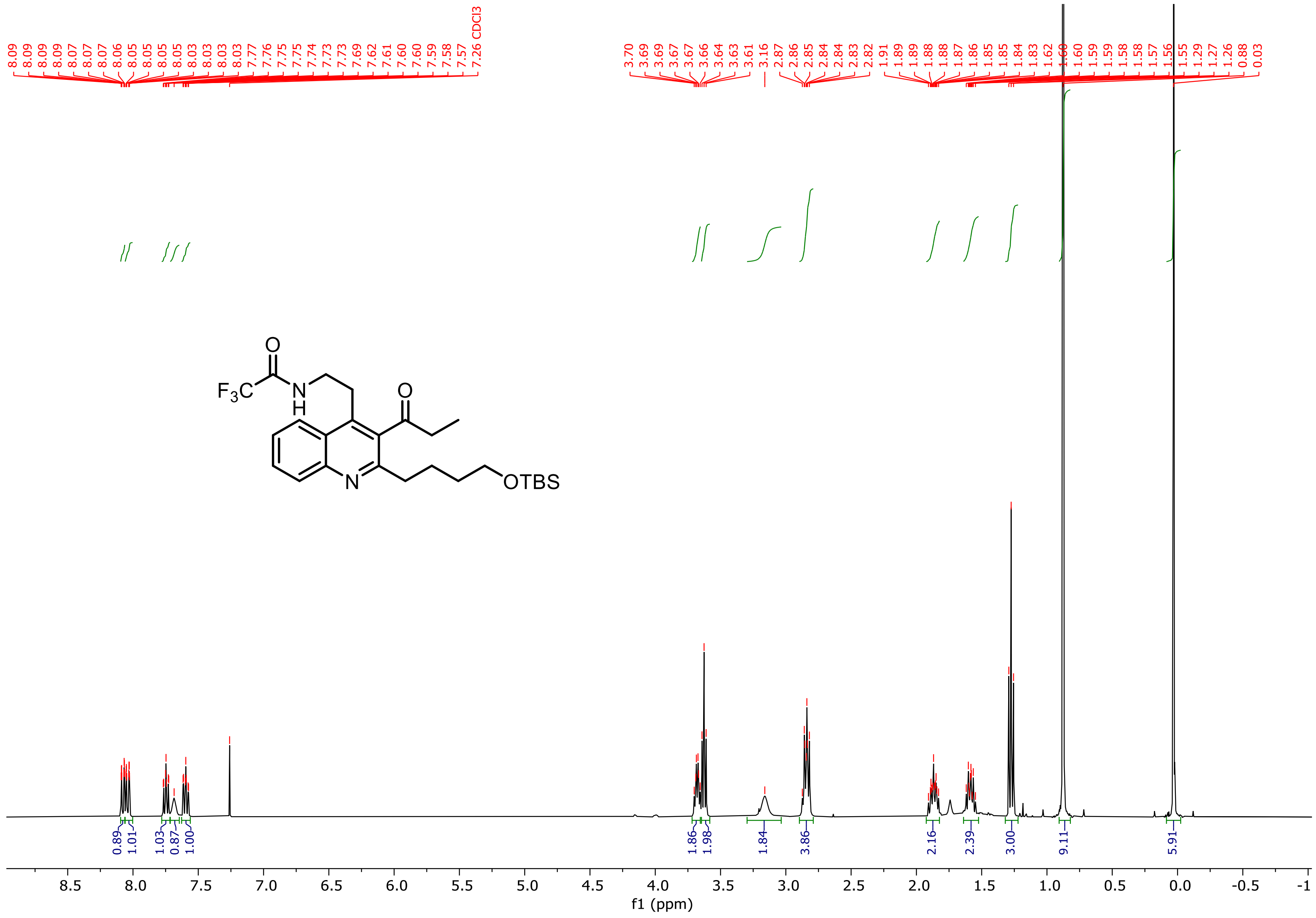

^{13}C NMR Spectrum of 50 (101 MHz, $CDCl_3$)

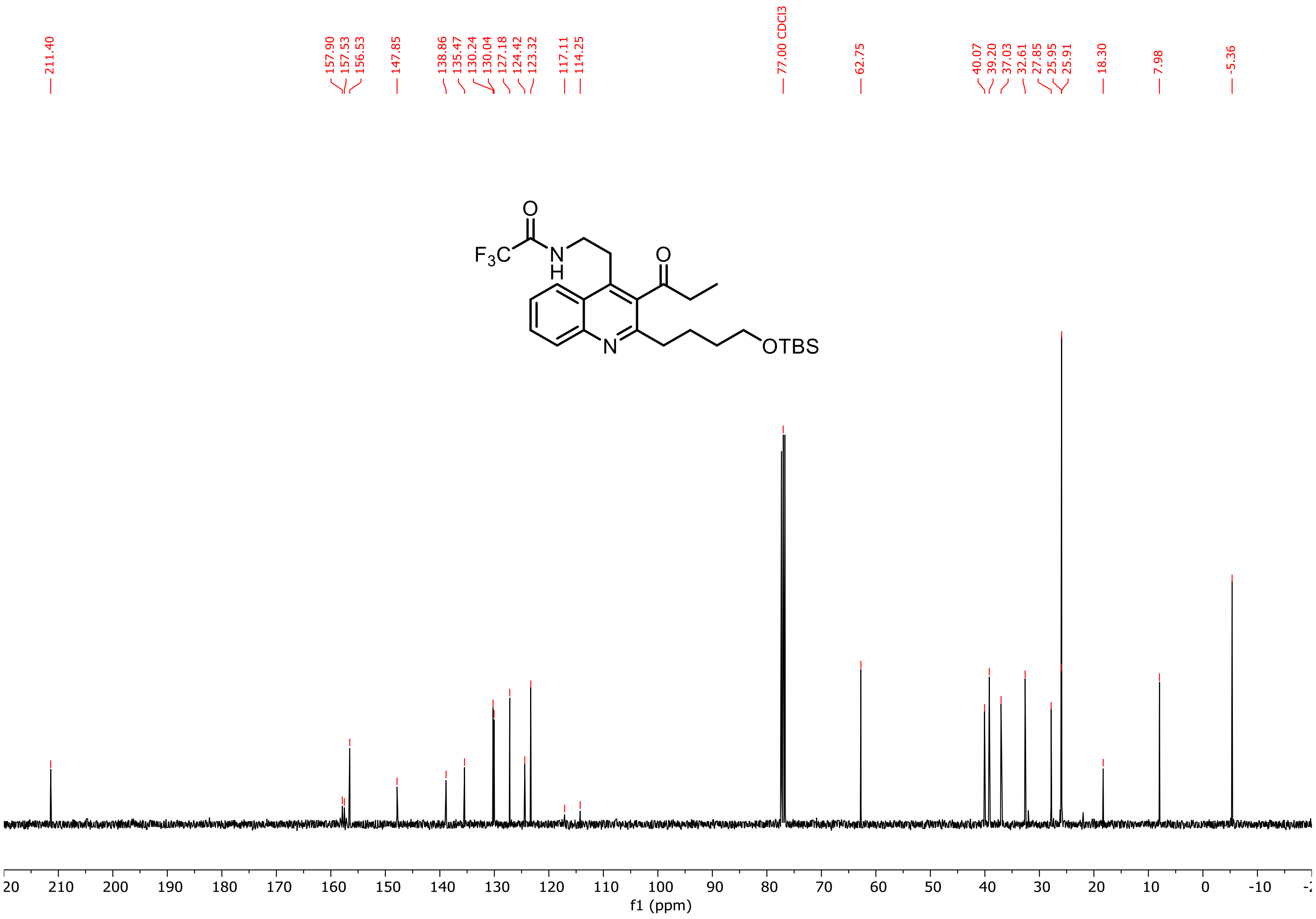

^{19}F NMR Spectrum of 50 (282 MHz, CDCl$_3$)

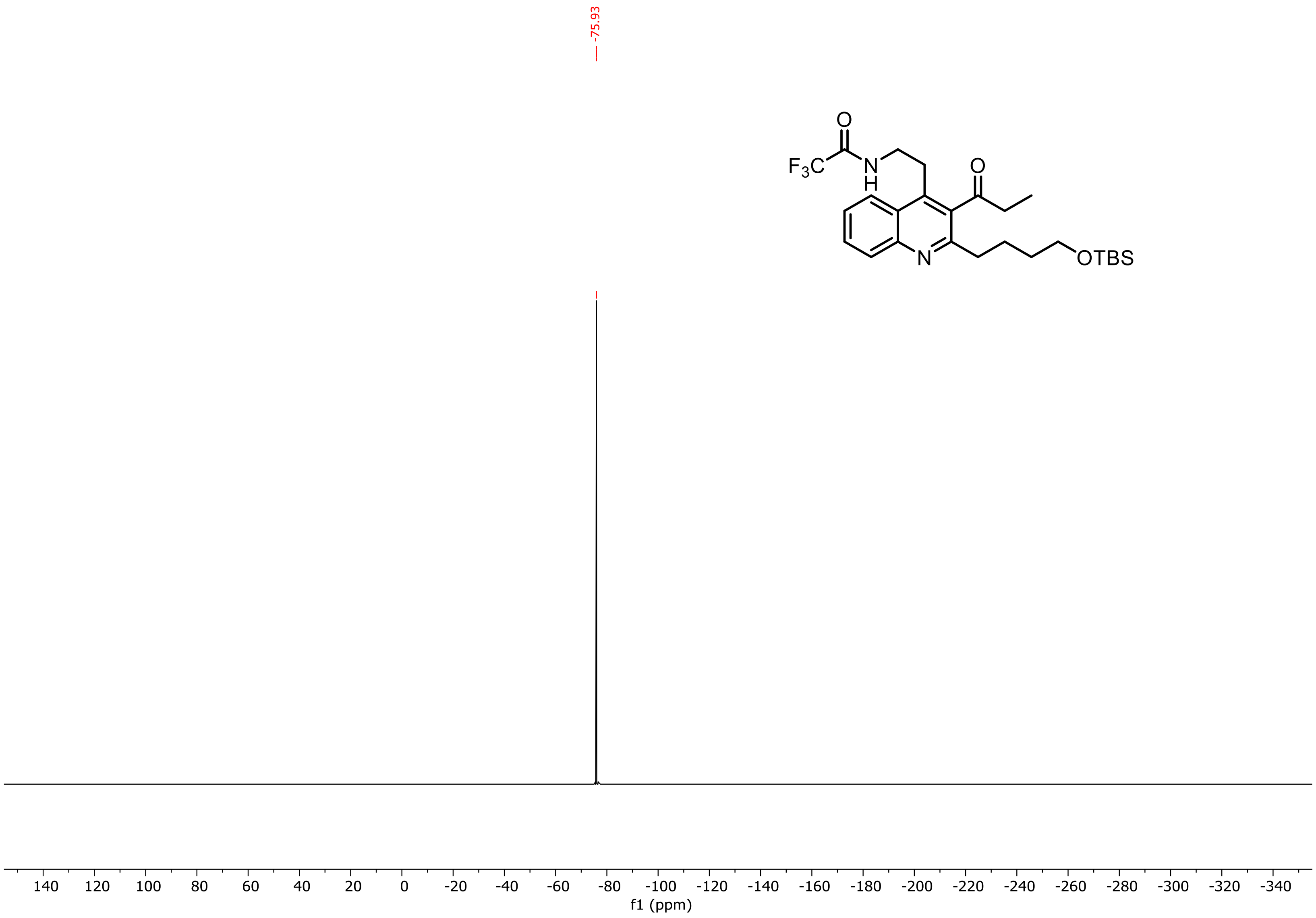

^{1}H NMR Spectrum of S1 (400 MHz, $CDCl_3$)

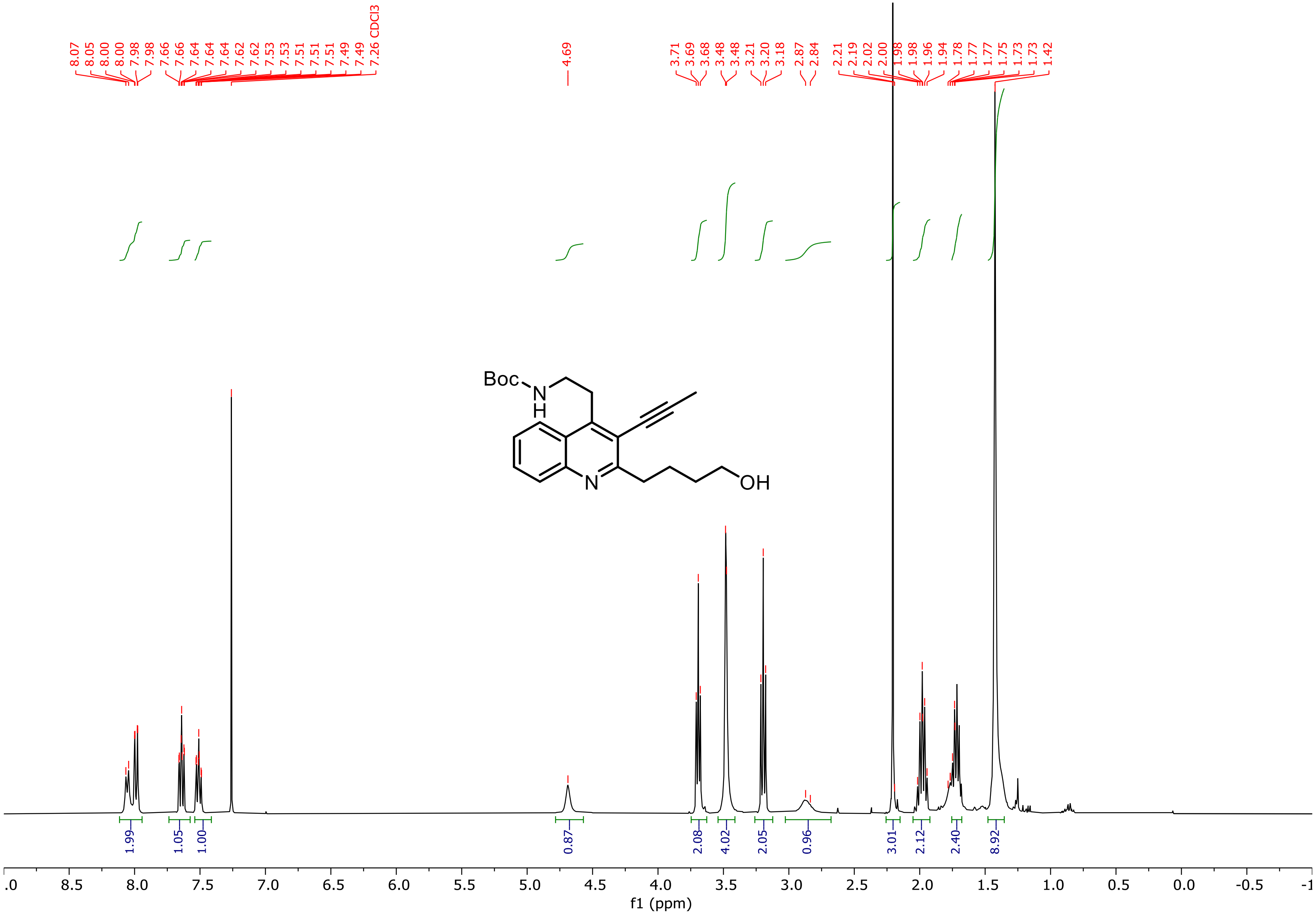

^{13}C NMR Spectrum of S1 (101 MHz, CDCl$_3$)

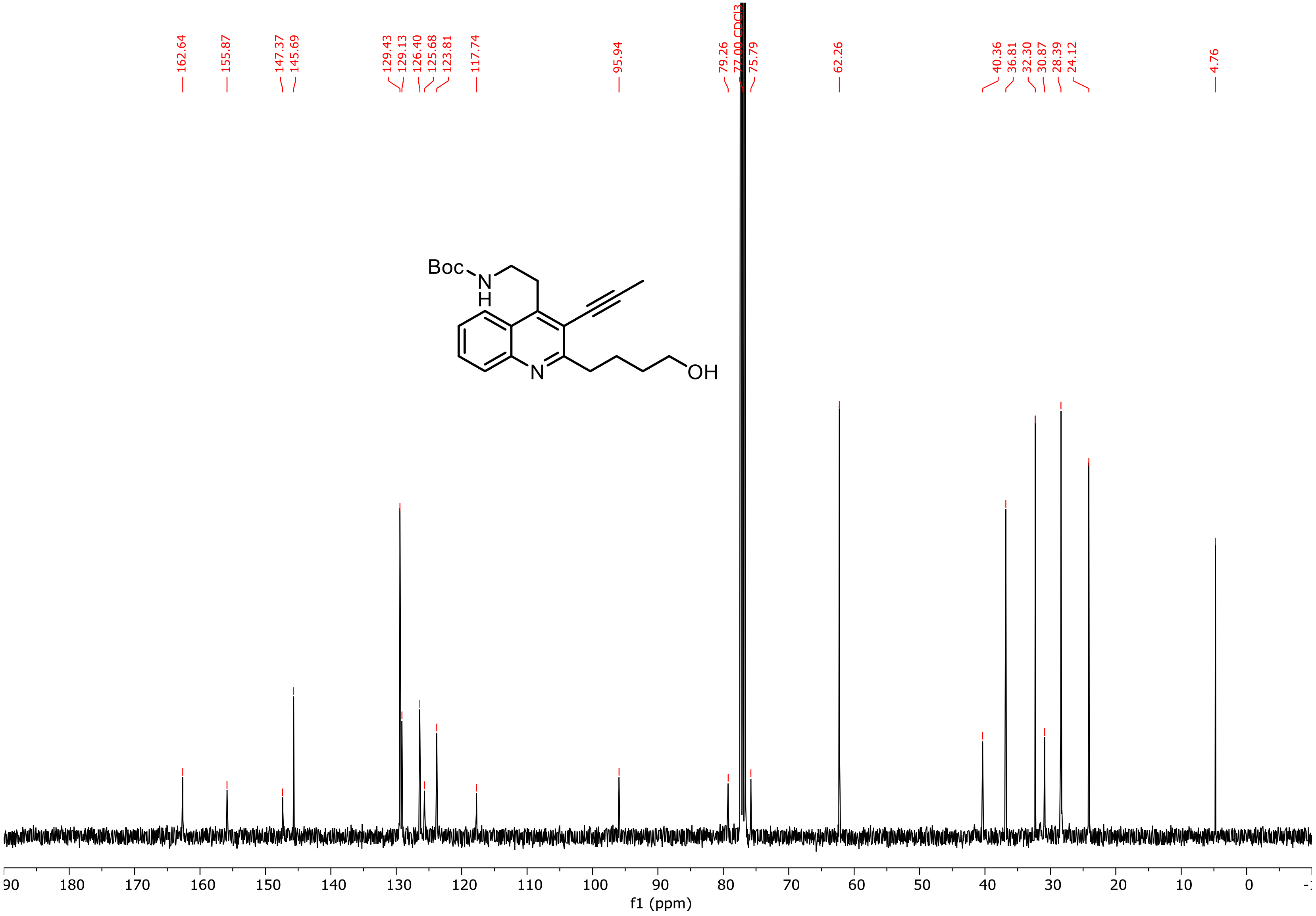

^{1}H NMR Spectrum of 52 (400 MHz, $CDCl_3$)

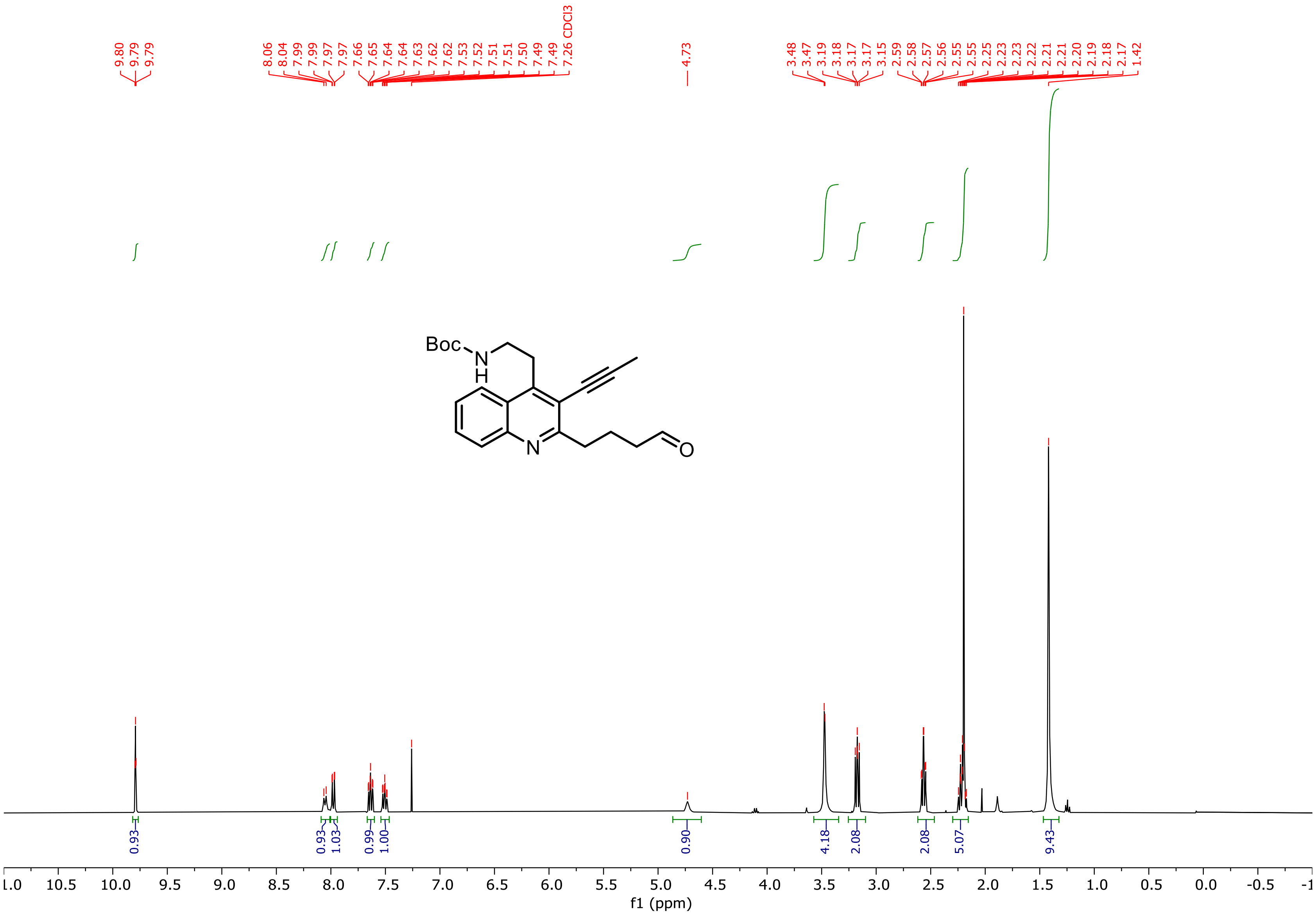

^{13}C NMR Spectrum of 52 (101 MHz, $CDCl_3$)

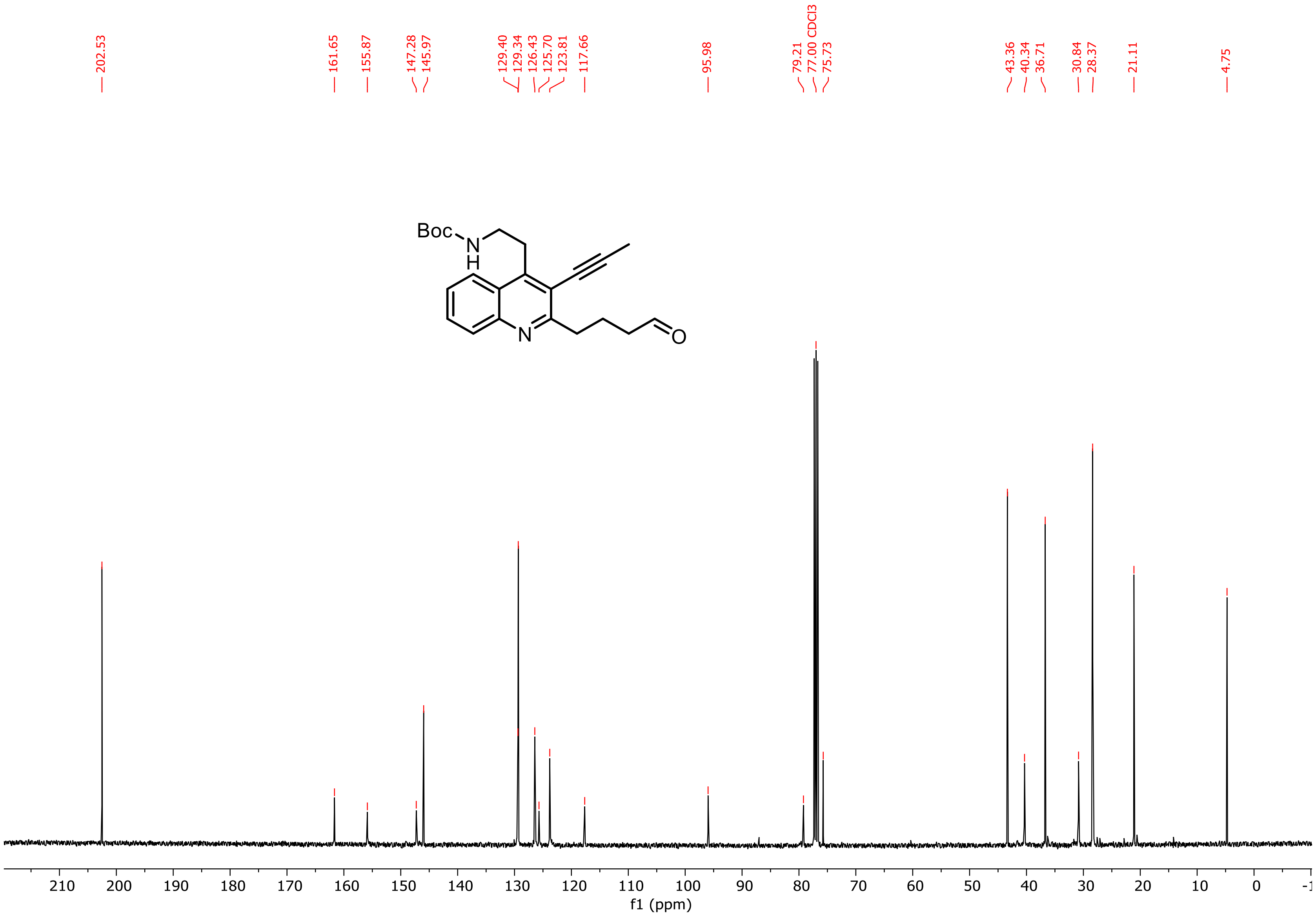

^{1}H NMR Spectrum of S4 (400 MHz, $CDCl_3$, mixture)

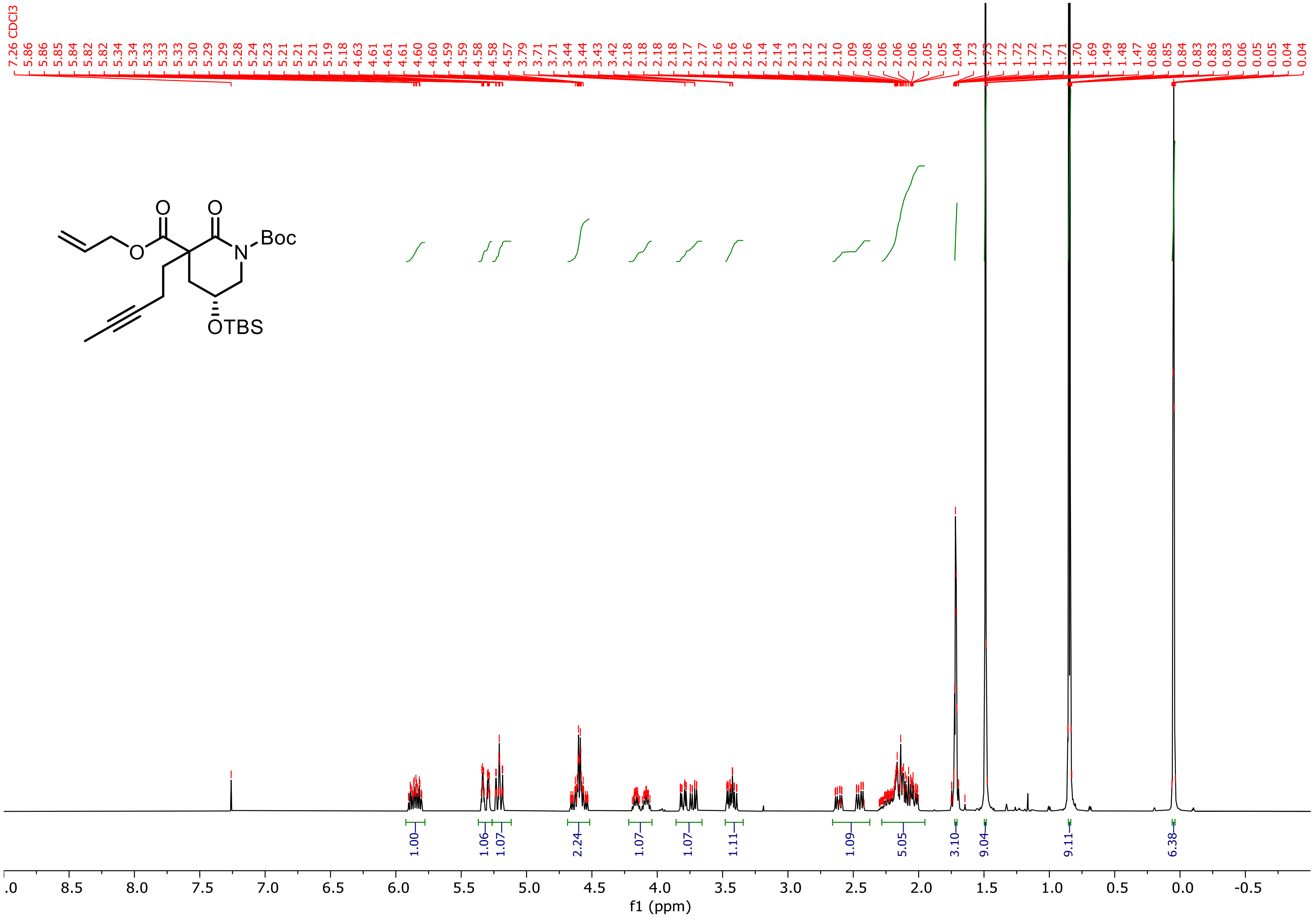

^{13}C NMR Spectrum of S4 (101 MHz, $CDCl_3$)

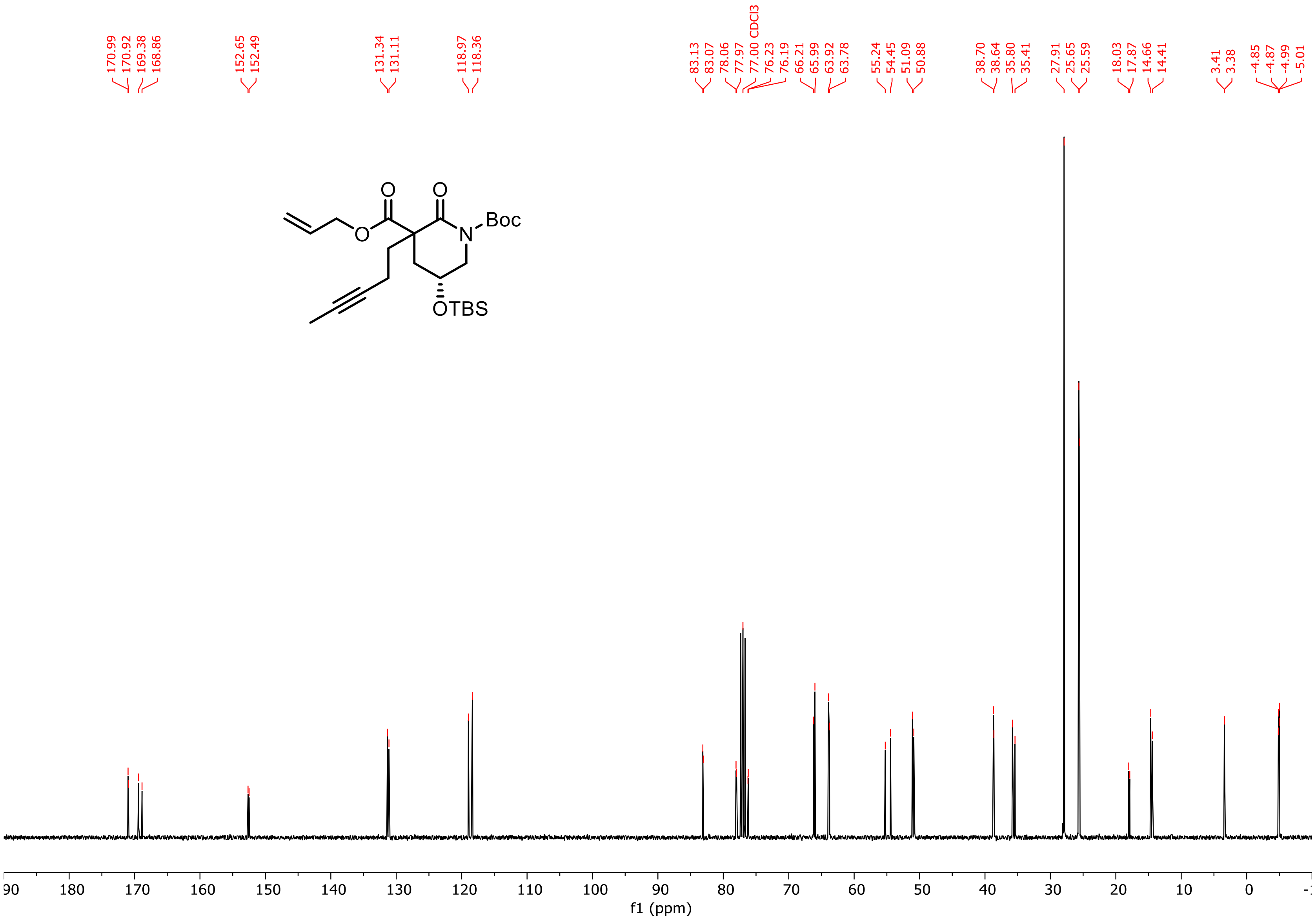

^{1}H NMR Spectrum of 53 (400 MHz, $CDCl_3$)

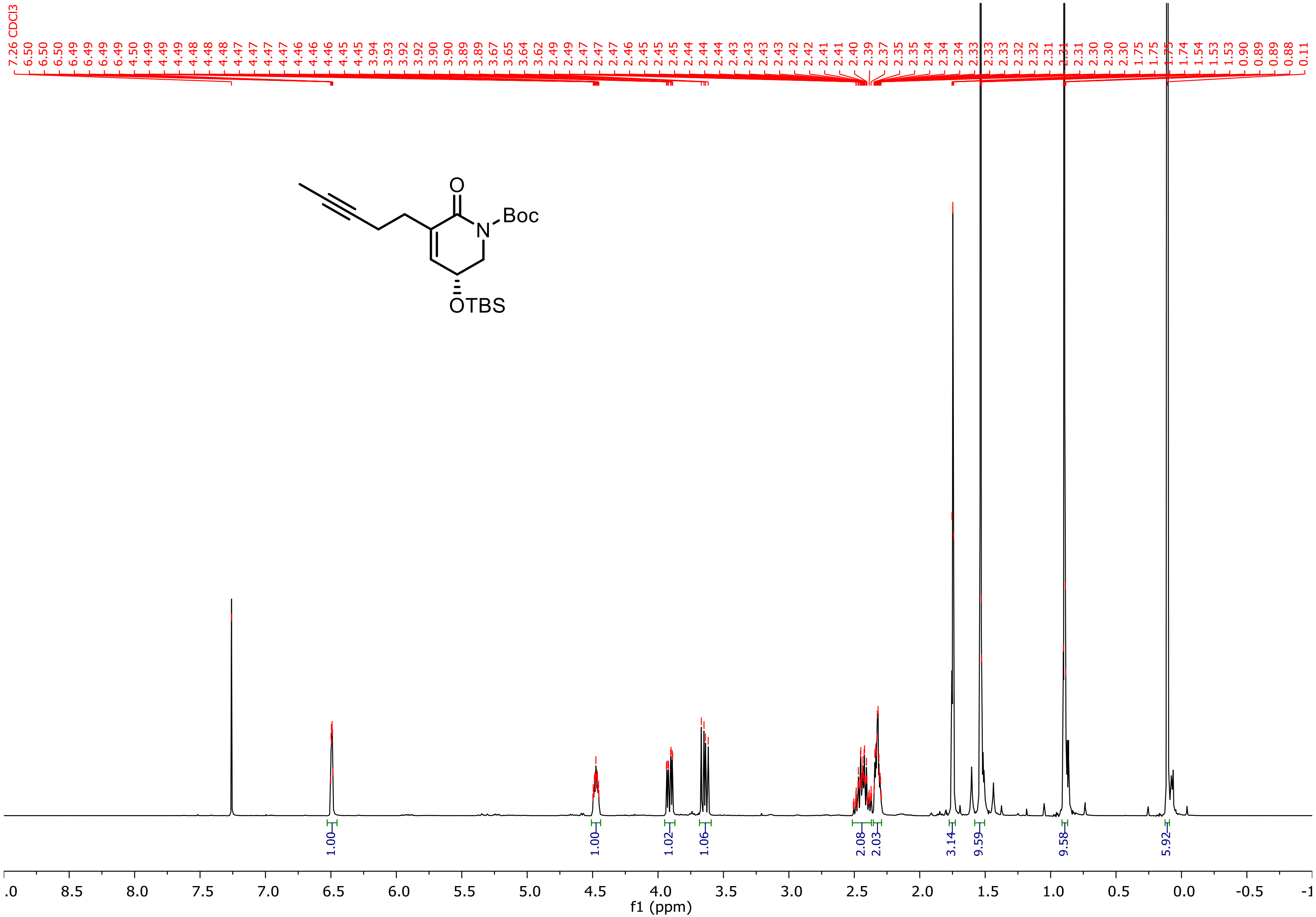

^{13}C NMR Spectrum of 53 (101 MHz, $CDCl_3$)

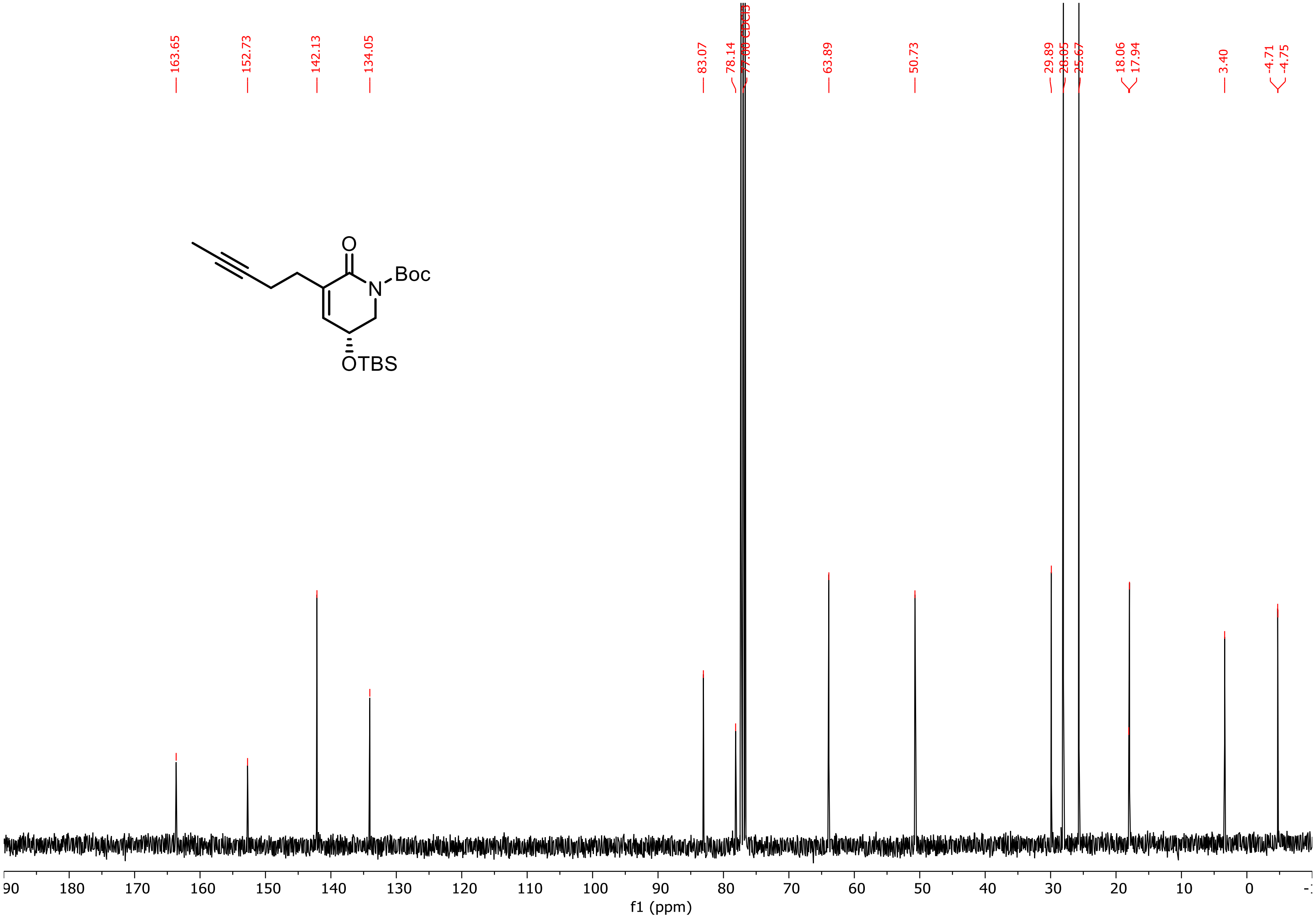

^{1}H NMR Spectrum of S5 (400 MHz, $CDCl_3$)

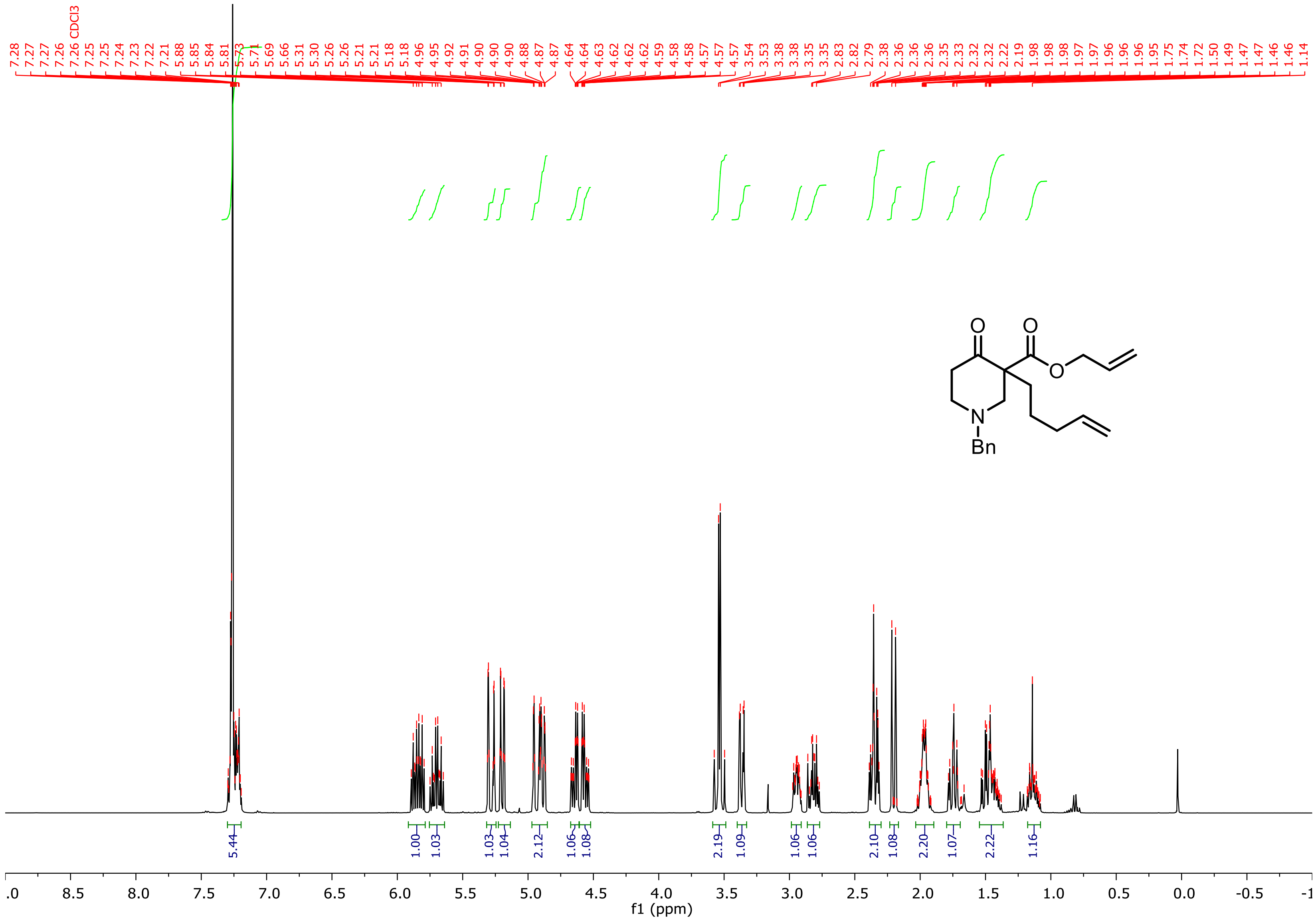

^{13}C NMR Spectrum of S5 (101 MHz, $CDCl_3$)

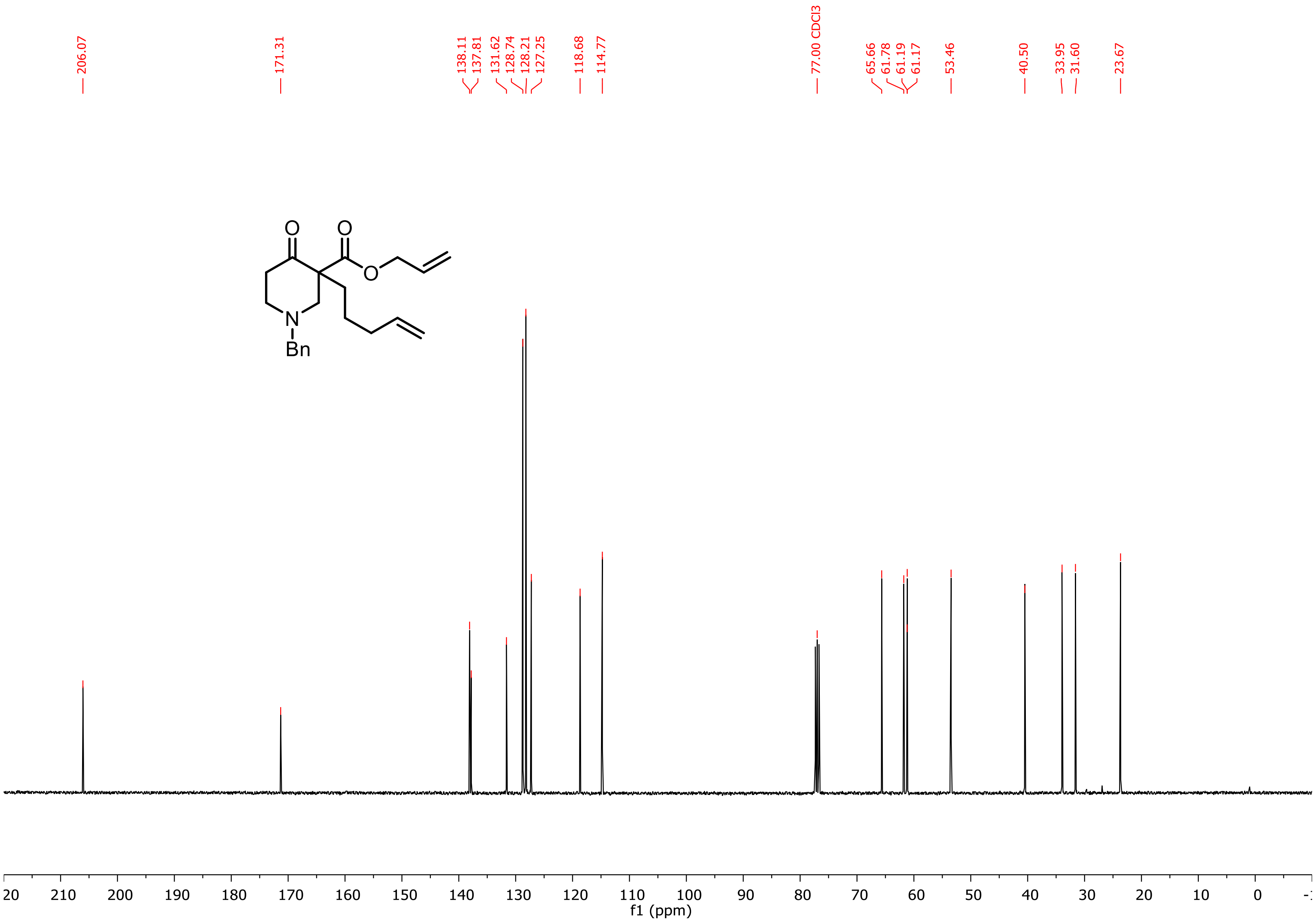

^{1}H NMR Spectrum of S6 (400 MHz, $CDCl_3$)

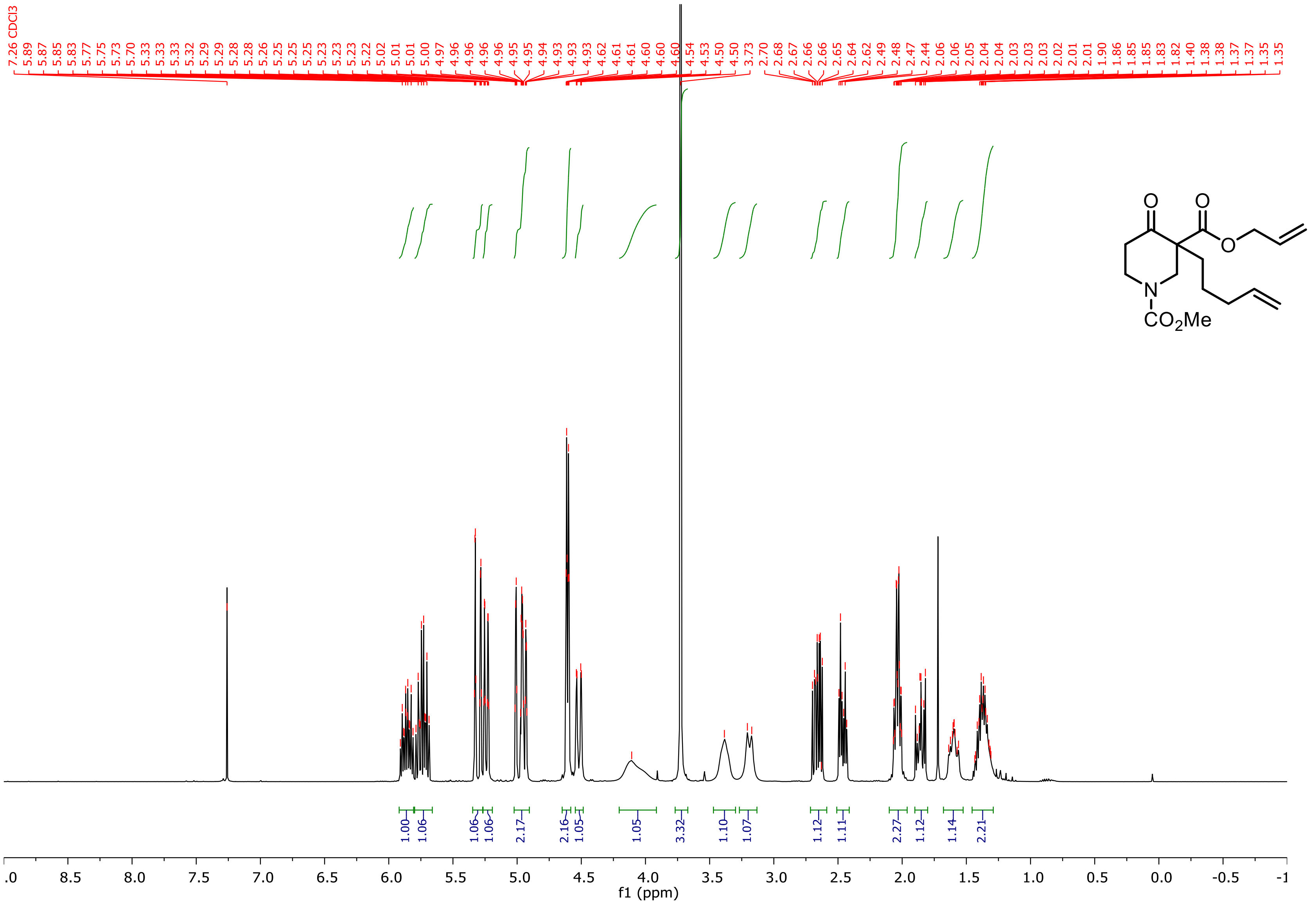

^{13}C NMR Spectrum of S6 (101 MHz, $CDCl_3$)

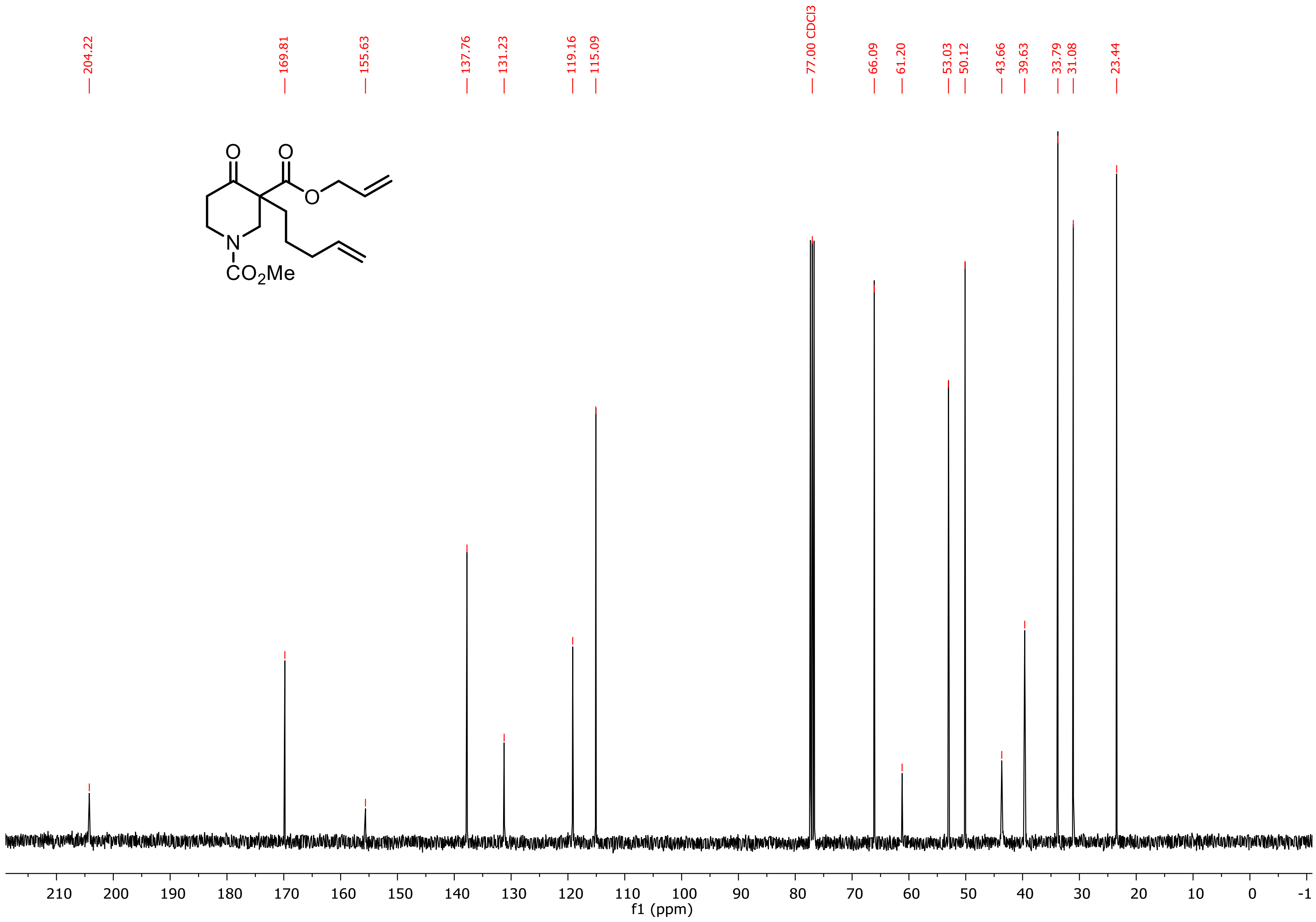

^{1}H NMR Spectrum of 54 (400 MHz, $CDCl_3$)

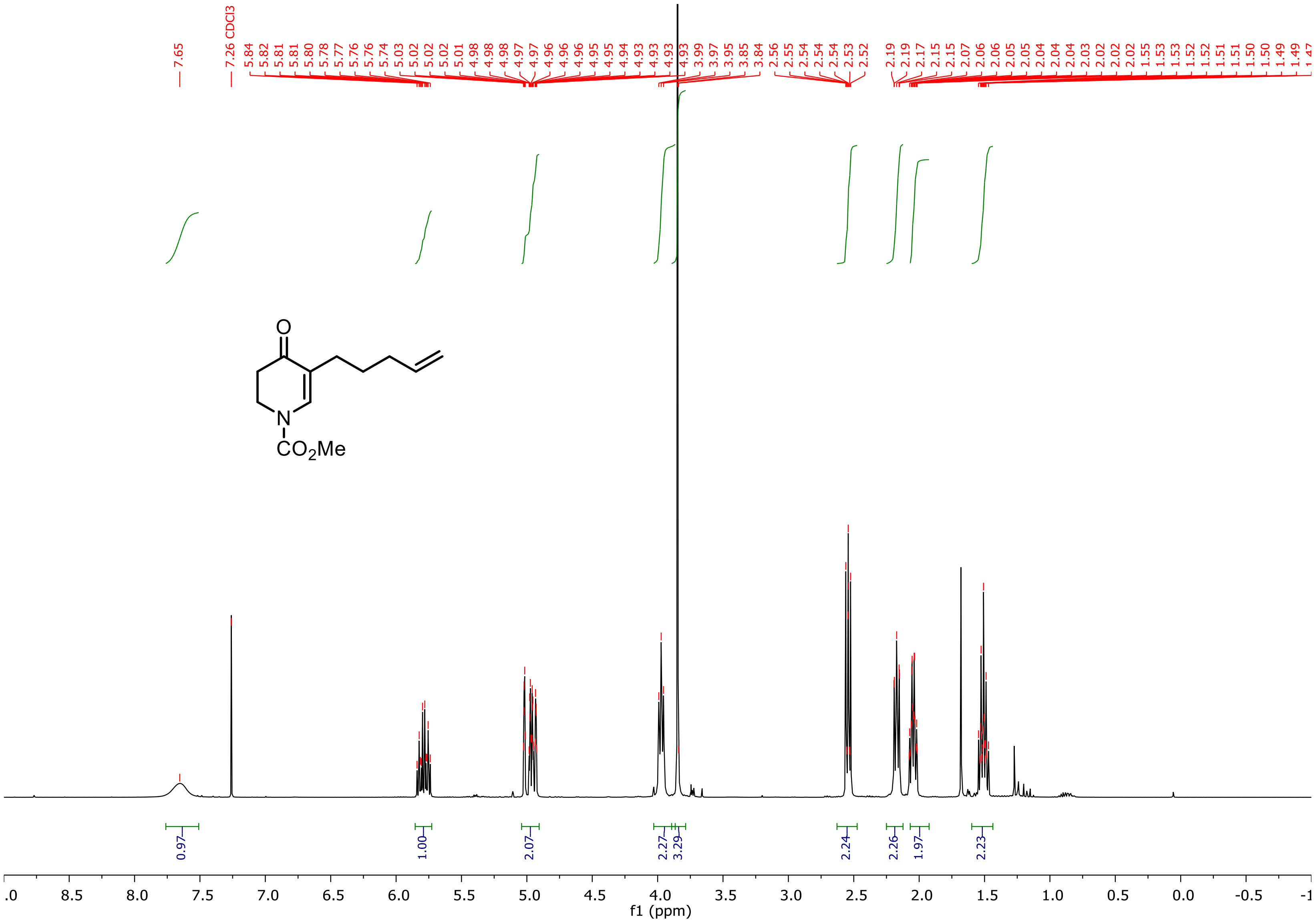

^{13}C NMR Spectrum of 54 (101 MHz, $CDCl_3$)

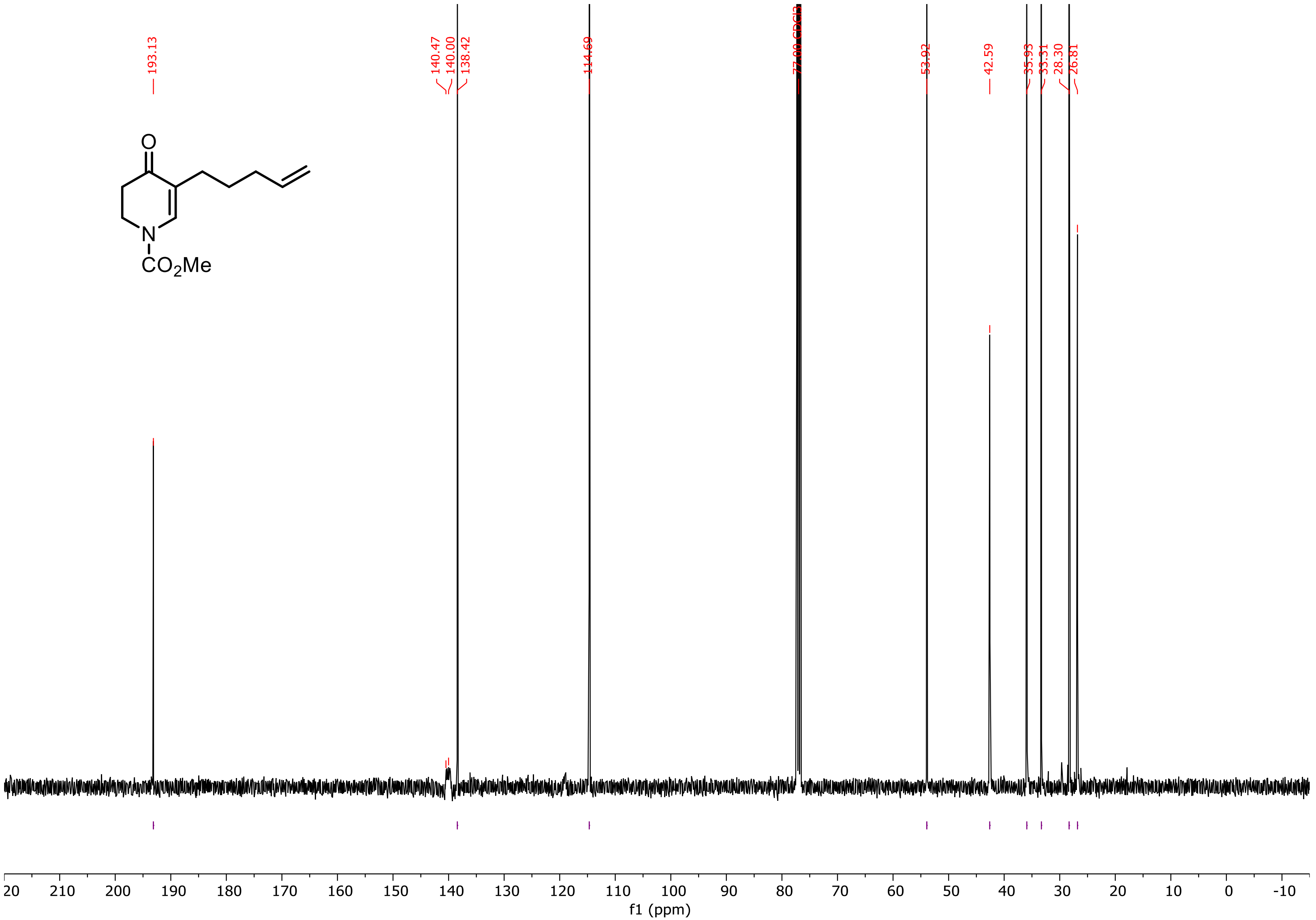

^{1}H NMR Spectrum of 55 (400 MHz, $CDCl_3$)

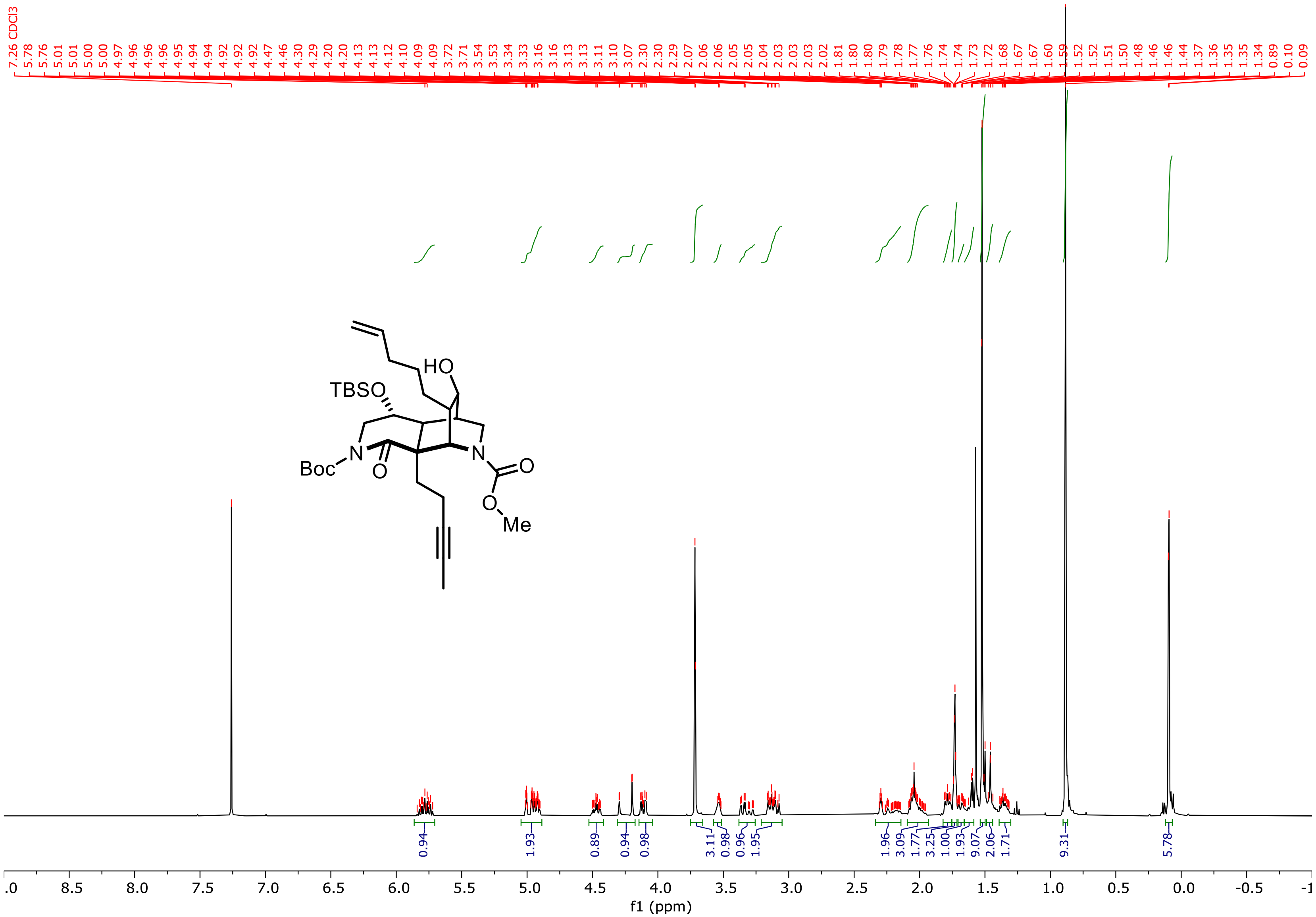

^{13}C NMR Spectrum of 55 (101 MHz, CDCl$_3$)

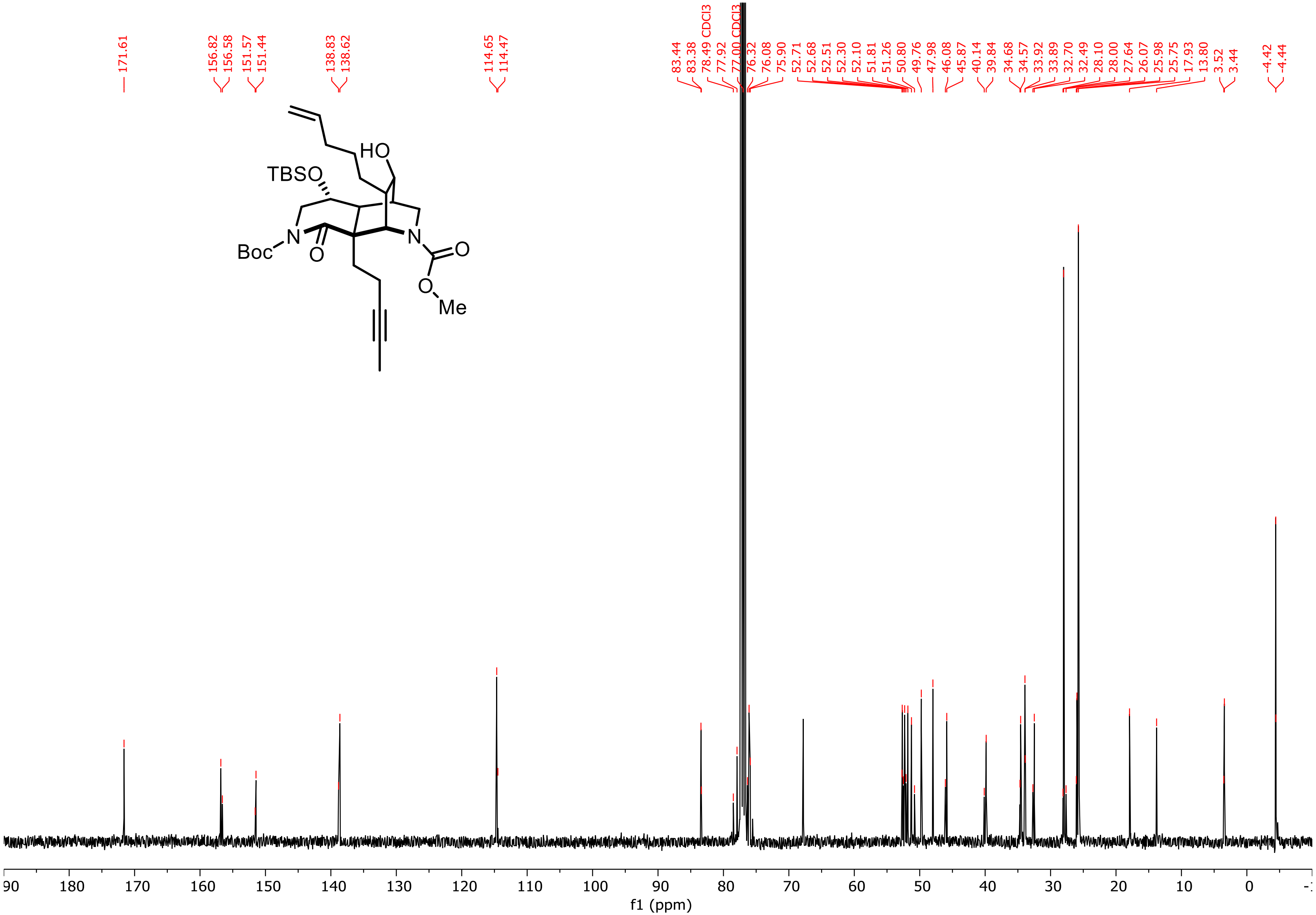

^{1}H NMR Spectrum of S7 (400 MHz, $CDCl_3$)

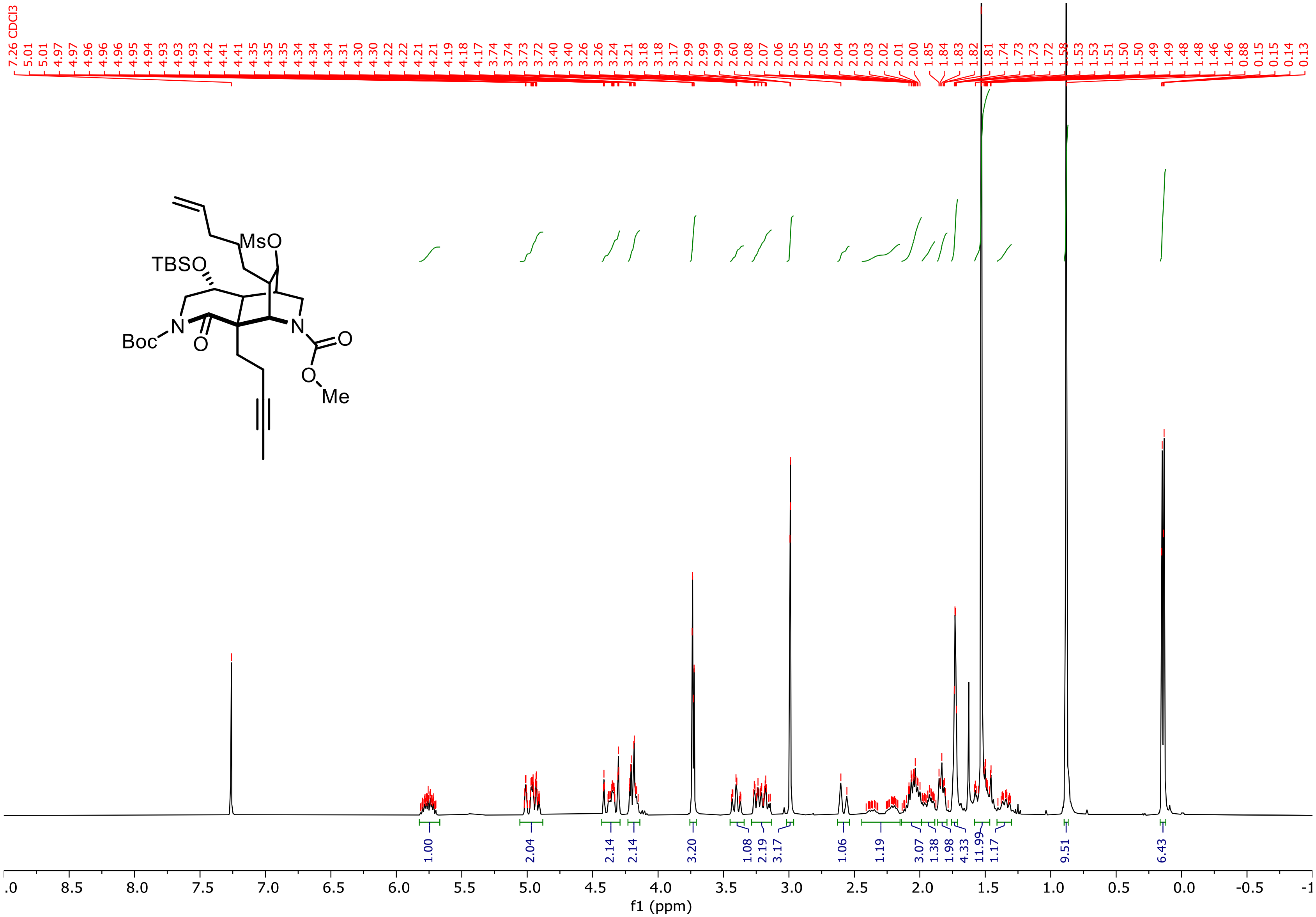

^{13}C NMR Spectrum of S7 (101 MHz, CDCl$_3$)

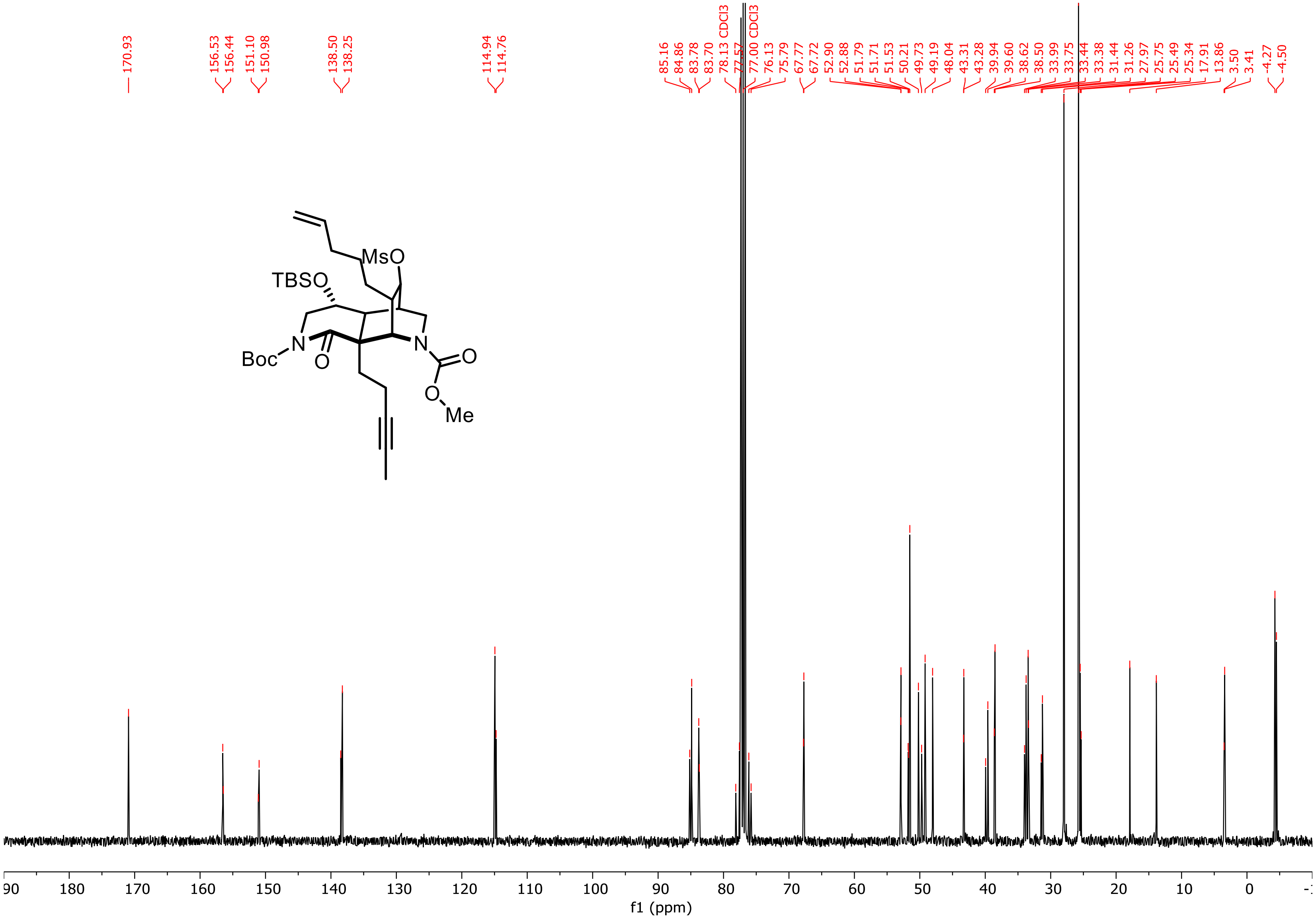

^{1}H NMR Spectrum of S8 (600 MHz, CDCl$_3$)

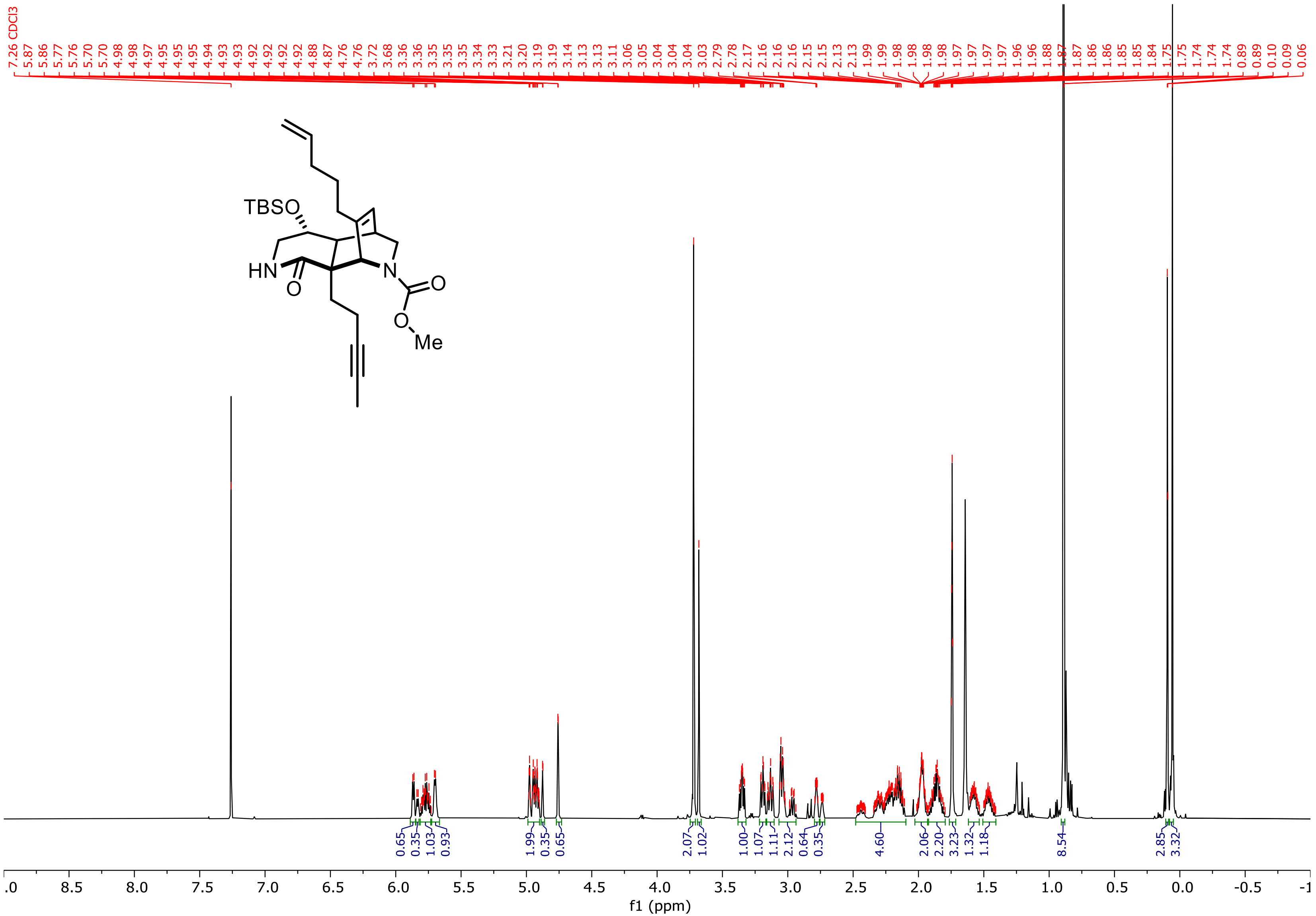

^{13}C NMR Spectrum of S8 (151 MHz, $CDCl_3$)

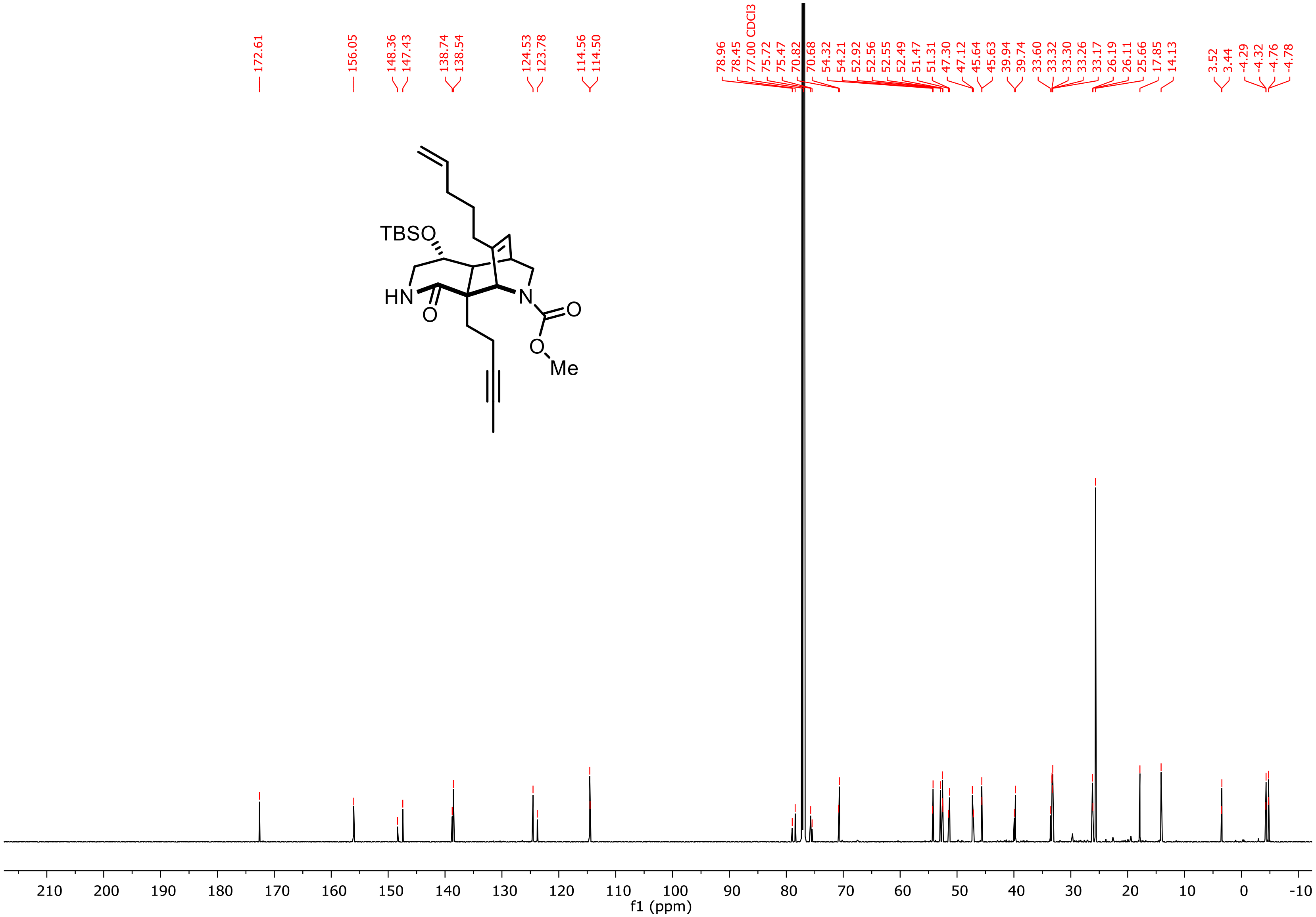

^{1}H NMR Spectrum of S9 (400 MHz, $CDCl_3$)

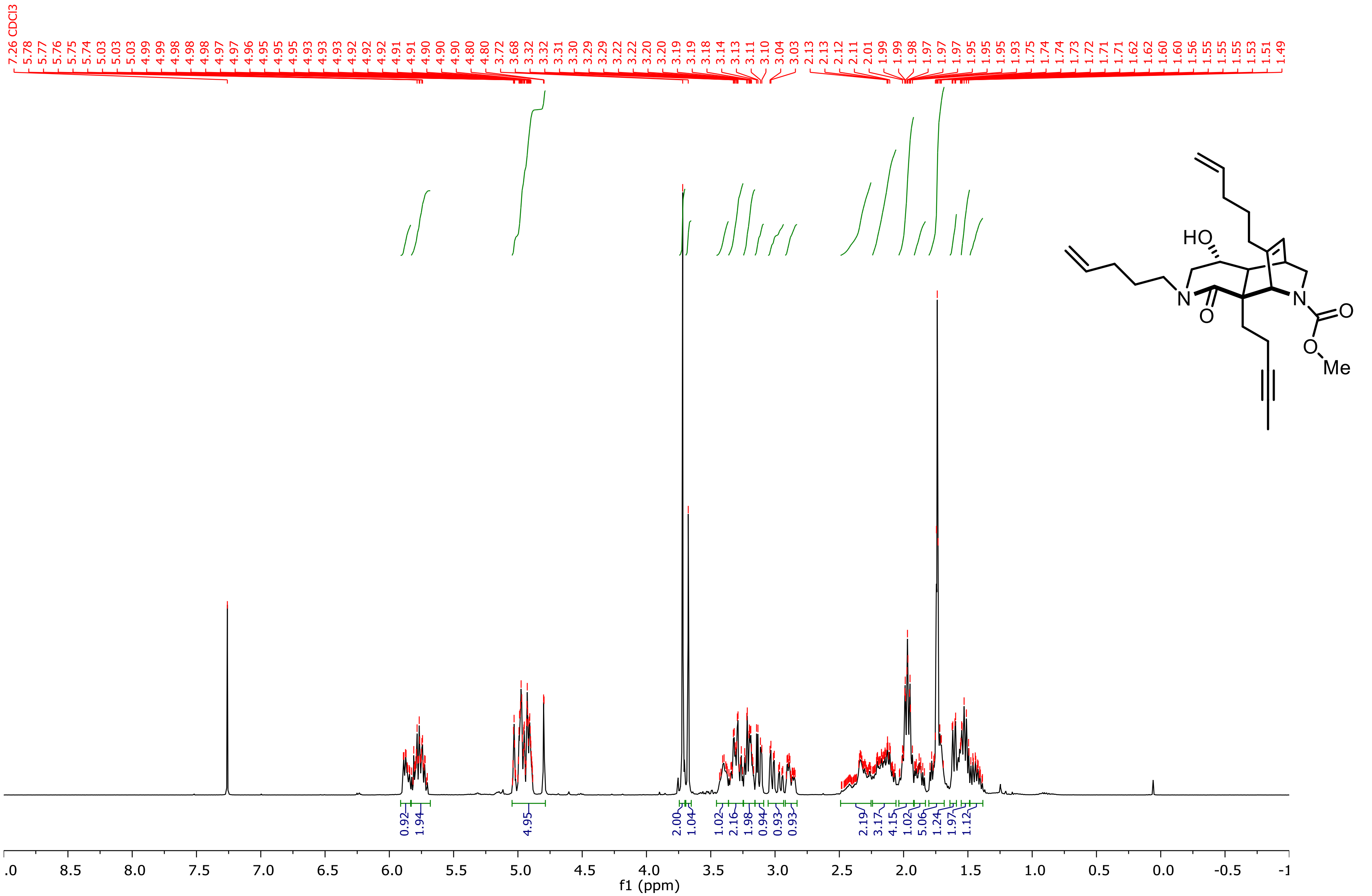

^{13}C NMR Spectrum of S9 (101 MHz, $CDCl_3$)

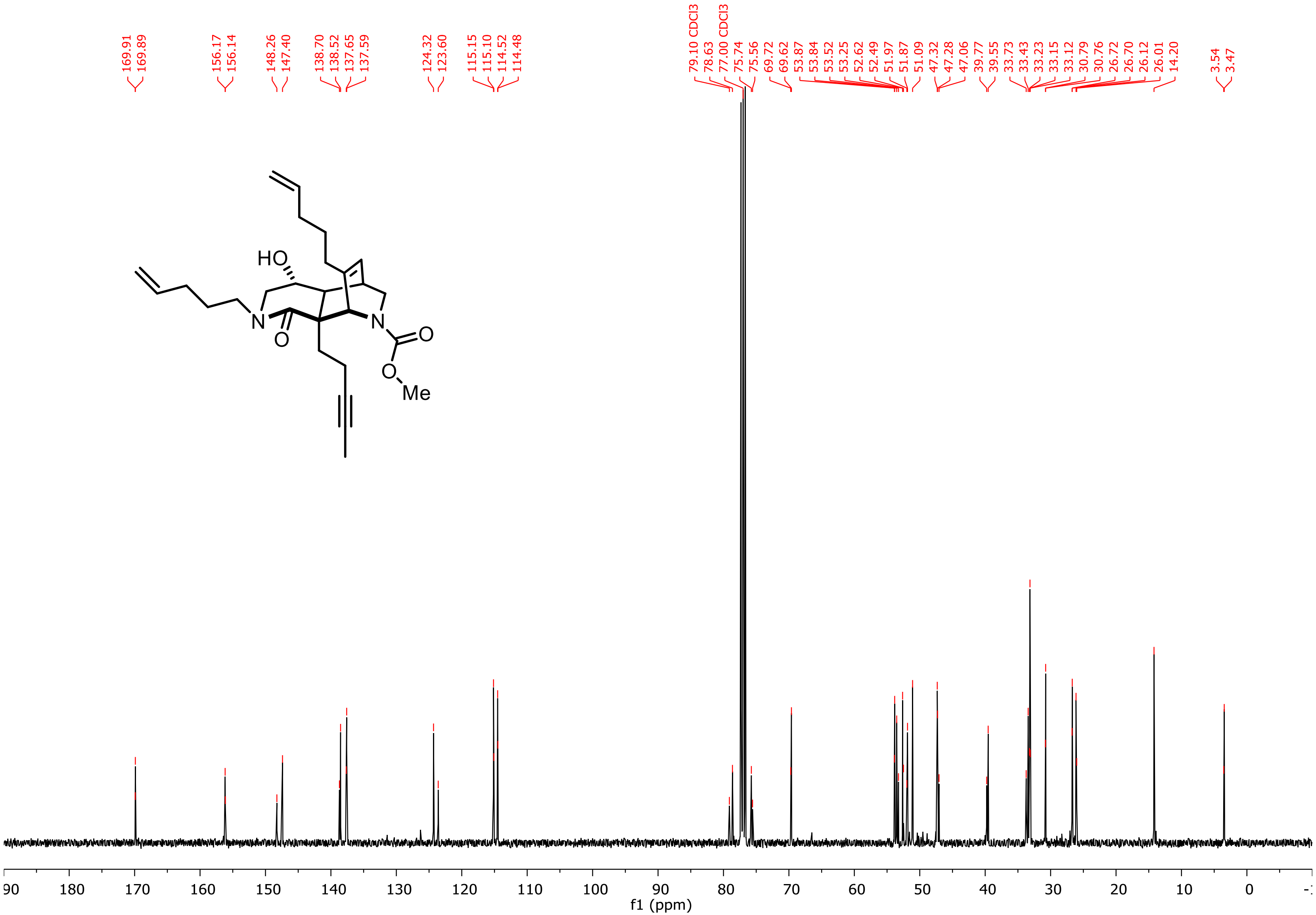

^{1}H NMR Spectrum of S10 (400 MHz, $CDCl_3$)

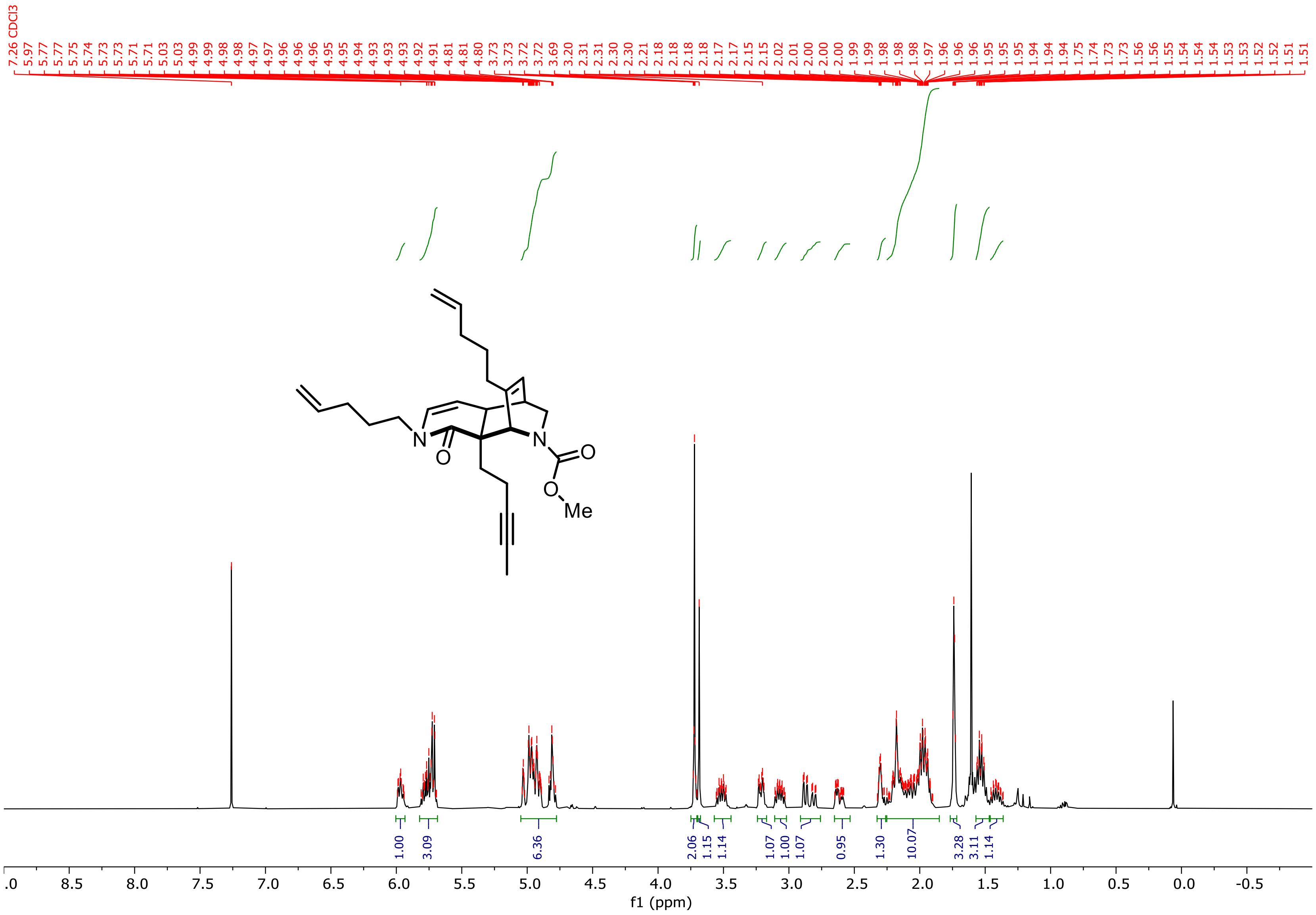

^{13}C NMR Spectrum of S10 (101 MHz, $CDCl_3$)

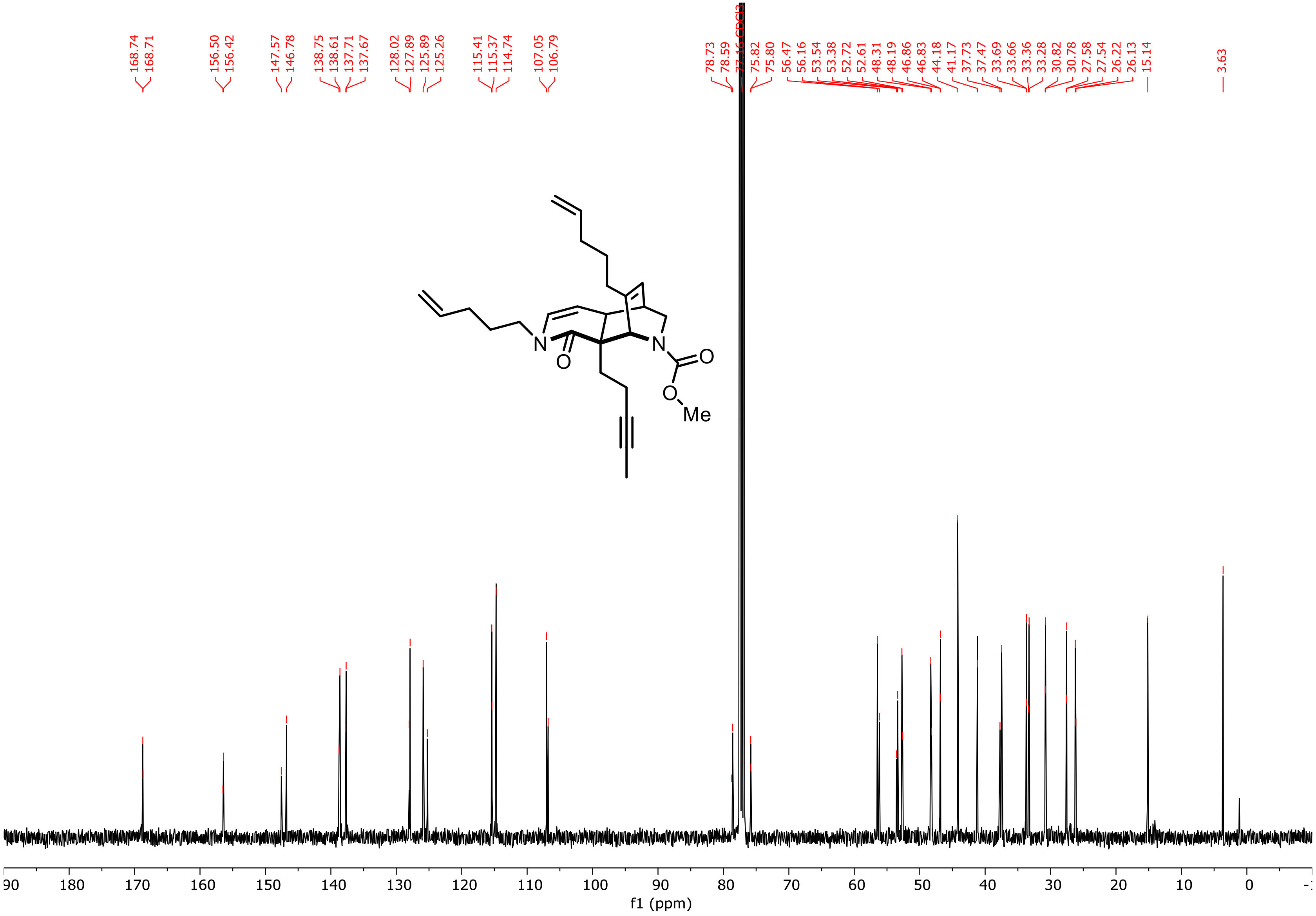

^{1}H NMR Spectrum of 56 (600 MHz, $CDCl_3$)

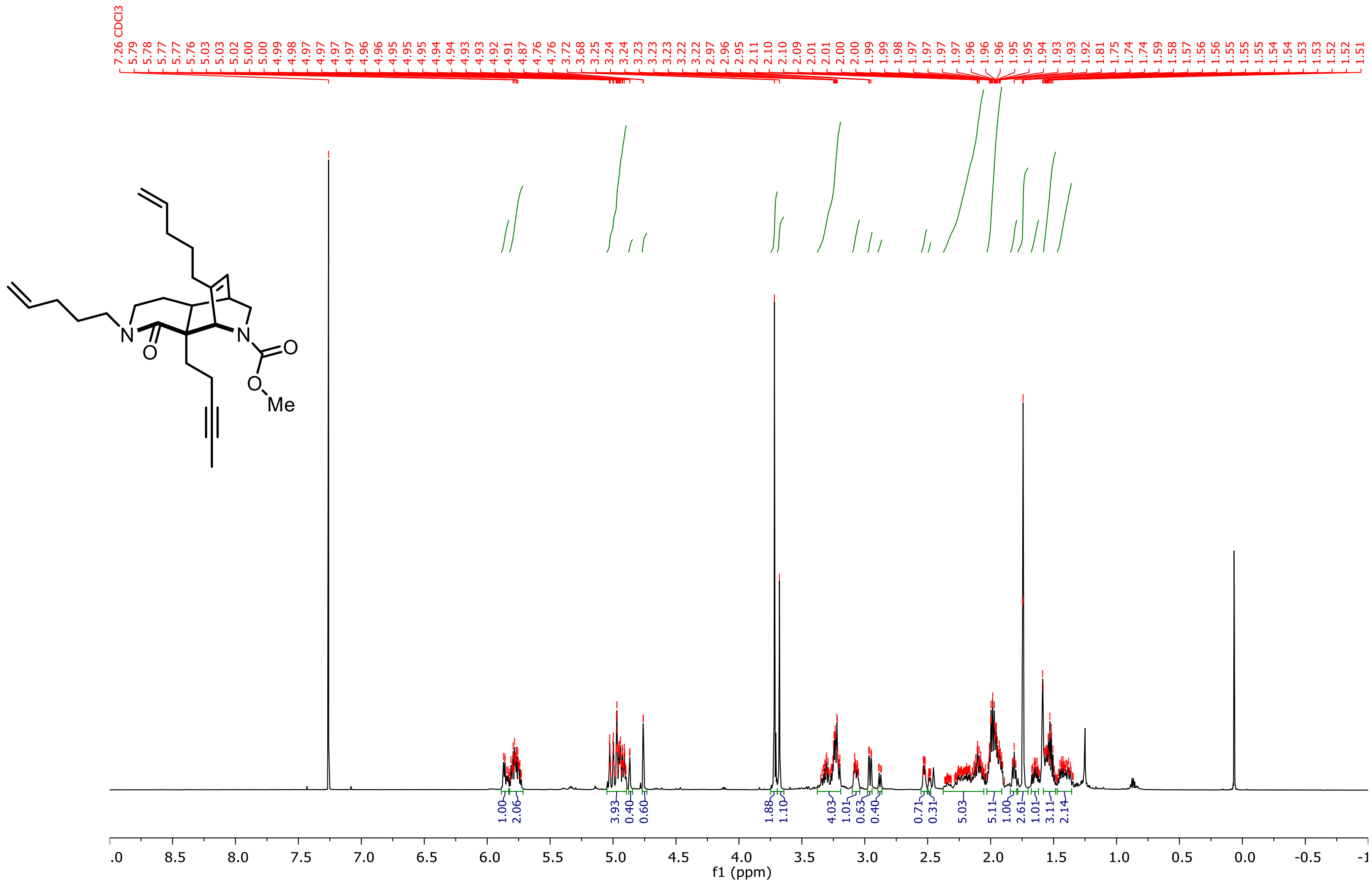

^{13}C NMR Spectrum of 56 (151 MHz, $CDCl_3$)

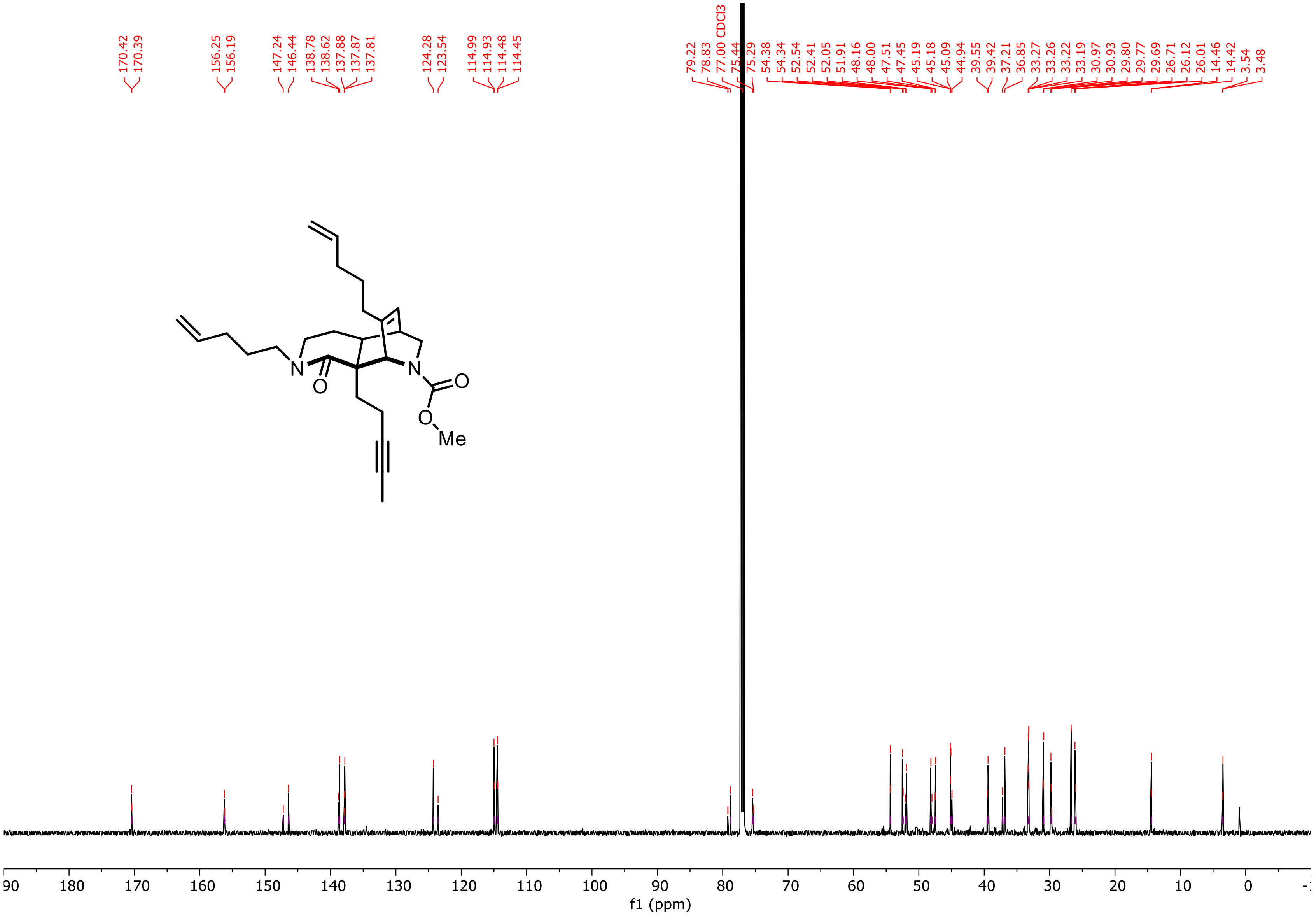

^{1}H NMR Spectrum of 57 (400 MHz, $CDCl_3$)

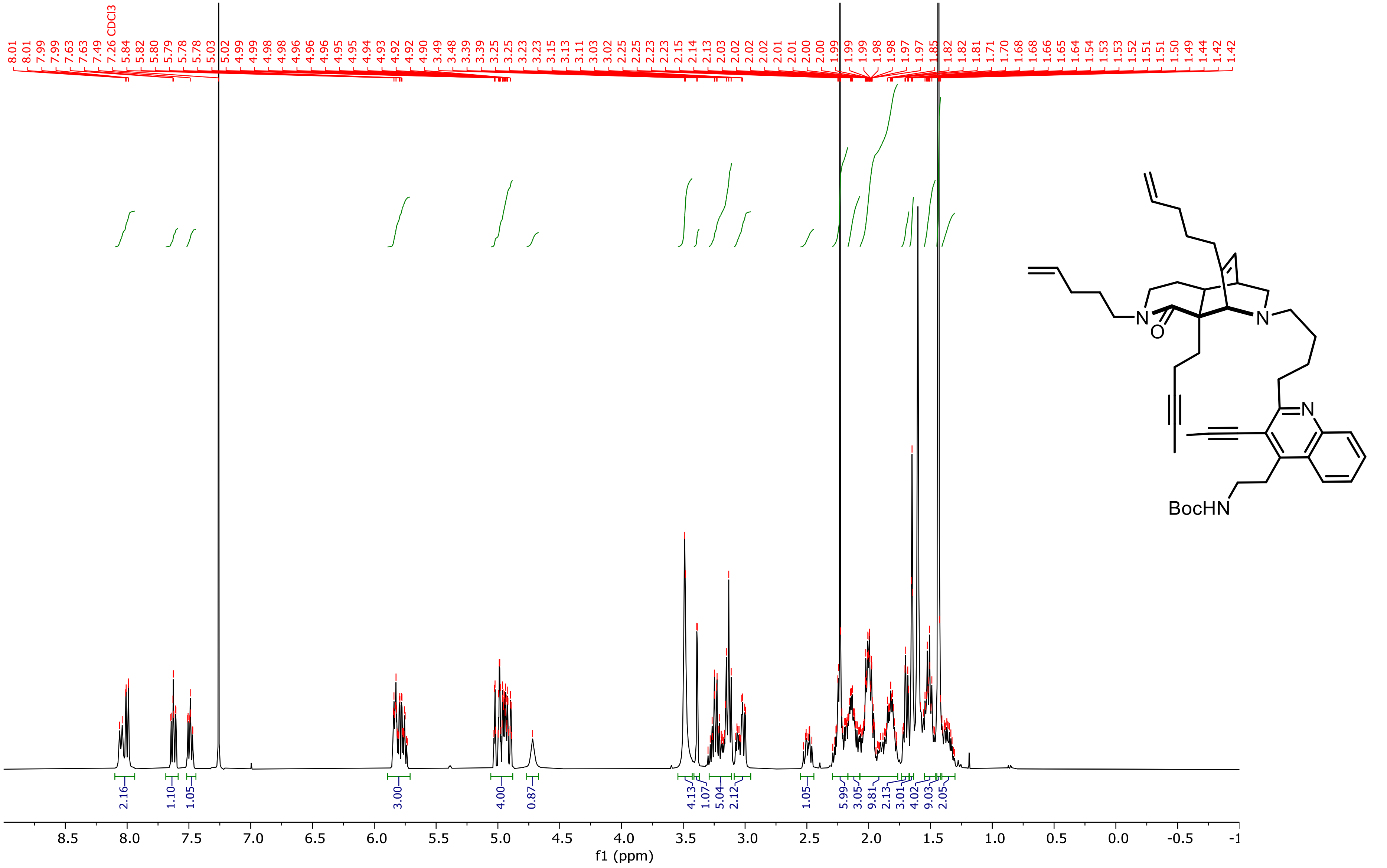

^{13}C NMR Spectrum of 57 (101 MHz, $CDCl_3$)

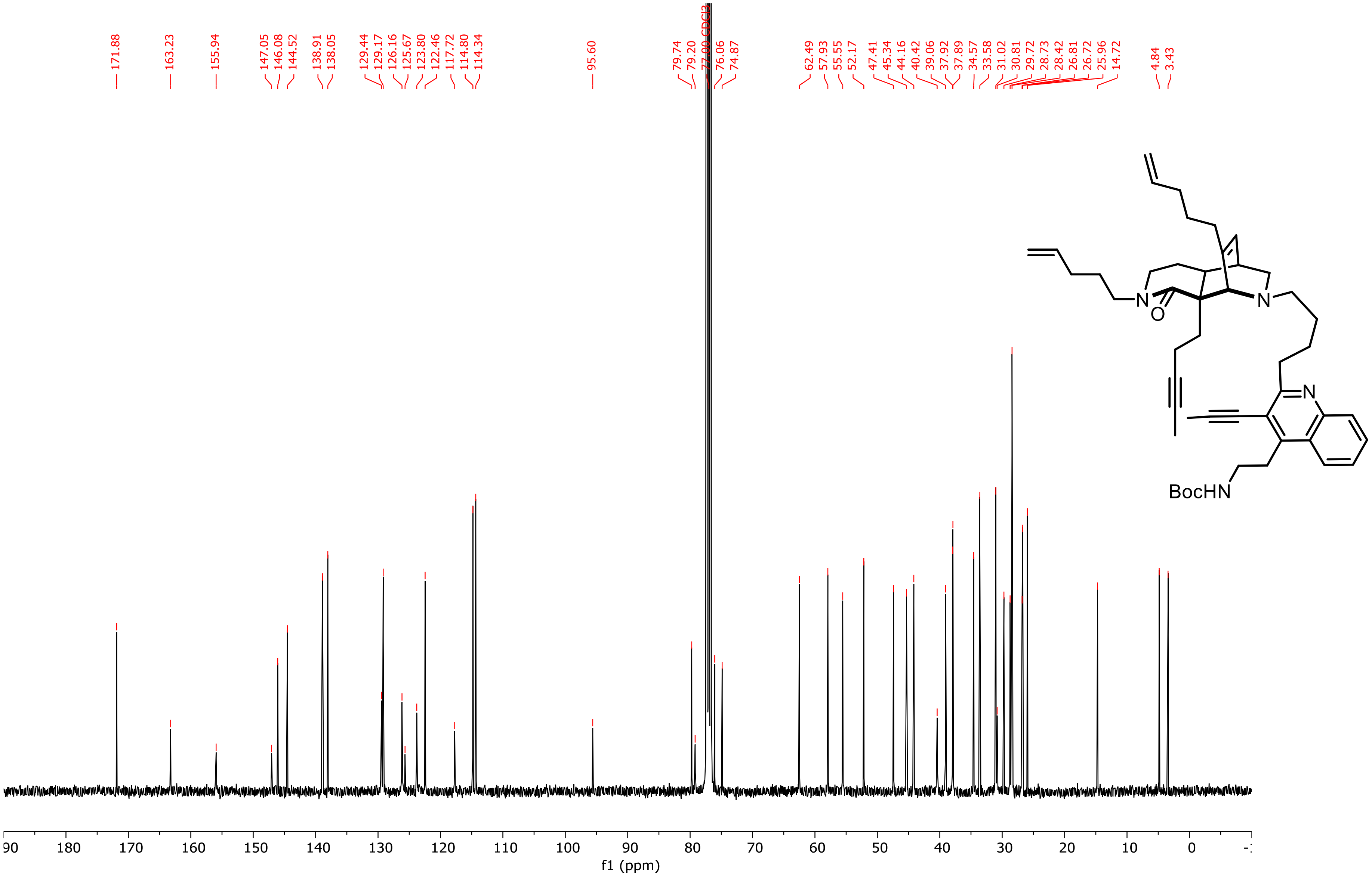

^{1}H NMR Spectrum of 58 (400 MHz, $CDCl_3$)

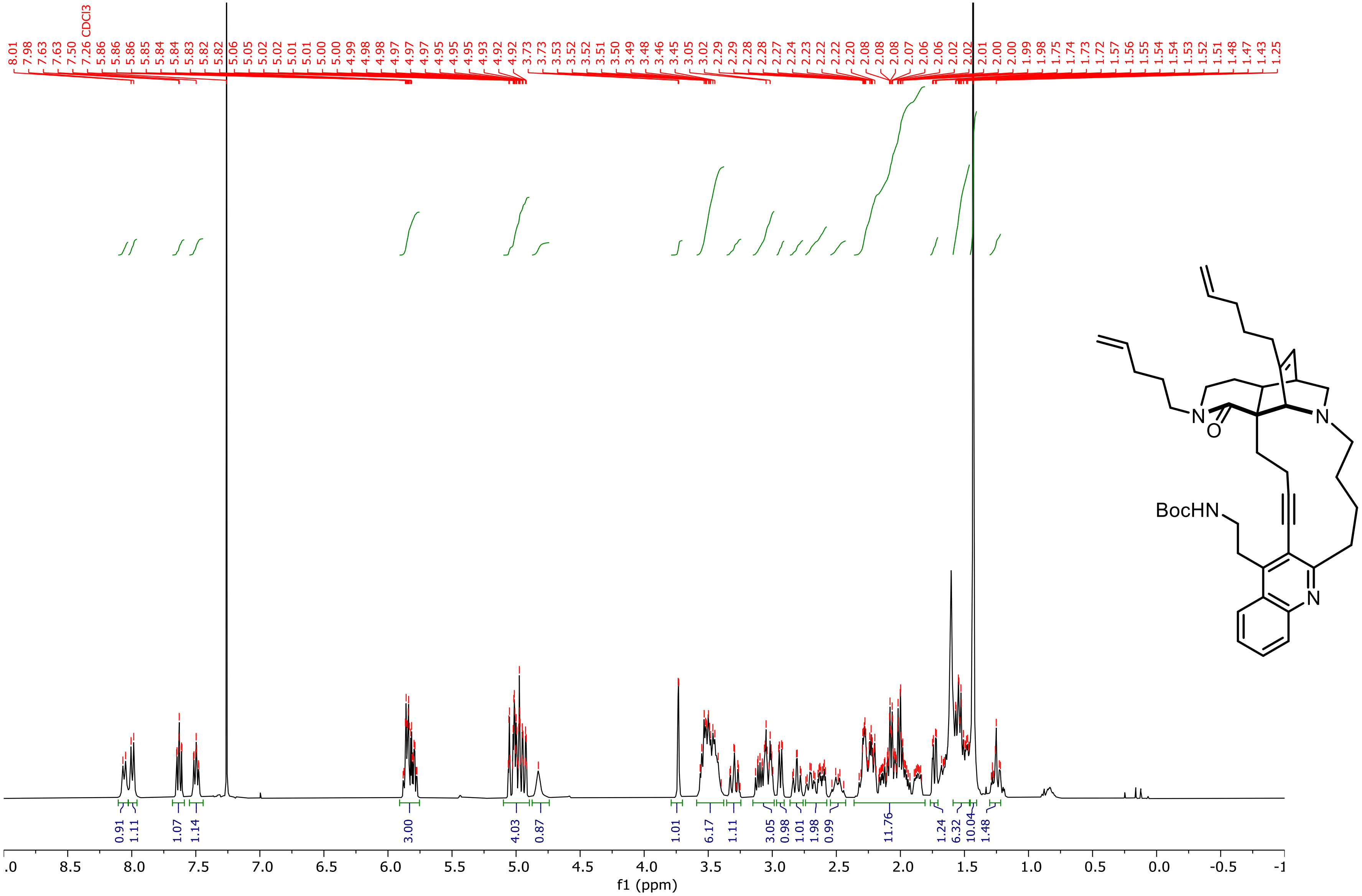

^{13}C NMR Spectrum of 58 (101 MHz, $CDCl_3$)

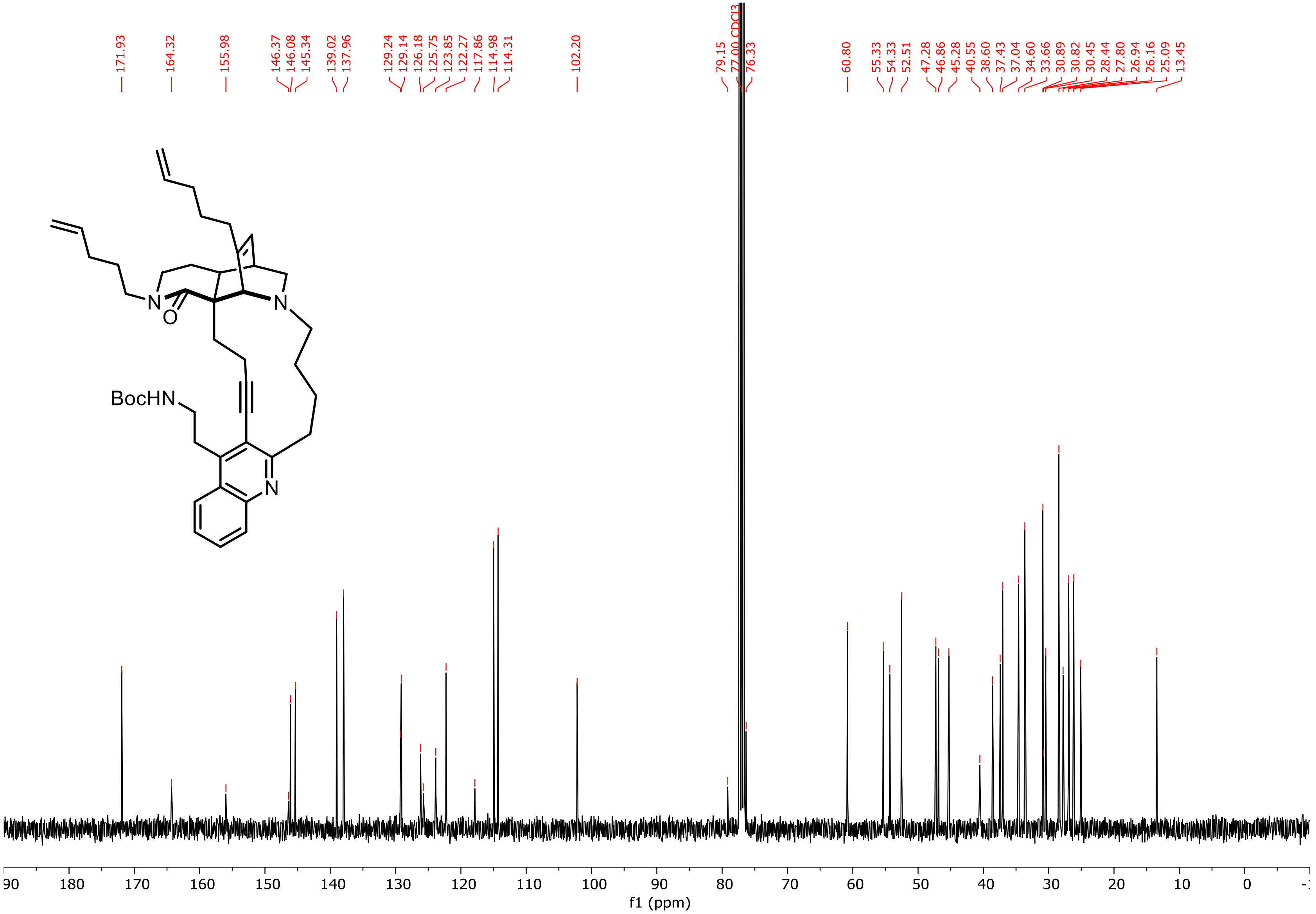

^{1}H NMR Spectrum of S11 (600 MHz, $CDCl_3$)

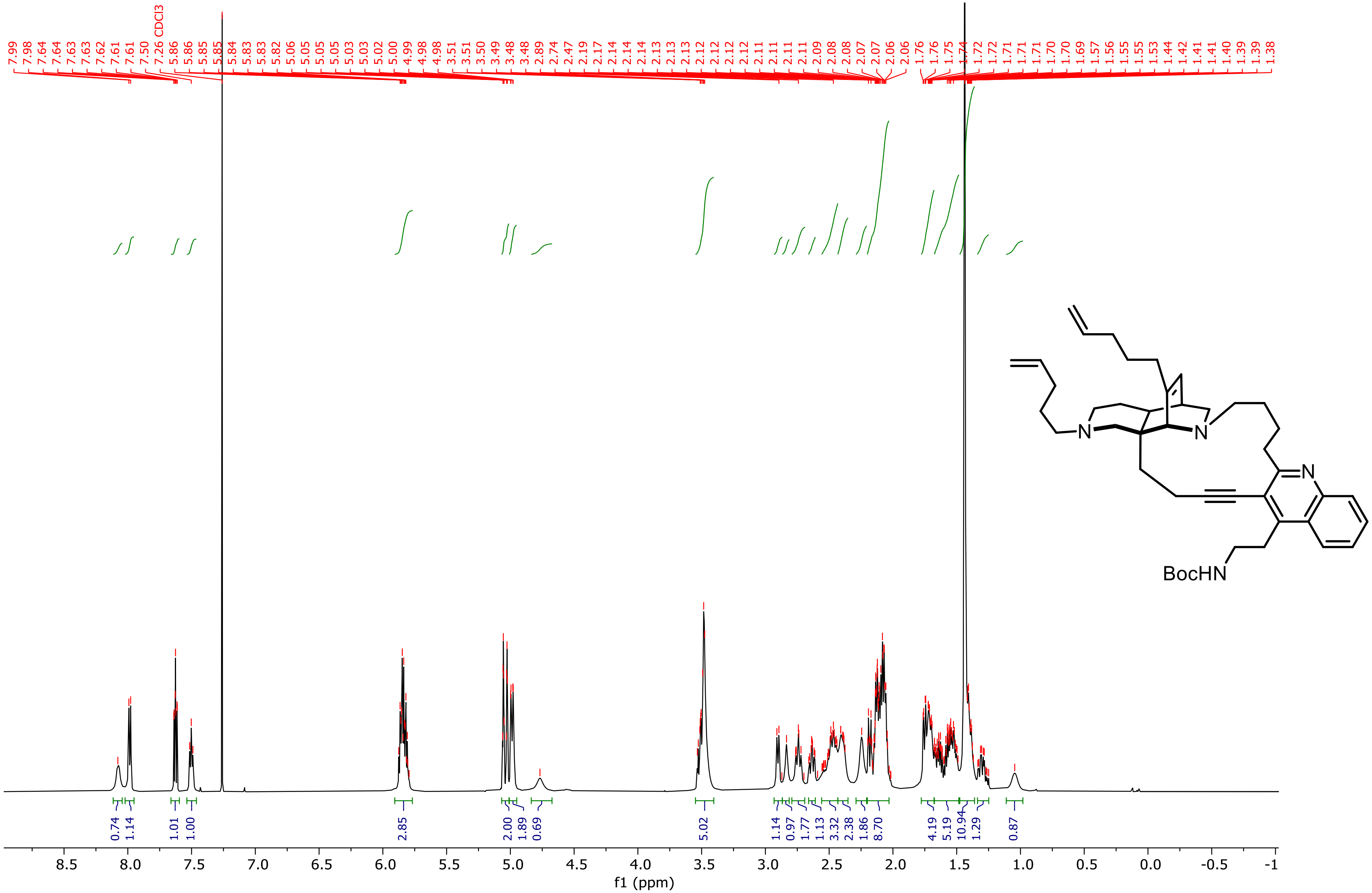

^{13}C NMR Spectrum of S11 (151 MHz, CDCl$_3$)

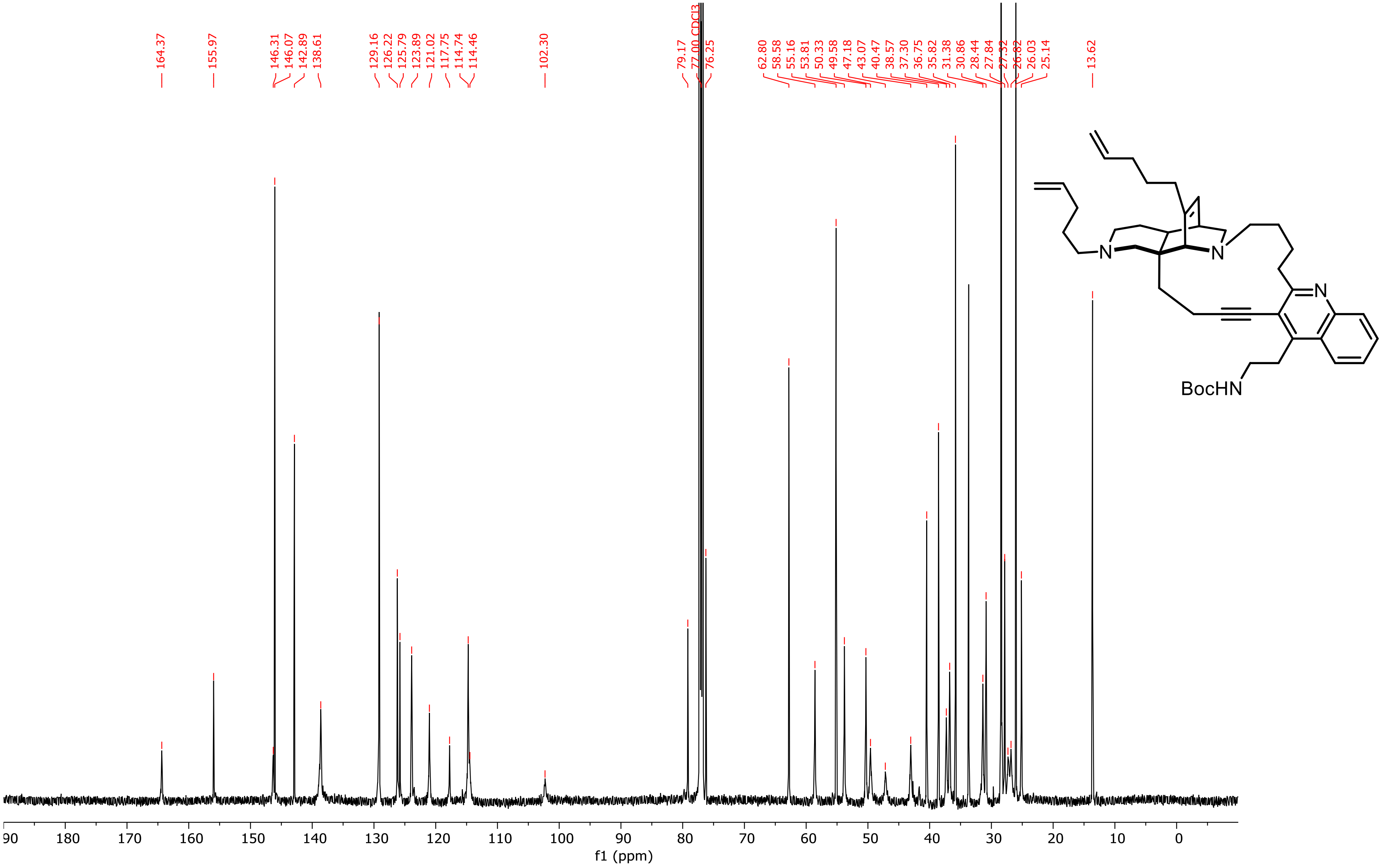

^{1}H NMR Spectrum of 61 (400 MHz, $CDCl_3$)

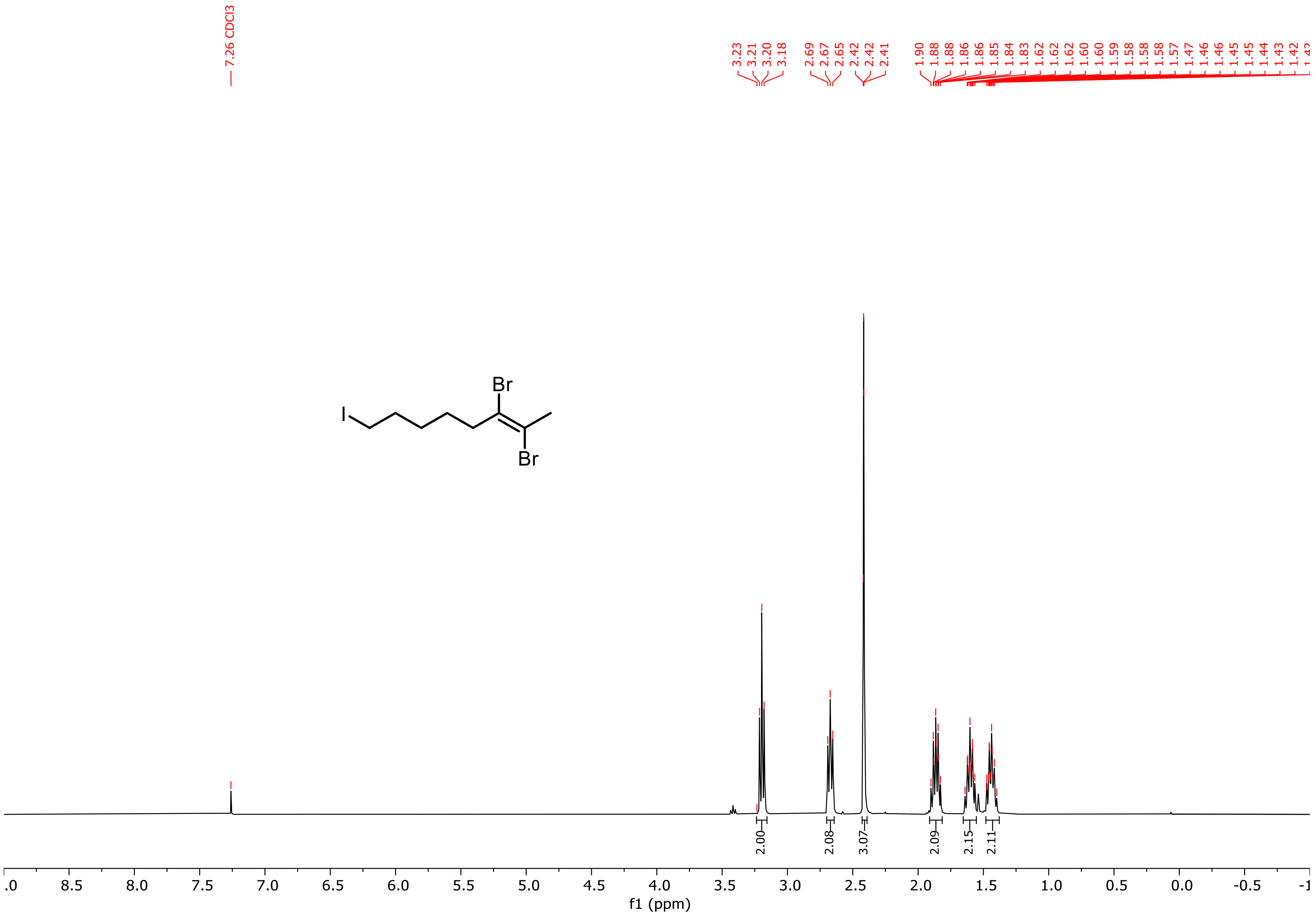

^{13}C NMR Spectrum of 61 (101 MHz, $CDCl_3$)

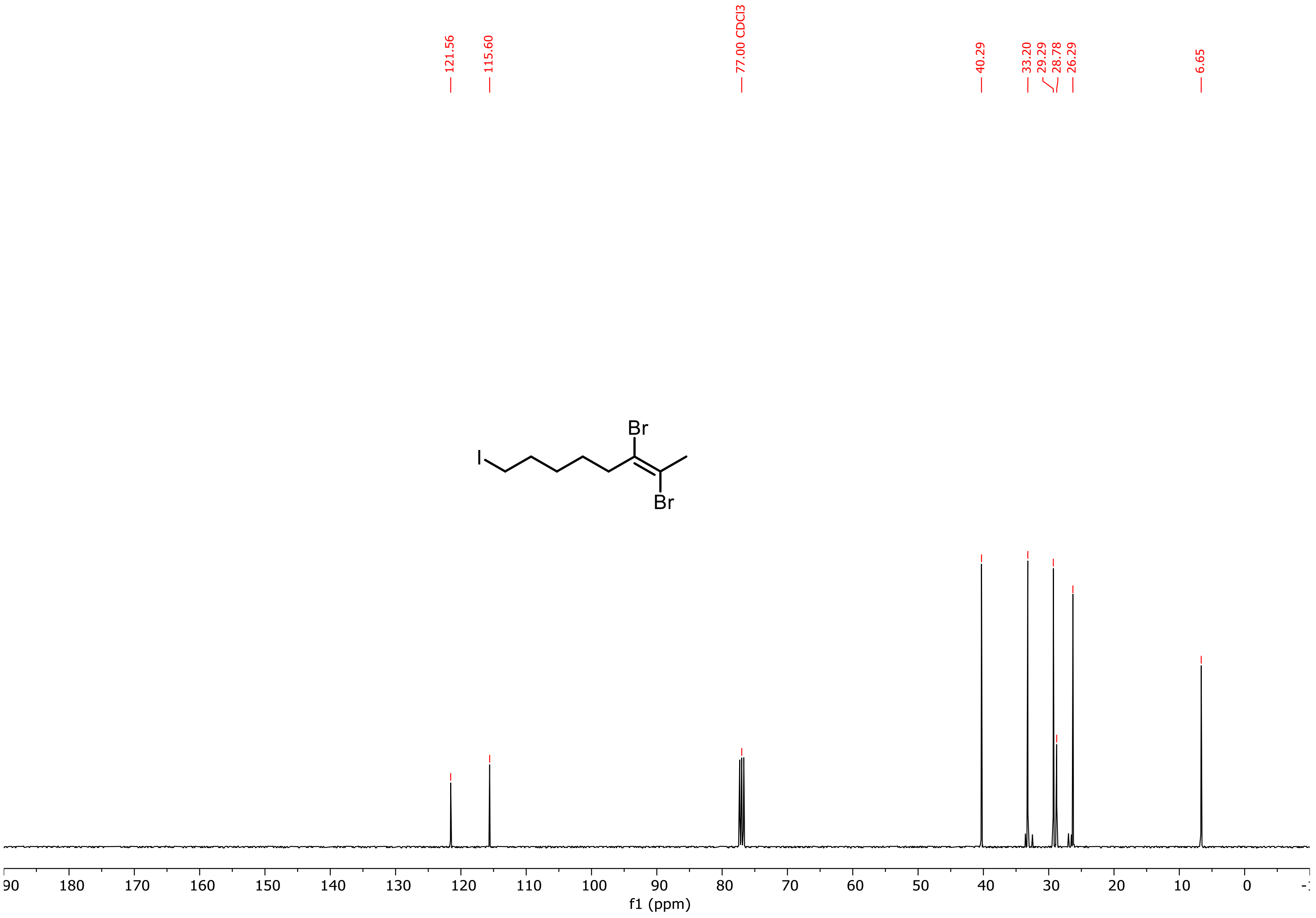

^{1}H NMR Spectrum of S12 (400 MHz, $CDCl_3$)

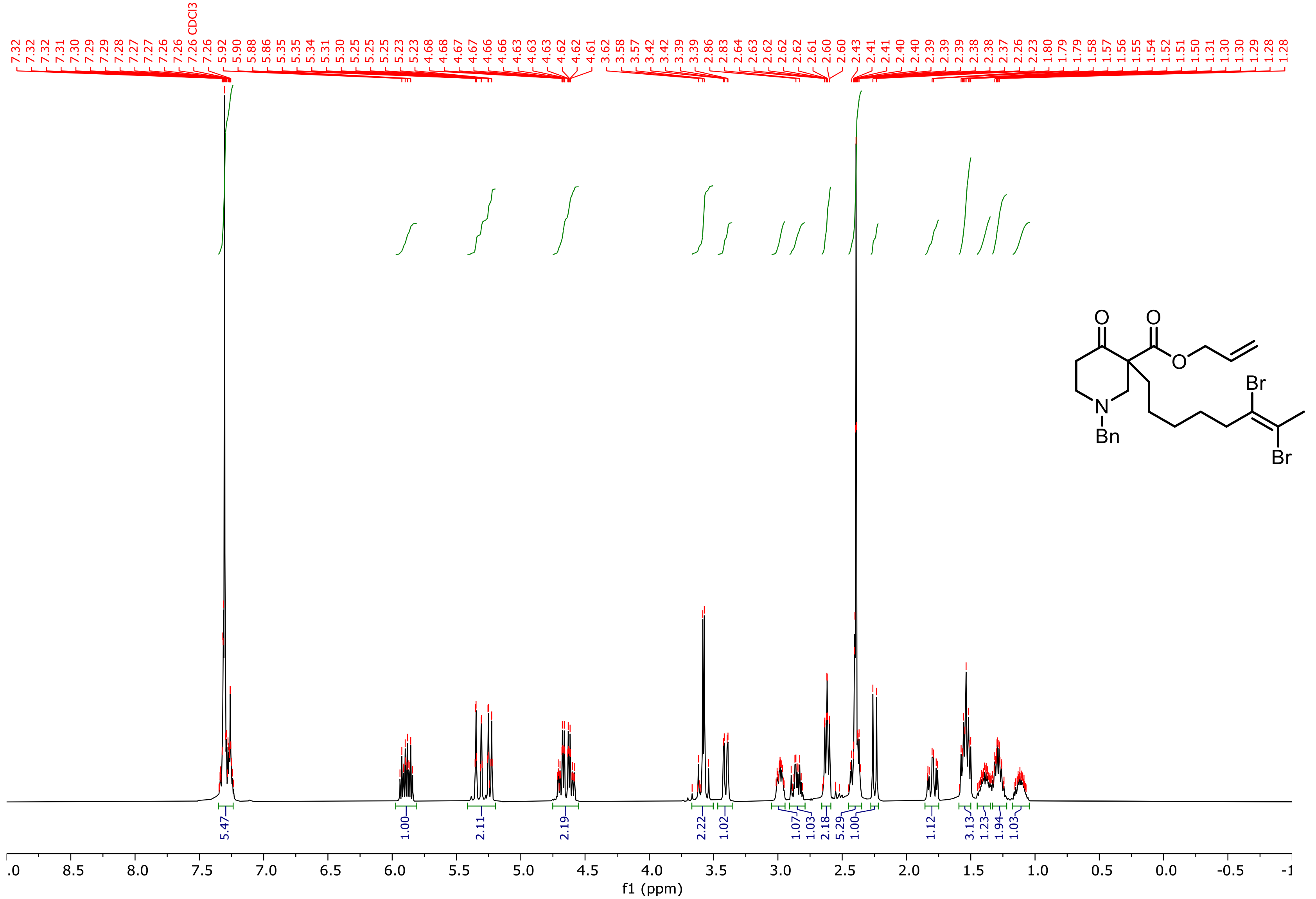

^{13}C NMR Spectrum of S12 (101 MHz, $CDCl_3$)

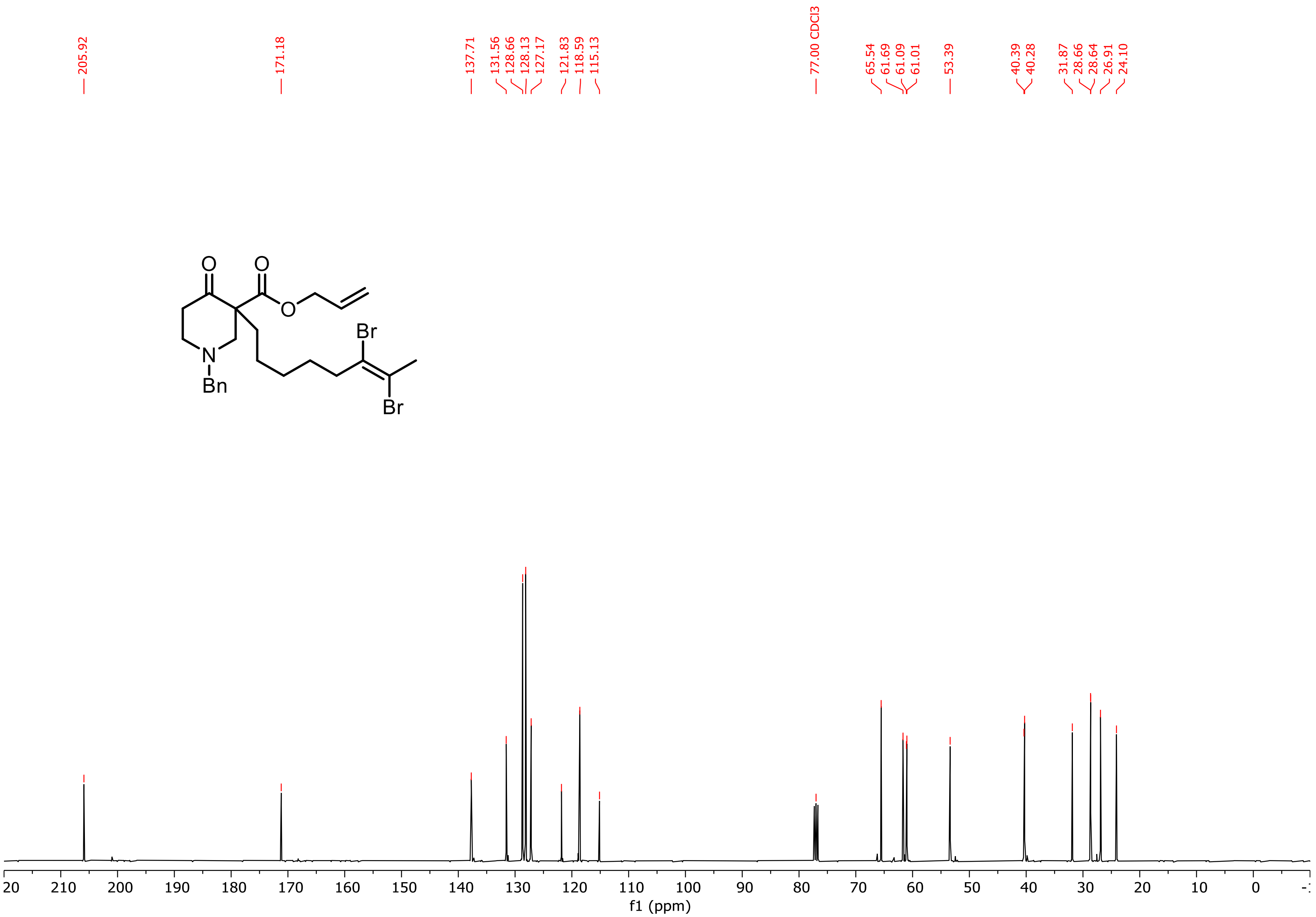

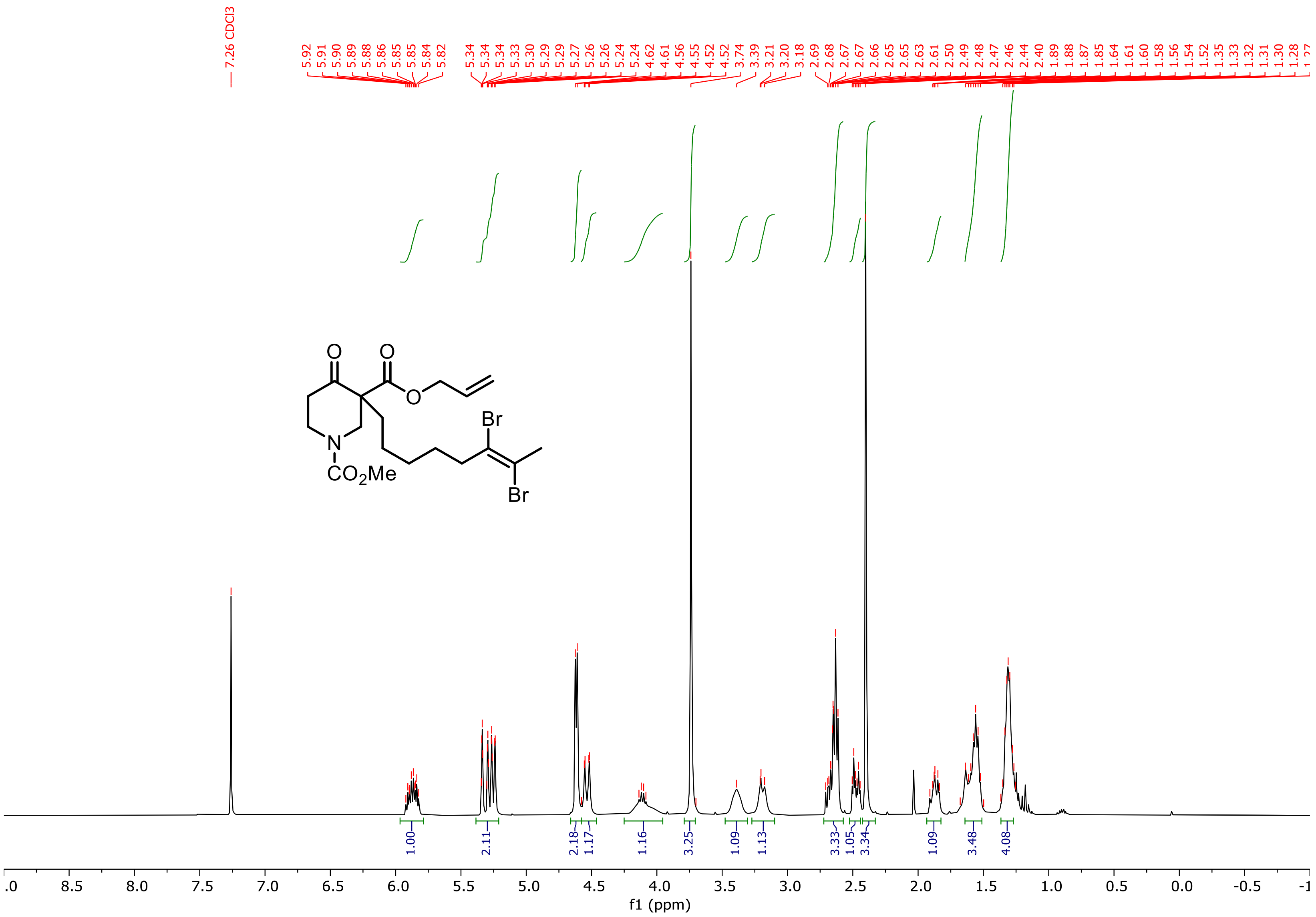

CO2Me
Br
Br
f1 (ppm)

^{13}C NMR Spectrum of 62 (101 MHz, $CDCl_3$)

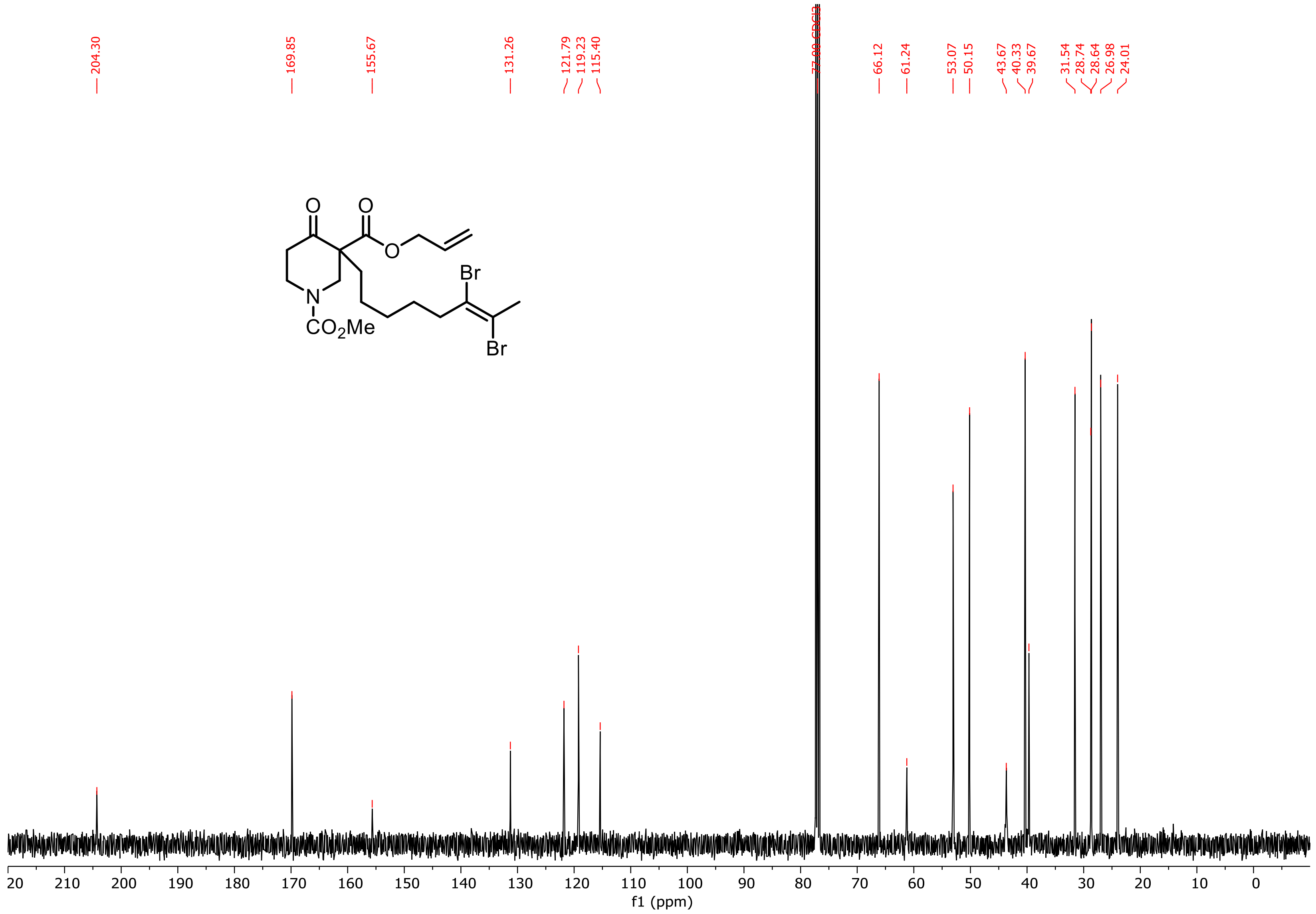

^{1}H NMR Spectrum of 63 (400 MHz, $CDCl_3$)

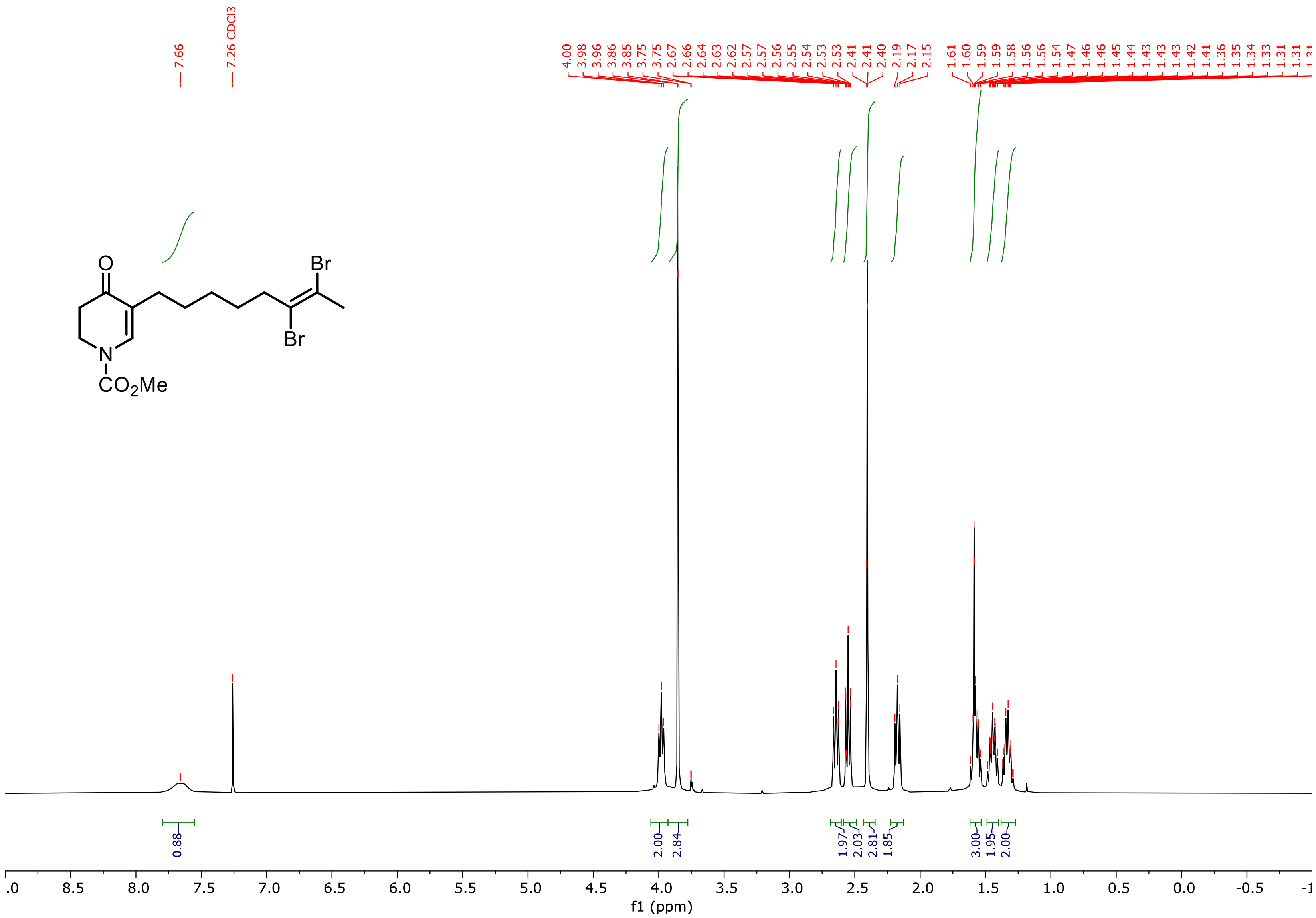

^{13}C NMR Spectrum of 63 (101 MHz, $CDCl_3$)

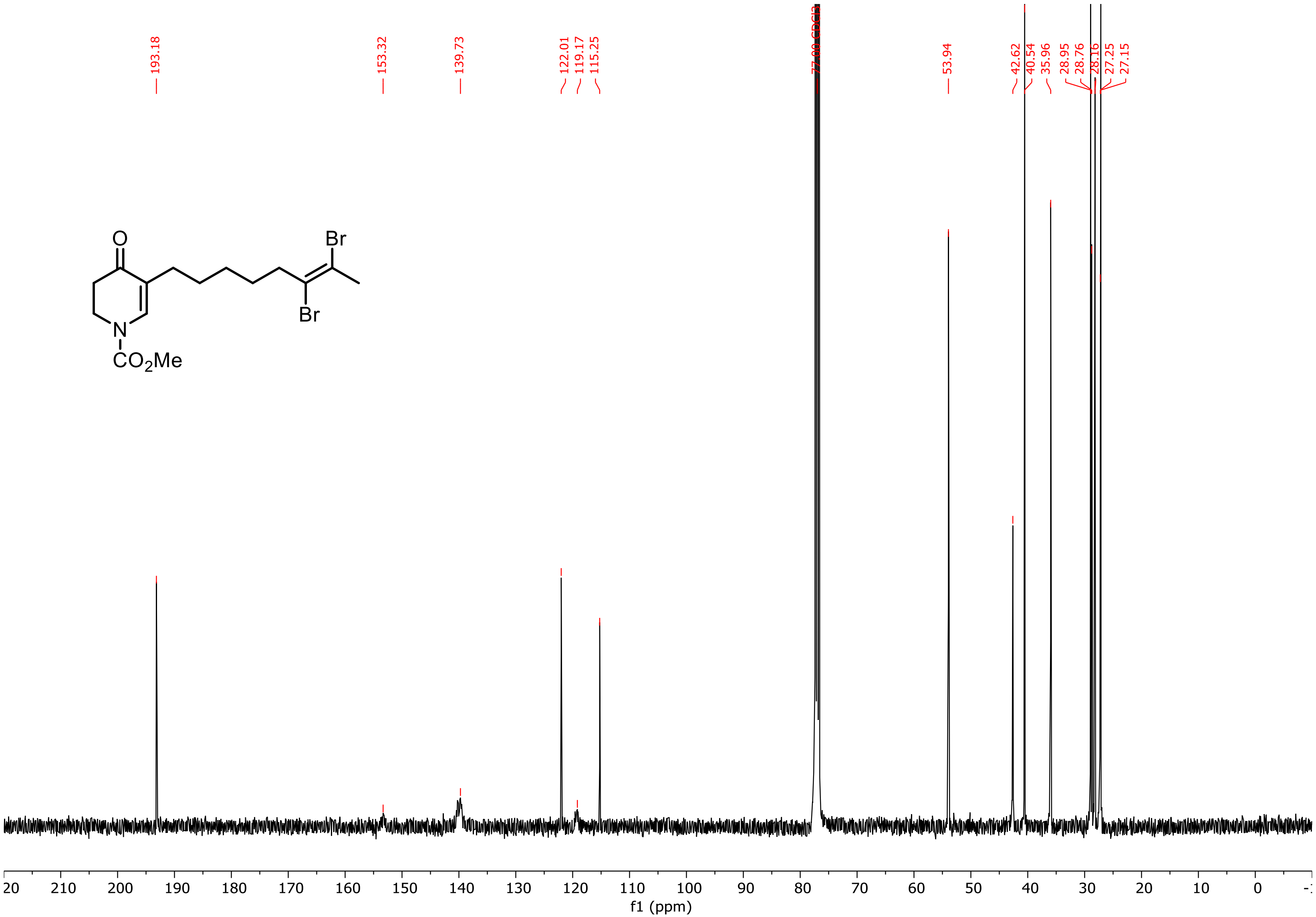

^{1}H NMR Spectrum of 64 (400 MHz, CDCl$_3$)

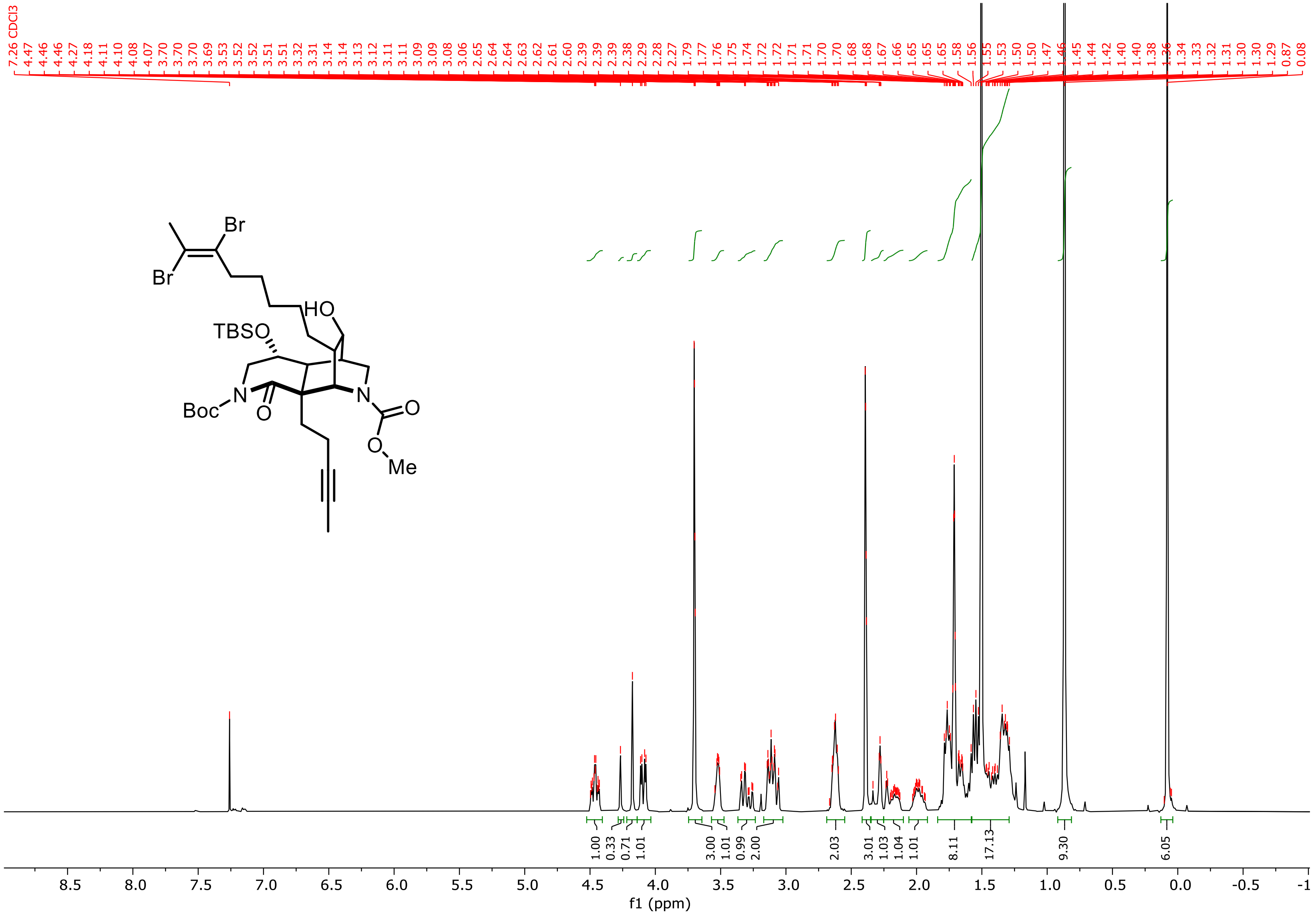

^{13}C NMR Spectrum of 64 (101 MHz, CDCl$_3$)

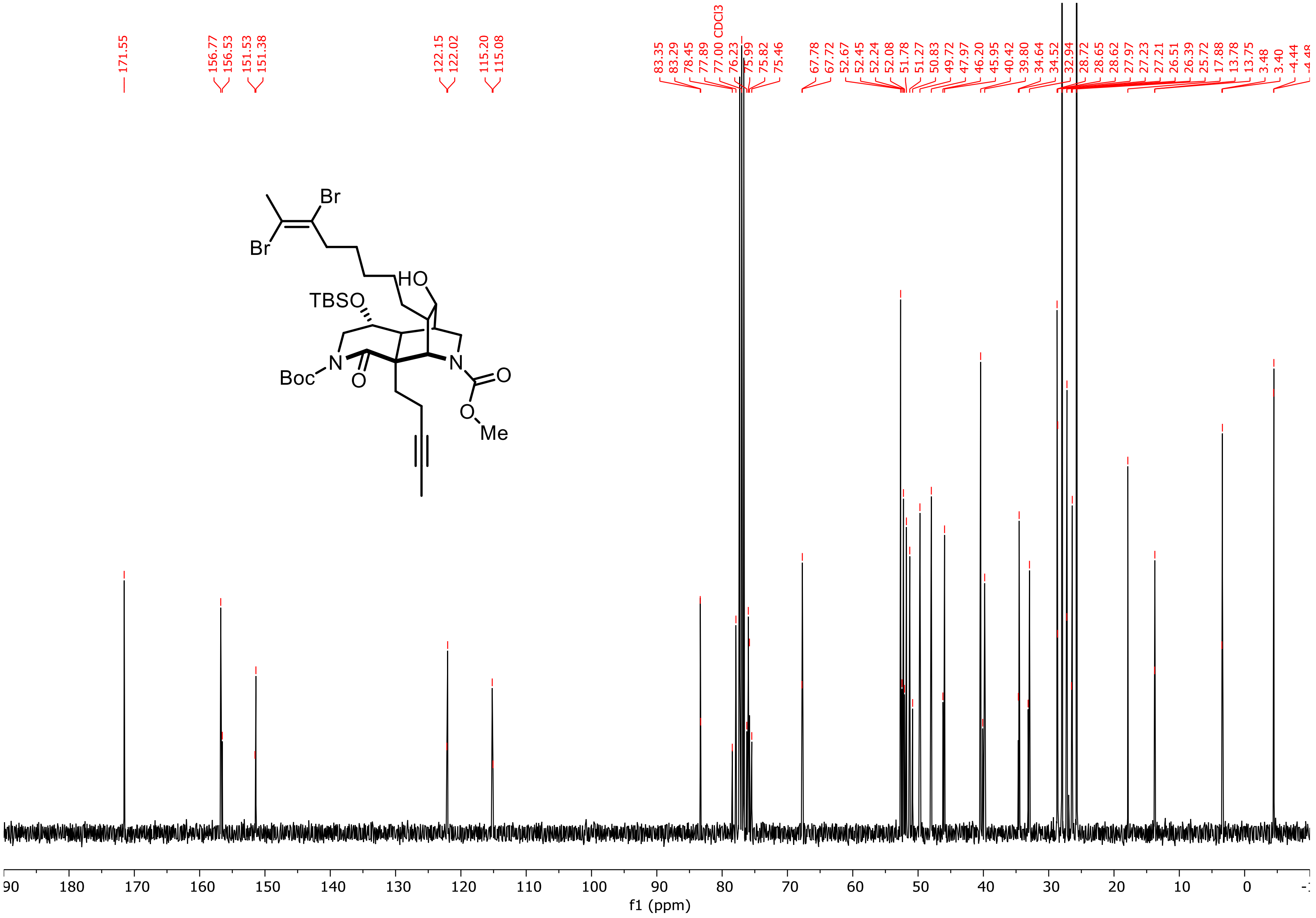

^{1}H NMR Spectrum of S13 (400 MHz, $CDCl_3$)

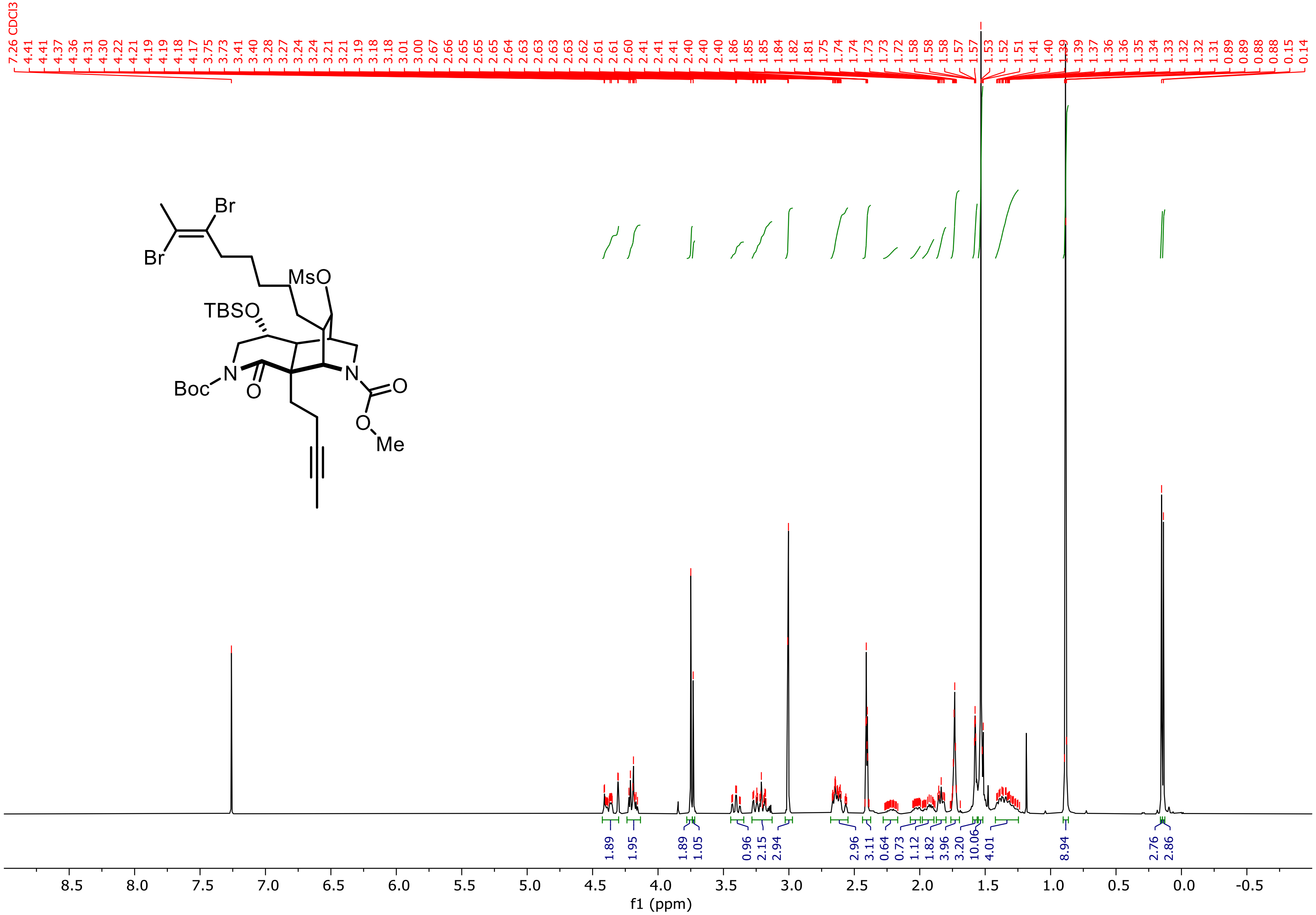

^{13}C NMR Spectrum of S13 (101 MHz, $CDCl_3$)

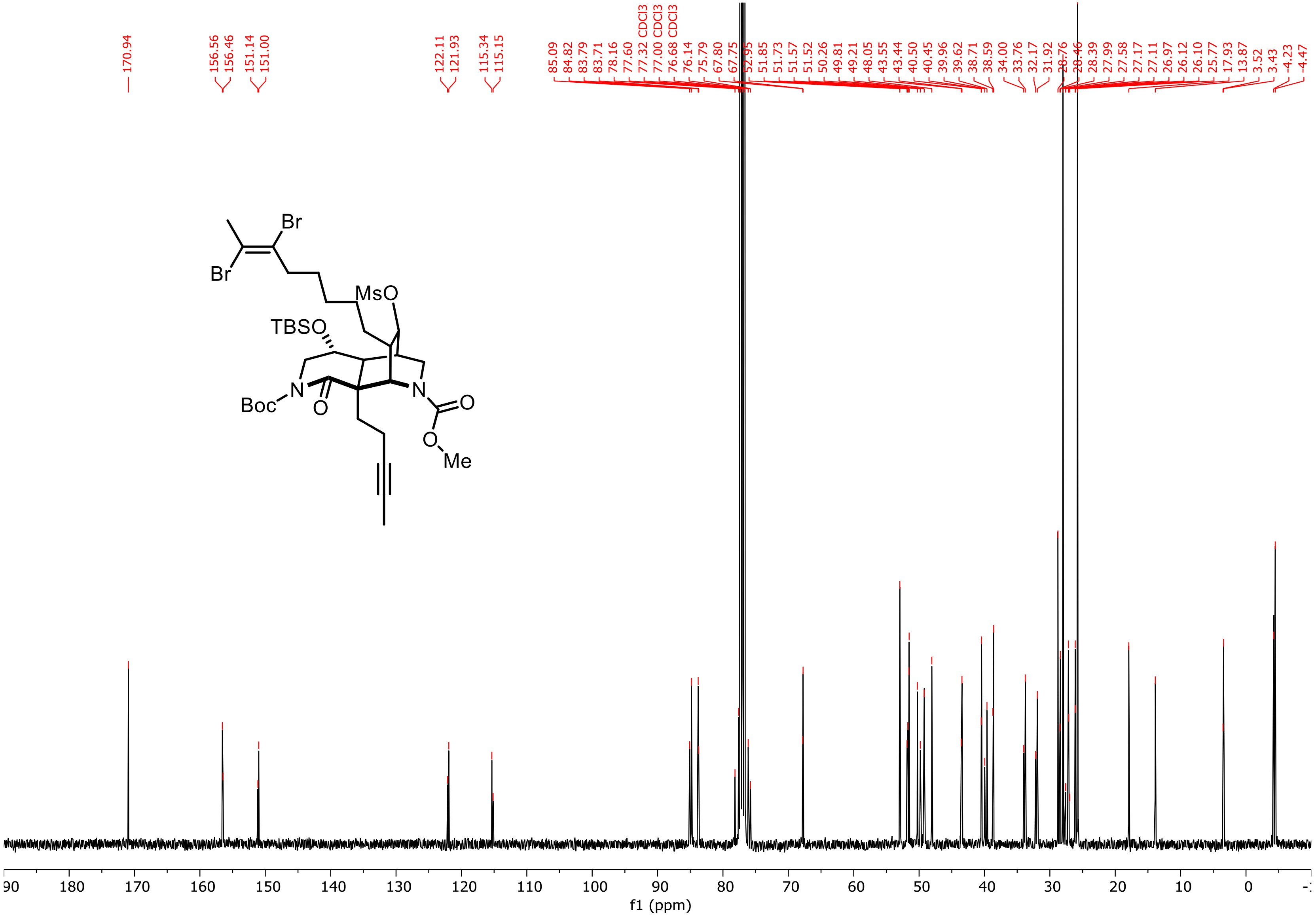

^{1}H NMR Spectrum of 65 (400 MHz, $CDCl_3$)

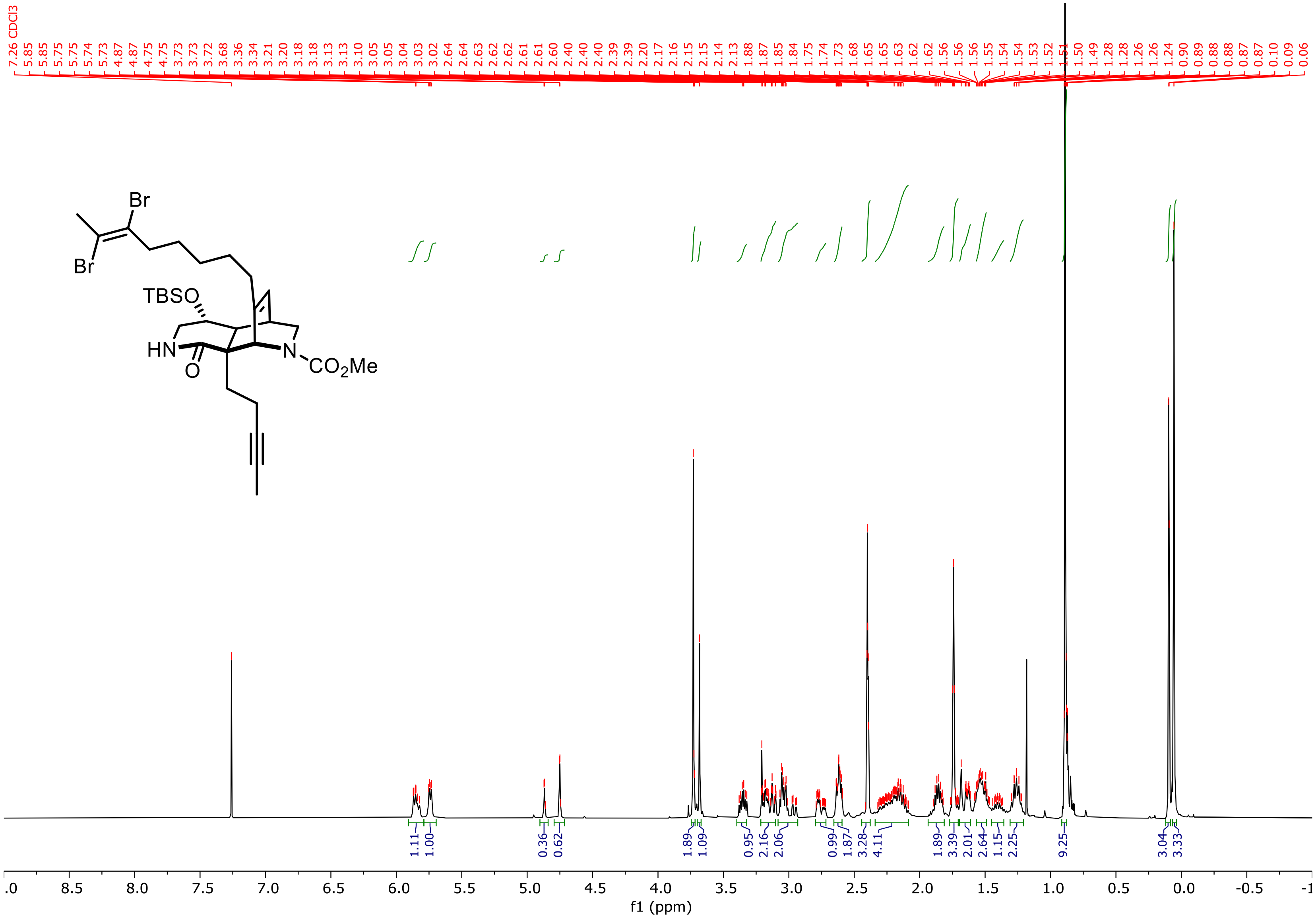

^{13}C NMR Spectrum of 65 (101 MHz, CDCl$_3$)

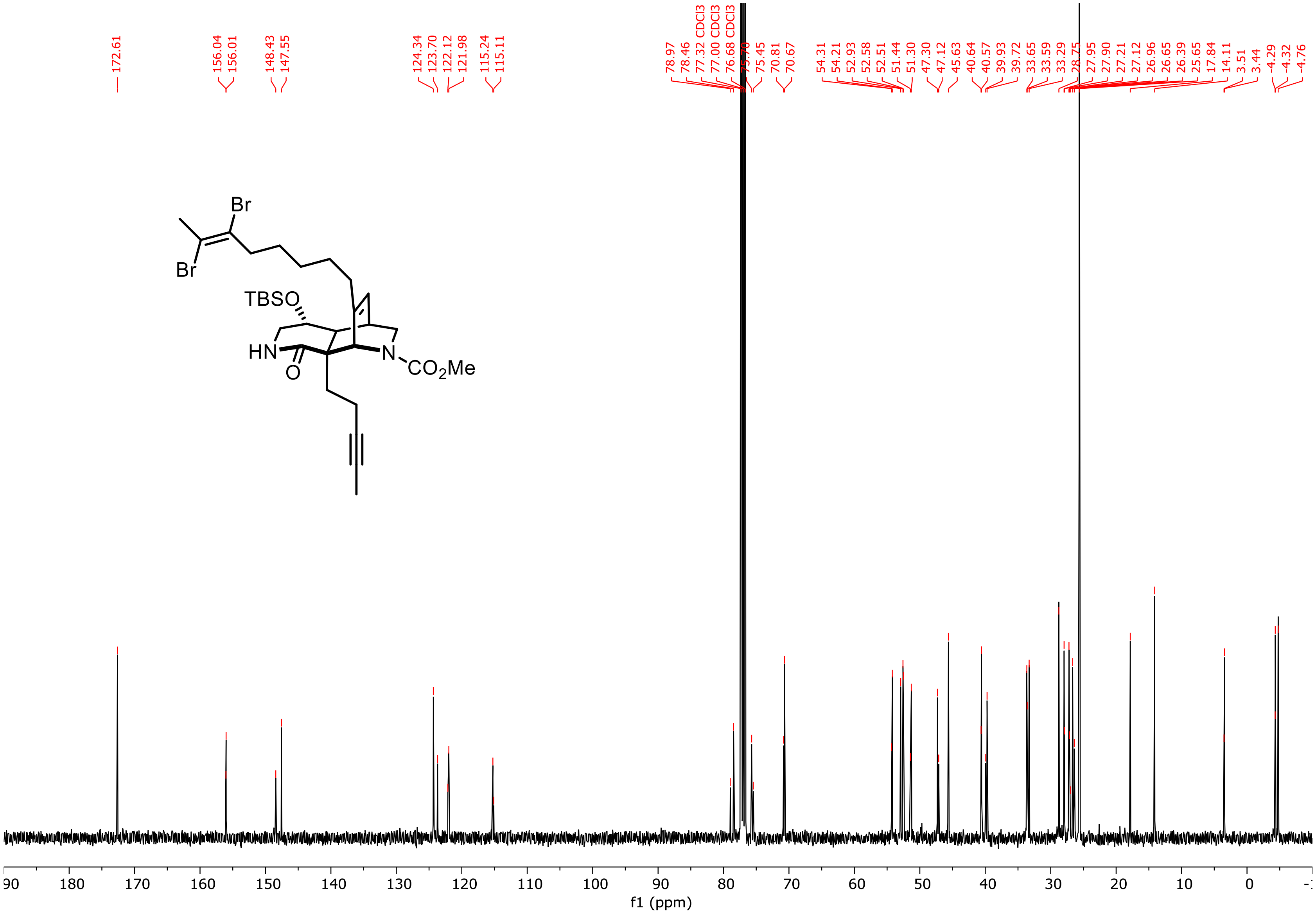

^{1}H NMR Spectrum of 67 (400 MHz, $CDCl_3$)

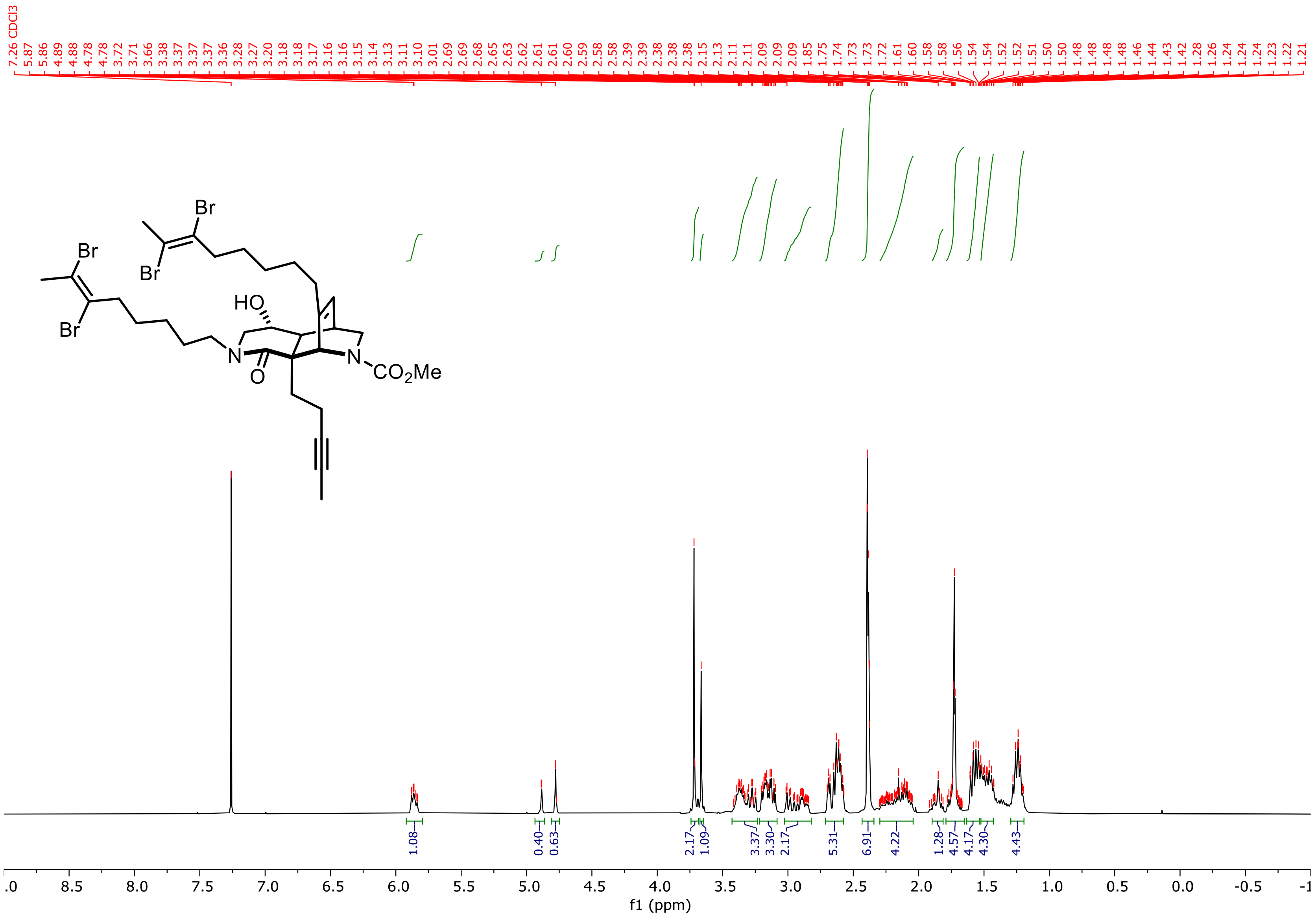

^{13}C NMR Spectrum of 67 (101 MHz, $CDCl_3$)

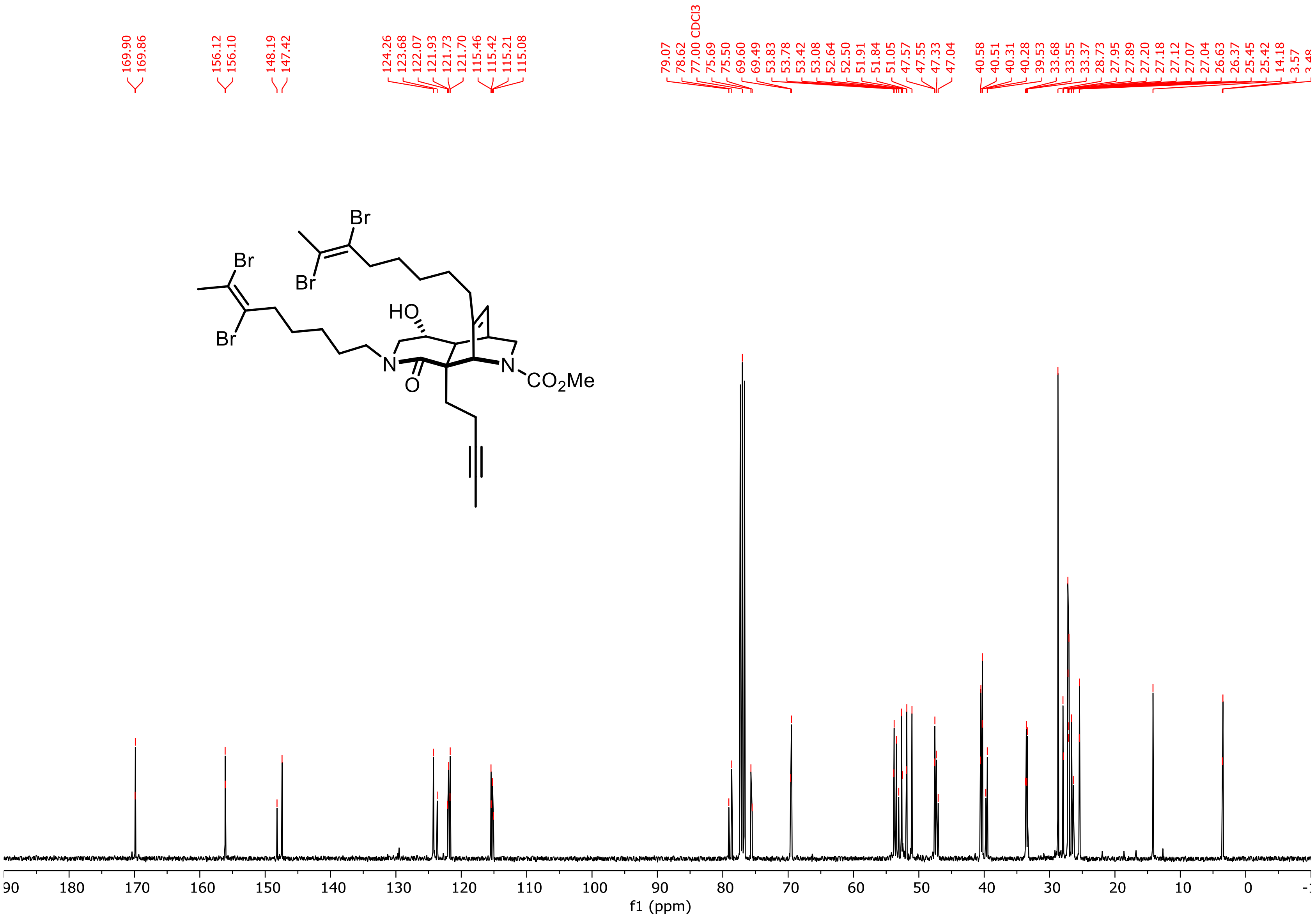

^1H NMR Spectrum of S14 (400 MHz, $CDCl_3$)

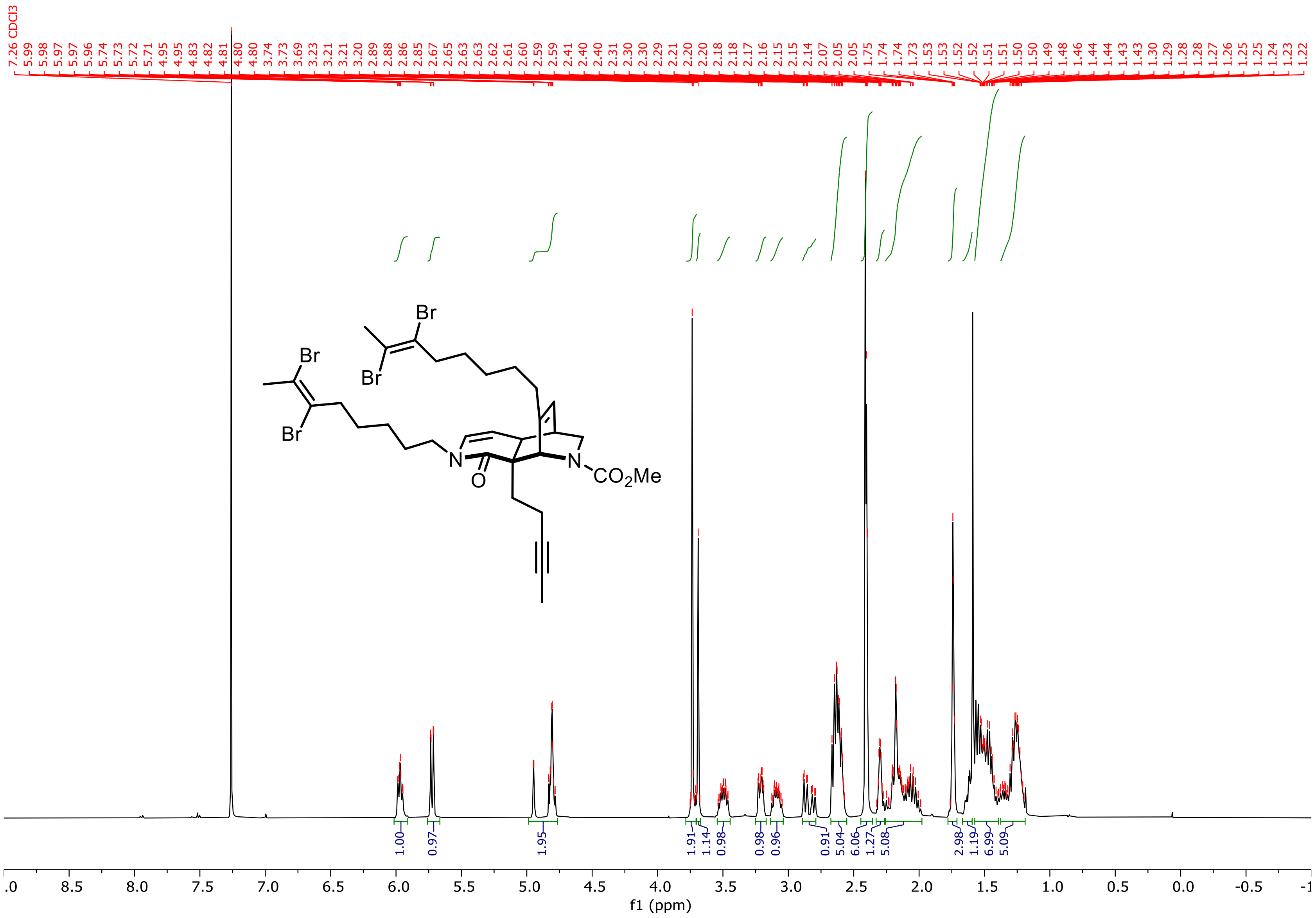

^{13}C NMR Spectrum of S14 (101 MHz, $CDCl_3$)

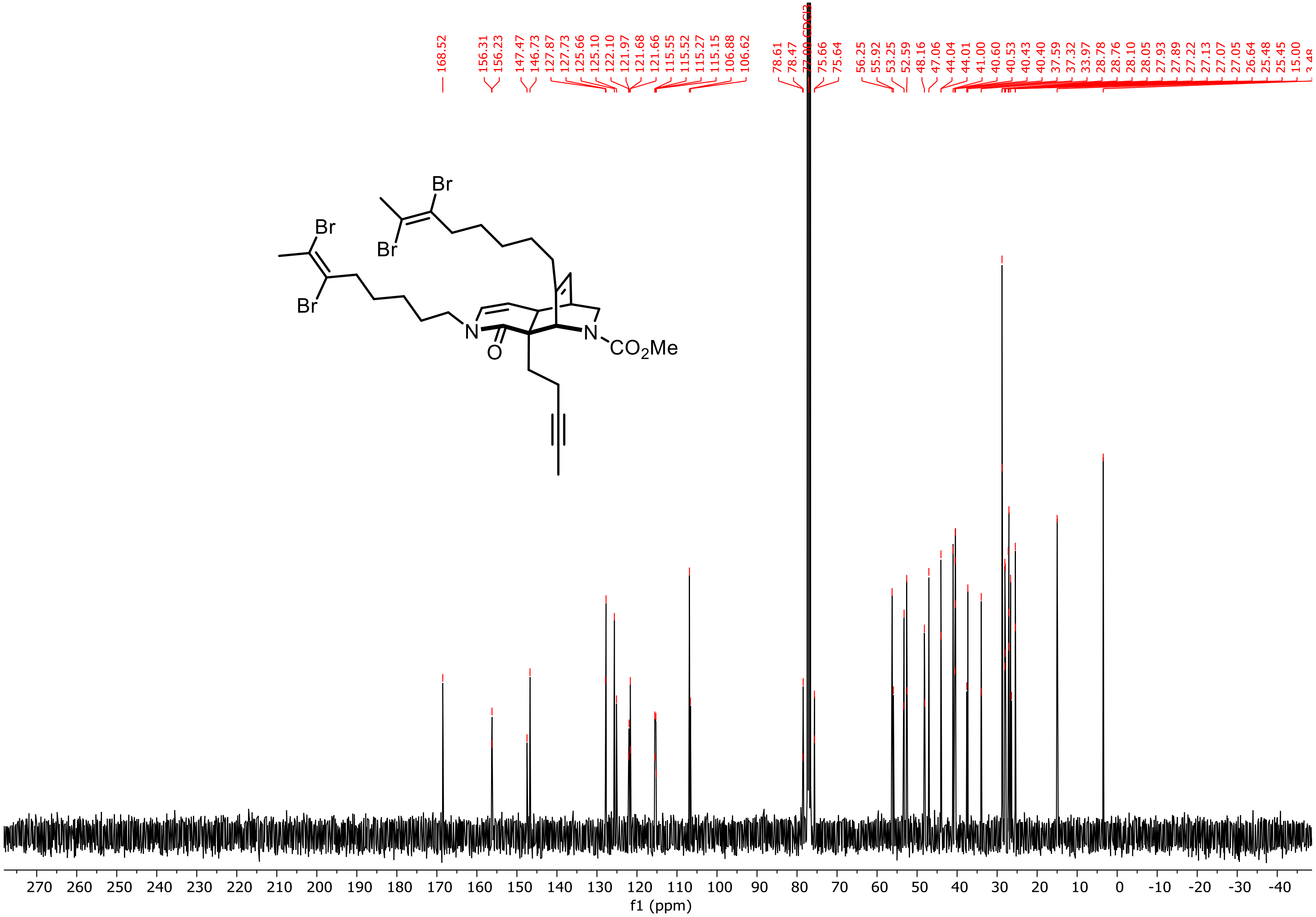

^{1}H NMR Spectrum of 68 (400 MHz, $CDCl_3$)

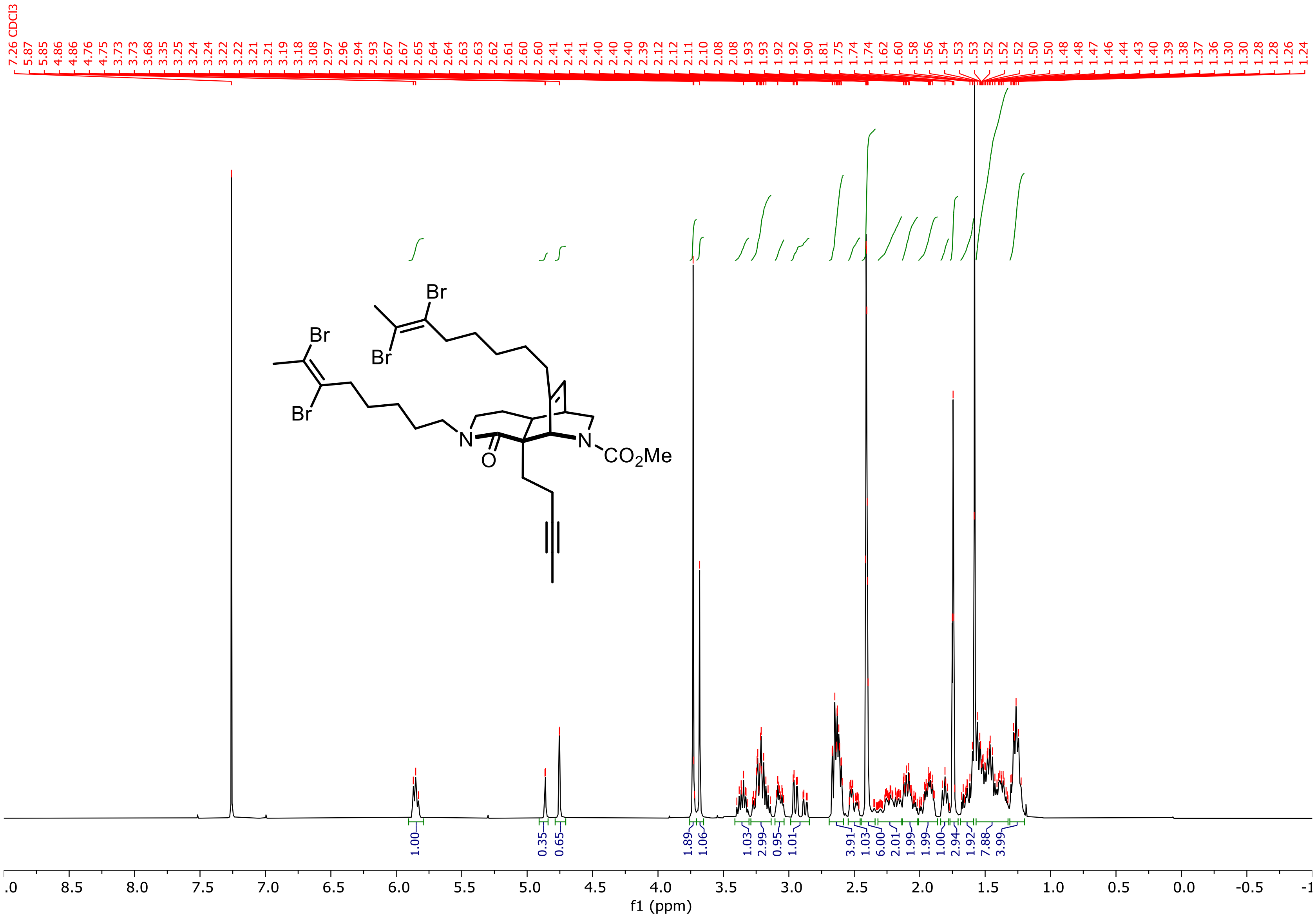

^{13}C NMR Spectrum of 68 (101 MHz, $CDCl_3$)

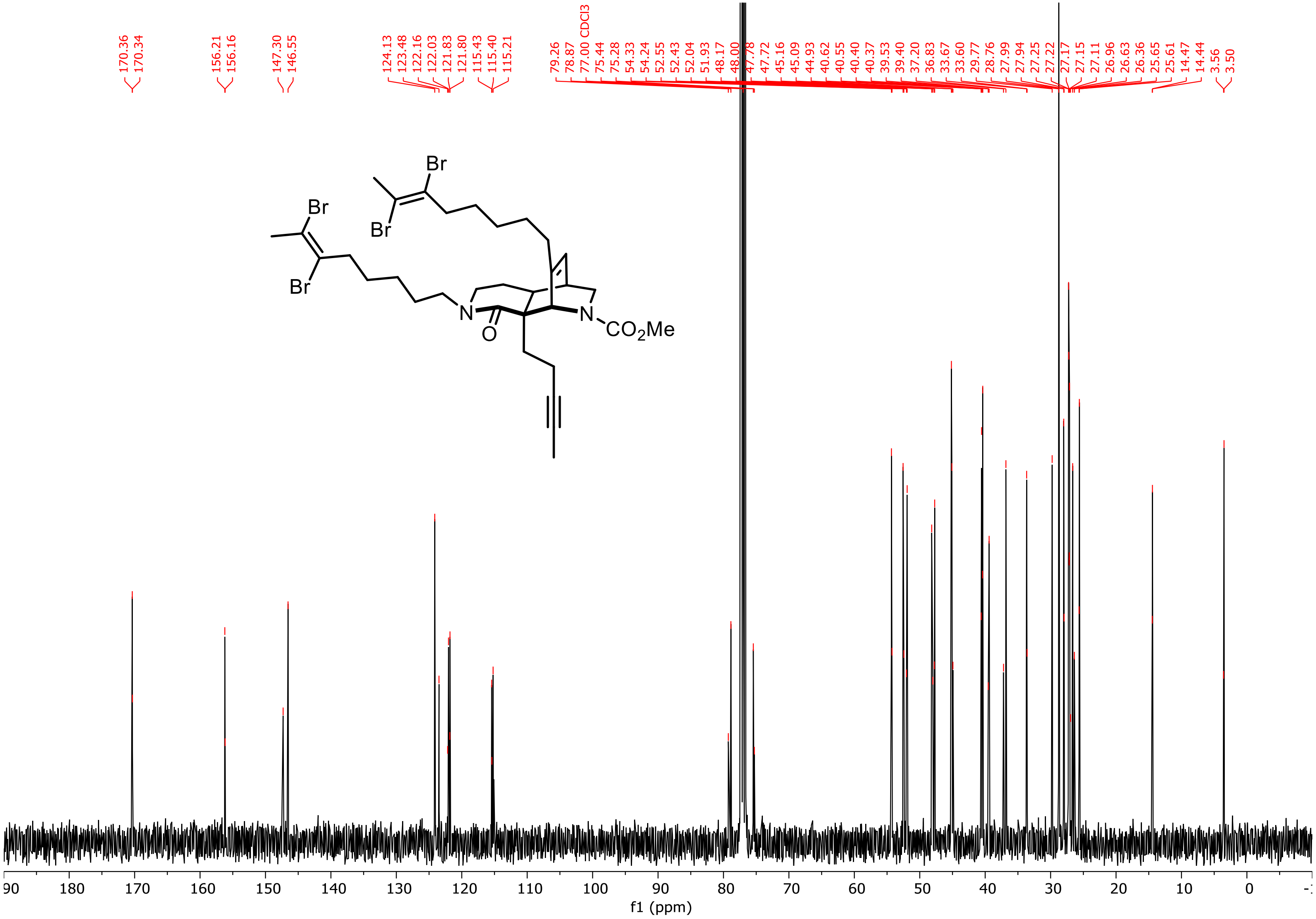

^{1}H NMR Spectrum of 69 (400 MHz, CDCl$_3$)

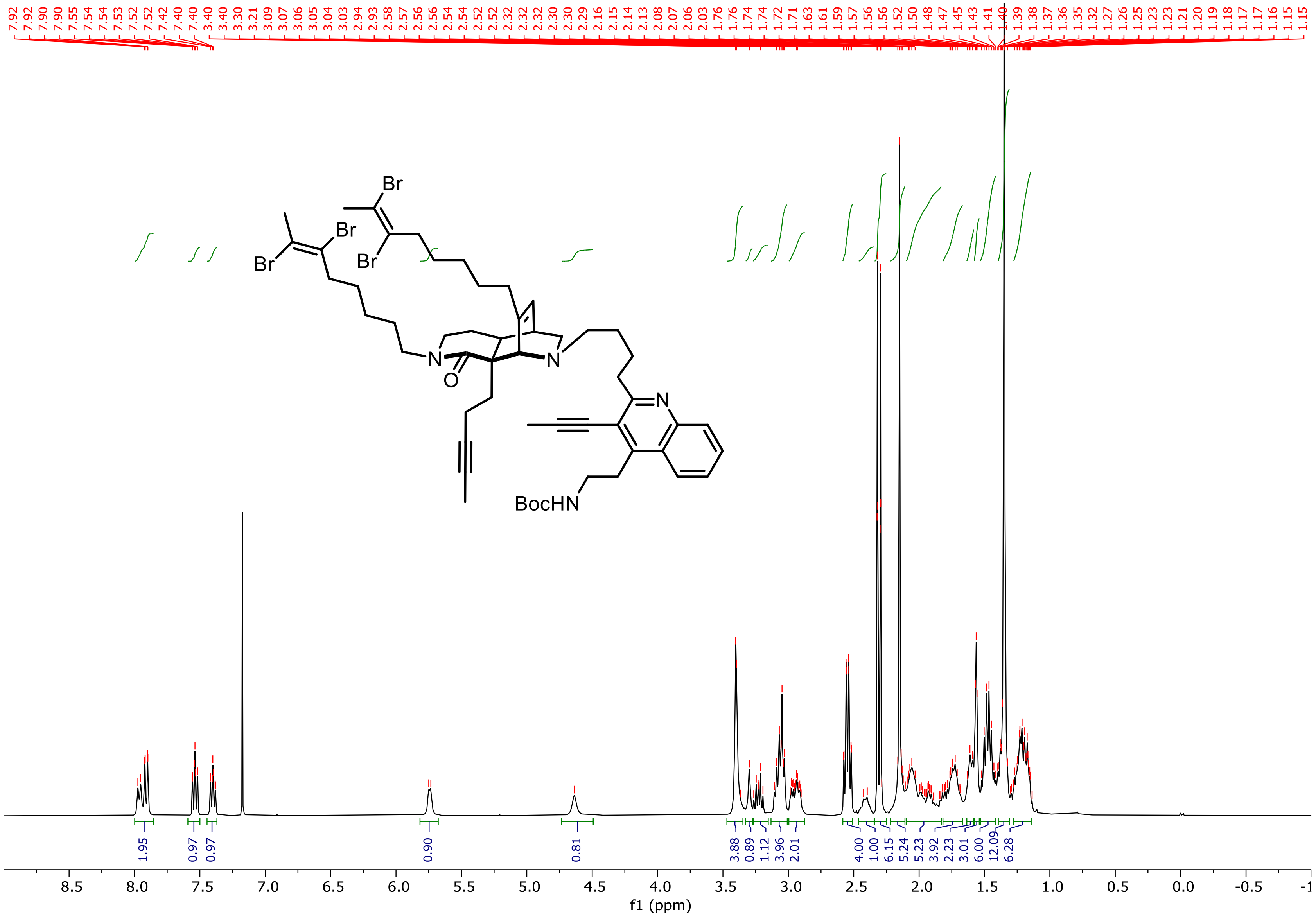

^{13}C NMR Spectrum of 69 (101 MHz, CDCl$_3$)

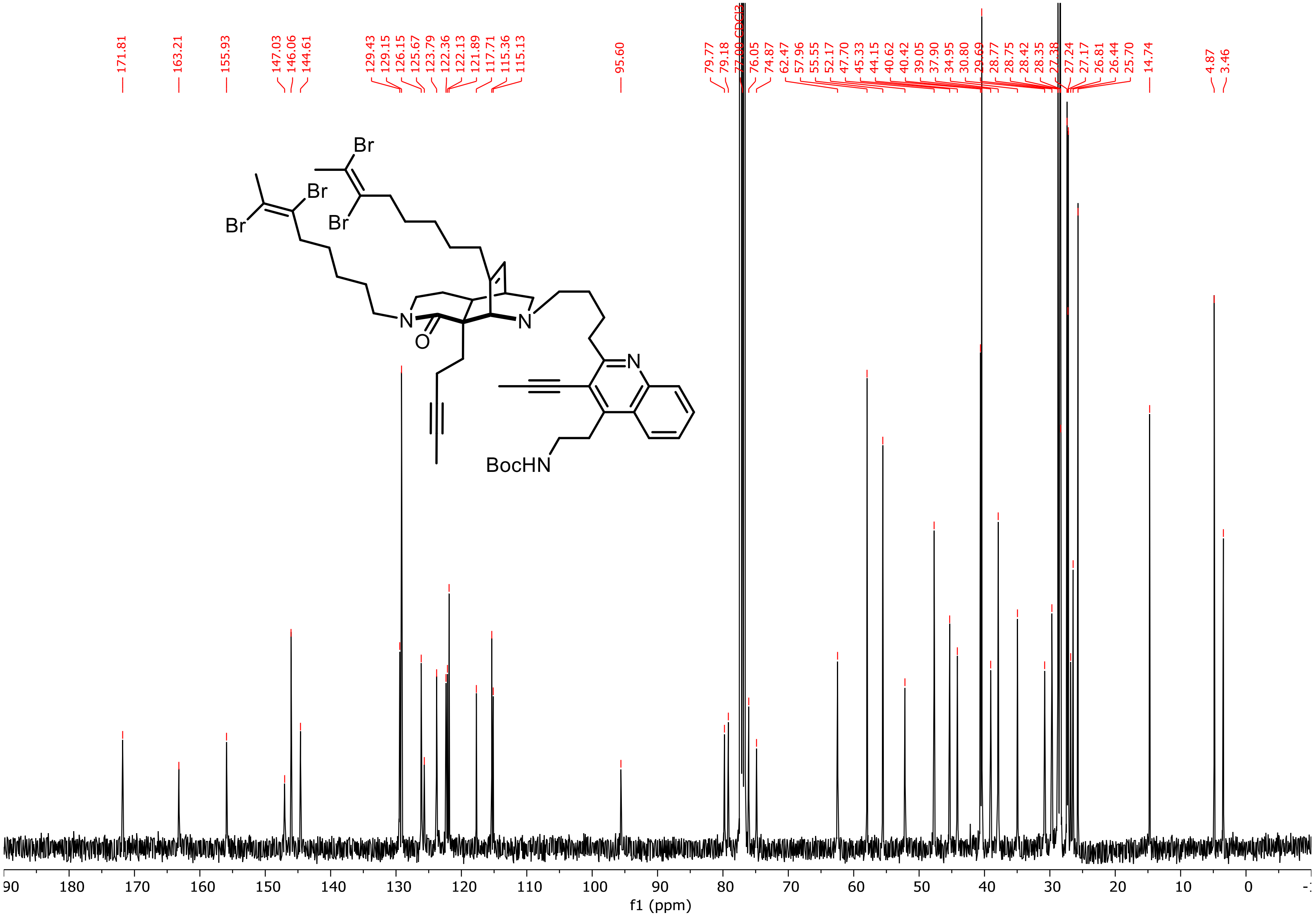

^{1}H NMR Spectrum of 70 (400 MHz, $CDCl_3$)

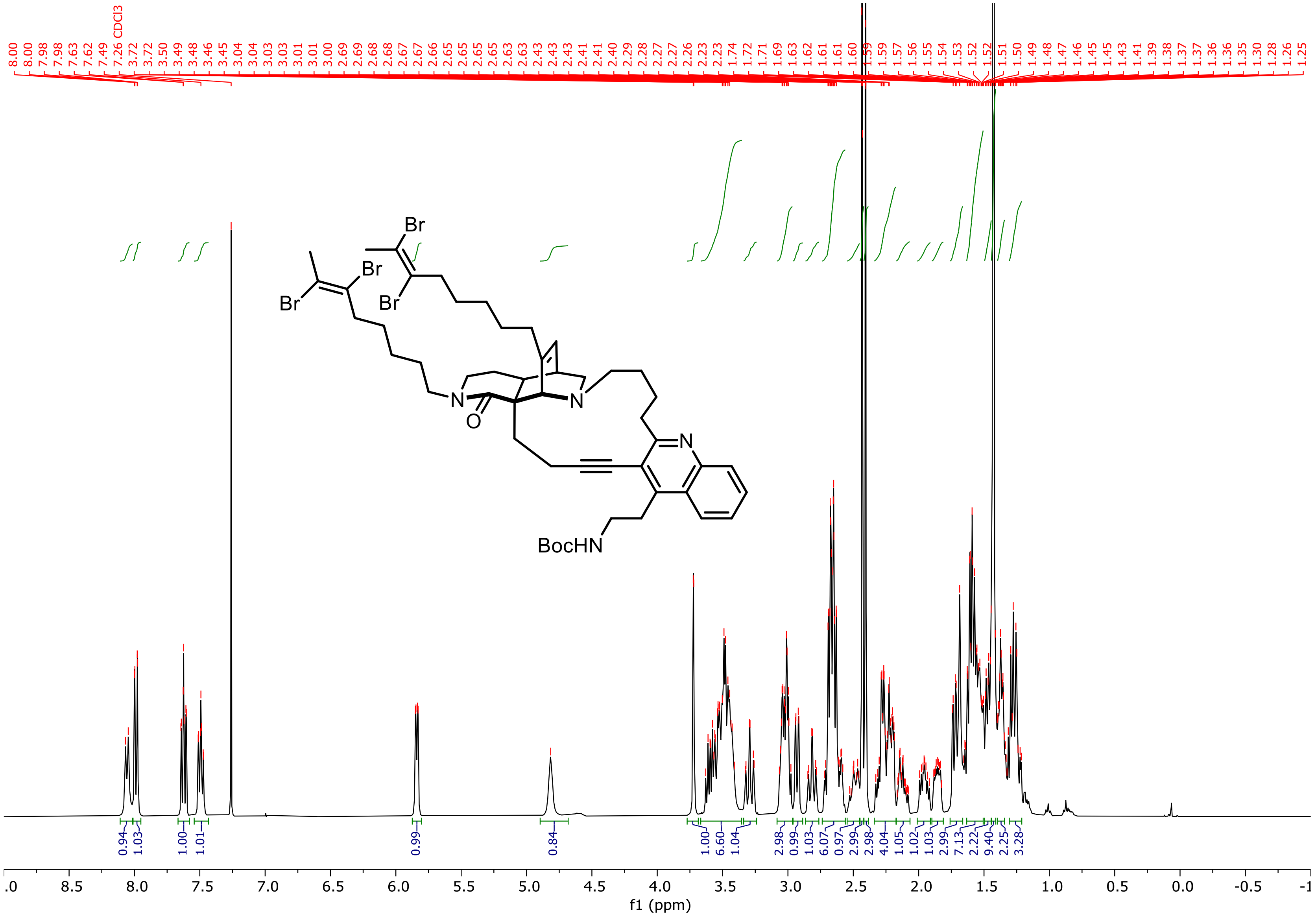

^{13}C NMR Spectrum of 70 (101 MHz, $CDCl_3$)

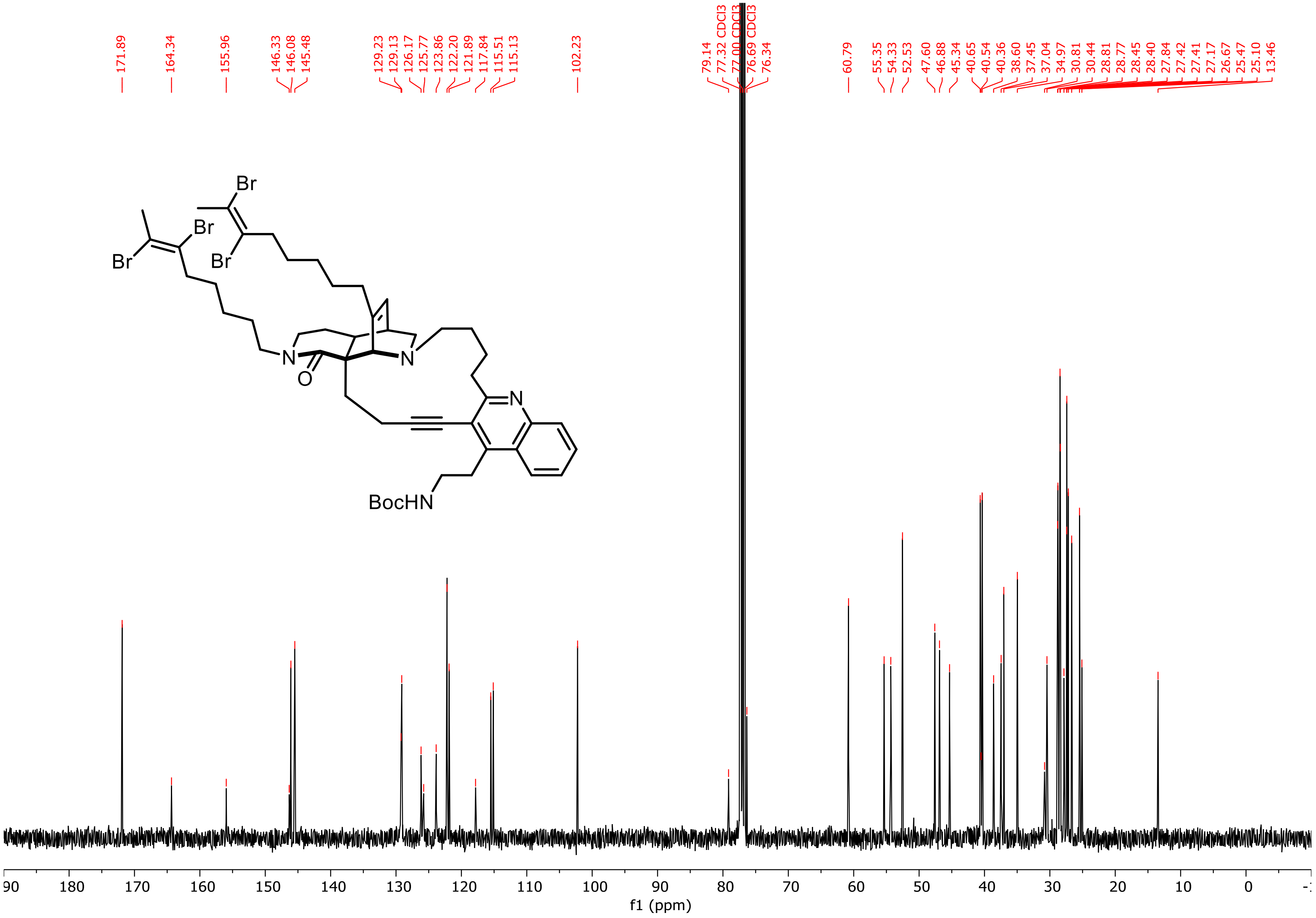

^{1}H NMR Spectrum of S15 (600 MHz, $CDCl_3$)

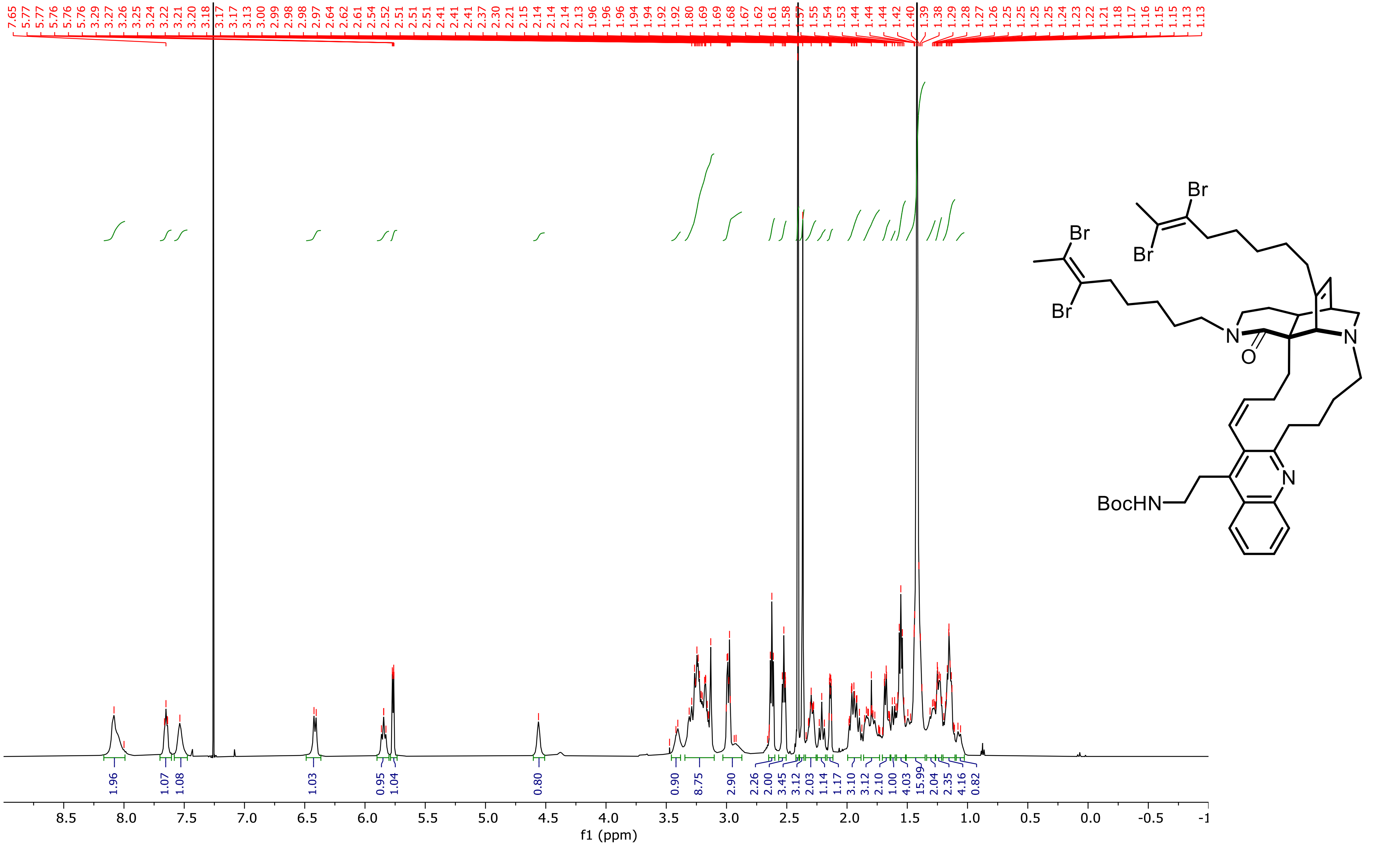

^{13}C NMR Spectrum of S15 (151 MHz, $CDCl_3$)

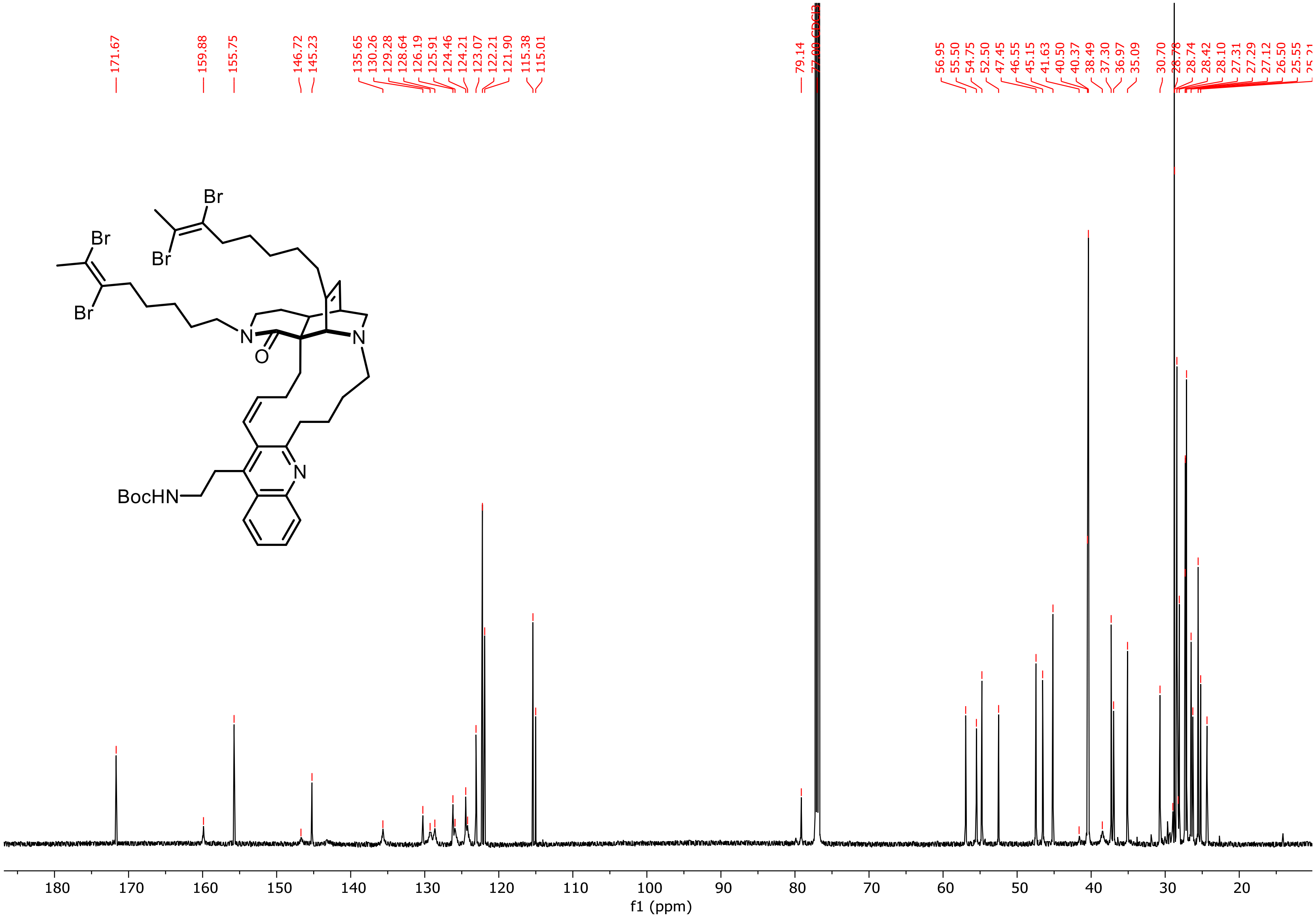

HSQC Spectrum of S15 ($CDCl_3$)

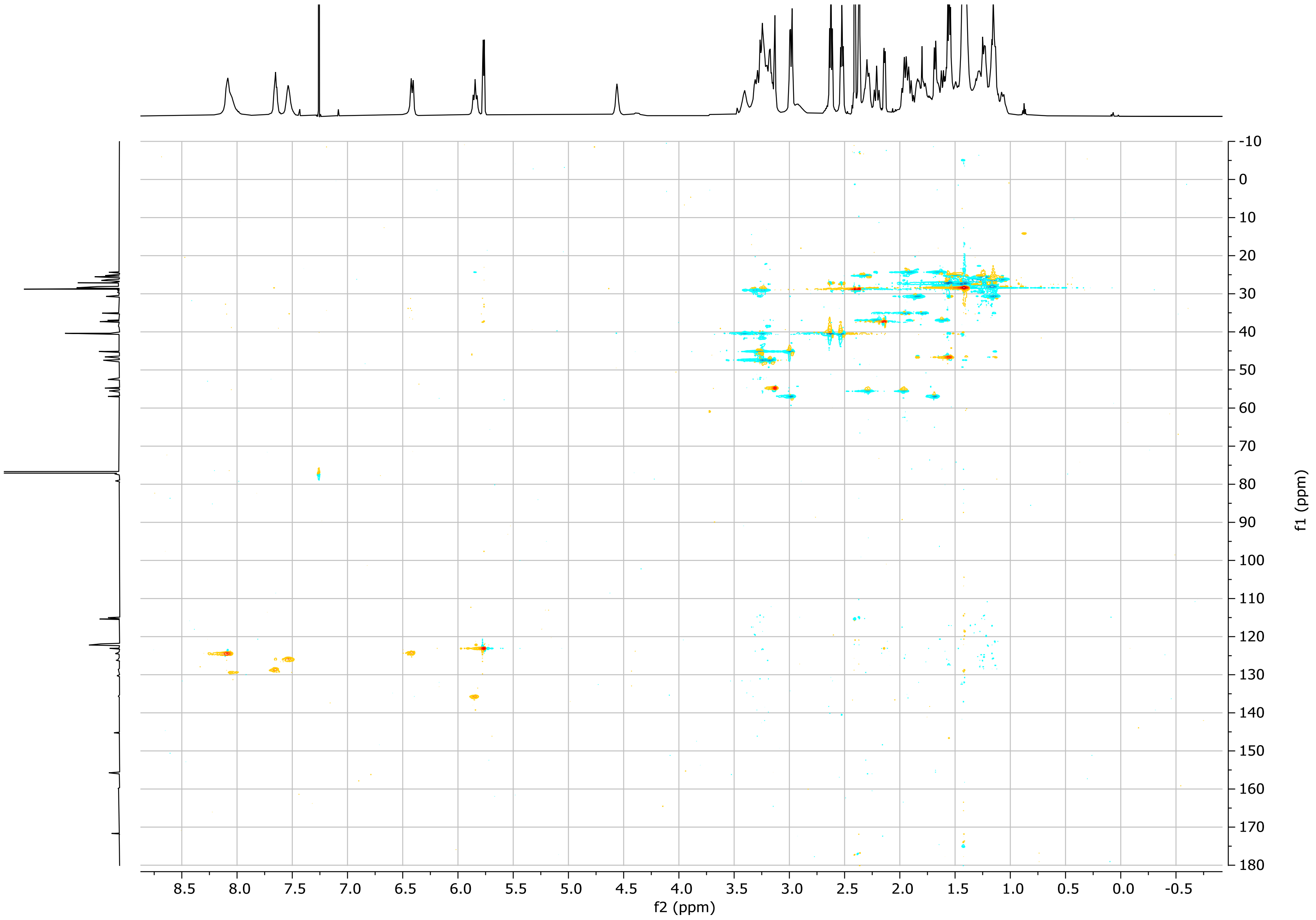

HMBC Spectrum of S15 ($CDCl_3$)

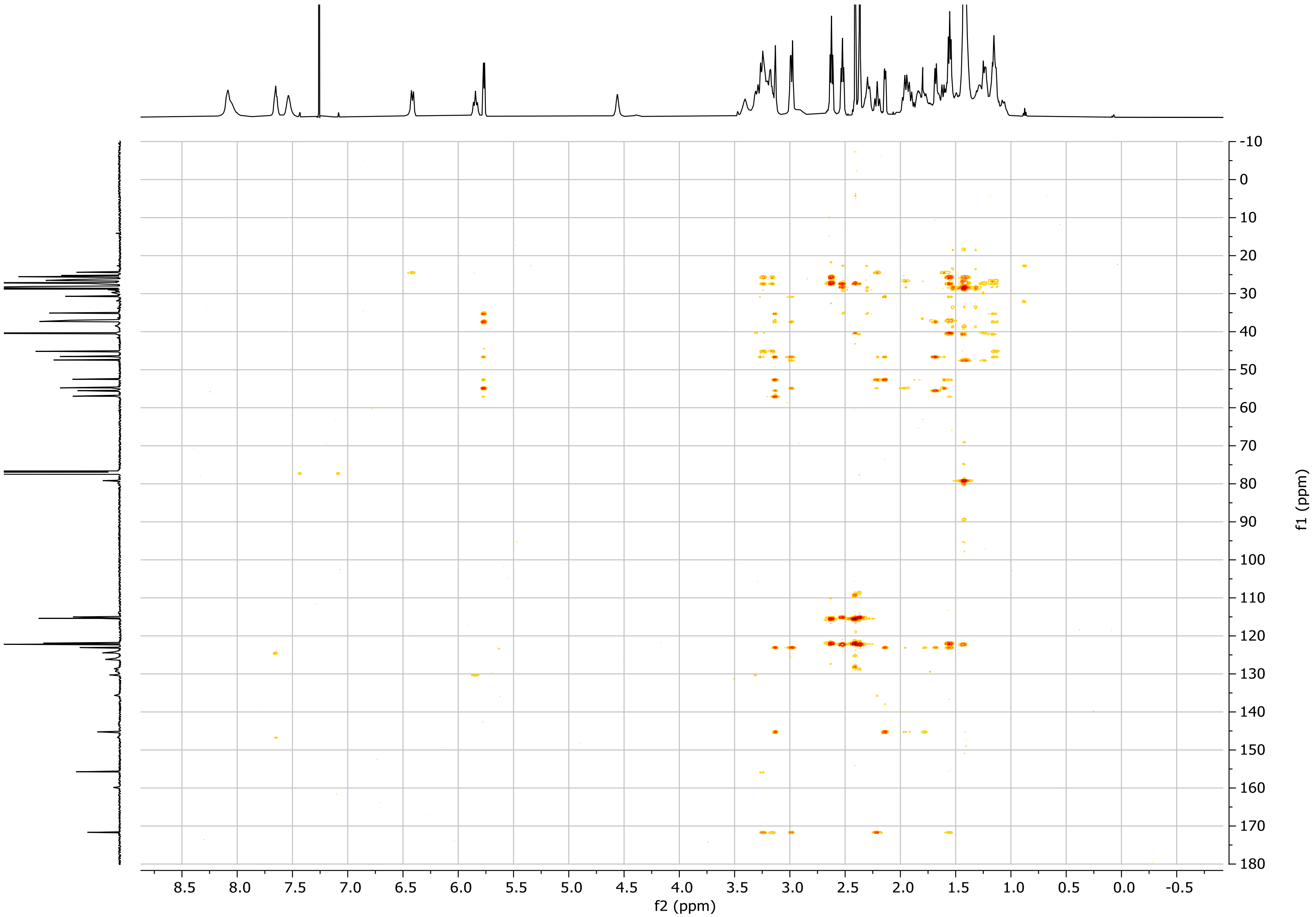

^{1}H NMR Spectrum of 59 (400 MHz, $CDCl_3$)

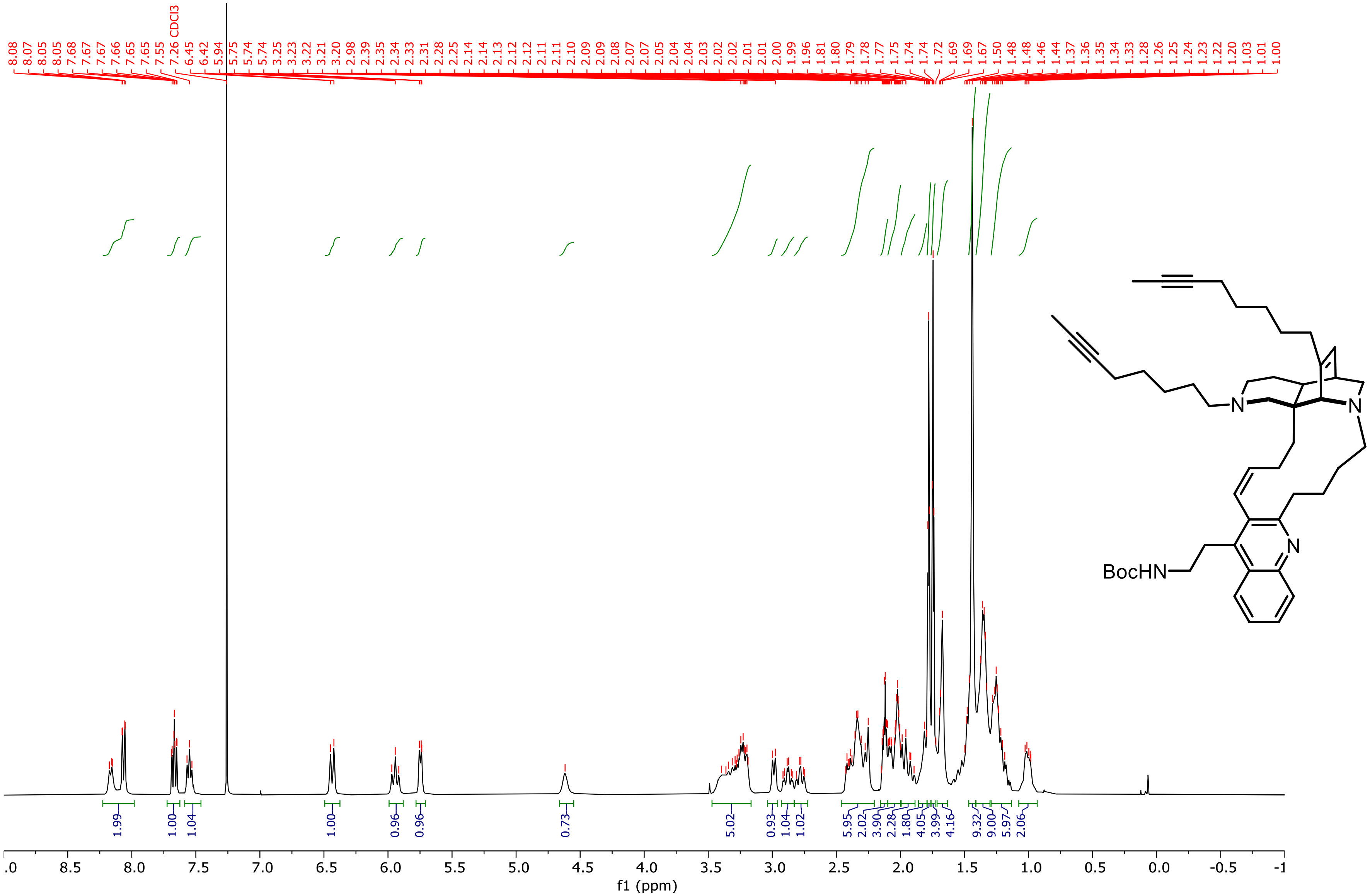

^{13}C NMR Spectrum of 59 (101 MHz, CDCl$_3$)

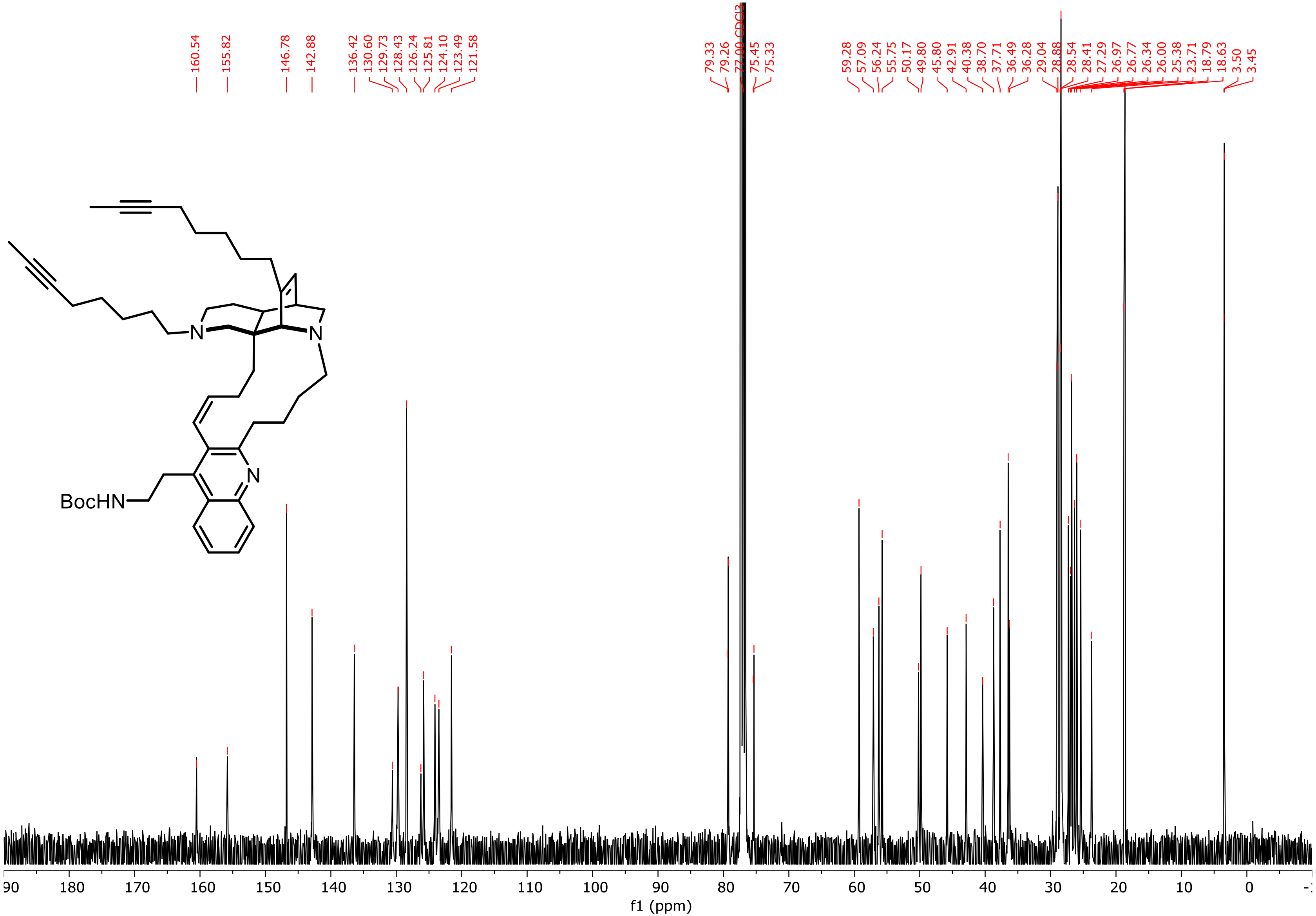

^{1}H NMR Spectrum of 71 (400 MHz, $CDCl_3$)

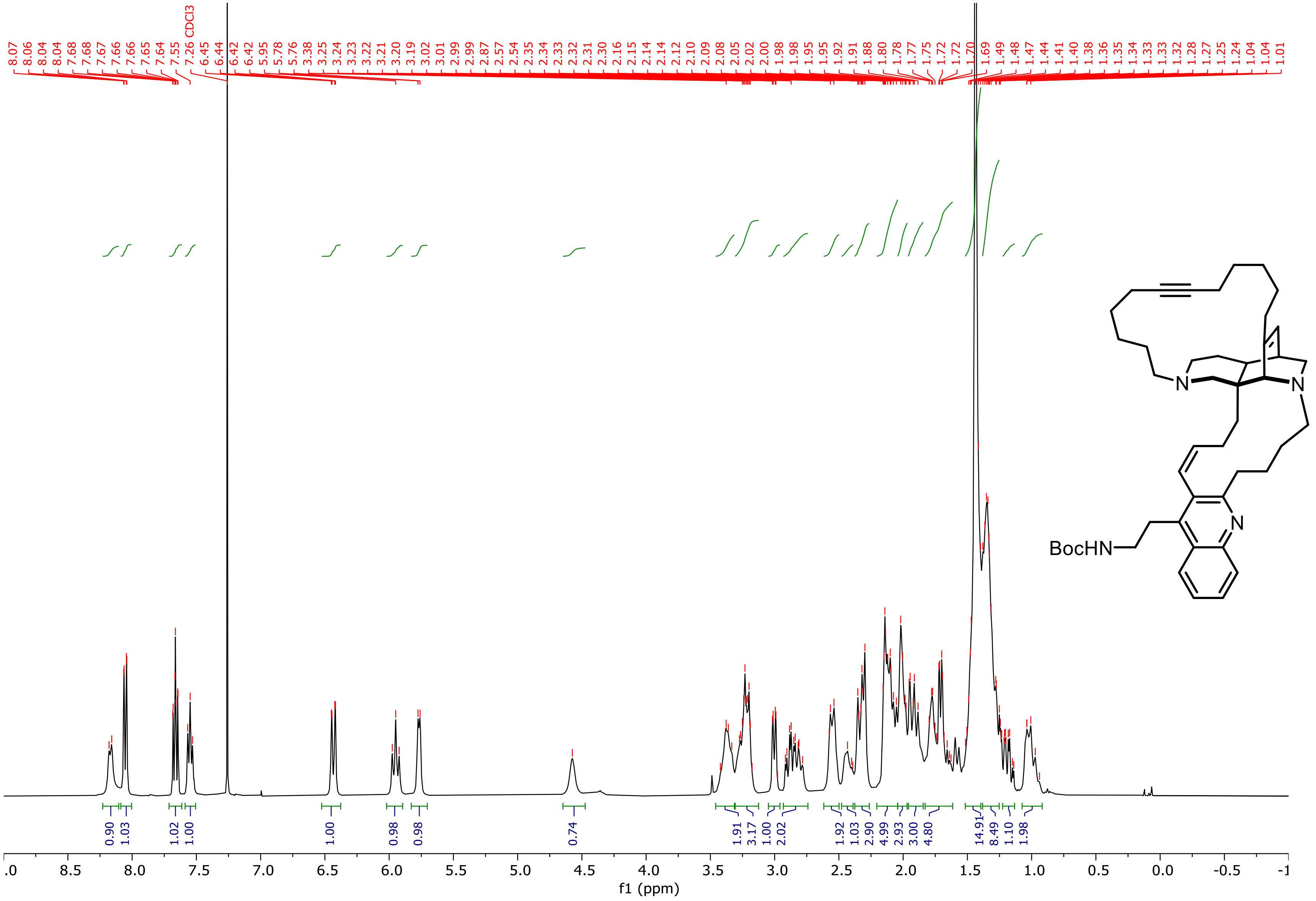

^{13}C NMR Spectrum of 71 (101 MHz, CDCl$_3$)

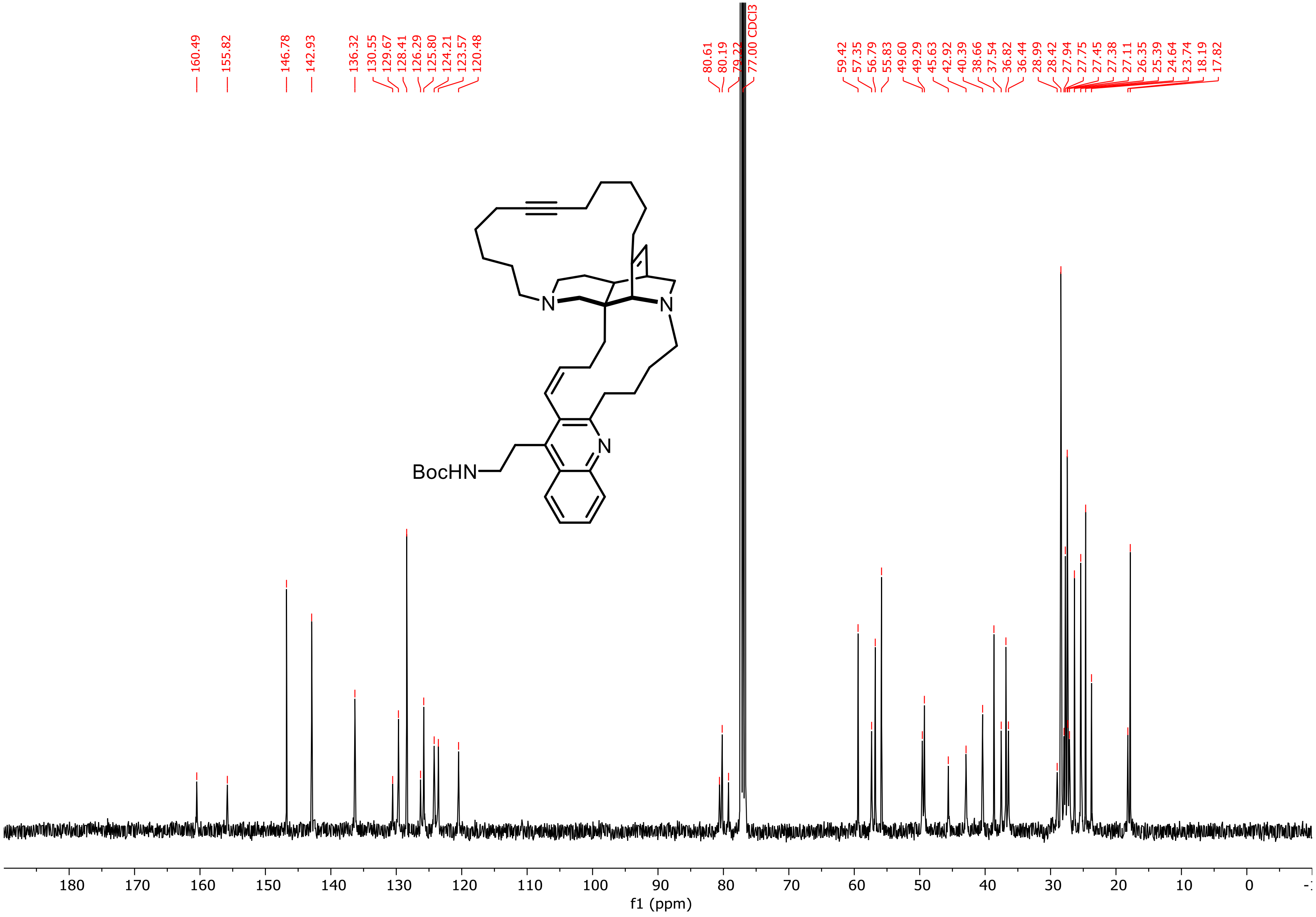

^{1}H NMR Spectrum of Njaoamine I (600 MHz, ca. 7.0 mg in [D$_5$]-pyridine)

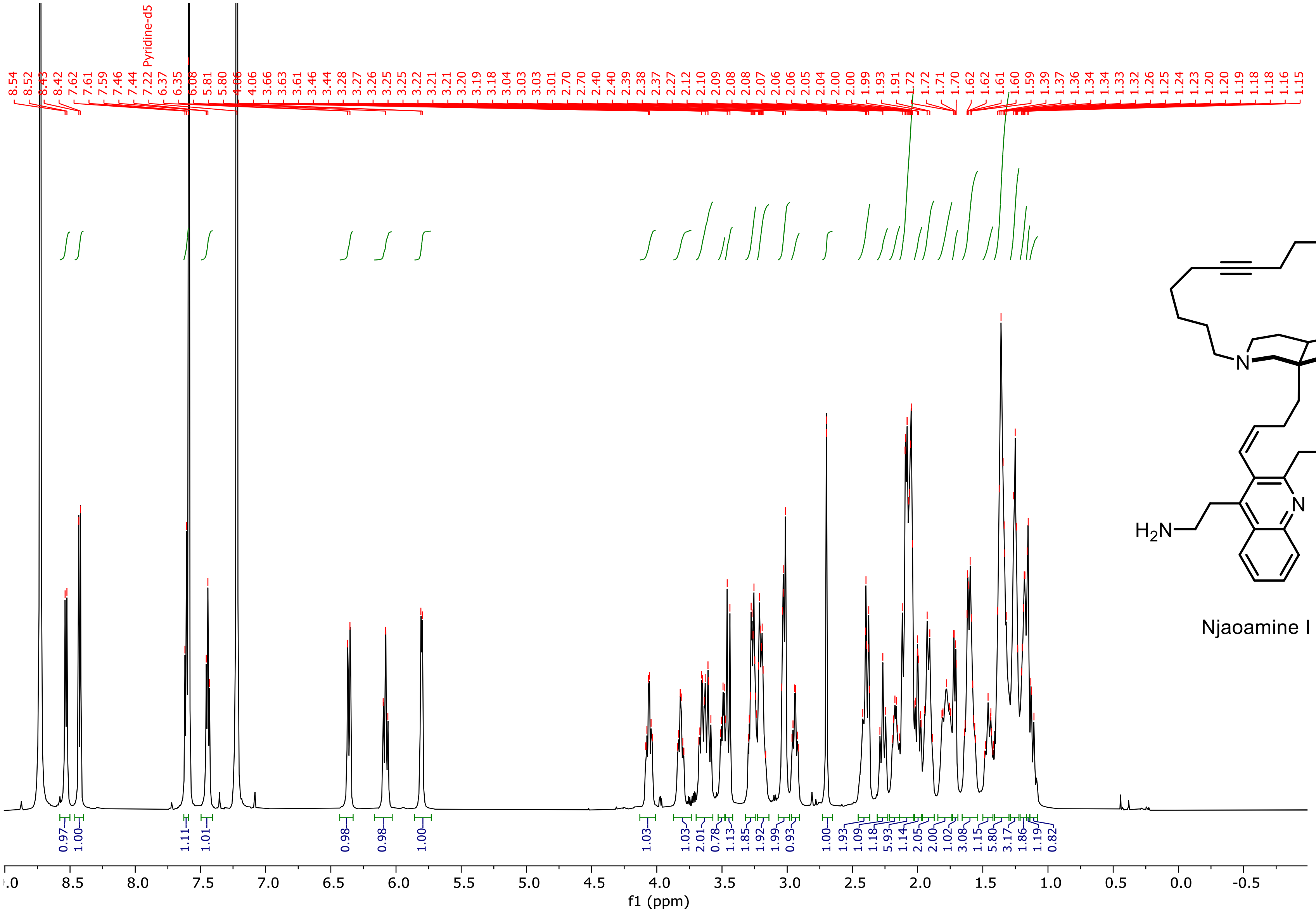

^{13}C NMR Spectrum of Njaoamine I (151 MHz, ca. 7.0 mg in [D$_5$]-pyridine)

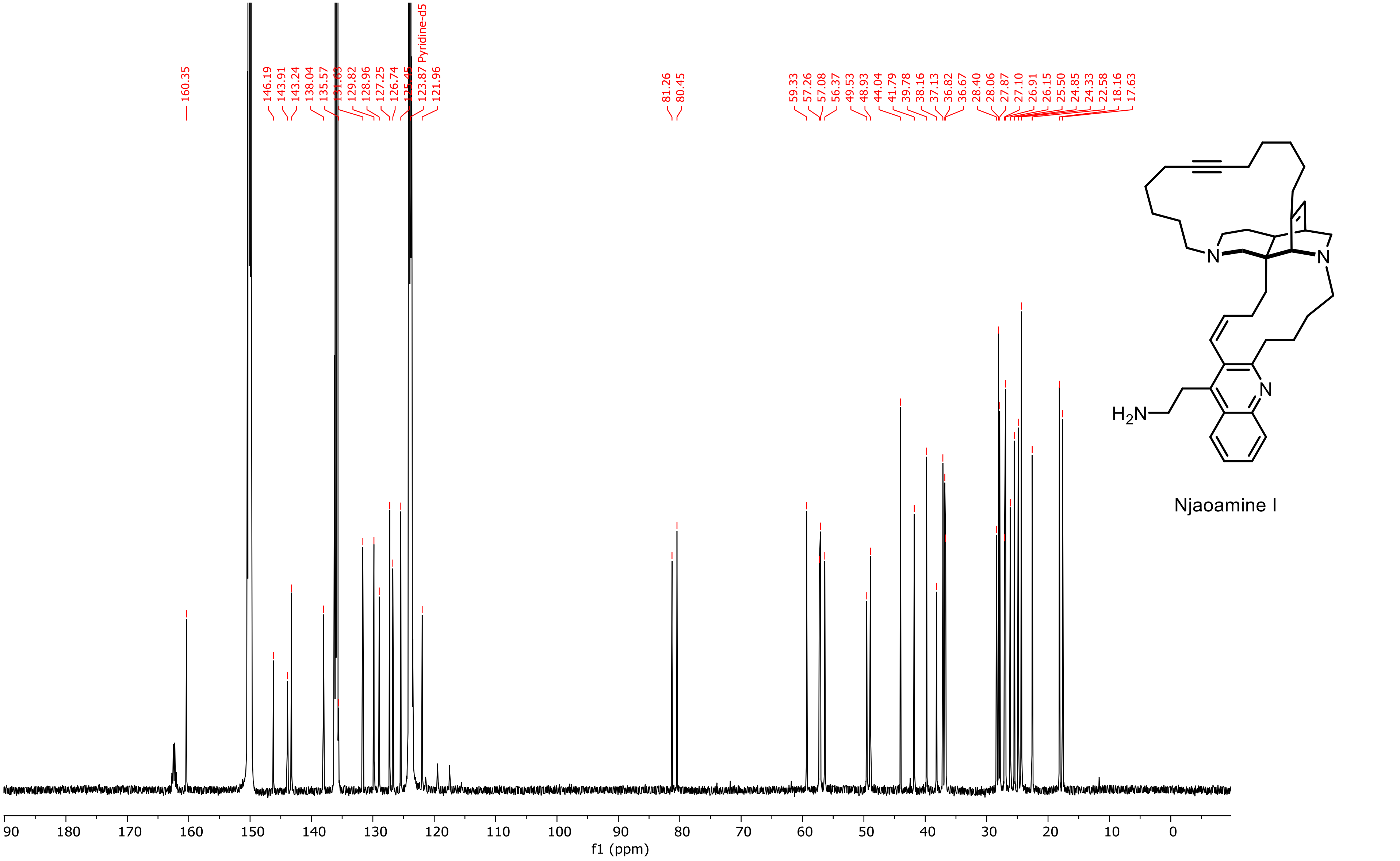

HSQC Spectrum of Njaoamine I (ca. 7.0 mg in [D$_5$]-pyridine)

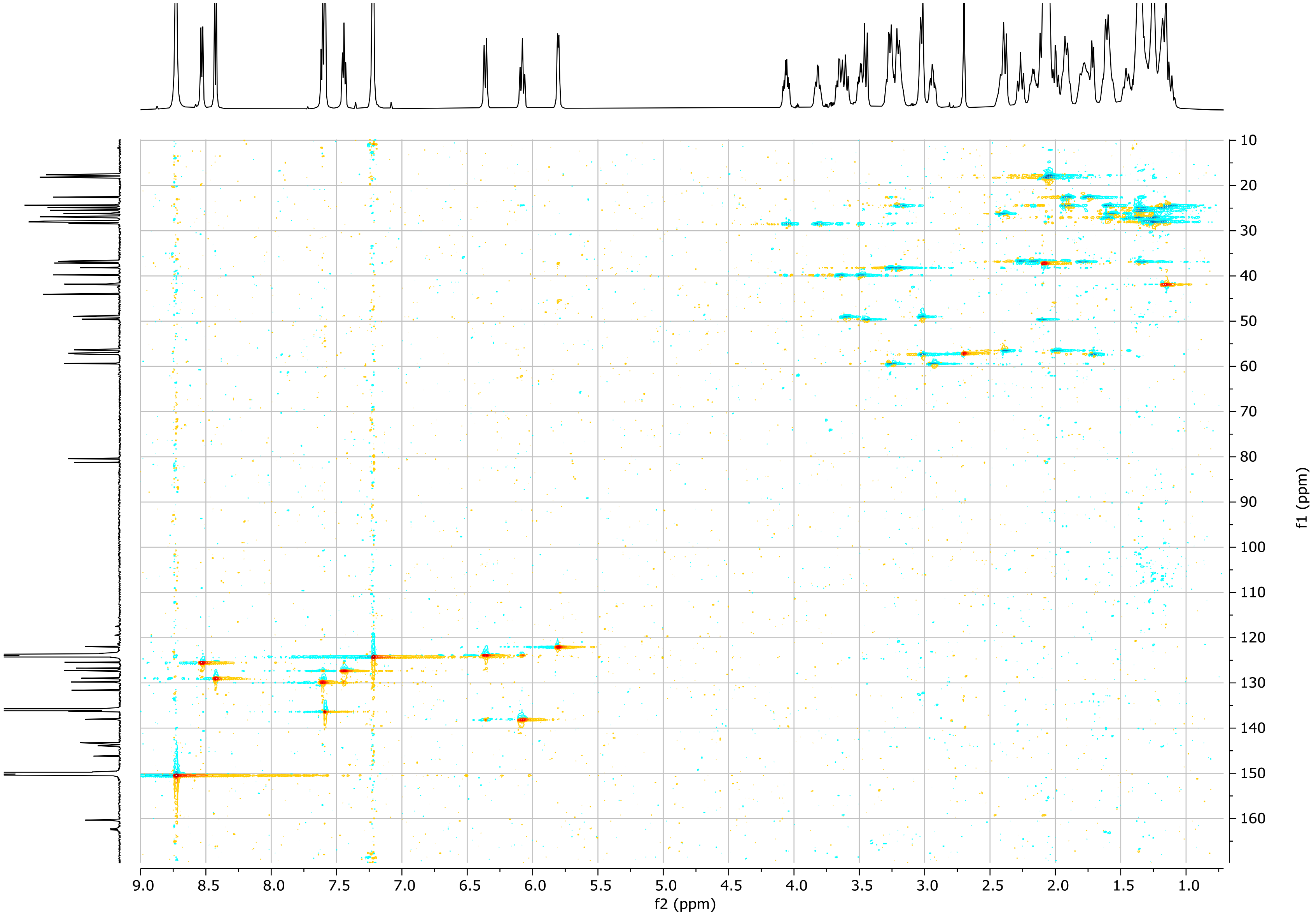

^{1}H NMR Spectrum of Njaoamine I (600 MHz, ca. 2.6 mg in [D_5]-pyridine, 298 K)

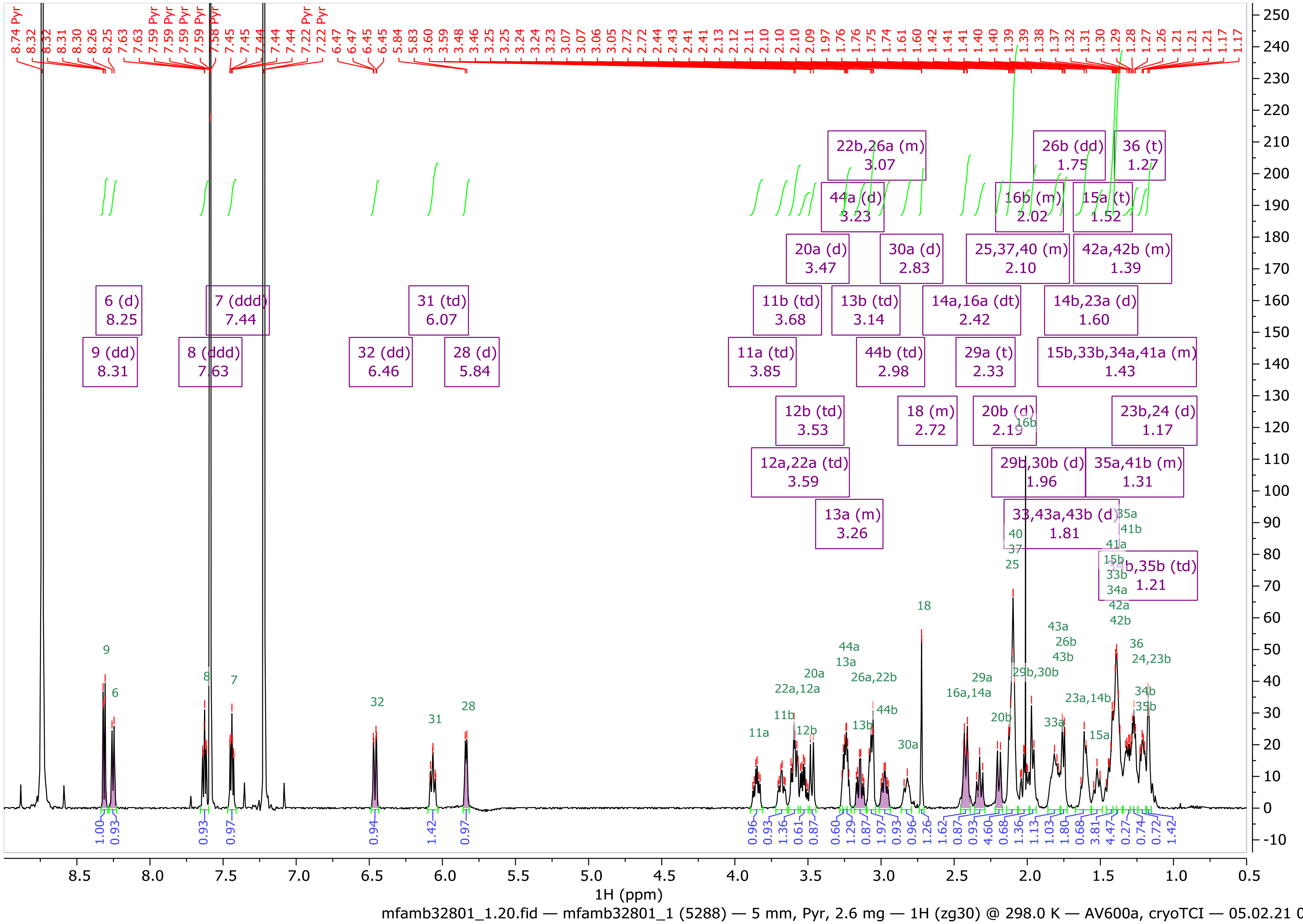

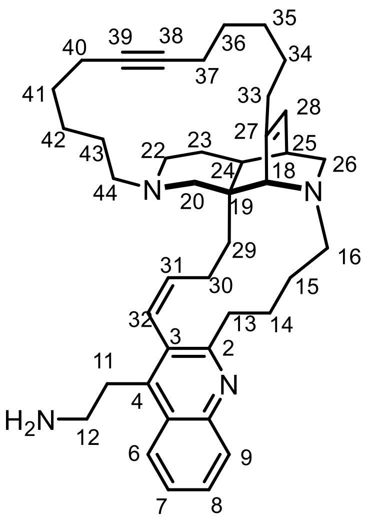

Njaoamine I

^{13}C NMR Spectrum of Njaoamine I (151 MHz, ca. 2.6 mg in [D_5]-pyridine, 298 K)

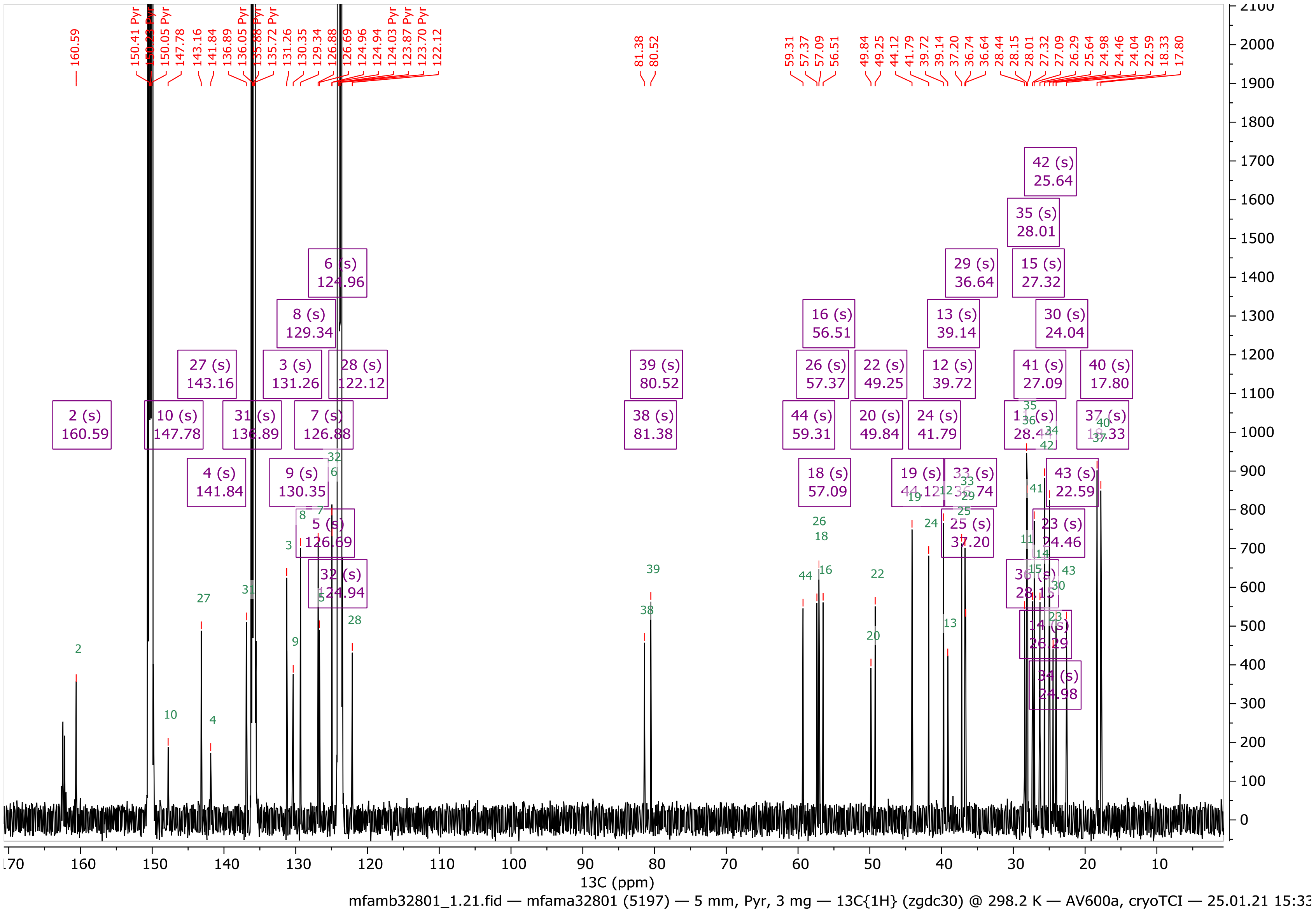

COSY Spectrum of Njaoamine I (ca. 2.6 mg in [D$_5$]-pyridine, 298 K)

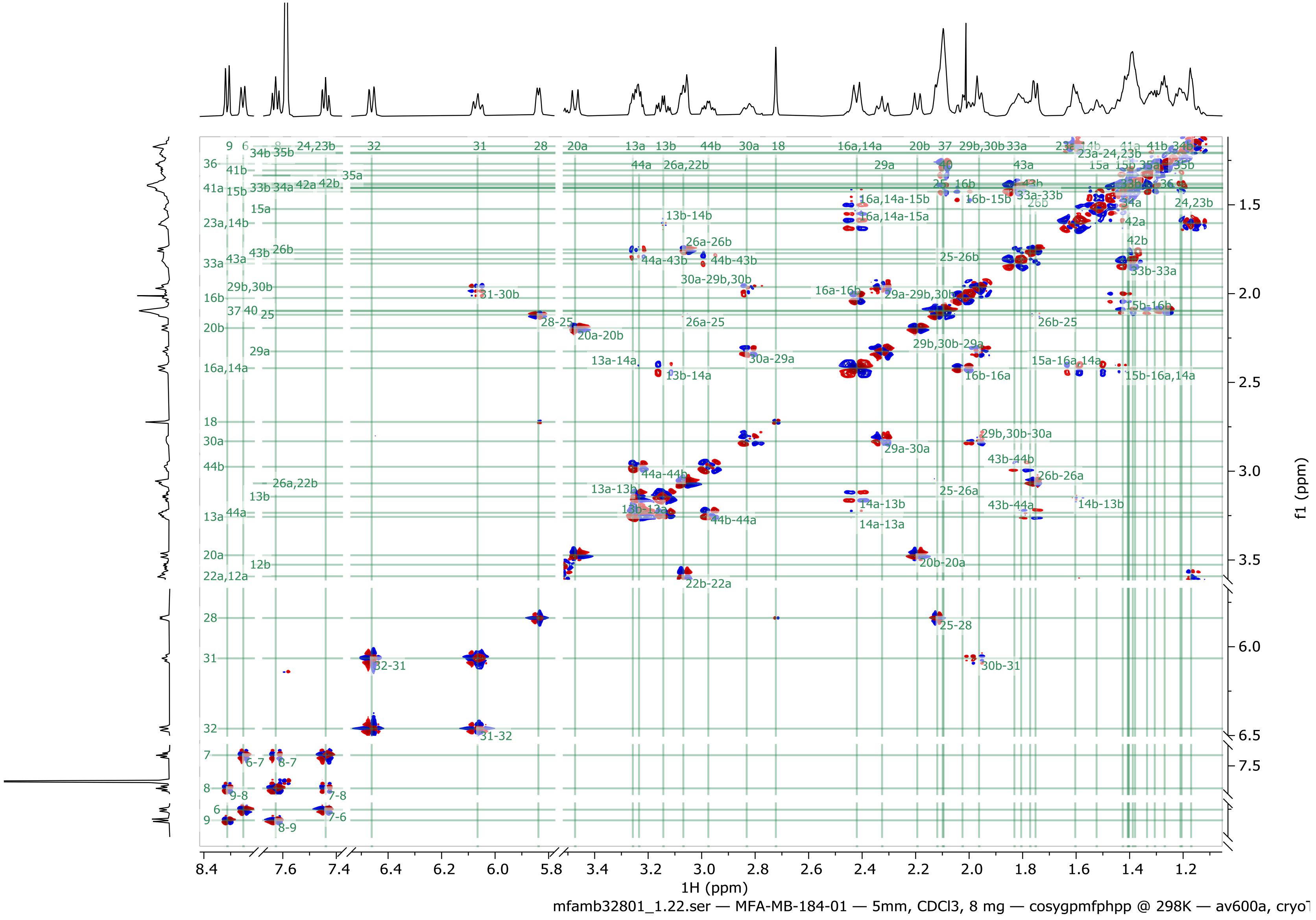

HSQC Spectrum of Njaoamine I (ca. 2.6 mg in [D$_5$]-pyridine, 298 K)

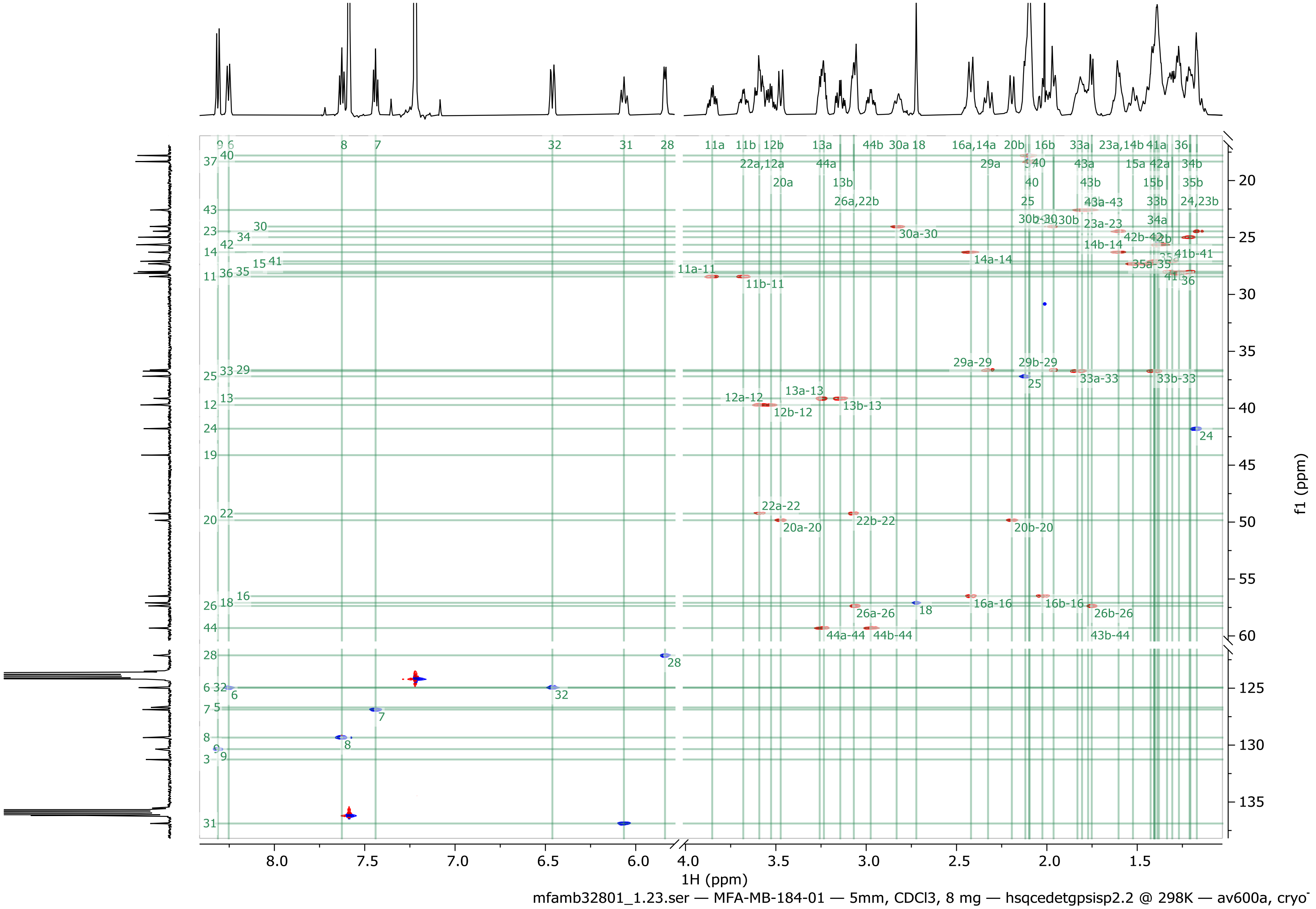

HMBC Spectrum of Njaoamine I (ca. 2.6 mg in [D_5]-pyridine, 298 K)

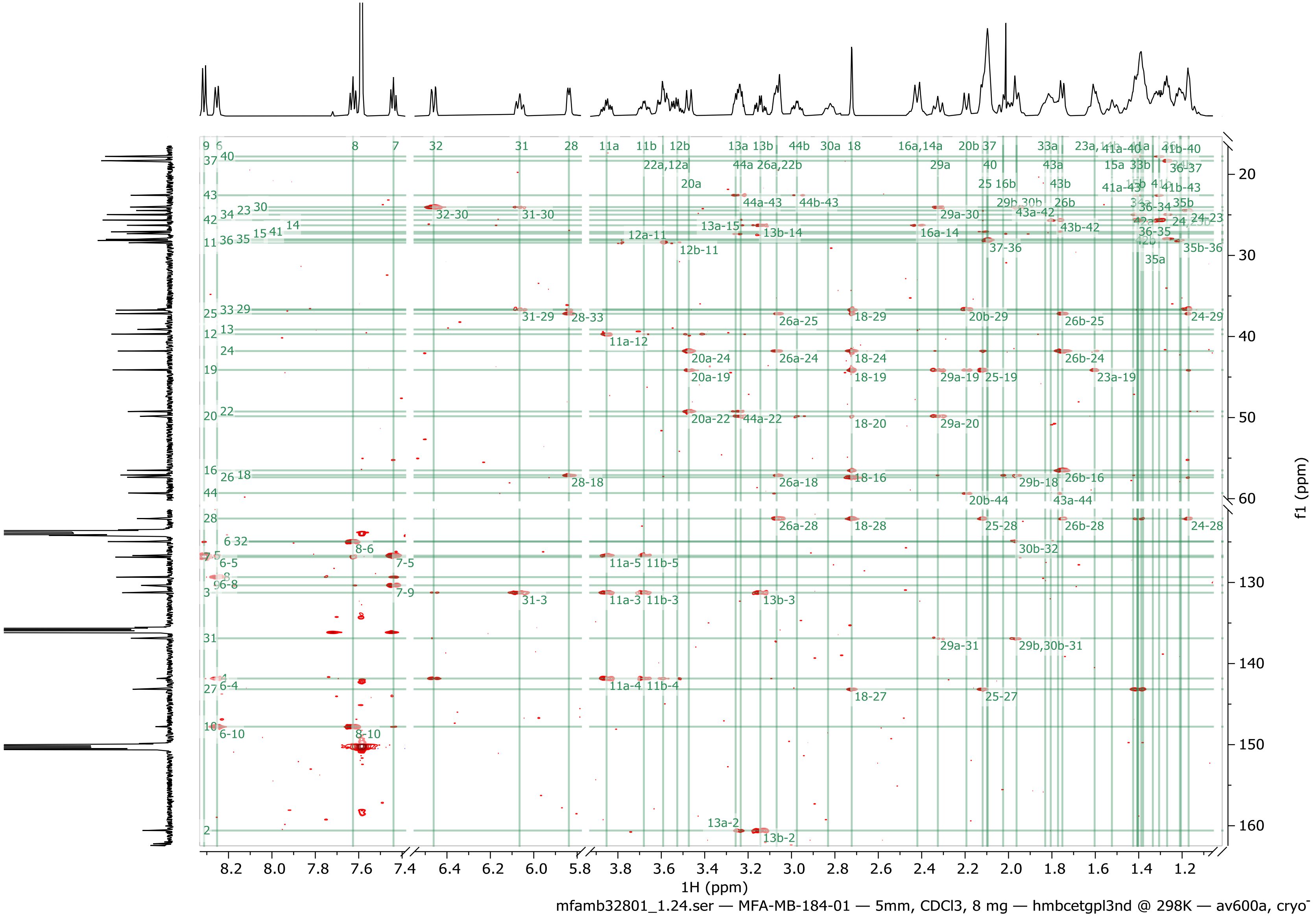

NOESY Spectrum of Njaoamine I (ca. 2.6 mg in [D$_5$]-pyridine, 298 K)

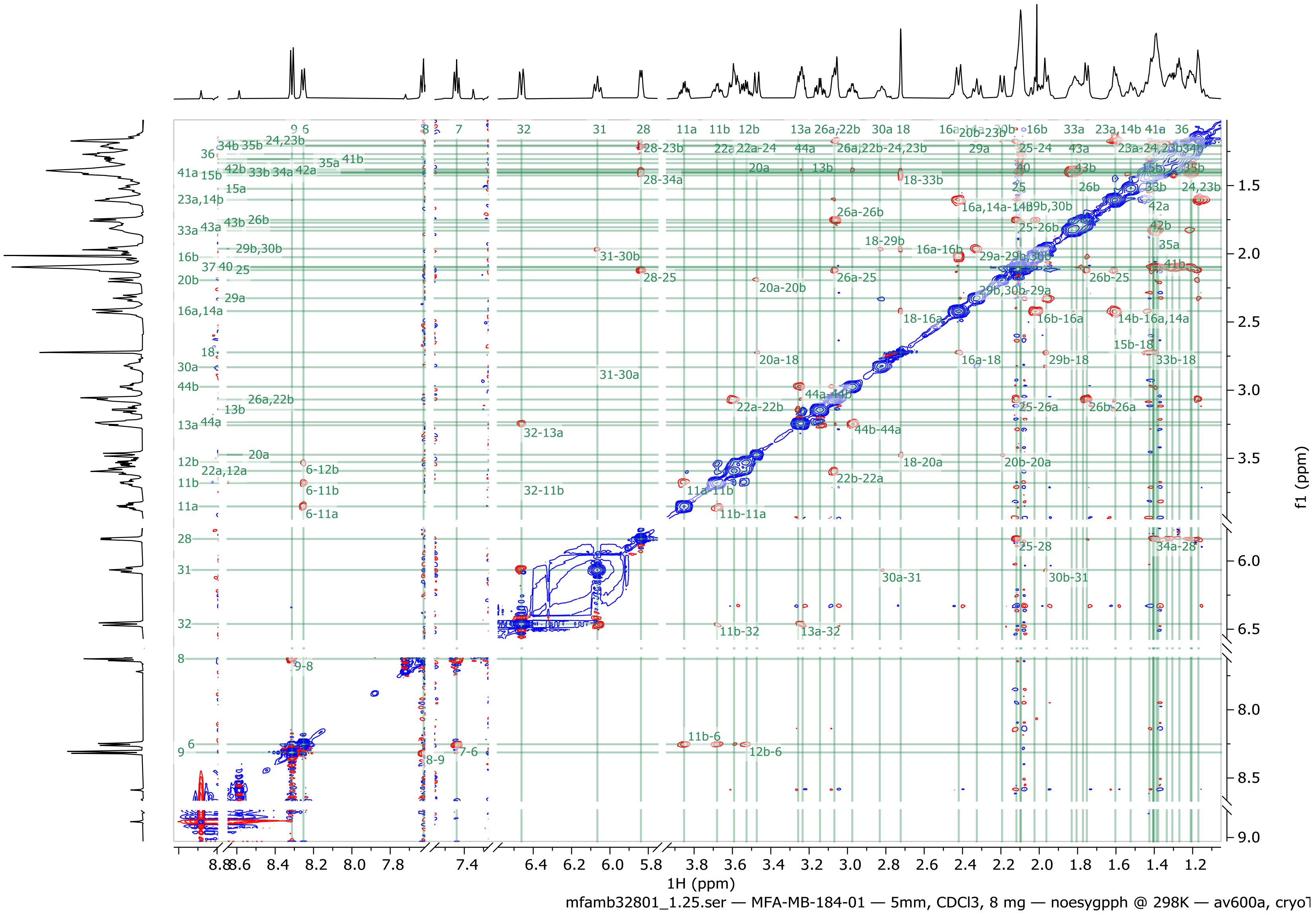

^{15}N-HMBC Spectrum of Njaoamine I (ca. 2.6 mg in [D$_5$]-pyridine, 298 K)

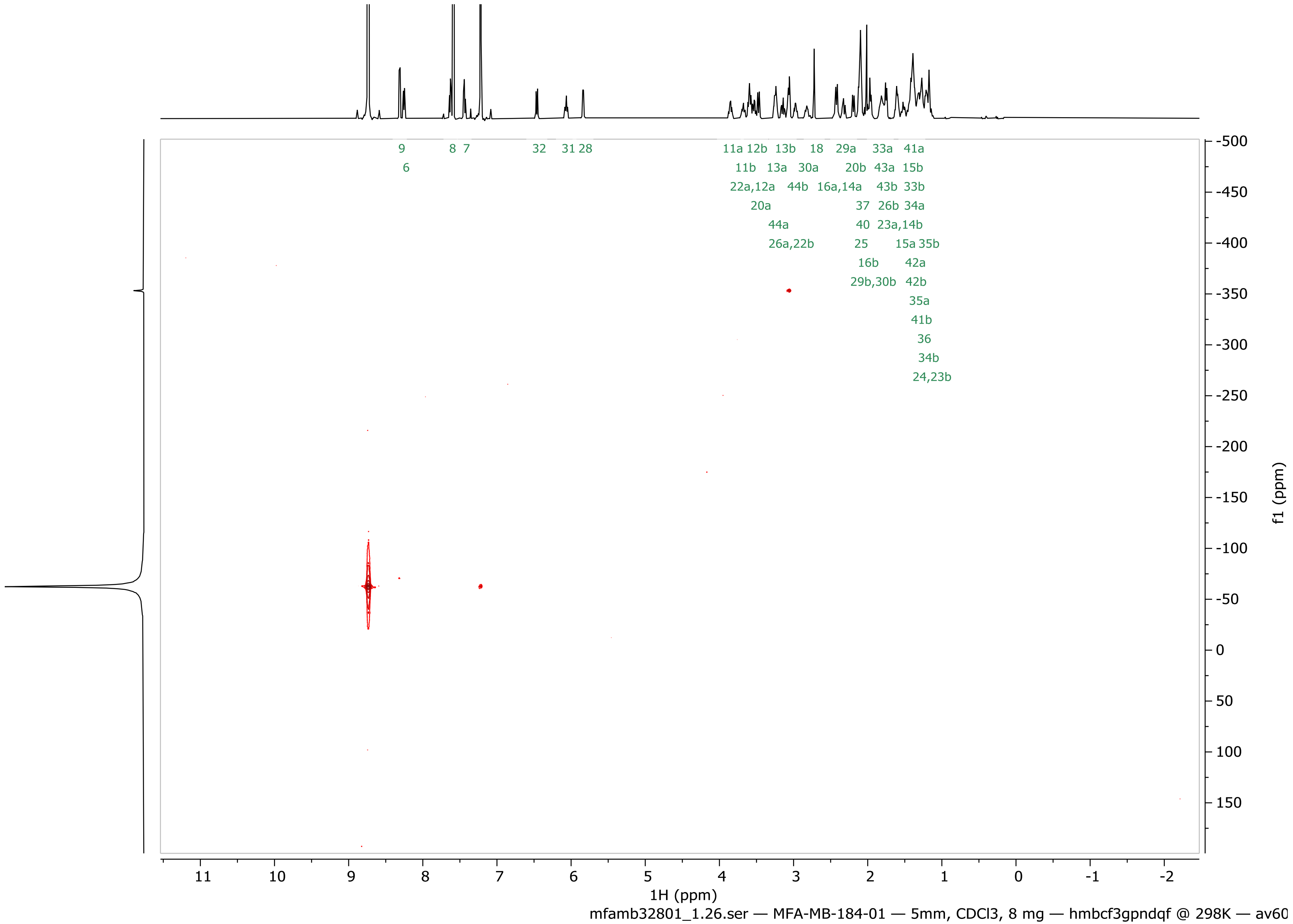

^{1}H NMR Spectrum of 72 (400 MHz, $CDCl_3$)

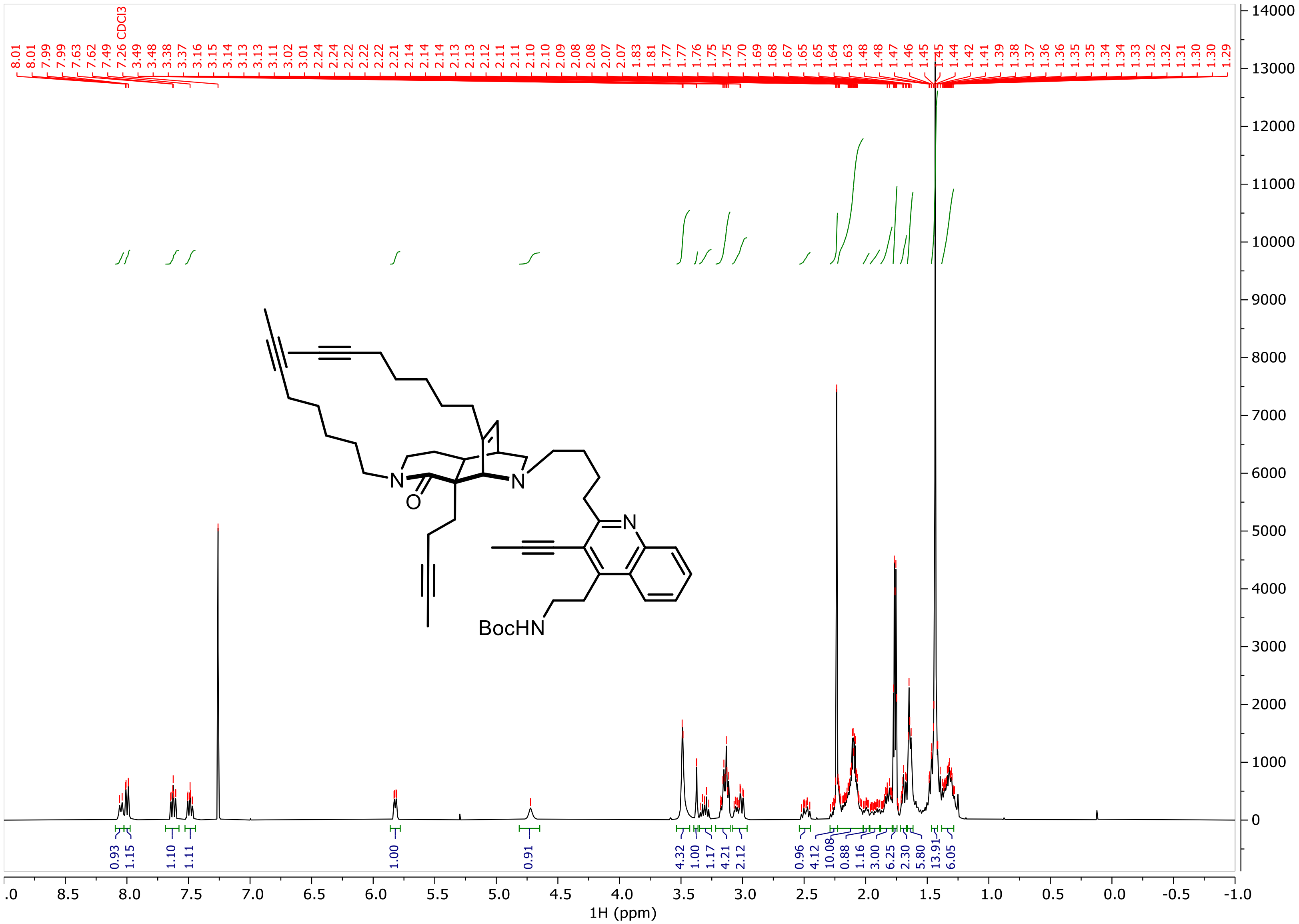

^{13}C NMR Spectrum of 72 (101 MHz, $CDCl_3$)

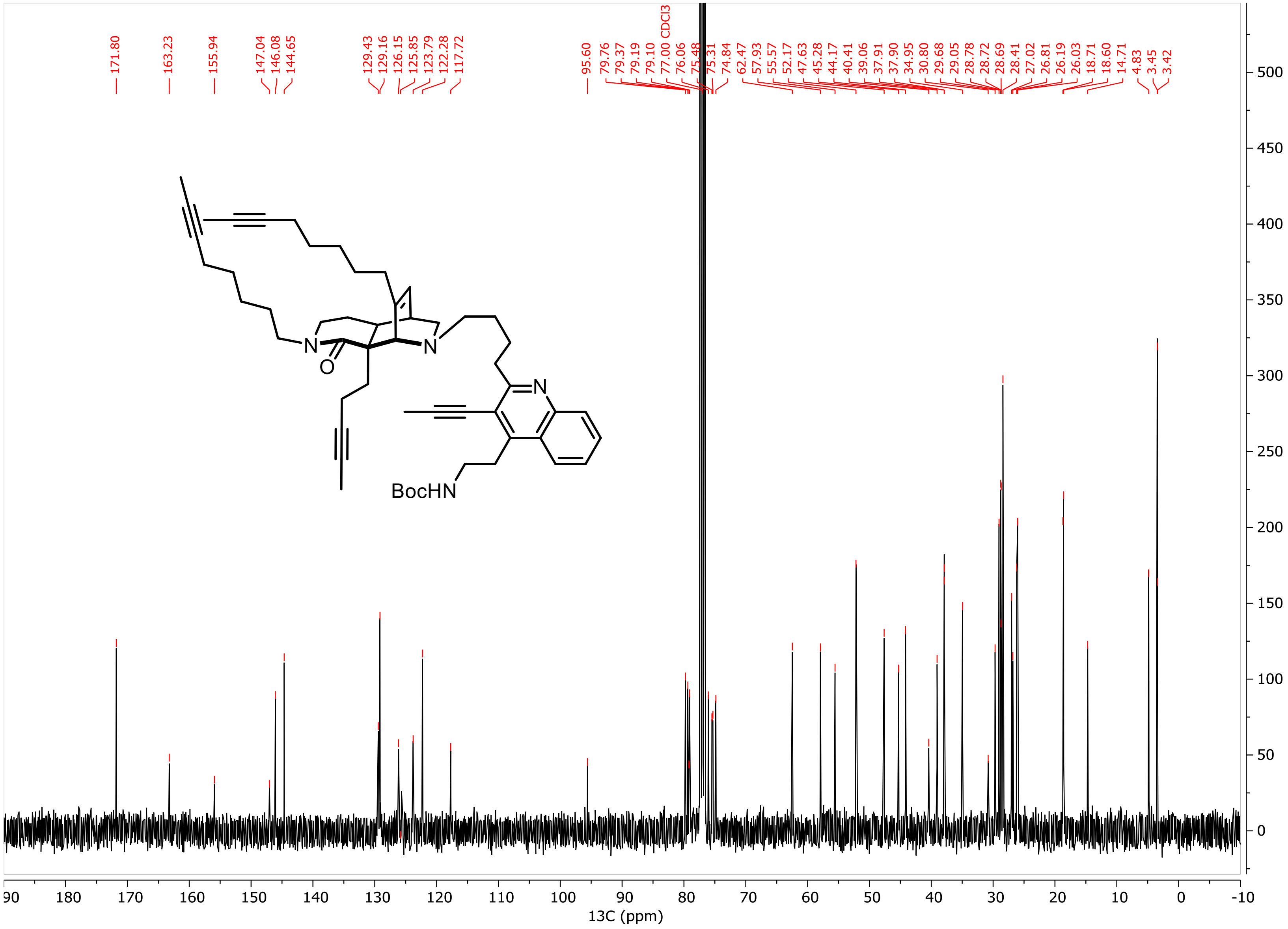

^{1}H NMR Spectrum of 73 (600 MHz, [D$_4$]-MeOH)

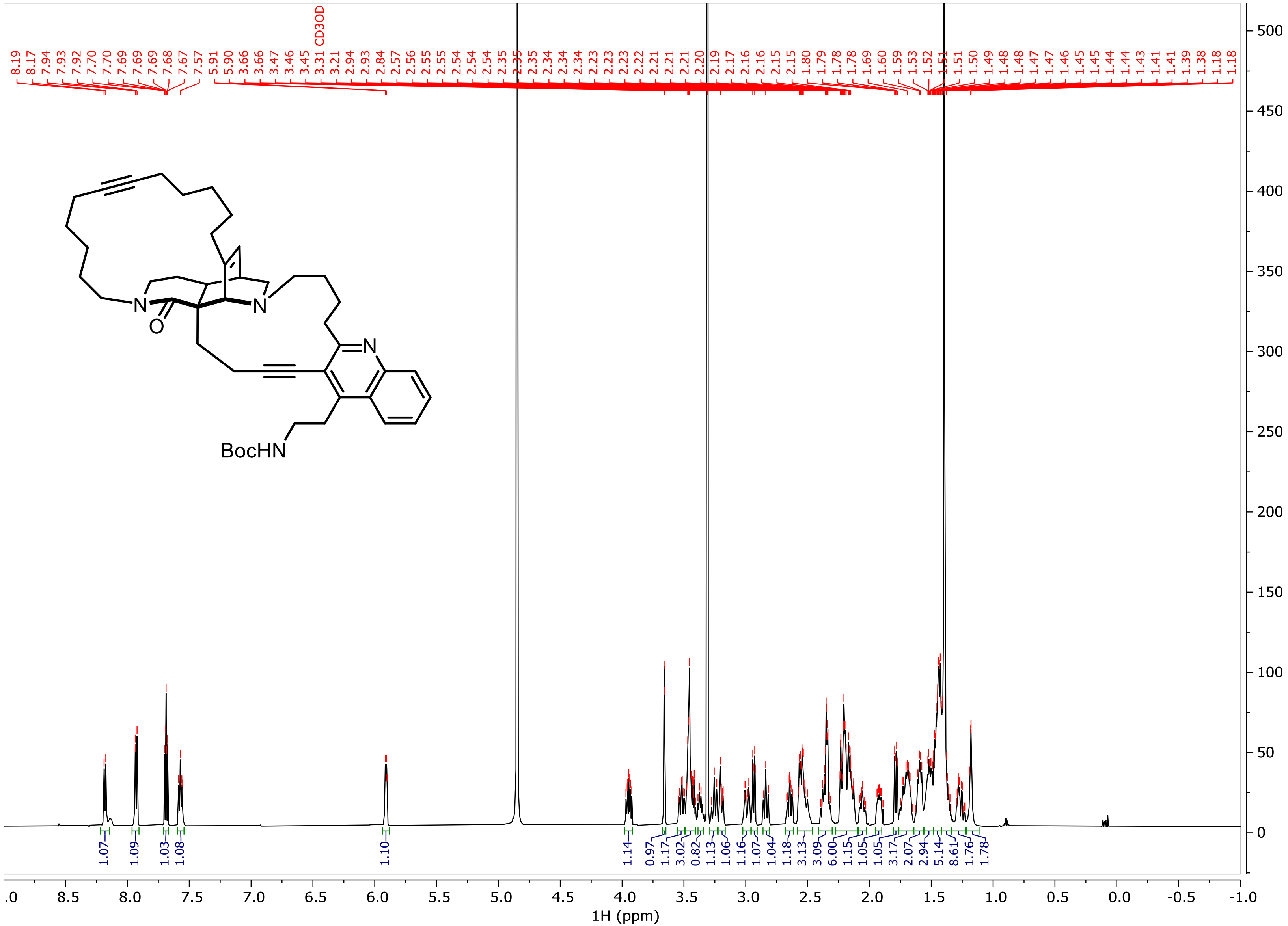

^{13}C NMR Spectrum of 73 (126 MHz, [D$_4$]-MeOH)

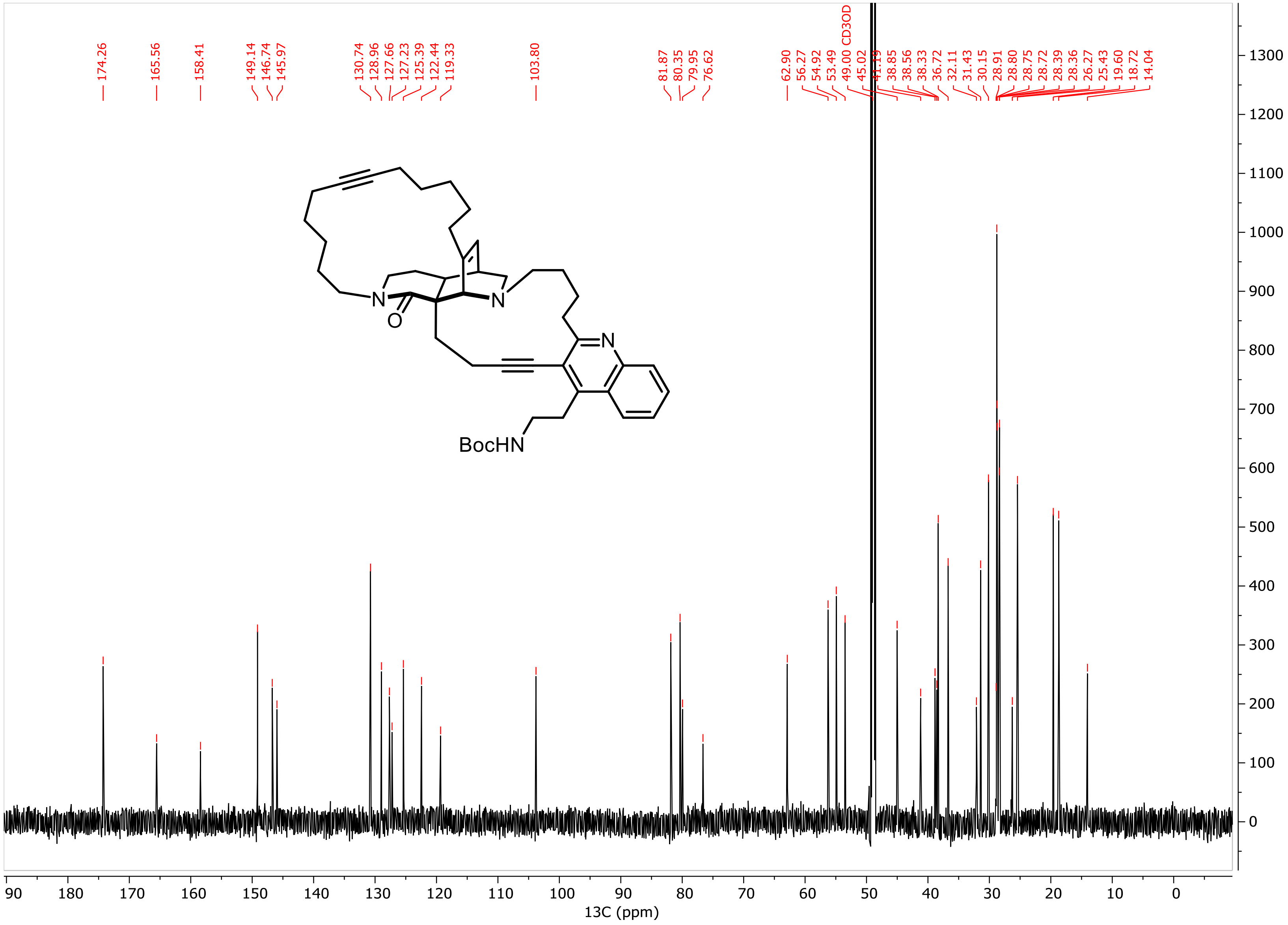

References

1. Hickmann, V.; Alcarazo, M.; Fürstner, A., Protecting-Group-Free and Catalysis-Based Total Synthesis of the Ecklonialactones. *J. Am. Chem. Soc.* **2010,** *132* (32), 11042-11044.
2. Meng, Z.; Fürstner, A., Total Synthesis Provides Strong Evidence: Xestocyclamine A is the Enantiomer of Ingenamine. *J. Am. Chem. Soc.* **2020,** *142* (27), 11703-11708.
3. Hillenbrand, J.; Leutzsch, M.; Yiannakas, E.; Gordon, C. P.; Wille, C.; Nöthling, N.; Copéret, C.; Fürstner, A., "Canopy Catalysts" for Alkyne Metathesis: Molybdenum Alkylidyne Complexes with a Tripodal Ligand Framework. *J. Am. Chem. Soc.* **2020,** *142* (25), 11279-11294.
4. Kaname, M.; Yoshifuji, S.; Sashida, H., An Efficient Transformation of Cyclic Ene-carbamates into m-(N-Formylamino)carboxylic Acids by Ruthenium Tetroxide Oxidation. *Chem. Pharm. Bull.* **2008,** *56*, 1310-1313.
5. Kobayashi, J.; Tsuda, M.; Kawasaki, N.; Matsumoto, K.; Adachi, T., Keramaphidin B, a Novel Pentacyclic Alkaloid form a Marine Sponge Amphimedon sp.: A Plausible Biosynthetic Precursor of Manzamine Alkaloids. *Tetrahedron Lett.* **1994,** *35*, 4383-4386.
6. Kong, F.; Andersen, R. J., Ingenamine Alkaloids Isolated from the Sponge Xestospongia Ingens: Structure and Absolute Configurations. *Tetrahedron* **1995,** *51*, 2895-2906.
7. Baldwin, J. E.; Claridge, T. D. W.; Culshaw, A. J.; Heupel, F. A.; Lee, V.; Spring, D. R.; Whitehead, R. C., Studies on the Biomimetic Synthesis of the Manzamine Alkaloids. *Chem. - Eur. J.* **1999,** *5*, 3154-3161.
8. Sørensen, U. S.; Falch, E.; Krogsgaard-Larsen, P., A Novel Route to 5-Substituted 3-Isoxazolols. Cyclization of N,O-DiBoc β-Keto Hydroxamic Acids Synthesized via Acyl Meldrum's Acids. *J. Org. Chem.* **2000,** *65* (4), 1003-1007.
9. Ghosh, A. K.; Li, J., An Asymmetric Total Synthesis of Brevisamide. *Org. Lett.* **2009,** *11* (18), 4164-4167.
10. Urda, C.; Pérez, M.; Rodríguez, J.; Fernández, R.; Jiménez, C.; Cuevas, C., Njaoamine I, a cytotoxic polycyclic alkaloid from the Haplosclerida sponge Haliclona (Reniera) sp. *Tetrahedron Lett.* **2018,** *59* (26), 2577-2580.
11. Koos, M. R. M.; Kummerlöwe, G.; Kaltschnee, L.; Thiele, C. M.; Luy, B., CLIP-COSY: A Clean In-Phase Experiment for the Rapid Acquisition of COSY-type Correlations. *Angew. Chem. Int. Ed.* **2016,** *55* (27), 7655-7659.
